# Elucidation of the Underlying Mechanism of Gujian Oral Liquid Acting on Osteoarthritis through Network Pharmacology, Molecular Docking, and Experiment

**DOI:** 10.1155/2022/9230784

**Published:** 2022-07-28

**Authors:** Congzi Wu, Qinwen Ge, Zhenyu Shi, Jun Ying, Qi Sun, Shanxing Zhang, Jiali Chen, Pinger Wang, Wenhua Yuan, Songfeng Hu, Hongting Jin, Peijian Tong

**Affiliations:** ^1^Institute of Orthopaedics and Traumatology, The First Affiliated Hospital of Zhejiang Chinese Medical University, Hangzhou, 310053 Zhejiang Province, China; ^2^The First College of Clinical Medicine, Zhejiang Chinese Medical University, Hangzhou, 310053 Zhejiang Province, China; ^3^Department of Orthopaedic Surgery, The First Affiliated Hospital of Zhejiang Chinese Medical University, Hangzhou, 310006 Zhejiang Province, China; ^4^Department of Orthopaedic Surgery, Fuyang Orthopaedics and Traumatology Affiliated Hospital of Zhejiang Chinese Medical University, Hangzhou, 311499 Zhejiang Province, China; ^5^Department of Orthopaedics and Traumatology, Shaoxing Hospital of Traditional Chinese Medicine, Shaoxing, 312099 Zhejiang Province, China

## Abstract

Gujian oral liquid (GJ), a traditional herbal formula in China, has been widely used to treat patients with osteoarthritis (OA). Nevertheless, the active component and potential mechanism of GJ are not fully elucidated. Thus, we investigate the effect of GJ and explore its underlying mechanism on OA through network pharmacology and experimental validation. First, a total of 175 bioactive compounds were identified, and 134 overlapping targets were acquired after comparing the targets of the GJ with those of OA. 8 hub targets, including IL6 and AKT1, were obtained in PPI network analysis. Then, we built up GJ-target-OA network and protein-protein interaction (PPI) network, followed by Gene Ontology (GO) and Kyoto Encyclopedia of Genes and Genomes (KEGG) pathway enrichment analyses. The results underlined inflammatory tumor necrosis factor (TNF) as a promising signaling pathway of GJ for OA treatment. Moreover, molecular docking also verified the top two active compounds had direct bindings with the top three target genes. Finally, we verified the effect of GJ on OA *in vivo* and *in vitro*. *In vivo* experiments validated that GJ not only significantly attenuated OA phenotypes including articular cartilage degeneration and subchondral bone sclerosis but also reduced the expressions of tumor necrosis factor-*α* (TNF-*α*) and p-p65 in articular chondrocytes. Besides, GJ serum also had a protective effect on chondrocytes against inflammation caused by TNF-*α in vitro*. Hence, our study predicted and verified that GJ could exert anti-inflammation and anticatabolism effects partially *via* regulating TNF-*α*/NF-kappa B (NF-*κ*B) signaling.

## 1. Introduction

Osteoarthritis (OA) is a common disease characterized by articular cartilage degeneration, synovial inflammation, and subchondral bone sclerosis, which affects over 250 million individuals worldwide [1, 2]. Although there are several identified risk factors such as age, gender, obesity, and heavy work activities, the exact pathogenesis of osteoarthritis remains unclear [3, 4]. However, low-grade joint inflammation is, in particular, the key mediator for treating OA, because it produces a variety of inflammatory factors such as TNF-*α* and interleukin-1*β* (IL-1*β*) [5].

Since OA affects the whole joint, the osteochondral unit formed by the articular cartilage, calcified cartilage, and subchondral bone remains the primary factor in altering joint function. If any composition or structure of this unit changes, the whole joint would be more likely to lose function like transferring loads in daily activities [6]. Articular cartilage primarily consists of water (60–85%), type II collagen (Col2), and proteoglycans [7]. As an avascular and aneural tissue, articular cartilage has a limited intrinsic capacity for self-healing after destruction or degeneration. Growing evidence indicated that cartilage plays a big part in knee joint homeostasis and OA pathogenesis [8–11]. Besides, the transformation from articular cartilage to calcified cartilage is responsible for aberrant stress distribution and increased bone homeostasis in subchondral bone [12, 13]. Therefore, degradation of articular cartilage and sclerosis of subchondral bone are deemed as two vital indications of OA progression [14]. Although there's plenty of treatment for OA, current mainstream medicine is principally concentrated on reducing symptoms and cannot prevent the disease from developing, let alone reverse its progression [15].

Recently, traditional Chinese medicine (TCM) has been selected as an alternative treatment for osteoarthritis (OA) [16, 17]. OA belongs to the scope of “arthralgia (Bi) syndrome” in TCM. It is mostly caused by the lack of healthy qi in the human body and the affection of three kinds of evil Qi of wind (Feng), cold (Han), and dampness (Shi) from the outside world. Based on this theory, a vast array of TCM formula has been emerged. For example, Gujian oral liquid (GJ), a traditional herbal formula in China, was modified from Xiaodu decoction, which originated in Waike Jingyao from the Song dynasty. GJ has been approved by the Zhejiang Food and Drug Administration to use in patients with OA at The First Affiliated Hospital of Zhejiang Chinese Medical University for decades and provided a good curative effect with safety [18]. It is composed of various traditional Chinese herbs: Astragalus mongholicus Bunge (Huang Qi), Salvia miltiorrhiza Bunge (Dan Shen), Eucommia ulmoides Oliv. (Du Zhong), Angelica sinensis (Oliv.) Diels (Dang Gui), Cuscuta chinensis Lam (Tu Si Zi), and Corydalis yanhusuo (Y.H.Chou and Chun C.Hsu) W.T.Wang ex Z.Y.Su and C.Y.Wu (Yan Hu Suo). It has been reported that this constituent herbs could treat diseases via a variety of mechanisms, such as immunomodulatory [19], anti-inflammatory [20], antioxidant [16], and antalgic [21], suggesting that GJ may exert a therapeutic effect through these mechanisms in pathological conditions.

Therefore, we aim to investigate the effect of GJ and explore its underlying mechanism on OA. For this purpose, we performed network pharmacology analysis, a method broadly used to predict the mechanism and interactions among “formula-target-disease” [22, 23]. Importantly, we have performed *in vivo* and *in vitro* experiments to confirm the predictions from network pharmacology analysis. The graphical abstract is shown in Supplementary Figure 1.

## 2. Material and Methods

### 2.1. Materials and Reagents

GJ was kindly provided by the Zhejiang Chinese Medical University (Hangzhou, China, product batch number: Z20100024) (Table 1). Dulbecco's modified Eagle's medium/F12 (DME/F12) cell medium, fetal bovine serum (FBS), and penicillin/streptomycin were obtained from Sigma (St. Louis, USA). Additionally, BAY 11-7082, an inhibitor of I*κ*B*α* phosphorylation, was purchased from Selleck (Houston, USA). The primary antibodies for Collagen II and MMP-13 were bought from Abcam (Cambridge, UK). IL-1*β* antibody were bought from R&D (Minneapolis, USA). TNF-*α* antibody and phosphorylated-p65 (p-p65) antibody were bought from the Arigo Biolaboratories Corp. (Shanghai, China). The second antibody were acquired from the Invitrogen Corporation (MD, USA).

### 2.2. Screening of Active Ingredients and Target Prediction in GJ

All the components of GJ were screened and constructed with the database of traditional Chinese medicine systems pharmacology (TCMSP, https://old.tcmsp-e.com/tcmsp.php) [24]. In this study, oral bioavailability (OB) was set more than 30% while drug-likeness (DL) was set more than 0.18 to get the candidate ingredients.

The potential targets were also downloaded from the TCMSP databases, and corresponding gene names were matched from the UniProt database (https://www.uniprot.org/uniprot/) using Perl, following removing other target proteins not belonging to *Homo sapiens*.

### 2.3. Searching for Known OA-Related Disease Targets

We used the keyword “osteoarthritis” to search and screen OA-related targets in the GeneCards (https://www.genecards.org/) and OMIM database (http://www.omim.org/); then, we combined all the screening results to get the relevant target information of osteoarthritis. Finally, we employed the “VennDiagram” data package of R version 4.0.3 (2020-10-10) to intersect the disease targets and GJ targets to plot the Venn diagram.

### 2.4. Network Construction and Analysis

#### 2.4.1. GJ-Target-OA Network

GJ-target-OA network was created based on the association between drugs, ingredients, gene symbols, and diseases (OA). Cytoscape software was used to visually analyze the GJ-target-OA network [25]. The top ten key components of the formula and the top 1-2 components of each herb from the “GJ-target-OA” network were screened according to degree values.

#### 2.4.2. PPI Network Construction and Key Targets Screening

We imported the targets obtained from intersection into the STRING online database to form a PPI network as a previous study [26]. Besides, these results were further investigated with Cytoscape. Specifically, we selected degree, betweenness centrality (BC), and closeness centrality to screen hub targets. And degree, BC, and closeness centrality should be greater than or equal to the median value.

#### 2.4.3. GO Function and KEGG Pathway Enrichment Analyses

We used R 4.2.3 clusterProfiler, Bioconductor v3.12 bioinformatics data packages (http://bioconductor.org/) for GO function analysis as well as KEGG pathway enrichment analysis. The *P* value threshold and *q* value were set to 0.05. After being processed using the Perl programming language, the pathway-involved gene ID was converted into gene names.

### 2.5. Molecular Docking

Firstly, the chemical structures of the top hub targets of the formula and each herb were downloaded from the PubChem database. Secondly, we downloaded the three-dimensional (3D) structures of IL-6 (PDB ID: 1ALU) and AKT1 (PDB ID: 4GV1) from PDB (http://www.rcsb.org/pdb). Thirdly, the ligands (the core compounds) and the receptors (the core target genes) were processed and docked in AutoDock4. Finally, the results were analyzed and presented using PyMOL (http://www.pymol.org).

### 2.6. Ultraperformance Liquid Chromatography (UPLC) Analysis

UPLC analysis of GJ was performed as a quality control. A Waters ACQUITY UPLC H-Class coupled with an ACQUITY UPLC ®BEH C18 column maintained at 30°C was used. The flow rate was 0.4 mL/min, and the detection wavelength and the injection volume were 280 nm and 2 *μ*L, respectively. The mobile phase consisted of acetonitrile and 0.1% phosphoric acid in water (pH = 5.6), and gradient elution was set as follows: 0–1 min, 5% A−10% A; 1–3 min, 10% A−20% A; 3–5 min, 20% A−28% A; 5–8 min, 28% A−30% A; 8–10 min, 30% A−40% A; 10–12 min, 40% A−80% A; 12–15 min, 80% A−80% A; 15–15.1 min, 80% A−10% A; 15–16 min, 10% A−10% A.

### 2.7. Animals and Experiments

10-week-old C57BL/6J male mice were obtained from the Shanghai Laboratory Animal Co., Ltd. (Shanghai, China) and divided as sham group, model group, and GJ group, with 10 mice in each group. Briefly, the model and GJ group mice were anesthetized with pentobarbital sodium (0.4 mg/10 g) by intraperitoneal injection. Then, we performed DMM surgery to establish the OA model as described previously [27]. Similar surgery for mice in sham group but did not expose the articular capsule. After DMM-operation, mice in GJ group were administrated with GJ (0.3 mL/20 g) by oral gavage once a day for 8 weeks, based on the formula of human-mouse equivalent dosage conversion [17]. The mice in sham and model groups were treated with saline at an equal dosage. All animal experiments in the present study were performed following institutional guidelines and approval by the Animal Care and Use Committee of Zhejiang Chinese Medical University.

### 2.8. Gait Analysis

According to relevant study [28], gait analysis was performed by DigiGait Imaging System (Boston, USA). Briefly, a video camera in the machine captured ventral images of four paws. Mice ran on a transparent flat treadmill at a speed of 18 cm/s for up to 30 seconds for each measurement. And the segment for analysis is 5 seconds. Stance width and paw area were measured automatically by the DigiGait image analysis software as previously published [29].

### 2.9. Micro-CT (*μ*CT) Evaluation

Right knee samples were scanned with *μ*CT (Skyscan1176, Belgium) at a high resolution of 8.7 *μ*m with a 45 kVp source and 500 mA current. The three-dimensional (3D) structure of the knee was reconstructed using NRecoN software. We measured quantitative morphometric analysis of subchondral bone about subchondral bone volume (BV, mm^3^), bone volume fraction (BV/TV), trabecular separation (Tb. Sp), and trabecular thickness (Tb. Th) as previously described [30].

### 2.10. Histological Analyses

The right knees were fixed with 4% paraformaldehyde (PFA) solution for three days and then placed in 14% ethylene diamine tetraacetic acid (EDTA) for two weeks. We then embedded these samples in paraffin and sectioned sagittally at three *μ*m. Here, we carried out Alcian blue hematoxylin/Orange G (ABH/OG) staining to investigate the cartilage degeneration. To quantificationally evaluate the histopathology of OA, both cartilage area and cartilage thickness were calculated quantificationally by Image J software. Additionally, two independent observers score the structure degeneration of cartilage using the OARSI histology scoring system [31].

### 2.11. Immunohistochemistry (IHC) and Immunofluorescence (IF) Analysis

For IHC, the slices were immersed in citrate buffer at 60°C for 4 hours following deparaffinization and rehydration. After that, we applied 0.5% Triton X-100 to the slices for permeabilization and followed by blocking the endogenous peroxidase activity for 10 min. Subsequently, sections were incubated with primary antibodies at 4°C overnight. On the next day, all the sections were incubated with second antibody for 30 minutes. Positive signal staining of sections was detected by diaminobenzidine and counterstained with hematoxylin for 20 s. For IF, the sections were incubated with the fluorescent secondary antibody for one hour in the dark the next day, then counterstained with DAPI for 5 minutes. The percent of positive expression was calculated by Image J software.

### 2.12. GJ-Containing Serum Preparation

For preparation of GJ-containing serum, 20 Sprague-Dawley male rats were randomly divided into two groups: control group (*n* = 10) and GJ group (*n* = 10). GJ group rats were treated with GJ (10 mL/kg) once a day by oral gavage for 7 days, while rats in control group were given the same amount of saline. One hour after the last gavage, the blood was collected from rats by cardiac puncture and being stood for 1 hour and then centrifuged at 3000 rpm for 10 minutes to isolate the supernatant serum. The collected serum was sterilized and stored at -80°C for later use.

### 2.13. Cell Culture

The isolation of chondrocytes was processed as described previously [32]. Briefly, the primary chondrocytes were isolated from two-week-old C57BL/6J mice. We isolated the hip articular cartilage aseptically and then digested the cartilage tissue with digestion medium containing Collagenase P (0.25 mg/mL, ROCHE) for 6 hours at 37°C. After digestion, we used a 70 *μ*m cell strainer to remove residue of cartilage. Finally, chondrocytes were plated in 6- or 96-well plates (2 × 10^5^ cells/well or 1 × 10^4^ cells/well, respectively) and incubated in cell medium DMEM/F-12 which containing 10% FBS and 1% streptomycin/penicillin for 48 hours. Then, the medium was changed with DMEM/F12 containing only 10% FBS. All cells were cultured in cell incubator at 37°C with 5% CO_2_.

### 2.14. Cell Counting Kit-8 (CCK-8) Assay

In this study, chondrocyte viability was determined by CCK-8. Briefly, primary chondrocytes were seeded in 96 well plates with a density of 1 × 10^4^ cells per well and treated with gradient concentration of the GJ or control serum. After 12 and 24 hours, we added the CCK-8 reagents for another 2 hours in dark and then measured the absorbance wavelength at 450 nm with a microplate reader.

### 2.15. Quantitative RT-PCR

Total RNA was extracted from primary mouse chondrocytes with TRIzol reagent. We used a cDNA Synthesis Kit (Bimake, Beijing) to reverse transcribe according to the manufacturer's instructions. And RT-PCR was performed using the SYBR Premix Ex Taq™ II kit (Takara, Dalian, China). The primer sequences of target gene are shown in Table 2. We employed *β-actin* as a reference gene in this study.

### 2.16. Statistical Analysis

The Shapiro–Wilk normality test was used to analyze whether the data were normally distributed. All the data were shown as means ± standard deviation and statistically analyzed by using GraphPad Prism software (version 9). One-way ANOVA followed by Tukey's multiple comparisons test was carried out in this study. The *P* value < 0.05 was considered statistically significant.

## 3. Results

### 3.1. Screening of GJ Bioactive Compounds and OA Therapeutic Targets

**Figure 1:**
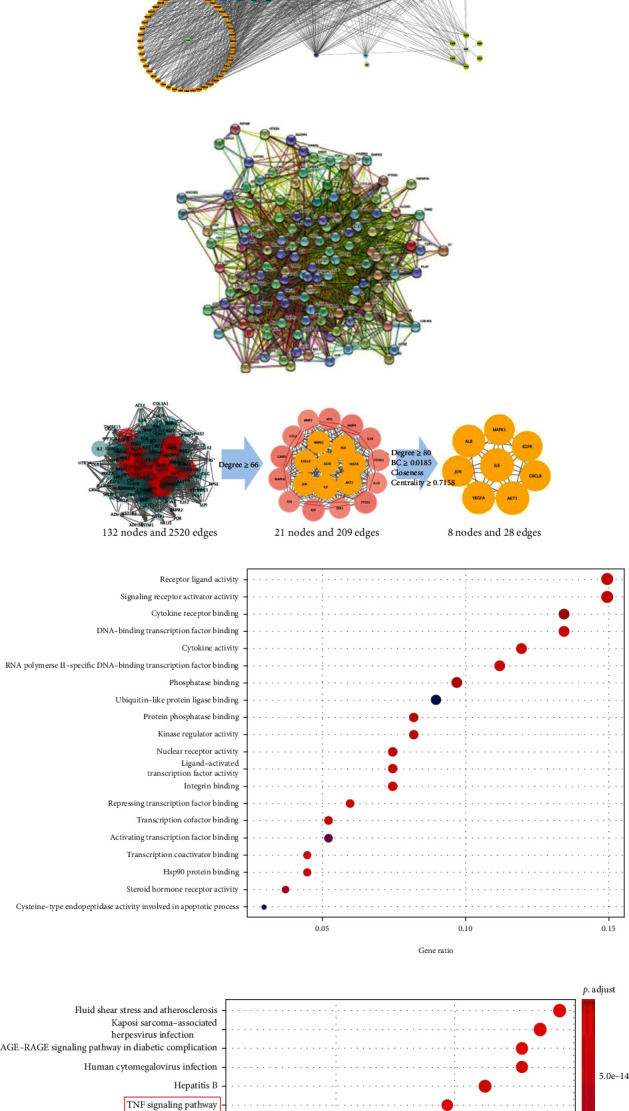
Network construction and GO and KEGG enrichment analysis. (a) Common targets between GJ and osteoarthritis. (b) GJ-target-OA network. The GJ-target-OA network was constructed by linking potential compounds of GJ to their putative targets. The polychromatic hexagons represented potential compounds (A1-G1 were common compounds among different herbs) and the blue diamonds indicated the targets. (c) PPI network of 134 common targets (d) and 8 hub targets. (e) GO enrichment analysis of 134 common targets. (f) KEGG pathway enrichment analysis of top 20 pathways. The red box indicates that TNF signaling pathway is in the top 10.

Firstly, the results of UPLC analyses suggested a good reproducibility for GJ (Supplementary Document S1). Then, according to the ADME thresholds of OB ≥ 30% and DL ≥ 0.18, 175 bioactive compounds (Supplementary Table S1) and 280 compound-related targets (Supplementary Table S2) were obtained from the TCMSP databases. Then, the targets were transformed using the UniProt database. A total of 230 targets (Supplementary Table S3) were determined, of which HQ, DS, DZ, DG, TSZ, and YHS targeted 180, 10, 182, 40, 188, and 184 genes, respectively. A total of 2984 OA-related therapeutic targets were obtained from the GeneCards and OMIM databases (Supplemetary Table S4). After comparing the targets of the GJ with those of the disease, 134 overlapping targets (Supplementary Table S5) were acquired, as the Venn diagram (Figure 1(a)) showed.

### 3.2. Construction of GJ-Target-OA Network and PPI Networks

We constructed an OA target network using Cytoscape to identify the relationships between 6 herbs of GJ and their corresponding compounds and OA common targets (Figure 1(b) and Supplementary Table S6). The polychromatic hexagons represent candidate compounds (A1-G1 are common compounds among different herbs) and the blue diamonds indicate the targets. The edges indicate that nodes can interact with each other. Quercetin, kaempferol, and beta-sitosterol were the top three critical components from the “GJ-target-OA” network screened according to degree values, while stigmasterol, 7-O-methylisomucronulatol, epiquinidine, (S)-coulerine, sesamin, and 3-hydroxymethylenetanshinquinone were the top of each herb (Supplementary Table S7).

Next, 134 common targets were imported into the String database to establish a PPI network, leaving 132 nodes and 2520 edges after removing 2 disconnected nodes (Figure 1(c) and Supplementary Table S8). Key targets were selected according to degree, BC, and closeness centrality to establish the most related targets of GJ for KOA treatment (Supplementary Table S9). Degree ≥ 66 was set during the first round of screening, and we obtained 21 center nodes from this round. Then, the degree ≥ 80, BC ≥ 0.0185, and closeness centrality ≥ 0.7158 were set for the second round of screening, and 8 large central nodes were obtained in the end (Figure 1(d)). The results showed that IL6 and AKT1, the top two genes, are separately linked to other 103 and 100 proteins, respectively.

### 3.3. GO Enrichment Analyses and KEGG Pathway Analyses

GO enrichment analysis of putative targets was performed to clarify the relevant biologic processes of GJ on OA (*P* < 0.01) (Supplementary Table S10). Here, the top 20 terms were chosen based on their *P* value (Figure 1(e)). The results indicated that several biological processes were involved in the antiosteoarthritis effects of GJ, including receptor-ligand activity (GO:0048018), signaling receptor activator activity (GO:0030546), cytokine receptor binding (GO:0005126), and DNA-binding transcription factor binding (GO:0140297).

To further elucidate the potential pathways involved in the antiosteoarthritis effects of GJ, KEGG pathway enrichment analysis of the 134 common genes was performed (Supplementary Table S11). Similarly, the top 20 enriched pathways involved in the antiosteoarthritis effects of GJ were screened (Figure 1(f)). The enriched genes were linked to various signaling pathways, including TNF signaling pathway (hsa04668), IL-17 signaling pathway (hsa04657), and Toll-like receptor signaling pathway(hsa04620), suggesting that these pathways may mediate the antiosteoarthritis effects of GJ.

### 3.4. Results of Molecular Docking

**Figure 2:**
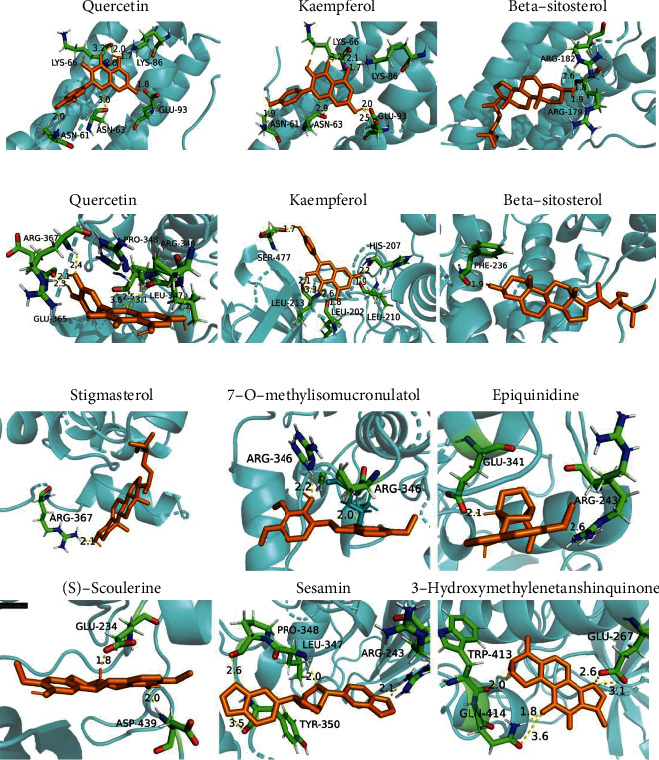
Molecular docking of key protein with quercein, kaempferol, and beta-sitosterol. (a) IL6 with quercetin, kaempferol, and beta-sitosterol. (b) AKT1 with quercetin, kaempferol, and beta-sitosterol. (c) AKT1 with stigmasterol, 7-O-methylisomucronulatol, epiquinidine, (S)-coulerine, sesamin, and 3-hydroxymethylenetanshinquinone. Orange represents quercetin, kaempferol, beta-sitosterol, stigmasterol, 7-O-methylisomucronulatol, epiquinidine, (S)-coulerine, sesamin, or 3-hydroxymethylenetanshinquinone, blue represents IL6 or AKT1, and the dotted lines indicate the hydrogen bonds with distance. Atoms C, N, and O are green, blue, and red, respectively.

We then docked the main components with corresponding targets by AutoDock according to the results of network pharmacology. As shown in Figure 2, the nine core compounds (quercetin, kaempferol, beta-sitosterol, stigmasterol, 7-O-methylisomucronulatol, epiquinidine, (S)-coulerine, sesamin, and 3-hydroxymethylenetanshinquinone) and protein crystal structures corresponding to the core target genes (IL6 and AKT1) are bound in the form of at least one hydrogen bond. For example, we found that quercetin occupied the hydrophobic pocket composed of the residues LYS-66 (bond length: 3.2 Å and 2.0 Å), LYS-86 (bond length: 1.7 Å), GLU-93 (bond length: 1.8 Å), ASN-63 (bond length: 3.0 Å), and ASN-61 (bond length: 2.0 Å) in IL6 (Figure 2(a)). Besides, the binding energy (affinity) of molecular docking is an important index to evaluate the binding ability between ligand and receptor. The binding energies were obtained, and all of them were less than -5 kcal/mol, suggesting that the compounds and target genes can bind well (Supplementary Table S11). Thus, the interactions between these inflammatory markers and core compounds indicated the anti-inflammatory effects of GJ (Supplementary Table S12).

### 3.5. GJ Reduced Aberrant Subchondral Bone Remodeling and Ameliorates Abnormal Gait

**Figure 3:**
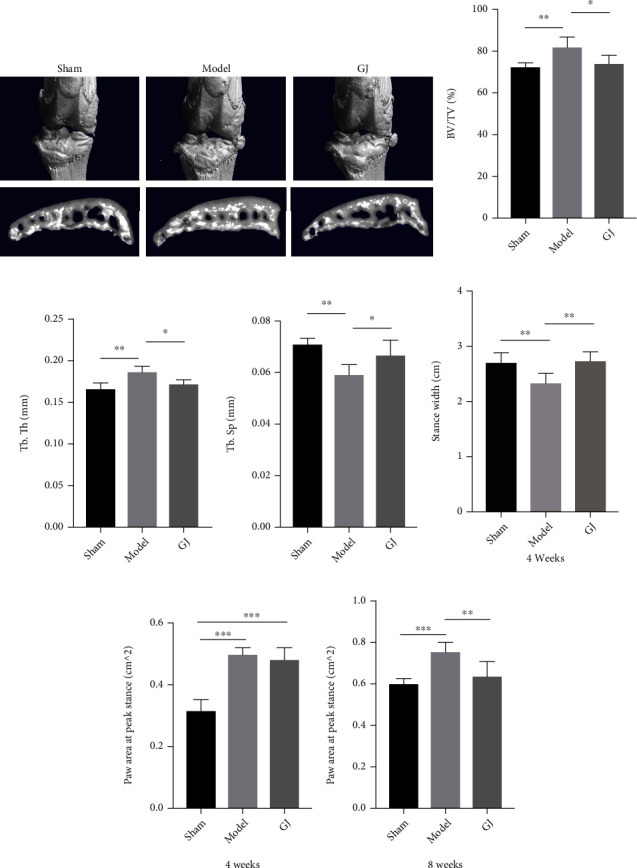
GJ reduced aberrant subchondral bone remodeling and ameliorated abnormal gait. (a) Representative 3D reconstructed *μ*-CT images and (b–d) quantitative analysis of mouse tibia subchondral bone plates from the sham, model, and GJ-treated mice 8 weeks after surgery. (e–g) The gait pattern of mice was analyzed at 4 weeks and 8 weeks post DMM surgery using DigiGait test. All data were shown as means ± SD (*n* = 6‐10). ^∗^*P* < 0.05, ^∗∗^*P* < 0.01, ^∗∗∗^*P* < 0.001 by one-way ANOVA.

To examine the effect of GJ on the microarchitecture of subchondral bone and gait, we performed the microcomputed tomography (*μ*CT) scanning and DigiGait test. Representative *μ*CT images showed that the tibial subchondral bone volume in DMM-induced mice dramatically increased relative to the sham group, while the GJ group reversed this trend (Figure 3(a)). Nevertheless, model and GJ-treated mice had little difference in osteophyte formation. Quantitative analyses showed that both BV/TV and Tb. Th were significantly decreased while Tb. Sp was increased in GJ mice compared to that of model mice by 8 weeks postsurgery (Figures 3(b)–3(d)). These results indicated that GJ could ameliorate the development of subchondral bone sclerosis.

Moreover, quantitative analysis of the DigiGait test showed that StanceWidth was shortened in the model group relative to the sham group 4 weeks postsurgery, while paw area at peak stance was both increased at 4 weeks and 8 weeks postsurgery (Figures 3(e)–3(g)). Notably, GJ treatment could modify these results, revealing that GJ may exert a protective effect on abnormal gait change.

### 3.6. GJ Protected against Articular Cartilage Degeneration and Inflammation in DMM-Induced Mice

**Figure 4:**
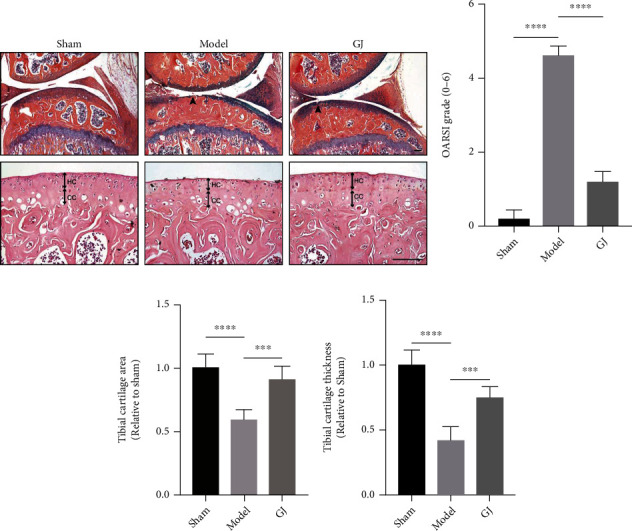
GJ protected against articular cartilage degeneration in DMM-induced mice. (a) ABH/OG staining (50x) and H&E staining (200x) of the tibia medial compartment. Arrows indicate the collapse of cartilage at 8 weeks postsurgery. Hyaline cartilage (HC) and calcified cartilage (CC) thickness were indicated by double arrowed lines. Scale bar = 100 *μ*m. Quantitative analysis of (b) OARSI scoring, (c) tibial cartilage area, and (d) tibial cartilage thickness. All data were shown as means ± SD (*n* = 6‐10). ^∗∗∗^*P* < 0.001, ^∗∗∗∗^*P* < 0.0001 by one-way ANOVA.

**Figure 5:**
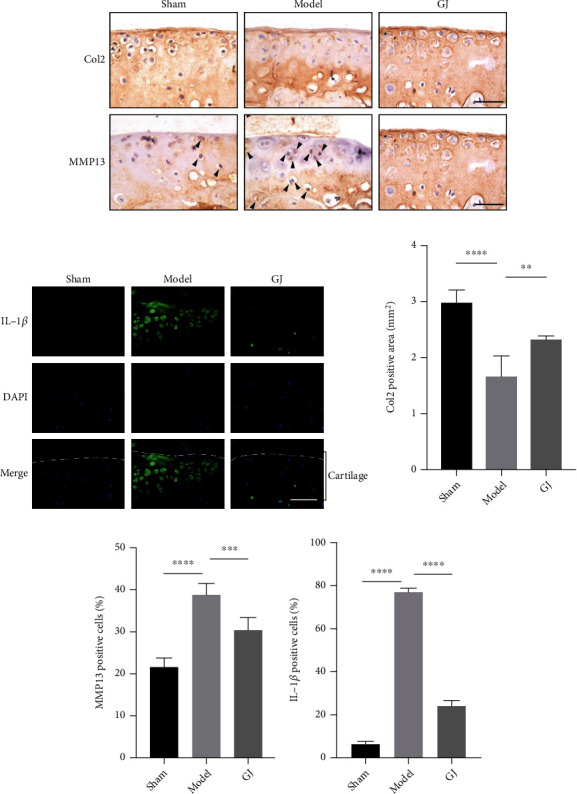
Effects of GJ on expressions of Col2, MMP13, and IL-1*β* of cartilage in DMM-induced mice. (a) IHC staining of col2 and MMP13 (400x). Arrows indicated positive expressions. Scale bar = 50 *μ*m. (b) Immunofluorescence staining of IL-1*β* in mouse tibial cartilage after DMM surgery (400x). Scale bars = 50 *μ*m. (c–e) Quantification of positive expressions of col2, MMP13, and IL-1*β*. All data were shown as means ± SD (*n* ≥ 6). ^∗∗^*P* < 0.01, ^∗∗∗^*P* < 0.001, ^∗∗∗∗^*P* < 0.0001 by one-way ANOVA.

ABH/OG staining and HE stainings were performed to investigate the morphology of articular cartilage, which could reflect cartilage degradation (Figure 4(a)). Specifically, the treatment of GJ significantly reduced all DMM-induced manifestations of OA, including articular cartilage destruction, subchondral bone sclerosis, and calcification of the articular cartilage. HE staining exhibited that GJ group maintained almost normal hyaline cartilage (HC) and calcified cartilage (CC) thickness whereas the thickness of CC increased in DMM group as the tidemark moved closer to the articular surface. OARSI scores also revealed the degeneration of articular cartilage post DMM, while the GJ-treated group decreased these scores (Figure 4(b)). Besides, the GJ-treated group exhibited significantly thicker articular cartilage and larger cartilage area relative to DMM-induced mice (Figures 4(c) and 4(d)). The effect of GJ on articular cartilage homeostasis was further confirmed by the higher expression of type II collagen (Col2) and lower MMP13 compared to DMM-induced mice (Figures 5(a), 5(c), and 5(d)). Importantly, the expression of IL-1*β* in the articular cartilage was increased in response to DMM surgery, yet this upregulation was significantly suppressed by GJ (Figures 5(b) and 5(e)). Together, these results suggested GJ could ameliorate DMM-induced cartilage degradation and OA-like inflammation.

### 3.7. GJ Contributed to Inhibiting Activation of TNF-*α*/(NF-Kappa B) NF-*κ*B Signaling in DMM-Induced Mice and Chondrocytes

**Figure 6:**
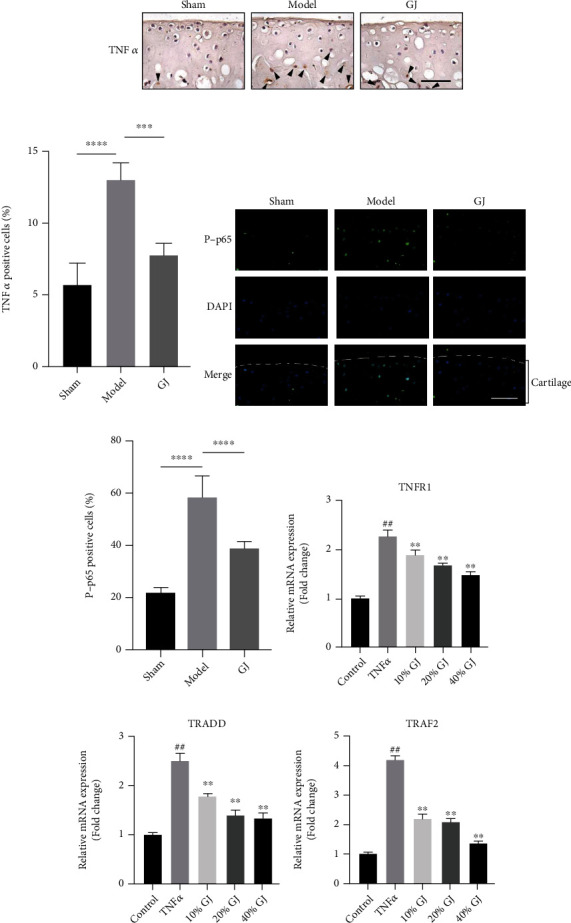
GJ inhibited DMM-induced TNF-*α*/NF-*κ*B inflammatory signaling. (a) Immunohistochemical staining of TNF-*α* (400x). Arrows denoted positive expressions. Scale bar = 50 *μ*m. (c) Immunofluorescence staining of p-p65 in mouse tibial cartilage after DMM surgery (400x). Scale bars = 50 *μ*m. (b, d) Quantification of the percentage of positive cells. Data were shown as means ± SD (*n* ≥ 6). ^∗∗∗^*P* < 0.001, ^∗∗∗∗^*P* < 0.0001 by one-way ANOVA. (e–g) The TNFR1 (e), TRADD (f), and TRAF2 (g) mRNA expression in control and TNF-*α*-induced (10 ng/mL) chondrocytes treated with different concentrations of GJ serum for 24 hours. Data were shown as means ± SD (*n* = 3). ^##^*P* < 0.01 vs. control group, ^∗∗^*P* < 0.01 vs. model group.

According to the results of network pharmacology and KEGG pathway analyses, we obtained several potential pathways which might closely link to the role of GJ in OA. However, considering that TNF was important in OA and members of the TNF family are the best-characterized inducers of NF-*κ*B amongst cytokines, we next used immunohistochemistry (IHC), immunofluorescence (IF), and RT-PCR to verify whether the effect of GJ was related to the TNF-*α*/NF-*κ*B signaling. As expected, expression of TNF-*α* was downregulated in tibial articular cartilage and subchondral bone from GJ-treated mice (Figures 6(a) and 6(b)). Besides, TNF-*α* increased the expressions of TNFR1, TRADD, and TRAF2 in chondrocytes in vitro, which was inhibited by pretreatment with different concentrations of GJ for 24 hours (Figures 6(e)–6(g)). Furthermore, as p-p65 is the primary transcription factor of NF-*κ*B signaling activation, IF result showed that the positive expression of p-p65 in the cartilage area was elevated in DMM-induced mice, which was significantly repressed following GJ intervention (Figures 6(c) and 6(d)). These results indicated that GJ treatment could inhibit the activation of TNF-*α*/NF-*κ*B signaling during OA development.

### 3.8. GJ Reduced the Catabolism and Inflammation of Chondrocytes Induced by TNF-*α* Treatment In Vitro

**Figure 7:**
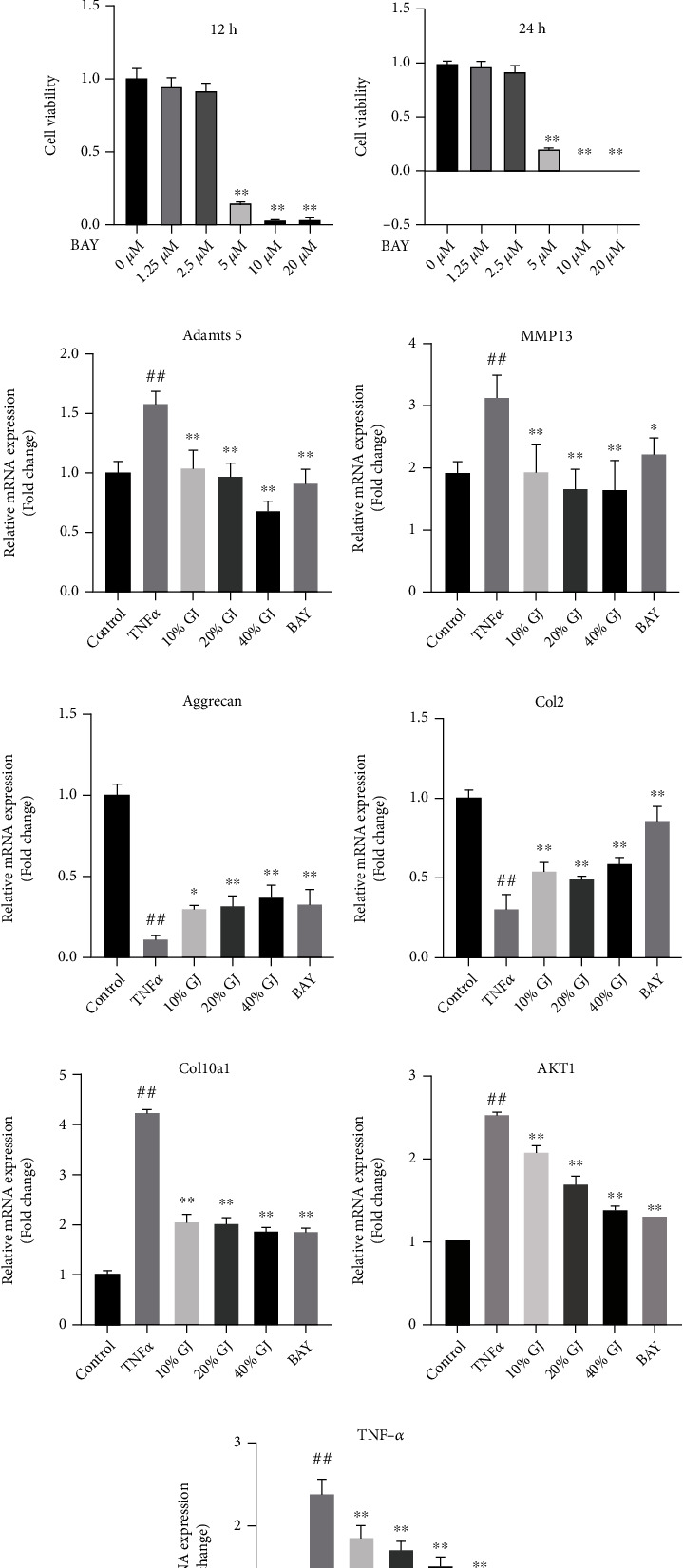
The effect of GJ serum on TNF-*α*-induced chondrocytes *in vitro*. (a, b) Chondrocytes viability treated with different concentrations of GJ serum (2.5%, 5%, 10%, 20%, and 40%) for 12 hours and 24 hours. (c, d) Chondrocyte viability treated with different concentrations of BAY 11-7082 for 12 hours and 24 hours. (e–j) The adamts5 (e), MMP13 (f), aggrecan (g), col2 (h), col10a1 (i), AKT1(j), and TNF-*α* (k) mRNA expression in control and TNF-*α*-induced (10 ng/mL) chondrocytes treated with different concentrations of GJ serum and BAY 11-7082 (2 *μ*M) for 24 hours. All the data were shown as means ± SD (*n* = 3). ^##^*P* < 0.01 vs. control group, ^∗^*P* < 0.05 vs. model group, and ^∗∗^*P* < 0.01 vs. model group.

**Figure 8:**
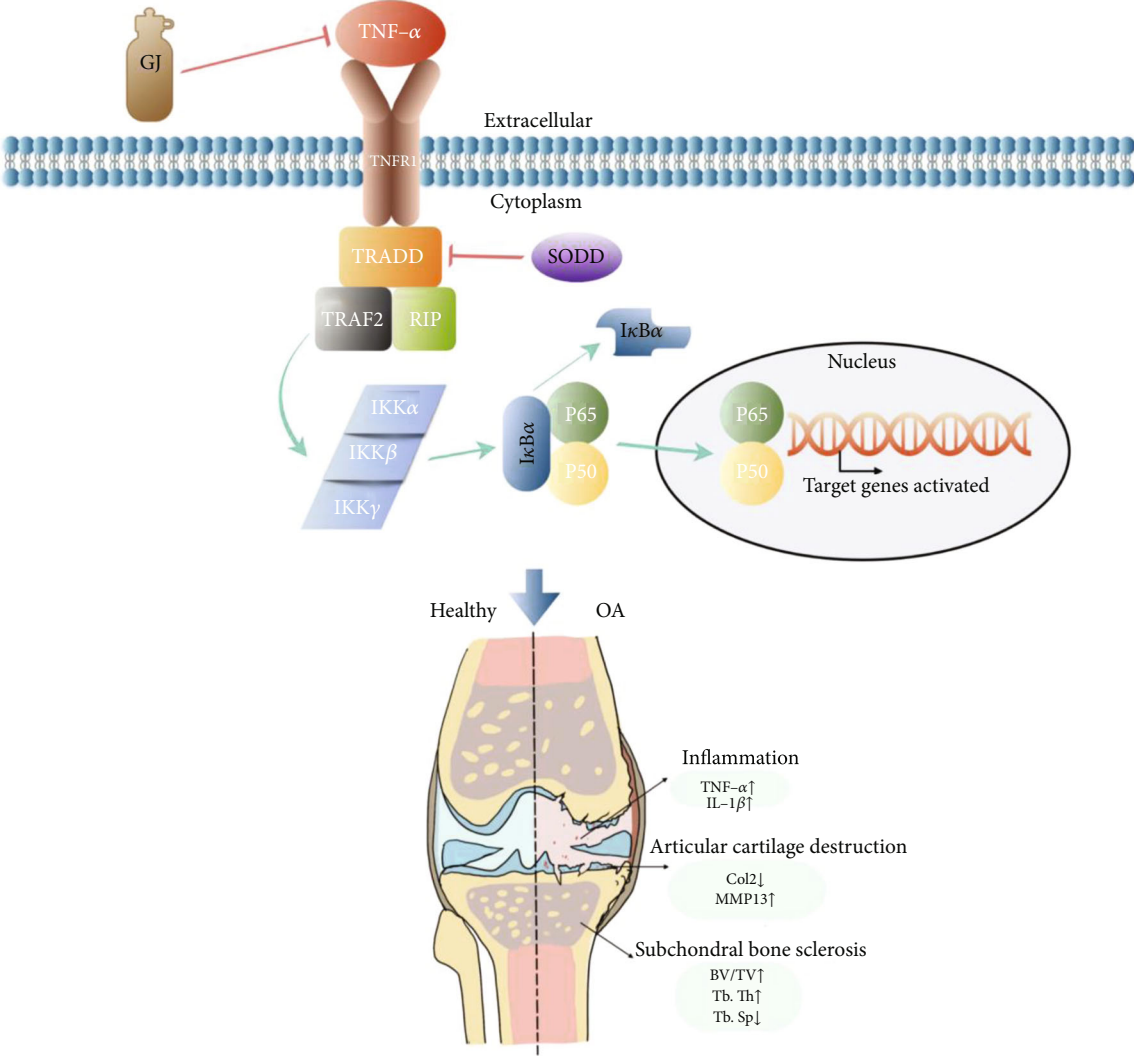
A proposed underlying mechanism of GJ in OA.

We then explored the protective effect of GJ on chondrocytes against inflammation caused by TNF-*α in vitro*. The CCK-8 results manifested that treatment with GJ serum or BAY 11-7082 for 12 and 24 h did not affect primary chondrocyte viability (Figures 7(a)–7(d)). In the RT-PCR assay, GJ serum enhanced the expression of aggrecan and col2 in chondrocytes treated with TNF-*α* (Figures 7(g) and 7(h)). Moreover, GJ serum significantly decreased the mRNA expression of adamts5 and MMP13, which agreed with the result of IHC (Figures 7(e) and 7(f)). And the expression levels of Col10a1, an indicator of chondrocyte hypertrophy, were downregulated in chondrocytes treated with GJ serum or BAY 11-7082 (Figure 7(i)). Moreover, AKT1 was a crucial upstream regulator of NF-*κ*B. Consistent with the molecular docking results, GJ serum dramatically reduced the gene expression level of AKT1 and TNF-*α* (Figures 7(j) and 7(k)), indicating that GJ, like an inhibitor of NF-*κ*B signaling, protected chondrocytes against catabolism and inflammation (Figure 8).

## 4. Discussion

In the present study, we have investigated that whether GJ has a protective effect on OA and explored its underlying mechanism. To clarify the effect of GJ on OA, we first performed network pharmacology to screen a total of 175 bioactive compounds according to OB and DL and obtained 134 overlapping targets after intersecting GJ and OA targets. Many of these targets are relevant to inflammation such as IL6, IL1*β*, CXCL8, and CCL2. On top of that, KEGG analysis also indicates that TNF-*α* and NF-*κ*B signaling is related to the function of GJ. Therefore, we hypothesized that anti-inflammation is an important effect of GJ in the treatment of OA and provided experimental evidence both in DMM mice and in TNF-*α*-reduced chondrocytes. Collectively, this study demonstrates that GJ could attenuate OA through a mechanism that involves TNF-*α*/NF-*κ*B signaling.

Identification of bioactive compounds of GJ is useful for exploring the mechanisms underlying the therapeutic effects of the treatment with TCM. In this study, we found that the top key bioactive compounds included quercetin and kaempferol. A previous study reported that quercetin could alleviate rat osteoarthritis by inhibiting inflammation and apoptosis of chondrocytes [33]. Similarly, kaempferol could also inhibit inflammation and extracellular matrix degradation in OA [34]. Moreover, the present study confirmed that both the top core compounds of the formula and each herb had high binding energy with IL6 and AKT1 through molecular docking. Therefore, all these findings indicated that multiple components in GJ played a critical role in the development of OA mediated by inflammation.

To investigate the curative effect of GJ on OA, we conducted a series of experiments. For instance, the ABH/OG and HE staining results showed that GJ could partially ameliorate cartilage wear and tear, which is one of the mainly accompanied signs during the OA progression [8, 35]. MMP13, a matrix-degrading enzyme regulated by inflammatory cytokines, is involved in the onset and development of OA [36]. Notably, GJ treatment could significantly change the expressions of col2, MMP13, IL-1*β*, and TNF-*α*, suggesting its contribution to inflammation and extracellular matrix degradation. Simultaneously, our results showed the therapeutic effects of GJ on the aberrant subchondral bone structure and gait post DMM surgery, most of which was in accord with previous reports [37, 38].

Indeed, KEGG pathway enrichment analysis of the 134 genes has also highlighted the TNF pathway, implying TNF-*α*/NF*κ*B might be involved in the anti-inflammation effect of GJ. Like other proinflammatory cytokines, TNF-*α* is one of the critical mediators in the degeneration of articular cartilage [39]. Meanwhile, TNF-*α*, by and large, contributes to the progressions of many inflammatory diseases like rheumatoid arthritis and OA [40]. Previous researchers reported that TNF-*α* could bind to tumor necrosis factor receptor 1(TNFR1) which interacts with TNF receptor-associated death domain protein (TRADD) through the silencer of death domain (SODD). TRADD assembles the TNF receptor-associated factor (TRAF) 2 and the death domain-containing serine-threonine kinase receptor-interacting protein (RIP), leading to the activation of inhibitor of NF-*κ*B kinases (IKKs) [41]. Increased activity of IKKs thus induces NF-*κ*B signaling by, in most cases, translocating the p65 (RelA) DNA-binding factor to the nucleus. P65, a key member of NF-*κ*B, is a family of transcription factors that regulate many signaling events, including cell proliferation and apoptosis, as well as inflammation and immune responses [42]. Therefore, inhibiting the activation of p65 would have a beneficial effect on chondrocyte homeostasis. Notably, NF-*κ*B, activated by these inflammatory cytokines or ECM degradation products, would in turn stimulate inflammatory mediators such as IL-1 *β*, TNF-*α*, and IL-6 through positive feedback loops [43]. In our study, GJ suppressed TNF signaling-related gene expressions like TNFR1, TRADD, and TRAF2 in TNF-*α*-induced inflammatory chondrocytes. GJ also inhibited the NF-*κ*B activity of DMM-induced mice by interrupting this interaction between TNF-*α* and NF-*κ*B, which was consistent with our network pharmacological results. Additionally, the gene expressions of adamts5, MMP13, aggrecan, col2, col10a1, AKT1, and TNF-*α* showed parallel trends between chondrocytes treated with GJ serum or NF-*κ*B inhibitor, indicating that GJ could partially inhibit TNF-*α*/NF-*κ*B signaling.

Nevertheless, our study still has several limitations. First, on account of incomplete public database information on herbal medicine, some compounds in GJ have been omitted which may lead to deviation in our study, so the chemical compounds of GJ will be analyzed by HPLC-MS/MS in future research. Moreover, we did not investigate the different dosages of GJ during treatment since the effect of drugs may have a dose-dependent manner. We would explore this issue further in future studies. Finally, our results did not distinguish the detailed mechanism of GJ for the activation of the TNF-*α*/NF-*κ*B signaling; chances are that GJ may alleviate OA through other signaling pathways. For instance, TNF signal transduction pathways could also activate other pathways including MAPK [44], apoptosis [45], and PI3K-Akt signaling in the process of OA [46].

## 5. Conclusion

Collectively, this study for the first time has investigated the underlying mechanism of GJ in ameliorating OA by combining network pharmacology with experimental validation. The network pharmacological analysis predicted that GJ produced its curative effects against OA via regulating multicomponent, multitarget, and multipathway. The in vivo and in vitro experiments verified that the mechanism of GJ treating OA was to inhibit the inflammation and catabolism of articular chondrocytes and subchondral bone partially *via* regulating TNF-*α*/NF-*κ*B signaling. Thus, our findings indicate that GJ might be an alternative therapeutic treatment for OA.

## Figures and Tables

**Table 1:** The compositions of GJ oral liquid.

Chinese name	Latin name	English name	Family	Parts used	Proportion
Huang Qi (HQ)	Astragalus mongholicus Bunge	Astragali Radix	Leguminosae	Root	20.69%
Dan Shen (DS)	Salvia miltiorrhiza Bunge	Salvia miltiorrhiza	Lamiaceae	Root	20.69%
Du Zhong (DZ)	Eucommia ulmoides Oliv.	Eucommia ulmoides	Eucommiaceae	Bark	10.34%
Dang Gui (DG)	Angelica sinensis (Oliv.) Diels	Angelicae Sinensis Radix	Apiaceae	Root	20.69%
Tu Si Zi (TSZ)	Cuscuta chinensis Lam.	Cuscuta seed	Convolvulaceae	Seed	20.69%
Yan Hu Suo (YHS)	Corydalis yanhusuo (Y.H.Chou and Chun C.Hsu) W.T.Wang ex Z.Y.Su and C.Y.Wu	Corydalis yanhusuo	Papaveraceae	Tuber	6.90%

**Table 2:** Primer sequences for quantitative RT-PCR.

Gene	Forward primer (5′-3′)	Reverse primer (5′-3′)
*β*-Actin	GGAGATTACTGCCCTGGCTCCTA	GACTCATCGTACTCCTGCTTGCTG
TNFR1	TGACAGGAAGGCTCAGATGTGC	ATGCTTGCCTCACAGTCCGCAC
TRADD	GTTGGCTGACTGATGAAGAGCG	CACACGTCAGTTTGCAGAGCTC
TRAF2	ACCTGTGATGGCTGTGGCAAGA	TCTGAACAGCCAACGGTGTGGA
Adamts5	CTGCCTTCAAGGCAAATGTGTGG	CAATGGCGGTAGGCAAACTGCA
MMP13	TTTGAGAACACGGGGAAGA	ACTTTGTTGCCAATTCCAGG
Aggrecan	CGCCACTTTCATGACCGAGA	TCATTCAGACCGATCCACTGGTAG
Col2	GCTGGTGAAGAAGGCAAACGAG	CCATCTTGACCTGGGAATCCAC
Col10a1	ACCCCAAGGACCTAAAGGAA	CCCCAGGATACCCTGTTTTT
AKT1	GGACTACTTGCACTCCGAGAAG	CATAGTGGCACCGTCCTTGATC
TNF*α*	GCCCACGTCGTAGCAAACCAC	TCGGGGCAGCCTTGTCCCTT

## Data Availability

The data used to support the findings of this study are available from the corresponding author upon request.

## Supplementary Materials

Supplementary Materials Table S1: ingredients of each herb contained in GJ oral liquid (OB ≥ 30%, DL ≥ 0.18). Table S2: known therapeutic targets correspond to the active ingredients. Table S3: the target protein corresponds to the gene name from UniProt. Table S4: known therapeutic targets for KOA. Table S5: the overlapping gene symbols between disease (osteoarthritis) and drug (GJ oral liquid). Supplementary Table S6: details of the active ingredients and the gene symbols. Supplementary Table S7: the top ten potentially effective compounds and the docking compounds in the prescription. Table S8: details of the PPI network. Table S9: key targets in the network. Table S10: details of GO enrichment analyses. Table S11: results of molecular docking. Table S12: details of KEGG pathway enrichment analyses.
