# Correction to “Elucidation of the Underlying Mechanism of Gujian Oral Liquid Acting on Osteoarthritis Through Network Pharmacology, Molecular Docking, and Experiment”

**DOI:** 10.1155/bmri/9809102

**Published:** 2025-10-16

C. Wu, Q. Ge, Z. Shi, et al., “Elucidation of the Underlying Mechanism of Gujian Oral Liquid Acting on Osteoarthritis Through Network Pharmacology, Molecular Docking, and Experiment,” *BioMed Research International* 2022 (2022): 9230784, https://doi.org/10.1155/2022/9230784.

In the article, there are errors in Figure 5a, introduced during the production process. Specifically, the Col2/GJ panel was erroneously duplicated in the MMP13/GJ panel. The correct Figure 5 is shown as follows:

Figure 5Effects of GJ on expressions of Col2, MMP13, and IL‐1*β* of cartilage in DMM‐induced mice. (a) IHC staining of Col2 and MMP13 (400×). Arrows indicated positive expressions. Scale bar = 50 *μ*m. (b) Immunofluorescence staining of IL‐1*β* in mouse tibial cartilage after DMM surgery (400×). Scale bars = 50 *μ*m. (c–e) Quantification of positive expressions of Col2, MMP13, and IL‐1*β*. All data were shown as means ± SD (*n* ≥ 6).  ^∗∗^ *p* < 0.01,  ^∗∗∗^ *p* < 0.001, and  ^∗∗∗∗^ *p* < 0.0001 by one‐way ANOVA.

**Figure:**
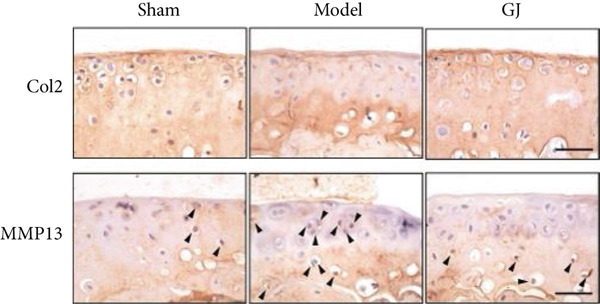
(a)

**Figure:**
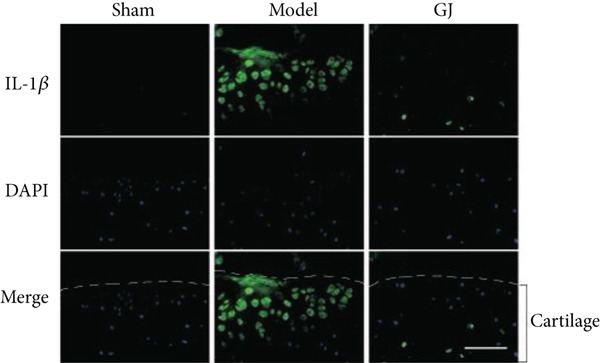
(b)

**Figure:**
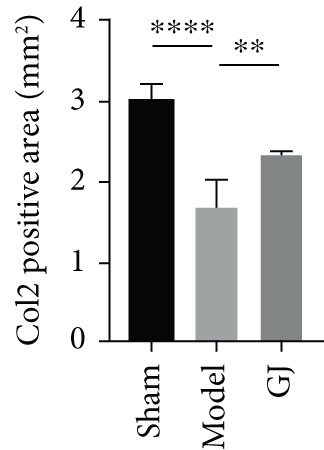
(c)

**Figure:**
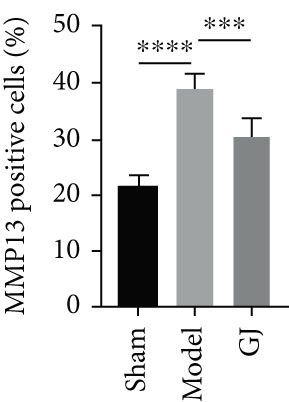
(d)

**Figure:**
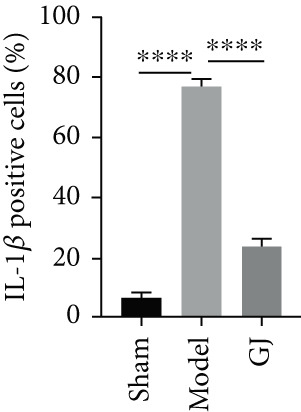
(e)

We apologize for this error.

