# Supplementary material for: Elucidation of the Underlying Mechanism of Gujian Oral Liquid Acting on Osteoarthritis through Network Pharmacology, Molecular Docking, and Experiment
Source: Biomed Res Int. 2022 Jul 28;2022:9230784. doi: 10.1155/2022/9230784 (PMC9352474; doi:10.1155/2022/9230784)
Supplement: Supplementary Materials — Table S1: ingredients of each herb contained in GJ oral liquid (OB ≥ 30%, DL ≥ 0.18). Table S2: known therapeutic targets correspond to the active ingredients. Table S3: the target protein corresponds to the gene name from UniProt. Table S4: known therapeutic targets for KOA. Table S5: the overlapping gene symbols between disease (osteoarthritis) and drug (GJ oral liquid). Supplementary Table S6: details of the active ingredients and the gene symbols. Supplementary Table S7: the top ten potentially effective compounds and the docking compounds in the prescription. Table S8: details of the PPI network. Table S9: key targets in the network. Table S10: details of GO enrichment analyses. Table S11: results of molecular docking. Table S12: details of KEGG pathway enrichment analyses. [file 9230784.f1.zip › Document S1 UPLC.pdf]

Academy of Chinese Medical Science, Zhejiang Chinese Medical University.

Ultra-performance Liquid Chromatography (UPLC) analysis

Sample name	Gujian oral liquid (GJ)	No.	/
Sample identification	/	The test items	UPLC of Chinese herbal compound
Sample status	liquid		
Environmental conditions	temperature 25 ℃ ;	Humidity 54.3 % RH	
The basis for the test	2020 Edition of the *Chinese Pharmacopoeia*		

Instruments and equipment	Name	Model	Numbering
	Liquid chromatograph	Waters ACQUITY UPLC H-Class	2017299406
	electronic scales	XS105DU	2016608106
		ME203E/02	2016618906
	UP Water Purification System	MILLIPORE Direct-8	2016609906
	Rotary evaporator	IKA（2L）	2016610206
	electric jacket	Hangzhou Mingyuan PTHW	/

Reagent	Name	Batch No	Manufacturer	Level
	methanol	20055100	TEDIA	*HPLC*
	acetonitrile	4027269299378	Merck	*HPLC*
	Phosphoric acid	10015418	HUSHI	*AR*

Reference Substance	Name	Batch No	Manufacturer	Specification	Content
	/	/	/	/	/

Reagent preparation		
	the flow rate	acetonitrile, water, phosphoric acid
	Sample solvent	methanol, water

	Reference solvent	/	
sample processing	0 mL, 1. 00 mL, 2. 00 mL and 4. 00 mL Gujian oral liquid were weighed accurately in four round-bottom flasks, and 20 mL 70 % methanol solution was added respectively. The weight was weighed and then cold-immersed for 1 h. After heating and refluxing for 30 min, static cooling was carried out, and then the weight was weighed again. The lost weight was filled with extraction solvent. After shaking and filtering, the filtrate was obtained.		
Instrument conditions	Workstation	Empower 3	
	Column number, model	186002350 / ACQUITY UPLC ®BEH C18 1.7μm 2.1×50mm Column	
	Chromatographic conditions	mobile phase	acetonitrile（A）-0.1% phosphoric acid in water (B)（pH=5.6
		flow rate	0.2 ml/min
		column temperature	30 ℃
		detection wavelength	280 nm
		injection volume (dilution)	2.00 ul
		injection volume(reference Substance)	/ ul
		injection volume (test solution)	/ ul
		running time	16 min
		theoretical plate number	≥2000
Elution method	gradient elution		

	Gradient elution program
	time /min | mobile phase A/% | mobile phase B/% 0
	0 | 5 | 95
	1 | 10 | 90
	3 | 20 | 80
	5 | 28 | 72
	8 | 30 | 70
	10 | 40 | 60
	12 | 80 | 20
	15 | 80 | 20
	15.1 | 10 | 90
	16 | 10 | 90
formula	/
The basis for the test	2020 Edition of the Chinese Pharmacopoeia
Test result	The test results of this product reach the standards
notes	The spectral and result data are shown in the the attached chart for 5 pages.<br>Batch file name: Gujian oral liquid 20210526 。

operator		reviewer	
operating date		reviewing date	

sample information

sample name:	BK-70%methanolic extract	collector:	System
sample type:	unknown	sample group name :	GJ20200526
bottle number:	1:A,3	collection Method Group	GJ6
injection time:	1	processing method:	GJ20210527_2
injection value:	2.00 ul	channel name:	PDA Ch1 280 nm@4.8 nm
running time:	16.0 Minutes	processing channel	PDA Ch1 280 nm@4.8 nm
		comments:	

Acquisition time: 2021/5/26 14:30:19 HKT
Processing time: 2021/5/27 14:09:29 HKT

Chromatogram of automatic scale

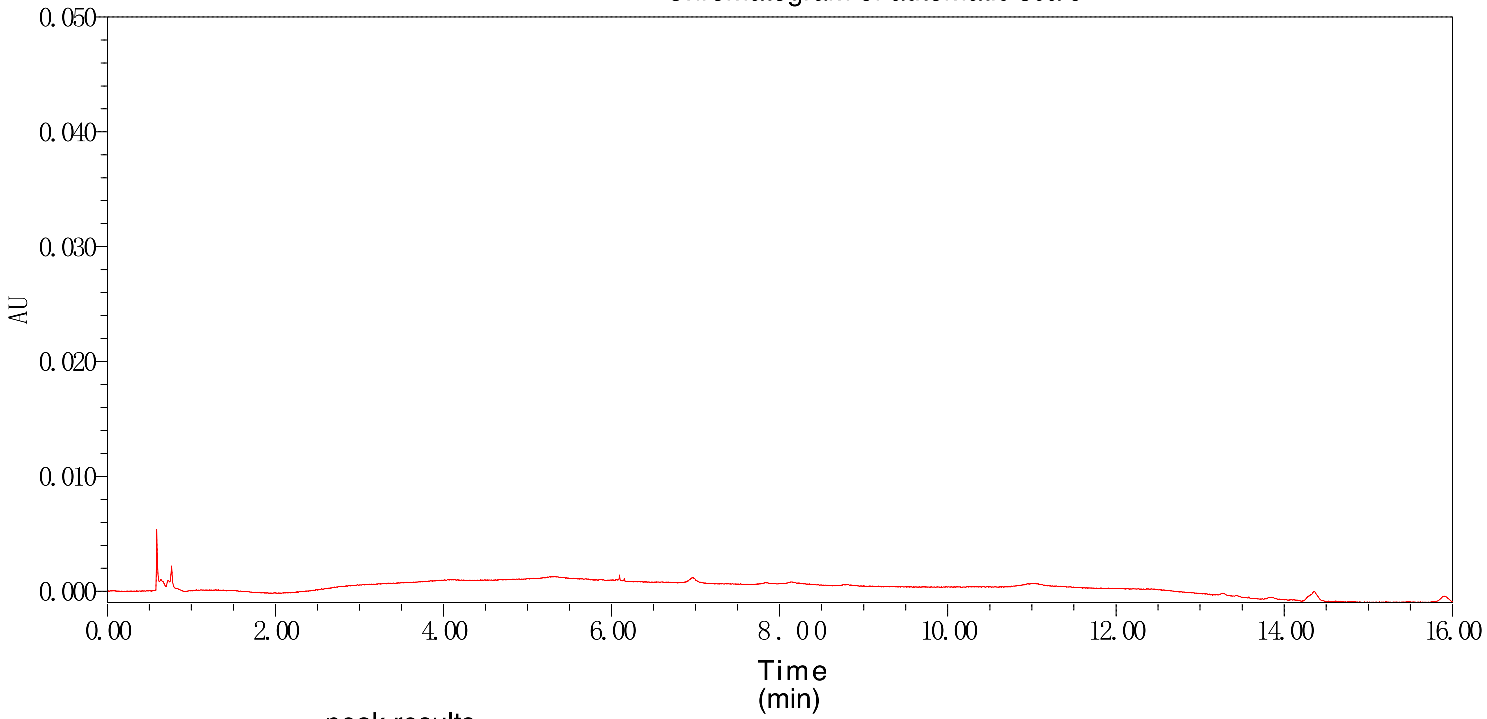

peak results

	name	retention time (min)	area (μV*s)	height (μV)	content	unit
1						

Printing date :
2021/5/27
15:02:32 Asia/Hong_Kong

sample information

sample name:	5 fold-70% methanolic extract	collector:	System
sample type:	unkown	sample group name	GJ20200526
bottle number:	1:A, 6	collection Method Group:	GJ6
injection time:	1	processing method:	GJ20210527_2
injection value:	2.00 ul	channel name:	PDA Ch1 280 nm@4. 8 nm
running time:	16.0 Minutes	processing channel	PDA Ch1 280 nm@4. 8 nm
		comments:	
Acquisition time:	2021/5/26 16:03:17 HKT		
Processing time:	2021/5/27 14:17:06 HKT		

Chromatogram of automatic scale

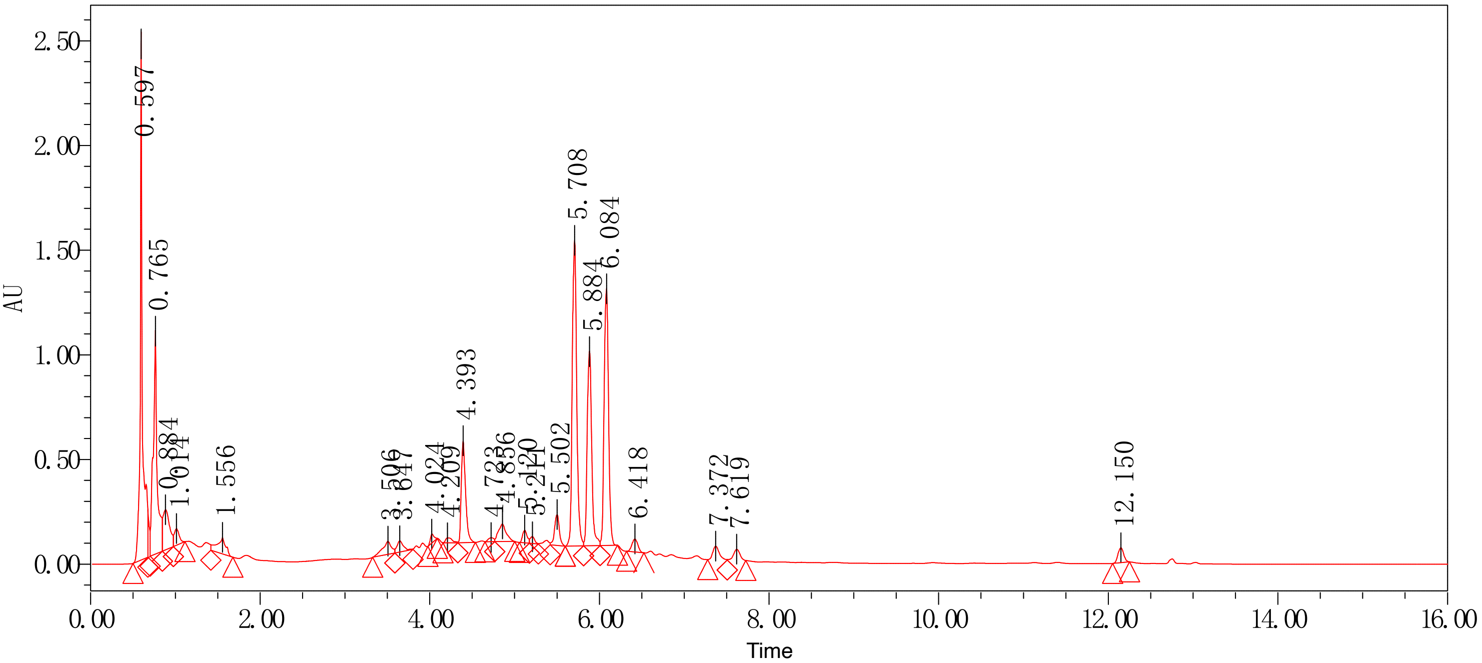

peak results

	name	retention time (min)	area (µV*s)	height (µV)	content	unit
1		0.597	4480196	2528646		
2		0.765	3361417	1068712		
3		0.884	1047141	191219		
4		1.014	326907	79087		
5		1.556	543886	79055		
6		3.506	450915	62650		
7		3.647	287001	54101		
8		4.024	127397	42783		
9		4.209	129968	23973		
10		4.393	1694448	487445		
11		4.723	86077	19293		
12		4.856	540309	83864		
13		5.120	224034	60361		

	name	retention time (min)	area (µV*s)	height (µV)	content	unit
14		5.211	118515	32205		
15		5.502	570992	147663		
16		5.708	5327667	1459609		
17		5.884	3426088	928693		
18		6.084	4431582	1227575		
19		6.418	301143	62123		
20		7.372	339590	65004		
21		7.619	271649	52580		
22		12.150	337367	70973		

Printing date:
2021/5/27
14:30:07 Asia/Hong_Kong

sample information

sample name	10 fold-70%methanolic extract	collector:	System
sample type:	unknown	sample group name :	GJ20200526
bottle number:	1:A,5	collection Method Group:	GJ6
injection time:	1	processing method:	GJ20210527_2
injection value:	2.00 ul	channel name:	PDA Ch1 280 nm@4. 8 nm
running time:	16.0 Minutes	processing channel	PDA Ch1 280 nm@4. 8 nm
		comments:	

Acquisition time: 2021/5/26 15:26:04 HKT
Processing time: 2021/5/27 14:18:01 HKT

Chromatogram of automatic scale

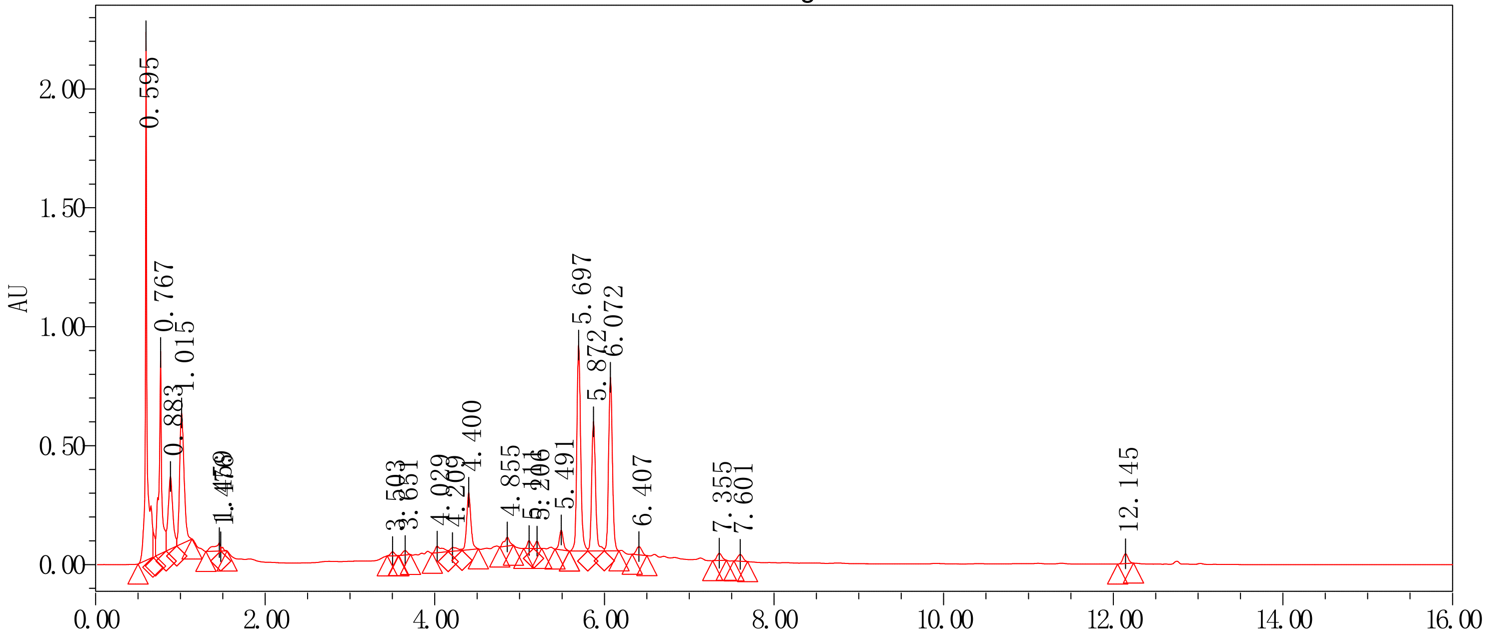

peak results

	name	retention time (min)	area (µV*s)	height (µV)	content	unit
1		0.595	3099230	2225568		
2		0.767	1853635	850716		
3		0.883	1159970	305084		
4		1.015	2011648	550984		
5		1.459	188589	35785		
6		1.476	36451	18186		
7		3.503	57329	17228		
8		3.651	73576	18967		
9		4.029	159555	27773		
10		4.209	96124	15877		
11		4.400	822721	241643		
12		4.855	163996	37528		
13		5.111	103721	32865		

	name	retention time (min)	area (µV*s)	height (µV)	content	unit
14		5.206	88467	30860		
15		5.491	270462	84086		
16		5.697	2733777	865562		
17		5.872	1735920	543547		
18		6.072	2263628	729550		
19		6.407	156368	34965		
20		7.355	125481	30284		
21		7.601	121190	27969		
22		12.145	174357	41124		

Printing date:
2021/5/27
14:29:37 Asia/Hong_Kong

Empower™ 3 SOFTWARE

sample information

sample name	20 fold-70%methanolic extract	collector:	System
sample type:	unknown	sample group name :	GJ20200526
bottle number	1:A,4	collection Method Group:	GJ6
injection time	1	processing method:	GJ20210527_2
injection value	2.00 ul	channel name:	PDA Ch1 280 nm@4. 8 nm
running time	16.0 Minutes	processing channel	PDA Ch1 280 nm@4. 8 nm
		comments:	

Acquisition time: 2021/5/26 15:07:28 HKT
Processing time: 2021/5/27 14:08:43 HKT

Chromatogram of automatic scale

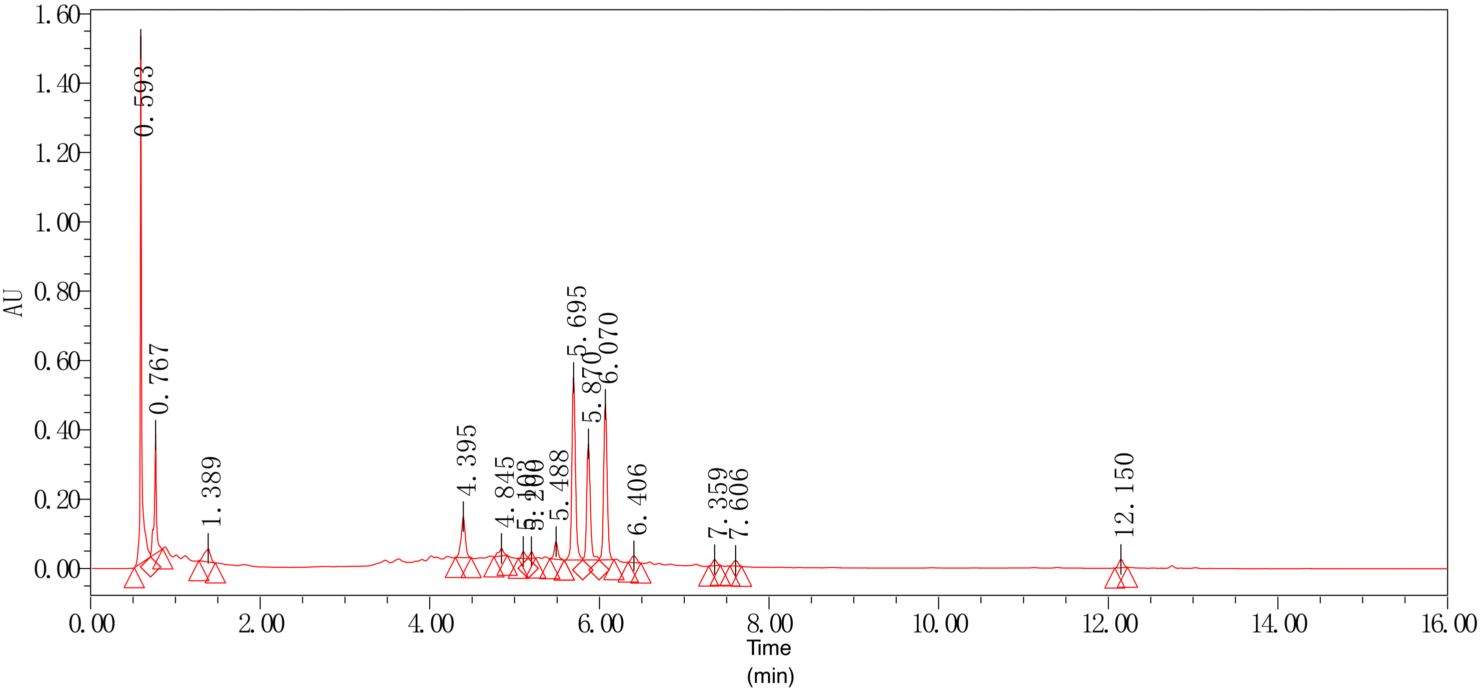

peak results

	name	retention time (min)	area (µV*s)	height (µV)	content	unit
1		0.593	1753042	1521436		
2		0.767	580314	342489		
3		1.389	192811	38979		
4		4.395	402046	118085		
5		4.845	90038	21740		
6		5.103	60310	19619		
7		5.200	51078	19107		
8		5.488	155281	51025		
9		5.695	1555106	526789		
10		5.870	995703	334906		
11		6.070	1291402	449460		
12		6.406	83040	19072		
13		7.359	63332	17245		

	name	retention time (min)	area (µV*s)	height (µV)	content	unit
14		7.606	65522	16370		
15		12.150	90247	23844		

Printing date:
2021/5/27
14:27:57 Asia/Hong_Kong
