# Supplementary material for: Elucidation of the Underlying Mechanism of Gujian Oral Liquid Acting on Osteoarthritis through Network Pharmacology, Molecular Docking, and Experiment
Source: Biomed Res Int. 2022 Jul 28;2022:9230784. doi: 10.1155/2022/9230784 (PMC9352474; doi:10.1155/2022/9230784)
Supplement: Supplementary Materials — Table S1: ingredients of each herb contained in GJ oral liquid (OB ≥ 30%, DL ≥ 0.18). Table S2: known therapeutic targets correspond to the active ingredients. Table S3: the target protein corresponds to the gene name from UniProt. Table S4: known therapeutic targets for KOA. Table S5: the overlapping gene symbols between disease (osteoarthritis) and drug (GJ oral liquid). Supplementary Table S6: details of the active ingredients and the gene symbols. Supplementary Table S7: the top ten potentially effective compounds and the docking compounds in the prescription. Table S8: details of the PPI network. Table S9: key targets in the network. Table S10: details of GO enrichment analyses. Table S11: results of molecular docking. Table S12: details of KEGG pathway enrichment analyses. [file 9230784.f1.zip › Supplementarymaterial_ (1).pdf]

Table S1 Ingredients of each herb contained in GJ oral liquid(OB≥30%,DL≥0.18)

Herb	Mol ID	Molecule Name	MW	AlogP	Hdon	Hacc	OB (%)	Caco-2	BBB	DL	FASA-	HL
Astragali	MOL000211	Mairin	456.78	6.52	2	3	55.38	0.73	0.22	0.78	0.26	8.87
Astragali	MOL000239	Jaranol	314.31	2.09	2	6	50.83	0.61	-0.22	0.29	0.29	15.5
Astragali	MOL000296	hederagenin	414.79	8.08	1	1	36.91	1.32	0.96	0.75	0	5.35
Astragali	MOL000033	(3S,8S,9S,10R,13F	428.82	8.54	1	1	36.23	1.45	1.09	0.78	0	5.22
Astragali	MOL000354	isorhamnetin	316.28	1.76	4	7	49.6	0.31	-0.54	0.31	0.32	14.34
Astragali	MOL000371	3,9-di-O-methylnis:	314.36	2.89	0	5	53.74	1.18	0.63	0.48	0	9
Astragali	MOL000374	5'-hydroxyiso-muro	642.67	-0.95	9	16	41.72	-2.47	-3.62	0.69	0	2.52
Astragali	MOL000378	7-O-methylisomucr	316.38	3.38	1	5	74.69	1.08	0.84	0.3	0	2.98
Astragali	MOL000379	9,10-dimethoxypter	462.49	0.74	4	10	36.74	-0.63	-1.5	0.92	0	13.06
Astragali	MOL000380	(6aR,11aR)-9,10-di	300.33	2.64	1	5	64.26	0.93	0.55	0.42	0	8.49
Astragali	MOL000387	Bifendate	418.38	2.56	0	10	31.1	0.15	-0.06	0.67	0	17.96
Astragali	MOL000392	formononetin	268.28	2.58	1	4	69.67	0.78	0.02	0.21	0	17.04
Astragali	MOL000398	isoflavanone	316.33	2.42	2	6	109.99	0.53	0.17	0.3	0	15.51
Astragali	MOL000417	Calycosin	284.28	2.32	2	5	47.75	0.52	-0.43	0.24	0	17.1
Astragali	MOL000422	kaempferol	286.25	1.77	4	6	41.88	0.26	-0.55	0.24	0	14.74
Astragali	MOL000433	FA	441.45	0.01	7	13	68.96	-1.5	-2.59	0.71	0	24.81
Astragali	MOL000438	(3R)-3-(2-hydroxy-	302.35	3.13	2	5	67.67	0.96	0.34	0.26	0	2.9
Astragali	MOL000439	isomucronulatol-7,2	626.67	-0.68	8	15	49.28	-2.22	-3.36	0.62	0	0.93
Astragali	MOL000442	1,7-Dihydroxy-3,9-	314.31	3.11	2	6	39.05	0.89	-0.04	0.48	0	7.95
Astragali	MOL000098	quercetin	302.25	1.5	5	7	46.43	0.05	-0.77	0.28	0.38	14.4
Salviae liguliobae	MOL001601	1,2,5,6-tetrahydrota	280.34	2.98	0	3	38.75	0.96	0.39	0.36	0.33	18.05
Salviae liguliobae	MOL001659	Poriferasterol	412.77	7.64	1	1	43.83	1.44	1.03	0.76	0.22	5.34
Salviae liguliobae	MOL001771	poriferast-5-en-3be	414.79	8.08	1	1	36.91	1.45	1.14	0.75	0	5.07
Salviae liguliobae	MOL001942	isoimperatorin	270.3	3.65	0	4	45.46	0.97	0.66	0.23	0.27	-1.44
Salviae liguliobae	MOL002222	sugiol	300.48	4.99	1	2	36.11	1.14	0.7	0.28	0.27	14.62
Salviae liguliobae	MOL002651	Dehydrotanshinone	292.35	4.22	0	3	43.76	1.02	0.52	0.4	0.33	23.71
Salviae liguliobae	MOL002776	Baicalin	446.39	0.64	6	11	40.12	-0.85	-1.74	0.75	0.36	17.36
Salviae liguliobae	MOL000569	digallate	322.24	1.53	6	9	61.85	-0.76	-1.52	0.26	0.43	5.29
Salviae liguliobae	MOL000006	luteolin	286.25	2.07	4	6	36.16	0.19	-0.84	0.25	0.39	15.94

Salviae liguliobae	MOL006824	α-amyrin	426.8	7.35	1	1	39.51	1.37	1.2	0.76	0.23	3.06
Salviae liguliobae	MOL007036	5,6-dihydroxy-7-isc	298.41	4.38	2	3	33.77	1.19	0.8	0.29	0.29	14.91
Salviae liguliobae	MOL007041	2-isopropyl-8-meth	264.34	4.16	0	2	40.86	1.23	0.81	0.23	0.43	14.89
Salviae liguliobae	MOL007045	3α-hydroxytanshinc	310.37	3.56	1	4	44.93	0.53	0.22	0.44	0.3	23.78
Salviae liguliobae	MOL007048	(E)-3-[2-(3,4-dihyd	312.29	3.21	4	6	48.24	0.18	-0.89	0.31	0.4	8.87
Salviae liguliobae	MOL007049	4-methylenemiltiror	266.36	4.33	0	2	34.35	1.25	0.87	0.23	0.38	14.6
Salviae liguliobae	MOL007050	2-(4-hydroxy-3-me	356.4	3.58	2	6	62.78	0.35	-0.73	0.4	0.24	7.89
Salviae liguliobae	MOL007051	6-o-syringyl-8-o-ac	628.64	-1.13	5	16	46.69	-1.73	-2.08	0.71	0.22	9.94
Salviae liguliobae	MOL007058	formyltanshinone	290.28	3.36	0	4	73.44	0.54	-0.28	0.42	0.41	24.12
Salviae liguliobae	MOL007059	3-beta-Hydroxymet	294.32	3.16	1	4	32.16	0.38	-0.48	0.41	0.36	22.51
Salviae liguliobae	MOL007061	Methylenetanshinqu	278.32	4.26	0	3	37.07	1.03	0.46	0.36	0.36	24.33
Salviae liguliobae	MOL007063	przewalskin a	398.49	2.25	1	6	37.11	-0.26	-0.69	0.65	0.38	1.63
Salviae liguliobae	MOL007064	przewalskin b	330.46	3.18	1	4	110.32	0.34	0.22	0.44	0.32	2.17
Salviae liguliobae	MOL007068	Przewaquinone B	292.3	2.99	1	4	62.24	0.39	-0.45	0.41	0.38	24.94
Salviae liguliobae	MOL007069	przewaquinone c	296.34	3.31	1	4	55.74	0.42	-0.3	0.4	0.32	23.7
Salviae liguliobae	MOL007070	(6S,7R)-6,7-dihydr	312.34	2.34	2	5	41.31	-0.06	-0.68	0.45	0.32	22.54
Salviae liguliobae	MOL007071	przewaquinone f	312.34	2.07	2	5	40.31	-0.09	-0.9	0.46	0.29	22.45
Salviae liguliobae	MOL007077	sclareol	308.56	4.27	2	2	43.67	0.84	0.51	0.21	0.27	4.71
Salviae liguliobae	MOL007079	tanshinaldehyde	308.35	3.83	0	4	52.47	0.57	-0.07	0.45	0.32	23.49
Salviae liguliobae	MOL007081	Danshenol B	354.48	2.59	1	4	57.95	0.53	0.11	0.56	0.3	4.28
Salviae liguliobae	MOL007082	Danshenol A	336.41	2.01	1	4	56.97	0.33	-0.01	0.52	0.34	5.15
Salviae liguliobae	MOL007085	Salvilenone	292.4	4.26	0	2	30.38	1.46	1.07	0.38	0.35	20.81
Salviae liguliobae	MOL007088	cryptotanshinone	296.39	3.44	0	3	52.34	0.95	0.51	0.4	0.29	17.3
Salviae liguliobae	MOL007093	dan-shexinkum d	336.41	2.83	1	4	38.88	0.67	-0.15	0.55	0.35	30
Salviae liguliobae	MOL007094	danshenspiroketalla	282.36	3.24	0	3	50.43	0.88	0.51	0.31	0.34	15.19
Salviae liguliobae	MOL007098	deoxyneocryptotans	298.41	4.32	1	3	49.4	0.85	0.24	0.29	0.3	27.17
Salviae liguliobae	MOL007100	dihydrotanshinlacto	266.31	2.77	0	3	38.68	1.26	0.81	0.32	0.38	5.42
Salviae liguliobae	MOL007101	dihydrotanshinone	278.32	2.86	0	3	45.04	0.95	0.43	0.36	0.4	18.32
Salviae liguliobae	MOL007105	epidanshenspiroketa	284.38	2.37	0	3	68.27	0.9	0.61	0.31	0.33	1.77
Salviae liguliobae	MOL007107	C09092	286.5	5.98	1	1	36.07	1.63	1.54	0.25	0.25	-0.16
Salviae liguliobae	MOL007108	isocryptotanshi-nor	296.39	3.59	0	3	54.98	0.93	0.34	0.39	0.3	31.92
Salviae liguliobae	MOL007111	Isotanshinone II	294.37	4.66	0	3	49.92	1.03	0.45	0.4	0.3	24.73

Salviae liguliobae	MOL007115	manool	304.57	5.5	1	1	45.04	1.28	1.16	0.2	0.28	5.81
Salviae liguliobae	MOL007118	microstegiol	298.46	4.75	1	2	39.61	1.05	0.99	0.28	0.33	4.52
Salviae liguliobae	MOL007119	miltionone Ⅰ	312.39	3.33	1	4	49.68	0.35	-0.11	0.32	0.35	41.49
Salviae liguliobae	MOL007120	miltionone Ⅱ	312.39	2.14	1	4	71.03	0.62	0.03	0.44	0.28	2.91
Salviae liguliobae	MOL007121	miltipolone	300.43	2.74	1	3	36.56	0.5	0.17	0.37	0.3	1.7
Salviae liguliobae	MOL007122	Miltirone	282.41	4.73	0	2	38.76	1.23	0.87	0.25	0.32	14.82
Salviae liguliobae	MOL007123	miltirone Ⅱ	272.32	0.77	1	4	44.95	0.04	-0.25	0.24	0.35	2.24
Salviae liguliobae	MOL007124	neocryptotanshinon	270.35	3.61	1	3	39.46	0.76	0.16	0.23	0.32	26.98
Salviae liguliobae	MOL007125	neocryptotanshinon	314.41	3.01	2	4	52.49	0.35	-0.13	0.32	0.28	14.46
Salviae liguliobae	MOL007127	1-methyl-8,9-dihyd	280.29	3.21	0	4	34.72	0.5	-0.27	0.37	0.33	37.89
Salviae liguliobae	MOL007130	prolithospermic aci	314.31	2.77	4	6	64.37	0.1	-0.75	0.31	0.42	8.82
Salviae liguliobae	MOL007132	(2R)-3-(3,4-dihydro	360.34	2.69	5	8	109.38	-0.33	-1.02	0.35	0.41	2.01
Salviae liguliobae	MOL007140	(Z)-3-[2-[(E)-2-(3,4	314.31	2.82	5	6	88.54	-0.09	-0.77	0.26	0.43	4.31
Salviae liguliobae	MOL007141	salvianolic acid g	340.3	2.2	4	7	45.56	-0.14	-0.97	0.61	0.45	2.4
Salviae liguliobae	MOL007142	salvianolic acid j	538.49	3.78	6	12	43.38	-0.82	-2.14	0.72	0.44	5.77
Salviae liguliobae	MOL007143	salvilenone Ⅰ	270.4	2.88	1	2	32.43	1.13	0.77	0.23	0.3	1
Salviae liguliobae	MOL007145	salviolone	268.38	4.05	1	2	31.72	1.04	0.72	0.24	0.36	0.33
Salviae liguliobae	MOL007149	NSC 122421	300.48	4.99	1	2	34.49	1.08	0.63	0.28	0.29	14.56
Salviae liguliobae	MOL007150	(6S)-6-hydroxy-1-r	312.34	2.42	2	5	75.39	0.03	-0.74	0.46	0.29	23.45
Salviae liguliobae	MOL007151	Tanshindiol B	312.34	2.34	2	5	42.67	0.05	-0.63	0.45	0.33	22.25
Salviae liguliobae	MOL007152	Przewaquinone E	312.34	2.34	2	5	42.85	-0.04	-0.65	0.45	0.32	22.44
Salviae liguliobae	MOL007154	tanshinone iia	294.37	4.66	0	3	49.89	1.05	0.7	0.4	0.31	23.56
Salviae liguliobae	MOL007155	(6S)-6-(hydroxyme	310.37	3.57	1	4	65.26	0.44	-0.31	0.45	0.29	23.48
Salviae liguliobae	MOL007156	tanshinone Ⅵ	296.34	2.44	2	4	45.64	0.48	-0.28	0.3	0.38	15.21
Eucommiae cortex	MOL002058	40957-99-1	388.45	2.12	2	7	57.2	0.49	-0.29	0.62	0.2	2.04
Eucommiae cortex	MOL000211	Mairin	456.78	6.52	2	3	55.38	0.73	0.22	0.78	0.26	8.87
Eucommiae cortex	MOL000358	beta-sitosterol	414.79	8.08	1	1	36.91	1.32	0.99	0.75	0.23	5.36
Eucommiae cortex	MOL000422	kaempferol	286.25	1.77	4	6	41.88	0.26	-0.55	0.24	0	14.74
Eucommiae cortex	MOL004367	olivil	376.44	1.68	4	7	62.23	-0.16	-0.75	0.41	0	2.27
Eucommiae cortex	MOL000443	Erythraline	297.38	2.06	0	4	49.18	0.91	0.55	0.55	0.22	11.11
Eucommiae cortex	MOL005922	Acanthoside B	580.64	0.2	5	13	43.35	-0.93	-1.88	0.77	0.17	2.94
Eucommiae cortex	MOL006709	AIDS214634	374.42	1.62	3	7	92.43	0.18	-0.56	0.55	0.24	1.83

Eucommiae cortex	MOL007059	3-beta-Hydroxymet	294.32	3.16	1	4	32.16	0.38	-0.48	0.41	0.36	22.51
Eucommiae cortex	MOL000073	ent-Epicatechin	290.29	1.92	5	6	48.96	0.02	-0.64	0.24	0.34	0.63
Eucommiae cortex	MOL007563	Yangambin	446.54	2.6	0	8	57.53	0.67	0.01	0.81	0.14	3.61
Eucommiae cortex	MOL009007	Eucommin A	550.61	0.21	5	12	30.51	-0.97	-1.71	0.85	0.24	4.5
Eucommiae cortex	MOL009009	(+)-medioresinol	388.45	2.12	2	7	87.19	0.5	-0.29	0.62	0.2	1.39
Eucommiae cortex	MOL009015	(-)-Tabernemontani	354.49	3.45	1	4	58.67	0.66	0.36	0.61	0.27	19.73
Eucommiae cortex	MOL009027	Cyclopamine	411.69	4.26	2	3	55.42	0.77	0	0.82	0.22	14.67
Eucommiae cortex	MOL009029	Dehydrodiconiferyl	358.42	2.16	3	6	51.44	0.04	-1.16	0.4	0.27	7.54
Eucommiae cortex	MOL009030	Dehydrodieugenol	326.42	4.8	2	4	30.1	1.19	0.63	0.24	0.28	2.2
Eucommiae cortex	MOL009031	Cinchonan-9-al, 6'-	324.46	2.73	1	4	68.22	0.63	0.17	0.4	0.25	3.94
Eucommiae cortex	MOL009038	GBGB	550.57	-4	8	15	45.58	-3.29	-5.43	0.83	0.26	10.25
Eucommiae cortex	MOL009042	Helenalin	262.33	1.3	1	4	77.01	0.06	-0.22	0.19	0.42	4.34
Eucommiae cortex	MOL009047	(+)-Eudesmin	386.48	2.64	0	6	33.29	0.93	0.19	0.62	0.18	2.24
Eucommiae cortex	MOL009053	4-[(2S,3R)-5-[(E)-3	358.42	2.16	3	6	50.76	0.03	-0.98	0.39	0.27	7.58
Eucommiae cortex	MOL009055	hirsutin_qt	345.35	2.15	3	7	49.81	0.62	0.02	0.37	0.21	1.4
Eucommiae cortex	MOL009057	liriodendrin_qt	450.48	1.57	4	10	53.14	0.34	-0.3	0.8	0.11	3.78
Eucommiae cortex	MOL000098	quercetin	302.25	1.5	5	7	46.43	0.05	-0.77	0.28	0.38	14.4
Eucommiae cortex	MOL002773	beta-carotene	536.96	12	0	0	37.18	2.25	1.52	0.58	0.33	4.36
Eucommiae cortex	MOL008240	(E)-3-[4-[(1R,2R)-2	374.42	2.19	3	7	56.32	-0.16	-1.11	0.36	0.32	4.18
Eucommiae cortex	MOL011604	Syringetin	346.31	1.74	4	8	36.82	0.05	-0.59	0.37	0.32	14.74
Angelicae Sinensis	MOL000358	beta-sitosterol	414.79	8.08	1	1	36.91	1.32	0.99	0.75	0.23	5.36
Angelicae Sinensis	MOL000449	Stigmasterol	412.77	7.64	1	1	43.83	1.44	1	0.76	0.22	5.57
Semen Cuseutae	MOL001558	sesamin	354.38	2.24	0	6	56.55	0.75	-0.08	0.83	0.31	13.44
Semen Cuseutae	MOL000184	NSC63551	412.77	7.56	0	1	39.25	1.42	1.22	0.76	0.22	5.78
Semen Cuseutae	MOL000354	isorhamnetin	316.28	1.76	4	7	49.6	0.31	-0.54	0.31	0.32	14.34
Semen Cuseutae	MOL000358	beta-sitosterol	414.79	8.08	1	1	36.91	1.32	0.99	0.75	0.23	5.36
Semen Cuseutae	MOL000422	kaempferol	286.25	1.77	4	6	41.88	0.26	-0.55	0.24	0	14.74
Semen Cuseutae	MOL005043	campest-5-en-3beta	400.76	7.63	1	1	37.58	1.32	0.94	0.71	0.23	4.43
Semen Cuseutae	MOL005440	Isofucosterol	412.77	7.83	1	1	43.78	1.36	0.97	0.76	0.24	5.18
Semen Cuseutae	MOL005944	matrine	248.41	1.42	0	3	63.77	1.39	1.52	0.25	0	6.69
Semen Cuseutae	MOL006649	sophranol	264.41	0.67	1	4	55.42	0.6	0.68	0.28	0.19	4.94
Semen Cuseutae	MOL000953	CLR	386.73	7.38	1	1	37.87	1.43	1.13	0.68	0.2	4.52

Semen Cuseutae	MOL000098	quercetin	302.25	1.5	5	7	46.43	0.05	-0.77	0.28	0.38	14.4
Rhizoma Corydalis	MOL001454	berberine	336.39	3.45	0	4	36.86	1.24	0.57	0.78	0.19	6.57
Rhizoma Corydalis	MOL001458	coptisine	320.34	3.25	0	4	30.67	1.21	0.32	0.86	0.26	9.33
Rhizoma Corydalis	MOL001460	Cryptopin	369.45	3.15	0	6	78.74	0.79	0.41	0.72	0.21	21.25
Rhizoma Corydalis	MOL001461	Dihydrochelerythri	349.41	3.91	0	5	32.73	1.13	0.61	0.81	0.26	5.6
Rhizoma Corydalis	MOL001463	Dihydrosanguinari	333.36	3.71	0	5	59.31	1	0.49	0.86	0.31	7.46
Rhizoma Corydalis	MOL001474	sanguinarine	332.35	3.47	0	4	37.81	1.26	0.15	0.86	0.3	7.84
Rhizoma Corydalis	MOL000217	(S)-Scoulerine	327.41	3.1	2	5	32.28	0.89	0.34	0.54	0.2	1.69
Rhizoma Corydalis	MOL002670	Cavidine	353.45	3.72	0	5	35.64	1.08	0.63	0.81	0	5.78
Rhizoma Corydalis	MOL002903	(R)-Canadine	339.42	3.4	0	5	55.37	1.04	0.57	0.77	0.2	6.41
Rhizoma Corydalis	MOL000359	sitosterol	414.79	8.08	1	1	36.91	1.32	0.87	0.75	0.22	5.37
Rhizoma Corydalis	MOL004071	Hyndarin	355.47	3.6	0	5	73.94	1	0.62	0.64	0	2.42
Rhizoma Corydalis	MOL004190	(-)-alpha-N-methyl	354.46	3.49	0	4	45.06	1.15	0.45	0.8	0.19	5.78
Rhizoma Corydalis	MOL004191	Capaurine	371.47	3.33	1	6	62.91	0.86	0.51	0.69	0.16	2.74
Rhizoma Corydalis	MOL004193	Clarkeanidine	327.41	3.1	2	5	86.65	0.86	0.42	0.54	0.21	2.74
Rhizoma Corydalis	MOL004195	CORYDALINE	369.5	3.92	0	5	65.84	1.23	0.9	0.68	0.14	1.77
Rhizoma Corydalis	MOL004196	Corydalmine	340.45	4.68	1	4	52.5	1.21	0.57	0.59	0.23	2.1
Rhizoma Corydalis	MOL004197	Corydine	341.44	3.28	1	5	37.16	1.29	0.79	0.55	0.22	2.25
Rhizoma Corydalis	MOL004198	18797-79-0	367.43	2.73	1	6	46.06	0.72	0.19	0.85	0.26	7.66
Rhizoma Corydalis	MOL004199	Corynoloxine	365.41	3.16	0	6	38.12	0.62	0.25	0.6	0.29	7.97
Rhizoma Corydalis	MOL004200	methyl-[2-(3,4,6,7-	355.47	3.44	1	5	61.15	1.06	0.19	0.44	0.22	2.01
Rhizoma Corydalis	MOL004202	dehydrocavidine	351.43	3.33	0	5	38.99	1.21	0.62	0.81	0.25	6.05
Rhizoma Corydalis	MOL004203	Dehydrocorybulbin	352.44	3.88	1	4	46.97	1.32	0.49	0.63	0.15	1.06
Rhizoma Corydalis	MOL004204	dehydrocorydaline	366.47	4.13	0	4	41.98	1.35	0.48	0.68	0.13	1.67
Rhizoma Corydalis	MOL004205	Dehydrocorydalmi	338.41	3.4	1	4	43.9	1.2	0.31	0.59	0.16	1.94
Rhizoma Corydalis	MOL004208	demethylcorydalma	327.41	3.1	2	5	38.99	0.85	0.46	0.54	0.23	2.03
Rhizoma Corydalis	MOL004209	13-methyldehydroc	352.44	3.88	1	4	35.94	1.15	0.2	0.63	0.16	1.68
Rhizoma Corydalis	MOL004210	(1S,8'R)-6,7-dimet	369.45	2.75	1	6	43.95	0.62	0.22	0.72	0.21	10.89
Rhizoma Corydalis	MOL004763	Izoteolin	327.41	3.03	2	5	39.53	0.84	0.37	0.51	0.23	0.02
Rhizoma Corydalis	MOL004214	isocorybulbine	368.51	5.14	1	4	40.18	1.18	0.69	0.66	0.21	2.26
Rhizoma Corydalis	MOL004215	leonticine	327.46	3.91	1	4	45.79	1.21	0.7	0.26	0.25	5.4
Rhizoma Corydalis	MOL004216	13-methylpalmatrul	352.44	3.88	1	4	40.97	1.09	0.23	0.63	0.17	1.53

Rhizoma Corydalis	MOL004220	N-methyllaurotetan	341.44	3.28	1	5	41.62	0.84	0.32	0.56	0.21	0.14
Rhizoma Corydalis	MOL004221	norglaucing	341.44	3	1	5	30.35	0.95	0.37	0.56	0.15	1.27
Rhizoma Corydalis	MOL004224	pontevedrine	381.41	2.63	0	7	30.28	0.64	-0.22	0.71	0.24	13.26
Rhizoma Corydalis	MOL004225	pseudocoptisine	320.34	3.25	0	4	38.97	1.23	0.12	0.86	0.27	8.56
Rhizoma Corydalis	MOL004226	24240-05-9	353.4	2.95	0	6	53.75	0.8	0.39	0.83	0.29	22.7
Rhizoma Corydalis	MOL004228	saulatine	396.47	3.25	0	6	42.74	0.55	-0.08	0.79	0.21	15.02
Rhizoma Corydalis	MOL004230	stylopine	323.37	3.2	0	5	48.25	0.93	0.52	0.85	0.27	9.57
Rhizoma Corydalis	MOL004231	Tetrahydrocorysam	337.4	3.52	0	5	34.17	1.07	0.48	0.86	0.27	8.96
Rhizoma Corydalis	MOL004232	tetrahydroprotopapa	329.43	3.34	2	5	57.28	0.79	0.32	0.33	0.26	2.6
Rhizoma Corydalis	MOL004233	ST057701	341.44	3.28	1	5	31.87	1.2	0.62	0.56	0.18	-0.34
Rhizoma Corydalis	MOL004234	2,3,9,10-tetrametho	381.46	3.14	0	6	76.77	1.17	0.36	0.73	0.19	1.97
Rhizoma Corydalis	MOL000449	Stigmasterol	412.77	7.64	1	1	43.83	1.44	1	0.76	0.22	5.57
Rhizoma Corydalis	MOL000785	palmatine	352.44	3.65	0	4	64.6	1.33	0.37	0.65	0.13	2.25
Rhizoma Corydalis	MOL000787	Fumarine	353.4	2.95	0	6	59.26	0.56	-0.13	0.83	0.3	23.46
Rhizoma Corydalis	MOL000790	Isocorypalmine	341.44	3.35	1	5	35.77	0.85	0.43	0.59	0.21	2.47
Rhizoma Corydalis	MOL000791	bicuculline	367.38	2.83	0	7	69.67	0.72	0.02	0.88	0.33	15.83
Rhizoma Corydalis	MOL000793	C09367	325.39	3.08	1	5	47.54	1.2	0.7	0.69	0.25	5.17
Rhizoma Corydalis	MOL000098	quercetin	302.25	1.5	5	7	46.43	0.05	-0.77	0.28	0.38	14.4

Table S2 Known therapeutic targets correspond to the active ingredients

Herb	MolId	MolName	Target	Target（delete duplicates）
Astragali	MOL000211	Mairin	Progesterone receptor	Progesterone receptor
Astragali	MOL000239	Jaranol	Nitric oxide synthase, inducible	Nitric oxide synthase, inducible
Astragali	MOL000239	Jaranol	Prostaglandin G/H synthase 1	Prostaglandin G/H synthase 1
Astragali	MOL000239	Jaranol	Androgen receptor	Androgen receptor
Astragali	MOL000239	Jaranol	Sodium channel protein type 5 subunit alpha	Sodium channel protein type 5 subunit alpha
Astragali	MOL000239	Jaranol	Prostaglandin G/H synthase 2	Prostaglandin G/H synthase 2
Astragali	MOL000239	Jaranol	Estrogen receptor beta	Estrogen receptor beta
Astragali	MOL000239	Jaranol	Dipeptidyl peptidase IV	Dipeptidyl peptidase IV
Astragali	MOL000239	Jaranol	Heat shock protein HSP 90	Heat shock protein HSP 90
Astragali	MOL000239	Jaranol	Cell division protein kinase 2	Cell division protein kinase 2
Astragali	MOL000239	Jaranol	Serine/threonine-protein kinase Chk1	Serine/threonine-protein kinase Chk1
Astragali	MOL000239	Jaranol	Trypsin-1	Trypsin-1
Astragali	MOL000239	Jaranol	Nuclear receptor coactivator 2	Nuclear receptor coactivator 2
Astragali	MOL000239	Jaranol	Calmodulin	Calmodulin
Astragali	MOL000296	hederagenin	Progesterone receptor	Muscarinic acetylcholine receptor M3
Astragali	MOL000296	hederagenin	Nuclear receptor coactivator 2	Muscarinic acetylcholine receptor M1
Astragali	MOL000296	hederagenin	Muscarinic acetylcholine receptor M3	Gamma-aminobutyric-acid receptor alpha-2 subunit

Astragali	MOL000296	hederagenin	Muscarinic acetylcholine receptor M1	Gamma-aminobutyric-acid receptor alpha-3 subunit
Astragali	MOL000296	hederagenin	Gamma-aminobutyric-acid receptor alpha-2 subunit	Muscarinic acetylcholine receptor M2
Astragali	MOL000296	hederagenin	Gamma-aminobutyric-acid receptor alpha-3 subunit	Alpha-1B adrenergic receptor
Astragali	MOL000296	hederagenin	Muscarinic acetylcholine receptor M2	Gamma-aminobutyric acid receptor subunit alpha-1
Astragali	MOL000296	hederagenin	Alpha-1B adrenergic receptor	Glutamate receptor 2
Astragali	MOL000296	hederagenin	Gamma-aminobutyric acid receptor subunit alpha-1	Gamma-aminobutyric-acid receptor subunit alpha-6
Astragali	MOL000296	hederagenin	Glutamate receptor 2	Gamma-aminobutyric-acid receptor alpha-5 subunit
Astragali	MOL000296	hederagenin	Gamma-aminobutyric-acid receptor subunit alpha-6	Ig gamma-1 chain C region
Astragali	MOL000296	hederagenin	Gamma-aminobutyric-acid receptor alpha-5 subunit	Alcohol dehydrogenase 1B
Astragali	MOL000296	hederagenin	Ig gamma-1 chain C region	Alcohol dehydrogenase 1C
Astragali	MOL000296	hederagenin	Alcohol dehydrogenase 1B	Lysozyme
Astragali	MOL000296	hederagenin	Alcohol dehydrogenase 1C	Nicotinate-nucleotide--dimethylbenzimidazole phosphoribosyltransferase
Astragali	MOL000296	hederagenin	Lysozyme	Retinoic acid receptor RXR-alpha
Astragali	MOL000296	hederagenin	Nicotinate-nucleotide--dimethylbenzimidazole phosphoribosyltransferase	CGMP-inhibited 3',5'-cyclic phosphodiesterase A
Astragali	MOL000296	hederagenin	Prostaglandin G/H synthase 1	Sodium-dependent noradrenaline transporter

Astragali	MOL000296	hederagenin	Sodium channel protein type 5 subunit alpha	Cytochrome P450-cam
Astragali	MOL000296	hederagenin	Prostaglandin G/H synthase 2	Estrogen receptor
Astragali	MOL000296	hederagenin	Retinoic acid receptor RXR-alpha	Peroxisome proliferator activated receptor gamma
Astragali	MOL000296	hederagenin	CGMP-inhibited 3',5'-cyclic phosphodiesterase A	mRNA of Protein-tyrosine phosphatase, non-receptor type 1
Astragali	MOL000296	hederagenin	Sodium-dependent noradrenaline transporter	Mitogen-activated protein kinase 14
Astragali	MOL000296	hederagenin	Cytochrome P450-cam	Glycogen synthase kinase-3 beta
Astragali	MOL000033	(3S,8S,9S,10R,13R,14S,17R)-10,13-dimethyl-17-[(2R,5S)-5-propan-2-yloctan-2-yl]-2,3,4,7,8,9,11,12,14,15,16,17-dodecahydro-1H-cyclopenta[a]phenanthren-3-ol	Progesterone receptor	Phosphatidylinositol-4,5-bisphosphate 3-kinase catalytic subunit, gamma isoform
Astragali	MOL000354	isorhamnetin	Nitric oxide synthase, inducible	mRNA of PKA Catalytic Subunit C-alpha
Astragali	MOL000354	isorhamnetin	Prostaglandin G/H synthase 1	Proto-oncogene serine/threonine-protein kinase Pim-1
Astragali	MOL000354	isorhamnetin	Estrogen receptor	Cyclin-A2

Astragali	MOL000354	isorhamnetin	Androgen receptor	Glycogen phosphorylase, muscle form
Astragali	MOL000354	isorhamnetin	Peroxisome proliferator activated receptor gamma	Peroxisome proliferator activated receptor delta
Astragali	MOL000354	isorhamnetin	Prostaglandin G/H synthase 2	Aldose reductase
Astragali	MOL000354	isorhamnetin	mRNA of Protein-tyrosine phosphatase, non-receptor type 1	Nuclear receptor coactivator 1
Astragali	MOL000354	isorhamnetin	Estrogen receptor beta	Coagulation factor VII
Astragali	MOL000354	isorhamnetin	Dipeptidyl peptidase IV	Thrombin
Astragali	MOL000354	isorhamnetin	Mitogen-activated protein kinase 14	Nitric-oxide synthase, endothelial
Astragali	MOL000354	isorhamnetin	Glycogen synthase kinase-3 beta	Acetylcholinesterase
Astragali	MOL000354	isorhamnetin	Heat shock protein HSP 90	Amine oxidase [flavin-containing] B
Astragali	MOL000354	isorhamnetin	Cell division protein kinase 2	Transcription factor p65
Astragali	MOL000354	isorhamnetin	Phosphatidylinositol-4,5-bisphosphate 3-kinase catalytic subunit, gamma isoform	Xanthine dehydrogenase/oxidase
Astragali	MOL000354	isorhamnetin	mRNA of PKA Catalytic Subunit C-alpha	Neutrophil cytosol factor 1
Astragali	MOL000354	isorhamnetin	Trypsin-1	Oxidized low-density lipoprotein receptor 1
Astragali	MOL000354	isorhamnetin	Proto-oncogene serine/threonine-protein kinase Pim-1	Beta-1 adrenergic receptor
Astragali	MOL000354	isorhamnetin	Cyclin-A2	5-hydroxytryptamine receptor 3A
Astragali	MOL000354	isorhamnetin	Nuclear receptor coactivator 2	Alpha-2C adrenergic receptor
Astragali	MOL000354	isorhamnetin	Calmodulin	Beta-2 adrenergic receptor
Astragali	MOL000354	isorhamnetin	Glycogen phosphorylase, muscle form	Alpha-1D adrenergic receptor

Astragali	MOL000354	isorhamnetin	Peroxisome proliferator activated receptor delta	Mu-type opioid receptor
Astragali	MOL000354	isorhamnetin	Serine/threonine-protein kinase Chk1	Dopamine D1 receptor
Astragali	MOL000354	isorhamnetin	Aldose reductase	Potassium voltage-gated channel subfamily H member 2
Astragali	MOL000354	isorhamnetin	Nuclear receptor coactivator 1	Coagulation factor Xa
Astragali	MOL000354	isorhamnetin	Coagulation factor VII	Muscarinic acetylcholine receptor M5
Astragali	MOL000354	isorhamnetin	Thrombin	Muscarinic acetylcholine receptor M4
Astragali	MOL000354	isorhamnetin	Nitric-oxide synthase, endothelial	Delta-type opioid receptor
Astragali	MOL000354	isorhamnetin	Acetylcholinesterase	5-hydroxytryptamine 2A receptor
Astragali	MOL000354	isorhamnetin	Gamma-aminobutyric acid receptor subunit alpha-1	Alpha-1A adrenergic receptor
Astragali	MOL000354	isorhamnetin	Amine oxidase [flavin-containing] B	Sodium-dependent dopamine transporter
Astragali	MOL000354	isorhamnetin	Glutamate receptor 2	Sodium-dependent serotonin transporter
Astragali	MOL000354	isorhamnetin	Cytochrome P450-cam	Retinoic acid receptor RXR-beta
Astragali	MOL000354	isorhamnetin	Transcription factor p65	Calcium-activated potassium channel subunit alpha 1
Astragali	MOL000354	isorhamnetin	Xanthine dehydrogenase/oxidase	DNA topoisomerase II
Astragali	MOL000354	isorhamnetin	Neutrophil cytosol factor 1	Neuronal acetylcholine receptor protein, alpha-7 chain
Astragali	MOL000354	isorhamnetin	Oxidized low-density lipoprotein receptor 1	Vascular endothelial growth factor receptor 2

Astragali	MOL000371	3,9-di-O-methylnissolin	Nitric oxide synthase, inducible	Hepatocyte growth factor receptor
Astragali	MOL000371	3,9-di-O-methylnissolin	Prostaglandin G/H synthase 1	cAMP-dependent protein kinase inhibitor alpha
Astragali	MOL000371	3,9-di-O-methylnissolin	Muscarinic acetylcholine receptor M3	Beta-lactamase
Astragali	MOL000371	3,9-di-O-methylnissolin	Thrombin	Transcription factor AP-1
Astragali	MOL000371	3,9-di-O-methylnissolin	Muscarinic acetylcholine receptor M1	Peroxisome proliferator-activated receptor gamma
Astragali	MOL000371	3,9-di-O-methylnissolin	Estrogen receptor	Interleukin-4
Astragali	MOL000371	3,9-di-O-methylnissolin	Beta-1 adrenergic receptor	NAD-dependent deacetylase sirtuin-1
Astragali	MOL000371	3,9-di-O-methylnissolin	Sodium channel protein type 5 subunit alpha	ATP synthase subunit beta, mitochondrial
Astragali	MOL000371	3,9-di-O-methylnissolin	Prostaglandin G/H synthase 2	NADH-ubiquinone oxidoreductase chain 6
Astragali	MOL000371	3,9-di-O-methylnissolin	Nitric-oxide synthase, endothelial	3 beta-hydroxysteroid dehydrogenase/Delta 5-->4-isomerase type 2
Astragali	MOL000371	3,9-di-O-methylnissolin	5-hydroxytryptamine receptor 3A	3 beta-hydroxysteroid dehydrogenase/Delta 5-->4-isomerase type 1
Astragali	MOL000371	3,9-di-O-methylnissolin	Alpha-2C adrenergic receptor	Inhibitor of nuclear factor kappa-B kinase subunit beta
Astragali	MOL000371	3,9-di-O-methylnissolin	Retinoic acid receptor RXR-alpha	RAC-alpha serine/threonine-protein kinase
Astragali	MOL000371	3,9-di-O-methylnissolin	Acetylcholinesterase	Apoptosis regulator Bcl-2

Astragali	MOL000371	3,9-di-O-methylnissolin	CGMP-inhibited 3',5'-cyclic phosphodiesterase A	Apoptosis regulator BAX
Astragali	MOL000371	3,9-di-O-methylnissolin	Alpha-1B adrenergic receptor	Tumor necrosis factor
Astragali	MOL000371	3,9-di-O-methylnissolin	Beta-2 adrenergic receptor	Activator of 90 kDa heat shock protein ATPase homolog 1
Astragali	MOL000371	3,9-di-O-methylnissolin	Alpha-1D adrenergic receptor	Caspase-3
Astragali	MOL000371	3,9-di-O-methylnissolin	Mu-type opioid receptor	Mitogen-activated protein kinase 8
Astragali	MOL000371	3,9-di-O-methylnissolin	Gamma-aminobutyric acid receptor subunit alpha-1	Interstitial collagenase
Astragali	MOL000371	3,9-di-O-methylnissolin	Trypsin-1	Signal transducer and activator of transcription 1-alpha/beta
Astragali	MOL000371	3,9-di-O-methylnissolin	Nuclear receptor coactivator 2	Cell division control protein 2 homolog
Astragali	MOL000371	3,9-di-O-methylnissolin	Calmodulin	Heme oxygenase 1
Astragali	MOL000378	7-O-methylisomucronulatol	Nitric oxide synthase, inducible	Cytochrome P450 3A4
Astragali	MOL000378	7-O-methylisomucronulatol	Prostaglandin G/H synthase 1	Cytochrome P450 1A2
Astragali	MOL000378	7-O-methylisomucronulatol	Dopamine D1 receptor	Cytochrome P450 1A1

Astragali	MOL000378	7-O-methylisomucronulatol	Muscarinic acetylcholine receptor M3	Intercellular adhesion molecule 1
Astragali	MOL000378	7-O-methylisomucronulatol	Thrombin	E-selectin
Astragali	MOL000378	7-O-methylisomucronulatol	Potassium voltage-gated channel subfamily H member 2	Vascular cell adhesion protein 1
Astragali	MOL000378	7-O-methylisomucronulatol	Muscarinic acetylcholine receptor M1	Nuclear receptor subfamily 1 group I member 2
Astragali	MOL000378	7-O-methylisomucronulatol	Estrogen receptor	Cytochrome P450 1B1
Astragali	MOL000378	7-O-methylisomucronulatol	Androgen receptor	Arachidonate 5-lipoxygenase
Astragali	MOL000378	7-O-methylisomucronulatol	Beta-1 adrenergic receptor	Hyaluronan synthase 2
Astragali	MOL000378	7-O-methylisomucronulatol	Sodium channel protein type 5 subunit alpha	Glutathione S-transferase P
Astragali	MOL000378	7-O-methylisomucronulatol	Peroxisome proliferator activated receptor gamma	Aryl hydrocarbon receptor
Astragali	MOL000378	7-O-methylisomucronulatol	Coagulation factor Xa	26S proteasome non-ATPase regulatory subunit 3

Astragali	MOL000378	7-O-methylisomucronulatol	Muscarinic acetylcholine receptor M5	Solute carrier family 2, facilitated glucose transporter member 4
Astragali	MOL000378	7-O-methylisomucronulatol	Prostaglandin G/H synthase 2	Nuclear receptor subfamily 1 group I member 3
Astragali	MOL000378	7-O-methylisomucronulatol	Nitric-oxide synthase, endothelial	Insulin receptor
Astragali	MOL000378	7-O-methylisomucronulatol	Alpha-2C adrenergic receptor	Type I iodothyronine deiodinase
Astragali	MOL000378	7-O-methylisomucronulatol	Muscarinic acetylcholine receptor M4	Serine/threonine-protein phosphatase 2B catalytic subunit alpha isoform
Astragali	MOL000378	7-O-methylisomucronulatol	Retinoic acid receptor RXR-alpha	Peroxidase C1A
Astragali	MOL000378	7-O-methylisomucronulatol	Delta-type opioid receptor	Glutathione S-transferase Mu 1
Astragali	MOL000378	7-O-methylisomucronulatol	CGMP-inhibited 3',5'-cyclic phosphodiesterase A	Glutathione S-transferase Mu 2
Astragali	MOL000378	7-O-methylisomucronulatol	5-hydroxytryptamine 2A receptor	Aldo-keto reductase family 1 member C3
Astragali	MOL000378	7-O-methylisomucronulatol	Alpha-1A adrenergic receptor	Antileukoproteinase

Astragali	MOL000378	7-O-methylisomucronulatol	Muscarinic acetylcholine receptor M2	Stromelysin-1
Astragali	MOL000378	7-O-methylisomucronulatol	Alpha-1B adrenergic receptor	Epidermal growth factor receptor
Astragali	MOL000378	7-O-methylisomucronulatol	Sodium-dependent dopamine transporter	Vascular endothelial growth factor A
Astragali	MOL000378	7-O-methylisomucronulatol	Beta-2 adrenergic receptor	G1/S-specific cyclin-D1
Astragali	MOL000378	7-O-methylisomucronulatol	Alpha-1D adrenergic receptor	Bcl-2-like protein 1
Astragali	MOL000378	7-O-methylisomucronulatol	Sodium-dependent serotonin transporter	Proto-oncogene c-Fos
Astragali	MOL000378	7-O-methylisomucronulatol	Estrogen receptor beta	Cyclin-dependent kinase inhibitor 1
Astragali	MOL000378	7-O-methylisomucronulatol	Gamma-aminobutyric acid receptor subunit alpha-1	Eukaryotic translation initiation factor 6
Astragali	MOL000378	7-O-methylisomucronulatol	Dipeptidyl peptidase IV	Caspase-9
Astragali	MOL000378	7-O-methylisomucronulatol	Mitogen-activated protein kinase 14	Urokinase-type plasminogen activator

Astragali	MOL000378	7-O-methylisomucronulatol	Glycogen synthase kinase-3 beta	72 kDa type IV collagenase
Astragali	MOL000378	7-O-methylisomucronulatol	Heat shock protein HSP 90	Matrix metalloproteinase-9
Astragali	MOL000378	7-O-methylisomucronulatol	Cell division protein kinase 2	Mitogen-activated protein kinase 1
Astragali	MOL000378	7-O-methylisomucronulatol	Serine/threonine-protein kinase Chk1	Interleukin-10
Astragali	MOL000378	7-O-methylisomucronulatol	mRNA of PKA Catalytic Subunit C-alpha	Pro-epidermal growth factor
Astragali	MOL000378	7-O-methylisomucronulatol	Retinoic acid receptor RXR-beta	Retinoblastoma-associated protein
Astragali	MOL000378	7-O-methylisomucronulatol	Trypsin-1	Interleukin-6
Astragali	MOL000378	7-O-methylisomucronulatol	Proto-oncogene serine/threonine-protein kinase Pim-1	Cyclin-dependent kinase inhibitor 2A, isoforms 1/2/3
Astragali	MOL000378	7-O-methylisomucronulatol	Cyclin-A2	Cellular tumor antigen p53
Astragali	MOL000378	7-O-methylisomucronulatol	Nuclear receptor coactivator 2	ETS domain-containing protein Elk-1

Astragali	MOL000378	7-O-methylisomucronulatol	Calcium-activated potassium channel subunit alpha 1	NF-kappa-B inhibitor alpha
Astragali	MOL000378	7-O-methylisomucronulatol	Calmodulin	NADPH--cytochrome P450 reductase
Astragali	MOL000379	9,10-dimethoxypterocarpan-3-O-β-D-glucoside	Prostaglandin G/H synthase 2	Ornithine decarboxylase
Astragali	MOL000379	9,10-dimethoxypterocarpan-3-O-β-D-glucoside	DNA topoisomerase II	Caspase-8
Astragali	MOL000379	9,10-dimethoxypterocarpan-3-O-β-D-glucoside	Nuclear receptor coactivator 2	DNA topoisomerase 1
Astragali	MOL000380	(6aR,11aR)-9,10-dimethoxy-6a,11a-dihydro-6H-benzofurano[3,2-c]chromen-3-ol	Nitric oxide synthase, inducible	RAF proto-oncogene serine/threonine-protein kinase

Astragali	MOL000380	(6aR,11aR)-9,10-dimethoxy-6a,11a-dihydro-6H-benzofurano[3,2-c]chromen-3-ol	Prostaglandin G/H synthase 1	Superoxide dismutase [Cu-Zn]
Astragali	MOL000380	(6aR,11aR)-9,10-dimethoxy-6a,11a-dihydro-6H-benzofurano[3,2-c]chromen-3-ol	Muscarinic acetylcholine receptor M3	Protein kinase C alpha type
Astragali	MOL000380	(6aR,11aR)-9,10-dimethoxy-6a,11a-dihydro-6H-benzofurano[3,2-c]chromen-3-ol	Thrombin	Hypoxia-inducible factor 1-alpha

Astragali	MOL000380	(6aR,11aR)-9,10-dimethoxy-6a,11a-dihydro-6H-benzofurano[3,2-c]chromen-3-ol	Muscarinic acetylcholine receptor M1	Protein CBFA2T1
Astragali	MOL000380	(6aR,11aR)-9,10-dimethoxy-6a,11a-dihydro-6H-benzofurano[3,2-c]chromen-3-ol	Estrogen receptor	Probable E3 ubiquitin-protein ligase HERC5
Astragali	MOL000380	(6aR,11aR)-9,10-dimethoxy-6a,11a-dihydro-6H-benzofurano[3,2-c]chromen-3-ol	Sodium channel protein type 5 subunit alpha	78 kDa glucose-regulated protein

Astragali	MOL000380	(6aR,11aR)-9,10-dimethoxy-6a,11a-dihydro-6H-benzofurano[3,2-c]chromen-3-ol	Prostaglandin G/H synthase 2	Receptor tyrosine-protein kinase erbB-2
Astragali	MOL000380	(6aR,11aR)-9,10-dimethoxy-6a,11a-dihydro-6H-benzofurano[3,2-c]chromen-3-ol	5-hydroxytryptamine receptor 3A	Acetyl-CoA carboxylase 1
Astragali	MOL000380	(6aR,11aR)-9,10-dimethoxy-6a,11a-dihydro-6H-benzofurano[3,2-c]chromen-3-ol	Retinoic acid receptor RXR-alpha	Caveolin-1

Astragali	MOL000380	(6aR,11aR)-9,10-dimethoxy-6a,11a-dihydro-6H-benzofurano[3,2-c]chromen-3-ol	Acetylcholinesterase	Myc proto-oncogene protein
Astragali	MOL000380	(6aR,11aR)-9,10-dimethoxy-6a,11a-dihydro-6H-benzofurano[3,2-c]chromen-3-ol	Alpha-1B adrenergic receptor	Tissue factor
Astragali	MOL000380	(6aR,11aR)-9,10-dimethoxy-6a,11a-dihydro-6H-benzofurano[3,2-c]chromen-3-ol	Beta-2 adrenergic receptor	Gap junction alpha-1 protein

Astragali	MOL000380	(6aR,11aR)-9,10-dimethoxy-6a,11a-dihydro-6H-benzofurano[3,2-c]chromen-3-ol	Alpha-1D adrenergic receptor	Interleukin-1 beta
Astragali	MOL000380	(6aR,11aR)-9,10-dimethoxy-6a,11a-dihydro-6H-benzofurano[3,2-c]chromen-3-ol	Gamma-aminobutyric acid receptor subunit alpha-1	C-C motif chemokine 2
Astragali	MOL000380	(6aR,11aR)-9,10-dimethoxy-6a,11a-dihydro-6H-benzofurano[3,2-c]chromen-3-ol	Heat shock protein HSP 90	Prostaglandin E2 receptor EP3 subtype

Astragali	MOL000380	(6aR,11aR)-9,10-dimethoxy-6a,11a-dihydro-6H-benzofurano[3,2-c]chromen-3-ol	Neuronal acetylcholine receptor protein, alpha-7 chain	Interleukin-8
Astragali	MOL000380	(6aR,11aR)-9,10-dimethoxy-6a,11a-dihydro-6H-benzofurano[3,2-c]chromen-3-ol	Trypsin-1	Protein kinase C beta type
Astragali	MOL000380	(6aR,11aR)-9,10-dimethoxy-6a,11a-dihydro-6H-benzofurano[3,2-c]chromen-3-ol	Nuclear receptor coactivator 2	Baculoviral IAP repeat-containing protein 5

Astragali	MOL000380	(6aR,11aR)-9,10-dimethoxy-6a,11a-dihydro-6H-benzofurano[3,2-c]chromen-3-ol	Nuclear receptor coactivator 1	Dual oxidase 2
Astragali	MOL000380	(6aR,11aR)-9,10-dimethoxy-6a,11a-dihydro-6H-benzofurano[3,2-c]chromen-3-ol	Calmodulin	Nitric oxide synthase, endothelial
Astragali	MOL000380	(6aR,11aR)-9,10-dimethoxy-6a,11a-dihydro-6H-benzofurano[3,2-c]chromen-3-ol	Muscarinic acetylcholine receptor M4	Heat shock protein beta-1
Astragali	MOL000387	Bifendate	Prostaglandin G/H synthase 2	Transforming growth factor beta-1
Astragali	MOL000387	Bifendate	Vascular endothelial growth factor receptor 2	Estrogen sulfotransferase
Astragali	MOL000387	Bifendate	Hepatocyte growth factor receptor	Maltase-glucoamylase, intestinal

Astragali	MOL000387	Bifendate	Heat shock protein HSP 90	Interleukin-2
Astragali	MOL000387	Bifendate	Calcium-activated potassium channel subunit alpha 1	G2/mitotic-specific cyclin-B1
Astragali	MOL000387	Bifendate	Prostaglandin G/H synthase 1	Tissue-type plasminogen activator
Astragali	MOL000387	Bifendate	DNA topoisomerase II	Thrombomodulin
Astragali	MOL000392	formononetin	Nitric oxide synthase, inducible	Plasminogen activator inhibitor 1
Astragali	MOL000392	formononetin	Prostaglandin G/H synthase 1	Collagen alpha-1(I) chain
Astragali	MOL000392	formononetin	Muscarinic acetylcholine receptor M1	Interferon gamma
Astragali	MOL000392	formononetin	Estrogen receptor	Phosphatidylinositol-3,4,5-trisphosphate 3-phosphatase and dual-specificity protein phosphatase PTEN
Astragali	MOL000392	formononetin	Androgen receptor	Interleukin-1 alpha
Astragali	MOL000392	formononetin	Peroxisome proliferator activated receptor gamma	Myeloperoxidase
Astragali	MOL000392	formononetin	Prostaglandin G/H synthase 2	DNA topoisomerase 2-alpha
Astragali	MOL000392	formononetin	Retinoic acid receptor RXR-alpha	ATP-binding cassette sub-family G member 2
Astragali	MOL000392	formononetin	CGMP-inhibited 3',5'-cyclic phosphodiesterase A	Nuclear factor erythroid 2-related factor 2
Astragali	MOL000392	formononetin	Alpha-1A adrenergic receptor	NAD(P)H dehydrogenase [quinone] 1
Astragali	MOL000392	formononetin	Sodium-dependent dopamine transporter	Poly [ADP-ribose] polymerase 1
Astragali	MOL000392	formononetin	Beta-2 adrenergic receptor	Collagen alpha-1(III) chain
Astragali	MOL000392	formononetin	Sodium-dependent serotonin transporter	DNA gyrase subunit B
Astragali	MOL000392	formononetin	Estrogen receptor beta	C-X-C motif chemokine 11

Astragali	MOL000392	formononetin	Dipeptidyl peptidase IV	C-X-C motif chemokine 2
Astragali	MOL000392	formononetin	Mitogen-activated protein kinase 14	DDB1- and CUL4-associated factor 5
Astragali	MOL000392	formononetin	Glycogen synthase kinase-3 beta	Serine/threonine-protein kinase Chk2
Astragali	MOL000392	formononetin	Heat shock protein HSP 90	Claudin-4
Astragali	MOL000392	formononetin	Cell division protein kinase 2	Peroxisome proliferator-activated receptor alpha
Astragali	MOL000392	formononetin	Amine oxidase [flavin-containing] B	Peroxisome proliferator-activated receptor delta
Astragali	MOL000392	formononetin	Serine/threonine-protein kinase Chk1	Heat shock factor protein 1
Astragali	MOL000392	formononetin	mRNA of PKA Catalytic Subunit C-alpha	C-reactive protein
Astragali	MOL000392	formononetin	Trypsin-1	C-X-C motif chemokine 10
Astragali	MOL000392	formononetin	Proto-oncogene serine/threonine-protein kinase Pim-1	Inhibitor of nuclear factor kappa-B kinase subunit alpha
Astragali	MOL000392	formononetin	Cyclin-A2	Osteopontin
Astragali	MOL000392	formononetin	Calmodulin	Runt-related transcription factor 2
Astragali	MOL000392	formononetin	cAMP-dependent protein kinase inhibitor alpha	Ras association domain-containing protein 1
Astragali	MOL000392	formononetin	Thrombin	Transcription factor E2F1
Astragali	MOL000392	formononetin	Nitric-oxide synthase, endothelial	Transcription factor E2F2
Astragali	MOL000392	formononetin	Acetylcholinesterase	Prostatic acid phosphatase
Astragali	MOL000392	formononetin	Beta-lactamase	Cathepsin D
Astragali	MOL000392	formononetin	Transcription factor AP-1	Insulin-like growth factor-binding protein 3
Astragali	MOL000392	formononetin	Peroxisome proliferator-activated receptor gamma	Insulin-like growth factor II
Astragali	MOL000392	formononetin	Interleukin-4	CD40 ligand

Astragali	MOL000392	formononetin	NAD-dependent deacetylase sirtuin-1	Interferon regulatory factor 1
Astragali	MOL000392	formononetin	ATP synthase subunit beta, mitochondrial	Receptor tyrosine-protein kinase erbB-3
Astragali	MOL000392	formononetin	NADH-ubiquinone oxidoreductase chain 6	Serum paraoxonase/arylesterase 1
Astragali	MOL000392	formononetin	3 beta-hydroxysteroid dehydrogenase/Delta 5-->4-isomerase type 2	Procollagen C-endopeptidase enhancer 1
Astragali	MOL000392	formononetin	3 beta-hydroxysteroid dehydrogenase/Delta 5-->4-isomerase type 1	Puromycin-sensitive aminopeptidase
Astragali	MOL000417	Calycosin	Nitric oxide synthase, inducible	Hexokinase-2
Astragali	MOL000417	Calycosin	Prostaglandin G/H synthase 1	Homeobox protein Nkx-3.1
Astragali	MOL000417	Calycosin	Estrogen receptor	Ras GTPase-activating protein 1
Astragali	MOL000417	Calycosin	Androgen receptor	Carbonic anhydrase II
Astragali	MOL000417	Calycosin	Peroxisome proliferator activated receptor gamma	Neuronal acetylcholine receptor subunit alpha-2
Astragali	MOL000417	Calycosin	Prostaglandin G/H synthase 2	Microtubule-associated protein 2
Astragali	MOL000417	Calycosin	Retinoic acid receptor RXR-alpha	Alpha-2B adrenergic receptor
Astragali	MOL000417	Calycosin	CGMP-inhibited 3',5'-cyclic phosphodiesterase A	Voltage-dependent L-type calcium channel subunit alpha-1S
Astragali	MOL000417	Calycosin	Estrogen receptor beta	D(1B) dopamine receptor
Astragali	MOL000417	Calycosin	Dipeptidyl peptidase IV	Alpha-2A adrenergic receptor
Astragali	MOL000417	Calycosin	Mitogen-activated protein kinase 14	5-hydroxytryptamine 2B receptor
Astragali	MOL000417	Calycosin	Glycogen synthase kinase-3 beta	5-hydroxytryptamine 2C receptor

Astragali	MOL000417	Calycosin	Heat shock protein HSP 90	D(3) dopamine receptor
Astragali	MOL000417	Calycosin	Cell division protein kinase 2	5-hydroxytryptamine 7 receptor
Astragali	MOL000417	Calycosin	Serine/threonine-protein kinase Chk1	5-hydroxytryptamine 1D receptor
Astragali	MOL000417	Calycosin	mRNA of PKA Catalytic Subunit C-alpha	D(2) dopamine receptor
Astragali	MOL000417	Calycosin	Trypsin-1	5-hydroxytryptamine 1B receptor
Astragali	MOL000417	Calycosin	Proto-oncogene serine/threonine-protein kinase Pim-1	cAMP and cAMP-inhibited cGMP 3',5'-cyclic phosphodiesterase 10A
Astragali	MOL000417	Calycosin	Cyclin-A2	Mineralocorticoid receptor
Astragali	MOL000417	Calycosin	Nuclear receptor coactivator 2	Glucocorticoid receptor
Astragali	MOL000417	Calycosin	Calmodulin	D(4) dopamine receptor
Astragali	MOL000417	Calycosin	Beta-2 adrenergic receptor	Cytochrome P450 2B1
Astragali	MOL000422	kaempferol	Nitric oxide synthase, inducible	Serum albumin
Astragali	MOL000422	kaempferol	Prostaglandin G/H synthase 1	Catenin beta-1
Astragali	MOL000422	kaempferol	Androgen receptor	Caspase-7
Astragali	MOL000422	kaempferol	Peroxisome proliferator activated receptor gamma	Stromelysin-2
Astragali	MOL000422	kaempferol	Prostaglandin G/H synthase 2	Leukotriene A-4 hydrolase
Astragali	MOL000422	kaempferol	Heat shock protein HSP 90	Amine oxidase [flavin-containing] A
Astragali	MOL000422	kaempferol	Phosphatidylinositol-4,5-bisphosphate 3-kinase catalytic subunit, gamma isoform	Chymotrypsinogen B
Astragali	MOL000422	kaempferol	mRNA of PKA Catalytic Subunit C-alpha	Fatty acid synthase

Astragali	MOL000422	kaempferol	Nuclear receptor coactivator 2	Glucose-6-phosphate 1-dehydrogenase
Astragali	MOL000422	kaempferol	Dipeptidyl peptidase IV	Endothelin-converting enzyme 1
Astragali	MOL000422	kaempferol	Trypsin-1	Medium-chain specific acyl-CoA dehydrogenase, mitochondrial
Astragali	MOL000422	kaempferol	Progesterone receptor	Cytochrome P450 2B6
Astragali	MOL000422	kaempferol	Thrombin	UDP-glucuronosyltransferase 1-1
Astragali	MOL000422	kaempferol	Muscarinic acetylcholine receptor M1	Sterol regulatory element-binding protein 1
Astragali	MOL000422	kaempferol	Nitric-oxide synthase, endothelial	NADPH oxidase 3
Astragali	MOL000422	kaempferol	Gamma-aminobutyric-acid receptor alpha-2 subunit	NADPH oxidase 1
Astragali	MOL000422	kaempferol	Acetylcholinesterase	Peroxisomal acyl-coenzyme A oxidase 1
Astragali	MOL000422	kaempferol	Sodium-dependent noradrenaline transporter	ATP-citrate synthase
Astragali	MOL000422	kaempferol	Muscarinic acetylcholine receptor M2	Peroxisomal bifunctional enzyme
Astragali	MOL000422	kaempferol	Alpha-1B adrenergic receptor	Methylglutaconyl-CoA hydratase, mitochondrial
Astragali	MOL000422	kaempferol	Gamma-aminobutyric acid receptor subunit alpha-1	Trifunctional enzyme subunit beta, mitochondrial
Astragali	MOL000422	kaempferol	DNA topoisomerase II	2,4-dienoyl-CoA reductase, mitochondrial
Astragali	MOL000422	kaempferol	Coagulation factor VII	3,2-trans-enoyl-CoA isomerase, mitochondrial

Astragali	MOL000422	kaempferol	Calmodulin	4-aminobutyrate aminotransferase, mitochondrial
Astragali	MOL000422	kaempferol	Transcription factor p65	Alcohol dehydrogenase 1A
Astragali	MOL000422	kaempferol	Inhibitor of nuclear factor kappa-B kinase subunit beta	Bacillolysin
Astragali	MOL000422	kaempferol	RAC-alpha serine/threonine-protein kinase	Heparanase
Astragali	MOL000422	kaempferol	Apoptosis regulator Bcl-2	Immediate early response 3-interacting protein 1
Astragali	MOL000422	kaempferol	Apoptosis regulator BAX	CD44 antigen
Astragali	MOL000422	kaempferol	Tumor necrosis factor	Pregnane X receptor
Astragali	MOL000422	kaempferol	Transcription factor AP-1	Beta-secretase
Astragali	MOL000422	kaempferol	Activator of 90 kDa heat shock protein ATPase homolog 1	Type IV phosphodiesterase
Astragali	MOL000422	kaempferol	Caspase-3	Gamma-aminobutyric acid type B receptor subunit 1
Astragali	MOL000422	kaempferol	Mitogen-activated protein kinase 8	Bone morphogenetic protein receptor type-2
Astragali	MOL000422	kaempferol	Xanthine dehydrogenase/oxidase	Metabotropic glutamate receptor 5
Astragali	MOL000422	kaempferol	Interstitial collagenase	Progonadoliberin-1
Astragali	MOL000422	kaempferol	Signal transducer and activator of transcription 1-alpha/beta	Aldehyde dehydrogenase, dimeric NADP-preferring
Astragali	MOL000422	kaempferol	Cell division control protein 2 homolog	Gonadotropin-releasing hormone receptor
Astragali	MOL000422	kaempferol	Peroxisome proliferator-activated receptor gamma	Corticoliberin
Astragali	MOL000422	kaempferol	Heme oxygenase 1	Glutamate [NMDA] receptor subunit epsilon-4

Astragali	MOL000422	kaempferol	Cytochrome P450 3A4	Gap junction beta-1 protein
Astragali	MOL000422	kaempferol	Cytochrome P450 1A2	Metabotropic glutamate receptor 1
Astragali	MOL000422	kaempferol	Cytochrome P450 1A1	Transitional endoplasmic reticulum ATPase
Astragali	MOL000422	kaempferol	Intercellular adhesion molecule 1	
Astragali	MOL000422	kaempferol	E-selectin	
Astragali	MOL000422	kaempferol	Vascular cell adhesion protein 1	
Astragali	MOL000422	kaempferol	Nuclear receptor subfamily 1 group I member 2	
Astragali	MOL000422	kaempferol	Cytochrome P450 1B1	
Astragali	MOL000422	kaempferol	Arachidonate 5-lipoxygenase	
Astragali	MOL000422	kaempferol	Hyaluronan synthase 2	
Astragali	MOL000422	kaempferol	Glutathione S-transferase P	
Astragali	MOL000422	kaempferol	Aryl hydrocarbon receptor	
Astragali	MOL000422	kaempferol	26S proteasome non-ATPase regulatory subunit 3	
Astragali	MOL000422	kaempferol	Solute carrier family 2, facilitated glucose transporter member 4	
Astragali	MOL000422	kaempferol	Nuclear receptor subfamily 1 group I member 3	
Astragali	MOL000422	kaempferol	Insulin receptor	
Astragali	MOL000422	kaempferol	Type I iodothyronine deiodinase	
Astragali	MOL000422	kaempferol	Serine/threonine-protein phosphatase 2B catalytic subunit alpha isoform	
Astragali	MOL000422	kaempferol	Peroxidase C1A	
Astragali	MOL000422	kaempferol	Glutathione S-transferase Mu 1	
Astragali	MOL000422	kaempferol	Glutathione S-transferase Mu 2	
Astragali	MOL000422	kaempferol	Aldo-keto reductase family 1 member C3	

Astragali	MOL000422	kaempferol	Antileukoproteinase
Astragali	MOL000433	FA	Cell division protein kinase 2
Astragali	MOL000433	FA	Thrombin
Astragali	MOL000433	FA	Glycogen synthase kinase-3 beta
Astragali	MOL000439	isomucronulatol-7,2'-di-O-glucosiole	DNA topoisomerase II
Astragali	MOL000442	1,7-Dihydroxy-3,9-dimethoxy pterocarpene	Prostaglandin G/H synthase 2
Astragali	MOL000442	1,7-Dihydroxy-3,9-dimethoxy pterocarpene	Retinoic acid receptor RXR-alpha
Astragali	MOL000442	1,7-Dihydroxy-3,9-dimethoxy pterocarpene	Heat shock protein HSP 90
Astragali	MOL000442	1,7-Dihydroxy-3,9-dimethoxy pterocarpene	Trypsin-1
Astragali	MOL000098	quercetin	Prostaglandin G/H synthase 1
Astragali	MOL000098	quercetin	Androgen receptor
Astragali	MOL000098	quercetin	Peroxisome proliferator activated receptor gamma
Astragali	MOL000098	quercetin	Prostaglandin G/H synthase 2
Astragali	MOL000098	quercetin	Heat shock protein HSP 90
Astragali	MOL000098	quercetin	Phosphatidylinositol-4,5-bisphosphate 3-kinase catalytic subunit, gamma isoform
Astragali	MOL000098	quercetin	Nuclear receptor coactivator 2
Astragali	MOL000098	quercetin	Dipeptidyl peptidase IV

Astragali	MOL000098	quercetin	Aldose reductase
Astragali	MOL000098	quercetin	Trypsin-1
Astragali	MOL000098	quercetin	DNA topoisomerase II
Astragali	MOL000098	quercetin	Thrombin
Astragali	MOL000098	quercetin	Potassium voltage-gated channel subfamily H member 2
Astragali	MOL000098	quercetin	Sodium channel protein type 5 subunit alpha
Astragali	MOL000098	quercetin	Coagulation factor Xa
Astragali	MOL000098	quercetin	Beta-2 adrenergic receptor
Astragali	MOL000098	quercetin	Stromelysin-1
Astragali	MOL000098	quercetin	mRNA of PKA Catalytic Subunit C-alpha
Astragali	MOL000098	quercetin	Coagulation factor VII
Astragali	MOL000098	quercetin	Nitric-oxide synthase, endothelial
Astragali	MOL000098	quercetin	Retinoic acid receptor RXR-alpha
Astragali	MOL000098	quercetin	Acetylcholinesterase
Astragali	MOL000098	quercetin	Gamma-aminobutyric acid receptor subunit alpha-1
Astragali	MOL000098	quercetin	Amine oxidase [flavin-containing] B
Astragali	MOL000098	quercetin	Transcription factor p65
Astragali	MOL000098	quercetin	Epidermal growth factor receptor
Astragali	MOL000098	quercetin	RAC-alpha serine/threonine-protein kinase
Astragali	MOL000098	quercetin	Vascular endothelial growth factor A
Astragali	MOL000098	quercetin	G1/S-specific cyclin-D1
Astragali	MOL000098	quercetin	Apoptosis regulator Bcl-2
Astragali	MOL000098	quercetin	Bcl-2-like protein 1
Astragali	MOL000098	quercetin	Proto-oncogene c-Fos
Astragali	MOL000098	quercetin	Cyclin-dependent kinase inhibitor 1

Astragali	MOL000098	quercetin	Eukaryotic translation initiation factor 6
Astragali	MOL000098	quercetin	Apoptosis regulator BAX
Astragali	MOL000098	quercetin	Caspase-9
Astragali	MOL000098	quercetin	Urokinase-type plasminogen activator
Astragali	MOL000098	quercetin	72 kDa type IV collagenase
Astragali	MOL000098	quercetin	Matrix metalloproteinase-9
Astragali	MOL000098	quercetin	Mitogen-activated protein kinase 1
Astragali	MOL000098	quercetin	Interleukin-10
Astragali	MOL000098	quercetin	Pro-epidermal growth factor
Astragali	MOL000098	quercetin	Retinoblastoma-associated protein
Astragali	MOL000098	quercetin	Tumor necrosis factor
Astragali	MOL000098	quercetin	Transcription factor AP-1
Astragali	MOL000098	quercetin	Interleukin-6
Astragali	MOL000098	quercetin	Cyclin-dependent kinase inhibitor 2A, isoforms 1/2/3
Astragali	MOL000098	quercetin	Activator of 90 kDa heat shock protein ATPase homolog 1
Astragali	MOL000098	quercetin	Caspase-3
Astragali	MOL000098	quercetin	Cellular tumor antigen p53
Astragali	MOL000098	quercetin	ETS domain-containing protein Elk-1
Astragali	MOL000098	quercetin	NF-kappa-B inhibitor alpha
Astragali	MOL000098	quercetin	NADPH--cytochrome P450 reductase
Astragali	MOL000098	quercetin	Ornithine decarboxylase
Astragali	MOL000098	quercetin	Xanthine dehydrogenase/oxidase
Astragali	MOL000098	quercetin	Caspase-8
Astragali	MOL000098	quercetin	DNA topoisomerase 1
Astragali	MOL000098	quercetin	RAF proto-oncogene serine/threonine-protein kinase

Astragali	MOL000098	quercetin	Superoxide dismutase [Cu-Zn]
Astragali	MOL000098	quercetin	Protein kinase C alpha type
Astragali	MOL000098	quercetin	Interstitial collagenase
Astragali	MOL000098	quercetin	Hypoxia-inducible factor 1-alpha
Astragali	MOL000098	quercetin	Signal transducer and activator of transcription 1-alpha/beta
Astragali	MOL000098	quercetin	Protein CBFA2T1
Astragali	MOL000098	quercetin	Probable E3 ubiquitin-protein ligase HERC5
Astragali	MOL000098	quercetin	Cell division control protein 2 homolog
Astragali	MOL000098	quercetin	78 kDa glucose-regulated protein
Astragali	MOL000098	quercetin	Receptor tyrosine-protein kinase erbB-2
Astragali	MOL000098	quercetin	Peroxisome proliferator-activated receptor gamma
Astragali	MOL000098	quercetin	Acetyl-CoA carboxylase 1
Astragali	MOL000098	quercetin	Heme oxygenase 1
Astragali	MOL000098	quercetin	Cytochrome P450 3A4
Astragali	MOL000098	quercetin	Cytochrome P450 1A2
Astragali	MOL000098	quercetin	Caveolin-1
Astragali	MOL000098	quercetin	Myc proto-oncogene protein
Astragali	MOL000098	quercetin	Tissue factor
Astragali	MOL000098	quercetin	Gap junction alpha-1 protein
Astragali	MOL000098	quercetin	Cytochrome P450 1A1
Astragali	MOL000098	quercetin	Intercellular adhesion molecule 1
Astragali	MOL000098	quercetin	Interleukin-1 beta
Astragali	MOL000098	quercetin	C-C motif chemokine 2
Astragali	MOL000098	quercetin	E-selectin
Astragali	MOL000098	quercetin	Vascular cell adhesion protein 1

Astragali	MOL000098	quercetin	Prostaglandin E2 receptor EP3 subtype
Astragali	MOL000098	quercetin	Interleukin-8
Astragali	MOL000098	quercetin	Protein kinase C beta type
Astragali	MOL000098	quercetin	Baculoviral IAP repeat-containing protein 5
Astragali	MOL000098	quercetin	Dual oxidase 2
Astragali	MOL000098	quercetin	Nitric oxide synthase, endothelial
Astragali	MOL000098	quercetin	Heat shock protein beta-1
Astragali	MOL000098	quercetin	Transforming growth factor beta-1
Astragali	MOL000098	quercetin	Estrogen sulfotransferase
Astragali	MOL000098	quercetin	Maltase-glucoamylase, intestinal
Astragali	MOL000098	quercetin	Interleukin-2
Astragali	MOL000098	quercetin	Nuclear receptor subfamily 1 group I member 2
Astragali	MOL000098	quercetin	Cytochrome P450 1B1
Astragali	MOL000098	quercetin	G2/mitotic-specific cyclin-B1
Astragali	MOL000098	quercetin	Tissue-type plasminogen activator
Astragali	MOL000098	quercetin	Thrombomodulin
Astragali	MOL000098	quercetin	Plasminogen activator inhibitor 1
Astragali	MOL000098	quercetin	Collagen alpha-1(I) chain
Astragali	MOL000098	quercetin	Interferon gamma
Astragali	MOL000098	quercetin	Arachidonate 5-lipoxygenase
Astragali	MOL000098	quercetin	Phosphatidylinositol-3,4,5-trisphosphate 3-phosphatase and dual-specificity protein phosphatase PTEN
Astragali	MOL000098	quercetin	Interleukin-1 alpha
Astragali	MOL000098	quercetin	Myeloperoxidase
Astragali	MOL000098	quercetin	DNA topoisomerase 2-alpha

Astragali	MOL000098	quercetin	Neutrophil cytosol factor 1
Astragali	MOL000098	quercetin	ATP-binding cassette sub-family G member 2
Astragali	MOL000098	quercetin	Hyaluronan synthase 2
Astragali	MOL000098	quercetin	Glutathione S-transferase P
Astragali	MOL000098	quercetin	Nuclear factor erythroid 2-related factor 2
Astragali	MOL000098	quercetin	NAD(P)H dehydrogenase [quinone] 1
Astragali	MOL000098	quercetin	Poly [ADP-ribose] polymerase 1
Astragali	MOL000098	quercetin	Aryl hydrocarbon receptor
Astragali	MOL000098	quercetin	26S proteasome non-ATPase regulatory subunit 3
Astragali	MOL000098	quercetin	Solute carrier family 2, facilitated glucose transporter member 4
Astragali	MOL000098	quercetin	Collagen alpha-1(III) chain
Astragali	MOL000098	quercetin	DNA gyrase subunit B
Astragali	MOL000098	quercetin	C-X-C motif chemokine 11
Astragali	MOL000098	quercetin	C-X-C motif chemokine 2
Astragali	MOL000098	quercetin	DDB1- and CUL4-associated factor 5
Astragali	MOL000098	quercetin	Nuclear receptor subfamily 1 group I member 3
Astragali	MOL000098	quercetin	Serine/threonine-protein kinase Chk2
Astragali	MOL000098	quercetin	Insulin receptor
Astragali	MOL000098	quercetin	Claudin-4
Astragali	MOL000098	quercetin	Peroxisome proliferator-activated receptor alpha
Astragali	MOL000098	quercetin	Peroxisome proliferator-activated receptor delta

Astragali	MOL000098	quercetin	Heat shock factor protein 1
Astragali	MOL000098	quercetin	C-reactive protein
Astragali	MOL000098	quercetin	C-X-C motif chemokine 10
Astragali	MOL000098	quercetin	Inhibitor of nuclear factor kappa-B kinase subunit alpha
Astragali	MOL000098	quercetin	Osteopontin
Astragali	MOL000098	quercetin	Runt-related transcription factor 2
Astragali	MOL000098	quercetin	Ras association domain-containing protein 1
Astragali	MOL000098	quercetin	Transcription factor E2F1
Astragali	MOL000098	quercetin	Transcription factor E2F2
Astragali	MOL000098	quercetin	Prostatic acid phosphatase
Astragali	MOL000098	quercetin	Cathepsin D
Astragali	MOL000098	quercetin	Insulin-like growth factor-binding protein 3
Astragali	MOL000098	quercetin	Insulin-like growth factor II
Astragali	MOL000098	quercetin	CD40 ligand
Astragali	MOL000098	quercetin	Interferon regulatory factor 1
Astragali	MOL000098	quercetin	Receptor tyrosine-protein kinase erbB-3
Astragali	MOL000098	quercetin	Serum paraoxonase/arylesterase 1
Astragali	MOL000098	quercetin	Type I iodothyronine deiodinase
Astragali	MOL000098	quercetin	Procollagen C-endopeptidase enhancer 1
Astragali	MOL000098	quercetin	Puromycin-sensitive aminopeptidase
Astragali	MOL000098	quercetin	Hexokinase-2
Astragali	MOL000098	quercetin	Homeobox protein Nkx-3.1
Astragali	MOL000098	quercetin	Ras GTPase-activating protein 1
Astragali	MOL000098	quercetin	Peroxidase C1A
Astragali	MOL000098	quercetin	Glutathione S-transferase Mu 1

Astragali	MOL000098	quercetin	Glutathione S-transferase Mu 2
Salviae liguliobae	MOL007059	3-beta-Hydroxymethyllenetanshiquinone	Dopamine D1 receptor
Salviae liguliobae	MOL007059	3-beta-Hydroxymethyllenetanshiquinone	Thrombin
Salviae liguliobae	MOL007059	3-beta-Hydroxymethyllenetanshiquinone	Muscarinic acetylcholine receptor M1
Salviae liguliobae	MOL007059	3-beta-Hydroxymethyllenetanshiquinone	Prostaglandin G/H synthase 2
Salviae liguliobae	MOL007059	3-beta-Hydroxymethyllenetanshiquinone	Carbonic anhydrase II
Salviae liguliobae	MOL007059	3-beta-Hydroxymethyllenetanshiquinone	Retinoic acid receptor RXR-alpha
Salviae liguliobae	MOL007059	3-beta-Hydroxymethyllenetanshiquinone	Delta-type opioid receptor

Salviae liguliobae	MOL007059	3-beta-Hydroxymethyllenetanshiquinone	Acetylcholinesterase
Salviae liguliobae	MOL007059	3-beta-Hydroxymethyllenetanshiquinone	Alpha-1A adrenergic receptor
Salviae liguliobae	MOL007059	3-beta-Hydroxymethyllenetanshiquinone	Beta-2 adrenergic receptor
Salviae liguliobae	MOL007059	3-beta-Hydroxymethyllenetanshiquinone	Mu-type opioid receptor
Salviae liguliobae	MOL007059	3-beta-Hydroxymethyllenetanshiquinone	Dipeptidyl peptidase IV
Salviae liguliobae	MOL007059	3-beta-Hydroxymethyllenetanshiquinone	Heat shock protein HSP 90
Salviae liguliobae	MOL007059	3-beta-Hydroxymethyllenetanshiquinone	Neuronal acetylcholine receptor protein, alpha-7 chain

Salviae liguliobae	MOL007059	3-beta-Hydroxymethyllenetanshiquinone	Ig gamma-1 chain C region
Salviae liguliobae	MOL007059	3-beta-Hydroxymethyllenetanshiquinone	Trypsin-1
Salviae liguliobae	MOL007059	3-beta-Hydroxymethyllenetanshiquinone	Nuclear receptor coactivator 1
Eucommiae corte:	MOL002058	40957-99-1	Potassium voltage-gated channel subfamily H member 2
Eucommiae corte:	MOL002058	40957-99-1	Sodium channel protein type 5 subunit alpha
Eucommiae corte:	MOL002058	40957-99-1	Coagulation factor Xa
Eucommiae corte:	MOL002058	40957-99-1	Prostaglandin G/H synthase 2
Eucommiae corte:	MOL002058	40957-99-1	DNA topoisomerase II
Eucommiae corte:	MOL002058	40957-99-1	Calmodulin
Eucommiae corte:	MOL002058	40957-99-1	Prostaglandin G/H synthase 1
Eucommiae corte:	MOL002058	40957-99-1	Nitric-oxide synthase, endothelial
Eucommiae corte:	MOL002058	40957-99-1	Heat shock protein HSP 90
Eucommiae corte:	MOL002058	40957-99-1	Nuclear receptor coactivator 2
Eucommiae corte:	MOL002058	40957-99-1	Coagulation factor VII
Eucommiae corte:	MOL000211	Mairin	Progesterone receptor
Eucommiae corte:	MOL000358	beta-sitosterol	Progesterone receptor
Eucommiae corte:	MOL000358	beta-sitosterol	Nuclear receptor coactivator 2
Eucommiae corte:	MOL000358	beta-sitosterol	Prostaglandin G/H synthase 1
Eucommiae corte:	MOL000358	beta-sitosterol	Prostaglandin G/H synthase 2

Eucommiae corte:	MOL000358	beta-sitosterol	Heat shock protein HSP 90
Eucommiae corte:	MOL000358	beta-sitosterol	Phosphatidylinositol-4,5-bisphosphate 3-kinase catalytic subunit, gamma isoform
Eucommiae corte:	MOL000358	beta-sitosterol	Potassium voltage-gated channel subfamily H member 2
Eucommiae corte:	MOL000358	beta-sitosterol	mRNA of PKA Catalytic Subunit C-alpha
Eucommiae corte:	MOL000358	beta-sitosterol	Dopamine D1 receptor
Eucommiae corte:	MOL000358	beta-sitosterol	Muscarinic acetylcholine receptor M3
Eucommiae corte:	MOL000358	beta-sitosterol	Muscarinic acetylcholine receptor M1
Eucommiae corte:	MOL000358	beta-sitosterol	Sodium channel protein type 5 subunit alpha
Eucommiae corte:	MOL000358	beta-sitosterol	Gamma-aminobutyric-acid receptor alpha-2 subunit
Eucommiae corte:	MOL000358	beta-sitosterol	Muscarinic acetylcholine receptor M4
Eucommiae corte:	MOL000358	beta-sitosterol	CGMP-inhibited 3',5'-cyclic phosphodiesterase A
Eucommiae corte:	MOL000358	beta-sitosterol	5-hydroxytryptamine 2A receptor
Eucommiae corte:	MOL000358	beta-sitosterol	Gamma-aminobutyric-acid receptor alpha-5 subunit
Eucommiae corte:	MOL000358	beta-sitosterol	Alpha-1A adrenergic receptor
Eucommiae corte:	MOL000358	beta-sitosterol	Gamma-aminobutyric-acid receptor alpha-3 subunit
Eucommiae corte:	MOL000358	beta-sitosterol	Muscarinic acetylcholine receptor M2
Eucommiae corte:	MOL000358	beta-sitosterol	Alpha-1B adrenergic receptor
Eucommiae corte:	MOL000358	beta-sitosterol	Beta-2 adrenergic receptor
Eucommiae corte:	MOL000358	beta-sitosterol	Neuronal acetylcholine receptor subunit alpha-2

Eucommiae corte:	MOL000358	beta-sitosterol	Sodium-dependent serotonin transporter
Eucommiae corte:	MOL000358	beta-sitosterol	Mu-type opioid receptor
Eucommiae corte:	MOL000358	beta-sitosterol	Gamma-aminobutyric acid receptor subunit alpha-1
Eucommiae corte:	MOL000358	beta-sitosterol	Neuronal acetylcholine receptor protein, alpha-7 chain
Eucommiae corte:	MOL000358	beta-sitosterol	Cytochrome P450-cam
Eucommiae corte:	MOL000358	beta-sitosterol	Apoptosis regulator Bcl-2
Eucommiae corte:	MOL000358	beta-sitosterol	Apoptosis regulator BAX
Eucommiae corte:	MOL000358	beta-sitosterol	Caspase-9
Eucommiae corte:	MOL000358	beta-sitosterol	Transcription factor AP-1
Eucommiae corte:	MOL000358	beta-sitosterol	Caspase-3
Eucommiae corte:	MOL000358	beta-sitosterol	Caspase-8
Eucommiae corte:	MOL000358	beta-sitosterol	Protein kinase C alpha type
Eucommiae corte:	MOL000358	beta-sitosterol	Transforming growth factor beta-1
Eucommiae corte:	MOL000358	beta-sitosterol	Serum paraoxonase/arylesterase 1
Eucommiae corte:	MOL000358	beta-sitosterol	Microtubule-associated protein 2
Eucommiae corte:	MOL000422	kaempferol	Nitric oxide synthase, inducible
Eucommiae corte:	MOL000422	kaempferol	Prostaglandin G/H synthase 1
Eucommiae corte:	MOL000422	kaempferol	Androgen receptor
Eucommiae corte:	MOL000422	kaempferol	Peroxisome proliferator activated receptor gamma
Eucommiae corte:	MOL000422	kaempferol	Prostaglandin G/H synthase 2
Eucommiae corte:	MOL000422	kaempferol	Heat shock protein HSP 90
Eucommiae corte:	MOL000422	kaempferol	Phosphatidylinositol-4,5-bisphosphate 3-kinase catalytic subunit, gamma isoform
Eucommiae corte:	MOL000422	kaempferol	mRNA of PKA Catalytic Subunit C-alpha

Eucommiae corte:	MOL000422	kaempferol	Nuclear receptor coactivator 2
Eucommiae corte:	MOL000422	kaempferol	Dipeptidyl peptidase IV
Eucommiae corte:	MOL000422	kaempferol	Trypsin-1
Eucommiae corte:	MOL000422	kaempferol	Progesterone receptor
Eucommiae corte:	MOL000422	kaempferol	Thrombin
Eucommiae corte:	MOL000422	kaempferol	Muscarinic acetylcholine receptor M1
Eucommiae corte:	MOL000422	kaempferol	Nitric-oxide synthase, endothelial
Eucommiae corte:	MOL000422	kaempferol	Gamma-aminobutyric-acid receptor alpha-2 subunit
Eucommiae corte:	MOL000422	kaempferol	Acetylcholinesterase
Eucommiae corte:	MOL000422	kaempferol	Sodium-dependent noradrenaline transporter
Eucommiae corte:	MOL000422	kaempferol	Muscarinic acetylcholine receptor M2
Eucommiae corte:	MOL000422	kaempferol	Alpha-1B adrenergic receptor
Eucommiae corte:	MOL000422	kaempferol	Gamma-aminobutyric acid receptor subunit alpha-1
Eucommiae corte:	MOL000422	kaempferol	DNA topoisomerase II
Eucommiae corte:	MOL000422	kaempferol	Coagulation factor VII
Eucommiae corte:	MOL000422	kaempferol	Calmodulin
Eucommiae corte:	MOL000422	kaempferol	Transcription factor p65
Eucommiae corte:	MOL000422	kaempferol	Inhibitor of nuclear factor kappa-B kinase subunit beta
Eucommiae corte:	MOL000422	kaempferol	RAC-alpha serine/threonine-protein kinase
Eucommiae corte:	MOL000422	kaempferol	Apoptosis regulator Bcl-2
Eucommiae corte:	MOL000422	kaempferol	Apoptosis regulator BAX
Eucommiae corte:	MOL000422	kaempferol	Tumor necrosis factor
Eucommiae corte:	MOL000422	kaempferol	Transcription factor AP-1
Eucommiae corte:	MOL000422	kaempferol	Activator of 90 kDa heat shock protein ATPase homolog 1

Eucommiae corte:	MOL000422	kaempferol	Caspase-3
Eucommiae corte:	MOL000422	kaempferol	Mitogen-activated protein kinase 8
Eucommiae corte:	MOL000422	kaempferol	Xanthine dehydrogenase/oxidase
Eucommiae corte:	MOL000422	kaempferol	Interstitial collagenase
Eucommiae corte:	MOL000422	kaempferol	Signal transducer and activator of transcription 1-alpha/beta
Eucommiae corte:	MOL000422	kaempferol	Cell division control protein 2 homolog
Eucommiae corte:	MOL000422	kaempferol	Peroxisome proliferator-activated receptor gamma
Eucommiae corte:	MOL000422	kaempferol	Heme oxygenase 1
Eucommiae corte:	MOL000422	kaempferol	Cytochrome P450 3A4
Eucommiae corte:	MOL000422	kaempferol	Cytochrome P450 1A2
Eucommiae corte:	MOL000422	kaempferol	Cytochrome P450 1A1
Eucommiae corte:	MOL000422	kaempferol	Intercellular adhesion molecule 1
Eucommiae corte:	MOL000422	kaempferol	E-selectin
Eucommiae corte:	MOL000422	kaempferol	Vascular cell adhesion protein 1
Eucommiae corte:	MOL000422	kaempferol	Nuclear receptor subfamily 1 group I member 2
Eucommiae corte:	MOL000422	kaempferol	Cytochrome P450 1B1
Eucommiae corte:	MOL000422	kaempferol	Arachidonate 5-lipoxygenase
Eucommiae corte:	MOL000422	kaempferol	Hyaluronan synthase 2
Eucommiae corte:	MOL000422	kaempferol	Glutathione S-transferase P
Eucommiae corte:	MOL000422	kaempferol	Aryl hydrocarbon receptor
Eucommiae corte:	MOL000422	kaempferol	26S proteasome non-ATPase regulatory subunit 3
Eucommiae corte:	MOL000422	kaempferol	Solute carrier family 2, facilitated glucose transporter member 4
Eucommiae corte:	MOL000422	kaempferol	Nuclear receptor subfamily 1 group I member 3

Eucommiae corte:	MOL000422	kaempferol	Insulin receptor
Eucommiae corte:	MOL000422	kaempferol	Type I iodothyronine deiodinase
Eucommiae corte:	MOL000422	kaempferol	Serine/threonine-protein phosphatase 2B catalytic subunit alpha isoform
Eucommiae corte:	MOL000422	kaempferol	Peroxidase C1A
Eucommiae corte:	MOL000422	kaempferol	Glutathione S-transferase Mu 1
Eucommiae corte:	MOL000422	kaempferol	Glutathione S-transferase Mu 2
Eucommiae corte:	MOL000422	kaempferol	Aldo-keto reductase family 1 member C3
Eucommiae corte:	MOL000422	kaempferol	Antileukoproteinase
Eucommiae corte:	MOL004367	olivil	Prostaglandin G/H synthase 2
Eucommiae corte:	MOL004367	olivil	Heat shock protein HSP 90
Eucommiae corte:	MOL004367	olivil	Nuclear receptor coactivator 2
Eucommiae corte:	MOL004367	olivil	Calmodulin
Eucommiae corte:	MOL000443	Erythraline	Prostaglandin G/H synthase 1
Eucommiae corte:	MOL000443	Erythraline	Muscarinic acetylcholine receptor M3
Eucommiae corte:	MOL000443	Erythraline	Muscarinic acetylcholine receptor M1
Eucommiae corte:	MOL000443	Erythraline	Sodium channel protein type 5 subunit alpha
Eucommiae corte:	MOL000443	Erythraline	Muscarinic acetylcholine receptor M5
Eucommiae corte:	MOL000443	Erythraline	Prostaglandin G/H synthase 2
Eucommiae corte:	MOL000443	Erythraline	Muscarinic acetylcholine receptor M4
Eucommiae corte:	MOL000443	Erythraline	Retinoic acid receptor RXR-alpha
Eucommiae corte:	MOL000443	Erythraline	Delta-type opioid receptor
Eucommiae corte:	MOL000443	Erythraline	Muscarinic acetylcholine receptor M2
Eucommiae corte:	MOL000443	Erythraline	Alpha-2B adrenergic receptor
Eucommiae corte:	MOL000443	Erythraline	Alpha-1B adrenergic receptor
Eucommiae corte:	MOL000443	Erythraline	Beta-2 adrenergic receptor
Eucommiae corte:	MOL000443	Erythraline	Alpha-1D adrenergic receptor

Eucommiae corte:	MOL000443	Erythraline	Neuronal acetylcholine receptor subunit alpha-2
Eucommiae corte:	MOL000443	Erythraline	Mu-type opioid receptor
Eucommiae corte:	MOL000443	Erythraline	Gamma-aminobutyric acid receptor subunit alpha-1
Eucommiae corte:	MOL000443	Erythraline	Phosphatidylinositol-4,5-bisphosphate 3-kinase catalytic subunit, gamma isoform
Eucommiae corte:	MOL000443	Erythraline	Neuronal acetylcholine receptor protein, alpha-7 chain
Eucommiae corte:	MOL000443	Erythraline	Dopamine D1 receptor
Eucommiae corte:	MOL005922	Acanthoside B	DNA topoisomerase II
Eucommiae corte:	MOL006709	AIDS214634	Prostaglandin G/H synthase 2
Eucommiae corte:	MOL006709	AIDS214634	Nuclear receptor coactivator 2
Eucommiae corte:	MOL006709	AIDS214634	Calmodulin
Eucommiae corte:	MOL006709	AIDS214634	Potassium voltage-gated channel subfamily H member 2
Eucommiae corte:	MOL006709	AIDS214634	Coagulation factor Xa
Eucommiae corte:	MOL006709	AIDS214634	Coagulation factor VII
Eucommiae corte:	MOL007059	3-beta-Hydroxymethyllenetanshiquinone	Dopamine D1 receptor
Eucommiae corte:	MOL007059	3-beta-Hydroxymethyllenetanshiquinone	Thrombin

Eucommiae corte:	MOL007059	3-beta-Hydroxymethyllenetanshiquinone	Muscarinic acetylcholine receptor M1
Eucommiae corte:	MOL007059	3-beta-Hydroxymethyllenetanshiquinone	Prostaglandin G/H synthase 2
Eucommiae corte:	MOL007059	3-beta-Hydroxymethyllenetanshiquinone	Carbonic anhydrase II
Eucommiae corte:	MOL007059	3-beta-Hydroxymethyllenetanshiquinone	Retinoic acid receptor RXR-alpha
Eucommiae corte:	MOL007059	3-beta-Hydroxymethyllenetanshiquinone	Delta-type opioid receptor
Eucommiae corte:	MOL007059	3-beta-Hydroxymethyllenetanshiquinone	Acetylcholinesterase
Eucommiae corte:	MOL007059	3-beta-Hydroxymethyllenetanshiquinone	Alpha-1A adrenergic receptor

Eucommiae corte:	MOL007059	3-beta-Hydroxymethyllenetanshiquinone	Beta-2 adrenergic receptor
Eucommiae corte:	MOL007059	3-beta-Hydroxymethyllenetanshiquinone	Mu-type opioid receptor
Eucommiae corte:	MOL007059	3-beta-Hydroxymethyllenetanshiquinone	Dipeptidyl peptidase IV
Eucommiae corte:	MOL007059	3-beta-Hydroxymethyllenetanshiquinone	Heat shock protein HSP 90
Eucommiae corte:	MOL007059	3-beta-Hydroxymethyllenetanshiquinone	Neuronal acetylcholine receptor protein, alpha-7 chain
Eucommiae corte:	MOL007059	3-beta-Hydroxymethyllenetanshiquinone	Ig gamma-1 chain C region
Eucommiae corte:	MOL007059	3-beta-Hydroxymethyllenetanshiquinone	Trypsin-1

Eucommiae corte:	MOL007059	3-beta-Hydroxymethyllenetanshiquinone	Nuclear receptor coactivator 1
Eucommiae corte:	MOL000073	ent-Epicatechin	Prostaglandin G/H synthase 1
Eucommiae corte:	MOL000073	ent-Epicatechin	Estrogen receptor
Eucommiae corte:	MOL000073	ent-Epicatechin	Prostaglandin G/H synthase 2
Eucommiae corte:	MOL000073	ent-Epicatechin	Heat shock protein HSP 90
Eucommiae corte:	MOL000073	ent-Epicatechin	Beta-lactamase
Eucommiae corte:	MOL000073	ent-Epicatechin	mRNA of PKA Catalytic Subunit C-alpha
Eucommiae corte:	MOL007563	Yangambin	Potassium voltage-gated channel subfamily H member 2
Eucommiae corte:	MOL007563	Yangambin	Sodium channel protein type 5 subunit alpha
Eucommiae corte:	MOL007563	Yangambin	Coagulation factor Xa
Eucommiae corte:	MOL007563	Yangambin	Prostaglandin G/H synthase 2
Eucommiae corte:	MOL007563	Yangambin	DNA topoisomerase II
Eucommiae corte:	MOL007563	Yangambin	Voltage-dependent L-type calcium channel subunit alpha-1S
Eucommiae corte:	MOL007563	Yangambin	Nuclear receptor coactivator 2
Eucommiae corte:	MOL007563	Yangambin	Calmodulin
Eucommiae corte:	MOL007563	Yangambin	Calcium-activated potassium channel subunit alpha 1
Eucommiae corte:	MOL009007	Eucommin A	DNA topoisomerase II
Eucommiae corte:	MOL009009	(+)-medioresinol	Potassium voltage-gated channel subfamily H member 2
Eucommiae corte:	MOL009009	(+)-medioresinol	Sodium channel protein type 5 subunit alpha

Eucommiae corte:	MOL009009	(+)-medioresinol	Coagulation factor Xa
Eucommiae corte:	MOL009009	(+)-medioresinol	Prostaglandin G/H synthase 2
Eucommiae corte:	MOL009009	(+)-medioresinol	Nitric-oxide synthase, endothelial
Eucommiae corte:	MOL009009	(+)-medioresinol	Coagulation factor VII
Eucommiae corte:	MOL009009	(+)-medioresinol	Alpha-1B adrenergic receptor
Eucommiae corte:	MOL009009	(+)-medioresinol	DNA topoisomerase II
Eucommiae corte:	MOL009009	(+)-medioresinol	Heat shock protein HSP 90
Eucommiae corte:	MOL009009	(+)-medioresinol	Nuclear receptor coactivator 2
Eucommiae corte:	MOL009009	(+)-medioresinol	Calmodulin
Eucommiae corte:	MOL009015	(-)-Tabernemontanine	Prostaglandin G/H synthase 1
Eucommiae corte:	MOL009015	(-)-Tabernemontanine	Dopamine D1 receptor
Eucommiae corte:	MOL009015	(-)-Tabernemontanine	Muscarinic acetylcholine receptor M3
Eucommiae corte:	MOL009015	(-)-Tabernemontanine	Potassium voltage-gated channel subfamily H member 2

Eucommiae corte:	MOL009015	(-)-Tabernemontanine	Muscarinic acetylcholine receptor M1
Eucommiae corte:	MOL009015	(-)-Tabernemontanine	Androgen receptor
Eucommiae corte:	MOL009015	(-)-Tabernemontanine	D(1B) dopamine receptor
Eucommiae corte:	MOL009015	(-)-Tabernemontanine	Sodium channel protein type 5 subunit alpha
Eucommiae corte:	MOL009015	(-)-Tabernemontanine	Coagulation factor Xa
Eucommiae corte:	MOL009015	(-)-Tabernemontanine	Muscarinic acetylcholine receptor M5
Eucommiae corte:	MOL009015	(-)-Tabernemontanine	Prostaglandin G/H synthase 2
Eucommiae corte:	MOL009015	(-)-Tabernemontanine	Alpha-2A adrenergic receptor
Eucommiae corte:	MOL009015	(-)-Tabernemontanine	5-hydroxytryptamine receptor 3A
Eucommiae corte:	MOL009015	(-)-Tabernemontanine	5-hydroxytryptamine 2B receptor

Eucommiae cortex	MOL009015	(-)-Tabernemontanine	Muscarinic acetylcholine receptor M4
Eucommiae cortex	MOL009015	(-)-Tabernemontanine	Delta-type opioid receptor
Eucommiae cortex	MOL009015	(-)-Tabernemontanine	Acetylcholinesterase
Eucommiae cortex	MOL009015	(-)-Tabernemontanine	CGMP-inhibited 3',5'-cyclic phosphodiesterase A
Eucommiae cortex	MOL009015	(-)-Tabernemontanine	5-hydroxytryptamine 2A receptor
Eucommiae cortex	MOL009015	(-)-Tabernemontanine	Sodium-dependent noradrenaline transporter
Eucommiae cortex	MOL009015	(-)-Tabernemontanine	Alpha-1A adrenergic receptor
Eucommiae cortex	MOL009015	(-)-Tabernemontanine	5-hydroxytryptamine 2C receptor
Eucommiae cortex	MOL009015	(-)-Tabernemontanine	Muscarinic acetylcholine receptor M2
Eucommiae cortex	MOL009015	(-)-Tabernemontanine	Alpha-2B adrenergic receptor

Eucommiae corte:	MOL009015	(-)-Tabernemontanine	Alpha-1B adrenergic receptor
Eucommiae corte:	MOL009015	(-)-Tabernemontanine	D(3) dopamine receptor
Eucommiae corte:	MOL009015	(-)-Tabernemontanine	5-hydroxytryptamine 7 receptor
Eucommiae corte:	MOL009015	(-)-Tabernemontanine	5-hydroxytryptamine 1D receptor
Eucommiae corte:	MOL009015	(-)-Tabernemontanine	Beta-2 adrenergic receptor
Eucommiae corte:	MOL009015	(-)-Tabernemontanine	Alpha-1D adrenergic receptor
Eucommiae corte:	MOL009015	(-)-Tabernemontanine	Neuronal acetylcholine receptor subunit alpha-2
Eucommiae corte:	MOL009015	(-)-Tabernemontanine	Sodium-dependent serotonin transporter
Eucommiae corte:	MOL009015	(-)-Tabernemontanine	D(2) dopamine receptor
Eucommiae corte:	MOL009015	(-)-Tabernemontanine	Mu-type opioid receptor

Eucommiae corte:	MOL009015	(-)-Tabernemontanine	Gamma-aminobutyric acid receptor subunit alpha-1
Eucommiae corte:	MOL009015	(-)-Tabernemontanine	5-hydroxytryptamine 1B receptor
Eucommiae corte:	MOL009015	(-)-Tabernemontanine	Heat shock protein HSP 90
Eucommiae corte:	MOL009015	(-)-Tabernemontanine	Phosphatidylinositol-4,5-bisphosphate 3-kinase catalytic subunit, gamma isoform
Eucommiae corte:	MOL009015	(-)-Tabernemontanine	Neuronal acetylcholine receptor protein, alpha-7 chain
Eucommiae corte:	MOL009015	(-)-Tabernemontanine	cAMP and cAMP-inhibited cGMP 3',5'-cyclic phosphodiesterase 10A
Eucommiae corte:	MOL009015	(-)-Tabernemontanine	Calmodulin
Eucommiae corte:	MOL009027	Cyclopamine	Androgen receptor
Eucommiae corte:	MOL009027	Cyclopamine	Mineralocorticoid receptor
Eucommiae corte:	MOL009027	Cyclopamine	Glucocorticoid receptor
Eucommiae corte:	MOL009029	Dehydrodiconiferyl alcohol 4,gamma'-di-O-beta-D-glucopyanoside_qt	Prostaglandin G/H synthase 1

Eucommiae corte:	MOL009029	Dehydrodiconiferyl alcohol 4,gamma'-di-O-beta-D-glucopyanoside_qt	Thrombin
Eucommiae corte:	MOL009029	Dehydrodiconiferyl alcohol 4,gamma'-di-O-beta-D-glucopyanoside_qt	Potassium voltage-gated channel subfamily H member 2
Eucommiae corte:	MOL009029	Dehydrodiconiferyl alcohol 4,gamma'-di-O-beta-D-glucopyanoside_qt	Estrogen receptor
Eucommiae corte:	MOL009029	Dehydrodiconiferyl alcohol 4,gamma'-di-O-beta-D-glucopyanoside_qt	Prostaglandin G/H synthase 2
Eucommiae corte:	MOL009029	Dehydrodiconiferyl alcohol 4,gamma'-di-O-beta-D-glucopyanoside_qt	Coagulation factor VII

Eucommiae corte:	MOL009029	Dehydrodiconiferyl alcohol 4,gamma'-di-O-beta-D-glucopyanoside_qt	Acetylcholinesterase
Eucommiae corte:	MOL009029	Dehydrodiconiferyl alcohol 4,gamma'-di-O-beta-D-glucopyanoside_qt	Dipeptidyl peptidase IV
Eucommiae corte:	MOL009029	Dehydrodiconiferyl alcohol 4,gamma'-di-O-beta-D-glucopyanoside_qt	Heat shock protein HSP 90
Eucommiae corte:	MOL009029	Dehydrodiconiferyl alcohol 4,gamma'-di-O-beta-D-glucopyanoside_qt	Trypsin-1
Eucommiae corte:	MOL009029	Dehydrodiconiferyl alcohol 4,gamma'-di-O-beta-D-glucopyanoside_qt	Proto-oncogene serine/threonine-protein kinase Pim-1

Eucommiae corte:	MOL009029	Dehydrodiconiferyl alcohol 4,gamma'-di-O-beta-D-glucopyanoside_qt	Cyclin-A2
Eucommiae corte:	MOL009029	Dehydrodiconiferyl alcohol 4,gamma'-di-O-beta-D-glucopyanoside_qt	Calmodulin
Eucommiae corte:	MOL009031	Cinchonan-9-al, 6'-methoxy-, (9R)-	Prostaglandin G/H synthase 1
Eucommiae corte:	MOL009031	Cinchonan-9-al, 6'-methoxy-, (9R)-	Dopamine D1 receptor
Eucommiae corte:	MOL009031	Cinchonan-9-al, 6'-methoxy-, (9R)-	Muscarinic acetylcholine receptor M3
Eucommiae corte:	MOL009031	Cinchonan-9-al, 6'-methoxy-, (9R)-	Potassium voltage-gated channel subfamily H member 2
Eucommiae corte:	MOL009031	Cinchonan-9-al, 6'-methoxy-, (9R)-	Muscarinic acetylcholine receptor M1
Eucommiae corte:	MOL009031	Cinchonan-9-al, 6'-methoxy-, (9R)-	Beta-1 adrenergic receptor

Eucommiae corte:	MOL009031	Cinchonan-9-al, 6'-methoxy-, (9R)-	Sodium channel protein type 5 subunit alpha
Eucommiae corte:	MOL009031	Cinchonan-9-al, 6'-methoxy-, (9R)-	Coagulation factor Xa
Eucommiae corte:	MOL009031	Cinchonan-9-al, 6'-methoxy-, (9R)-	Muscarinic acetylcholine receptor M5
Eucommiae corte:	MOL009031	Cinchonan-9-al, 6'-methoxy-, (9R)-	Prostaglandin G/H synthase 2
Eucommiae corte:	MOL009031	Cinchonan-9-al, 6'-methoxy-, (9R)-	Alpha-2A adrenergic receptor
Eucommiae corte:	MOL009031	Cinchonan-9-al, 6'-methoxy-, (9R)-	5-hydroxytryptamine receptor 3A
Eucommiae corte:	MOL009031	Cinchonan-9-al, 6'-methoxy-, (9R)-	Alpha-2C adrenergic receptor
Eucommiae corte:	MOL009031	Cinchonan-9-al, 6'-methoxy-, (9R)-	D(4) dopamine receptor
Eucommiae corte:	MOL009031	Cinchonan-9-al, 6'-methoxy-, (9R)-	Muscarinic acetylcholine receptor M4
Eucommiae corte:	MOL009031	Cinchonan-9-al, 6'-methoxy-, (9R)-	Retinoic acid receptor RXR-alpha

Eucommiae corte:	MOL009031	Cinchonan-9-al, 6'-methoxy-, (9R)-	Delta-type opioid receptor
Eucommiae corte:	MOL009031	Cinchonan-9-al, 6'-methoxy-, (9R)-	CGMP-inhibited 3',5'-cyclic phosphodiesterase A
Eucommiae corte:	MOL009031	Cinchonan-9-al, 6'-methoxy-, (9R)-	5-hydroxytryptamine 2A receptor
Eucommiae corte:	MOL009031	Cinchonan-9-al, 6'-methoxy-, (9R)-	Sodium-dependent noradrenaline transporter
Eucommiae corte:	MOL009031	Cinchonan-9-al, 6'-methoxy-, (9R)-	Alpha-1A adrenergic receptor
Eucommiae corte:	MOL009031	Cinchonan-9-al, 6'-methoxy-, (9R)-	5-hydroxytryptamine 2C receptor
Eucommiae corte:	MOL009031	Cinchonan-9-al, 6'-methoxy-, (9R)-	Muscarinic acetylcholine receptor M2
Eucommiae corte:	MOL009031	Cinchonan-9-al, 6'-methoxy-, (9R)-	Alpha-2B adrenergic receptor
Eucommiae corte:	MOL009031	Cinchonan-9-al, 6'-methoxy-, (9R)-	Alpha-1B adrenergic receptor
Eucommiae corte:	MOL009031	Cinchonan-9-al, 6'-methoxy-, (9R)-	D(3) dopamine receptor

Eucommiae corte:	MOL009031	Cinchonan-9-al, 6'-methoxy-, (9R)-	Sodium-dependent dopamine transporter
Eucommiae corte:	MOL009031	Cinchonan-9-al, 6'-methoxy-, (9R)-	5-hydroxytryptamine 7 receptor
Eucommiae corte:	MOL009031	Cinchonan-9-al, 6'-methoxy-, (9R)-	Beta-2 adrenergic receptor
Eucommiae corte:	MOL009031	Cinchonan-9-al, 6'-methoxy-, (9R)-	Alpha-1D adrenergic receptor
Eucommiae corte:	MOL009031	Cinchonan-9-al, 6'-methoxy-, (9R)-	Sodium-dependent serotonin transporter
Eucommiae corte:	MOL009031	Cinchonan-9-al, 6'-methoxy-, (9R)-	D(2) dopamine receptor
Eucommiae corte:	MOL009031	Cinchonan-9-al, 6'-methoxy-, (9R)-	Epidermal growth factor receptor
Eucommiae corte:	MOL009031	Cinchonan-9-al, 6'-methoxy-, (9R)-	Mu-type opioid receptor
Eucommiae corte:	MOL009031	Cinchonan-9-al, 6'-methoxy-, (9R)-	Heat shock protein HSP 90
Eucommiae corte:	MOL009031	Cinchonan-9-al, 6'-methoxy-, (9R)-	Neuronal acetylcholine receptor protein, alpha-7 chain

Eucommiae corte:	MOL009031	Cinchonan-9-al, 6'-methoxy-, (9R)-	mRNA of PKA Catalytic Subunit C-alpha
Eucommiae corte:	MOL009031	Cinchonan-9-al, 6'-methoxy-, (9R)-	Calmodulin
Eucommiae corte:	MOL009042	Helenalin	Gamma-aminobutyric acid receptor subunit alpha-1
Eucommiae corte:	MOL009047	(+)-Eudesmin	Muscarinic acetylcholine receptor M3
Eucommiae corte:	MOL009047	(+)-Eudesmin	Potassium voltage-gated channel subfamily H member 2
Eucommiae corte:	MOL009047	(+)-Eudesmin	Sodium channel protein type 5 subunit alpha
Eucommiae corte:	MOL009047	(+)-Eudesmin	Coagulation factor Xa
Eucommiae corte:	MOL009047	(+)-Eudesmin	Prostaglandin G/H synthase 2
Eucommiae corte:	MOL009047	(+)-Eudesmin	Alpha-1B adrenergic receptor
Eucommiae corte:	MOL009047	(+)-Eudesmin	Beta-2 adrenergic receptor
Eucommiae corte:	MOL009047	(+)-Eudesmin	Heat shock protein HSP 90
Eucommiae corte:	MOL009047	(+)-Eudesmin	Nuclear receptor coactivator 2
Eucommiae corte:	MOL009047	(+)-Eudesmin	Calmodulin
Eucommiae corte:	MOL009053	4-[(2S,3R)-5-[(E)-3-hydroxyprop-1-enyl]-7-methoxy-3-methylol-2,3-dihydrobenzofuran-2-yl]-2-methoxy-phenol	Thrombin

Eucommiae corte:	MOL009053	4-[(2S,3R)-5-[(E)-3-hydroxyprop-1-enyl]-7-methoxy-3-methylol-2,3-dihydrobenzofuran-2-yl]-2-methoxy-phenol	Potassium voltage-gated channel subfamily H member 2
Eucommiae corte:	MOL009053	4-[(2S,3R)-5-[(E)-3-hydroxyprop-1-enyl]-7-methoxy-3-methylol-2,3-dihydrobenzofuran-2-yl]-2-methoxy-phenol	Estrogen receptor
Eucommiae corte:	MOL009053	4-[(2S,3R)-5-[(E)-3-hydroxyprop-1-enyl]-7-methoxy-3-methylol-2,3-dihydrobenzofuran-2-yl]-2-methoxy-phenol	Coagulation factor Xa

Eucommiae corte:	MOL009053	4-[(2S,3R)-5-[(E)-3-hydroxyprop-1-enyl]-7-methoxy-3-methylol-2,3-dihydrobenzofuran-2-yl]-2-methoxy-phenol	Prostaglandin G/H synthase 2
Eucommiae corte:	MOL009053	4-[(2S,3R)-5-[(E)-3-hydroxyprop-1-enyl]-7-methoxy-3-methylol-2,3-dihydrobenzofuran-2-yl]-2-methoxy-phenol	Dipeptidyl peptidase IV
Eucommiae corte:	MOL009053	4-[(2S,3R)-5-[(E)-3-hydroxyprop-1-enyl]-7-methoxy-3-methylol-2,3-dihydrobenzofuran-2-yl]-2-methoxy-phenol	Heat shock protein HSP 90

Eucommiae corte:	MOL009053	4-[(2S,3R)-5-[(E)-3-hydroxyprop-1-enyl]-7-methoxy-3-methylol-2,3-dihydrobenzofuran-2-yl]-2-methoxy-phenol	Trypsin-1
Eucommiae corte:	MOL009053	4-[(2S,3R)-5-[(E)-3-hydroxyprop-1-enyl]-7-methoxy-3-methylol-2,3-dihydrobenzofuran-2-yl]-2-methoxy-phenol	Proto-oncogene serine/threonine-protein kinase Pim-1
Eucommiae corte:	MOL009053	4-[(2S,3R)-5-[(E)-3-hydroxyprop-1-enyl]-7-methoxy-3-methylol-2,3-dihydrobenzofuran-2-yl]-2-methoxy-phenol	Calmodulin

Eucommiae corte:	MOL009053	4-[(2S,3R)-5-[(E)-3-hydroxyprop-1-enyl]-7-methoxy-3-methylol-2,3-dihydrobenzofuran-2-yl]-2-methoxy-phenol	Acetylcholinesterase
Eucommiae corte:	MOL009055	hirsutin_qt	Nitric oxide synthase, inducible
Eucommiae corte:	MOL009055	hirsutin_qt	Prostaglandin G/H synthase 1
Eucommiae corte:	MOL009055	hirsutin_qt	Sodium channel protein type 5 subunit alpha
Eucommiae corte:	MOL009055	hirsutin_qt	Coagulation factor Xa
Eucommiae corte:	MOL009055	hirsutin_qt	Prostaglandin G/H synthase 2
Eucommiae corte:	MOL009055	hirsutin_qt	Carbonic anhydrase II
Eucommiae corte:	MOL009055	hirsutin_qt	Coagulation factor VII
Eucommiae corte:	MOL009055	hirsutin_qt	Vascular endothelial growth factor receptor 2
Eucommiae corte:	MOL009055	hirsutin_qt	Estrogen receptor beta
Eucommiae corte:	MOL009055	hirsutin_qt	Mitogen-activated protein kinase 14
Eucommiae corte:	MOL009055	hirsutin_qt	Glycogen synthase kinase-3 beta
Eucommiae corte:	MOL009055	hirsutin_qt	Heat shock protein HSP 90
Eucommiae corte:	MOL009055	hirsutin_qt	Ig gamma-1 chain C region
Eucommiae corte:	MOL009055	hirsutin_qt	Nuclear receptor coactivator 2
Eucommiae corte:	MOL009055	hirsutin_qt	Calmodulin
Eucommiae corte:	MOL009057	liriodendrin_qt	Nuclear receptor coactivator 2
Eucommiae corte:	MOL000098	quercetin	Prostaglandin G/H synthase 1
Eucommiae corte:	MOL000098	quercetin	Androgen receptor

Eucommiae corte:	MOL000098	quercetin	Peroxisome proliferator activated receptor gamma
Eucommiae corte:	MOL000098	quercetin	Prostaglandin G/H synthase 2
Eucommiae corte:	MOL000098	quercetin	Heat shock protein HSP 90
Eucommiae corte:	MOL000098	quercetin	Phosphatidylinositol-4,5-bisphosphate 3-kinase catalytic subunit, gamma isoform
Eucommiae corte:	MOL000098	quercetin	Nuclear receptor coactivator 2
Eucommiae corte:	MOL000098	quercetin	Dipeptidyl peptidase IV
Eucommiae corte:	MOL000098	quercetin	Aldose reductase
Eucommiae corte:	MOL000098	quercetin	Trypsin-1
Eucommiae corte:	MOL000098	quercetin	DNA topoisomerase II
Eucommiae corte:	MOL000098	quercetin	Thrombin
Eucommiae corte:	MOL000098	quercetin	Potassium voltage-gated channel subfamily H member 2
Eucommiae corte:	MOL000098	quercetin	Sodium channel protein type 5 subunit alpha
Eucommiae corte:	MOL000098	quercetin	Coagulation factor Xa
Eucommiae corte:	MOL000098	quercetin	Beta-2 adrenergic receptor
Eucommiae corte:	MOL000098	quercetin	Stromelysin-1
Eucommiae corte:	MOL000098	quercetin	mRNA of PKA Catalytic Subunit C-alpha
Eucommiae corte:	MOL000098	quercetin	Coagulation factor VII
Eucommiae corte:	MOL000098	quercetin	Nitric-oxide synthase, endothelial
Eucommiae corte:	MOL000098	quercetin	Retinoic acid receptor RXR-alpha
Eucommiae corte:	MOL000098	quercetin	Acetylcholinesterase
Eucommiae corte:	MOL000098	quercetin	Gamma-aminobutyric acid receptor subunit alpha-1
Eucommiae corte:	MOL000098	quercetin	Amine oxidase [flavin-containing] B
Eucommiae corte:	MOL000098	quercetin	Transcription factor p65

Eucommiae corte:	MOL000098	quercetin	Epidermal growth factor receptor
Eucommiae corte:	MOL000098	quercetin	RAC-alpha serine/threonine-protein kinase
Eucommiae corte:	MOL000098	quercetin	Vascular endothelial growth factor A
Eucommiae corte:	MOL000098	quercetin	G1/S-specific cyclin-D1
Eucommiae corte:	MOL000098	quercetin	Apoptosis regulator Bcl-2
Eucommiae corte:	MOL000098	quercetin	Bcl-2-like protein 1
Eucommiae corte:	MOL000098	quercetin	Proto-oncogene c-Fos
Eucommiae corte:	MOL000098	quercetin	Cyclin-dependent kinase inhibitor 1
Eucommiae corte:	MOL000098	quercetin	Eukaryotic translation initiation factor 6
Eucommiae corte:	MOL000098	quercetin	Apoptosis regulator BAX
Eucommiae corte:	MOL000098	quercetin	Caspase-9
Eucommiae corte:	MOL000098	quercetin	Urokinase-type plasminogen activator
Eucommiae corte:	MOL000098	quercetin	72 kDa type IV collagenase
Eucommiae corte:	MOL000098	quercetin	Matrix metalloproteinase-9
Eucommiae corte:	MOL000098	quercetin	Mitogen-activated protein kinase 1
Eucommiae corte:	MOL000098	quercetin	Interleukin-10
Eucommiae corte:	MOL000098	quercetin	Pro-epidermal growth factor
Eucommiae corte:	MOL000098	quercetin	Retinoblastoma-associated protein
Eucommiae corte:	MOL000098	quercetin	Tumor necrosis factor
Eucommiae corte:	MOL000098	quercetin	Transcription factor AP-1
Eucommiae corte:	MOL000098	quercetin	Interleukin-6
Eucommiae corte:	MOL000098	quercetin	Cyclin-dependent kinase inhibitor 2A, isoforms 1/2/3
Eucommiae corte:	MOL000098	quercetin	Activator of 90 kDa heat shock protein ATPase homolog 1
Eucommiae corte:	MOL000098	quercetin	Caspase-3
Eucommiae corte:	MOL000098	quercetin	Cellular tumor antigen p53
Eucommiae corte:	MOL000098	quercetin	ETS domain-containing protein Elk-1

Eucommiae corte:	MOL000098	quercetin	NF-kappa-B inhibitor alpha
Eucommiae corte:	MOL000098	quercetin	NADPH--cytochrome P450 reductase
Eucommiae corte:	MOL000098	quercetin	Ornithine decarboxylase
Eucommiae corte:	MOL000098	quercetin	Xanthine dehydrogenase/oxidase
Eucommiae corte:	MOL000098	quercetin	Caspase-8
Eucommiae corte:	MOL000098	quercetin	DNA topoisomerase 1
Eucommiae corte:	MOL000098	quercetin	RAF proto-oncogene serine/threonine-protein kinase
Eucommiae corte:	MOL000098	quercetin	Superoxide dismutase [Cu-Zn]
Eucommiae corte:	MOL000098	quercetin	Protein kinase C alpha type
Eucommiae corte:	MOL000098	quercetin	Interstitial collagenase
Eucommiae corte:	MOL000098	quercetin	Hypoxia-inducible factor 1-alpha
Eucommiae corte:	MOL000098	quercetin	Signal transducer and activator of transcription 1-alpha/beta
Eucommiae corte:	MOL000098	quercetin	Protein CBFA2T1
Eucommiae corte:	MOL000098	quercetin	Probable E3 ubiquitin-protein ligase HERC5
Eucommiae corte:	MOL000098	quercetin	Cell division control protein 2 homolog
Eucommiae corte:	MOL000098	quercetin	78 kDa glucose-regulated protein
Eucommiae corte:	MOL000098	quercetin	Receptor tyrosine-protein kinase erbB-2
Eucommiae corte:	MOL000098	quercetin	Peroxisome proliferator-activated receptor gamma
Eucommiae corte:	MOL000098	quercetin	Acetyl-CoA carboxylase 1
Eucommiae corte:	MOL000098	quercetin	Heme oxygenase 1
Eucommiae corte:	MOL000098	quercetin	Cytochrome P450 3A4
Eucommiae corte:	MOL000098	quercetin	Cytochrome P450 1A2
Eucommiae corte:	MOL000098	quercetin	Caveolin-1

Eucommiae corte:	MOL000098	quercetin	Myc proto-oncogene protein
Eucommiae corte:	MOL000098	quercetin	Tissue factor
Eucommiae corte:	MOL000098	quercetin	Gap junction alpha-1 protein
Eucommiae corte:	MOL000098	quercetin	Cytochrome P450 1A1
Eucommiae corte:	MOL000098	quercetin	Intercellular adhesion molecule 1
Eucommiae corte:	MOL000098	quercetin	Interleukin-1 beta
Eucommiae corte:	MOL000098	quercetin	C-C motif chemokine 2
Eucommiae corte:	MOL000098	quercetin	E-selectin
Eucommiae corte:	MOL000098	quercetin	Vascular cell adhesion protein 1
Eucommiae corte:	MOL000098	quercetin	Prostaglandin E2 receptor EP3 subtype
Eucommiae corte:	MOL000098	quercetin	Interleukin-8
Eucommiae corte:	MOL000098	quercetin	Protein kinase C beta type
Eucommiae corte:	MOL000098	quercetin	Baculoviral IAP repeat-containing protein 5
Eucommiae corte:	MOL000098	quercetin	Dual oxidase 2
Eucommiae corte:	MOL000098	quercetin	Nitric oxide synthase, endothelial
Eucommiae corte:	MOL000098	quercetin	Heat shock protein beta-1
Eucommiae corte:	MOL000098	quercetin	Transforming growth factor beta-1
Eucommiae corte:	MOL000098	quercetin	Estrogen sulfotransferase
Eucommiae corte:	MOL000098	quercetin	Maltase-glucoamylase, intestinal
Eucommiae corte:	MOL000098	quercetin	Interleukin-2
Eucommiae corte:	MOL000098	quercetin	Nuclear receptor subfamily 1 group I member 2
Eucommiae corte:	MOL000098	quercetin	Cytochrome P450 1B1
Eucommiae corte:	MOL000098	quercetin	G2/mitotic-specific cyclin-B1
Eucommiae corte:	MOL000098	quercetin	Tissue-type plasminogen activator
Eucommiae corte:	MOL000098	quercetin	Thrombomodulin
Eucommiae corte:	MOL000098	quercetin	Plasminogen activator inhibitor 1
Eucommiae corte:	MOL000098	quercetin	Collagen alpha-1(I) chain

Eucommiae corte:	MOL000098	quercetin	Interferon gamma
Eucommiae corte:	MOL000098	quercetin	Arachidonate 5-lipoxygenase
Eucommiae corte:	MOL000098	quercetin	Phosphatidylinositol-3,4,5-trisphosphate 3-phosphatase and dual-specificity protein phosphatase PTEN
Eucommiae corte:	MOL000098	quercetin	Interleukin-1 alpha
Eucommiae corte:	MOL000098	quercetin	Myeloperoxidase
Eucommiae corte:	MOL000098	quercetin	DNA topoisomerase 2-alpha
Eucommiae corte:	MOL000098	quercetin	Neutrophil cytosol factor 1
Eucommiae corte:	MOL000098	quercetin	ATP-binding cassette sub-family G member 2
Eucommiae corte:	MOL000098	quercetin	Hyaluronan synthase 2
Eucommiae corte:	MOL000098	quercetin	Glutathione S-transferase P
Eucommiae corte:	MOL000098	quercetin	Nuclear factor erythroid 2-related factor 2
Eucommiae corte:	MOL000098	quercetin	NAD(P)H dehydrogenase [quinone] 1
Eucommiae corte:	MOL000098	quercetin	Poly [ADP-ribose] polymerase 1
Eucommiae corte:	MOL000098	quercetin	Aryl hydrocarbon receptor
Eucommiae corte:	MOL000098	quercetin	26S proteasome non-ATPase regulatory subunit 3
Eucommiae corte:	MOL000098	quercetin	Solute carrier family 2, facilitated glucose transporter member 4
Eucommiae corte:	MOL000098	quercetin	Collagen alpha-1(III) chain
Eucommiae corte:	MOL000098	quercetin	DNA gyrase subunit B
Eucommiae corte:	MOL000098	quercetin	C-X-C motif chemokine 11
Eucommiae corte:	MOL000098	quercetin	C-X-C motif chemokine 2
Eucommiae corte:	MOL000098	quercetin	DDB1- and CUL4-associated factor 5

Eucommiae corte:	MOL000098	quercetin	Nuclear receptor subfamily 1 group I member 3
Eucommiae corte:	MOL000098	quercetin	Serine/threonine-protein kinase Chk2
Eucommiae corte:	MOL000098	quercetin	Insulin receptor
Eucommiae corte:	MOL000098	quercetin	Claudin-4
Eucommiae corte:	MOL000098	quercetin	Peroxisome proliferator-activated receptor alpha
Eucommiae corte:	MOL000098	quercetin	Peroxisome proliferator-activated receptor delta
Eucommiae corte:	MOL000098	quercetin	Heat shock factor protein 1
Eucommiae corte:	MOL000098	quercetin	C-reactive protein
Eucommiae corte:	MOL000098	quercetin	C-X-C motif chemokine 10
Eucommiae corte:	MOL000098	quercetin	Inhibitor of nuclear factor kappa-B kinase subunit alpha
Eucommiae corte:	MOL000098	quercetin	Osteopontin
Eucommiae corte:	MOL000098	quercetin	Runt-related transcription factor 2
Eucommiae corte:	MOL000098	quercetin	Ras association domain-containing protein 1
Eucommiae corte:	MOL000098	quercetin	Transcription factor E2F1
Eucommiae corte:	MOL000098	quercetin	Transcription factor E2F2
Eucommiae corte:	MOL000098	quercetin	Prostatic acid phosphatase
Eucommiae corte:	MOL000098	quercetin	Cathepsin D
Eucommiae corte:	MOL000098	quercetin	Insulin-like growth factor-binding protein 3
Eucommiae corte:	MOL000098	quercetin	Insulin-like growth factor II
Eucommiae corte:	MOL000098	quercetin	CD40 ligand
Eucommiae corte:	MOL000098	quercetin	Interferon regulatory factor 1
Eucommiae corte:	MOL000098	quercetin	Receptor tyrosine-protein kinase erbB-3
Eucommiae corte:	MOL000098	quercetin	Serum paraoxonase/arylesterase 1

Eucommiae corte:	MOL000098	quercetin	Type I iodothyronine deiodinase
Eucommiae corte:	MOL000098	quercetin	Procollagen C-endopeptidase enhancer 1
Eucommiae corte:	MOL000098	quercetin	Puromycin-sensitive aminopeptidase
Eucommiae corte:	MOL000098	quercetin	Hexokinase-2
Eucommiae corte:	MOL000098	quercetin	Homeobox protein Nkx-3.1
Eucommiae corte:	MOL000098	quercetin	Ras GTPase-activating protein 1
Eucommiae corte:	MOL000098	quercetin	Peroxidase C1A
Eucommiae corte:	MOL000098	quercetin	Glutathione S-transferase Mu 1
Eucommiae corte:	MOL000098	quercetin	Glutathione S-transferase Mu 2
Eucommiae corte:	MOL002773	beta-carotene	RAC-alpha serine/threonine-protein kinase
Eucommiae corte:	MOL002773	beta-carotene	Vascular endothelial growth factor A
Eucommiae corte:	MOL002773	beta-carotene	Apoptosis regulator Bcl-2
Eucommiae corte:	MOL002773	beta-carotene	Caspase-9
Eucommiae corte:	MOL002773	beta-carotene	72 kDa type IV collagenase
Eucommiae corte:	MOL002773	beta-carotene	Transcription factor AP-1
Eucommiae corte:	MOL002773	beta-carotene	Caspase-3
Eucommiae corte:	MOL002773	beta-carotene	Prostaglandin G/H synthase 2
Eucommiae corte:	MOL002773	beta-carotene	Caspase-8
Eucommiae corte:	MOL002773	beta-carotene	Interstitial collagenase
Eucommiae corte:	MOL002773	beta-carotene	Heme oxygenase 1
Eucommiae corte:	MOL002773	beta-carotene	Cytochrome P450 3A4
Eucommiae corte:	MOL002773	beta-carotene	Cytochrome P450 2B1
Eucommiae corte:	MOL002773	beta-carotene	Cytochrome P450 1A2
Eucommiae corte:	MOL002773	beta-carotene	Serum albumin
Eucommiae corte:	MOL002773	beta-carotene	Caveolin-1
Eucommiae corte:	MOL002773	beta-carotene	Catenin beta-1
Eucommiae corte:	MOL002773	beta-carotene	Myc proto-oncogene protein
Eucommiae corte:	MOL002773	beta-carotene	Caspase-7

Eucommiae corte:	MOL002773	beta-carotene	Tissue factor
Eucommiae corte:	MOL002773	beta-carotene	Gap junction alpha-1 protein
Eucommiae corte:	MOL002773	beta-carotene	Stromelysin-2
Eucommiae corte:	MOL008240	(E)-3-[4-[(1R,2R)-2-hydroxy-2-(4-hydroxy-3-methoxy-phenyl)-1-methylol-ethoxy]-3-methoxy-phenyl]acrolein	Nitric oxide synthase, inducible
Eucommiae corte:	MOL008240	(E)-3-[4-[(1R,2R)-2-hydroxy-2-(4-hydroxy-3-methoxy-phenyl)-1-methylol-ethoxy]-3-methoxy-phenyl]acrolein	Thrombin

Eucommiae corte:	MOL008240	(E)-3-[4-[(1R,2R)-2-hydroxy-2-(4-hydroxy-3-methoxy-phenyl)-1-methylol-ethoxy]-3-methoxy-phenyl]acrolein	Estrogen receptor
Eucommiae corte:	MOL008240	(E)-3-[4-[(1R,2R)-2-hydroxy-2-(4-hydroxy-3-methoxy-phenyl)-1-methylol-ethoxy]-3-methoxy-phenyl]acrolein	Peroxisome proliferator activated receptor gamma
Eucommiae corte:	MOL008240	(E)-3-[4-[(1R,2R)-2-hydroxy-2-(4-hydroxy-3-methoxy-phenyl)-1-methylol-ethoxy]-3-methoxy-phenyl]acrolein	Coagulation factor Xa

Eucommiae corte:	MOL008240	(E)-3-[4-[(1R,2R)-2-hydroxy-2-(4-hydroxy-3-methoxy-phenyl)-1-methylol-ethoxy]-3-methoxy-phenyl]acrolein	Prostaglandin G/H synthase 2
Eucommiae corte:	MOL008240	(E)-3-[4-[(1R,2R)-2-hydroxy-2-(4-hydroxy-3-methoxy-phenyl)-1-methylol-ethoxy]-3-methoxy-phenyl]acrolein	Coagulation factor VII
Eucommiae corte:	MOL008240	(E)-3-[4-[(1R,2R)-2-hydroxy-2-(4-hydroxy-3-methoxy-phenyl)-1-methylol-ethoxy]-3-methoxy-phenyl]acrolein	Dipeptidyl peptidase IV

Eucommiae corte:	MOL008240	(E)-3-[4-[(1R,2R)-2-hydroxy-2-(4-hydroxy-3-methoxy-phenyl)-1-methylol-ethoxy]-3-methoxy-phenyl]acrolein	Mitogen-activated protein kinase 14
Eucommiae corte:	MOL008240	(E)-3-[4-[(1R,2R)-2-hydroxy-2-(4-hydroxy-3-methoxy-phenyl)-1-methylol-ethoxy]-3-methoxy-phenyl]acrolein	Glycogen synthase kinase-3 beta
Eucommiae corte:	MOL008240	(E)-3-[4-[(1R,2R)-2-hydroxy-2-(4-hydroxy-3-methoxy-phenyl)-1-methylol-ethoxy]-3-methoxy-phenyl]acrolein	Heat shock protein HSP 90

Eucommiae corte:	MOL008240	(E)-3-[4-[(1R,2R)-2-hydroxy-2-(4-hydroxy-3-methoxy-phenyl)-1-methylol-ethoxy]-3-methoxy-phenyl]acrolein	Cell division protein kinase 2
Eucommiae corte:	MOL008240	(E)-3-[4-[(1R,2R)-2-hydroxy-2-(4-hydroxy-3-methoxy-phenyl)-1-methylol-ethoxy]-3-methoxy-phenyl]acrolein	Trypsin-1
Eucommiae corte:	MOL008240	(E)-3-[4-[(1R,2R)-2-hydroxy-2-(4-hydroxy-3-methoxy-phenyl)-1-methylol-ethoxy]-3-methoxy-phenyl]acrolein	Proto-oncogene serine/threonine-protein kinase Pim-1

Eucommiae corte	MOL008240	(E)-3-[4-[(1R,2R)-2-hydroxy-2-(4-hydroxy-3-methoxy-phenyl)-1-methylol-ethoxy]-3-methoxy-phenyl]acrolein	Cyclin-A2
Eucommiae corte	MOL008240	(E)-3-[4-[(1R,2R)-2-hydroxy-2-(4-hydroxy-3-methoxy-phenyl)-1-methylol-ethoxy]-3-methoxy-phenyl]acrolein	Calmodulin
Eucommiae corte	MOL011604	Syringetin	Nitric oxide synthase, inducible
Eucommiae corte	MOL011604	Syringetin	Estrogen receptor
Eucommiae corte	MOL011604	Syringetin	Androgen receptor
Eucommiae corte	MOL011604	Syringetin	Sodium channel protein type 5 subunit alpha
Eucommiae corte	MOL011604	Syringetin	Peroxisome proliferator activated receptor gamma
Eucommiae corte	MOL011604	Syringetin	Prostaglandin G/H synthase 2
Eucommiae corte	MOL011604	Syringetin	Coagulation factor VII
Eucommiae corte	MOL011604	Syringetin	DNA topoisomerase II

Eucommiae corte:	MOL011604	Syringetin	Estrogen receptor beta
Eucommiae corte:	MOL011604	Syringetin	Dipeptidyl peptidase IV
Eucommiae corte:	MOL011604	Syringetin	Mitogen-activated protein kinase 14
Eucommiae corte:	MOL011604	Syringetin	Glycogen synthase kinase-3 beta
Eucommiae corte:	MOL011604	Syringetin	Heat shock protein HSP 90
Eucommiae corte:	MOL011604	Syringetin	Cell division protein kinase 2
Eucommiae corte:	MOL011604	Syringetin	Trypsin-1
Eucommiae corte:	MOL011604	Syringetin	Proto-oncogene serine/threonine-protein kinase Pim-1
Eucommiae corte:	MOL011604	Syringetin	Cyclin-A2
Eucommiae corte:	MOL011604	Syringetin	Calmodulin
Eucommiae corte:	MOL011604	Syringetin	Nuclear receptor coactivator 2
Angelicae Sinens	MOL000358	beta-sitosterol	Progesterone receptor
Angelicae Sinens	MOL000358	beta-sitosterol	Nuclear receptor coactivator 2
Angelicae Sinens	MOL000358	beta-sitosterol	Prostaglandin G/H synthase 1
Angelicae Sinens	MOL000358	beta-sitosterol	Prostaglandin G/H synthase 2
Angelicae Sinens	MOL000358	beta-sitosterol	Heat shock protein HSP 90
Angelicae Sinens	MOL000358	beta-sitosterol	Phosphatidylinositol-4,5-bisphosphate 3-kinase catalytic subunit, gamma isoform
Angelicae Sinens	MOL000358	beta-sitosterol	Potassium voltage-gated channel subfamily H member 2
Angelicae Sinens	MOL000358	beta-sitosterol	mRNA of PKA Catalytic Subunit C-alpha
Angelicae Sinens	MOL000358	beta-sitosterol	Dopamine D1 receptor
Angelicae Sinens	MOL000358	beta-sitosterol	Muscarinic acetylcholine receptor M3
Angelicae Sinens	MOL000358	beta-sitosterol	Muscarinic acetylcholine receptor M1
Angelicae Sinens	MOL000358	beta-sitosterol	Sodium channel protein type 5 subunit alpha

Angelicae Sinens	MOL000358	beta-sitosterol	Gamma-aminobutyric-acid receptor alpha-2 subunit
Angelicae Sinens	MOL000358	beta-sitosterol	Muscarinic acetylcholine receptor M4
Angelicae Sinens	MOL000358	beta-sitosterol	CGMP-inhibited 3',5'-cyclic phosphodiesterase A
Angelicae Sinens	MOL000358	beta-sitosterol	5-hydroxytryptamine 2A receptor
Angelicae Sinens	MOL000358	beta-sitosterol	Gamma-aminobutyric-acid receptor alpha-5 subunit
Angelicae Sinens	MOL000358	beta-sitosterol	Alpha-1A adrenergic receptor
Angelicae Sinens	MOL000358	beta-sitosterol	Gamma-aminobutyric-acid receptor alpha-3 subunit
Angelicae Sinens	MOL000358	beta-sitosterol	Muscarinic acetylcholine receptor M2
Angelicae Sinens	MOL000358	beta-sitosterol	Alpha-1B adrenergic receptor
Angelicae Sinens	MOL000358	beta-sitosterol	Beta-2 adrenergic receptor
Angelicae Sinens	MOL000358	beta-sitosterol	Neuronal acetylcholine receptor subunit alpha-2
Angelicae Sinens	MOL000358	beta-sitosterol	Sodium-dependent serotonin transporter
Angelicae Sinens	MOL000358	beta-sitosterol	Mu-type opioid receptor
Angelicae Sinens	MOL000358	beta-sitosterol	Gamma-aminobutyric acid receptor subunit alpha-1
Angelicae Sinens	MOL000358	beta-sitosterol	Neuronal acetylcholine receptor protein, alpha-7 chain
Angelicae Sinens	MOL000358	beta-sitosterol	Cytochrome P450-cam
Angelicae Sinens	MOL000358	beta-sitosterol	Apoptosis regulator Bcl-2
Angelicae Sinens	MOL000358	beta-sitosterol	Apoptosis regulator BAX
Angelicae Sinens	MOL000358	beta-sitosterol	Caspase-9
Angelicae Sinens	MOL000358	beta-sitosterol	Transcription factor AP-1
Angelicae Sinens	MOL000358	beta-sitosterol	Caspase-3
Angelicae Sinens	MOL000358	beta-sitosterol	Caspase-8

Angelicae Sinens	MOL000358	beta-sitosterol	Protein kinase C alpha type
Angelicae Sinens	MOL000358	beta-sitosterol	Transforming growth factor beta-1
Angelicae Sinens	MOL000358	beta-sitosterol	Serum paraoxonase/arylesterase 1
Angelicae Sinens	MOL000358	beta-sitosterol	Microtubule-associated protein 2
Angelicae Sinens	MOL000449	Stigmasterol	Progesterone receptor
Angelicae Sinens	MOL000449	Stigmasterol	Mineralocorticoid receptor
Angelicae Sinens	MOL000449	Stigmasterol	Nuclear receptor coactivator 2
Angelicae Sinens	MOL000449	Stigmasterol	Alcohol dehydrogenase 1C
Angelicae Sinens	MOL000449	Stigmasterol	Ig gamma-1 chain C region
Angelicae Sinens	MOL000449	Stigmasterol	Retinoic acid receptor RXR-alpha
Angelicae Sinens	MOL000449	Stigmasterol	Nuclear receptor coactivator 1
Angelicae Sinens	MOL000449	Stigmasterol	Prostaglandin G/H synthase 1
Angelicae Sinens	MOL000449	Stigmasterol	Prostaglandin G/H synthase 2
Angelicae Sinens	MOL000449	Stigmasterol	Alpha-2A adrenergic receptor
Angelicae Sinens	MOL000449	Stigmasterol	Sodium-dependent noradrenaline transporter
Angelicae Sinens	MOL000449	Stigmasterol	Sodium-dependent dopamine transporter
Angelicae Sinens	MOL000449	Stigmasterol	Beta-2 adrenergic receptor
Angelicae Sinens	MOL000449	Stigmasterol	Aldose reductase
Angelicae Sinens	MOL000449	Stigmasterol	Urokinase-type plasminogen activator
Angelicae Sinens	MOL000449	Stigmasterol	Leukotriene A-4 hydrolase
Angelicae Sinens	MOL000449	Stigmasterol	Amine oxidase [flavin-containing] B
Angelicae Sinens	MOL000449	Stigmasterol	Amine oxidase [flavin-containing] A
Angelicae Sinens	MOL000449	Stigmasterol	mRNA of PKA Catalytic Subunit C-alpha
Angelicae Sinens	MOL000449	Stigmasterol	Chymotrypsinogen B
Angelicae Sinens	MOL000449	Stigmasterol	Muscarinic acetylcholine receptor M3
Angelicae Sinens	MOL000449	Stigmasterol	Muscarinic acetylcholine receptor M1
Angelicae Sinens	MOL000449	Stigmasterol	Beta-1 adrenergic receptor

Angelicae Sinens	MOL000449	Stigmasterol	Sodium channel protein type 5 subunit alpha
Angelicae Sinens	MOL000449	Stigmasterol	5-hydroxytryptamine 2A receptor
Angelicae Sinens	MOL000449	Stigmasterol	Alpha-1A adrenergic receptor
Angelicae Sinens	MOL000449	Stigmasterol	Gamma-aminobutyric-acid receptor alpha-3 subunit
Angelicae Sinens	MOL000449	Stigmasterol	Muscarinic acetylcholine receptor M2
Angelicae Sinens	MOL000449	Stigmasterol	Alpha-1B adrenergic receptor
Angelicae Sinens	MOL000449	Stigmasterol	Gamma-aminobutyric acid receptor subunit alpha-1
Angelicae Sinens	MOL000449	Stigmasterol	Neuronal acetylcholine receptor protein, alpha-7 chain
Semen Cuseutae	MOL001558	sesamin	Coagulation factor Xa
Semen Cuseutae	MOL001558	sesamin	Prostaglandin G/H synthase 2
Semen Cuseutae	MOL001558	sesamin	Sodium channel protein type 5 subunit alpha
Semen Cuseutae	MOL001558	sesamin	G1/S-specific cyclin-D1
Semen Cuseutae	MOL001558	sesamin	Interleukin-10
Semen Cuseutae	MOL001558	sesamin	Fatty acid synthase
Semen Cuseutae	MOL001558	sesamin	Acetyl-CoA carboxylase 1
Semen Cuseutae	MOL001558	sesamin	Glucose-6-phosphate 1-dehydrogenase
Semen Cuseutae	MOL001558	sesamin	Nitric oxide synthase, endothelial
Semen Cuseutae	MOL001558	sesamin	Endothelin-converting enzyme 1
Semen Cuseutae	MOL001558	sesamin	Medium-chain specific acyl-CoA dehydrogenase, mitochondrial
Semen Cuseutae	MOL001558	sesamin	Cytochrome P450 2B6
Semen Cuseutae	MOL001558	sesamin	UDP-glucuronosyltransferase 1-1
Semen Cuseutae	MOL001558	sesamin	Sterol regulatory element-binding protein 1

Semen Cuseutae	MOL001558	sesamin	NADPH oxidase 3
Semen Cuseutae	MOL001558	sesamin	NADPH oxidase 1
Semen Cuseutae	MOL001558	sesamin	Peroxisomal acyl-coenzyme A oxidase 1
Semen Cuseutae	MOL001558	sesamin	ATP-citrate synthase
Semen Cuseutae	MOL001558	sesamin	Peroxisomal bifunctional enzyme
Semen Cuseutae	MOL001558	sesamin	Methylglutaconyl-CoA hydratase, mitochondrial
Semen Cuseutae	MOL001558	sesamin	Trifunctional enzyme subunit beta, mitochondrial
Semen Cuseutae	MOL001558	sesamin	2,4-dienoyl-CoA reductase, mitochondrial
Semen Cuseutae	MOL001558	sesamin	3,2-trans-enoyl-CoA isomerase, mitochondrial
Semen Cuseutae	MOL000184	NSC63551	Progesterone receptor
Semen Cuseutae	MOL000354	isorhamnetin	Nitric oxide synthase, inducible
Semen Cuseutae	MOL000354	isorhamnetin	Prostaglandin G/H synthase 1
Semen Cuseutae	MOL000354	isorhamnetin	Estrogen receptor
Semen Cuseutae	MOL000354	isorhamnetin	Androgen receptor
Semen Cuseutae	MOL000354	isorhamnetin	Peroxisome proliferator activated receptor gamma
Semen Cuseutae	MOL000354	isorhamnetin	Prostaglandin G/H synthase 2
Semen Cuseutae	MOL000354	isorhamnetin	mRNA of Protein-tyrosine phosphatase, non-receptor type 1
Semen Cuseutae	MOL000354	isorhamnetin	Estrogen receptor beta
Semen Cuseutae	MOL000354	isorhamnetin	Dipeptidyl peptidase IV
Semen Cuseutae	MOL000354	isorhamnetin	Mitogen-activated protein kinase 14
Semen Cuseutae	MOL000354	isorhamnetin	Glycogen synthase kinase-3 beta
Semen Cuseutae	MOL000354	isorhamnetin	Heat shock protein HSP 90
Semen Cuseutae	MOL000354	isorhamnetin	Cell division protein kinase 2

Semen Cuseutae	MOL000354	isorhamnetin	Phosphatidylinositol-4,5-bisphosphate 3-kinase catalytic subunit, gamma isoform
Semen Cuseutae	MOL000354	isorhamnetin	mRNA of PKA Catalytic Subunit C-alpha
Semen Cuseutae	MOL000354	isorhamnetin	Trypsin-1
Semen Cuseutae	MOL000354	isorhamnetin	Proto-oncogene serine/threonine-protein kinase Pim-1
Semen Cuseutae	MOL000354	isorhamnetin	Cyclin-A2
Semen Cuseutae	MOL000354	isorhamnetin	Nuclear receptor coactivator 2
Semen Cuseutae	MOL000354	isorhamnetin	Calmodulin
Semen Cuseutae	MOL000354	isorhamnetin	Glycogen phosphorylase, muscle form
Semen Cuseutae	MOL000354	isorhamnetin	Peroxisome proliferator activated receptor delta
Semen Cuseutae	MOL000354	isorhamnetin	Serine/threonine-protein kinase Chk1
Semen Cuseutae	MOL000354	isorhamnetin	Aldose reductase
Semen Cuseutae	MOL000354	isorhamnetin	Nuclear receptor coactivator 1
Semen Cuseutae	MOL000354	isorhamnetin	Coagulation factor VII
Semen Cuseutae	MOL000354	isorhamnetin	Thrombin
Semen Cuseutae	MOL000354	isorhamnetin	Nitric-oxide synthase, endothelial
Semen Cuseutae	MOL000354	isorhamnetin	Acetylcholinesterase
Semen Cuseutae	MOL000354	isorhamnetin	Gamma-aminobutyric acid receptor subunit alpha-1
Semen Cuseutae	MOL000354	isorhamnetin	Amine oxidase [flavin-containing] B
Semen Cuseutae	MOL000354	isorhamnetin	Glutamate receptor 2
Semen Cuseutae	MOL000354	isorhamnetin	Cytochrome P450-cam
Semen Cuseutae	MOL000354	isorhamnetin	Transcription factor p65
Semen Cuseutae	MOL000354	isorhamnetin	Xanthine dehydrogenase/oxidase
Semen Cuseutae	MOL000354	isorhamnetin	Neutrophil cytosol factor 1

Semen Cuseutae	MOL000354	isorhamnetin	Oxidized low-density lipoprotein receptor 1
Semen Cuseutae	MOL000358	beta-sitosterol	Progesterone receptor
Semen Cuseutae	MOL000358	beta-sitosterol	Nuclear receptor coactivator 2
Semen Cuseutae	MOL000358	beta-sitosterol	Prostaglandin G/H synthase 1
Semen Cuseutae	MOL000358	beta-sitosterol	Prostaglandin G/H synthase 2
Semen Cuseutae	MOL000358	beta-sitosterol	Heat shock protein HSP 90
Semen Cuseutae	MOL000358	beta-sitosterol	Phosphatidylinositol-4,5-bisphosphate 3-kinase catalytic subunit, gamma isoform
Semen Cuseutae	MOL000358	beta-sitosterol	Potassium voltage-gated channel subfamily H member 2
Semen Cuseutae	MOL000358	beta-sitosterol	mRNA of PKA Catalytic Subunit C-alpha
Semen Cuseutae	MOL000358	beta-sitosterol	Dopamine D1 receptor
Semen Cuseutae	MOL000358	beta-sitosterol	Muscarinic acetylcholine receptor M3
Semen Cuseutae	MOL000358	beta-sitosterol	Muscarinic acetylcholine receptor M1
Semen Cuseutae	MOL000358	beta-sitosterol	Sodium channel protein type 5 subunit alpha
Semen Cuseutae	MOL000358	beta-sitosterol	Gamma-aminobutyric-acid receptor alpha-2 subunit
Semen Cuseutae	MOL000358	beta-sitosterol	Muscarinic acetylcholine receptor M4
Semen Cuseutae	MOL000358	beta-sitosterol	CGMP-inhibited 3',5'-cyclic phosphodiesterase A
Semen Cuseutae	MOL000358	beta-sitosterol	5-hydroxytryptamine 2A receptor
Semen Cuseutae	MOL000358	beta-sitosterol	Gamma-aminobutyric-acid receptor alpha-5 subunit
Semen Cuseutae	MOL000358	beta-sitosterol	Alpha-1A adrenergic receptor
Semen Cuseutae	MOL000358	beta-sitosterol	Gamma-aminobutyric-acid receptor alpha-3 subunit

Semen Cuseutae	MOL000358	beta-sitosterol	Muscarinic acetylcholine receptor M2
Semen Cuseutae	MOL000358	beta-sitosterol	Alpha-1B adrenergic receptor
Semen Cuseutae	MOL000358	beta-sitosterol	Beta-2 adrenergic receptor
Semen Cuseutae	MOL000358	beta-sitosterol	Neuronal acetylcholine receptor subunit alpha-2
Semen Cuseutae	MOL000358	beta-sitosterol	Sodium-dependent serotonin transporter
Semen Cuseutae	MOL000358	beta-sitosterol	Mu-type opioid receptor
Semen Cuseutae	MOL000358	beta-sitosterol	Gamma-aminobutyric acid receptor subunit alpha-1
Semen Cuseutae	MOL000358	beta-sitosterol	Neuronal acetylcholine receptor protein, alpha-7 chain
Semen Cuseutae	MOL000358	beta-sitosterol	Cytochrome P450-cam
Semen Cuseutae	MOL000358	beta-sitosterol	Apoptosis regulator Bcl-2
Semen Cuseutae	MOL000358	beta-sitosterol	Apoptosis regulator BAX
Semen Cuseutae	MOL000358	beta-sitosterol	Caspase-9
Semen Cuseutae	MOL000358	beta-sitosterol	Transcription factor AP-1
Semen Cuseutae	MOL000358	beta-sitosterol	Caspase-3
Semen Cuseutae	MOL000358	beta-sitosterol	Caspase-8
Semen Cuseutae	MOL000358	beta-sitosterol	Protein kinase C alpha type
Semen Cuseutae	MOL000358	beta-sitosterol	Transforming growth factor beta-1
Semen Cuseutae	MOL000358	beta-sitosterol	Serum paraoxonase/arylesterase 1
Semen Cuseutae	MOL000358	beta-sitosterol	Microtubule-associated protein 2
Semen Cuseutae	MOL000422	kaempferol	Nitric oxide synthase, inducible
Semen Cuseutae	MOL000422	kaempferol	Prostaglandin G/H synthase 1
Semen Cuseutae	MOL000422	kaempferol	Androgen receptor
Semen Cuseutae	MOL000422	kaempferol	Peroxisome proliferator activated receptor gamma
Semen Cuseutae	MOL000422	kaempferol	Prostaglandin G/H synthase 2
Semen Cuseutae	MOL000422	kaempferol	Heat shock protein HSP 90

Semen Cuseutae	MOL000422	kaempferol	Phosphatidylinositol-4,5-bisphosphate 3-kinase catalytic subunit, gamma isoform
Semen Cuseutae	MOL000422	kaempferol	mRNA of PKA Catalytic Subunit C-alpha
Semen Cuseutae	MOL000422	kaempferol	Nuclear receptor coactivator 2
Semen Cuseutae	MOL000422	kaempferol	Dipeptidyl peptidase IV
Semen Cuseutae	MOL000422	kaempferol	Trypsin-1
Semen Cuseutae	MOL000422	kaempferol	Progesterone receptor
Semen Cuseutae	MOL000422	kaempferol	Thrombin
Semen Cuseutae	MOL000422	kaempferol	Muscarinic acetylcholine receptor M1
Semen Cuseutae	MOL000422	kaempferol	Nitric-oxide synthase, endothelial
Semen Cuseutae	MOL000422	kaempferol	Gamma-aminobutyric-acid receptor alpha-2 subunit
Semen Cuseutae	MOL000422	kaempferol	Acetylcholinesterase
Semen Cuseutae	MOL000422	kaempferol	Sodium-dependent noradrenaline transporter
Semen Cuseutae	MOL000422	kaempferol	Muscarinic acetylcholine receptor M2
Semen Cuseutae	MOL000422	kaempferol	Alpha-1B adrenergic receptor
Semen Cuseutae	MOL000422	kaempferol	Gamma-aminobutyric acid receptor subunit alpha-1
Semen Cuseutae	MOL000422	kaempferol	DNA topoisomerase II
Semen Cuseutae	MOL000422	kaempferol	Coagulation factor VII
Semen Cuseutae	MOL000422	kaempferol	Calmodulin
Semen Cuseutae	MOL000422	kaempferol	Transcription factor p65
Semen Cuseutae	MOL000422	kaempferol	Inhibitor of nuclear factor kappa-B kinase subunit beta
Semen Cuseutae	MOL000422	kaempferol	RAC-alpha serine/threonine-protein kinase
Semen Cuseutae	MOL000422	kaempferol	Apoptosis regulator Bcl-2

Semen Cuseutae	MOL000422	kaempferol	Apoptosis regulator BAX
Semen Cuseutae	MOL000422	kaempferol	Tumor necrosis factor
Semen Cuseutae	MOL000422	kaempferol	Transcription factor AP-1
Semen Cuseutae	MOL000422	kaempferol	Activator of 90 kDa heat shock protein ATPase homolog 1
Semen Cuseutae	MOL000422	kaempferol	Caspase-3
Semen Cuseutae	MOL000422	kaempferol	Mitogen-activated protein kinase 8
Semen Cuseutae	MOL000422	kaempferol	Xanthine dehydrogenase/oxidase
Semen Cuseutae	MOL000422	kaempferol	Interstitial collagenase
Semen Cuseutae	MOL000422	kaempferol	Signal transducer and activator of transcription 1-alpha/beta
Semen Cuseutae	MOL000422	kaempferol	Cell division control protein 2 homolog
Semen Cuseutae	MOL000422	kaempferol	Peroxisome proliferator-activated receptor gamma
Semen Cuseutae	MOL000422	kaempferol	Heme oxygenase 1
Semen Cuseutae	MOL000422	kaempferol	Cytochrome P450 3A4
Semen Cuseutae	MOL000422	kaempferol	Cytochrome P450 1A2
Semen Cuseutae	MOL000422	kaempferol	Cytochrome P450 1A1
Semen Cuseutae	MOL000422	kaempferol	Intercellular adhesion molecule 1
Semen Cuseutae	MOL000422	kaempferol	E-selectin
Semen Cuseutae	MOL000422	kaempferol	Vascular cell adhesion protein 1
Semen Cuseutae	MOL000422	kaempferol	Nuclear receptor subfamily 1 group I member 2
Semen Cuseutae	MOL000422	kaempferol	Cytochrome P450 1B1
Semen Cuseutae	MOL000422	kaempferol	Arachidonate 5-lipoxygenase
Semen Cuseutae	MOL000422	kaempferol	Hyaluronan synthase 2
Semen Cuseutae	MOL000422	kaempferol	Glutathione S-transferase P
Semen Cuseutae	MOL000422	kaempferol	Aryl hydrocarbon receptor

Semen Cuseutae	MOL000422	kaempferol	26S proteasome non-ATPase regulatory subunit 3
Semen Cuseutae	MOL000422	kaempferol	Solute carrier family 2, facilitated glucose transporter member 4
Semen Cuseutae	MOL000422	kaempferol	Nuclear receptor subfamily 1 group I member 3
Semen Cuseutae	MOL000422	kaempferol	Insulin receptor
Semen Cuseutae	MOL000422	kaempferol	Type I iodothyronine deiodinase
Semen Cuseutae	MOL000422	kaempferol	Serine/threonine-protein phosphatase 2B catalytic subunit alpha isoform
Semen Cuseutae	MOL000422	kaempferol	Peroxidase C1A
Semen Cuseutae	MOL000422	kaempferol	Glutathione S-transferase Mu 1
Semen Cuseutae	MOL000422	kaempferol	Glutathione S-transferase Mu 2
Semen Cuseutae	MOL000422	kaempferol	Aldo-keto reductase family 1 member C3
Semen Cuseutae	MOL000422	kaempferol	Antileukoproteinase
Semen Cuseutae	MOL005043	campest-5-en-3beta-ol	Progesterone receptor
Semen Cuseutae	MOL005440	Isofucosterol	Progesterone receptor
Semen Cuseutae	MOL005440	Isofucosterol	Mineralocorticoid receptor
Semen Cuseutae	MOL005440	Isofucosterol	4-aminobutyrate aminotransferase, mitochondrial
Semen Cuseutae	MOL005440	Isofucosterol	Gamma-aminobutyric acid receptor subunit alpha-1
Semen Cuseutae	MOL005440	Isofucosterol	Alcohol dehydrogenase 1B
Semen Cuseutae	MOL005440	Isofucosterol	Alcohol dehydrogenase 1C
Semen Cuseutae	MOL005440	Isofucosterol	Alcohol dehydrogenase 1A
Semen Cuseutae	MOL005440	Isofucosterol	Cytochrome P450-cam
Semen Cuseutae	MOL005440	Isofucosterol	Lysozyme
Semen Cuseutae	MOL005440	Isofucosterol	Bacillolysin

Semen Cuseutae	MOL005440	Isofucosterol	Nicotinate-nucleotide--dimethylbenzimidazole phosphoribosyltransferase
Semen Cuseutae	MOL005440	Isofucosterol	Nuclear receptor coactivator 2
Semen Cuseutae	MOL005944	matrine	Transcription factor p65
Semen Cuseutae	MOL005944	matrine	72 kDa type IV collagenase
Semen Cuseutae	MOL005944	matrine	Tumor necrosis factor
Semen Cuseutae	MOL005944	matrine	Interleukin-6
Semen Cuseutae	MOL005944	matrine	Caspase-3
Semen Cuseutae	MOL005944	matrine	Myc proto-oncogene protein
Semen Cuseutae	MOL005944	matrine	Intercellular adhesion molecule 1
Semen Cuseutae	MOL005944	matrine	Heparanase
Semen Cuseutae	MOL005944	matrine	Immediate early response 3-interacting protein 1
Semen Cuseutae	MOL005944	matrine	CD44 antigen
Semen Cuseutae	MOL000953	CLR	Progesterone receptor
Semen Cuseutae	MOL000953	CLR	Mineralocorticoid receptor
Semen Cuseutae	MOL000953	CLR	Cytochrome P450-cam
Semen Cuseutae	MOL000953	CLR	Nuclear receptor coactivator 2
Semen Cuseutae	MOL000098	quercetin	Prostaglandin G/H synthase 1
Semen Cuseutae	MOL000098	quercetin	Androgen receptor
Semen Cuseutae	MOL000098	quercetin	Peroxisome proliferator activated receptor gamma
Semen Cuseutae	MOL000098	quercetin	Prostaglandin G/H synthase 2
Semen Cuseutae	MOL000098	quercetin	Heat shock protein HSP 90
Semen Cuseutae	MOL000098	quercetin	Phosphatidylinositol-4,5-bisphosphate 3-kinase catalytic subunit, gamma isoform
Semen Cuseutae	MOL000098	quercetin	Nuclear receptor coactivator 2
Semen Cuseutae	MOL000098	quercetin	Dipeptidyl peptidase IV

Semen Cuseutae	MOL000098	quercetin	Aldose reductase
Semen Cuseutae	MOL000098	quercetin	Trypsin-1
Semen Cuseutae	MOL000098	quercetin	DNA topoisomerase II
Semen Cuseutae	MOL000098	quercetin	Thrombin
Semen Cuseutae	MOL000098	quercetin	Potassium voltage-gated channel subfamily H member 2
Semen Cuseutae	MOL000098	quercetin	Sodium channel protein type 5 subunit alpha
Semen Cuseutae	MOL000098	quercetin	Coagulation factor Xa
Semen Cuseutae	MOL000098	quercetin	Beta-2 adrenergic receptor
Semen Cuseutae	MOL000098	quercetin	Stromelysin-1
Semen Cuseutae	MOL000098	quercetin	mRNA of PKA Catalytic Subunit C-alpha
Semen Cuseutae	MOL000098	quercetin	Coagulation factor VII
Semen Cuseutae	MOL000098	quercetin	Nitric-oxide synthase, endothelial
Semen Cuseutae	MOL000098	quercetin	Retinoic acid receptor RXR-alpha
Semen Cuseutae	MOL000098	quercetin	Acetylcholinesterase
Semen Cuseutae	MOL000098	quercetin	Gamma-aminobutyric acid receptor subunit alpha-1
Semen Cuseutae	MOL000098	quercetin	Amine oxidase [flavin-containing] B
Semen Cuseutae	MOL000098	quercetin	Transcription factor p65
Semen Cuseutae	MOL000098	quercetin	Epidermal growth factor receptor
Semen Cuseutae	MOL000098	quercetin	RAC-alpha serine/threonine-protein kinase
Semen Cuseutae	MOL000098	quercetin	Vascular endothelial growth factor A
Semen Cuseutae	MOL000098	quercetin	G1/S-specific cyclin-D1
Semen Cuseutae	MOL000098	quercetin	Apoptosis regulator Bcl-2
Semen Cuseutae	MOL000098	quercetin	Bcl-2-like protein 1
Semen Cuseutae	MOL000098	quercetin	Proto-oncogene c-Fos
Semen Cuseutae	MOL000098	quercetin	Cyclin-dependent kinase inhibitor 1

Semen Cuseutae	MOL000098	quercetin	Eukaryotic translation initiation factor 6
Semen Cuseutae	MOL000098	quercetin	Apoptosis regulator BAX
Semen Cuseutae	MOL000098	quercetin	Caspase-9
Semen Cuseutae	MOL000098	quercetin	Urokinase-type plasminogen activator
Semen Cuseutae	MOL000098	quercetin	72 kDa type IV collagenase
Semen Cuseutae	MOL000098	quercetin	Matrix metalloproteinase-9
Semen Cuseutae	MOL000098	quercetin	Mitogen-activated protein kinase 1
Semen Cuseutae	MOL000098	quercetin	Interleukin-10
Semen Cuseutae	MOL000098	quercetin	Pro-epidermal growth factor
Semen Cuseutae	MOL000098	quercetin	Retinoblastoma-associated protein
Semen Cuseutae	MOL000098	quercetin	Tumor necrosis factor
Semen Cuseutae	MOL000098	quercetin	Transcription factor AP-1
Semen Cuseutae	MOL000098	quercetin	Interleukin-6
Semen Cuseutae	MOL000098	quercetin	Cyclin-dependent kinase inhibitor 2A, isoforms 1/2/3
Semen Cuseutae	MOL000098	quercetin	Activator of 90 kDa heat shock protein ATPase homolog 1
Semen Cuseutae	MOL000098	quercetin	Caspase-3
Semen Cuseutae	MOL000098	quercetin	Cellular tumor antigen p53
Semen Cuseutae	MOL000098	quercetin	ETS domain-containing protein Elk-1
Semen Cuseutae	MOL000098	quercetin	NF-kappa-B inhibitor alpha
Semen Cuseutae	MOL000098	quercetin	NADPH--cytochrome P450 reductase
Semen Cuseutae	MOL000098	quercetin	Ornithine decarboxylase
Semen Cuseutae	MOL000098	quercetin	Xanthine dehydrogenase/oxidase
Semen Cuseutae	MOL000098	quercetin	Caspase-8
Semen Cuseutae	MOL000098	quercetin	DNA topoisomerase 1
Semen Cuseutae	MOL000098	quercetin	RAF proto-oncogene serine/threonine-protein kinase

Semen Cuseutae	MOL000098	quercetin	Superoxide dismutase [Cu-Zn]
Semen Cuseutae	MOL000098	quercetin	Protein kinase C alpha type
Semen Cuseutae	MOL000098	quercetin	Interstitial collagenase
Semen Cuseutae	MOL000098	quercetin	Hypoxia-inducible factor 1-alpha
Semen Cuseutae	MOL000098	quercetin	Signal transducer and activator of transcription 1-alpha/beta
Semen Cuseutae	MOL000098	quercetin	Protein CBFA2T1
Semen Cuseutae	MOL000098	quercetin	Probable E3 ubiquitin-protein ligase HERC5
Semen Cuseutae	MOL000098	quercetin	Cell division control protein 2 homolog
Semen Cuseutae	MOL000098	quercetin	78 kDa glucose-regulated protein
Semen Cuseutae	MOL000098	quercetin	Receptor tyrosine-protein kinase erbB-2
Semen Cuseutae	MOL000098	quercetin	Peroxisome proliferator-activated receptor gamma
Semen Cuseutae	MOL000098	quercetin	Acetyl-CoA carboxylase 1
Semen Cuseutae	MOL000098	quercetin	Heme oxygenase 1
Semen Cuseutae	MOL000098	quercetin	Cytochrome P450 3A4
Semen Cuseutae	MOL000098	quercetin	Cytochrome P450 1A2
Semen Cuseutae	MOL000098	quercetin	Caveolin-1
Semen Cuseutae	MOL000098	quercetin	Myc proto-oncogene protein
Semen Cuseutae	MOL000098	quercetin	Tissue factor
Semen Cuseutae	MOL000098	quercetin	Gap junction alpha-1 protein
Semen Cuseutae	MOL000098	quercetin	Cytochrome P450 1A1
Semen Cuseutae	MOL000098	quercetin	Intercellular adhesion molecule 1
Semen Cuseutae	MOL000098	quercetin	Interleukin-1 beta
Semen Cuseutae	MOL000098	quercetin	C-C motif chemokine 2
Semen Cuseutae	MOL000098	quercetin	E-selectin
Semen Cuseutae	MOL000098	quercetin	Vascular cell adhesion protein 1

Semen Cuseutae	MOL000098	quercetin	Prostaglandin E2 receptor EP3 subtype
Semen Cuseutae	MOL000098	quercetin	Interleukin-8
Semen Cuseutae	MOL000098	quercetin	Protein kinase C beta type
Semen Cuseutae	MOL000098	quercetin	Baculoviral IAP repeat-containing protein 5
Semen Cuseutae	MOL000098	quercetin	Dual oxidase 2
Semen Cuseutae	MOL000098	quercetin	Nitric oxide synthase, endothelial
Semen Cuseutae	MOL000098	quercetin	Heat shock protein beta-1
Semen Cuseutae	MOL000098	quercetin	Transforming growth factor beta-1
Semen Cuseutae	MOL000098	quercetin	Estrogen sulfotransferase
Semen Cuseutae	MOL000098	quercetin	Maltase-glucoamylase, intestinal
Semen Cuseutae	MOL000098	quercetin	Interleukin-2
Semen Cuseutae	MOL000098	quercetin	Nuclear receptor subfamily 1 group I member 2
Semen Cuseutae	MOL000098	quercetin	Cytochrome P450 1B1
Semen Cuseutae	MOL000098	quercetin	G2/mitotic-specific cyclin-B1
Semen Cuseutae	MOL000098	quercetin	Tissue-type plasminogen activator
Semen Cuseutae	MOL000098	quercetin	Thrombomodulin
Semen Cuseutae	MOL000098	quercetin	Plasminogen activator inhibitor 1
Semen Cuseutae	MOL000098	quercetin	Collagen alpha-1(I) chain
Semen Cuseutae	MOL000098	quercetin	Interferon gamma
Semen Cuseutae	MOL000098	quercetin	Arachidonate 5-lipoxygenase
Semen Cuseutae	MOL000098	quercetin	Phosphatidylinositol-3,4,5-trisphosphate 3-phosphatase and dual-specificity protein phosphatase PTEN
Semen Cuseutae	MOL000098	quercetin	Interleukin-1 alpha
Semen Cuseutae	MOL000098	quercetin	Myeloperoxidase
Semen Cuseutae	MOL000098	quercetin	DNA topoisomerase 2-alpha

Semen Cuseutae	MOL000098	quercetin	Neutrophil cytosol factor 1
Semen Cuseutae	MOL000098	quercetin	ATP-binding cassette sub-family G member 2
Semen Cuseutae	MOL000098	quercetin	Hyaluronan synthase 2
Semen Cuseutae	MOL000098	quercetin	Glutathione S-transferase P
Semen Cuseutae	MOL000098	quercetin	Nuclear factor erythroid 2-related factor 2
Semen Cuseutae	MOL000098	quercetin	NAD(P)H dehydrogenase [quinone] 1
Semen Cuseutae	MOL000098	quercetin	Poly [ADP-ribose] polymerase 1
Semen Cuseutae	MOL000098	quercetin	Aryl hydrocarbon receptor
Semen Cuseutae	MOL000098	quercetin	26S proteasome non-ATPase regulatory subunit 3
Semen Cuseutae	MOL000098	quercetin	Solute carrier family 2, facilitated glucose transporter member 4
Semen Cuseutae	MOL000098	quercetin	Collagen alpha-1(III) chain
Semen Cuseutae	MOL000098	quercetin	DNA gyrase subunit B
Semen Cuseutae	MOL000098	quercetin	C-X-C motif chemokine 11
Semen Cuseutae	MOL000098	quercetin	C-X-C motif chemokine 2
Semen Cuseutae	MOL000098	quercetin	DDB1- and CUL4-associated factor 5
Semen Cuseutae	MOL000098	quercetin	Nuclear receptor subfamily 1 group I member 3
Semen Cuseutae	MOL000098	quercetin	Serine/threonine-protein kinase Chk2
Semen Cuseutae	MOL000098	quercetin	Insulin receptor
Semen Cuseutae	MOL000098	quercetin	Claudin-4
Semen Cuseutae	MOL000098	quercetin	Peroxisome proliferator-activated receptor alpha
Semen Cuseutae	MOL000098	quercetin	Peroxisome proliferator-activated receptor delta

Semen Cuseutae	MOL000098	quercetin	Heat shock factor protein 1
Semen Cuseutae	MOL000098	quercetin	C-reactive protein
Semen Cuseutae	MOL000098	quercetin	C-X-C motif chemokine 10
Semen Cuseutae	MOL000098	quercetin	Inhibitor of nuclear factor kappa-B kinase subunit alpha
Semen Cuseutae	MOL000098	quercetin	Osteopontin
Semen Cuseutae	MOL000098	quercetin	Runt-related transcription factor 2
Semen Cuseutae	MOL000098	quercetin	Ras association domain-containing protein 1
Semen Cuseutae	MOL000098	quercetin	Transcription factor E2F1
Semen Cuseutae	MOL000098	quercetin	Transcription factor E2F2
Semen Cuseutae	MOL000098	quercetin	Prostatic acid phosphatase
Semen Cuseutae	MOL000098	quercetin	Cathepsin D
Semen Cuseutae	MOL000098	quercetin	Insulin-like growth factor-binding protein 3
Semen Cuseutae	MOL000098	quercetin	Insulin-like growth factor II
Semen Cuseutae	MOL000098	quercetin	CD40 ligand
Semen Cuseutae	MOL000098	quercetin	Interferon regulatory factor 1
Semen Cuseutae	MOL000098	quercetin	Receptor tyrosine-protein kinase erbB-3
Semen Cuseutae	MOL000098	quercetin	Serum paraoxonase/arylesterase 1
Semen Cuseutae	MOL000098	quercetin	Type I iodothyronine deiodinase
Semen Cuseutae	MOL000098	quercetin	Procollagen C-endopeptidase enhancer 1
Semen Cuseutae	MOL000098	quercetin	Puromycin-sensitive aminopeptidase
Semen Cuseutae	MOL000098	quercetin	Hexokinase-2
Semen Cuseutae	MOL000098	quercetin	Homeobox protein Nkx-3.1
Semen Cuseutae	MOL000098	quercetin	Ras GTPase-activating protein 1
Semen Cuseutae	MOL000098	quercetin	Peroxidase C1A
Semen Cuseutae	MOL000098	quercetin	Glutathione S-transferase Mu 1

Semen Cuseutae	MOL000098	quercetin	Glutathione S-transferase Mu 2
Rhizoma Corydal	MOL001454	berberine	Nitric oxide synthase, inducible
Rhizoma Corydal	MOL001454	berberine	Prostaglandin G/H synthase 1
Rhizoma Corydal	MOL001454	berberine	Potassium voltage-gated channel subfamily H member 2
Rhizoma Corydal	MOL001454	berberine	Estrogen receptor
Rhizoma Corydal	MOL001454	berberine	Androgen receptor
Rhizoma Corydal	MOL001454	berberine	Sodium channel protein type 5 subunit alpha
Rhizoma Corydal	MOL001454	berberine	Coagulation factor Xa
Rhizoma Corydal	MOL001454	berberine	Prostaglandin G/H synthase 2
Rhizoma Corydal	MOL001454	berberine	Nitric-oxide synthase, endothelial
Rhizoma Corydal	MOL001454	berberine	Retinoic acid receptor RXR-alpha
Rhizoma Corydal	MOL001454	berberine	Beta-2 adrenergic receptor
Rhizoma Corydal	MOL001454	berberine	Heat shock protein HSP 90
Rhizoma Corydal	MOL001454	berberine	mRNA of PKA Catalytic Subunit C-alpha
Rhizoma Corydal	MOL001454	berberine	Trypsin-1
Rhizoma Corydal	MOL001454	berberine	Nuclear receptor coactivator 2
Rhizoma Corydal	MOL001454	berberine	cAMP and cAMP-inhibited cGMP 3',5'-cyclic phosphodiesterase 10A
Rhizoma Corydal	MOL001454	berberine	Calmodulin
Rhizoma Corydal	MOL001458	coptisine	Nitric oxide synthase, inducible
Rhizoma Corydal	MOL001458	coptisine	Prostaglandin G/H synthase 1
Rhizoma Corydal	MOL001458	coptisine	Potassium voltage-gated channel subfamily H member 2
Rhizoma Corydal	MOL001458	coptisine	Estrogen receptor
Rhizoma Corydal	MOL001458	coptisine	Androgen receptor
Rhizoma Corydal	MOL001458	coptisine	Sodium channel protein type 5 subunit alpha

Rhizoma Corydal MOL001458	coptisine	Prostaglandin G/H synthase 2
Rhizoma Corydal MOL001458	coptisine	Nitric-oxide synthase, endothelial
Rhizoma Corydal MOL001458	coptisine	Trypsin-1
Rhizoma Corydal MOL001460	Cryptopin	Prostaglandin G/H synthase 1
Rhizoma Corydal MOL001460	Cryptopin	Dopamine D1 receptor
Rhizoma Corydal MOL001460	Cryptopin	Muscarinic acetylcholine receptor M3
Rhizoma Corydal MOL001460	Cryptopin	Potassium voltage-gated channel subfamily H member 2
Rhizoma Corydal MOL001460	Cryptopin	Muscarinic acetylcholine receptor M1
Rhizoma Corydal MOL001460	Cryptopin	Sodium channel protein type 5 subunit alpha
Rhizoma Corydal MOL001460	Cryptopin	Coagulation factor Xa
Rhizoma Corydal MOL001460	Cryptopin	Muscarinic acetylcholine receptor M5
Rhizoma Corydal MOL001460	Cryptopin	Prostaglandin G/H synthase 2
Rhizoma Corydal MOL001460	Cryptopin	5-hydroxytryptamine receptor 3A
Rhizoma Corydal MOL001460	Cryptopin	Coagulation factor VII
Rhizoma Corydal MOL001460	Cryptopin	Vascular endothelial growth factor receptor 2
Rhizoma Corydal MOL001460	Cryptopin	Muscarinic acetylcholine receptor M4
Rhizoma Corydal MOL001460	Cryptopin	Delta-type opioid receptor
Rhizoma Corydal MOL001460	Cryptopin	CGMP-inhibited 3',5'-cyclic phosphodiesterase A
Rhizoma Corydal MOL001460	Cryptopin	Alpha-1B adrenergic receptor
Rhizoma Corydal MOL001460	Cryptopin	Beta-2 adrenergic receptor
Rhizoma Corydal MOL001460	Cryptopin	Alpha-1D adrenergic receptor
Rhizoma Corydal MOL001460	Cryptopin	DNA topoisomerase II
Rhizoma Corydal MOL001460	Cryptopin	Mu-type opioid receptor
Rhizoma Corydal MOL001460	Cryptopin	Heat shock protein HSP 90
Rhizoma Corydal MOL001460	Cryptopin	mRNA of PKA Catalytic Subunit C-alpha

Rhizoma Corydal	MOL001460	Cryptopin	Voltage-dependent L-type calcium channel subunit alpha-1S
Rhizoma Corydal	MOL001460	Cryptopin	Nuclear receptor coactivator 1
Rhizoma Corydal	MOL001460	Cryptopin	cAMP and cAMP-inhibited cGMP 3',5'-cyclic phosphodiesterase 10A
Rhizoma Corydal	MOL001460	Cryptopin	Calmodulin
Rhizoma Corydal	MOL001460	Cryptopin	5-hydroxytryptamine 2A receptor
Rhizoma Corydal	MOL001460	Cryptopin	Sodium-dependent serotonin transporter
Rhizoma Corydal	MOL001461	Dihydrochelery thrine	Nitric oxide synthase, inducible
Rhizoma Corydal	MOL001461	Dihydrochelery thrine	Prostaglandin G/H synthase 1
Rhizoma Corydal	MOL001461	Dihydrochelery thrine	Potassium voltage-gated channel subfamily H member 2
Rhizoma Corydal	MOL001461	Dihydrochelery thrine	Estrogen receptor
Rhizoma Corydal	MOL001461	Dihydrochelery thrine	Androgen receptor
Rhizoma Corydal	MOL001461	Dihydrochelery thrine	Sodium channel protein type 5 subunit alpha
Rhizoma Corydal	MOL001461	Dihydrochelery thrine	Peroxisome proliferator activated receptor gamma
Rhizoma Corydal	MOL001461	Dihydrochelery thrine	Coagulation factor Xa
Rhizoma Corydal	MOL001461	Dihydrochelery thrine	Prostaglandin G/H synthase 2
Rhizoma Corydal	MOL001461	Dihydrochelery thrine	Nitric-oxide synthase, endothelial

Rhizoma Corydal	MOL001461	Dihydrochelery thrine	Retinoic acid receptor RXR-alpha
Rhizoma Corydal	MOL001461	Dihydrochelery thrine	Acetylcholinesterase
Rhizoma Corydal	MOL001461	Dihydrochelery thrine	Estrogen receptor beta
Rhizoma Corydal	MOL001461	Dihydrochelery thrine	Mitogen-activated protein kinase 14
Rhizoma Corydal	MOL001461	Dihydrochelery thrine	Heat shock protein HSP 90
Rhizoma Corydal	MOL001461	Dihydrochelery thrine	Cell division protein kinase 2
Rhizoma Corydal	MOL001461	Dihydrochelery thrine	mRNA of PKA Catalytic Subunit C-alpha
Rhizoma Corydal	MOL001461	Dihydrochelery thrine	Trypsin-1
Rhizoma Corydal	MOL001461	Dihydrochelery thrine	Proto-oncogene serine/threonine-protein kinase Pim-1
Rhizoma Corydal	MOL001461	Dihydrochelery thrine	Cyclin-A2
Rhizoma Corydal	MOL001461	Dihydrochelery thrine	Nuclear receptor coactivator 2
Rhizoma Corydal	MOL001461	Dihydrochelery thrine	Calmodulin
Rhizoma Corydal	MOL001463	Dihydrosangui narine	Nitric oxide synthase, inducible
Rhizoma Corydal	MOL001463	Dihydrosangui narine	Prostaglandin G/H synthase 1
Rhizoma Corydal	MOL001463	Dihydrosangui narine	Estrogen receptor

Rhizoma Corydal	MOL001463	Dihydrosanguinarine	Androgen receptor
Rhizoma Corydal	MOL001463	Dihydrosanguinarine	Peroxisome proliferator activated receptor gamma
Rhizoma Corydal	MOL001463	Dihydrosanguinarine	Coagulation factor Xa
Rhizoma Corydal	MOL001463	Dihydrosanguinarine	Prostaglandin G/H synthase 2
Rhizoma Corydal	MOL001463	Dihydrosanguinarine	Acetylcholinesterase
Rhizoma Corydal	MOL001463	Dihydrosanguinarine	Estrogen receptor beta
Rhizoma Corydal	MOL001463	Dihydrosanguinarine	Mitogen-activated protein kinase 14
Rhizoma Corydal	MOL001463	Dihydrosanguinarine	Cell division protein kinase 2
Rhizoma Corydal	MOL001463	Dihydrosanguinarine	mRNA of PKA Catalytic Subunit C-alpha
Rhizoma Corydal	MOL001463	Dihydrosanguinarine	Trypsin-1
Rhizoma Corydal	MOL001463	Dihydrosanguinarine	Proto-oncogene serine/threonine-protein kinase Pim-1
Rhizoma Corydal	MOL001463	Dihydrosanguinarine	Cyclin-A2
Rhizoma Corydal	MOL001463	Dihydrosanguinarine	Nuclear receptor coactivator 2
Rhizoma Corydal	MOL001463	Dihydrosanguinarine	Calmodulin
Rhizoma Corydal	MOL001474	sanguinarine	Prostaglandin G/H synthase 1
Rhizoma Corydal	MOL001474	sanguinarine	Prostaglandin G/H synthase 2

Rhizoma Corydal	MOL001474	sanguinarine	Retinoic acid receptor RXR-alpha
Rhizoma Corydal	MOL001474	sanguinarine	mRNA of PKA Catalytic Subunit C-alpha
Rhizoma Corydal	MOL000217	(S)-Scoulerine	Prostaglandin G/H synthase 1
Rhizoma Corydal	MOL000217	(S)-Scoulerine	Dopamine D1 receptor
Rhizoma Corydal	MOL000217	(S)-Scoulerine	Muscarinic acetylcholine receptor M3
Rhizoma Corydal	MOL000217	(S)-Scoulerine	Potassium voltage-gated channel subfamily H member 2
Rhizoma Corydal	MOL000217	(S)-Scoulerine	Muscarinic acetylcholine receptor M1
Rhizoma Corydal	MOL000217	(S)-Scoulerine	D(1B) dopamine receptor
Rhizoma Corydal	MOL000217	(S)-Scoulerine	Sodium channel protein type 5 subunit alpha
Rhizoma Corydal	MOL000217	(S)-Scoulerine	Coagulation factor Xa
Rhizoma Corydal	MOL000217	(S)-Scoulerine	Muscarinic acetylcholine receptor M5
Rhizoma Corydal	MOL000217	(S)-Scoulerine	Prostaglandin G/H synthase 2
Rhizoma Corydal	MOL000217	(S)-Scoulerine	Alpha-2A adrenergic receptor
Rhizoma Corydal	MOL000217	(S)-Scoulerine	Carbonic anhydrase II
Rhizoma Corydal	MOL000217	(S)-Scoulerine	Coagulation factor VII
Rhizoma Corydal	MOL000217	(S)-Scoulerine	Alpha-2C adrenergic receptor
Rhizoma Corydal	MOL000217	(S)-Scoulerine	Muscarinic acetylcholine receptor M4
Rhizoma Corydal	MOL000217	(S)-Scoulerine	Delta-type opioid receptor
Rhizoma Corydal	MOL000217	(S)-Scoulerine	CGMP-inhibited 3',5'-cyclic phosphodiesterase A
Rhizoma Corydal	MOL000217	(S)-Scoulerine	5-hydroxytryptamine 2A receptor
Rhizoma Corydal	MOL000217	(S)-Scoulerine	Alpha-1A adrenergic receptor
Rhizoma Corydal	MOL000217	(S)-Scoulerine	5-hydroxytryptamine 2C receptor
Rhizoma Corydal	MOL000217	(S)-Scoulerine	Muscarinic acetylcholine receptor M2
Rhizoma Corydal	MOL000217	(S)-Scoulerine	Alpha-1B adrenergic receptor
Rhizoma Corydal	MOL000217	(S)-Scoulerine	D(3) dopamine receptor

Rhizoma Corydal	MOL000217	(S)-Scoulerine	Sodium-dependent dopamine transporter
Rhizoma Corydal	MOL000217	(S)-Scoulerine	Beta-2 adrenergic receptor
Rhizoma Corydal	MOL000217	(S)-Scoulerine	Alpha-1D adrenergic receptor
Rhizoma Corydal	MOL000217	(S)-Scoulerine	DNA topoisomerase II
Rhizoma Corydal	MOL000217	(S)-Scoulerine	Sodium-dependent serotonin transporter
Rhizoma Corydal	MOL000217	(S)-Scoulerine	Mu-type opioid receptor
Rhizoma Corydal	MOL000217	(S)-Scoulerine	Heat shock protein HSP 90
Rhizoma Corydal	MOL000217	(S)-Scoulerine	Nuclear receptor coactivator 2
Rhizoma Corydal	MOL000217	(S)-Scoulerine	Nuclear receptor coactivator 1
Rhizoma Corydal	MOL000217	(S)-Scoulerine	Calmodulin
Rhizoma Corydal	MOL000217	(S)-Scoulerine	Retinoic acid receptor RXR-alpha
Rhizoma Corydal	MOL000217	(S)-Scoulerine	Sodium-dependent noradrenaline transporter
Rhizoma Corydal	MOL000217	(S)-Scoulerine	Alpha-2B adrenergic receptor
Rhizoma Corydal	MOL002670	Cavidine	Prostaglandin G/H synthase 1
Rhizoma Corydal	MOL002670	Cavidine	Muscarinic acetylcholine receptor M3
Rhizoma Corydal	MOL002670	Cavidine	Potassium voltage-gated channel subfamily H member 2
Rhizoma Corydal	MOL002670	Cavidine	Muscarinic acetylcholine receptor M1
Rhizoma Corydal	MOL002670	Cavidine	Beta-1 adrenergic receptor
Rhizoma Corydal	MOL002670	Cavidine	Sodium channel protein type 5 subunit alpha
Rhizoma Corydal	MOL002670	Cavidine	Coagulation factor Xa
Rhizoma Corydal	MOL002670	Cavidine	Muscarinic acetylcholine receptor M5
Rhizoma Corydal	MOL002670	Cavidine	Prostaglandin G/H synthase 2
Rhizoma Corydal	MOL002670	Cavidine	5-hydroxytryptamine receptor 3A
Rhizoma Corydal	MOL002670	Cavidine	Alpha-2C adrenergic receptor
Rhizoma Corydal	MOL002670	Cavidine	Muscarinic acetylcholine receptor M4

Rhizoma Corydal	MOL002670	Cavidine	Retinoic acid receptor RXR-alpha
Rhizoma Corydal	MOL002670	Cavidine	Delta-type opioid receptor
Rhizoma Corydal	MOL002670	Cavidine	5-hydroxytryptamine 2A receptor
Rhizoma Corydal	MOL002670	Cavidine	5-hydroxytryptamine 2C receptor
Rhizoma Corydal	MOL002670	Cavidine	Alpha-1B adrenergic receptor
Rhizoma Corydal	MOL002670	Cavidine	Beta-2 adrenergic receptor
Rhizoma Corydal	MOL002670	Cavidine	Alpha-1D adrenergic receptor
Rhizoma Corydal	MOL002670	Cavidine	DNA topoisomerase II
Rhizoma Corydal	MOL002670	Cavidine	Mu-type opioid receptor
Rhizoma Corydal	MOL002670	Cavidine	Heat shock protein HSP 90
Rhizoma Corydal	MOL002670	Cavidine	Retinoic acid receptor RXR-beta
Rhizoma Corydal	MOL002670	Cavidine	Calmodulin
Rhizoma Corydal	MOL002670	Cavidine	Dopamine D1 receptor
Rhizoma Corydal	MOL002670	Cavidine	Sodium-dependent serotonin transporter
Rhizoma Corydal	MOL002670	Cavidine	Coagulation factor VII
Rhizoma Corydal	MOL002670	Cavidine	cAMP and cAMP-inhibited cGMP 3',5'-cyclic phosphodiesterase 10A
Rhizoma Corydal	MOL002903	(R)-Canadine	Prostaglandin G/H synthase 1
Rhizoma Corydal	MOL002903	(R)-Canadine	Muscarinic acetylcholine receptor M3
Rhizoma Corydal	MOL002903	(R)-Canadine	Potassium voltage-gated channel subfamily H member 2
Rhizoma Corydal	MOL002903	(R)-Canadine	Muscarinic acetylcholine receptor M1
Rhizoma Corydal	MOL002903	(R)-Canadine	Sodium channel protein type 5 subunit alpha
Rhizoma Corydal	MOL002903	(R)-Canadine	Coagulation factor Xa
Rhizoma Corydal	MOL002903	(R)-Canadine	Muscarinic acetylcholine receptor M5
Rhizoma Corydal	MOL002903	(R)-Canadine	Prostaglandin G/H synthase 2
Rhizoma Corydal	MOL002903	(R)-Canadine	5-hydroxytryptamine receptor 3A
Rhizoma Corydal	MOL002903	(R)-Canadine	Alpha-2C adrenergic receptor

Rhizoma Corydal	MOL002903	(R)-Canadine	Muscarinic acetylcholine receptor M4
Rhizoma Corydal	MOL002903	(R)-Canadine	Delta-type opioid receptor
Rhizoma Corydal	MOL002903	(R)-Canadine	5-hydroxytryptamine 2A receptor
Rhizoma Corydal	MOL002903	(R)-Canadine	5-hydroxytryptamine 2C receptor
Rhizoma Corydal	MOL002903	(R)-Canadine	Alpha-1B adrenergic receptor
Rhizoma Corydal	MOL002903	(R)-Canadine	Sodium-dependent dopamine transporter
Rhizoma Corydal	MOL002903	(R)-Canadine	Beta-2 adrenergic receptor
Rhizoma Corydal	MOL002903	(R)-Canadine	Alpha-1D adrenergic receptor
Rhizoma Corydal	MOL002903	(R)-Canadine	Sodium-dependent serotonin transporter
Rhizoma Corydal	MOL002903	(R)-Canadine	Mu-type opioid receptor
Rhizoma Corydal	MOL002903	(R)-Canadine	Heat shock protein HSP 90
Rhizoma Corydal	MOL002903	(R)-Canadine	mRNA of PKA Catalytic Subunit C-alpha
Rhizoma Corydal	MOL002903	(R)-Canadine	cAMP and cAMP-inhibited cGMP 3',5'-cyclic phosphodiesterase 10A
Rhizoma Corydal	MOL002903	(R)-Canadine	Calmodulin
Rhizoma Corydal	MOL002903	(R)-Canadine	Dopamine D1 receptor
Rhizoma Corydal	MOL002903	(R)-Canadine	D(1B) dopamine receptor
Rhizoma Corydal	MOL002903	(R)-Canadine	Retinoic acid receptor RXR-alpha
Rhizoma Corydal	MOL002903	(R)-Canadine	Sodium-dependent noradrenaline transporter
Rhizoma Corydal	MOL002903	(R)-Canadine	Alpha-1A adrenergic receptor
Rhizoma Corydal	MOL002903	(R)-Canadine	Muscarinic acetylcholine receptor M2
Rhizoma Corydal	MOL002903	(R)-Canadine	Calcium-activated potassium channel subunit alpha 1
Rhizoma Corydal	MOL000359	sitosterol	Progesterone receptor
Rhizoma Corydal	MOL000359	sitosterol	Nuclear receptor coactivator 2
Rhizoma Corydal	MOL000359	sitosterol	Mineralocorticoid receptor

Rhizoma Corydal MOL004071	Hyndarin	Dopamine D1 receptor
Rhizoma Corydal MOL004071	Hyndarin	Muscarinic acetylcholine receptor M3
Rhizoma Corydal MOL004071	Hyndarin	Potassium voltage-gated channel subfamily H member 2
Rhizoma Corydal MOL004071	Hyndarin	Muscarinic acetylcholine receptor M1
Rhizoma Corydal MOL004071	Hyndarin	Sodium channel protein type 5 subunit alpha
Rhizoma Corydal MOL004071	Hyndarin	Coagulation factor Xa
Rhizoma Corydal MOL004071	Hyndarin	Muscarinic acetylcholine receptor M5
Rhizoma Corydal MOL004071	Hyndarin	Prostaglandin G/H synthase 2
Rhizoma Corydal MOL004071	Hyndarin	5-hydroxytryptamine receptor 3A
Rhizoma Corydal MOL004071	Hyndarin	Carbonic anhydrase II
Rhizoma Corydal MOL004071	Hyndarin	Alpha-2C adrenergic receptor
Rhizoma Corydal MOL004071	Hyndarin	Muscarinic acetylcholine receptor M4
Rhizoma Corydal MOL004071	Hyndarin	Delta-type opioid receptor
Rhizoma Corydal MOL004071	Hyndarin	5-hydroxytryptamine 2A receptor
Rhizoma Corydal MOL004071	Hyndarin	Sodium-dependent noradrenaline transporter
Rhizoma Corydal MOL004071	Hyndarin	5-hydroxytryptamine 2C receptor
Rhizoma Corydal MOL004071	Hyndarin	Muscarinic acetylcholine receptor M2
Rhizoma Corydal MOL004071	Hyndarin	Alpha-1B adrenergic receptor
Rhizoma Corydal MOL004071	Hyndarin	Sodium-dependent dopamine transporter
Rhizoma Corydal MOL004071	Hyndarin	Beta-2 adrenergic receptor
Rhizoma Corydal MOL004071	Hyndarin	Alpha-1D adrenergic receptor
Rhizoma Corydal MOL004071	Hyndarin	Sodium-dependent serotonin transporter
Rhizoma Corydal MOL004071	Hyndarin	Mu-type opioid receptor
Rhizoma Corydal MOL004071	Hyndarin	Heat shock protein HSP 90
Rhizoma Corydal MOL004071	Hyndarin	Retinoic acid receptor RXR-beta

Rhizoma Corydal	MOL004071	Hyndarin	Calmodulin
Rhizoma Corydal	MOL004071	Hyndarin	Prostaglandin G/H synthase 1
Rhizoma Corydal	MOL004071	Hyndarin	Beta-1 adrenergic receptor
Rhizoma Corydal	MOL004071	Hyndarin	Retinoic acid receptor RXR-alpha
Rhizoma Corydal	MOL004071	Hyndarin	D(3) dopamine receptor
Rhizoma Corydal	MOL004071	Hyndarin	D(2) dopamine receptor
Rhizoma Corydal	MOL004071	Hyndarin	Nuclear receptor coactivator 1
Rhizoma Corydal	MOL004190	(-)-alpha-N-methylcanadine	Muscarinic acetylcholine receptor M3
Rhizoma Corydal	MOL004190	(-)-alpha-N-methylcanadine	Potassium voltage-gated channel subfamily H member 2
Rhizoma Corydal	MOL004190	(-)-alpha-N-methylcanadine	Muscarinic acetylcholine receptor M1
Rhizoma Corydal	MOL004190	(-)-alpha-N-methylcanadine	Sodium channel protein type 5 subunit alpha
Rhizoma Corydal	MOL004190	(-)-alpha-N-methylcanadine	Coagulation factor Xa
Rhizoma Corydal	MOL004190	(-)-alpha-N-methylcanadine	Muscarinic acetylcholine receptor M5
Rhizoma Corydal	MOL004190	(-)-alpha-N-methylcanadine	Prostaglandin G/H synthase 2
Rhizoma Corydal	MOL004190	(-)-alpha-N-methylcanadine	Carbonic anhydrase II
Rhizoma Corydal	MOL004190	(-)-alpha-N-methylcanadine	Muscarinic acetylcholine receptor M4
Rhizoma Corydal	MOL004190	(-)-alpha-N-methylcanadine	Alpha-1B adrenergic receptor
Rhizoma Corydal	MOL004190	(-)-alpha-N-methylcanadine	Beta-2 adrenergic receptor

Rhizoma Corydal MOL004190	(-)-alpha-N-methylcanadine	Alpha-1D adrenergic receptor
Rhizoma Corydal MOL004190	(-)-alpha-N-methylcanadine	Nuclear receptor coactivator 2
Rhizoma Corydal MOL004190	(-)-alpha-N-methylcanadine	Nuclear receptor coactivator 1
Rhizoma Corydal MOL004190	(-)-alpha-N-methylcanadine	Calmodulin
Rhizoma Corydal MOL004191	Capaurine	Prostaglandin G/H synthase 1
Rhizoma Corydal MOL004191	Capaurine	Dopamine D1 receptor
Rhizoma Corydal MOL004191	Capaurine	Muscarinic acetylcholine receptor M3
Rhizoma Corydal MOL004191	Capaurine	Potassium voltage-gated channel subfamily H member 2
Rhizoma Corydal MOL004191	Capaurine	Muscarinic acetylcholine receptor M1
Rhizoma Corydal MOL004191	Capaurine	Sodium channel protein type 5 subunit alpha
Rhizoma Corydal MOL004191	Capaurine	Coagulation factor Xa
Rhizoma Corydal MOL004191	Capaurine	Muscarinic acetylcholine receptor M5
Rhizoma Corydal MOL004191	Capaurine	Prostaglandin G/H synthase 2
Rhizoma Corydal MOL004191	Capaurine	Nitric-oxide synthase, endothelial
Rhizoma Corydal MOL004191	Capaurine	Carbonic anhydrase II
Rhizoma Corydal MOL004191	Capaurine	Vascular endothelial growth factor receptor 2
Rhizoma Corydal MOL004191	Capaurine	Muscarinic acetylcholine receptor M4
Rhizoma Corydal MOL004191	Capaurine	Retinoic acid receptor RXR-alpha
Rhizoma Corydal MOL004191	Capaurine	Delta-type opioid receptor
Rhizoma Corydal MOL004191	Capaurine	5-hydroxytryptamine 2A receptor
Rhizoma Corydal MOL004191	Capaurine	5-hydroxytryptamine 2C receptor
Rhizoma Corydal MOL004191	Capaurine	Alpha-1B adrenergic receptor

Rhizoma Corydal MOL004191	Capaurine	Sodium-dependent dopamine transporter
Rhizoma Corydal MOL004191	Capaurine	Beta-2 adrenergic receptor
Rhizoma Corydal MOL004191	Capaurine	Alpha-1D adrenergic receptor
Rhizoma Corydal MOL004191	Capaurine	DNA topoisomerase II
Rhizoma Corydal MOL004191	Capaurine	Sodium-dependent serotonin transporter
Rhizoma Corydal MOL004191	Capaurine	Mu-type opioid receptor
Rhizoma Corydal MOL004191	Capaurine	Heat shock protein HSP 90
Rhizoma Corydal MOL004191	Capaurine	Retinoic acid receptor RXR-beta
Rhizoma Corydal MOL004191	Capaurine	Calcium-activated potassium channel subunit alpha 1
Rhizoma Corydal MOL004191	Capaurine	Calmodulin
Rhizoma Corydal MOL004193	Clarkeanidine	Prostaglandin G/H synthase 1
Rhizoma Corydal MOL004193	Clarkeanidine	Dopamine D1 receptor
Rhizoma Corydal MOL004193	Clarkeanidine	Muscarinic acetylcholine receptor M3
Rhizoma Corydal MOL004193	Clarkeanidine	Potassium voltage-gated channel subfamily H member 2
Rhizoma Corydal MOL004193	Clarkeanidine	Muscarinic acetylcholine receptor M1
Rhizoma Corydal MOL004193	Clarkeanidine	Sodium channel protein type 5 subunit alpha
Rhizoma Corydal MOL004193	Clarkeanidine	Coagulation factor Xa
Rhizoma Corydal MOL004193	Clarkeanidine	Muscarinic acetylcholine receptor M5
Rhizoma Corydal MOL004193	Clarkeanidine	Prostaglandin G/H synthase 2
Rhizoma Corydal MOL004193	Clarkeanidine	Carbonic anhydrase II
Rhizoma Corydal MOL004193	Clarkeanidine	Alpha-2C adrenergic receptor
Rhizoma Corydal MOL004193	Clarkeanidine	Muscarinic acetylcholine receptor M4
Rhizoma Corydal MOL004193	Clarkeanidine	Retinoic acid receptor RXR-alpha
Rhizoma Corydal MOL004193	Clarkeanidine	Delta-type opioid receptor
Rhizoma Corydal MOL004193	Clarkeanidine	5-hydroxytryptamine 2A receptor

Rhizoma Corydal	MOL004193	Clarkeanidine	Sodium-dependent noradrenaline transporter
Rhizoma Corydal	MOL004193	Clarkeanidine	Alpha-1A adrenergic receptor
Rhizoma Corydal	MOL004193	Clarkeanidine	5-hydroxytryptamine 2C receptor
Rhizoma Corydal	MOL004193	Clarkeanidine	Muscarinic acetylcholine receptor M2
Rhizoma Corydal	MOL004193	Clarkeanidine	Alpha-2B adrenergic receptor
Rhizoma Corydal	MOL004193	Clarkeanidine	Alpha-1B adrenergic receptor
Rhizoma Corydal	MOL004193	Clarkeanidine	Sodium-dependent dopamine transporter
Rhizoma Corydal	MOL004193	Clarkeanidine	Beta-2 adrenergic receptor
Rhizoma Corydal	MOL004193	Clarkeanidine	Alpha-1D adrenergic receptor
Rhizoma Corydal	MOL004193	Clarkeanidine	DNA topoisomerase II
Rhizoma Corydal	MOL004193	Clarkeanidine	Sodium-dependent serotonin transporter
Rhizoma Corydal	MOL004193	Clarkeanidine	Mu-type opioid receptor
Rhizoma Corydal	MOL004193	Clarkeanidine	Heat shock protein HSP 90
Rhizoma Corydal	MOL004193	Clarkeanidine	mRNA of PKA Catalytic Subunit C-alpha
Rhizoma Corydal	MOL004193	Clarkeanidine	Calmodulin
Rhizoma Corydal	MOL004195	CORYDALINE	Dopamine D1 receptor
Rhizoma Corydal	MOL004195	CORYDALINE	Muscarinic acetylcholine receptor M3
Rhizoma Corydal	MOL004195	CORYDALINE	Potassium voltage-gated channel subfamily H member 2
Rhizoma Corydal	MOL004195	CORYDALINE	Muscarinic acetylcholine receptor M1
Rhizoma Corydal	MOL004195	CORYDALINE	Sodium channel protein type 5 subunit alpha

Rhizoma Corydal	MOL004195	CORYDALINE	Muscarinic acetylcholine receptor M5
Rhizoma Corydal	MOL004195	CORYDALINE	Prostaglandin G/H synthase 2
Rhizoma Corydal	MOL004195	CORYDALINE	Carbonic anhydrase II
Rhizoma Corydal	MOL004195	CORYDALINE	Muscarinic acetylcholine receptor M4
Rhizoma Corydal	MOL004195	CORYDALINE	Retinoic acid receptor RXR-alpha
Rhizoma Corydal	MOL004195	CORYDALINE	Delta-type opioid receptor
Rhizoma Corydal	MOL004195	CORYDALINE	5-hydroxytryptamine 2A receptor
Rhizoma Corydal	MOL004195	CORYDALINE	5-hydroxytryptamine 2C receptor
Rhizoma Corydal	MOL004195	CORYDALINE	Alpha-2B adrenergic receptor
Rhizoma Corydal	MOL004195	CORYDALINE	Alpha-1B adrenergic receptor
Rhizoma Corydal	MOL004195	CORYDALINE	Sodium-dependent dopamine transporter
Rhizoma Corydal	MOL004195	CORYDALINE	Beta-2 adrenergic receptor
Rhizoma Corydal	MOL004195	CORYDALINE	Alpha-1D adrenergic receptor
Rhizoma Corydal	MOL004195	CORYDALINE	DNA topoisomerase II
Rhizoma Corydal	MOL004195	CORYDALINE	Sodium-dependent serotonin transporter

Rhizoma Corydal	MOL004195	CORYDALINE	D(2) dopamine receptor
Rhizoma Corydal	MOL004195	CORYDALINE	Mu-type opioid receptor
Rhizoma Corydal	MOL004195	CORYDALINE	Heat shock protein HSP 90
Rhizoma Corydal	MOL004195	CORYDALINE	Retinoic acid receptor RXR-beta
Rhizoma Corydal	MOL004195	CORYDALINE	Calmodulin
Rhizoma Corydal	MOL004196	Corydalmine	Prostaglandin G/H synthase 1
Rhizoma Corydal	MOL004196	Corydalmine	Muscarinic acetylcholine receptor M3
Rhizoma Corydal	MOL004196	Corydalmine	Potassium voltage-gated channel subfamily H member 2
Rhizoma Corydal	MOL004196	Corydalmine	Muscarinic acetylcholine receptor M1
Rhizoma Corydal	MOL004196	Corydalmine	Estrogen receptor
Rhizoma Corydal	MOL004196	Corydalmine	Sodium channel protein type 5 subunit alpha
Rhizoma Corydal	MOL004196	Corydalmine	Peroxisome proliferator activated receptor gamma
Rhizoma Corydal	MOL004196	Corydalmine	Coagulation factor Xa
Rhizoma Corydal	MOL004196	Corydalmine	Muscarinic acetylcholine receptor M5
Rhizoma Corydal	MOL004196	Corydalmine	Prostaglandin G/H synthase 2
Rhizoma Corydal	MOL004196	Corydalmine	Carbonic anhydrase II
Rhizoma Corydal	MOL004196	Corydalmine	Alpha-2C adrenergic receptor
Rhizoma Corydal	MOL004196	Corydalmine	Muscarinic acetylcholine receptor M4
Rhizoma Corydal	MOL004196	Corydalmine	Retinoic acid receptor RXR-alpha
Rhizoma Corydal	MOL004196	Corydalmine	Delta-type opioid receptor
Rhizoma Corydal	MOL004196	Corydalmine	Acetylcholinesterase
Rhizoma Corydal	MOL004196	Corydalmine	Alpha-1B adrenergic receptor

Rhizoma Corydal	MOL004196	Corydalmine	Beta-2 adrenergic receptor
Rhizoma Corydal	MOL004196	Corydalmine	Alpha-1D adrenergic receptor
Rhizoma Corydal	MOL004196	Corydalmine	Mu-type opioid receptor
Rhizoma Corydal	MOL004196	Corydalmine	Heat shock protein HSP 90
Rhizoma Corydal	MOL004196	Corydalmine	Retinoic acid receptor RXR-beta
Rhizoma Corydal	MOL004196	Corydalmine	Proto-oncogene serine/threonine-protein kinase Pim-1
Rhizoma Corydal	MOL004196	Corydalmine	Nuclear receptor coactivator 2
Rhizoma Corydal	MOL004196	Corydalmine	Nuclear receptor coactivator 1
Rhizoma Corydal	MOL004196	Corydalmine	Calmodulin
Rhizoma Corydal	MOL004197	Corydine	Prostaglandin G/H synthase 1
Rhizoma Corydal	MOL004197	Corydine	Muscarinic acetylcholine receptor M3
Rhizoma Corydal	MOL004197	Corydine	Potassium voltage-gated channel subfamily H member 2
Rhizoma Corydal	MOL004197	Corydine	Muscarinic acetylcholine receptor M1
Rhizoma Corydal	MOL004197	Corydine	Androgen receptor
Rhizoma Corydal	MOL004197	Corydine	Sodium channel protein type 5 subunit alpha
Rhizoma Corydal	MOL004197	Corydine	Coagulation factor Xa
Rhizoma Corydal	MOL004197	Corydine	Muscarinic acetylcholine receptor M5
Rhizoma Corydal	MOL004197	Corydine	Prostaglandin G/H synthase 2
Rhizoma Corydal	MOL004197	Corydine	Retinoic acid receptor RXR-alpha
Rhizoma Corydal	MOL004197	Corydine	Delta-type opioid receptor
Rhizoma Corydal	MOL004197	Corydine	Acetylcholinesterase
Rhizoma Corydal	MOL004197	Corydine	Alpha-1B adrenergic receptor
Rhizoma Corydal	MOL004197	Corydine	Beta-2 adrenergic receptor
Rhizoma Corydal	MOL004197	Corydine	Alpha-1D adrenergic receptor
Rhizoma Corydal	MOL004197	Corydine	DNA topoisomerase II
Rhizoma Corydal	MOL004197	Corydine	Sodium-dependent serotonin transporter

Rhizoma Corydal	MOL004197	Corydine	Mu-type opioid receptor
Rhizoma Corydal	MOL004197	Corydine	Pregnane X receptor
Rhizoma Corydal	MOL004197	Corydine	Heat shock protein HSP 90
Rhizoma Corydal	MOL004197	Corydine	Calcium-activated potassium channel subunit alpha 1
Rhizoma Corydal	MOL004198	18797-79-0	Prostaglandin G/H synthase 1
Rhizoma Corydal	MOL004198	18797-79-0	Muscarinic acetylcholine receptor M3
Rhizoma Corydal	MOL004198	18797-79-0	Muscarinic acetylcholine receptor M1
Rhizoma Corydal	MOL004198	18797-79-0	Sodium channel protein type 5 subunit alpha
Rhizoma Corydal	MOL004198	18797-79-0	Muscarinic acetylcholine receptor M5
Rhizoma Corydal	MOL004198	18797-79-0	Prostaglandin G/H synthase 2
Rhizoma Corydal	MOL004198	18797-79-0	Carbonic anhydrase II
Rhizoma Corydal	MOL004198	18797-79-0	Delta-type opioid receptor
Rhizoma Corydal	MOL004198	18797-79-0	Alpha-1B adrenergic receptor
Rhizoma Corydal	MOL004198	18797-79-0	DNA topoisomerase II
Rhizoma Corydal	MOL004198	18797-79-0	Mu-type opioid receptor
Rhizoma Corydal	MOL004198	18797-79-0	Calmodulin
Rhizoma Corydal	MOL004199	Corynoloxine	Prostaglandin G/H synthase 1
Rhizoma Corydal	MOL004199	Corynoloxine	Androgen receptor
Rhizoma Corydal	MOL004199	Corynoloxine	Sodium channel protein type 5 subunit alpha
Rhizoma Corydal	MOL004199	Corynoloxine	Prostaglandin G/H synthase 2
Rhizoma Corydal	MOL004199	Corynoloxine	Delta-type opioid receptor
Rhizoma Corydal	MOL004199	Corynoloxine	Acetylcholinesterase
Rhizoma Corydal	MOL004199	Corynoloxine	DNA topoisomerase II
Rhizoma Corydal	MOL004199	Corynoloxine	Mu-type opioid receptor

Rhizoma Corydal	MOL004200	methyl-[2-(3,4,6,7-tetramethoxy-1-phenanthryl)ethyl]amine	Prostaglandin G/H synthase 1
Rhizoma Corydal	MOL004200	methyl-[2-(3,4,6,7-tetramethoxy-1-phenanthryl)ethyl]amine	Potassium voltage-gated channel subfamily H member 2
Rhizoma Corydal	MOL004200	methyl-[2-(3,4,6,7-tetramethoxy-1-phenanthryl)ethyl]amine	Sodium channel protein type 5 subunit alpha
Rhizoma Corydal	MOL004200	methyl-[2-(3,4,6,7-tetramethoxy-1-phenanthryl)ethyl]amine	Prostaglandin G/H synthase 2
Rhizoma Corydal	MOL004200	methyl-[2-(3,4,6,7-tetramethoxy-1-phenanthryl)ethyl]amine	Nitric-oxide synthase, endothelial
Rhizoma Corydal	MOL004200	methyl-[2-(3,4,6,7-tetramethoxy-1-phenanthryl)ethyl]amine	Retinoic acid receptor RXR-alpha

Rhizoma Corydal	MOL004200	methyl-[2-(3,4,6,7-tetramethoxy-1-phenanthryl)ethyl]amine	DNA topoisomerase II
Rhizoma Corydal	MOL004200	methyl-[2-(3,4,6,7-tetramethoxy-1-phenanthryl)ethyl]amine	Pregnane X receptor
Rhizoma Corydal	MOL004200	methyl-[2-(3,4,6,7-tetramethoxy-1-phenanthryl)ethyl]amine	Heat shock protein HSP 90
Rhizoma Corydal	MOL004200	methyl-[2-(3,4,6,7-tetramethoxy-1-phenanthryl)ethyl]amine	Nuclear receptor coactivator 2
Rhizoma Corydal	MOL004200	methyl-[2-(3,4,6,7-tetramethoxy-1-phenanthryl)ethyl]amine	Calmodulin
Rhizoma Corydal	MOL004202	dehydrocavidine	Prostaglandin G/H synthase 1
Rhizoma Corydal	MOL004202	dehydrocavidine	Dopamine D1 receptor

Rhizoma Corydal	MOL004202	dehydrocavidine	Muscarinic acetylcholine receptor M3
Rhizoma Corydal	MOL004202	dehydrocavidine	Potassium voltage-gated channel subfamily H member 2
Rhizoma Corydal	MOL004202	dehydrocavidine	Muscarinic acetylcholine receptor M1
Rhizoma Corydal	MOL004202	dehydrocavidine	Sodium channel protein type 5 subunit alpha
Rhizoma Corydal	MOL004202	dehydrocavidine	Coagulation factor Xa
Rhizoma Corydal	MOL004202	dehydrocavidine	Muscarinic acetylcholine receptor M5
Rhizoma Corydal	MOL004202	dehydrocavidine	Prostaglandin G/H synthase 2
Rhizoma Corydal	MOL004202	dehydrocavidine	Coagulation factor VII
Rhizoma Corydal	MOL004202	dehydrocavidine	Muscarinic acetylcholine receptor M4
Rhizoma Corydal	MOL004202	dehydrocavidine	Retinoic acid receptor RXR-alpha
Rhizoma Corydal	MOL004202	dehydrocavidine	Delta-type opioid receptor
Rhizoma Corydal	MOL004202	dehydrocavidine	5-hydroxytryptamine 2A receptor
Rhizoma Corydal	MOL004202	dehydrocavidine	5-hydroxytryptamine 2C receptor
Rhizoma Corydal	MOL004202	dehydrocavidine	Muscarinic acetylcholine receptor M2
Rhizoma Corydal	MOL004202	dehydrocavidine	Alpha-1B adrenergic receptor

Rhizoma Corydal	MOL004202	dehydrocavidine	Beta-2 adrenergic receptor
Rhizoma Corydal	MOL004202	dehydrocavidine	Alpha-1D adrenergic receptor
Rhizoma Corydal	MOL004202	dehydrocavidine	DNA topoisomerase II
Rhizoma Corydal	MOL004202	dehydrocavidine	Mu-type opioid receptor
Rhizoma Corydal	MOL004202	dehydrocavidine	Heat shock protein HSP 90
Rhizoma Corydal	MOL004202	dehydrocavidine	Calmodulin
Rhizoma Corydal	MOL004203	Dehydrocorybulbine	Nitric oxide synthase, inducible
Rhizoma Corydal	MOL004203	Dehydrocorybulbine	Prostaglandin G/H synthase 1
Rhizoma Corydal	MOL004203	Dehydrocorybulbine	Potassium voltage-gated channel subfamily H member 2
Rhizoma Corydal	MOL004203	Dehydrocorybulbine	Estrogen receptor
Rhizoma Corydal	MOL004203	Dehydrocorybulbine	Androgen receptor
Rhizoma Corydal	MOL004203	Dehydrocorybulbine	Sodium channel protein type 5 subunit alpha
Rhizoma Corydal	MOL004203	Dehydrocorybulbine	Prostaglandin G/H synthase 2
Rhizoma Corydal	MOL004203	Dehydrocorybulbine	Retinoic acid receptor RXR-alpha
Rhizoma Corydal	MOL004203	Dehydrocorybulbine	Mitogen-activated protein kinase 14

Rhizoma Corydal	MOL004203	Dehydrocorybulbine	Serine/threonine-protein kinase Chk1
Rhizoma Corydal	MOL004203	Dehydrocorybulbine	Trypsin-1
Rhizoma Corydal	MOL004203	Dehydrocorybulbine	Proto-oncogene serine/threonine-protein kinase Pim-1
Rhizoma Corydal	MOL004203	Dehydrocorybulbine	Nuclear receptor coactivator 2
Rhizoma Corydal	MOL004203	Dehydrocorybulbine	Calmodulin
Rhizoma Corydal	MOL004204	dehydrocorydaline	Nitric oxide synthase, inducible
Rhizoma Corydal	MOL004204	dehydrocorydaline	Prostaglandin G/H synthase 1
Rhizoma Corydal	MOL004204	dehydrocorydaline	Potassium voltage-gated channel subfamily H member 2
Rhizoma Corydal	MOL004204	dehydrocorydaline	Estrogen receptor
Rhizoma Corydal	MOL004204	dehydrocorydaline	Androgen receptor
Rhizoma Corydal	MOL004204	dehydrocorydaline	Sodium channel protein type 5 subunit alpha
Rhizoma Corydal	MOL004204	dehydrocorydaline	Prostaglandin G/H synthase 2
Rhizoma Corydal	MOL004204	dehydrocorydaline	Retinoic acid receptor RXR-alpha
Rhizoma Corydal	MOL004204	dehydrocorydaline	DNA topoisomerase II
Rhizoma Corydal	MOL004204	dehydrocorydaline	Mitogen-activated protein kinase 14

Rhizoma Corydal	MOL004204	dehydrocorydaline	Serine/threonine-protein kinase Chk1
Rhizoma Corydal	MOL004204	dehydrocorydaline	Retinoic acid receptor RXR-beta
Rhizoma Corydal	MOL004204	dehydrocorydaline	Trypsin-1
Rhizoma Corydal	MOL004204	dehydrocorydaline	Proto-oncogene serine/threonine-protein kinase Pim-1
Rhizoma Corydal	MOL004204	dehydrocorydaline	Nuclear receptor coactivator 2
Rhizoma Corydal	MOL004204	dehydrocorydaline	cAMP and cAMP-inhibited cGMP 3',5'-cyclic phosphodiesterase 10A
Rhizoma Corydal	MOL004204	dehydrocorydaline	Calmodulin
Rhizoma Corydal	MOL004205	Dehydrocorydalmine	Nitric oxide synthase, inducible
Rhizoma Corydal	MOL004205	Dehydrocorydalmine	Prostaglandin G/H synthase 1
Rhizoma Corydal	MOL004205	Dehydrocorydalmine	Potassium voltage-gated channel subfamily H member 2
Rhizoma Corydal	MOL004205	Dehydrocorydalmine	Estrogen receptor
Rhizoma Corydal	MOL004205	Dehydrocorydalmine	Androgen receptor
Rhizoma Corydal	MOL004205	Dehydrocorydalmine	Sodium channel protein type 5 subunit alpha
Rhizoma Corydal	MOL004205	Dehydrocorydalmine	Prostaglandin G/H synthase 2
Rhizoma Corydal	MOL004205	Dehydrocorydalmine	Nitric-oxide synthase, endothelial

Rhizoma Corydal	MOL004205	Dehydrocorydalmine	Coagulation factor VII
Rhizoma Corydal	MOL004205	Dehydrocorydalmine	Retinoic acid receptor RXR-alpha
Rhizoma Corydal	MOL004205	Dehydrocorydalmine	Beta-2 adrenergic receptor
Rhizoma Corydal	MOL004205	Dehydrocorydalmine	Estrogen receptor beta
Rhizoma Corydal	MOL004205	Dehydrocorydalmine	Heat shock protein HSP 90
Rhizoma Corydal	MOL004205	Dehydrocorydalmine	Cell division protein kinase 2
Rhizoma Corydal	MOL004205	Dehydrocorydalmine	Trypsin-1
Rhizoma Corydal	MOL004205	Dehydrocorydalmine	Proto-oncogene serine/threonine-protein kinase Pim-1
Rhizoma Corydal	MOL004205	Dehydrocorydalmine	Cyclin-A2
Rhizoma Corydal	MOL004205	Dehydrocorydalmine	Nuclear receptor coactivator 2
Rhizoma Corydal	MOL004205	Dehydrocorydalmine	Calcium-activated potassium channel subunit alpha 1
Rhizoma Corydal	MOL004205	Dehydrocorydalmine	Calmodulin
Rhizoma Corydal	MOL004208	demethylcorydalmatine	Prostaglandin G/H synthase 1
Rhizoma Corydal	MOL004208	demethylcorydalmatine	Dopamine D1 receptor
Rhizoma Corydal	MOL004208	demethylcorydalmatine	Muscarinic acetylcholine receptor M3

Rhizoma Corydal	MOL004208	demethylcorydalmatine	Potassium voltage-gated channel subfamily H member 2
Rhizoma Corydal	MOL004208	demethylcorydalmatine	Muscarinic acetylcholine receptor M1
Rhizoma Corydal	MOL004208	demethylcorydalmatine	Sodium channel protein type 5 subunit alpha
Rhizoma Corydal	MOL004208	demethylcorydalmatine	Muscarinic acetylcholine receptor M5
Rhizoma Corydal	MOL004208	demethylcorydalmatine	Prostaglandin G/H synthase 2
Rhizoma Corydal	MOL004208	demethylcorydalmatine	Alpha-2C adrenergic receptor
Rhizoma Corydal	MOL004208	demethylcorydalmatine	Muscarinic acetylcholine receptor M4
Rhizoma Corydal	MOL004208	demethylcorydalmatine	Delta-type opioid receptor
Rhizoma Corydal	MOL004208	demethylcorydalmatine	Alpha-1B adrenergic receptor
Rhizoma Corydal	MOL004208	demethylcorydalmatine	Sodium-dependent dopamine transporter
Rhizoma Corydal	MOL004208	demethylcorydalmatine	Beta-2 adrenergic receptor
Rhizoma Corydal	MOL004208	demethylcorydalmatine	Alpha-1D adrenergic receptor
Rhizoma Corydal	MOL004208	demethylcorydalmatine	Sodium-dependent serotonin transporter
Rhizoma Corydal	MOL004208	demethylcorydalmatine	Mu-type opioid receptor
Rhizoma Corydal	MOL004208	demethylcorydalmatine	Heat shock protein HSP 90

Rhizoma Corydal	MOL004208	demethylcorydalmatine	Calmodulin
Rhizoma Corydal	MOL004209	13-methyldehydrocorydalmine	Nitric oxide synthase, inducible
Rhizoma Corydal	MOL004209	13-methyldehydrocorydalmine	Prostaglandin G/H synthase 1
Rhizoma Corydal	MOL004209	13-methyldehydrocorydalmine	Potassium voltage-gated channel subfamily H member 2
Rhizoma Corydal	MOL004209	13-methyldehydrocorydalmine	Estrogen receptor
Rhizoma Corydal	MOL004209	13-methyldehydrocorydalmine	Androgen receptor
Rhizoma Corydal	MOL004209	13-methyldehydrocorydalmine	Prostaglandin G/H synthase 2
Rhizoma Corydal	MOL004209	13-methyldehydrocorydalmine	Nitric-oxide synthase, endothelial
Rhizoma Corydal	MOL004209	13-methyldehydrocorydalmine	Retinoic acid receptor RXR-alpha
Rhizoma Corydal	MOL004209	13-methyldehydrocorydalmine	Mitogen-activated protein kinase 14

Rhizoma Corydal	MOL004209	13-methyldehydrocorydalmine	Serine/threonine-protein kinase Chk1
Rhizoma Corydal	MOL004209	13-methyldehydrocorydalmine	Trypsin-1
Rhizoma Corydal	MOL004209	13-methyldehydrocorydalmine	Proto-oncogene serine/threonine-protein kinase Pim-1
Rhizoma Corydal	MOL004209	13-methyldehydrocorydalmine	Nuclear receptor coactivator 2
Rhizoma Corydal	MOL004209	13-methyldehydrocorydalmine	Calmodulin
Rhizoma Corydal	MOL004210	(1S,8'R)-6,7-dimethoxy-2-methylspiro[3,4-dihydroisoquinoline-1,7'-6,8-dihydrocyclopenta[g][1,3]benzodioxole]-8'-ol	Prostaglandin G/H synthase 1

Rhizoma Corydal	MOL004210	(1S,8'R)-6,7-dimethoxy-2-methylspiro[3,4-dihydroisoquinoline-1,7'-6,8-dihydrocyclopenta[g][1,3]benzodioxole]-8'-ol	Muscarinic acetylcholine receptor M3
Rhizoma Corydal	MOL004210	(1S,8'R)-6,7-dimethoxy-2-methylspiro[3,4-dihydroisoquinoline-1,7'-6,8-dihydrocyclopenta[g][1,3]benzodioxole]-8'-ol	Sodium channel protein type 5 subunit alpha
Rhizoma Corydal	MOL004210	(1S,8'R)-6,7-dimethoxy-2-methylspiro[3,4-dihydroisoquinoline-1,7'-6,8-dihydrocyclopenta[g][1,3]benzodioxole]-8'-ol	Coagulation factor Xa

Rhizoma Corydal	MOL004210	(1S,8'R)-6,7-dimethoxy-2-methylspiro[3,4-dihydroisoquinoline-1,7'-6,8-dihydrocyclopenta[g][1,3]benzodioxole]-8'-ol	Muscarinic acetylcholine receptor M5
Rhizoma Corydal	MOL004210	(1S,8'R)-6,7-dimethoxy-2-methylspiro[3,4-dihydroisoquinoline-1,7'-6,8-dihydrocyclopenta[g][1,3]benzodioxole]-8'-ol	Prostaglandin G/H synthase 2
Rhizoma Corydal	MOL004210	(1S,8'R)-6,7-dimethoxy-2-methylspiro[3,4-dihydroisoquinoline-1,7'-6,8-dihydrocyclopenta[g][1,3]benzodioxole]-8'-ol	Delta-type opioid receptor

Rhizoma Corydal	MOL004210	(1S,8'R)-6,7-dimethoxy-2-methylspiro[3,4-dihydroisoquinoline-1,7'-6,8-dihydrocyclopenta[g][1,3]benzodioxole]-8'-ol	Alpha-1B adrenergic receptor
Rhizoma Corydal	MOL004210	(1S,8'R)-6,7-dimethoxy-2-methylspiro[3,4-dihydroisoquinoline-1,7'-6,8-dihydrocyclopenta[g][1,3]benzodioxole]-8'-ol	Alpha-1D adrenergic receptor
Rhizoma Corydal	MOL004210	(1S,8'R)-6,7-dimethoxy-2-methylspiro[3,4-dihydroisoquinoline-1,7'-6,8-dihydrocyclopenta[g][1,3]benzodioxole]-8'-ol	DNA topoisomerase II

Rhizoma Corydal	MOL004210	(1S,8'R)-6,7-dimethoxy-2-methylspiro[3,4-dihydroisoquinoline-1,7'-6,8-dihydrocyclopenta[g][1,3]benzodioxole]-8'-ol	Mu-type opioid receptor
Rhizoma Corydal	MOL004210	(1S,8'R)-6,7-dimethoxy-2-methylspiro[3,4-dihydroisoquinoline-1,7'-6,8-dihydrocyclopenta[g][1,3]benzodioxole]-8'-ol	Nuclear receptor coactivator 1
Rhizoma Corydal	MOL004210	(1S,8'R)-6,7-dimethoxy-2-methylspiro[3,4-dihydroisoquinoline-1,7'-6,8-dihydrocyclopenta[g][1,3]benzodioxole]-8'-ol	Calmodulin
Rhizoma Corydal	MOL004763	Izoteolin	Prostaglandin G/H synthase 1
Rhizoma Corydal	MOL004763	Izoteolin	Muscarinic acetylcholine receptor M3
Rhizoma Corydal	MOL004763	Izoteolin	Muscarinic acetylcholine receptor M1

Rhizoma Corydal MOL004763	Izoteolin	Androgen receptor
Rhizoma Corydal MOL004763	Izoteolin	Sodium channel protein type 5 subunit alpha
Rhizoma Corydal MOL004763	Izoteolin	Muscarinic acetylcholine receptor M5
Rhizoma Corydal MOL004763	Izoteolin	Prostaglandin G/H synthase 2
Rhizoma Corydal MOL004763	Izoteolin	Muscarinic acetylcholine receptor M4
Rhizoma Corydal MOL004763	Izoteolin	Retinoic acid receptor RXR-alpha
Rhizoma Corydal MOL004763	Izoteolin	Delta-type opioid receptor
Rhizoma Corydal MOL004763	Izoteolin	Acetylcholinesterase
Rhizoma Corydal MOL004763	Izoteolin	Alpha-1B adrenergic receptor
Rhizoma Corydal MOL004763	Izoteolin	Sodium-dependent dopamine transporter
Rhizoma Corydal MOL004763	Izoteolin	Beta-2 adrenergic receptor
Rhizoma Corydal MOL004763	Izoteolin	Alpha-1D adrenergic receptor
Rhizoma Corydal MOL004763	Izoteolin	DNA topoisomerase II
Rhizoma Corydal MOL004763	Izoteolin	Sodium-dependent serotonin transporter
Rhizoma Corydal MOL004763	Izoteolin	Mu-type opioid receptor
Rhizoma Corydal MOL004763	Izoteolin	Heat shock protein HSP 90
Rhizoma Corydal MOL004763	Izoteolin	Nitric-oxide synthase, endothelial
Rhizoma Corydal MOL004763	Izoteolin	Alpha-1A adrenergic receptor
Rhizoma Corydal MOL004214	isocorybulbine	Prostaglandin G/H synthase 1
Rhizoma Corydal MOL004214	isocorybulbine	Muscarinic acetylcholine receptor M3
Rhizoma Corydal MOL004214	isocorybulbine	Potassium voltage-gated channel subfamily H member 2
Rhizoma Corydal MOL004214	isocorybulbine	Estrogen receptor
Rhizoma Corydal MOL004214	isocorybulbine	Sodium channel protein type 5 subunit alpha
Rhizoma Corydal MOL004214	isocorybulbine	Prostaglandin G/H synthase 2
Rhizoma Corydal MOL004214	isocorybulbine	Carbonic anhydrase II

Rhizoma Corydal	MOL004214	isocorybulbine	Retinoic acid receptor RXR-alpha
Rhizoma Corydal	MOL004214	isocorybulbine	Delta-type opioid receptor
Rhizoma Corydal	MOL004214	isocorybulbine	Acetylcholinesterase
Rhizoma Corydal	MOL004214	isocorybulbine	Alpha-1B adrenergic receptor
Rhizoma Corydal	MOL004214	isocorybulbine	Beta-2 adrenergic receptor
Rhizoma Corydal	MOL004214	isocorybulbine	Alpha-1D adrenergic receptor
Rhizoma Corydal	MOL004214	isocorybulbine	DNA topoisomerase II
Rhizoma Corydal	MOL004214	isocorybulbine	Mu-type opioid receptor
Rhizoma Corydal	MOL004214	isocorybulbine	Retinoic acid receptor RXR-beta
Rhizoma Corydal	MOL004214	isocorybulbine	Proto-oncogene serine/threonine-protein kinase Pim-1
Rhizoma Corydal	MOL004214	isocorybulbine	Nuclear receptor coactivator 2
Rhizoma Corydal	MOL004214	isocorybulbine	Nuclear receptor coactivator 1
Rhizoma Corydal	MOL004214	isocorybulbine	Calmodulin
Rhizoma Corydal	MOL004215	leonticine	Prostaglandin G/H synthase 1
Rhizoma Corydal	MOL004215	leonticine	Dopamine D1 receptor
Rhizoma Corydal	MOL004215	leonticine	Muscarinic acetylcholine receptor M3
Rhizoma Corydal	MOL004215	leonticine	Potassium voltage-gated channel subfamily H member 2
Rhizoma Corydal	MOL004215	leonticine	Muscarinic acetylcholine receptor M1
Rhizoma Corydal	MOL004215	leonticine	D(1B) dopamine receptor
Rhizoma Corydal	MOL004215	leonticine	Beta-1 adrenergic receptor
Rhizoma Corydal	MOL004215	leonticine	Sodium channel protein type 5 subunit alpha
Rhizoma Corydal	MOL004215	leonticine	Muscarinic acetylcholine receptor M5
Rhizoma Corydal	MOL004215	leonticine	Prostaglandin G/H synthase 2
Rhizoma Corydal	MOL004215	leonticine	Alpha-2A adrenergic receptor
Rhizoma Corydal	MOL004215	leonticine	Alpha-2C adrenergic receptor
Rhizoma Corydal	MOL004215	leonticine	Muscarinic acetylcholine receptor M4
Rhizoma Corydal	MOL004215	leonticine	Retinoic acid receptor RXR-alpha

Rhizoma Corydal	MOL004215	leonticine	Delta-type opioid receptor
Rhizoma Corydal	MOL004215	leonticine	CGMP-inhibited 3',5'-cyclic phosphodiesterase A
Rhizoma Corydal	MOL004215	leonticine	5-hydroxytryptamine 2A receptor
Rhizoma Corydal	MOL004215	leonticine	Sodium-dependent noradrenaline transporter
Rhizoma Corydal	MOL004215	leonticine	Alpha-1A adrenergic receptor
Rhizoma Corydal	MOL004215	leonticine	5-hydroxytryptamine 2C receptor
Rhizoma Corydal	MOL004215	leonticine	Muscarinic acetylcholine receptor M2
Rhizoma Corydal	MOL004215	leonticine	Alpha-2B adrenergic receptor
Rhizoma Corydal	MOL004215	leonticine	Alpha-1B adrenergic receptor
Rhizoma Corydal	MOL004215	leonticine	Sodium-dependent dopamine transporter
Rhizoma Corydal	MOL004215	leonticine	Beta-2 adrenergic receptor
Rhizoma Corydal	MOL004215	leonticine	Alpha-1D adrenergic receptor
Rhizoma Corydal	MOL004215	leonticine	Sodium-dependent serotonin transporter
Rhizoma Corydal	MOL004215	leonticine	D(2) dopamine receptor
Rhizoma Corydal	MOL004215	leonticine	Mu-type opioid receptor
Rhizoma Corydal	MOL004215	leonticine	Gamma-aminobutyric acid receptor subunit alpha-1
Rhizoma Corydal	MOL004215	leonticine	5-hydroxytryptamine 1B receptor
Rhizoma Corydal	MOL004215	leonticine	Beta-secretase
Rhizoma Corydal	MOL004215	leonticine	Heat shock protein HSP 90
Rhizoma Corydal	MOL004215	leonticine	mRNA of PKA Catalytic Subunit C-alpha
Rhizoma Corydal	MOL004215	leonticine	Calmodulin
Rhizoma Corydal	MOL004216	13-methylpalmatrubine	Nitric oxide synthase, inducible

Rhizoma Corydal	MOL004216	13-methylpalmatrubine	Prostaglandin G/H synthase 1
Rhizoma Corydal	MOL004216	13-methylpalmatrubine	Potassium voltage-gated channel subfamily H member 2
Rhizoma Corydal	MOL004216	13-methylpalmatrubine	Estrogen receptor
Rhizoma Corydal	MOL004216	13-methylpalmatrubine	Androgen receptor
Rhizoma Corydal	MOL004216	13-methylpalmatrubine	Sodium channel protein type 5 subunit alpha
Rhizoma Corydal	MOL004216	13-methylpalmatrubine	Prostaglandin G/H synthase 2
Rhizoma Corydal	MOL004216	13-methylpalmatrubine	Nitric-oxide synthase, endothelial
Rhizoma Corydal	MOL004216	13-methylpalmatrubine	Retinoic acid receptor RXR-alpha
Rhizoma Corydal	MOL004216	13-methylpalmatrubine	Mitogen-activated protein kinase 14
Rhizoma Corydal	MOL004216	13-methylpalmatrubine	Heat shock protein HSP 90

Rhizoma Corydal	MOL004216	13-methylpalmatrubine	Serine/threonine-protein kinase Chk1
Rhizoma Corydal	MOL004216	13-methylpalmatrubine	Trypsin-1
Rhizoma Corydal	MOL004216	13-methylpalmatrubine	Proto-oncogene serine/threonine-protein kinase Pim-1
Rhizoma Corydal	MOL004216	13-methylpalmatrubine	Nuclear receptor coactivator 2
Rhizoma Corydal	MOL004216	13-methylpalmatrubine	Calmodulin
Rhizoma Corydal	MOL004220	N-methyllaurotetanine	Prostaglandin G/H synthase 1
Rhizoma Corydal	MOL004220	N-methyllaurotetanine	Muscarinic acetylcholine receptor M3
Rhizoma Corydal	MOL004220	N-methyllaurotetanine	Muscarinic acetylcholine receptor M1
Rhizoma Corydal	MOL004220	N-methyllaurotetanine	Androgen receptor
Rhizoma Corydal	MOL004220	N-methyllaurotetanine	Sodium channel protein type 5 subunit alpha

Rhizoma Corydal	MOL004220	N-methyllaurotetanine	Muscarinic acetylcholine receptor M5
Rhizoma Corydal	MOL004220	N-methyllaurotetanine	Prostaglandin G/H synthase 2
Rhizoma Corydal	MOL004220	N-methyllaurotetanine	Nitric-oxide synthase, endothelial
Rhizoma Corydal	MOL004220	N-methyllaurotetanine	Retinoic acid receptor RXR-alpha
Rhizoma Corydal	MOL004220	N-methyllaurotetanine	Delta-type opioid receptor
Rhizoma Corydal	MOL004220	N-methyllaurotetanine	Alpha-1B adrenergic receptor
Rhizoma Corydal	MOL004220	N-methyllaurotetanine	Beta-2 adrenergic receptor
Rhizoma Corydal	MOL004220	N-methyllaurotetanine	Alpha-1D adrenergic receptor
Rhizoma Corydal	MOL004220	N-methyllaurotetanine	DNA topoisomerase II
Rhizoma Corydal	MOL004220	N-methyllaurotetanine	Mu-type opioid receptor

Rhizoma Corydal	MOL004220	N-methyllauroteta nine	Heat shock protein HSP 90
Rhizoma Corydal	MOL004221	norglaucing	Prostaglandin G/H synthase 1
Rhizoma Corydal	MOL004221	norglaucing	Muscarinic acetylcholine receptor M3
Rhizoma Corydal	MOL004221	norglaucing	Potassium voltage-gated channel subfamily H member 2
Rhizoma Corydal	MOL004221	norglaucing	Muscarinic acetylcholine receptor M1
Rhizoma Corydal	MOL004221	norglaucing	Androgen receptor
Rhizoma Corydal	MOL004221	norglaucing	Sodium channel protein type 5 subunit alpha
Rhizoma Corydal	MOL004221	norglaucing	Coagulation factor Xa
Rhizoma Corydal	MOL004221	norglaucing	Muscarinic acetylcholine receptor M5
Rhizoma Corydal	MOL004221	norglaucing	Prostaglandin G/H synthase 2
Rhizoma Corydal	MOL004221	norglaucing	Nitric-oxide synthase, endothelial
Rhizoma Corydal	MOL004221	norglaucing	Retinoic acid receptor RXR-alpha
Rhizoma Corydal	MOL004221	norglaucing	Delta-type opioid receptor
Rhizoma Corydal	MOL004221	norglaucing	Acetylcholinesterase
Rhizoma Corydal	MOL004221	norglaucing	Alpha-1B adrenergic receptor
Rhizoma Corydal	MOL004221	norglaucing	Beta-2 adrenergic receptor
Rhizoma Corydal	MOL004221	norglaucing	Alpha-1D adrenergic receptor
Rhizoma Corydal	MOL004221	norglaucing	DNA topoisomerase II
Rhizoma Corydal	MOL004221	norglaucing	Mu-type opioid receptor
Rhizoma Corydal	MOL004221	norglaucing	Heat shock protein HSP 90
Rhizoma Corydal	MOL004221	norglaucing	Retinoic acid receptor RXR-beta
Rhizoma Corydal	MOL004221	norglaucing	Nuclear receptor coactivator 1
Rhizoma Corydal	MOL004224	pontevedrine	Prostaglandin G/H synthase 1
Rhizoma Corydal	MOL004224	pontevedrine	Potassium voltage-gated channel subfamily H member 2
Rhizoma Corydal	MOL004224	pontevedrine	Coagulation factor Xa

Rhizoma Corydal	MOL004224	pontevedrine	Prostaglandin G/H synthase 2
Rhizoma Corydal	MOL004224	pontevedrine	DNA topoisomerase II
Rhizoma Corydal	MOL004224	pontevedrine	Nuclear receptor coactivator 2
Rhizoma Corydal	MOL004225	pseudocoptisine	Nitric oxide synthase, inducible
Rhizoma Corydal	MOL004225	pseudocoptisine	Prostaglandin G/H synthase 1
Rhizoma Corydal	MOL004225	pseudocoptisine	Estrogen receptor
Rhizoma Corydal	MOL004225	pseudocoptisine	Androgen receptor
Rhizoma Corydal	MOL004225	pseudocoptisine	Prostaglandin G/H synthase 2
Rhizoma Corydal	MOL004225	pseudocoptisine	Trypsin-1
Rhizoma Corydal	MOL004226	24240-05-9	Prostaglandin G/H synthase 1
Rhizoma Corydal	MOL004226	24240-05-9	Muscarinic acetylcholine receptor M3
Rhizoma Corydal	MOL004226	24240-05-9	Potassium voltage-gated channel subfamily H member 2
Rhizoma Corydal	MOL004226	24240-05-9	Muscarinic acetylcholine receptor M1
Rhizoma Corydal	MOL004226	24240-05-9	Sodium channel protein type 5 subunit alpha
Rhizoma Corydal	MOL004226	24240-05-9	Coagulation factor Xa
Rhizoma Corydal	MOL004226	24240-05-9	Muscarinic acetylcholine receptor M5
Rhizoma Corydal	MOL004226	24240-05-9	Prostaglandin G/H synthase 2
Rhizoma Corydal	MOL004226	24240-05-9	5-hydroxytryptamine receptor 3A
Rhizoma Corydal	MOL004226	24240-05-9	Coagulation factor VII
Rhizoma Corydal	MOL004226	24240-05-9	Delta-type opioid receptor
Rhizoma Corydal	MOL004226	24240-05-9	CGMP-inhibited 3',5'-cyclic phosphodiesterase A
Rhizoma Corydal	MOL004226	24240-05-9	Alpha-1B adrenergic receptor
Rhizoma Corydal	MOL004226	24240-05-9	Alpha-1D adrenergic receptor
Rhizoma Corydal	MOL004226	24240-05-9	Mu-type opioid receptor
Rhizoma Corydal	MOL004226	24240-05-9	Heat shock protein HSP 90
Rhizoma Corydal	MOL004226	24240-05-9	mRNA of PKA Catalytic Subunit C-alpha

Rhizoma Corydal MOL004226	24240-05-9	Calmodulin
Rhizoma Corydal MOL004228	saulatine	Muscarinic acetylcholine receptor M3
Rhizoma Corydal MOL004228	saulatine	Potassium voltage-gated channel subfamily H member 2
Rhizoma Corydal MOL004228	saulatine	Sodium channel protein type 5 subunit alpha
Rhizoma Corydal MOL004228	saulatine	Coagulation factor Xa
Rhizoma Corydal MOL004228	saulatine	Muscarinic acetylcholine receptor M5
Rhizoma Corydal MOL004228	saulatine	Prostaglandin G/H synthase 2
Rhizoma Corydal MOL004228	saulatine	Alpha-1B adrenergic receptor
Rhizoma Corydal MOL004230	stylopine	Prostaglandin G/H synthase 1
Rhizoma Corydal MOL004230	stylopine	Muscarinic acetylcholine receptor M3
Rhizoma Corydal MOL004230	stylopine	Muscarinic acetylcholine receptor M1
Rhizoma Corydal MOL004230	stylopine	Sodium channel protein type 5 subunit alpha
Rhizoma Corydal MOL004230	stylopine	Muscarinic acetylcholine receptor M5
Rhizoma Corydal MOL004230	stylopine	Prostaglandin G/H synthase 2
Rhizoma Corydal MOL004230	stylopine	5-hydroxytryptamine receptor 3A
Rhizoma Corydal MOL004230	stylopine	Muscarinic acetylcholine receptor M4
Rhizoma Corydal MOL004230	stylopine	Delta-type opioid receptor
Rhizoma Corydal MOL004230	stylopine	5-hydroxytryptamine 2A receptor
Rhizoma Corydal MOL004230	stylopine	Alpha-1B adrenergic receptor
Rhizoma Corydal MOL004230	stylopine	Sodium-dependent dopamine transporter
Rhizoma Corydal MOL004230	stylopine	Beta-2 adrenergic receptor
Rhizoma Corydal MOL004230	stylopine	Alpha-1D adrenergic receptor
Rhizoma Corydal MOL004230	stylopine	Mu-type opioid receptor
Rhizoma Corydal MOL004230	stylopine	Calmodulin
Rhizoma Corydal MOL004230	stylopine	Retinoic acid receptor RXR-alpha

Rhizoma Corydal	MOL004231	Tetrahydrocory samine	Prostaglandin G/H synthase 1
Rhizoma Corydal	MOL004231	Tetrahydrocory samine	Muscarinic acetylcholine receptor M3
Rhizoma Corydal	MOL004231	Tetrahydrocory samine	Potassium voltage-gated channel subfamily H member 2
Rhizoma Corydal	MOL004231	Tetrahydrocory samine	Muscarinic acetylcholine receptor M1
Rhizoma Corydal	MOL004231	Tetrahydrocory samine	Sodium channel protein type 5 subunit alpha
Rhizoma Corydal	MOL004231	Tetrahydrocory samine	Muscarinic acetylcholine receptor M5
Rhizoma Corydal	MOL004231	Tetrahydrocory samine	Prostaglandin G/H synthase 2
Rhizoma Corydal	MOL004231	Tetrahydrocory samine	Muscarinic acetylcholine receptor M4
Rhizoma Corydal	MOL004231	Tetrahydrocory samine	Retinoic acid receptor RXR-alpha
Rhizoma Corydal	MOL004231	Tetrahydrocory samine	Delta-type opioid receptor
Rhizoma Corydal	MOL004231	Tetrahydrocory samine	Alpha-1B adrenergic receptor
Rhizoma Corydal	MOL004231	Tetrahydrocory samine	Beta-2 adrenergic receptor
Rhizoma Corydal	MOL004231	Tetrahydrocory samine	Alpha-1D adrenergic receptor
Rhizoma Corydal	MOL004231	Tetrahydrocory samine	Mu-type opioid receptor
Rhizoma Corydal	MOL004231	Tetrahydrocory samine	Calmodulin

Rhizoma Corydal	MOL004232	tetrahydroprotopapaverine	Dopamine D1 receptor
Rhizoma Corydal	MOL004232	tetrahydroprotopapaverine	Muscarinic acetylcholine receptor M3
Rhizoma Corydal	MOL004232	tetrahydroprotopapaverine	Potassium voltage-gated channel subfamily H member 2
Rhizoma Corydal	MOL004232	tetrahydroprotopapaverine	Muscarinic acetylcholine receptor M1
Rhizoma Corydal	MOL004232	tetrahydroprotopapaverine	Beta-1 adrenergic receptor
Rhizoma Corydal	MOL004232	tetrahydroprotopapaverine	Sodium channel protein type 5 subunit alpha
Rhizoma Corydal	MOL004232	tetrahydroprotopapaverine	Coagulation factor Xa
Rhizoma Corydal	MOL004232	tetrahydroprotopapaverine	Muscarinic acetylcholine receptor M5
Rhizoma Corydal	MOL004232	tetrahydroprotopapaverine	Prostaglandin G/H synthase 2
Rhizoma Corydal	MOL004232	tetrahydroprotopapaverine	Alpha-2C adrenergic receptor
Rhizoma Corydal	MOL004232	tetrahydroprotopapaverine	Muscarinic acetylcholine receptor M4
Rhizoma Corydal	MOL004232	tetrahydroprotopapaverine	Retinoic acid receptor RXR-alpha
Rhizoma Corydal	MOL004232	tetrahydroprotopapaverine	Delta-type opioid receptor
Rhizoma Corydal	MOL004232	tetrahydroprotopapaverine	Sodium-dependent noradrenaline transporter
Rhizoma Corydal	MOL004232	tetrahydroprotopapaverine	5-hydroxytryptamine 2C receptor

Rhizoma Corydal	MOL004232	tetrahydroproto papaverine	Alpha-1B adrenergic receptor
Rhizoma Corydal	MOL004232	tetrahydroproto papaverine	Sodium-dependent dopamine transporter
Rhizoma Corydal	MOL004232	tetrahydroproto papaverine	Beta-2 adrenergic receptor
Rhizoma Corydal	MOL004232	tetrahydroproto papaverine	Alpha-1D adrenergic receptor
Rhizoma Corydal	MOL004232	tetrahydroproto papaverine	Sodium-dependent serotonin transporter
Rhizoma Corydal	MOL004232	tetrahydroproto papaverine	Mu-type opioid receptor
Rhizoma Corydal	MOL004232	tetrahydroproto papaverine	Heat shock protein HSP 90
Rhizoma Corydal	MOL004232	tetrahydroproto papaverine	Calmodulin
Rhizoma Corydal	MOL004233	ST057701	Prostaglandin G/H synthase 1
Rhizoma Corydal	MOL004233	ST057701	Muscarinic acetylcholine receptor M3
Rhizoma Corydal	MOL004233	ST057701	Potassium voltage-gated channel subfamily H member 2
Rhizoma Corydal	MOL004233	ST057701	Muscarinic acetylcholine receptor M1
Rhizoma Corydal	MOL004233	ST057701	Androgen receptor
Rhizoma Corydal	MOL004233	ST057701	Sodium channel protein type 5 subunit alpha
Rhizoma Corydal	MOL004233	ST057701	Coagulation factor Xa
Rhizoma Corydal	MOL004233	ST057701	Muscarinic acetylcholine receptor M5
Rhizoma Corydal	MOL004233	ST057701	Prostaglandin G/H synthase 2
Rhizoma Corydal	MOL004233	ST057701	Nitric-oxide synthase, endothelial
Rhizoma Corydal	MOL004233	ST057701	Muscarinic acetylcholine receptor M4
Rhizoma Corydal	MOL004233	ST057701	Retinoic acid receptor RXR-alpha

Rhizoma Corydal	MOL004233	ST057701	Delta-type opioid receptor
Rhizoma Corydal	MOL004233	ST057701	5-hydroxytryptamine 2A receptor
Rhizoma Corydal	MOL004233	ST057701	5-hydroxytryptamine 2C receptor
Rhizoma Corydal	MOL004233	ST057701	Alpha-1B adrenergic receptor
Rhizoma Corydal	MOL004233	ST057701	Sodium-dependent dopamine transporter
Rhizoma Corydal	MOL004233	ST057701	Beta-2 adrenergic receptor
Rhizoma Corydal	MOL004233	ST057701	Alpha-1D adrenergic receptor
Rhizoma Corydal	MOL004233	ST057701	DNA topoisomerase II
Rhizoma Corydal	MOL004233	ST057701	Sodium-dependent serotonin transporter
Rhizoma Corydal	MOL004233	ST057701	Mu-type opioid receptor
Rhizoma Corydal	MOL004233	ST057701	Heat shock protein HSP 90
Rhizoma Corydal	MOL004233	ST057701	Trypsin-1
Rhizoma Corydal	MOL004233	ST057701	Calmodulin
Rhizoma Corydal	MOL004234	2,3,9,10-tetramethoxy-13-methyl-5,6-dihydroisoquinolino[2,1-b]isoquinolin-8-one	Nitric oxide synthase, inducible
Rhizoma Corydal	MOL004234	2,3,9,10-tetramethoxy-13-methyl-5,6-dihydroisoquinolino[2,1-b]isoquinolin-8-one	Prostaglandin G/H synthase 1

Rhizoma Corydal	MOL004234	2,3,9,10-tetramethoxy-13-methyl-5,6-dihydroisoquinolino[2,1-b]isoquinolin-8-one	Potassium voltage-gated channel subfamily H member 2
Rhizoma Corydal	MOL004234	2,3,9,10-tetramethoxy-13-methyl-5,6-dihydroisoquinolino[2,1-b]isoquinolin-8-one	Androgen receptor
Rhizoma Corydal	MOL004234	2,3,9,10-tetramethoxy-13-methyl-5,6-dihydroisoquinolino[2,1-b]isoquinolin-8-one	Sodium channel protein type 5 subunit alpha
Rhizoma Corydal	MOL004234	2,3,9,10-tetramethoxy-13-methyl-5,6-dihydroisoquinolino[2,1-b]isoquinolin-8-one	Coagulation factor Xa

Rhizoma Corydal	MOL004234	2,3,9,10-tetramethoxy-13-methyl-5,6-dihydroisoquinolino[2,1-b]isoquinolin-8-one	Prostaglandin G/H synthase 2
Rhizoma Corydal	MOL004234	2,3,9,10-tetramethoxy-13-methyl-5,6-dihydroisoquinolino[2,1-b]isoquinolin-8-one	DNA topoisomerase II
Rhizoma Corydal	MOL004234	2,3,9,10-tetramethoxy-13-methyl-5,6-dihydroisoquinolino[2,1-b]isoquinolin-8-one	Heat shock protein HSP 90
Rhizoma Corydal	MOL004234	2,3,9,10-tetramethoxy-13-methyl-5,6-dihydroisoquinolino[2,1-b]isoquinolin-8-one	Serine/threonine-protein kinase Chk1

Rhizoma Corydal	MOL004234	2,3,9,10-tetramethoxy-13-methyl-5,6-dihydroisoquinolino[2,1-b]isoquinolin-8-one	Trypsin-1
Rhizoma Corydal	MOL004234	2,3,9,10-tetramethoxy-13-methyl-5,6-dihydroisoquinolino[2,1-b]isoquinolin-8-one	Nuclear receptor coactivator 2
Rhizoma Corydal	MOL004234	2,3,9,10-tetramethoxy-13-methyl-5,6-dihydroisoquinolino[2,1-b]isoquinolin-8-one	Calmodulin
Rhizoma Corydal	MOL000449	Stigmasterol	Progesterone receptor
Rhizoma Corydal	MOL000449	Stigmasterol	Mineralocorticoid receptor
Rhizoma Corydal	MOL000449	Stigmasterol	Nuclear receptor coactivator 2
Rhizoma Corydal	MOL000449	Stigmasterol	Alcohol dehydrogenase 1C
Rhizoma Corydal	MOL000449	Stigmasterol	Ig gamma-1 chain C region
Rhizoma Corydal	MOL000449	Stigmasterol	Retinoic acid receptor RXR-alpha
Rhizoma Corydal	MOL000449	Stigmasterol	Nuclear receptor coactivator 1
Rhizoma Corydal	MOL000449	Stigmasterol	Prostaglandin G/H synthase 1
Rhizoma Corydal	MOL000449	Stigmasterol	Prostaglandin G/H synthase 2

Rhizoma Corydal	MOL000449	Stigmasterol	Alpha-2A adrenergic receptor
Rhizoma Corydal	MOL000449	Stigmasterol	Sodium-dependent noradrenaline transporter
Rhizoma Corydal	MOL000449	Stigmasterol	Sodium-dependent dopamine transporter
Rhizoma Corydal	MOL000449	Stigmasterol	Beta-2 adrenergic receptor
Rhizoma Corydal	MOL000449	Stigmasterol	Aldose reductase
Rhizoma Corydal	MOL000449	Stigmasterol	Urokinase-type plasminogen activator
Rhizoma Corydal	MOL000449	Stigmasterol	Leukotriene A-4 hydrolase
Rhizoma Corydal	MOL000449	Stigmasterol	Amine oxidase [flavin-containing] B
Rhizoma Corydal	MOL000449	Stigmasterol	Amine oxidase [flavin-containing] A
Rhizoma Corydal	MOL000449	Stigmasterol	mRNA of PKA Catalytic Subunit C-alpha
Rhizoma Corydal	MOL000449	Stigmasterol	Chymotrypsinogen B
Rhizoma Corydal	MOL000449	Stigmasterol	Muscarinic acetylcholine receptor M3
Rhizoma Corydal	MOL000449	Stigmasterol	Muscarinic acetylcholine receptor M1
Rhizoma Corydal	MOL000449	Stigmasterol	Beta-1 adrenergic receptor
Rhizoma Corydal	MOL000449	Stigmasterol	Sodium channel protein type 5 subunit alpha
Rhizoma Corydal	MOL000449	Stigmasterol	5-hydroxytryptamine 2A receptor
Rhizoma Corydal	MOL000449	Stigmasterol	Alpha-1A adrenergic receptor
Rhizoma Corydal	MOL000449	Stigmasterol	Gamma-aminobutyric-acid receptor alpha-3 subunit
Rhizoma Corydal	MOL000449	Stigmasterol	Muscarinic acetylcholine receptor M2
Rhizoma Corydal	MOL000449	Stigmasterol	Alpha-1B adrenergic receptor
Rhizoma Corydal	MOL000449	Stigmasterol	Gamma-aminobutyric acid receptor subunit alpha-1
Rhizoma Corydal	MOL000449	Stigmasterol	Neuronal acetylcholine receptor protein, alpha-7 chain
Rhizoma Corydal	MOL000785	palmatine	Nitric oxide synthase, inducible

Rhizoma Corydal	MOL000785	palmatine	Prostaglandin G/H synthase 1
Rhizoma Corydal	MOL000785	palmatine	Potassium voltage-gated channel subfamily H member 2
Rhizoma Corydal	MOL000785	palmatine	Estrogen receptor
Rhizoma Corydal	MOL000785	palmatine	Androgen receptor
Rhizoma Corydal	MOL000785	palmatine	Sodium channel protein type 5 subunit alpha
Rhizoma Corydal	MOL000785	palmatine	Prostaglandin G/H synthase 2
Rhizoma Corydal	MOL000785	palmatine	Nitric-oxide synthase, endothelial
Rhizoma Corydal	MOL000785	palmatine	Retinoic acid receptor RXR-alpha
Rhizoma Corydal	MOL000785	palmatine	Beta-2 adrenergic receptor
Rhizoma Corydal	MOL000785	palmatine	Estrogen receptor beta
Rhizoma Corydal	MOL000785	palmatine	Heat shock protein HSP 90
Rhizoma Corydal	MOL000785	palmatine	Trypsin-1
Rhizoma Corydal	MOL000785	palmatine	Proto-oncogene serine/threonine-protein kinase Pim-1
Rhizoma Corydal	MOL000785	palmatine	Nuclear receptor coactivator 2
Rhizoma Corydal	MOL000785	palmatine	Calmodulin
Rhizoma Corydal	MOL000785	palmatine	mRNA of PKA Catalytic Subunit C-alpha
Rhizoma Corydal	MOL000785	palmatine	Cell division protein kinase 2
Rhizoma Corydal	MOL000785	palmatine	Coagulation factor VII
Rhizoma Corydal	MOL000787	Fumarine	Prostaglandin G/H synthase 1
Rhizoma Corydal	MOL000787	Fumarine	Muscarinic acetylcholine receptor M3
Rhizoma Corydal	MOL000787	Fumarine	Potassium voltage-gated channel subfamily H member 2
Rhizoma Corydal	MOL000787	Fumarine	Muscarinic acetylcholine receptor M1
Rhizoma Corydal	MOL000787	Fumarine	Sodium channel protein type 5 subunit alpha
Rhizoma Corydal	MOL000787	Fumarine	Coagulation factor Xa

Rhizoma Corydal	MOL000787	Fumarine	Muscarinic acetylcholine receptor M5
Rhizoma Corydal	MOL000787	Fumarine	Prostaglandin G/H synthase 2
Rhizoma Corydal	MOL000787	Fumarine	5-hydroxytryptamine receptor 3A
Rhizoma Corydal	MOL000787	Fumarine	Coagulation factor VII
Rhizoma Corydal	MOL000787	Fumarine	Muscarinic acetylcholine receptor M4
Rhizoma Corydal	MOL000787	Fumarine	Delta-type opioid receptor
Rhizoma Corydal	MOL000787	Fumarine	5-hydroxytryptamine 2A receptor
Rhizoma Corydal	MOL000787	Fumarine	Alpha-1B adrenergic receptor
Rhizoma Corydal	MOL000787	Fumarine	Beta-2 adrenergic receptor
Rhizoma Corydal	MOL000787	Fumarine	Alpha-1D adrenergic receptor
Rhizoma Corydal	MOL000787	Fumarine	Mu-type opioid receptor
Rhizoma Corydal	MOL000787	Fumarine	Heat shock protein HSP 90
Rhizoma Corydal	MOL000787	Fumarine	mRNA of PKA Catalytic Subunit C-alpha
Rhizoma Corydal	MOL000787	Fumarine	Calmodulin
Rhizoma Corydal	MOL000787	Fumarine	Sodium-dependent serotonin transporter
Rhizoma Corydal	MOL000787	Fumarine	Voltage-dependent L-type calcium channel subunit alpha-1S
Rhizoma Corydal	MOL000787	Fumarine	CGMP-inhibited 3',5'-cyclic phosphodiesterase A
Rhizoma Corydal	MOL000787	Fumarine	Sodium-dependent dopamine transporter
Rhizoma Corydal	MOL000787	Fumarine	Type IV phosphodiesterase
Rhizoma Corydal	MOL000787	Fumarine	DNA topoisomerase II
Rhizoma Corydal	MOL000787	Fumarine	Dopamine D1 receptor
Rhizoma Corydal	MOL000787	Fumarine	Vascular endothelial growth factor receptor 2
Rhizoma Corydal	MOL000790	Isocorypalmine	Prostaglandin G/H synthase 1
Rhizoma Corydal	MOL000790	Isocorypalmine	Dopamine D1 receptor

Rhizoma Corydal	MOL000790	Isocorypalmine	Muscarinic acetylcholine receptor M3
Rhizoma Corydal	MOL000790	Isocorypalmine	Potassium voltage-gated channel subfamily H member 2
Rhizoma Corydal	MOL000790	Isocorypalmine	Muscarinic acetylcholine receptor M1
Rhizoma Corydal	MOL000790	Isocorypalmine	D(1B) dopamine receptor
Rhizoma Corydal	MOL000790	Isocorypalmine	Sodium channel protein type 5 subunit alpha
Rhizoma Corydal	MOL000790	Isocorypalmine	Coagulation factor Xa
Rhizoma Corydal	MOL000790	Isocorypalmine	Muscarinic acetylcholine receptor M5
Rhizoma Corydal	MOL000790	Isocorypalmine	Prostaglandin G/H synthase 2
Rhizoma Corydal	MOL000790	Isocorypalmine	5-hydroxytryptamine receptor 3A
Rhizoma Corydal	MOL000790	Isocorypalmine	Carbonic anhydrase II
Rhizoma Corydal	MOL000790	Isocorypalmine	Alpha-2C adrenergic receptor
Rhizoma Corydal	MOL000790	Isocorypalmine	Muscarinic acetylcholine receptor M4
Rhizoma Corydal	MOL000790	Isocorypalmine	Retinoic acid receptor RXR-alpha
Rhizoma Corydal	MOL000790	Isocorypalmine	Delta-type opioid receptor
Rhizoma Corydal	MOL000790	Isocorypalmine	5-hydroxytryptamine 2A receptor
Rhizoma Corydal	MOL000790	Isocorypalmine	Sodium-dependent noradrenaline transporter
Rhizoma Corydal	MOL000790	Isocorypalmine	Alpha-1A adrenergic receptor
Rhizoma Corydal	MOL000790	Isocorypalmine	5-hydroxytryptamine 2C receptor
Rhizoma Corydal	MOL000790	Isocorypalmine	Muscarinic acetylcholine receptor M2
Rhizoma Corydal	MOL000790	Isocorypalmine	Alpha-2B adrenergic receptor
Rhizoma Corydal	MOL000790	Isocorypalmine	Alpha-1B adrenergic receptor
Rhizoma Corydal	MOL000790	Isocorypalmine	D(3) dopamine receptor
Rhizoma Corydal	MOL000790	Isocorypalmine	Sodium-dependent dopamine transporter
Rhizoma Corydal	MOL000790	Isocorypalmine	Beta-2 adrenergic receptor
Rhizoma Corydal	MOL000790	Isocorypalmine	Alpha-1D adrenergic receptor

Rhizoma Corydal	MOL000790	Isocorypalmine	Sodium-dependent serotonin transporter
Rhizoma Corydal	MOL000790	Isocorypalmine	Mu-type opioid receptor
Rhizoma Corydal	MOL000790	Isocorypalmine	Heat shock protein HSP 90
Rhizoma Corydal	MOL000790	Isocorypalmine	mRNA of PKA Catalytic Subunit C-alpha
Rhizoma Corydal	MOL000790	Isocorypalmine	Retinoic acid receptor RXR-beta
Rhizoma Corydal	MOL000790	Isocorypalmine	Ig gamma-1 chain C region
Rhizoma Corydal	MOL000790	Isocorypalmine	Nuclear receptor coactivator 1
Rhizoma Corydal	MOL000790	Isocorypalmine	Calmodulin
Rhizoma Corydal	MOL000790	Isocorypalmine	Calcium-activated potassium channel subunit alpha 1
Rhizoma Corydal	MOL000791	bicuculline	Prostaglandin G/H synthase 1
Rhizoma Corydal	MOL000791	bicuculline	Thrombin
Rhizoma Corydal	MOL000791	bicuculline	Potassium voltage-gated channel subfamily H member 2
Rhizoma Corydal	MOL000791	bicuculline	Androgen receptor
Rhizoma Corydal	MOL000791	bicuculline	Sodium channel protein type 5 subunit alpha
Rhizoma Corydal	MOL000791	bicuculline	Coagulation factor Xa
Rhizoma Corydal	MOL000791	bicuculline	Prostaglandin G/H synthase 2
Rhizoma Corydal	MOL000791	bicuculline	Vascular endothelial growth factor receptor 2
Rhizoma Corydal	MOL000791	bicuculline	Acetylcholinesterase
Rhizoma Corydal	MOL000791	bicuculline	DNA topoisomerase II
Rhizoma Corydal	MOL000791	bicuculline	Heat shock protein HSP 90
Rhizoma Corydal	MOL000791	bicuculline	mRNA of Protein-tyrosine phosphatase, non-receptor type 1
Rhizoma Corydal	MOL000791	bicuculline	mRNA of PKA Catalytic Subunit C-alpha

Rhizoma Corydal MOL000791	bicuculline	Proto-oncogene c-Fos
Rhizoma Corydal MOL000791	bicuculline	Gap junction alpha-1 protein
Rhizoma Corydal MOL000791	bicuculline	Gamma-aminobutyric acid type B receptor subunit 1
Rhizoma Corydal MOL000791	bicuculline	Bone morphogenetic protein receptor type-2
Rhizoma Corydal MOL000791	bicuculline	Metabotropic glutamate receptor 5
Rhizoma Corydal MOL000791	bicuculline	Progonadoliberin-1
Rhizoma Corydal MOL000791	bicuculline	Aldehyde dehydrogenase, dimeric NADP-preferring
Rhizoma Corydal MOL000791	bicuculline	Gonadotropin-releasing hormone receptor
Rhizoma Corydal MOL000791	bicuculline	Corticoliberin
Rhizoma Corydal MOL000791	bicuculline	Glutamate [NMDA] receptor subunit epsilon-4
Rhizoma Corydal MOL000791	bicuculline	Sodium-dependent noradrenaline transporter
Rhizoma Corydal MOL000791	bicuculline	Gap junction beta-1 protein
Rhizoma Corydal MOL000791	bicuculline	Metabotropic glutamate receptor 1
Rhizoma Corydal MOL000791	bicuculline	Transitional endoplasmic reticulum ATPase
Rhizoma Corydal MOL000793	C09367	Prostaglandin G/H synthase 1
Rhizoma Corydal MOL000793	C09367	Muscarinic acetylcholine receptor M3
Rhizoma Corydal MOL000793	C09367	Potassium voltage-gated channel subfamily H member 2
Rhizoma Corydal MOL000793	C09367	Muscarinic acetylcholine receptor M1
Rhizoma Corydal MOL000793	C09367	Androgen receptor
Rhizoma Corydal MOL000793	C09367	Sodium channel protein type 5 subunit alpha
Rhizoma Corydal MOL000793	C09367	Coagulation factor Xa

Rhizoma Corydal MOL000793	C09367	Muscarinic acetylcholine receptor M5
Rhizoma Corydal MOL000793	C09367	Prostaglandin G/H synthase 2
Rhizoma Corydal MOL000793	C09367	Nitric-oxide synthase, endothelial
Rhizoma Corydal MOL000793	C09367	Retinoic acid receptor RXR-alpha
Rhizoma Corydal MOL000793	C09367	Delta-type opioid receptor
Rhizoma Corydal MOL000793	C09367	Alpha-1B adrenergic receptor
Rhizoma Corydal MOL000793	C09367	Beta-2 adrenergic receptor
Rhizoma Corydal MOL000793	C09367	Alpha-1D adrenergic receptor
Rhizoma Corydal MOL000793	C09367	DNA topoisomerase II
Rhizoma Corydal MOL000793	C09367	Mu-type opioid receptor
Rhizoma Corydal MOL000793	C09367	Calmodulin
Rhizoma Corydal MOL000793	C09367	Muscarinic acetylcholine receptor M4
Rhizoma Corydal MOL000098	quercetin	Prostaglandin G/H synthase 1
Rhizoma Corydal MOL000098	quercetin	Androgen receptor
Rhizoma Corydal MOL000098	quercetin	Peroxisome proliferator activated receptor gamma
Rhizoma Corydal MOL000098	quercetin	Prostaglandin G/H synthase 2
Rhizoma Corydal MOL000098	quercetin	Heat shock protein HSP 90
Rhizoma Corydal MOL000098	quercetin	Phosphatidylinositol-4,5-bisphosphate 3-kinase catalytic subunit, gamma isoform
Rhizoma Corydal MOL000098	quercetin	Nuclear receptor coactivator 2
Rhizoma Corydal MOL000098	quercetin	Dipeptidyl peptidase IV
Rhizoma Corydal MOL000098	quercetin	Aldose reductase
Rhizoma Corydal MOL000098	quercetin	Trypsin-1
Rhizoma Corydal MOL000098	quercetin	DNA topoisomerase II
Rhizoma Corydal MOL000098	quercetin	Thrombin
Rhizoma Corydal MOL000098	quercetin	Potassium voltage-gated channel subfamily H member 2

Rhizoma Corydal	MOL000098	quercetin	Sodium channel protein type 5 subunit alpha
Rhizoma Corydal	MOL000098	quercetin	Coagulation factor Xa
Rhizoma Corydal	MOL000098	quercetin	Beta-2 adrenergic receptor
Rhizoma Corydal	MOL000098	quercetin	Stromelysin-1
Rhizoma Corydal	MOL000098	quercetin	mRNA of PKA Catalytic Subunit C-alpha
Rhizoma Corydal	MOL000098	quercetin	Coagulation factor VII
Rhizoma Corydal	MOL000098	quercetin	Nitric-oxide synthase, endothelial
Rhizoma Corydal	MOL000098	quercetin	Retinoic acid receptor RXR-alpha
Rhizoma Corydal	MOL000098	quercetin	Acetylcholinesterase
Rhizoma Corydal	MOL000098	quercetin	Gamma-aminobutyric acid receptor subunit alpha-1
Rhizoma Corydal	MOL000098	quercetin	Amine oxidase [flavin-containing] B
Rhizoma Corydal	MOL000098	quercetin	Transcription factor p65
Rhizoma Corydal	MOL000098	quercetin	Epidermal growth factor receptor
Rhizoma Corydal	MOL000098	quercetin	RAC-alpha serine/threonine-protein kinase
Rhizoma Corydal	MOL000098	quercetin	Vascular endothelial growth factor A
Rhizoma Corydal	MOL000098	quercetin	G1/S-specific cyclin-D1
Rhizoma Corydal	MOL000098	quercetin	Apoptosis regulator Bcl-2
Rhizoma Corydal	MOL000098	quercetin	Bcl-2-like protein 1
Rhizoma Corydal	MOL000098	quercetin	Proto-oncogene c-Fos
Rhizoma Corydal	MOL000098	quercetin	Cyclin-dependent kinase inhibitor 1
Rhizoma Corydal	MOL000098	quercetin	Eukaryotic translation initiation factor 6
Rhizoma Corydal	MOL000098	quercetin	Apoptosis regulator BAX
Rhizoma Corydal	MOL000098	quercetin	Caspase-9
Rhizoma Corydal	MOL000098	quercetin	Urokinase-type plasminogen activator
Rhizoma Corydal	MOL000098	quercetin	72 kDa type IV collagenase

Rhizoma Corydal	MOL000098	quercetin	Matrix metalloproteinase-9
Rhizoma Corydal	MOL000098	quercetin	Mitogen-activated protein kinase 1
Rhizoma Corydal	MOL000098	quercetin	Interleukin-10
Rhizoma Corydal	MOL000098	quercetin	Pro-epidermal growth factor
Rhizoma Corydal	MOL000098	quercetin	Retinoblastoma-associated protein
Rhizoma Corydal	MOL000098	quercetin	Tumor necrosis factor
Rhizoma Corydal	MOL000098	quercetin	Transcription factor AP-1
Rhizoma Corydal	MOL000098	quercetin	Interleukin-6
Rhizoma Corydal	MOL000098	quercetin	Cyclin-dependent kinase inhibitor 2A, isoforms 1/2/3
Rhizoma Corydal	MOL000098	quercetin	Activator of 90 kDa heat shock protein ATPase homolog 1
Rhizoma Corydal	MOL000098	quercetin	Caspase-3
Rhizoma Corydal	MOL000098	quercetin	Cellular tumor antigen p53
Rhizoma Corydal	MOL000098	quercetin	ETS domain-containing protein Elk-1
Rhizoma Corydal	MOL000098	quercetin	NF-kappa-B inhibitor alpha
Rhizoma Corydal	MOL000098	quercetin	NADPH--cytochrome P450 reductase
Rhizoma Corydal	MOL000098	quercetin	Ornithine decarboxylase
Rhizoma Corydal	MOL000098	quercetin	Xanthine dehydrogenase/oxidase
Rhizoma Corydal	MOL000098	quercetin	Caspase-8
Rhizoma Corydal	MOL000098	quercetin	DNA topoisomerase 1
Rhizoma Corydal	MOL000098	quercetin	RAF proto-oncogene serine/threonine-protein kinase
Rhizoma Corydal	MOL000098	quercetin	Superoxide dismutase [Cu-Zn]
Rhizoma Corydal	MOL000098	quercetin	Protein kinase C alpha type
Rhizoma Corydal	MOL000098	quercetin	Interstitial collagenase
Rhizoma Corydal	MOL000098	quercetin	Hypoxia-inducible factor 1-alpha
Rhizoma Corydal	MOL000098	quercetin	Signal transducer and activator of transcription 1-alpha/beta

Rhizoma Corydal	MOL000098	quercetin	Protein CBFA2T1
Rhizoma Corydal	MOL000098	quercetin	Probable E3 ubiquitin-protein ligase HERC5
Rhizoma Corydal	MOL000098	quercetin	Cell division control protein 2 homolog
Rhizoma Corydal	MOL000098	quercetin	78 kDa glucose-regulated protein
Rhizoma Corydal	MOL000098	quercetin	Receptor tyrosine-protein kinase erbB-2
Rhizoma Corydal	MOL000098	quercetin	Peroxisome proliferator-activated receptor gamma
Rhizoma Corydal	MOL000098	quercetin	Acetyl-CoA carboxylase 1
Rhizoma Corydal	MOL000098	quercetin	Heme oxygenase 1
Rhizoma Corydal	MOL000098	quercetin	Cytochrome P450 3A4
Rhizoma Corydal	MOL000098	quercetin	Cytochrome P450 1A2
Rhizoma Corydal	MOL000098	quercetin	Caveolin-1
Rhizoma Corydal	MOL000098	quercetin	Myc proto-oncogene protein
Rhizoma Corydal	MOL000098	quercetin	Tissue factor
Rhizoma Corydal	MOL000098	quercetin	Gap junction alpha-1 protein
Rhizoma Corydal	MOL000098	quercetin	Cytochrome P450 1A1
Rhizoma Corydal	MOL000098	quercetin	Intercellular adhesion molecule 1
Rhizoma Corydal	MOL000098	quercetin	Interleukin-1 beta
Rhizoma Corydal	MOL000098	quercetin	C-C motif chemokine 2
Rhizoma Corydal	MOL000098	quercetin	E-selectin
Rhizoma Corydal	MOL000098	quercetin	Vascular cell adhesion protein 1
Rhizoma Corydal	MOL000098	quercetin	Prostaglandin E2 receptor EP3 subtype
Rhizoma Corydal	MOL000098	quercetin	Interleukin-8
Rhizoma Corydal	MOL000098	quercetin	Protein kinase C beta type
Rhizoma Corydal	MOL000098	quercetin	Baculoviral IAP repeat-containing protein 5

Rhizoma Corydal	MOL000098	quercetin	Dual oxidase 2
Rhizoma Corydal	MOL000098	quercetin	Nitric oxide synthase, endothelial
Rhizoma Corydal	MOL000098	quercetin	Heat shock protein beta-1
Rhizoma Corydal	MOL000098	quercetin	Transforming growth factor beta-1
Rhizoma Corydal	MOL000098	quercetin	Estrogen sulfotransferase
Rhizoma Corydal	MOL000098	quercetin	Maltase-glucoamylase, intestinal
Rhizoma Corydal	MOL000098	quercetin	Interleukin-2
Rhizoma Corydal	MOL000098	quercetin	Nuclear receptor subfamily 1 group I member 2
Rhizoma Corydal	MOL000098	quercetin	Cytochrome P450 1B1
Rhizoma Corydal	MOL000098	quercetin	G2/mitotic-specific cyclin-B1
Rhizoma Corydal	MOL000098	quercetin	Tissue-type plasminogen activator
Rhizoma Corydal	MOL000098	quercetin	Thrombomodulin
Rhizoma Corydal	MOL000098	quercetin	Plasminogen activator inhibitor 1
Rhizoma Corydal	MOL000098	quercetin	Collagen alpha-1(I) chain
Rhizoma Corydal	MOL000098	quercetin	Interferon gamma
Rhizoma Corydal	MOL000098	quercetin	Arachidonate 5-lipoxygenase
Rhizoma Corydal	MOL000098	quercetin	Phosphatidylinositol-3,4,5-trisphosphate 3-phosphatase and dual-specificity protein phosphatase PTEN
Rhizoma Corydal	MOL000098	quercetin	Interleukin-1 alpha
Rhizoma Corydal	MOL000098	quercetin	Myeloperoxidase
Rhizoma Corydal	MOL000098	quercetin	DNA topoisomerase 2-alpha
Rhizoma Corydal	MOL000098	quercetin	Neutrophil cytosol factor 1
Rhizoma Corydal	MOL000098	quercetin	ATP-binding cassette sub-family G member 2
Rhizoma Corydal	MOL000098	quercetin	Hyaluronan synthase 2
Rhizoma Corydal	MOL000098	quercetin	Glutathione S-transferase P

Rhizoma Corydal MOL000098	quercetin	Nuclear factor erythroid 2-related factor 2
Rhizoma Corydal MOL000098	quercetin	NAD(P)H dehydrogenase [quinone] 1
Rhizoma Corydal MOL000098	quercetin	Poly [ADP-ribose] polymerase 1
Rhizoma Corydal MOL000098	quercetin	Aryl hydrocarbon receptor
Rhizoma Corydal MOL000098	quercetin	26S proteasome non-ATPase regulatory subunit 3
Rhizoma Corydal MOL000098	quercetin	Solute carrier family 2, facilitated glucose transporter member 4
Rhizoma Corydal MOL000098	quercetin	Collagen alpha-1(III) chain
Rhizoma Corydal MOL000098	quercetin	DNA gyrase subunit B
Rhizoma Corydal MOL000098	quercetin	C-X-C motif chemokine 11
Rhizoma Corydal MOL000098	quercetin	C-X-C motif chemokine 2
Rhizoma Corydal MOL000098	quercetin	DDB1- and CUL4-associated factor 5
Rhizoma Corydal MOL000098	quercetin	Nuclear receptor subfamily 1 group I member 3
Rhizoma Corydal MOL000098	quercetin	Serine/threonine-protein kinase Chk2
Rhizoma Corydal MOL000098	quercetin	Insulin receptor
Rhizoma Corydal MOL000098	quercetin	Claudin-4
Rhizoma Corydal MOL000098	quercetin	Peroxisome proliferator-activated receptor alpha
Rhizoma Corydal MOL000098	quercetin	Peroxisome proliferator-activated receptor delta
Rhizoma Corydal MOL000098	quercetin	Heat shock factor protein 1
Rhizoma Corydal MOL000098	quercetin	C-reactive protein
Rhizoma Corydal MOL000098	quercetin	C-X-C motif chemokine 10
Rhizoma Corydal MOL000098	quercetin	Inhibitor of nuclear factor kappa-B kinase subunit alpha

Rhizoma Corydal MOL000098	quercetin	Osteopontin
Rhizoma Corydal MOL000098	quercetin	Runt-related transcription factor 2
Rhizoma Corydal MOL000098	quercetin	Ras association domain-containing protein 1
Rhizoma Corydal MOL000098	quercetin	Transcription factor E2F1
Rhizoma Corydal MOL000098	quercetin	Transcription factor E2F2
Rhizoma Corydal MOL000098	quercetin	Prostatic acid phosphatase
Rhizoma Corydal MOL000098	quercetin	Cathepsin D
Rhizoma Corydal MOL000098	quercetin	Insulin-like growth factor-binding protein 3
Rhizoma Corydal MOL000098	quercetin	Insulin-like growth factor II
Rhizoma Corydal MOL000098	quercetin	CD40 ligand
Rhizoma Corydal MOL000098	quercetin	Interferon regulatory factor 1
Rhizoma Corydal MOL000098	quercetin	Receptor tyrosine-protein kinase erbB-3
Rhizoma Corydal MOL000098	quercetin	Serum paraoxonase/arylesterase 1
Rhizoma Corydal MOL000098	quercetin	Type I iodothyronine deiodinase
Rhizoma Corydal MOL000098	quercetin	Procollagen C-endopeptidase enhancer 1
Rhizoma Corydal MOL000098	quercetin	Puromycin-sensitive aminopeptidase
Rhizoma Corydal MOL000098	quercetin	Hexokinase-2
Rhizoma Corydal MOL000098	quercetin	Homeobox protein Nkx-3.1
Rhizoma Corydal MOL000098	quercetin	Ras GTPase-activating protein 1
Rhizoma Corydal MOL000098	quercetin	Peroxidase C1A
Rhizoma Corydal MOL000098	quercetin	Glutathione S-transferase Mu 1
Rhizoma Corydal MOL000098	quercetin	Glutathione S-transferase Mu 2

Table S3 The target protein corresponds to the gene name from Uniport

The target protein corresponds to the gene name from Uniport					The target protein corresponds to the gene name from Uniport （Delete duplicates）	
**Herb**	**MolId**	**MolName**	**Target**	**Symbol**	**Target**	**Symbol**
Astragali	MOL000211	Mairin	Progesterone receptor	PGR	Progesterone receptor	PGR
Astragali	MOL000239	Jaranol	Nitric oxide synthase, inducible	NOS2	Nitric oxide synthase, inducible	NOS2
Astragali	MOL000239	Jaranol	Prostaglandin G/H synthase 1	PTGS1	Prostaglandin G/H synthase 1	PTGS1
Astragali	MOL000239	Jaranol	Androgen receptor	AR	Androgen receptor	AR
Astragali	MOL000239	Jaranol	Sodium channel protein type 5 subunit alpha	SCN5A	Sodium channel protein type 5 subunit alpha	SCN5A
Astragali	MOL000239	Jaranol	Prostaglandin G/H synthase 2	PTGS2	Prostaglandin G/H synthase 2	PTGS2
Astragali	MOL000239	Jaranol	Estrogen receptor beta	ESR2	Estrogen receptor beta	ESR2
Astragali	MOL000239	Jaranol	Serine/threonine-protein kinase Chk1	CHEK1	Serine/threonine-protein kinase Chk1	CHEK1
Astragali	MOL000239	Jaranol	Trypsin-1	PRSS1	Trypsin-1	PRSS1
Astragali	MOL000239	Jaranol	Nuclear receptor coactivator 2	NCOA2	Nuclear receptor coactivator 2	NCOA2
Astragali	MOL000296	hederagenin	Progesterone receptor	PGR	Muscarinic acetylcholine receptor M3	CHRM3
Astragali	MOL000296	hederagenin	Nuclear receptor coactivator 2	NCOA2	Muscarinic acetylcholine receptor M1	CHRM1
Astragali	MOL000296	hederagenin	Muscarinic acetylcholine receptor M3	CHRM3	Muscarinic acetylcholine receptor M2	CHRM2

Astragali	MOL000296	hederagenin	Muscarinic acetylcholine receptor M1	CHRM1	Alpha-1B adrenergic receptor	ADRA1B
Astragali	MOL000296	hederagenin	Muscarinic acetylcholine receptor M2	CHRM2	Gamma-aminobutyric acid receptor subunit alpha-1	GABRA1
Astragali	MOL000296	hederagenin	Alpha-1B adrenergic receptor	ADRA1B	Glutamate receptor 2	GRIA2
Astragali	MOL000296	hederagenin	Gamma-aminobutyric acid receptor subunit alpha-1	GABRA1	Alcohol dehydrogenase 1B	ADH1B
Astragali	MOL000296	hederagenin	Glutamate receptor 2	GRIA2	Alcohol dehydrogenase 1C	ADH1C
Astragali	MOL000296	hederagenin	Alcohol dehydrogenase 1B	ADH1B	Lysozyme	LYZ
Astragali	MOL000296	hederagenin	Alcohol dehydrogenase 1C	ADH1C	Retinoic acid receptor RXR-alpha	RXRA
Astragali	MOL000296	hederagenin	Lysozyme	LYZ	Sodium-dependent noradrenaline transporter	SLC6A2
Astragali	MOL000296	hederagenin	Prostaglandin G/H synthase 1	PTGS1	Estrogen receptor	ESR1
Astragali	MOL000296	hederagenin	Sodium channel protein type 5 subunit alpha	SCN5A	Peroxisome proliferator activated receptor gamma	PPARG
Astragali	MOL000296	hederagenin	Prostaglandin G/H synthase 2	PTGS2	Mitogen-activated protein kinase 14	MAPK14
Astragali	MOL000296	hederagenin	Retinoic acid receptor RXR-alpha	RXRA	Glycogen synthase kinase-3 beta	GSK3B
Astragali	MOL000296	hederagenin	Sodium-dependent noradrenaline transporter	SLC6A2	Cyclin-A2	CCNA2

Astragali	MOL000033	(3S,8S,9S,10R,13R,14S,17R)-10,13-dimethyl-17-[(2R,5S)-5-propan-2-yloctan-2-yl]-2,3,4,7,8,9,11,12,14,15,16,17-dodecahydro-1H-cyclopenta[a]phenanthren-3-ol	Progesterone receptor	PGR	Glycogen phosphorylase, muscle form	PYGM
Astragali	MOL000354	isorhamnetin	Nitric oxide synthase, inducible	NOS2	Aldose reductase	AKR1B1
Astragali	MOL000354	isorhamnetin	Prostaglandin G/H synthase 1	PTGS1	Nuclear receptor coactivator 1	NCOA1
Astragali	MOL000354	isorhamnetin	Estrogen receptor	ESR1	Coagulation factor VII	F7

Astragali	MOL000354	isorham netin	Androgen receptor	AR	Acetylcholinesterase	ACHE
Astragali	MOL000354	isorham netin	Peroxisome proliferator activated receptor gamma	PPARG	Amine oxidase [flavin-containing] B	MAOB
Astragali	MOL000354	isorham netin	Prostaglandin G/H synthase 2	PTGS2	Transcription factor p65	RELA
Astragali	MOL000354	isorham netin	Estrogen receptor beta	ESR2	Neutrophil cytosol factor 1	NCF1
Astragali	MOL000354	isorham netin	Mitogen-activated protein kinase 14	MAPK1 4	Oxidized low-density lipoprotein receptor 1	OLR1
Astragali	MOL000354	isorham netin	Glycogen synthase kinase-3 beta	GSK3B	Beta-1 adrenergic receptor	ADRB1
Astragali	MOL000354	isorham netin	Trypsin-1	PRSS1	5-hydroxytryptamine receptor 3A	HTR3A
Astragali	MOL000354	isorham netin	Cyclin-A2	CCNA2	Alpha-2C adrenergic receptor	ADRA2C
Astragali	MOL000354	isorham netin	Nuclear receptor coactivator 2	NCOA2	Beta-2 adrenergic receptor	ADRB2
Astragali	MOL000354	isorham netin	Glycogen phosphorylase, muscle form	PYGM	Alpha-1D adrenergic receptor	ADRA1D
Astragali	MOL000354	isorham netin	Serine/threonine-protein kinase Chk1	CHEK1	Mu-type opioid receptor	OPRM1
Astragali	MOL000354	isorham netin	Aldose reductase	AKR1B 1	Potassium voltage-gated channel subfamily H member 2	KCNH2
Astragali	MOL000354	isorham netin	Nuclear receptor coactivator 1	NCOA1	Muscarinic acetylcholine receptor M5	CHRM5
Astragali	MOL000354	isorham netin	Coagulation factor VII	F7	Muscarinic acetylcholine receptor M4	CHRM4
Astragali	MOL000354	isorham netin	Acetylcholinesterase	ACHE	Delta-type opioid receptor	OPRD1

Astragali	MOL000354	isorham netin	Gamma-aminobutyric acid receptor subunit alpha-1	GABRA 1	Alpha-1A adrenergic receptor	ADRA1A
Astragali	MOL000354	isorham netin	Amine oxidase [flavin-containing] B	MAOB	Sodium-dependent dopamine transporter	SLC6A3
Astragali	MOL000354	isorham netin	Glutamate receptor 2	GRIA2	Sodium-dependent serotonin transporter	SLC6A4
Astragali	MOL000354	isorham netin	Transcription factor p65	RELA	Retinoic acid receptor RXR-beta	RXRB
Astragali	MOL000354	isorham netin	Neutrophil cytosol factor 1	NCF1	Vascular endothelial growth factor receptor 2	KDR
Astragali	MOL000354	isorham netin	Oxidized low-density lipoprotein receptor 1	OLR1	Hepatocyte growth factor receptor	MET
Astragali	MOL000371	3,9-di-O-methylni ssolin	Nitric oxide synthase, inducible	NOS2	cAMP-dependent protein kinase inhibitor alpha	PKIA
Astragali	MOL000371	3,9-di-O-methylni ssolin	Prostaglandin G/H synthase 1	PTGS1	Transcription factor AP-1	JUN
Astragali	MOL000371	3,9-di-O-methylni ssolin	Muscarinic acetylcholine receptor M3	CHRM3	Peroxisome proliferator-activated receptor gamma	PPARG
Astragali	MOL000371	3,9-di-O-methylni ssolin	Muscarinic acetylcholine receptor M1	CHRM1	Interleukin-4	IL4

Astragali	MOL000371	3,9-di-O-methylnissolin	Estrogen receptor	ESR1	ATP synthase subunit beta, mitochondrial	ATP5F1B
Astragali	MOL000371	3,9-di-O-methylnissolin	Beta-1 adrenergic receptor	ADRB1	NADH-ubiquinone oxidoreductase chain 6	ND6
Astragali	MOL000371	3,9-di-O-methylnissolin	Sodium channel protein type 5 subunit alpha	SCN5A	3 beta-hydroxysteroid dehydrogenase/Delta 5-->4-isomerase type 2	HSD3B2
Astragali	MOL000371	3,9-di-O-methylnissolin	Prostaglandin G/H synthase 2	PTGS2	3 beta-hydroxysteroid dehydrogenase/Delta 5-->4-isomerase type 1	HSD3B1
Astragali	MOL000371	3,9-di-O-methylnissolin	5-hydroxytryptamine receptor 3A	HTR3A	Inhibitor of nuclear factor kappa-B kinase subunit beta	IKBKB
Astragali	MOL000371	3,9-di-O-methylnissolin	Alpha-2C adrenergic receptor	ADRA2C	RAC-alpha serine/threonine-protein kinase	AKT1
Astragali	MOL000371	3,9-di-O-methylnissolin	Retinoic acid receptor RXR-alpha	RXRA	Apoptosis regulator Bcl-2	BCL2

Astragali	MOL000371	3,9-di-O-methylnissolin	Acetylcholinesterase	ACHE	Apoptosis regulator BAX	BAX
Astragali	MOL000371	3,9-di-O-methylnissolin	Alpha-1B adrenergic receptor	ADRA1B	Tumor necrosis factor	TNFSF15
Astragali	MOL000371	3,9-di-O-methylnissolin	Beta-2 adrenergic receptor	ADRB2	Activator of 90 kDa heat shock protein ATPase homolog 1	AHSA1
Astragali	MOL000371	3,9-di-O-methylnissolin	Alpha-1D adrenergic receptor	ADRA1D	Caspase-3	CASP3
Astragali	MOL000371	3,9-di-O-methylnissolin	Mu-type opioid receptor	OPRM1	Mitogen-activated protein kinase 8	MAPK8
Astragali	MOL000371	3,9-di-O-methylnissolin	Gamma-aminobutyric acid receptor subunit alpha-1	GABRA1	Interstitial collagenase	MMP1
Astragali	MOL000371	3,9-di-O-methylnissolin	Trypsin-1	PRSS1	Signal transducer and activator of transcription 1-alpha/beta	STAT1

Astragali	MOL000371	3,9-di-O-methylnissolin	Nuclear receptor coactivator 2	NCOA2	Heme oxygenase 1	HMOX1
Astragali	MOL000378	7-O-methylisomucronulatol	Nitric oxide synthase, inducible	NOS2	Cytochrome P450 3A4	CYP3A4
Astragali	MOL000378	7-O-methylisomucronulatol	Prostaglandin G/H synthase 1	PTGS1	Cytochrome P450 1A2	CYP1A2
Astragali	MOL000378	7-O-methylisomucronulatol	Muscarinic acetylcholine receptor M3	CHRM3	Cytochrome P450 1A1	CYP1A1
Astragali	MOL000378	7-O-methylisomucronulatol	Potassium voltage-gated channel subfamily H member 2	KCNH2	Intercellular adhesion molecule 1	ICAM1
Astragali	MOL000378	7-O-methylisomucronulatol	Muscarinic acetylcholine receptor M1	CHRM1	E-selectin	SELE
Astragali	MOL000378	7-O-methylisomucronulatol	Estrogen receptor	ESR1	Vascular cell adhesion protein 1	VCAM1

Astragali	MOL000378	7-O-methylisomucronulatol	Androgen receptor	AR	Nuclear receptor subfamily 1 group I member 2	NR1I2
Astragali	MOL000378	7-O-methylisomucronulatol	Beta-1 adrenergic receptor	ADRB1	Cytochrome P450 1B1	CYP1B1
Astragali	MOL000378	7-O-methylisomucronulatol	Sodium channel protein type 5 subunit alpha	SCN5A	Arachidonate 5-lipoxygenase	ALOX5
Astragali	MOL000378	7-O-methylisomucronulatol	Peroxisome proliferator activated receptor gamma	PPARG	Hyaluronan synthase 2	HAS2
Astragali	MOL000378	7-O-methylisomucronulatol	Muscarinic acetylcholine receptor M5	CHRM5	Glutathione S-transferase P	GSTP1
Astragali	MOL000378	7-O-methylisomucronulatol	Prostaglandin G/H synthase 2	PTGS2	Aryl hydrocarbon receptor	AHR
Astragali	MOL000378	7-O-methylisomucronulatol	Alpha-2C adrenergic receptor	ADRA2C	26S proteasome non-ATPase regulatory subunit 3	PSMD3

Astragali	MOL000378	7-O-methylisomucronulatol	Muscarinic acetylcholine receptor M4	CHRM4	Solute carrier family 2, facilitated glucose transporter member 4	SLC2A4
Astragali	MOL000378	7-O-methylisomucronulatol	Retinoic acid receptor RXR-alpha	RXRA	Nuclear receptor subfamily 1 group I member 3	NR1I3
Astragali	MOL000378	7-O-methylisomucronulatol	Delta-type opioid receptor	OPRD1	Insulin receptor	INSR
Astragali	MOL000378	7-O-methylisomucronulatol	Alpha-1A adrenergic receptor	ADRA1A	Type I iodothyronine deiodinase	DIO1
Astragali	MOL000378	7-O-methylisomucronulatol	Muscarinic acetylcholine receptor M2	CHRM2	Serine/threonine-protein phosphatase 2B catalytic subunit alpha isoform	PPP3CA
Astragali	MOL000378	7-O-methylisomucronulatol	Alpha-1B adrenergic receptor	ADRA1B	Glutathione S-transferase Mu 1	GSTM1
Astragali	MOL000378	7-O-methylisomucronulatol	Sodium-dependent dopamine transporter	SLC6A3	Glutathione S-transferase Mu 2	GSTM2

Astragali	MOL000378	7-O-methylisomucronulatol	Beta-2 adrenergic receptor	ADRB2	Aldo-keto reductase family 1 member C3	AKR1C3
Astragali	MOL000378	7-O-methylisomucronulatol	Alpha-1D adrenergic receptor	ADRA1D	Antileukoproteinase	SLPI
Astragali	MOL000378	7-O-methylisomucronulatol	Sodium-dependent serotonin transporter	SLC6A4	Stromelysin-1	MMP3
Astragali	MOL000378	7-O-methylisomucronulatol	Estrogen receptor beta	ESR2	Epidermal growth factor receptor	EGFR
Astragali	MOL000378	7-O-methylisomucronulatol	Gamma-aminobutyric acid receptor subunit alpha-1	GABRA1	Vascular endothelial growth factor A	VEGFA
Astragali	MOL000378	7-O-methylisomucronulatol	Mitogen-activated protein kinase 14	MAPK14	G1/S-specific cyclin-D1	CCND1
Astragali	MOL000378	7-O-methylisomucronulatol	Glycogen synthase kinase-3 beta	GSK3B	Bcl-2-like protein 1	BCL2L1

Astragali	MOL000378	7-O-methylisomucronulatol	Serine/threonine-protein kinase Chk1	CHEK1	Proto-oncogene c-Fos	FOS
Astragali	MOL000378	7-O-methylisomucronulatol	Retinoic acid receptor RXR-beta	RXRB	Cyclin-dependent kinase inhibitor 1	CDKN1A
Astragali	MOL000378	7-O-methylisomucronulatol	Trypsin-1	PRSS1	Eukaryotic translation initiation factor 6	EIF6
Astragali	MOL000378	7-O-methylisomucronulatol	Cyclin-A2	CCNA2	Caspase-9	CASP9
Astragali	MOL000378	7-O-methylisomucronulatol	Nuclear receptor coactivator 2	NCOA2	Urokinase-type plasminogen activator	PLAU
Astragali	MOL000379	9,10-dimethoxypterocarpan-3-O-β-D-glucoside	Prostaglandin G/H synthase 2	PTGS2	72 kDa type IV collagenase	MMP2

Astragali	MOL000379	9,10-dimethoxypterocarpan-3-O-β-D-glucoside	Nuclear receptor coactivator 2	NCOA2	Matrix metalloproteinase-9	MMP9
Astragali	MOL000380	(6aR,11aR)-9,10-dimethoxy-6a,11a-dihydro-6H-benzofurano[3,2-c]chromen-3-ol	Nitric oxide synthase, inducible	NOS2	Mitogen-activated protein kinase 1	MAPK1

Astragali	MOL000380	(6aR,11aR)-9,10-dimethoxy-6a,11a-dihydro-6H-benzofurano[3,2-c]chromen-3-ol	Prostaglandin G/H synthase 1	PTGS1	Interleukin-10	IL10
Astragali	MOL000380	(6aR,11aR)-9,10-dimethoxy-6a,11a-dihydro-6H-benzofurano[3,2-c]chromen-3-ol	Muscarinic acetylcholine receptor M3	CHRM3	Pro-epidermal growth factor	EGF

Astragali	MOL000380	(6aR,11aR)-9,10-dimethoxy-6a,11a-dihydro-6H-benzofurano[3,2-c]chromen-3-ol	Muscarinic acetylcholine receptor M1	CHRM1	Retinoblastoma-associated protein	RB1
Astragali	MOL000380	(6aR,11aR)-9,10-dimethoxy-6a,11a-dihydro-6H-benzofurano[3,2-c]chromen-3-ol	Estrogen receptor	ESR1	Interleukin-6	IL6

Astragali	MOL000380	(6aR,11aR)-9,10-dimethoxy-6a,11a-dihydro-6H-benzofurano[3,2-c]chromen-3-ol	Sodium channel protein type 5 subunit alpha	SCN5A	Cellular tumor antigen p53	TP63
Astragali	MOL000380	(6aR,11aR)-9,10-dimethoxy-6a,11a-dihydro-6H-benzofurano[3,2-c]chromen-3-ol	Prostaglandin G/H synthase 2	PTGS2	ETS domain-containing protein Elk-1	ELK1

Astragali	MOL000380	(6aR,11aR)-9,10-dimethoxy-6a,11a-dihydro-6H-benzofurano[3,2-c]chromen-3-ol	5-hydroxytryptamine receptor 3A	HTR3A	NF-kappa-B inhibitor alpha	NFKBIA	
Astragali	MOL000380	(6aR,11aR)-9,10-dimethoxy-6a,11a-dihydro-6H-benzofurano[3,2-c]chromen-3-ol	Retinoic acid receptor RXR-alpha	RXRA	NADPH--cytochrome P450 reductase	POR	

Astragali	MOL000380	(6aR,11aR)-9,10-dimethoxy-6a,11a-dihydro-6H-benzofurano[3,2-c]chromen-3-ol	Acetylcholinesterase	ACHE	Ornithine decarboxylase	ODC1
Astragali	MOL000380	(6aR,11aR)-9,10-dimethoxy-6a,11a-dihydro-6H-benzofurano[3,2-c]chromen-3-ol	Alpha-1B adrenergic receptor	ADRA1B	Caspase-8	CASP8

Astragali	MOL000380	(6aR,11aR)-9,10-dimethoxy-6a,11a-dihydro-6H-benzofurano[3,2-c]chromen-3-ol	Beta-2 adrenergic receptor	ADRB2	DNA topoisomerase 1	TOP1
Astragali	MOL000380	(6aR,11aR)-9,10-dimethoxy-6a,11a-dihydro-6H-benzofurano[3,2-c]chromen-3-ol	Alpha-1D adrenergic receptor	ADRA1D	RAF proto-oncogene serine/threonine-protein kinase	RAF1

Astragali	MOL000380	(6aR,11aR)-9,10-dimethoxy-6a,11a-dihydro-6H-benzofurano[3,2-c]chromen-3-ol	Gamma-aminobutyric acid receptor subunit alpha-1	GABRA1	Superoxide dismutase [Cu-Zn]	SOD1
Astragali	MOL000380	(6aR,11aR)-9,10-dimethoxy-6a,11a-dihydro-6H-benzofurano[3,2-c]chromen-3-ol	Trypsin-1	PRSS1	Protein kinase C alpha type	PRKCA

Astragali	MOL000380	(6aR,11aR)-9,10-dimethoxy-6a,11a-dihydro-6H-benzofurano[3,2-c]chromen-3-ol	Nuclear receptor coactivator 2	NCOA2	Hypoxia-inducible factor 1-alpha	HIF1A
Astragali	MOL000380	(6aR,11aR)-9,10-dimethoxy-6a,11a-dihydro-6H-benzofurano[3,2-c]chromen-3-ol	Nuclear receptor coactivator 1	NCOA1	Protein CBFA2T1	RUNX1T1

Astragali	MOL000380	(6aR,11aR)-9,10-dimethoxy-6a,11a-dihydro-6H-benzofurano[3,2-c]chromen-3-ol	Muscarinic acetylcholine receptor M4	CHRM4	Receptor tyrosine-protein kinase erbB-2	ERBB2
Astragali	MOL000387	Bifendate	Prostaglandin G/H synthase 2	PTGS2	Acetyl-CoA carboxylase 1	ACACA
Astragali	MOL000387	Bifendate	Vascular endothelial growth factor receptor 2	KDR	Caveolin-1	CAV1
Astragali	MOL000387	Bifendate	Hepatocyte growth factor receptor	MET	Myc proto-oncogene protein	MYC
Astragali	MOL000387	Bifendate	Prostaglandin G/H synthase 1	PTGS1	Tissue factor	F3
Astragali	MOL000392	formononetin	Nitric oxide synthase, inducible	NOS2	Gap junction alpha-1 protein	GJA1
Astragali	MOL000392	formononetin	Prostaglandin G/H synthase 1	PTGS1	Interleukin-1 beta	IL1B
Astragali	MOL000392	formononetin	Muscarinic acetylcholine receptor M1	CHRM1	C-C motif chemokine 2	CCL2
Astragali	MOL000392	formononetin	Estrogen receptor	ESR1	Prostaglandin E2 receptor EP3 subtype	PTGER3
Astragali	MOL000392	formononetin	Androgen receptor	AR	Interleukin-8	CXCL8

Astragali	MOL000392	formono netin	Peroxisome proliferator activated receptor gamma	PPARG	Protein kinase C beta type	PRKCB
Astragali	MOL000392	formono netin	Prostaglandin G/H synthase 2	PTGS2	Baculoviral IAP repeat-containing protein 5	BIRC5
Astragali	MOL000392	formono netin	Retinoic acid receptor RXR-alpha	RXRA	Dual oxidase 2	DUOX2
Astragali	MOL000392	formono netin	Alpha-1A adrenergic receptor	ADRA1 A	Nitric oxide synthase, endothelial	NOS3
Astragali	MOL000392	formono netin	Sodium-dependent dopamine transporter	SLC6A3	Heat shock protein beta-1	HSPB1
Astragali	MOL000392	formono netin	Beta-2 adrenergic receptor	ADRB2	Estrogen sulfotransferase	SULT1E1
Astragali	MOL000392	formono netin	Sodium-dependent serotonin transporter	SLC6A4	Maltase-glucoamylase, intestinal	MGAM
Astragali	MOL000392	formono netin	Estrogen receptor beta	ESR2	Interleukin-2	IL2
Astragali	MOL000392	formono netin	Mitogen-activated protein kinase 14	MAPK1 4	G2/mitotic-specific cyclin-B1	CCNB1
Astragali	MOL000392	formono netin	Glycogen synthase kinase-3 beta	GSK3B	Tissue-type plasminogen activator	PLAT
Astragali	MOL000392	formono netin	Amine oxidase [flavin-containing] B	MAOB	Thrombomodulin	THBD
Astragali	MOL000392	formono netin	Serine/threonine-protein kinase Chk1	CHEK1	Plasminogen activator inhibitor 1	SERPINE1
Astragali	MOL000392	formono netin	Trypsin-1	PRSS1	Collagen alpha-1(I) chain	COL1A1
Astragali	MOL000392	formono netin	Cyclin-A2	CCNA2	Interferon gamma	IFNG
Astragali	MOL000392	formono netin	cAMP-dependent protein kinase inhibitor alpha	PKIA	Interleukin-1 alpha	IL1A

Astragali	MOL000392	formono netin	Acetylcholinesterase	ACHE	Myeloperoxidase	MPO
Astragali	MOL000392	formono netin	Transcription factor AP-1	JUN	DNA topoisomerase 2-alpha	TOP2A
Astragali	MOL000392	formono netin	Peroxisome proliferator-activated receptor gamma	PPARG	ATP-binding cassette sub-family G member 2	ABCG2
Astragali	MOL000392	formono netin	Interleukin-4	IL4	Nuclear factor erythroid 2-related factor 2	NFE2L2
Astragali	MOL000392	formono netin	ATP synthase subunit beta, mitochondrial	ATP5F1 B	NAD(P)H dehydrogenase [quinone] 1	NQO1
Astragali	MOL000392	formono netin	NADH-ubiquinone oxidoreductase chain 6	ND6	Poly [ADP-ribose] polymerase 1	PARP1
Astragali	MOL000392	formono netin	3 beta-hydroxysteroid dehydrogenase/Delta 5-->4-isomerase type 2	HSD3B 2	Collagen alpha-1(III) chain	COL3A1
Astragali	MOL000392	formono netin	3 beta-hydroxysteroid dehydrogenase/Delta 5-->4-isomerase type 1	HSD3B 1	C-X-C motif chemokine 11	CXCL11
Astragali	MOL000417	Calycosi n	Nitric oxide synthase, inducible	NOS2	C-X-C motif chemokine 2	CXCL2
Astragali	MOL000417	Calycosi n	Prostaglandin G/H synthase 1	PTGS1	DDB1- and CUL4-associated factor 5	DCAF5
Astragali	MOL000417	Calycosi n	Estrogen receptor	ESR1	Serine/threonine-protein kinase Chk2	CHEK2
Astragali	MOL000417	Calycosi n	Androgen receptor	AR	Claudin-4	CLDN4
Astragali	MOL000417	Calycosi n	Peroxisome proliferator activated receptor gamma	PPARG	Peroxisome proliferator-activated receptor alpha	PPARA
Astragali	MOL000417	Calycosi n	Prostaglandin G/H synthase 2	PTGS2	Peroxisome proliferator-activated receptor delta	PPARD

Astragali	MOL000417	Calycosin	Retinoic acid receptor RXR-alpha	RXRA	Heat shock factor protein 1	HSF1
Astragali	MOL000417	Calycosin	Estrogen receptor beta	ESR2	C-reactive protein	CRP
Astragali	MOL000417	Calycosin	Mitogen-activated protein kinase 14	MAPK14	C-X-C motif chemokine 10	CXCL10
Astragali	MOL000417	Calycosin	Glycogen synthase kinase-3 beta	GSK3B	Inhibitor of nuclear factor kappa-B kinase subunit alpha	CHUK
Astragali	MOL000417	Calycosin	Serine/threonine-protein kinase Chk1	CHEK1	Osteopontin	SPP1
Astragali	MOL000417	Calycosin	Trypsin-1	PRSS1	Runt-related transcription factor 2	RUNX2
Astragali	MOL000417	Calycosin	Cyclin-A2	CCNA2	Ras association domain-containing protein 1	RASSF1
Astragali	MOL000417	Calycosin	Nuclear receptor coactivator 2	NCOA2	Transcription factor E2F1	E2F1
Astragali	MOL000417	Calycosin	Beta-2 adrenergic receptor	ADRB2	Transcription factor E2F2	E2F2
Astragali	MOL000422	kaempferol	Nitric oxide synthase, inducible	NOS2	Prostatic acid phosphatase	ACPP
Astragali	MOL000422	kaempferol	Prostaglandin G/H synthase 1	PTGS1	Cathepsin D	CTSD
Astragali	MOL000422	kaempferol	Androgen receptor	AR	Insulin-like growth factor-binding protein 3	IGFBP3
Astragali	MOL000422	kaempferol	Peroxisome proliferator activated receptor gamma	PPARG	Insulin-like growth factor II	IGF2
Astragali	MOL000422	kaempferol	Prostaglandin G/H synthase 2	PTGS2	CD40 ligand	CD40LG
Astragali	MOL000422	kaempferol	Nuclear receptor coactivator 2	NCOA2	Interferon regulatory factor 1	IRF1

Astragali	MOL000422	kaempferol	Trypsin-1	PRSS1	Receptor tyrosine-protein kinase erbB-3	ERBB3
Astragali	MOL000422	kaempferol	Progesterone receptor	PGR	Serum paraoxonase/arylesterase 1	PON1
Astragali	MOL000422	kaempferol	Muscarinic acetylcholine receptor M1	CHRM1	Procollagen C-endopeptidase enhancer 1	PCOLCE
Astragali	MOL000422	kaempferol	Acetylcholinesterase	ACHE	Puromycin-sensitive aminopeptidase	NPEPPS
Astragali	MOL000422	kaempferol	Sodium-dependent noradrenaline transporter	SLC6A2	Hexokinase-2	HK2
Astragali	MOL000422	kaempferol	Muscarinic acetylcholine receptor M2	CHRM2	Ras GTPase-activating protein 1	RASA1
Astragali	MOL000422	kaempferol	Alpha-1B adrenergic receptor	ADRA1B	Neuronal acetylcholine receptor subunit alpha-2	CHRNA2
Astragali	MOL000422	kaempferol	Gamma-aminobutyric acid receptor subunit alpha-1	GABRA1	Microtubule-associated protein 2	MAP2
Astragali	MOL000422	kaempferol	Coagulation factor VII	F7	Alpha-2B adrenergic receptor	ADRA2B
Astragali	MOL000422	kaempferol	Transcription factor p65	RELA	Voltage-dependent L-type calcium channel subunit alpha-1S	CACNA1S
Astragali	MOL000422	kaempferol	Inhibitor of nuclear factor kappa-B kinase subunit beta	IKBKB	D(1B) dopamine receptor	DRD5
Astragali	MOL000422	kaempferol	RAC-alpha serine/threonine-protein kinase	AKT1	Alpha-2A adrenergic receptor	ADRA2A
Astragali	MOL000422	kaempferol	Apoptosis regulator Bcl-2	BCL2	D(3) dopamine receptor	DRD3
Astragali	MOL000422	kaempferol	Apoptosis regulator BAX	BAX	D(2) dopamine receptor	DRD2

Astragali	MOL000422	kaempferol	Tumor necrosis factor	TNFSF15	cAMP and cAMP-inhibited cGMP 3',5'-cyclic phosphodiesterase 10A	PDE10A
Astragali	MOL000422	kaempferol	Transcription factor AP-1	JUN	Mineralocorticoid receptor	NR3C2
Astragali	MOL000422	kaempferol	Activator of 90 kDa heat shock protein ATPase homolog 1	AHSA1	Glucocorticoid receptor	NR3C1
Astragali	MOL000422	kaempferol	Caspase-3	CASP3	D(4) dopamine receptor	DRD4
Astragali	MOL000422	kaempferol	Mitogen-activated protein kinase 8	MAPK8	Serum albumin	ALB
Astragali	MOL000422	kaempferol	Interstitial collagenase	MMP1	Catenin beta-1	CTNNB1
Astragali	MOL000422	kaempferol	Signal transducer and activator of transcription 1-alpha/beta	STAT1	Caspase-7	CASP7
Astragali	MOL000422	kaempferol	Peroxisome proliferator-activated receptor gamma	PPARG	Stromelysin-2	MMP10
Astragali	MOL000422	kaempferol	Heme oxygenase 1	HMOX1	Leukotriene A-4 hydrolase	LTA4H
Astragali	MOL000422	kaempferol	Cytochrome P450 3A4	CYP3A4	Amine oxidase [flavin-containing] A	MAOA
Astragali	MOL000422	kaempferol	Cytochrome P450 1A2	CYP1A2	Chymotrypsinogen B	CTRB1
Astragali	MOL000422	kaempferol	Cytochrome P450 1A1	CYP1A1	Fatty acid synthase	FASN
Astragali	MOL000422	kaempferol	Intercellular adhesion molecule 1	ICAM1	Glucose-6-phosphate 1-dehydrogenase	G6PD
Astragali	MOL000422	kaempferol	E-selectin	SELE	Endothelin-converting enzyme 1	ECE1

Astragali	MOL000422	kaempferol	Vascular cell adhesion protein 1	VCAM1	Medium-chain specific acyl-CoA dehydrogenase, mitochondrial	ACADM
Astragali	MOL000422	kaempferol	Nuclear receptor subfamily 1 group I member 2	NR1I2	Cytochrome P450 2B6	CYP2B6
Astragali	MOL000422	kaempferol	Cytochrome P450 1B1	CYP1B1	UDP-glucuronosyltransferase 1-1	UGT1A1
Astragali	MOL000422	kaempferol	Arachidonate 5-lipoxygenase	ALOX5	Sterol regulatory element-binding protein 1	SREBF1
Astragali	MOL000422	kaempferol	Hyaluronan synthase 2	HAS2	NADPH oxidase 3	NOX3
Astragali	MOL000422	kaempferol	Glutathione S-transferase P	GSTP1	NADPH oxidase 1	NOX1
Astragali	MOL000422	kaempferol	Aryl hydrocarbon receptor	AHR	Peroxisomal acyl-coenzyme A oxidase 1	ACOX1
Astragali	MOL000422	kaempferol	26S proteasome non-ATPase regulatory subunit 3	PSMD3	ATP-citrate synthase	ACLY
Astragali	MOL000422	kaempferol	Solute carrier family 2, facilitated glucose transporter member 4	SLC2A4	Peroxisomal bifunctional enzyme	EHHADH
Astragali	MOL000422	kaempferol	Nuclear receptor subfamily 1 group I member 3	NR1I3	Methylglutaconyl-CoA hydratase, mitochondrial	AUH
Astragali	MOL000422	kaempferol	Insulin receptor	INSR	Trifunctional enzyme subunit beta, mitochondrial	HADHB
Astragali	MOL000422	kaempferol	Type I iodothyronine deiodinase	DIO1	2,4-dienoyl-CoA reductase, mitochondrial	DECR1
Astragali	MOL000422	kaempferol	Serine/threonine-protein phosphatase 2B catalytic subunit alpha isoform	PPP3CA	4-aminobutyrate aminotransferase, mitochondrial	ABAT

Astragali	MOL000422	kaempferol	Glutathione S-transferase Mu 1	GSTM1	Alcohol dehydrogenase 1A	ADH1A
Astragali	MOL000422	kaempferol	Glutathione S-transferase Mu 2	GSTM2	Heparanase	HPSE
Astragali	MOL000422	kaempferol	Aldo-keto reductase family 1 member C3	AKR1C3	Immediate early response 3-interacting protein 1	IER3IP1
Astragali	MOL000422	kaempferol	Antileukoproteinase	SLPI	CD44 antigen	CD44
Astragali	MOL000433	FA	Glycogen synthase kinase-3 beta	GSK3B	Gamma-aminobutyric acid type B receptor subunit 1	GABBR1
Astragali	MOL000442	1,7-Dihydroxy-3,9-dimethoxy pterocarpene	Prostaglandin G/H synthase 2	PTGS2	Bone morphogenetic protein receptor type-2	BMPR2
Astragali	MOL000442	1,7-Dihydroxy-3,9-dimethoxy pterocarpene	Retinoic acid receptor RXR-alpha	RXRA	Metabotropic glutamate receptor 5	GRM5

Astragali	MOL000442	1,7-Dihydroxy-3,9-dimethoxy pterocarpene	Trypsin-1	PRSS1	Progonadoliberin-1	GNRH1
Astragali	MOL000098	quercetin	Prostaglandin G/H synthase 1	PTGS1	Aldehyde dehydrogenase, dimeric NADP-preferring	ALDH3A1
Astragali	MOL000098	quercetin	Androgen receptor	AR	Gonadotropin-releasing hormone receptor	GNRHR
Astragali	MOL000098	quercetin	Peroxisome proliferator activated receptor gamma	PPARG	Corticoliberin	CRH
Astragali	MOL000098	quercetin	Prostaglandin G/H synthase 2	PTGS2	Gap junction beta-1 protein	GJB1
Astragali	MOL000098	quercetin	Nuclear receptor coactivator 2	NCOA2	Metabotropic glutamate receptor 1	GRM1
Astragali	MOL000098	quercetin	Aldose reductase	AKR1B1	Transitional endoplasmic reticulum ATPase	VCP
Astragali	MOL000098	quercetin	Trypsin-1	PRSS1		
Astragali	MOL000098	quercetin	Potassium voltage-gated channel subfamily H member 2	KCNH2		
Astragali	MOL000098	quercetin	Sodium channel protein type 5 subunit alpha	SCN5A		
Astragali	MOL000098	quercetin	Beta-2 adrenergic receptor	ADRB2		
Astragali	MOL000098	quercetin	Stromelysin-1	MMP3		

Astragali	MOL000098	quercetin	Coagulation factor VII	F7
Astragali	MOL000098	quercetin	Retinoic acid receptor RXR-alpha	RXRA
Astragali	MOL000098	quercetin	Acetylcholinesterase	ACHE
Astragali	MOL000098	quercetin	Gamma-aminobutyric acid receptor subunit alpha-1	GABRA1
Astragali	MOL000098	quercetin	Amine oxidase [flavin-containing] B	MAOB
Astragali	MOL000098	quercetin	Transcription factor p65	RELA
Astragali	MOL000098	quercetin	Epidermal growth factor receptor	EGFR
Astragali	MOL000098	quercetin	RAC-alpha serine/threonine-protein kinase	AKT1
Astragali	MOL000098	quercetin	Vascular endothelial growth factor A	VEGFA
Astragali	MOL000098	quercetin	G1/S-specific cyclin-D1	CCND1
Astragali	MOL000098	quercetin	Apoptosis regulator Bcl-2	BCL2
Astragali	MOL000098	quercetin	Bcl-2-like protein 1	BCL2L1
Astragali	MOL000098	quercetin	Proto-oncogene c-Fos	FOS
Astragali	MOL000098	quercetin	Cyclin-dependent kinase inhibitor 1	CDKN1A

Astragali	MOL000098	quercetin	Eukaryotic translation initiation factor 6	EIF6
Astragali	MOL000098	quercetin	Apoptosis regulator BAX	BAX
Astragali	MOL000098	quercetin	Caspase-9	CASP9
Astragali	MOL000098	quercetin	Urokinase-type plasminogen activator	PLAU
Astragali	MOL000098	quercetin	72 kDa type IV collagenase	MMP2
Astragali	MOL000098	quercetin	Matrix metalloproteinase-9	MMP9
Astragali	MOL000098	quercetin	Mitogen-activated protein kinase 1	MAPK1
Astragali	MOL000098	quercetin	Interleukin-10	IL10
Astragali	MOL000098	quercetin	Pro-epidermal growth factor	EGF
Astragali	MOL000098	quercetin	Retinoblastoma-associated protein	RB1
Astragali	MOL000098	quercetin	Tumor necrosis factor	TNFSF15
Astragali	MOL000098	quercetin	Transcription factor AP-1	JUN
Astragali	MOL000098	quercetin	Interleukin-6	IL6
Astragali	MOL000098	quercetin	Activator of 90 kDa heat shock protein ATPase homolog 1	AHSA1

Astragali	MOL000098	quercetin	Caspase-3	CASP3
Astragali	MOL000098	quercetin	Cellular tumor antigen p53	TP63
Astragali	MOL000098	quercetin	ETS domain-containing protein Elk-1	ELK1
Astragali	MOL000098	quercetin	NF-kappa-B inhibitor alpha	NFKBIA
Astragali	MOL000098	quercetin	NADPH--cytochrome P450 reductase	POR
Astragali	MOL000098	quercetin	Ornithine decarboxylase	ODC1
Astragali	MOL000098	quercetin	Caspase-8	CASP8
Astragali	MOL000098	quercetin	DNA topoisomerase 1	TOP1
Astragali	MOL000098	quercetin	RAF proto-oncogene serine/threonine-protein kinase	RAF1
Astragali	MOL000098	quercetin	Superoxide dismutase [Cu-Zn]	SOD1
Astragali	MOL000098	quercetin	Protein kinase C alpha type	PRKCA
Astragali	MOL000098	quercetin	Interstitial collagenase	MMP1
Astragali	MOL000098	quercetin	Hypoxia-inducible factor 1-alpha	HIF1A
Astragali	MOL000098	quercetin	Signal transducer and activator of transcription 1-alpha/beta	STAT1

Astragali	MOL000098	quercetin	Protein CBFA2T1	RUNX1T1
Astragali	MOL000098	quercetin	Receptor tyrosine-protein kinase erbB-2	ERBB2
Astragali	MOL000098	quercetin	Peroxisome proliferator-activated receptor gamma	PPARG
Astragali	MOL000098	quercetin	Acetyl-CoA carboxylase 1	ACACA
Astragali	MOL000098	quercetin	Heme oxygenase 1	HMOX1
Astragali	MOL000098	quercetin	Cytochrome P450 3A4	CYP3A4
Astragali	MOL000098	quercetin	Cytochrome P450 1A2	CYP1A2
Astragali	MOL000098	quercetin	Caveolin-1	CAV1
Astragali	MOL000098	quercetin	Myc proto-oncogene protein	MYC
Astragali	MOL000098	quercetin	Tissue factor	F3
Astragali	MOL000098	quercetin	Gap junction alpha-1 protein	GJA1
Astragali	MOL000098	quercetin	Cytochrome P450 1A1	CYP1A1
Astragali	MOL000098	quercetin	Intercellular adhesion molecule 1	ICAM1
Astragali	MOL000098	quercetin	Interleukin-1 beta	IL1B
Astragali	MOL000098	quercetin	C-C motif chemokine 2	CCL2

Astragali	MOL000098	quercetin	E-selectin	SELE
Astragali	MOL000098	quercetin	Vascular cell adhesion protein 1	VCAM1
Astragali	MOL000098	quercetin	Prostaglandin E2 receptor EP3 subtype	PTGER3
Astragali	MOL000098	quercetin	Interleukin-8	CXCL8
Astragali	MOL000098	quercetin	Protein kinase C beta type	PRKCB
Astragali	MOL000098	quercetin	Baculoviral IAP repeat-containing protein 5	BIRC5
Astragali	MOL000098	quercetin	Dual oxidase 2	DUOX2
Astragali	MOL000098	quercetin	Nitric oxide synthase, endothelial	NOS3
Astragali	MOL000098	quercetin	Heat shock protein beta-1	HSPB1
Astragali	MOL000098	quercetin	Estrogen sulfotransferase	SULT1E1
Astragali	MOL000098	quercetin	Maltase-glucoamylase, intestinal	MGAM
Astragali	MOL000098	quercetin	Interleukin-2	IL2
Astragali	MOL000098	quercetin	Nuclear receptor subfamily 1 group I member 2	NR1I2
Astragali	MOL000098	quercetin	Cytochrome P450 1B1	CYP1B1
Astragali	MOL000098	quercetin	G2/mitotic-specific cyclin-B1	CCNB1

Astragali	MOL000098	quercetin	Tissue-type plasminogen activator	PLAT
Astragali	MOL000098	quercetin	Thrombomodulin	THBD
Astragali	MOL000098	quercetin	Plasminogen activator inhibitor 1	SERPINE1
Astragali	MOL000098	quercetin	Collagen alpha-1(I) chain	COL1A1
Astragali	MOL000098	quercetin	Interferon gamma	IFNG
Astragali	MOL000098	quercetin	Arachidonate 5-lipoxygenase	ALOX5
Astragali	MOL000098	quercetin	Interleukin-1 alpha	IL1A
Astragali	MOL000098	quercetin	Myeloperoxidase	MPO
Astragali	MOL000098	quercetin	DNA topoisomerase 2-alpha	TOP2A
Astragali	MOL000098	quercetin	Neutrophil cytosol factor 1	NCF1
Astragali	MOL000098	quercetin	ATP-binding cassette sub-family G member 2	ABCG2
Astragali	MOL000098	quercetin	Hyaluronan synthase 2	HAS2
Astragali	MOL000098	quercetin	Glutathione S-transferase P	GSTP1
Astragali	MOL000098	quercetin	Nuclear factor erythroid 2-related factor 2	NFE2L2
Astragali	MOL000098	quercetin	NAD(P)H dehydrogenase [quinone] 1	NQO1

Astragali	MOL000098	quercetin	Poly [ADP-ribose] polymerase 1	PARP1
Astragali	MOL000098	quercetin	Aryl hydrocarbon receptor	AHR
Astragali	MOL000098	quercetin	26S proteasome non-ATPase regulatory subunit 3	PSMD3
Astragali	MOL000098	quercetin	Solute carrier family 2, facilitated glucose transporter member 4	SLC2A4
Astragali	MOL000098	quercetin	Collagen alpha-1(III) chain	COL3A1
Astragali	MOL000098	quercetin	C-X-C motif chemokine 11	CXCL11
Astragali	MOL000098	quercetin	C-X-C motif chemokine 2	CXCL2
Astragali	MOL000098	quercetin	DDB1- and CUL4-associated factor 5	DCAF5
Astragali	MOL000098	quercetin	Nuclear receptor subfamily 1 group I member 3	NR1I3
Astragali	MOL000098	quercetin	Serine/threonine-protein kinase Chk2	CHEK2
Astragali	MOL000098	quercetin	Insulin receptor	INSR
Astragali	MOL000098	quercetin	Claudin-4	CLDN4
Astragali	MOL000098	quercetin	Peroxisome proliferator-activated receptor alpha	PPARA
Astragali	MOL000098	quercetin	Peroxisome proliferator-activated receptor delta	PPARD

Astragali	MOL000098	quercetin	Heat shock factor protein 1	HSF1
Astragali	MOL000098	quercetin	C-reactive protein	CRP
Astragali	MOL000098	quercetin	C-X-C motif chemokine 10	CXCL10
Astragali	MOL000098	quercetin	Inhibitor of nuclear factor kappa-B kinase subunit alpha	CHUK
Astragali	MOL000098	quercetin	Osteopontin	SPP1
Astragali	MOL000098	quercetin	Runt-related transcription factor 2	RUNX2
Astragali	MOL000098	quercetin	Ras association domain-containing protein 1	RASSF1
Astragali	MOL000098	quercetin	Transcription factor E2F1	E2F1
Astragali	MOL000098	quercetin	Transcription factor E2F2	E2F2
Astragali	MOL000098	quercetin	Prostatic acid phosphatase	ACPP
Astragali	MOL000098	quercetin	Cathepsin D	CTSD
Astragali	MOL000098	quercetin	Insulin-like growth factor-binding protein 3	IGFBP3
Astragali	MOL000098	quercetin	Insulin-like growth factor II	IGF2
Astragali	MOL000098	quercetin	CD40 ligand	CD40LG

Astragali	MOL000098	querceti n	Interferon regulatory factor 1	IRF1
Astragali	MOL000098	querceti n	Receptor tyrosine-protein kinase erbB-3	ERBB3
Astragali	MOL000098	querceti n	Serum paraoxonase/arylesterase 1	PON1
Astragali	MOL000098	querceti n	Type I iodothyronine deiodinase	DIO1
Astragali	MOL000098	querceti n	Procollagen C-endopeptidase enhancer 1	PCOLC E
Astragali	MOL000098	querceti n	Puromycin-sensitive aminopeptidase	NPEPPS
Astragali	MOL000098	querceti n	Hexokinase-2	HK2
Astragali	MOL000098	querceti n	Ras GTPase-activating protein 1	RASA1
Astragali	MOL000098	querceti n	Glutathione S-transferase Mu 1	GSTM1
Astragali	MOL000098	querceti n	Glutathione S-transferase Mu 2	GSTM2
Salviae lig	MOL007059	3-beta-Hydrox ymethyll enetansh iquinone	Muscarinic acetylcholine receptor M1	CHRM1
Salviae lig	MOL007059	3-beta-Hydrox ymethyll enetansh iquinone	Prostaglandin G/H synthase 2	PTGS2

Salviae lig	MOL007059	3-beta-Hydroxymethyllenetanshiquinone	Retinoic acid receptor RXR-alpha	RXRA
Salviae lig	MOL007059	3-beta-Hydroxymethyllenetanshiquinone	Delta-type opioid receptor	OPRD1
Salviae lig	MOL007059	3-beta-Hydroxymethyllenetanshiquinone	Acetylcholinesterase	ACHE
Salviae lig	MOL007059	3-beta-Hydroxymethyllenetanshiquinone	Alpha-1A adrenergic receptor	ADRA1A
Salviae lig	MOL007059	3-beta-Hydroxymethyllenetanshiquinone	Beta-2 adrenergic receptor	ADRB2
Salviae lig	MOL007059	3-beta-Hydroxymethyllenetanshiquinone	Mu-type opioid receptor	OPRM1

Salviae lig	MOL007059	3-beta-Hydroxymethyllenetanshiquinone	Trypsin-1	PRSS1
Eucommia	MOL007059	3-beta-Hydroxymethyllenetanshiquinone	Nuclear receptor coactivator 1	NCOA1
Eucommia	MOL002058	40957-99-1	Potassium voltage-gated channel subfamily H member 2	KCNH2
Eucommia	MOL002058	40957-99-1	Sodium channel protein type 5 subunit alpha	SCN5A
Eucommia	MOL002058	40957-99-1	Prostaglandin G/H synthase 2	PTGS2
Eucommia	MOL002058	40957-99-1	Prostaglandin G/H synthase 1	PTGS1
Eucommia	MOL002058	40957-99-1	Nuclear receptor coactivator 2	NCOA2
Eucommia	MOL002058	40957-99-1	Coagulation factor VII	F7
Eucommia	MOL000211	Mairin	Progesterone receptor	PGR
Eucommia	MOL000358	beta-sitosterol	Progesterone receptor	PGR
Eucommia	MOL000358	beta-sitosterol	Nuclear receptor coactivator 2	NCOA2

Eucommia	MOL000358	beta-sitosterol	Prostaglandin G/H synthase 1	PTGS1
Eucommia	MOL000358	beta-sitosterol	Prostaglandin G/H synthase 2	PTGS2
Eucommia	MOL000358	beta-sitosterol	Potassium voltage-gated channel subfamily H member 2	KCNH2
Eucommia	MOL000358	beta-sitosterol	Muscarinic acetylcholine receptor M3	CHRM3
Eucommia	MOL000358	beta-sitosterol	Muscarinic acetylcholine receptor M1	CHRM1
Eucommia	MOL000358	beta-sitosterol	Sodium channel protein type 5 subunit alpha	SCN5A
Eucommia	MOL000358	beta-sitosterol	Muscarinic acetylcholine receptor M4	CHRM4
Eucommia	MOL000358	beta-sitosterol	Alpha-1A adrenergic receptor	ADRA1A
Eucommia	MOL000358	beta-sitosterol	Muscarinic acetylcholine receptor M2	CHRM2
Eucommia	MOL000358	beta-sitosterol	Alpha-1B adrenergic receptor	ADRA1B

Eucommia	MOL000358	beta-sitosterol	Beta-2 adrenergic receptor	ADRB2
Eucommia	MOL000358	beta-sitosterol	Neuronal acetylcholine receptor subunit alpha-2	CHRNA2
Eucommia	MOL000358	beta-sitosterol	Sodium-dependent serotonin transporter	SLC6A4
Eucommia	MOL000358	beta-sitosterol	Mu-type opioid receptor	OPRM1
Eucommia	MOL000358	beta-sitosterol	Gamma-aminobutyric acid receptor subunit alpha-1	GABRA1
Eucommia	MOL000358	beta-sitosterol	Apoptosis regulator Bcl-2	BCL2
Eucommia	MOL000358	beta-sitosterol	Apoptosis regulator BAX	BAX
Eucommia	MOL000358	beta-sitosterol	Caspase-9	CASP9
Eucommia	MOL000358	beta-sitosterol	Transcription factor AP-1	JUN
Eucommia	MOL000358	beta-sitosterol	Caspase-3	CASP3

Eucommia	MOL000358	beta-sitosterol	Caspase-8	CASP8
Eucommia	MOL000358	beta-sitosterol	Protein kinase C alpha type	PRKCA
Eucommia	MOL000358	beta-sitosterol	Serum paraoxonase/arylesterase 1	PON1
Eucommia	MOL000358	beta-sitosterol	Microtubule-associated protein 2	MAP2
Eucommia	MOL000422	kaempferol	Nitric oxide synthase, inducible	NOS2
Eucommia	MOL000422	kaempferol	Prostaglandin G/H synthase 1	PTGS1
Eucommia	MOL000422	kaempferol	Androgen receptor	AR
Eucommia	MOL000422	kaempferol	Peroxisome proliferator activated receptor gamma	PPARG
Eucommia	MOL000422	kaempferol	Prostaglandin G/H synthase 2	PTGS2
Eucommia	MOL000422	kaempferol	Nuclear receptor coactivator 2	NCOA2
Eucommia	MOL000422	kaempferol	Trypsin-1	PRSS1
Eucommia	MOL000422	kaempferol	Progesterone receptor	PGR
Eucommia	MOL000422	kaempferol	Muscarinic acetylcholine receptor M1	CHRM1

Eucommia	MOL000422	kaempferol	Acetylcholinesterase	ACHE
Eucommia	MOL000422	kaempferol	Sodium-dependent noradrenaline transporter	SLC6A2
Eucommia	MOL000422	kaempferol	Muscarinic acetylcholine receptor M2	CHRM2
Eucommia	MOL000422	kaempferol	Alpha-1B adrenergic receptor	ADRA1B
Eucommia	MOL000422	kaempferol	Gamma-aminobutyric acid receptor subunit alpha-1	GABRA1
Eucommia	MOL000422	kaempferol	Coagulation factor VII	F7
Eucommia	MOL000422	kaempferol	Transcription factor p65	RELA
Eucommia	MOL000422	kaempferol	Inhibitor of nuclear factor kappa-B kinase subunit beta	IKBKB
Eucommia	MOL000422	kaempferol	RAC-alpha serine/threonine-protein kinase	AKT1
Eucommia	MOL000422	kaempferol	Apoptosis regulator Bcl-2	BCL2
Eucommia	MOL000422	kaempferol	Apoptosis regulator BAX	BAX
Eucommia	MOL000422	kaempferol	Tumor necrosis factor	TNFSF15
Eucommia	MOL000422	kaempferol	Transcription factor AP-1	JUN
Eucommia	MOL000422	kaempferol	Activator of 90 kDa heat shock protein ATPase homolog 1	AHSA1

Eucommia	MOL000422	kaempferol	Caspase-3	CASP3
Eucommia	MOL000422	kaempferol	Mitogen-activated protein kinase 8	MAPK8
Eucommia	MOL000422	kaempferol	Interstitial collagenase	MMP1
Eucommia	MOL000422	kaempferol	Signal transducer and activator of transcription 1-alpha/beta	STAT1
Eucommia	MOL000422	kaempferol	Peroxisome proliferator-activated receptor gamma	PPARG
Eucommia	MOL000422	kaempferol	Heme oxygenase 1	HMOX1
Eucommia	MOL000422	kaempferol	Cytochrome P450 3A4	CYP3A4
Eucommia	MOL000422	kaempferol	Cytochrome P450 1A2	CYP1A2
Eucommia	MOL000422	kaempferol	Cytochrome P450 1A1	CYP1A1
Eucommia	MOL000422	kaempferol	Intercellular adhesion molecule 1	ICAM1
Eucommia	MOL000422	kaempferol	E-selectin	SELE
Eucommia	MOL000422	kaempferol	Vascular cell adhesion protein 1	VCAM1
Eucommia	MOL000422	kaempferol	Nuclear receptor subfamily 1 group I member 2	NR1I2
Eucommia	MOL000422	kaempferol	Cytochrome P450 1B1	CYP1B1

Eucommia	MOL000422	kaempferol	Arachidonate 5-lipoxygenase	ALOX5
Eucommia	MOL000422	kaempferol	Hyaluronan synthase 2	HAS2
Eucommia	MOL000422	kaempferol	Glutathione S-transferase P	GSTP1
Eucommia	MOL000422	kaempferol	Aryl hydrocarbon receptor	AHR
Eucommia	MOL000422	kaempferol	26S proteasome non-ATPase regulatory subunit 3	PSMD3
Eucommia	MOL000422	kaempferol	Solute carrier family 2, facilitated glucose transporter member 4	SLC2A4
Eucommia	MOL000422	kaempferol	Nuclear receptor subfamily 1 group I member 3	NR1I3
Eucommia	MOL000422	kaempferol	Insulin receptor	INSR
Eucommia	MOL000422	kaempferol	Type I iodothyronine deiodinase	DIO1
Eucommia	MOL000422	kaempferol	Serine/threonine-protein phosphatase 2B catalytic subunit alpha isoform	PPP3CA
Eucommia	MOL000422	kaempferol	Glutathione S-transferase Mu 1	GSTM1
Eucommia	MOL000422	kaempferol	Glutathione S-transferase Mu 2	GSTM2
Eucommia	MOL000422	kaempferol	Aldo-keto reductase family 1 member C3	AKR1C3

Eucommia	MOL000422	kaempferol	Antileukoproteinase	SLPI
Eucommia	MOL004367	olivil	Prostaglandin G/H synthase 2	PTGS2
Eucommia	MOL004367	olivil	Nuclear receptor coactivator 2	NCOA2
Eucommia	MOL000443	Erythraline	Prostaglandin G/H synthase 1	PTGS1
Eucommia	MOL000443	Erythraline	Muscarinic acetylcholine receptor M3	CHRM3
Eucommia	MOL000443	Erythraline	Muscarinic acetylcholine receptor M1	CHRM1
Eucommia	MOL000443	Erythraline	Sodium channel protein type 5 subunit alpha	SCN5A
Eucommia	MOL000443	Erythraline	Muscarinic acetylcholine receptor M5	CHRM5
Eucommia	MOL000443	Erythraline	Prostaglandin G/H synthase 2	PTGS2
Eucommia	MOL000443	Erythraline	Muscarinic acetylcholine receptor M4	CHRM4
Eucommia	MOL000443	Erythraline	Retinoic acid receptor RXR-alpha	RXRA
Eucommia	MOL000443	Erythraline	Delta-type opioid receptor	OPRD1
Eucommia	MOL000443	Erythraline	Muscarinic acetylcholine receptor M2	CHRM2
Eucommia	MOL000443	Erythraline	Alpha-2B adrenergic receptor	ADRA2B
Eucommia	MOL000443	Erythraline	Alpha-1B adrenergic receptor	ADRA1B

Eucommia	MOL000443	Erythraline	Beta-2 adrenergic receptor	ADRB2
Eucommia	MOL000443	Erythraline	Alpha-1D adrenergic receptor	ADRA1D
Eucommia	MOL000443	Erythraline	Neuronal acetylcholine receptor subunit alpha-2	CHRNA2
Eucommia	MOL000443	Erythraline	Mu-type opioid receptor	OPRM1
Eucommia	MOL000443	Erythraline	Gamma-aminobutyric acid receptor subunit alpha-1	GABRA1
Eucommia	MOL006709	AIDS214634	Prostaglandin G/H synthase 2	PTGS2
Eucommia	MOL006709	AIDS214634	Nuclear receptor coactivator 2	NCOA2
Eucommia	MOL006709	AIDS214634	Potassium voltage-gated channel subfamily H member 2	KCNH2
Eucommia	MOL006709	AIDS214634	Coagulation factor VII	F7
Eucommia	MOL007059	3-beta-Hydroxymethyllenetanshiquinone	Muscarinic acetylcholine receptor M1	CHRM1
Eucommia	MOL007059	3-beta-Hydroxymethyllenetanshiquinone	Prostaglandin G/H synthase 2	PTGS2

Eucommia	MOL007059	3-beta-Hydroxymethyllenetanshiquinone	Retinoic acid receptor RXR-alpha	RXRA
Eucommia	MOL007059	3-beta-Hydroxymethyllenetanshiquinone	Delta-type opioid receptor	OPRD1
Eucommia	MOL007059	3-beta-Hydroxymethyllenetanshiquinone	Acetylcholinesterase	ACHE
Eucommia	MOL007059	3-beta-Hydroxymethyllenetanshiquinone	Alpha-1A adrenergic receptor	ADRA1A
Eucommia	MOL007059	3-beta-Hydroxymethyllenetanshiquinone	Beta-2 adrenergic receptor	ADRB2
Eucommia	MOL007059	3-beta-Hydroxymethyllenetanshiquinone	Mu-type opioid receptor	OPRM1

Eucommia	MOL007059	3-beta-Hydroxymethyllenetanshiquinone	Trypsin-1	PRSS1
Eucommia	MOL007059	3-beta-Hydroxymethyllenetanshiquinone	Nuclear receptor coactivator 1	NCOA1
Eucommia	MOL000073	ent-Epicatechin	Prostaglandin G/H synthase 1	PTGS1
Eucommia	MOL000073	ent-Epicatechin	Estrogen receptor	ESR1
Eucommia	MOL000073	ent-Epicatechin	Prostaglandin G/H synthase 2	PTGS2
Eucommia	MOL007563	Yangambin	Potassium voltage-gated channel subfamily H member 2	KCNH2
Eucommia	MOL007563	Yangambin	Sodium channel protein type 5 subunit alpha	SCN5A
Eucommia	MOL007563	Yangambin	Prostaglandin G/H synthase 2	PTGS2
Eucommia	MOL007563	Yangambin	Voltage-dependent L-type calcium channel subunit alpha-1S	CACNA1S

Eucommia	MOL007563	Yangambin	Nuclear receptor coactivator 2	NCOA2
Eucommia	MOL009009	(+)-medioresinol	Potassium voltage-gated channel subfamily H member 2	KCNH2
Eucommia	MOL009009	(+)-medioresinol	Sodium channel protein type 5 subunit alpha	SCN5A
Eucommia	MOL009009	(+)-medioresinol	Prostaglandin G/H synthase 2	PTGS2
Eucommia	MOL009009	(+)-medioresinol	Coagulation factor VII	F7
Eucommia	MOL009009	(+)-medioresinol	Alpha-1B adrenergic receptor	ADRA1B
Eucommia	MOL009009	(+)-medioresinol	Nuclear receptor coactivator 2	NCOA2
Eucommia	MOL009015	(-)-Tabernemontanine	Prostaglandin G/H synthase 1	PTGS1
Eucommia	MOL009015	(-)-Tabernemontanine	Muscarinic acetylcholine receptor M3	CHRM3

Eucommia	MOL009015	(-)-Tabernemontanine	Potassium voltage-gated channel subfamily H member 2	KCNH2
Eucommia	MOL009015	(-)-Tabernemontanine	Muscarinic acetylcholine receptor M1	CHRM1
Eucommia	MOL009015	(-)-Tabernemontanine	Androgen receptor	AR
Eucommia	MOL009015	(-)-Tabernemontanine	D(1B) dopamine receptor	DRD5
Eucommia	MOL009015	(-)-Tabernemontanine	Sodium channel protein type 5 subunit alpha	SCN5A
Eucommia	MOL009015	(-)-Tabernemontanine	Muscarinic acetylcholine receptor M5	CHRM5
Eucommia	MOL009015	(-)-Tabernemontanine	Prostaglandin G/H synthase 2	PTGS2

Eucommia	MOL009015	(-)-Tabernemontanine	Alpha-2A adrenergic receptor	ADRA2A
Eucommia	MOL009015	(-)-Tabernemontanine	5-hydroxytryptamine receptor 3A	HTR3A
Eucommia	MOL009015	(-)-Tabernemontanine	Muscarinic acetylcholine receptor M4	CHRM4
Eucommia	MOL009015	(-)-Tabernemontanine	Delta-type opioid receptor	OPRD1
Eucommia	MOL009015	(-)-Tabernemontanine	Acetylcholinesterase	ACHE
Eucommia	MOL009015	(-)-Tabernemontanine	Sodium-dependent noradrenaline transporter	SLC6A2
Eucommia	MOL009015	(-)-Tabernemontanine	Alpha-1A adrenergic receptor	ADRA1A

Eucommia	MOL009015	(-)-Tabernemontanine	Muscarinic acetylcholine receptor M2	CHRM2
Eucommia	MOL009015	(-)-Tabernemontanine	Alpha-2B adrenergic receptor	ADRA2B
Eucommia	MOL009015	(-)-Tabernemontanine	Alpha-1B adrenergic receptor	ADRA1B
Eucommia	MOL009015	(-)-Tabernemontanine	D(3) dopamine receptor	DRD3
Eucommia	MOL009015	(-)-Tabernemontanine	Beta-2 adrenergic receptor	ADRB2
Eucommia	MOL009015	(-)-Tabernemontanine	Alpha-1D adrenergic receptor	ADRA1D
Eucommia	MOL009015	(-)-Tabernemontanine	Neuronal acetylcholine receptor subunit alpha-2	CHRNA2

Eucommia	MOL009015	(-)-Tabernemontanine	Sodium-dependent serotonin transporter	SLC6A4
Eucommia	MOL009015	(-)-Tabernemontanine	D(2) dopamine receptor	DRD2
Eucommia	MOL009015	(-)-Tabernemontanine	Mu-type opioid receptor	OPRM1
Eucommia	MOL009015	(-)-Tabernemontanine	Gamma-aminobutyric acid receptor subunit alpha-1	GABRA1
Eucommia	MOL009015	(-)-Tabernemontanine	cAMP and cAMP-inhibited cGMP 3',5'-cyclic phosphodiesterase 10A	PDE10A
Eucommia	MOL009027	Cyclopamine	Androgen receptor	AR
Eucommia	MOL009027	Cyclopamine	Mineralocorticoid receptor	NR3C2
Eucommia	MOL009027	Cyclopamine	Glucocorticoid receptor	NR3C1

Eucommia	MOL009029	Dehydrodiconiferyl alcohol 4,gamma'-di-O-beta-D-glucopyanoside_qt	Prostaglandin G/H synthase 1	PTGS1
Eucommia	MOL009029	Dehydrodiconiferyl alcohol 4,gamma'-di-O-beta-D-glucopyanoside_qt	Potassium voltage-gated channel subfamily H member 2	KCNH2
Eucommia	MOL009029	Dehydrodiconiferyl alcohol 4,gamma'-di-O-beta-D-glucopyanoside_qt	Estrogen receptor	ESR1

Eucommia	MOL009029	Dehydrodiconiferyl alcohol 4,gamma'-di-O-beta-D-glucopyanoside_qt	Prostaglandin G/H synthase 2	PTGS2
Eucommia	MOL009029	Dehydrodiconiferyl alcohol 4,gamma'-di-O-beta-D-glucopyanoside_qt	Coagulation factor VII	F7
Eucommia	MOL009029	Dehydrodiconiferyl alcohol 4,gamma'-di-O-beta-D-glucopyanoside_qt	Acetylcholinesterase	ACHE

Eucommia	MOL009029	Dehydrodiconiferyl alcohol 4,gamma'-di-O-beta-D-glucopyanoside_qt	Trypsin-1	PRSS1
Eucommia	MOL009029	Dehydrodiconiferyl alcohol 4,gamma'-di-O-beta-D-glucopyanoside_qt	Cyclin-A2	CCNA2
Eucommia	MOL009031	Cinchonan-9-al, 6'-methoxy-, (9R)-	Prostaglandin G/H synthase 1	PTGS1
Eucommia	MOL009031	Cinchonan-9-al, 6'-methoxy-, (9R)-	Muscarinic acetylcholine receptor M3	CHRM3

Eucommia	MOL009031	Cinchonan-9-al, 6'-methoxy-, (9R)-	Potassium voltage-gated channel subfamily H member 2	KCNH2
Eucommia	MOL009031	Cinchonan-9-al, 6'-methoxy-, (9R)-	Muscarinic acetylcholine receptor M1	CHRM1
Eucommia	MOL009031	Cinchonan-9-al, 6'-methoxy-, (9R)-	Beta-1 adrenergic receptor	ADRB1
Eucommia	MOL009031	Cinchonan-9-al, 6'-methoxy-, (9R)-	Sodium channel protein type 5 subunit alpha	SCN5A
Eucommia	MOL009031	Cinchonan-9-al, 6'-methoxy-, (9R)-	Muscarinic acetylcholine receptor M5	CHRM5
Eucommia	MOL009031	Cinchonan-9-al, 6'-methoxy-, (9R)-	Prostaglandin G/H synthase 2	PTGS2

Eucommia	MOL009031	Cinchonan-9-al, 6'-methoxy-, (9R)-	Alpha-2A adrenergic receptor	ADRA2A
Eucommia	MOL009031	Cinchonan-9-al, 6'-methoxy-, (9R)-	5-hydroxytryptamine receptor 3A	HTR3A
Eucommia	MOL009031	Cinchonan-9-al, 6'-methoxy-, (9R)-	Alpha-2C adrenergic receptor	ADRA2C
Eucommia	MOL009031	Cinchonan-9-al, 6'-methoxy-, (9R)-	D(4) dopamine receptor	DRD4
Eucommia	MOL009031	Cinchonan-9-al, 6'-methoxy-, (9R)-	Muscarinic acetylcholine receptor M4	CHRM4
Eucommia	MOL009031	Cinchonan-9-al, 6'-methoxy-, (9R)-	Retinoic acid receptor RXR-alpha	RXRA

Eucommia	MOL009031	Cinchonan-9-al, 6'-methoxy-, (9R)-	Delta-type opioid receptor	OPRD1
Eucommia	MOL009031	Cinchonan-9-al, 6'-methoxy-, (9R)-	Sodium-dependent noradrenaline transporter	SLC6A2
Eucommia	MOL009031	Cinchonan-9-al, 6'-methoxy-, (9R)-	Alpha-1A adrenergic receptor	ADRA1A
Eucommia	MOL009031	Cinchonan-9-al, 6'-methoxy-, (9R)-	Muscarinic acetylcholine receptor M2	CHRM2
Eucommia	MOL009031	Cinchonan-9-al, 6'-methoxy-, (9R)-	Alpha-2B adrenergic receptor	ADRA2B
Eucommia	MOL009031	Cinchonan-9-al, 6'-methoxy-, (9R)-	Alpha-1B adrenergic receptor	ADRA1B

Eucommia	MOL009031	Cinchonan-9-al, 6'-methoxy-, (9R)-	D(3) dopamine receptor	DRD3
Eucommia	MOL009031	Cinchonan-9-al, 6'-methoxy-, (9R)-	Sodium-dependent dopamine transporter	SLC6A3
Eucommia	MOL009031	Cinchonan-9-al, 6'-methoxy-, (9R)-	Beta-2 adrenergic receptor	ADRB2
Eucommia	MOL009031	Cinchonan-9-al, 6'-methoxy-, (9R)-	Alpha-1D adrenergic receptor	ADRA1D
Eucommia	MOL009031	Cinchonan-9-al, 6'-methoxy-, (9R)-	Sodium-dependent serotonin transporter	SLC6A4
Eucommia	MOL009031	Cinchonan-9-al, 6'-methoxy-, (9R)-	D(2) dopamine receptor	DRD2

Eucommia	MOL009031	Cinchonan-9-al, 6'-methoxy-, (9R)-	Epidermal growth factor receptor	EGFR
Eucommia	MOL009031	Cinchonan-9-al, 6'-methoxy-, (9R)-	Mu-type opioid receptor	OPRM1
Eucommia	MOL009042	Helenalin	Gamma-aminobutyric acid receptor subunit alpha-1	GABRA1
Eucommia	MOL009047	(+)-Eudesmin	Muscarinic acetylcholine receptor M3	CHRM3
Eucommia	MOL009047	(+)-Eudesmin	Potassium voltage-gated channel subfamily H member 2	KCNH2
Eucommia	MOL009047	(+)-Eudesmin	Sodium channel protein type 5 subunit alpha	SCN5A
Eucommia	MOL009047	(+)-Eudesmin	Prostaglandin G/H synthase 2	PTGS2
Eucommia	MOL009047	(+)-Eudesmin	Alpha-1B adrenergic receptor	ADRA1B
Eucommia	MOL009047	(+)-Eudesmin	Beta-2 adrenergic receptor	ADRB2

Eucommia	MOL009047	(+)-Eudesmin	Nuclear receptor coactivator 2	NCOA2
Eucommia	MOL009053	4-[(2S,3R)-5-[(E)-3-hydroxyprop-1-enyl]-7-methoxy-3-methylol-2,3-dihydrobenzofuran-2-yl]-2-methoxy-phenol	Potassium voltage-gated channel subfamily H member 2	KCNH2

Eucommia	MOL009053	4-[(2S,3R)-5-[(E)-3-hydroxyprop-1-enyl]-7-methoxy-3-methylol-2,3-dihydrobenzofuran-2-yl]-2-methoxy-phenol	Estrogen receptor	ESR1

Eucommia	MOL009053	4-[(2S,3R)-5-[(E)-3-hydroxyprop-1-enyl]-7-methoxy-3-methylol-2,3-dihydrobenzofuran-2-yl]-2-methoxy-phenol	Prostaglandin G/H synthase 2	PTGS2

Eucommia	MOL009053	4-[(2S,3R)-5-[(E)-3-hydroxyprop-1-enyl]-7-methoxy-3-methylol-2,3-dihydrobenzofuran-2-yl]-2-methoxy-phenol	Trypsin-1	PRSS1

Eucommia	MOL009053	4-[(2S,3R)-5-[(E)-3-hydroxyprop-1-enyl]-7-methoxy-3-methylol-2,3-dihydrobenzofuran-2-yl]-2-methoxy-phenol	Acetylcholinesterase	ACHE
Eucommia	MOL009055	hirsutin_qt	Nitric oxide synthase, inducible	NOS2
Eucommia	MOL009055	hirsutin_qt	Prostaglandin G/H synthase 1	PTGS1
Eucommia	MOL009055	hirsutin_qt	Sodium channel protein type 5 subunit alpha	SCN5A
Eucommia	MOL009055	hirsutin_qt	Prostaglandin G/H synthase 2	PTGS2
Eucommia	MOL009055	hirsutin_qt	Coagulation factor VII	F7
Eucommia	MOL009055	hirsutin_qt	Vascular endothelial growth factor receptor 2	KDR

Eucommia	MOL009055	hirsutin_qt	Estrogen receptor beta	ESR2
Eucommia	MOL009055	hirsutin_qt	Mitogen-activated protein kinase 14	MAPK14
Eucommia	MOL009055	hirsutin_qt	Glycogen synthase kinase-3 beta	GSK3B
Eucommia	MOL009055	hirsutin_qt	Nuclear receptor coactivator 2	NCOA2
Eucommia	MOL009057	liriodendrin_qt	Nuclear receptor coactivator 2	NCOA2
Eucommia	MOL000098	quercetin	Prostaglandin G/H synthase 1	PTGS1
Eucommia	MOL000098	quercetin	Androgen receptor	AR
Eucommia	MOL000098	quercetin	Peroxisome proliferator activated receptor gamma	PPARG
Eucommia	MOL000098	quercetin	Prostaglandin G/H synthase 2	PTGS2
Eucommia	MOL000098	quercetin	Nuclear receptor coactivator 2	NCOA2
Eucommia	MOL000098	quercetin	Aldose reductase	AKR1B1
Eucommia	MOL000098	quercetin	Trypsin-1	PRSS1
Eucommia	MOL000098	quercetin	Potassium voltage-gated channel subfamily H member 2	KCNH2
Eucommia	MOL000098	quercetin	Sodium channel protein type 5 subunit alpha	SCN5A

Eucommia	MOL000098	querceti n	Beta-2 adrenergic receptor	ADRB2
Eucommia	MOL000098	querceti n	Stromelysin-1	MMP3
Eucommia	MOL000098	querceti n	Coagulation factor VII	F7
Eucommia	MOL000098	querceti n	Retinoic acid receptor RXR-alpha	RXRA
Eucommia	MOL000098	querceti n	Acetylcholinesterase	ACHE
Eucommia	MOL000098	querceti n	Gamma-aminobutyric acid receptor subunit alpha-1	GABRA 1
Eucommia	MOL000098	querceti n	Amine oxidase [flavin-containing] B	MAOB
Eucommia	MOL000098	querceti n	Transcription factor p65	RELA
Eucommia	MOL000098	querceti n	Epidermal growth factor receptor	EGFR
Eucommia	MOL000098	querceti n	RAC-alpha serine/threonine-protein kinase	AKT1
Eucommia	MOL000098	querceti n	Vascular endothelial growth factor A	VEGFA
Eucommia	MOL000098	querceti n	G1/S-specific cyclin-D1	CCND1
Eucommia	MOL000098	querceti n	Apoptosis regulator Bcl-2	BCL2
Eucommia	MOL000098	querceti n	Bcl-2-like protein 1	BCL2L1

Eucommia	MOL000098	quercetin	Proto-oncogene c-Fos	FOS
Eucommia	MOL000098	quercetin	Cyclin-dependent kinase inhibitor 1	CDKN1A
Eucommia	MOL000098	quercetin	Eukaryotic translation initiation factor 6	EIF6
Eucommia	MOL000098	quercetin	Apoptosis regulator BAX	BAX
Eucommia	MOL000098	quercetin	Caspase-9	CASP9
Eucommia	MOL000098	quercetin	Urokinase-type plasminogen activator	PLAU
Eucommia	MOL000098	quercetin	72 kDa type IV collagenase	MMP2
Eucommia	MOL000098	quercetin	Matrix metalloproteinase-9	MMP9
Eucommia	MOL000098	quercetin	Mitogen-activated protein kinase 1	MAPK1
Eucommia	MOL000098	quercetin	Interleukin-10	IL10
Eucommia	MOL000098	quercetin	Pro-epidermal growth factor	EGF
Eucommia	MOL000098	quercetin	Retinoblastoma-associated protein	RB1
Eucommia	MOL000098	quercetin	Tumor necrosis factor	TNFSF15
Eucommia	MOL000098	quercetin	Transcription factor AP-1	JUN
Eucommia	MOL000098	quercetin	Interleukin-6	IL6

Eucommia	MOL000098	querceti n	Activator of 90 kDa heat shock protein ATPase homolog 1	AHSA1
Eucommia	MOL000098	querceti n	Caspase-3	CASP3
Eucommia	MOL000098	querceti n	Cellular tumor antigen p53	TP63
Eucommia	MOL000098	querceti n	ETS domain-containing protein Elk-1	ELK1
Eucommia	MOL000098	querceti n	NF-kappa-B inhibitor alpha	NFKBI A
Eucommia	MOL000098	querceti n	NADPH--cytochrome P450 reductase	POR
Eucommia	MOL000098	querceti n	Ornithine decarboxylase	ODC1
Eucommia	MOL000098	querceti n	Caspase-8	CASP8
Eucommia	MOL000098	querceti n	DNA topoisomerase 1	TOP1
Eucommia	MOL000098	querceti n	RAF proto-oncogene serine/threonine-protein kinase	RAF1
Eucommia	MOL000098	querceti n	Superoxide dismutase [Cu-Zn]	SOD1
Eucommia	MOL000098	querceti n	Protein kinase C alpha type	PRKCA
Eucommia	MOL000098	querceti n	Interstitial collagenase	MMP1
Eucommia	MOL000098	querceti n	Hypoxia-inducible factor 1-alpha	HIF1A

Eucommia	MOL000098	querceti n	Signal transducer and activator of transcription 1-alpha/beta	STAT1
Eucommia	MOL000098	querceti n	Protein CBFA2T1	RUNX1 T1
Eucommia	MOL000098	querceti n	Receptor tyrosine-protein kinase erbB-2	ERBB2
Eucommia	MOL000098	querceti n	Peroxisome proliferator-activated receptor gamma	PPARG
Eucommia	MOL000098	querceti n	Acetyl-CoA carboxylase 1	ACACA
Eucommia	MOL000098	querceti n	Heme oxygenase 1	HMOX1
Eucommia	MOL000098	querceti n	Cytochrome P450 3A4	CYP3A 4
Eucommia	MOL000098	querceti n	Cytochrome P450 1A2	CYP1A 2
Eucommia	MOL000098	querceti n	Caveolin-1	CAV1
Eucommia	MOL000098	querceti n	Myc proto-oncogene protein	MYC
Eucommia	MOL000098	querceti n	Tissue factor	F3
Eucommia	MOL000098	querceti n	Gap junction alpha-1 protein	GJA1
Eucommia	MOL000098	querceti n	Cytochrome P450 1A1	CYP1A 1
Eucommia	MOL000098	querceti n	Intercellular adhesion molecule 1	ICAM1

Eucommia	MOL000098	quercetin	Interleukin-1 beta	IL1B
Eucommia	MOL000098	quercetin	C-C motif chemokine 2	CCL2
Eucommia	MOL000098	quercetin	E-selectin	SELE
Eucommia	MOL000098	quercetin	Vascular cell adhesion protein 1	VCAM1
Eucommia	MOL000098	quercetin	Prostaglandin E2 receptor EP3 subtype	PTGER3
Eucommia	MOL000098	quercetin	Interleukin-8	CXCL8
Eucommia	MOL000098	quercetin	Protein kinase C beta type	PRKCB
Eucommia	MOL000098	quercetin	Baculoviral IAP repeat-containing protein 5	BIRC5
Eucommia	MOL000098	quercetin	Dual oxidase 2	DUOX2
Eucommia	MOL000098	quercetin	Nitric oxide synthase, endothelial	NOS3
Eucommia	MOL000098	quercetin	Heat shock protein beta-1	HSPB1
Eucommia	MOL000098	quercetin	Estrogen sulfotransferase	SULT1E1
Eucommia	MOL000098	quercetin	Maltase-glucoamylase, intestinal	MGAM
Eucommia	MOL000098	quercetin	Interleukin-2	IL2
Eucommia	MOL000098	quercetin	Nuclear receptor subfamily 1 group I member 2	NR1I2

Eucommia	MOL000098	quercetin	Cytochrome P450 1B1	CYP1B1
Eucommia	MOL000098	quercetin	G2/mitotic-specific cyclin-B1	CCNB1
Eucommia	MOL000098	quercetin	Tissue-type plasminogen activator	PLAT
Eucommia	MOL000098	quercetin	Thrombomodulin	THBD
Eucommia	MOL000098	quercetin	Plasminogen activator inhibitor 1	SERPINE1
Eucommia	MOL000098	quercetin	Collagen alpha-1(I) chain	COL1A1
Eucommia	MOL000098	quercetin	Interferon gamma	IFNG
Eucommia	MOL000098	quercetin	Arachidonate 5-lipoxygenase	ALOX5
Eucommia	MOL000098	quercetin	Interleukin-1 alpha	IL1A
Eucommia	MOL000098	quercetin	Myeloperoxidase	MPO
Eucommia	MOL000098	quercetin	DNA topoisomerase 2-alpha	TOP2A
Eucommia	MOL000098	quercetin	Neutrophil cytosol factor 1	NCF1
Eucommia	MOL000098	quercetin	ATP-binding cassette sub-family G member 2	ABCG2
Eucommia	MOL000098	quercetin	Hyaluronan synthase 2	HAS2
Eucommia	MOL000098	quercetin	Glutathione S-transferase P	GSTP1

Eucommia	MOL000098	querceti n	Nuclear factor erythroid 2-related factor 2	NFE2L2
Eucommia	MOL000098	querceti n	NAD(P)H dehydrogenase [quinone] 1	NQO1
Eucommia	MOL000098	querceti n	Poly [ADP-ribose] polymerase 1	PARP1
Eucommia	MOL000098	querceti n	Aryl hydrocarbon receptor	AHR
Eucommia	MOL000098	querceti n	26S proteasome non-ATPase regulatory subunit 3	PSMD3
Eucommia	MOL000098	querceti n	Solute carrier family 2, facilitated glucose transporter member 4	SLC2A4
Eucommia	MOL000098	querceti n	Collagen alpha-1(III) chain	COL3A 1
Eucommia	MOL000098	querceti n	C-X-C motif chemokine 11	CXCL11
Eucommia	MOL000098	querceti n	C-X-C motif chemokine 2	CXCL2
Eucommia	MOL000098	querceti n	DDB1- and CUL4-associated factor 5	DCAF5
Eucommia	MOL000098	querceti n	Nuclear receptor subfamily 1 group I member 3	NR1I3
Eucommia	MOL000098	querceti n	Serine/threonine-protein kinase Chk2	CHEK2
Eucommia	MOL000098	querceti n	Insulin receptor	INSR
Eucommia	MOL000098	querceti n	Claudin-4	CLDN4

Eucommia	MOL000098	quercetin	Peroxisome proliferator-activated receptor alpha	PPARA
Eucommia	MOL000098	quercetin	Peroxisome proliferator-activated receptor delta	PPARD
Eucommia	MOL000098	quercetin	Heat shock factor protein 1	HSF1
Eucommia	MOL000098	quercetin	C-reactive protein	CRP
Eucommia	MOL000098	quercetin	C-X-C motif chemokine 10	CXCL10
Eucommia	MOL000098	quercetin	Inhibitor of nuclear factor kappa-B kinase subunit alpha	CHUK
Eucommia	MOL000098	quercetin	Osteopontin	SPP1
Eucommia	MOL000098	quercetin	Runt-related transcription factor 2	RUNX2
Eucommia	MOL000098	quercetin	Ras association domain-containing protein 1	RASSF1
Eucommia	MOL000098	quercetin	Transcription factor E2F1	E2F1
Eucommia	MOL000098	quercetin	Transcription factor E2F2	E2F2
Eucommia	MOL000098	quercetin	Prostatic acid phosphatase	ACPP
Eucommia	MOL000098	quercetin	Cathepsin D	CTSD
Eucommia	MOL000098	quercetin	Insulin-like growth factor-binding protein 3	IGFBP3

Eucommia	MOL000098	querceti n	Insulin-like growth factor II	IGF2
Eucommia	MOL000098	querceti n	CD40 ligand	CD40L G
Eucommia	MOL000098	querceti n	Interferon regulatory factor 1	IRF1
Eucommia	MOL000098	querceti n	Receptor tyrosine-protein kinase erbB-3	ERBB3
Eucommia	MOL000098	querceti n	Serum paraoxonase/arylesterase 1	PON1
Eucommia	MOL000098	querceti n	Type I iodothyronine deiodinase	DIO1
Eucommia	MOL000098	querceti n	Procollagen C-endopeptidase enhancer 1	PCOLC E
Eucommia	MOL000098	querceti n	Puromycin-sensitive aminopeptidase	NPEPPS
Eucommia	MOL000098	querceti n	Hexokinase-2	HK2
Eucommia	MOL000098	querceti n	Ras GTPase-activating protein 1	RASA1
Eucommia	MOL000098	querceti n	Glutathione S-transferase Mu 1	GSTM1
Eucommia	MOL000098	querceti n	Glutathione S-transferase Mu 2	GSTM2
Eucommia	MOL002773	beta-carotene	RAC-alpha serine/threonine-protein kinase	AKT1
Eucommia	MOL002773	beta-carotene	Vascular endothelial growth factor A	VEGFA

Eucommia	MOL002773	beta-carotene	Apoptosis regulator Bcl-2	BCL2
Eucommia	MOL002773	beta-carotene	Caspase-9	CASP9
Eucommia	MOL002773	beta-carotene	72 kDa type IV collagenase	MMP2
Eucommia	MOL002773	beta-carotene	Transcription factor AP-1	JUN
Eucommia	MOL002773	beta-carotene	Caspase-3	CASP3
Eucommia	MOL002773	beta-carotene	Prostaglandin G/H synthase 2	PTGS2
Eucommia	MOL002773	beta-carotene	Caspase-8	CASP8
Eucommia	MOL002773	beta-carotene	Interstitial collagenase	MMP1
Eucommia	MOL002773	beta-carotene	Heme oxygenase 1	HMOX1
Eucommia	MOL002773	beta-carotene	Cytochrome P450 3A4	CYP3A4
Eucommia	MOL002773	beta-carotene	Cytochrome P450 1A2	CYP1A2
Eucommia	MOL002773	beta-carotene	Serum albumin	ALB
Eucommia	MOL002773	beta-carotene	Caveolin-1	CAV1
Eucommia	MOL002773	beta-carotene	Catenin beta-1	CTNNB1
Eucommia	MOL002773	beta-carotene	Myc proto-oncogene protein	MYC

Eucommia	MOL002773	beta-carotene	Caspase-7	CASP7
Eucommia	MOL002773	beta-carotene	Tissue factor	F3
Eucommia	MOL002773	beta-carotene	Gap junction alpha-1 protein	GJA1
Eucommia	MOL002773	beta-carotene	Stromelysin-2	MMP10
Eucommia	MOL008240	(E)-3-[4-[(1R,2R)-2-hydroxy-2-(4-hydroxy-3-methoxy-phenyl)-1-methylol-ethoxy]-3-methoxy-phenyl]acrolein	Nitric oxide synthase, inducible	NOS2

Eucommia	MOL008240	(E)-3-[4-[(1R,2R)-2-hydroxy-2-(4-hydroxy-3-methoxy-phenyl)-1-methylol-ethoxy]-3-methoxy-phenyl]acrolein	Estrogen receptor	ESR1

Eucommia	MOL008240	(E)-3-[4-[(1R,2R)-2-hydroxy-2-(4-hydroxy-3-methoxy-phenyl)-1-methylol-ethoxy]-3-methoxy-phenyl]acrolein	Peroxisome proliferator activated receptor gamma	PPARG

Eucommia	MOL008240	(E)-3-[4-[(1R,2R)-2-hydroxy-2-(4-hydroxy-3-methoxy-phenyl)-1-methylol-ethoxy]-3-methoxy-phenyl]acrolein	Prostaglandin G/H synthase 2	PTGS2

Eucommia	MOL008240	(E)-3-[4-[(1R,2R)-2-hydroxy-2-(4-hydroxy-3-methoxy-phenyl)-1-methylol-ethoxy]-3-methoxy-phenyl]acrolein	Coagulation factor VII	F7

Eucommia	MOL008240	(E)-3-[4-[(1R,2R)-2-hydroxy-2-(4-hydroxy-3-methoxy-phenyl)-1-methylol-ethoxy]-3-methoxy-phenyl]acrolein	Mitogen-activated protein kinase 14	MAPK14

Eucommia	MOL008240	(E)-3-[4-[(1R,2R)-2-hydroxy-2-(4-hydroxy-3-methoxy-phenyl)-1-methylol-ethoxy]-3-methoxy-phenyl]acrolein	Glycogen synthase kinase-3 beta	GSK3B

Eucommia	MOL008240	(E)-3-[4-[(1R,2R)-2-hydroxy-2-(4-hydroxy-3-methoxy-phenyl)-1-methylol-ethoxy]-3-methoxy-phenyl]acrolein	Trypsin-1	PRSS1

Eucommia	MOL008240	(E)-3-[4-[(1R,2R)-2-hydroxy-2-(4-hydroxy-3-methoxy-phenyl)-1-methylol-ethoxy]-3-methoxy-phenyl]acrolein	Cyclin-A2	CCNA2
Eucommia	MOL011604	Syringetin	Nitric oxide synthase, inducible	NOS2
Eucommia	MOL011604	Syringetin	Estrogen receptor	ESR1
Eucommia	MOL011604	Syringetin	Androgen receptor	AR
Eucommia	MOL011604	Syringetin	Sodium channel protein type 5 subunit alpha	SCN5A
Eucommia	MOL011604	Syringetin	Peroxisome proliferator activated receptor gamma	PPARG

Eucommia	MOL011604	Syringet in	Prostaglandin G/H synthase 2	PTGS2
Eucommia	MOL011604	Syringet in	Coagulation factor VII	F7
Eucommia	MOL011604	Syringet in	Estrogen receptor beta	ESR2
Eucommia	MOL011604	Syringet in	Mitogen-activated protein kinase 14	MAPK1 4
Eucommia	MOL011604	Syringet in	Glycogen synthase kinase-3 beta	GSK3B
Eucommia	MOL011604	Syringet in	Trypsin-1	PRSS1
Eucommia	MOL011604	Syringet in	Cyclin-A2	CCNA2
Eucommia	MOL011604	Syringet in	Nuclear receptor coactivator 2	NCOA2
Angelicae	MOL000358	beta-sitostero l	Progesterone receptor	PGR
Angelicae	MOL000358	beta-sitostero l	Nuclear receptor coactivator 2	NCOA2
Angelicae	MOL000358	beta-sitostero l	Prostaglandin G/H synthase 1	PTGS1
Angelicae	MOL000358	beta-sitostero l	Prostaglandin G/H synthase 2	PTGS2

Angelicae	MOL000358	beta-sitosterol	Potassium voltage-gated channel subfamily H member 2	KCNH2
Angelicae	MOL000358	beta-sitosterol	Muscarinic acetylcholine receptor M3	CHRM3
Angelicae	MOL000358	beta-sitosterol	Muscarinic acetylcholine receptor M1	CHRM1
Angelicae	MOL000358	beta-sitosterol	Sodium channel protein type 5 subunit alpha	SCN5A
Angelicae	MOL000358	beta-sitosterol	Muscarinic acetylcholine receptor M4	CHRM4
Angelicae	MOL000358	beta-sitosterol	Alpha-1A adrenergic receptor	ADRA1A
Angelicae	MOL000358	beta-sitosterol	Muscarinic acetylcholine receptor M2	CHRM2
Angelicae	MOL000358	beta-sitosterol	Alpha-1B adrenergic receptor	ADRA1B
Angelicae	MOL000358	beta-sitosterol	Beta-2 adrenergic receptor	ADRB2
Angelicae	MOL000358	beta-sitosterol	Neuronal acetylcholine receptor subunit alpha-2	CHRNA2

Angelicae	MOL000358	beta-sitosterol	Sodium-dependent serotonin transporter	SLC6A4
Angelicae	MOL000358	beta-sitosterol	Mu-type opioid receptor	OPRM1
Angelicae	MOL000358	beta-sitosterol	Gamma-aminobutyric acid receptor subunit alpha-1	GABRA1
Angelicae	MOL000358	beta-sitosterol	Apoptosis regulator Bcl-2	BCL2
Angelicae	MOL000358	beta-sitosterol	Apoptosis regulator BAX	BAX
Angelicae	MOL000358	beta-sitosterol	Caspase-9	CASP9
Angelicae	MOL000358	beta-sitosterol	Transcription factor AP-1	JUN
Angelicae	MOL000358	beta-sitosterol	Caspase-3	CASP3
Angelicae	MOL000358	beta-sitosterol	Caspase-8	CASP8
Angelicae	MOL000358	beta-sitosterol	Protein kinase C alpha type	PRKCA

Angelicae	MOL000358	beta-sitosterol	Serum paraoxonase/arylesterase 1	PON1
Angelicae	MOL000358	beta-sitosterol	Microtubule-associated protein 2	MAP2
Angelicae	MOL000449	Stigmasterol	Progesterone receptor	PGR
Angelicae	MOL000449	Stigmasterol	Mineralocorticoid receptor	NR3C2
Angelicae	MOL000449	Stigmasterol	Nuclear receptor coactivator 2	NCOA2
Angelicae	MOL000449	Stigmasterol	Alcohol dehydrogenase 1C	ADH1C
Angelicae	MOL000449	Stigmasterol	Retinoic acid receptor RXR-alpha	RXRA
Angelicae	MOL000449	Stigmasterol	Nuclear receptor coactivator 1	NCOA1
Angelicae	MOL000449	Stigmasterol	Prostaglandin G/H synthase 1	PTGS1
Angelicae	MOL000449	Stigmasterol	Prostaglandin G/H synthase 2	PTGS2
Angelicae	MOL000449	Stigmasterol	Alpha-2A adrenergic receptor	ADRA2A
Angelicae	MOL000449	Stigmasterol	Sodium-dependent noradrenaline transporter	SLC6A2
Angelicae	MOL000449	Stigmasterol	Sodium-dependent dopamine transporter	SLC6A3
Angelicae	MOL000449	Stigmasterol	Beta-2 adrenergic receptor	ADRB2

Angelicae	MOL000449	Stigmasterol	Aldose reductase	AKR1B1
Angelicae	MOL000449	Stigmasterol	Urokinase-type plasminogen activator	PLAU
Angelicae	MOL000449	Stigmasterol	Leukotriene A-4 hydrolase	LTA4H
Angelicae	MOL000449	Stigmasterol	Amine oxidase [flavin-containing] B	MAOB
Angelicae	MOL000449	Stigmasterol	Amine oxidase [flavin-containing] A	MAOA
Angelicae	MOL000449	Stigmasterol	Chymotrypsinogen B	CTRB1
Angelicae	MOL000449	Stigmasterol	Muscarinic acetylcholine receptor M3	CHRM3
Angelicae	MOL000449	Stigmasterol	Muscarinic acetylcholine receptor M1	CHRM1
Angelicae	MOL000449	Stigmasterol	Beta-1 adrenergic receptor	ADRB1
Angelicae	MOL000449	Stigmasterol	Sodium channel protein type 5 subunit alpha	SCN5A
Angelicae	MOL000449	Stigmasterol	Alpha-1A adrenergic receptor	ADRA1A
Angelicae	MOL000449	Stigmasterol	Muscarinic acetylcholine receptor M2	CHRM2
Angelicae	MOL000449	Stigmasterol	Alpha-1B adrenergic receptor	ADRA1B
Angelicae	MOL000449	Stigmasterol	Gamma-aminobutyric acid receptor subunit alpha-1	GABRA1
Semen Cu	MOL001558	sesamin	Prostaglandin G/H synthase 2	PTGS2

Semen Cu	MOL001558	sesamin	Sodium channel protein type 5 subunit alpha	SCN5A
Semen Cu	MOL001558	sesamin	G1/S-specific cyclin-D1	CCND1
Semen Cu	MOL001558	sesamin	Interleukin-10	IL10
Semen Cu	MOL001558	sesamin	Fatty acid synthase	FASN
Semen Cu	MOL001558	sesamin	Acetyl-CoA carboxylase 1	ACACA
Semen Cu	MOL001558	sesamin	Glucose-6-phosphate 1-dehydrogenase	G6PD
Semen Cu	MOL001558	sesamin	Nitric oxide synthase, endothelial	NOS3
Semen Cu	MOL001558	sesamin	Endothelin-converting enzyme 1	ECE1
Semen Cu	MOL001558	sesamin	Medium-chain specific acyl-CoA dehydrogenase, mitochondrial	ACAD M
Semen Cu	MOL001558	sesamin	Cytochrome P450 2B6	CYP2B6
Semen Cu	MOL001558	sesamin	UDP-glucuronosyltransferase 1-1	UGT1A 1
Semen Cu	MOL001558	sesamin	Sterol regulatory element-binding protein 1	SREBF1
Semen Cu	MOL001558	sesamin	NADPH oxidase 3	NOX3
Semen Cu	MOL001558	sesamin	NADPH oxidase 1	NOX1
Semen Cu	MOL001558	sesamin	Peroxisomal acyl-coenzyme A oxidase 1	ACOX1
Semen Cu	MOL001558	sesamin	ATP-citrate synthase	ACLY
Semen Cu	MOL001558	sesamin	Peroxisomal bifunctional enzyme	EHHAD H
Semen Cu	MOL001558	sesamin	Methylglutaconyl-CoA hydratase, mitochondrial	AUH

Semen Cu:	MOL001558	sesamin	Trifunctional enzyme subunit beta, mitochondrial	HADHB
Semen Cu:	MOL001558	sesamin	2,4-dienoyl-CoA reductase, mitochondrial	DECR1
Semen Cu:	MOL000184	NSC63551	Progesterone receptor	PGR
Semen Cu:	MOL000354	isorhamnetin	Nitric oxide synthase, inducible	NOS2
Semen Cu:	MOL000354	isorhamnetin	Prostaglandin G/H synthase 1	PTGS1
Semen Cu:	MOL000354	isorhamnetin	Estrogen receptor	ESR1
Semen Cu:	MOL000354	isorhamnetin	Androgen receptor	AR
Semen Cu:	MOL000354	isorhamnetin	Peroxisome proliferator activated receptor gamma	PPARG
Semen Cu:	MOL000354	isorhamnetin	Prostaglandin G/H synthase 2	PTGS2
Semen Cu:	MOL000354	isorhamnetin	Estrogen receptor beta	ESR2
Semen Cu:	MOL000354	isorhamnetin	Mitogen-activated protein kinase 14	MAPK14
Semen Cu:	MOL000354	isorhamnetin	Glycogen synthase kinase-3 beta	GSK3B
Semen Cu:	MOL000354	isorhamnetin	Trypsin-1	PRSS1
Semen Cu:	MOL000354	isorhamnetin	Cyclin-A2	CCNA2
Semen Cu:	MOL000354	isorhamnetin	Nuclear receptor coactivator 2	NCOA2

Semen Cu	MOL000354	isorham netin	Glycogen phosphorylase, muscle form	PYGM
Semen Cu	MOL000354	isorham netin	Serine/threonine-protein kinase Chk1	CHEK1
Semen Cu	MOL000354	isorham netin	Aldose reductase	AKR1B 1
Semen Cu	MOL000354	isorham netin	Nuclear receptor coactivator 1	NCOA1
Semen Cu	MOL000354	isorham netin	Coagulation factor VII	F7
Semen Cu	MOL000354	isorham netin	Acetylcholinesterase	ACHE
Semen Cu	MOL000354	isorham netin	Gamma-aminobutyric acid receptor subunit alpha-1	GABRA 1
Semen Cu	MOL000354	isorham netin	Amine oxidase [flavin-containing] B	MAOB
Semen Cu	MOL000354	isorham netin	Glutamate receptor 2	GRIA2
Semen Cu	MOL000354	isorham netin	Transcription factor p65	RELA
Semen Cu	MOL000354	isorham netin	Neutrophil cytosol factor 1	NCF1
Semen Cu	MOL000354	isorham netin	Oxidized low-density lipoprotein receptor 1	OLR1
Semen Cu	MOL000358	beta-sitostero l	Progesterone receptor	PGR
Semen Cu	MOL000358	beta-sitostero l	Nuclear receptor coactivator 2	NCOA2

Semen Cu	MOL000358	beta-sitosterol	Prostaglandin G/H synthase 1	PTGS1
Semen Cu	MOL000358	beta-sitosterol	Prostaglandin G/H synthase 2	PTGS2
Semen Cu	MOL000358	beta-sitosterol	Potassium voltage-gated channel subfamily H member 2	KCNH2
Semen Cu	MOL000358	beta-sitosterol	Muscarinic acetylcholine receptor M3	CHRM3
Semen Cu	MOL000358	beta-sitosterol	Muscarinic acetylcholine receptor M1	CHRM1
Semen Cu	MOL000358	beta-sitosterol	Sodium channel protein type 5 subunit alpha	SCN5A
Semen Cu	MOL000358	beta-sitosterol	Muscarinic acetylcholine receptor M4	CHRM4
Semen Cu	MOL000358	beta-sitosterol	Alpha-1A adrenergic receptor	ADRA1A
Semen Cu	MOL000358	beta-sitosterol	Muscarinic acetylcholine receptor M2	CHRM2
Semen Cu	MOL000358	beta-sitosterol	Alpha-1B adrenergic receptor	ADRA1B

Semen Cu	MOL000358	beta-sitosterol	Beta-2 adrenergic receptor	ADRB2
Semen Cu	MOL000358	beta-sitosterol	Neuronal acetylcholine receptor subunit alpha-2	CHRNA2
Semen Cu	MOL000358	beta-sitosterol	Sodium-dependent serotonin transporter	SLC6A4
Semen Cu	MOL000358	beta-sitosterol	Mu-type opioid receptor	OPRM1
Semen Cu	MOL000358	beta-sitosterol	Gamma-aminobutyric acid receptor subunit alpha-1	GABRA1
Semen Cu	MOL000358	beta-sitosterol	Apoptosis regulator Bcl-2	BCL2
Semen Cu	MOL000358	beta-sitosterol	Apoptosis regulator BAX	BAX
Semen Cu	MOL000358	beta-sitosterol	Caspase-9	CASP9
Semen Cu	MOL000358	beta-sitosterol	Transcription factor AP-1	JUN
Semen Cu	MOL000358	beta-sitosterol	Caspase-3	CASP3

Semen Cu	MOL000358	beta-sitosterol	Caspase-8	CASP8
Semen Cu	MOL000358	beta-sitosterol	Protein kinase C alpha type	PRKCA
Semen Cu	MOL000358	beta-sitosterol	Serum paraoxonase/arylesterase 1	PON1
Semen Cu	MOL000358	beta-sitosterol	Microtubule-associated protein 2	MAP2
Semen Cu	MOL000422	kaempferol	Nitric oxide synthase, inducible	NOS2
Semen Cu	MOL000422	kaempferol	Prostaglandin G/H synthase 1	PTGS1
Semen Cu	MOL000422	kaempferol	Androgen receptor	AR
Semen Cu	MOL000422	kaempferol	Peroxisome proliferator activated receptor gamma	PPARG
Semen Cu	MOL000422	kaempferol	Prostaglandin G/H synthase 2	PTGS2
Semen Cu	MOL000422	kaempferol	Nuclear receptor coactivator 2	NCOA2
Semen Cu	MOL000422	kaempferol	Trypsin-1	PRSS1
Semen Cu	MOL000422	kaempferol	Progesterone receptor	PGR
Semen Cu	MOL000422	kaempferol	Muscarinic acetylcholine receptor M1	CHRM1

Semen Cu:	MOL000422	kaempfe rol	Acetylcholinesterase	ACHE
Semen Cu:	MOL000422	kaempfe rol	Sodium-dependent noradrenaline transporter	SLC6A2
Semen Cu:	MOL000422	kaempfe rol	Muscarinic acetylcholine receptor M2	CHRM2
Semen Cu:	MOL000422	kaempfe rol	Alpha-1B adrenergic receptor	ADRA1 B
Semen Cu:	MOL000422	kaempfe rol	Gamma-aminobutyric acid receptor subunit alpha-1	GABRA 1
Semen Cu:	MOL000422	kaempfe rol	Coagulation factor VII	F7
Semen Cu:	MOL000422	kaempfe rol	Transcription factor p65	RELA
Semen Cu:	MOL000422	kaempfe rol	Inhibitor of nuclear factor kappa-B kinase subunit beta	IKBKB
Semen Cu:	MOL000422	kaempfe rol	RAC-alpha serine/threonine-protein kinase	AKT1
Semen Cu:	MOL000422	kaempfe rol	Apoptosis regulator Bcl-2	BCL2
Semen Cu:	MOL000422	kaempfe rol	Apoptosis regulator BAX	BAX
Semen Cu:	MOL000422	kaempfe rol	Tumor necrosis factor	TNFSF1 5
Semen Cu:	MOL000422	kaempfe rol	Transcription factor AP-1	JUN
Semen Cu:	MOL000422	kaempfe rol	Activator of 90 kDa heat shock protein ATPase homolog 1	AHSA1

Semen Cu	MOL000422	kaempferol	Caspase-3	CASP3
Semen Cu	MOL000422	kaempferol	Mitogen-activated protein kinase 8	MAPK8
Semen Cu	MOL000422	kaempferol	Interstitial collagenase	MMP1
Semen Cu	MOL000422	kaempferol	Signal transducer and activator of transcription 1-alpha/beta	STAT1
Semen Cu	MOL000422	kaempferol	Peroxisome proliferator-activated receptor gamma	PPARG
Semen Cu	MOL000422	kaempferol	Heme oxygenase 1	HMOX1
Semen Cu	MOL000422	kaempferol	Cytochrome P450 3A4	CYP3A4
Semen Cu	MOL000422	kaempferol	Cytochrome P450 1A2	CYP1A2
Semen Cu	MOL000422	kaempferol	Cytochrome P450 1A1	CYP1A1
Semen Cu	MOL000422	kaempferol	Intercellular adhesion molecule 1	ICAM1
Semen Cu	MOL000422	kaempferol	E-selectin	SELE
Semen Cu	MOL000422	kaempferol	Vascular cell adhesion protein 1	VCAM1
Semen Cu	MOL000422	kaempferol	Nuclear receptor subfamily 1 group I member 2	NR1I2
Semen Cu	MOL000422	kaempferol	Cytochrome P450 1B1	CYP1B1

Semen Cu	MOL000422	kaempferol	Arachidonate 5-lipoxygenase	ALOX5
Semen Cu	MOL000422	kaempferol	Hyaluronan synthase 2	HAS2
Semen Cu	MOL000422	kaempferol	Glutathione S-transferase P	GSTP1
Semen Cu	MOL000422	kaempferol	Aryl hydrocarbon receptor	AHR
Semen Cu	MOL000422	kaempferol	26S proteasome non-ATPase regulatory subunit 3	PSMD3
Semen Cu	MOL000422	kaempferol	Solute carrier family 2, facilitated glucose transporter member 4	SLC2A4
Semen Cu	MOL000422	kaempferol	Nuclear receptor subfamily 1 group I member 3	NR1I3
Semen Cu	MOL000422	kaempferol	Insulin receptor	INSR
Semen Cu	MOL000422	kaempferol	Type I iodothyronine deiodinase	DIO1
Semen Cu	MOL000422	kaempferol	Serine/threonine-protein phosphatase 2B catalytic subunit alpha isoform	PPP3CA
Semen Cu	MOL000422	kaempferol	Glutathione S-transferase Mu 1	GSTM1
Semen Cu	MOL000422	kaempferol	Glutathione S-transferase Mu 2	GSTM2
Semen Cu	MOL000422	kaempferol	Aldo-keto reductase family 1 member C3	AKR1C3

Semen Cu	MOL000422	kaempferol	Antileukoproteinase	SLPI
Semen Cu	MOL005043	campest-5-en-3beta-ol	Progesterone receptor	PGR
Semen Cu	MOL005440	Isofucosterol	Progesterone receptor	PGR
Semen Cu	MOL005440	Isofucosterol	Mineralocorticoid receptor	NR3C2
Semen Cu	MOL005440	Isofucosterol	4-aminobutyrate aminotransferase, mitochondrial	ABAT
Semen Cu	MOL005440	Isofucosterol	Gamma-aminobutyric acid receptor subunit alpha-1	GABRA1
Semen Cu	MOL005440	Isofucosterol	Alcohol dehydrogenase 1B	ADH1B
Semen Cu	MOL005440	Isofucosterol	Alcohol dehydrogenase 1C	ADH1C
Semen Cu	MOL005440	Isofucosterol	Alcohol dehydrogenase 1A	ADH1A
Semen Cu	MOL005440	Isofucosterol	Lysozyme	LYZ
Semen Cu	MOL005440	Isofucosterol	Nuclear receptor coactivator 2	NCOA2
Semen Cu	MOL005944	matrine	Transcription factor p65	RELA
Semen Cu	MOL005944	matrine	72 kDa type IV collagenase	MMP2
Semen Cu	MOL005944	matrine	Tumor necrosis factor	TNFSF15
Semen Cu	MOL005944	matrine	Interleukin-6	IL6
Semen Cu	MOL005944	matrine	Caspase-3	CASP3

Semen Cu	MOL005944	matrine	Myc proto-oncogene protein	MYC
Semen Cu	MOL005944	matrine	Intercellular adhesion molecule 1	ICAM1
Semen Cu	MOL005944	matrine	Heparanase	HPSE
Semen Cu	MOL005944	matrine	Immediate early response 3-interacting protein 1	IER3IP1
Semen Cu	MOL005944	matrine	CD44 antigen	CD44
Semen Cu	MOL000953	CLR	Progesterone receptor	PGR
Semen Cu	MOL000953	CLR	Mineralocorticoid receptor	NR3C2
Semen Cu	MOL000953	CLR	Nuclear receptor coactivator 2	NCOA2
Semen Cu	MOL000098	quercetin	Prostaglandin G/H synthase 1	PTGS1
Semen Cu	MOL000098	quercetin	Androgen receptor	AR
Semen Cu	MOL000098	quercetin	Peroxisome proliferator activated receptor gamma	PPARG
Semen Cu	MOL000098	quercetin	Prostaglandin G/H synthase 2	PTGS2
Semen Cu	MOL000098	quercetin	Nuclear receptor coactivator 2	NCOA2
Semen Cu	MOL000098	quercetin	Aldose reductase	AKR1B1
Semen Cu	MOL000098	quercetin	Trypsin-1	PRSS1
Semen Cu	MOL000098	quercetin	Potassium voltage-gated channel subfamily H member 2	KCNH2

Semen Cu	MOL000098	quercetin	Sodium channel protein type 5 subunit alpha	SCN5A
Semen Cu	MOL000098	quercetin	Beta-2 adrenergic receptor	ADRB2
Semen Cu	MOL000098	quercetin	Stromelysin-1	MMP3
Semen Cu	MOL000098	quercetin	Coagulation factor VII	F7
Semen Cu	MOL000098	quercetin	Retinoic acid receptor RXR-alpha	RXRA
Semen Cu	MOL000098	quercetin	Acetylcholinesterase	ACHE
Semen Cu	MOL000098	quercetin	Gamma-aminobutyric acid receptor subunit alpha-1	GABRA1
Semen Cu	MOL000098	quercetin	Amine oxidase [flavin-containing] B	MAOB
Semen Cu	MOL000098	quercetin	Transcription factor p65	RELA
Semen Cu	MOL000098	quercetin	Epidermal growth factor receptor	EGFR
Semen Cu	MOL000098	quercetin	RAC-alpha serine/threonine-protein kinase	AKT1
Semen Cu	MOL000098	quercetin	Vascular endothelial growth factor A	VEGFA
Semen Cu	MOL000098	quercetin	G1/S-specific cyclin-D1	CCND1
Semen Cu	MOL000098	quercetin	Apoptosis regulator Bcl-2	BCL2

Semen Cu	MOL000098	quercetin	Bcl-2-like protein 1	BCL2L1
Semen Cu	MOL000098	quercetin	Proto-oncogene c-Fos	FOS
Semen Cu	MOL000098	quercetin	Cyclin-dependent kinase inhibitor 1	CDKN1A
Semen Cu	MOL000098	quercetin	Eukaryotic translation initiation factor 6	EIF6
Semen Cu	MOL000098	quercetin	Apoptosis regulator BAX	BAX
Semen Cu	MOL000098	quercetin	Caspase-9	CASP9
Semen Cu	MOL000098	quercetin	Urokinase-type plasminogen activator	PLAU
Semen Cu	MOL000098	quercetin	72 kDa type IV collagenase	MMP2
Semen Cu	MOL000098	quercetin	Matrix metalloproteinase-9	MMP9
Semen Cu	MOL000098	quercetin	Mitogen-activated protein kinase 1	MAPK1
Semen Cu	MOL000098	quercetin	Interleukin-10	IL10
Semen Cu	MOL000098	quercetin	Pro-epidermal growth factor	EGF
Semen Cu	MOL000098	quercetin	Retinoblastoma-associated protein	RB1
Semen Cu	MOL000098	quercetin	Tumor necrosis factor	TNFSF15
Semen Cu	MOL000098	quercetin	Transcription factor AP-1	JUN

Semen Cu	MOL000098	quercetin	Interleukin-6	IL6
Semen Cu	MOL000098	quercetin	Activator of 90 kDa heat shock protein ATPase homolog 1	AHSA1
Semen Cu	MOL000098	quercetin	Caspase-3	CASP3
Semen Cu	MOL000098	quercetin	Cellular tumor antigen p53	TP63
Semen Cu	MOL000098	quercetin	ETS domain-containing protein Elk-1	ELK1
Semen Cu	MOL000098	quercetin	NF-kappa-B inhibitor alpha	NFKBIA
Semen Cu	MOL000098	quercetin	NADPH--cytochrome P450 reductase	POR
Semen Cu	MOL000098	quercetin	Ornithine decarboxylase	ODC1
Semen Cu	MOL000098	quercetin	Caspase-8	CASP8
Semen Cu	MOL000098	quercetin	DNA topoisomerase 1	TOP1
Semen Cu	MOL000098	quercetin	RAF proto-oncogene serine/threonine-protein kinase	RAF1
Semen Cu	MOL000098	quercetin	Superoxide dismutase [Cu-Zn]	SOD1
Semen Cu	MOL000098	quercetin	Protein kinase C alpha type	PRKCA
Semen Cu	MOL000098	quercetin	Interstitial collagenase	MMP1

Semen Cu:	MOL000098	quercetin	Hypoxia-inducible factor 1-alpha	HIF1A
Semen Cu:	MOL000098	quercetin	Signal transducer and activator of transcription 1-alpha/beta	STAT1
Semen Cu:	MOL000098	quercetin	Protein CBFA2T1	RUNX1T1
Semen Cu:	MOL000098	quercetin	Receptor tyrosine-protein kinase erbB-2	ERBB2
Semen Cu:	MOL000098	quercetin	Peroxisome proliferator-activated receptor gamma	PPARG
Semen Cu:	MOL000098	quercetin	Acetyl-CoA carboxylase 1	ACACA
Semen Cu:	MOL000098	quercetin	Heme oxygenase 1	HMOX1
Semen Cu:	MOL000098	quercetin	Cytochrome P450 3A4	CYP3A4
Semen Cu:	MOL000098	quercetin	Cytochrome P450 1A2	CYP1A2
Semen Cu:	MOL000098	quercetin	Caveolin-1	CAV1
Semen Cu:	MOL000098	quercetin	Myc proto-oncogene protein	MYC
Semen Cu:	MOL000098	quercetin	Tissue factor	F3
Semen Cu:	MOL000098	quercetin	Gap junction alpha-1 protein	GJA1
Semen Cu:	MOL000098	quercetin	Cytochrome P450 1A1	CYP1A1

Semen Cu:	MOL000098	quercetin	Intercellular adhesion molecule 1	ICAM1
Semen Cu:	MOL000098	quercetin	Interleukin-1 beta	IL1B
Semen Cu:	MOL000098	quercetin	C-C motif chemokine 2	CCL2
Semen Cu:	MOL000098	quercetin	E-selectin	SELE
Semen Cu:	MOL000098	quercetin	Vascular cell adhesion protein 1	VCAM1
Semen Cu:	MOL000098	quercetin	Prostaglandin E2 receptor EP3 subtype	PTGER3
Semen Cu:	MOL000098	quercetin	Interleukin-8	CXCL8
Semen Cu:	MOL000098	quercetin	Protein kinase C beta type	PRKCB
Semen Cu:	MOL000098	quercetin	Baculoviral IAP repeat-containing protein 5	BIRC5
Semen Cu:	MOL000098	quercetin	Dual oxidase 2	DUOX2
Semen Cu:	MOL000098	quercetin	Nitric oxide synthase, endothelial	NOS3
Semen Cu:	MOL000098	quercetin	Heat shock protein beta-1	HSPB1
Semen Cu:	MOL000098	quercetin	Estrogen sulfotransferase	SULT1E1
Semen Cu:	MOL000098	quercetin	Maltase-glucoamylase, intestinal	MGAM
Semen Cu:	MOL000098	quercetin	Interleukin-2	IL2

Semen Cu	MOL000098	quercetin	Nuclear receptor subfamily 1 group I member 2	NR1I2
Semen Cu	MOL000098	quercetin	Cytochrome P450 1B1	CYP1B1
Semen Cu	MOL000098	quercetin	G2/mitotic-specific cyclin-B1	CCNB1
Semen Cu	MOL000098	quercetin	Tissue-type plasminogen activator	PLAT
Semen Cu	MOL000098	quercetin	Thrombomodulin	THBD
Semen Cu	MOL000098	quercetin	Plasminogen activator inhibitor 1	SERPINE1
Semen Cu	MOL000098	quercetin	Collagen alpha-1(I) chain	COL1A1
Semen Cu	MOL000098	quercetin	Interferon gamma	IFNG
Semen Cu	MOL000098	quercetin	Arachidonate 5-lipoxygenase	ALOX5
Semen Cu	MOL000098	quercetin	Interleukin-1 alpha	IL1A
Semen Cu	MOL000098	quercetin	Myeloperoxidase	MPO
Semen Cu	MOL000098	quercetin	DNA topoisomerase 2-alpha	TOP2A
Semen Cu	MOL000098	quercetin	Neutrophil cytosol factor 1	NCF1
Semen Cu	MOL000098	quercetin	ATP-binding cassette sub-family G member 2	ABCG2
Semen Cu	MOL000098	quercetin	Hyaluronan synthase 2	HAS2

Semen Cu:	MOL000098	querceti n	Glutathione S-transferase P	GSTP1
Semen Cu:	MOL000098	querceti n	Nuclear factor erythroid 2-related factor 2	NFE2L2
Semen Cu:	MOL000098	querceti n	NAD(P)H dehydrogenase [quinone] 1	NQO1
Semen Cu:	MOL000098	querceti n	Poly [ADP-ribose] polymerase 1	PARP1
Semen Cu:	MOL000098	querceti n	Aryl hydrocarbon receptor	AHR
Semen Cu:	MOL000098	querceti n	26S proteasome non-ATPase regulatory subunit 3	PSMD3
Semen Cu:	MOL000098	querceti n	Solute carrier family 2, facilitated glucose transporter member 4	SLC2A4
Semen Cu:	MOL000098	querceti n	Collagen alpha-1(III) chain	COL3A 1
Semen Cu:	MOL000098	querceti n	C-X-C motif chemokine 11	CXCL11
Semen Cu:	MOL000098	querceti n	C-X-C motif chemokine 2	CXCL2
Semen Cu:	MOL000098	querceti n	DDB1- and CUL4-associated factor 5	DCAF5
Semen Cu:	MOL000098	querceti n	Nuclear receptor subfamily 1 group I member 3	NR1I3
Semen Cu:	MOL000098	querceti n	Serine/threonine-protein kinase Chk2	CHEK2
Semen Cu:	MOL000098	querceti n	Insulin receptor	INSR

Semen Cu	MOL000098	quercetin	Claudin-4	CLDN4
Semen Cu	MOL000098	quercetin	Peroxisome proliferator-activated receptor alpha	PPARA
Semen Cu	MOL000098	quercetin	Peroxisome proliferator-activated receptor delta	PPARD
Semen Cu	MOL000098	quercetin	Heat shock factor protein 1	HSF1
Semen Cu	MOL000098	quercetin	C-reactive protein	CRP
Semen Cu	MOL000098	quercetin	C-X-C motif chemokine 10	CXCL10
Semen Cu	MOL000098	quercetin	Inhibitor of nuclear factor kappa-B kinase subunit alpha	CHUK
Semen Cu	MOL000098	quercetin	Osteopontin	SPP1
Semen Cu	MOL000098	quercetin	Runt-related transcription factor 2	RUNX2
Semen Cu	MOL000098	quercetin	Ras association domain-containing protein 1	RASSF1
Semen Cu	MOL000098	quercetin	Transcription factor E2F1	E2F1
Semen Cu	MOL000098	quercetin	Transcription factor E2F2	E2F2
Semen Cu	MOL000098	quercetin	Prostatic acid phosphatase	ACPP
Semen Cu	MOL000098	quercetin	Cathepsin D	CTSD

Semen Cu:	MOL000098	querceti n	Insulin-like growth factor-binding protein 3	IGFBP3
Semen Cu:	MOL000098	querceti n	Insulin-like growth factor II	IGF2
Semen Cu:	MOL000098	querceti n	CD40 ligand	CD40L G
Semen Cu:	MOL000098	querceti n	Interferon regulatory factor 1	IRF1
Semen Cu:	MOL000098	querceti n	Receptor tyrosine-protein kinase erbB-3	ERBB3
Semen Cu:	MOL000098	querceti n	Serum paraoxonase/arylesterase 1	PON1
Semen Cu:	MOL000098	querceti n	Type I iodothyronine deiodinase	DIO1
Semen Cu:	MOL000098	querceti n	Procollagen C-endopeptidase enhancer 1	PCOLC E
Semen Cu:	MOL000098	querceti n	Puromycin-sensitive aminopeptidase	NPEPPS
Semen Cu:	MOL000098	querceti n	Hexokinase-2	HK2
Semen Cu:	MOL000098	querceti n	Ras GTPase-activating protein 1	RASA1
Semen Cu:	MOL000098	querceti n	Glutathione S-transferase Mu 1	GSTM1
Semen Cu:	MOL000098	querceti n	Glutathione S-transferase Mu 2	GSTM2
Rhizoma (	MOL001454	berberin e	Nitric oxide synthase, inducible	NOS2
Rhizoma (	MOL001454	berberin e	Prostaglandin G/H synthase 1	PTGS1

Rhizoma C	MOL001454	berberin e	Potassium voltage-gated channel subfamily H member 2	KCNH2
Rhizoma C	MOL001454	berberin e	Estrogen receptor	ESR1
Rhizoma C	MOL001454	berberin e	Androgen receptor	AR
Rhizoma C	MOL001454	berberin e	Sodium channel protein type 5 subunit alpha	SCN5A
Rhizoma C	MOL001454	berberin e	Prostaglandin G/H synthase 2	PTGS2
Rhizoma C	MOL001454	berberin e	Retinoic acid receptor RXR-alpha	RXRA
Rhizoma C	MOL001454	berberin e	Beta-2 adrenergic receptor	ADRB2
Rhizoma C	MOL001454	berberin e	Trypsin-1	PRSS1
Rhizoma C	MOL001454	berberin e	Nuclear receptor coactivator 2	NCOA2
Rhizoma C	MOL001454	berberin e	cAMP and cAMP-inhibited cGMP 3',5'-cyclic phosphodiesterase 10A	PDE10A
Rhizoma C	MOL001458	coptisine	Nitric oxide synthase, inducible	NOS2
Rhizoma C	MOL001458	coptisine	Prostaglandin G/H synthase 1	PTGS1
Rhizoma C	MOL001458	coptisine	Potassium voltage-gated channel subfamily H member 2	KCNH2
Rhizoma C	MOL001458	coptisine	Estrogen receptor	ESR1

Rhizoma C	MOL001458	coptisine	Androgen receptor	AR
Rhizoma C	MOL001458	coptisine	Sodium channel protein type 5 subunit alpha	SCN5A
Rhizoma C	MOL001458	coptisine	Prostaglandin G/H synthase 2	PTGS2
Rhizoma C	MOL001458	coptisine	Trypsin-1	PRSS1
Rhizoma C	MOL001460	Cryptopin	Prostaglandin G/H synthase 1	PTGS1
Rhizoma C	MOL001460	Cryptopin	Muscarinic acetylcholine receptor M3	CHRM3
Rhizoma C	MOL001460	Cryptopin	Potassium voltage-gated channel subfamily H member 2	KCNH2
Rhizoma C	MOL001460	Cryptopin	Muscarinic acetylcholine receptor M1	CHRM1
Rhizoma C	MOL001460	Cryptopin	Sodium channel protein type 5 subunit alpha	SCN5A
Rhizoma C	MOL001460	Cryptopin	Muscarinic acetylcholine receptor M5	CHRM5
Rhizoma C	MOL001460	Cryptopin	Prostaglandin G/H synthase 2	PTGS2
Rhizoma C	MOL001460	Cryptopin	5-hydroxytryptamine receptor 3A	HTR3A
Rhizoma C	MOL001460	Cryptopin	Coagulation factor VII	F7
Rhizoma C	MOL001460	Cryptopin	Vascular endothelial growth factor receptor 2	KDR
Rhizoma C	MOL001460	Cryptopin	Muscarinic acetylcholine receptor M4	CHRM4

Rhizoma C	MOL001460	Cryptopin	Delta-type opioid receptor	OPRD1
Rhizoma C	MOL001460	Cryptopin	Alpha-1B adrenergic receptor	ADRA1B
Rhizoma C	MOL001460	Cryptopin	Beta-2 adrenergic receptor	ADRB2
Rhizoma C	MOL001460	Cryptopin	Alpha-1D adrenergic receptor	ADRA1D
Rhizoma C	MOL001460	Cryptopin	Mu-type opioid receptor	OPRM1
Rhizoma C	MOL001460	Cryptopin	Voltage-dependent L-type calcium channel subunit alpha-1S	CACNA1S
Rhizoma C	MOL001460	Cryptopin	Nuclear receptor coactivator 1	NCOA1
Rhizoma C	MOL001460	Cryptopin	cAMP and cAMP-inhibited cGMP 3',5'-cyclic phosphodiesterase 10A	PDE10A
Rhizoma C	MOL001460	Cryptopin	Sodium-dependent serotonin transporter	SLC6A4
Rhizoma C	MOL001461	Dihydrochelerythrine	Nitric oxide synthase, inducible	NOS2
Rhizoma C	MOL001461	Dihydrochelerythrine	Prostaglandin G/H synthase 1	PTGS1
Rhizoma C	MOL001461	Dihydrochelerythrine	Potassium voltage-gated channel subfamily H member 2	KCNH2

Rhizoma C	MOL001461	Dihydro cheleryt hrine	Estrogen receptor	ESR1
Rhizoma C	MOL001461	Dihydro cheleryt hrine	Androgen receptor	AR
Rhizoma C	MOL001461	Dihydro cheleryt hrine	Sodium channel protein type 5 subunit alpha	SCN5A
Rhizoma C	MOL001461	Dihydro cheleryt hrine	Peroxisome proliferator activated receptor gamma	PPARG
Rhizoma C	MOL001461	Dihydro cheleryt hrine	Prostaglandin G/H synthase 2	PTGS2
Rhizoma C	MOL001461	Dihydro cheleryt hrine	Retinoic acid receptor RXR-alpha	RXRA
Rhizoma C	MOL001461	Dihydro cheleryt hrine	Acetylcholinesterase	ACHE
Rhizoma C	MOL001461	Dihydro cheleryt hrine	Estrogen receptor beta	ESR2
Rhizoma C	MOL001461	Dihydro cheleryt hrine	Mitogen-activated protein kinase 14	MAPK1 4
Rhizoma C	MOL001461	Dihydro cheleryt hrine	Trypsin-1	PRSS1

Rhizoma (	MOL001461	Dihydro cheleryt hrine	Cyclin-A2	CCNA2
Rhizoma (	MOL001461	Dihydro cheleryt hrine	Nuclear receptor coactivator 2	NCOA2
Rhizoma (	MOL001463	Dihydro sanguina rine	Nitric oxide synthase, inducible	NOS2
Rhizoma (	MOL001463	Dihydro sanguina rine	Prostaglandin G/H synthase 1	PTGS1
Rhizoma (	MOL001463	Dihydro sanguina rine	Estrogen receptor	ESR1
Rhizoma (	MOL001463	Dihydro sanguina rine	Androgen receptor	AR
Rhizoma (	MOL001463	Dihydro sanguina rine	Peroxisome proliferator activated receptor gamma	PPARG
Rhizoma (	MOL001463	Dihydro sanguina rine	Prostaglandin G/H synthase 2	PTGS2
Rhizoma (	MOL001463	Dihydro sanguina rine	Acetylcholinesterase	ACHE
Rhizoma (	MOL001463	Dihydro sanguina rine	Estrogen receptor beta	ESR2

Rhizoma C	MOL001463	Dihydro sanguina rine	Mitogen-activated protein kinase 14	MAPK1 4
Rhizoma C	MOL001463	Dihydro sanguina rine	Trypsin-1	PRSS1
Rhizoma C	MOL001463	Dihydro sanguina rine	Cyclin-A2	CCNA2
Rhizoma C	MOL001463	Dihydro sanguina rine	Nuclear receptor coactivator 2	NCOA2
Rhizoma C	MOL001474	sanguina rine	Prostaglandin G/H synthase 1	PTGS1
Rhizoma C	MOL001474	sanguina rine	Prostaglandin G/H synthase 2	PTGS2
Rhizoma C	MOL001474	sanguina rine	Retinoic acid receptor RXR-alpha	RXRA
Rhizoma C	MOL000217	(S)-Scouleri ne	Prostaglandin G/H synthase 1	PTGS1
Rhizoma C	MOL000217	(S)-Scouleri ne	Muscarinic acetylcholine receptor M3	CHRM3
Rhizoma C	MOL000217	(S)-Scouleri ne	Potassium voltage-gated channel subfamily H member 2	KCNH2
Rhizoma C	MOL000217	(S)-Scouleri ne	Muscarinic acetylcholine receptor M1	CHRM1

Rhizoma C	MOL000217	(S)-Scoulerine	D(1B) dopamine receptor	DRD5
Rhizoma C	MOL000217	(S)-Scoulerine	Sodium channel protein type 5 subunit alpha	SCN5A
Rhizoma C	MOL000217	(S)-Scoulerine	Muscarinic acetylcholine receptor M5	CHRM5
Rhizoma C	MOL000217	(S)-Scoulerine	Prostaglandin G/H synthase 2	PTGS2
Rhizoma C	MOL000217	(S)-Scoulerine	Alpha-2A adrenergic receptor	ADRA2A
Rhizoma C	MOL000217	(S)-Scoulerine	Coagulation factor VII	F7
Rhizoma C	MOL000217	(S)-Scoulerine	Alpha-2C adrenergic receptor	ADRA2C
Rhizoma C	MOL000217	(S)-Scoulerine	Muscarinic acetylcholine receptor M4	CHRM4
Rhizoma C	MOL000217	(S)-Scoulerine	Delta-type opioid receptor	OPRD1
Rhizoma C	MOL000217	(S)-Scoulerine	Alpha-1A adrenergic receptor	ADRA1A

Rhizoma C	MOL000217	(S)-Scoulerine	Muscarinic acetylcholine receptor M2	CHRM2
Rhizoma C	MOL000217	(S)-Scoulerine	Alpha-1B adrenergic receptor	ADRA1B
Rhizoma C	MOL000217	(S)-Scoulerine	D(3) dopamine receptor	DRD3
Rhizoma C	MOL000217	(S)-Scoulerine	Sodium-dependent dopamine transporter	SLC6A3
Rhizoma C	MOL000217	(S)-Scoulerine	Beta-2 adrenergic receptor	ADRB2
Rhizoma C	MOL000217	(S)-Scoulerine	Alpha-1D adrenergic receptor	ADRA1D
Rhizoma C	MOL000217	(S)-Scoulerine	Sodium-dependent serotonin transporter	SLC6A4
Rhizoma C	MOL000217	(S)-Scoulerine	Mu-type opioid receptor	OPRM1
Rhizoma C	MOL000217	(S)-Scoulerine	Nuclear receptor coactivator 2	NCOA2
Rhizoma C	MOL000217	(S)-Scoulerine	Nuclear receptor coactivator 1	NCOA1

Rhizoma C	MOL000217	(S)-Scouleri ne	Retinoic acid receptor RXR-alpha	RXRA
Rhizoma C	MOL000217	(S)-Scouleri ne	Sodium-dependent noradrenaline transporter	SLC6A2
Rhizoma C	MOL000217	(S)-Scouleri ne	Alpha-2B adrenergic receptor	ADRA2 B
Rhizoma C	MOL002670	Cavidine	Prostaglandin G/H synthase 1	PTGS1
Rhizoma C	MOL002670	Cavidine	Muscarinic acetylcholine receptor M3	CHRM3
Rhizoma C	MOL002670	Cavidine	Potassium voltage-gated channel subfamily H member 2	KCNH2
Rhizoma C	MOL002670	Cavidine	Muscarinic acetylcholine receptor M1	CHRM1
Rhizoma C	MOL002670	Cavidine	Beta-1 adrenergic receptor	ADRB1
Rhizoma C	MOL002670	Cavidine	Sodium channel protein type 5 subunit alpha	SCN5A
Rhizoma C	MOL002670	Cavidine	Muscarinic acetylcholine receptor M5	CHRM5
Rhizoma C	MOL002670	Cavidine	Prostaglandin G/H synthase 2	PTGS2
Rhizoma C	MOL002670	Cavidine	5-hydroxytryptamine receptor 3A	HTR3A
Rhizoma C	MOL002670	Cavidine	Alpha-2C adrenergic receptor	ADRA2 C

Rhizoma C	MOL002670	Cavidine	Muscarinic acetylcholine receptor M4	CHRM4
Rhizoma C	MOL002670	Cavidine	Retinoic acid receptor RXR-alpha	RXRA
Rhizoma C	MOL002670	Cavidine	Delta-type opioid receptor	OPRD1
Rhizoma C	MOL002670	Cavidine	Alpha-1B adrenergic receptor	ADRA1B
Rhizoma C	MOL002670	Cavidine	Beta-2 adrenergic receptor	ADRB2
Rhizoma C	MOL002670	Cavidine	Alpha-1D adrenergic receptor	ADRA1D
Rhizoma C	MOL002670	Cavidine	Mu-type opioid receptor	OPRM1
Rhizoma C	MOL002670	Cavidine	Retinoic acid receptor RXR-beta	RXRB
Rhizoma C	MOL002670	Cavidine	Sodium-dependent serotonin transporter	SLC6A4
Rhizoma C	MOL002670	Cavidine	Coagulation factor VII	F7
Rhizoma C	MOL002670	Cavidine	cAMP and cAMP-inhibited cGMP 3',5'-cyclic phosphodiesterase 10A	PDE10A
Rhizoma C	MOL002903	(R)-Canadine	Prostaglandin G/H synthase 1	PTGS1
Rhizoma C	MOL002903	(R)-Canadine	Muscarinic acetylcholine receptor M3	CHRM3
Rhizoma C	MOL002903	(R)-Canadine	Potassium voltage-gated channel subfamily H member 2	KCNH2

Rhizoma C	MOL002903	(R)-Canadine	Muscarinic acetylcholine receptor M1	CHRM1
Rhizoma C	MOL002903	(R)-Canadine	Sodium channel protein type 5 subunit alpha	SCN5A
Rhizoma C	MOL002903	(R)-Canadine	Muscarinic acetylcholine receptor M5	CHRM5
Rhizoma C	MOL002903	(R)-Canadine	Prostaglandin G/H synthase 2	PTGS2
Rhizoma C	MOL002903	(R)-Canadine	5-hydroxytryptamine receptor 3A	HTR3A
Rhizoma C	MOL002903	(R)-Canadine	Alpha-2C adrenergic receptor	ADRA2C
Rhizoma C	MOL002903	(R)-Canadine	Muscarinic acetylcholine receptor M4	CHRM4
Rhizoma C	MOL002903	(R)-Canadine	Delta-type opioid receptor	OPRD1
Rhizoma C	MOL002903	(R)-Canadine	Alpha-1B adrenergic receptor	ADRA1B
Rhizoma C	MOL002903	(R)-Canadine	Sodium-dependent dopamine transporter	SLC6A3

Rhizoma C	MOL002903	(R)-Canadin e	Beta-2 adrenergic receptor	ADRB2
Rhizoma C	MOL002903	(R)-Canadin e	Alpha-1D adrenergic receptor	ADRA1 D
Rhizoma C	MOL002903	(R)-Canadin e	Sodium-dependent serotonin transporter	SLC6A4
Rhizoma C	MOL002903	(R)-Canadin e	Mu-type opioid receptor	OPRM1
Rhizoma C	MOL002903	(R)-Canadin e	cAMP and cAMP-inhibited cGMP 3',5'-cyclic phosphodiesterase 10A	PDE10A
Rhizoma C	MOL002903	(R)-Canadin e	D(1B) dopamine receptor	DRD5
Rhizoma C	MOL002903	(R)-Canadin e	Retinoic acid receptor RXR-alpha	RXRA
Rhizoma C	MOL002903	(R)-Canadin e	Sodium-dependent noradrenaline transporter	SLC6A2
Rhizoma C	MOL002903	(R)-Canadin e	Alpha-1A adrenergic receptor	ADRA1 A
Rhizoma C	MOL002903	(R)-Canadin e	Muscarinic acetylcholine receptor M2	CHRM2

Rhizoma C	MOL000359	sitosterol	Progesterone receptor	PGR
Rhizoma C	MOL000359	sitosterol	Nuclear receptor coactivator 2	NCOA2
Rhizoma C	MOL000359	sitosterol	Mineralocorticoid receptor	NR3C2
Rhizoma C	MOL004071	Hyndarin	Muscarinic acetylcholine receptor M3	CHRM3
Rhizoma C	MOL004071	Hyndarin	Potassium voltage-gated channel subfamily H member 2	KCNH2
Rhizoma C	MOL004071	Hyndarin	Muscarinic acetylcholine receptor M1	CHRM1
Rhizoma C	MOL004071	Hyndarin	Sodium channel protein type 5 subunit alpha	SCN5A
Rhizoma C	MOL004071	Hyndarin	Muscarinic acetylcholine receptor M5	CHRM5
Rhizoma C	MOL004071	Hyndarin	Prostaglandin G/H synthase 2	PTGS2
Rhizoma C	MOL004071	Hyndarin	5-hydroxytryptamine receptor 3A	HTR3A
Rhizoma C	MOL004071	Hyndarin	Alpha-2C adrenergic receptor	ADRA2C
Rhizoma C	MOL004071	Hyndarin	Muscarinic acetylcholine receptor M4	CHRM4
Rhizoma C	MOL004071	Hyndarin	Delta-type opioid receptor	OPRD1
Rhizoma C	MOL004071	Hyndarin	Sodium-dependent noradrenaline transporter	SLC6A2

Rhizoma C	MOL004071	Hyndari n	Muscarinic acetylcholine receptor M2	CHRM2
Rhizoma C	MOL004071	Hyndari n	Alpha-1B adrenergic receptor	ADRA1 B
Rhizoma C	MOL004071	Hyndari n	Sodium-dependent dopamine transporter	SLC6A3
Rhizoma C	MOL004071	Hyndari n	Beta-2 adrenergic receptor	ADRB2
Rhizoma C	MOL004071	Hyndari n	Alpha-1D adrenergic receptor	ADRA1 D
Rhizoma C	MOL004071	Hyndari n	Sodium-dependent serotonin transporter	SLC6A4
Rhizoma C	MOL004071	Hyndari n	Mu-type opioid receptor	OPRM1
Rhizoma C	MOL004071	Hyndari n	Retinoic acid receptor RXR-beta	RXRB
Rhizoma C	MOL004071	Hyndari n	Prostaglandin G/H synthase 1	PTGS1
Rhizoma C	MOL004071	Hyndari n	Beta-1 adrenergic receptor	ADRB1
Rhizoma C	MOL004071	Hyndari n	Retinoic acid receptor RXR-alpha	RXRA
Rhizoma C	MOL004071	Hyndari n	D(3) dopamine receptor	DRD3
Rhizoma C	MOL004071	Hyndari n	D(2) dopamine receptor	DRD2
Rhizoma C	MOL004071	Hyndari n	Nuclear receptor coactivator 1	NCOA1

Rhizoma C	MOL004190	(-)-alpha-N-methylcanadine	Muscarinic acetylcholine receptor M3	CHRM3
Rhizoma C	MOL004190	(-)-alpha-N-methylcanadine	Potassium voltage-gated channel subfamily H member 2	KCNH2
Rhizoma C	MOL004190	(-)-alpha-N-methylcanadine	Muscarinic acetylcholine receptor M1	CHRM1
Rhizoma C	MOL004190	(-)-alpha-N-methylcanadine	Sodium channel protein type 5 subunit alpha	SCN5A
Rhizoma C	MOL004190	(-)-alpha-N-methylcanadine	Muscarinic acetylcholine receptor M5	CHRM5
Rhizoma C	MOL004190	(-)-alpha-N-methylcanadine	Prostaglandin G/H synthase 2	PTGS2
Rhizoma C	MOL004190	(-)-alpha-N-methylcanadine	Muscarinic acetylcholine receptor M4	CHRM4

Rhizoma C	MOL004190	(-)-alpha-N-methylcanadine	Alpha-1B adrenergic receptor	ADRA1B
Rhizoma C	MOL004190	(-)-alpha-N-methylcanadine	Beta-2 adrenergic receptor	ADRB2
Rhizoma C	MOL004190	(-)-alpha-N-methylcanadine	Alpha-1D adrenergic receptor	ADRA1D
Rhizoma C	MOL004190	(-)-alpha-N-methylcanadine	Nuclear receptor coactivator 2	NCOA2
Rhizoma C	MOL004190	(-)-alpha-N-methylcanadine	Nuclear receptor coactivator 1	NCOA1
Rhizoma C	MOL004191	Capaurine	Prostaglandin G/H synthase 1	PTGS1
Rhizoma C	MOL004191	Capaurine	Muscarinic acetylcholine receptor M3	CHRM3
Rhizoma C	MOL004191	Capaurine	Potassium voltage-gated channel subfamily H member 2	KCNH2
Rhizoma C	MOL004191	Capaurine	Muscarinic acetylcholine receptor M1	CHRM1

Rhizoma C	MOL004191	Capaurine	Sodium channel protein type 5 subunit alpha	SCN5A
Rhizoma C	MOL004191	Capaurine	Muscarinic acetylcholine receptor M5	CHRM5
Rhizoma C	MOL004191	Capaurine	Prostaglandin G/H synthase 2	PTGS2
Rhizoma C	MOL004191	Capaurine	Vascular endothelial growth factor receptor 2	KDR
Rhizoma C	MOL004191	Capaurine	Muscarinic acetylcholine receptor M4	CHRM4
Rhizoma C	MOL004191	Capaurine	Retinoic acid receptor RXR-alpha	RXRA
Rhizoma C	MOL004191	Capaurine	Delta-type opioid receptor	OPRD1
Rhizoma C	MOL004191	Capaurine	Alpha-1B adrenergic receptor	ADRA1B
Rhizoma C	MOL004191	Capaurine	Sodium-dependent dopamine transporter	SLC6A3
Rhizoma C	MOL004191	Capaurine	Beta-2 adrenergic receptor	ADRB2
Rhizoma C	MOL004191	Capaurine	Alpha-1D adrenergic receptor	ADRA1D
Rhizoma C	MOL004191	Capaurine	Sodium-dependent serotonin transporter	SLC6A4
Rhizoma C	MOL004191	Capaurine	Mu-type opioid receptor	OPRM1
Rhizoma C	MOL004191	Capaurine	Retinoic acid receptor RXR-beta	RXRB
Rhizoma C	MOL004193	Clarkeanidine	Prostaglandin G/H synthase 1	PTGS1

Rhizoma C	MOL004193	Clarkeanidine	Muscarinic acetylcholine receptor M3	CHRM3
Rhizoma C	MOL004193	Clarkeanidine	Potassium voltage-gated channel subfamily H member 2	KCNH2
Rhizoma C	MOL004193	Clarkeanidine	Muscarinic acetylcholine receptor M1	CHRM1
Rhizoma C	MOL004193	Clarkeanidine	Sodium channel protein type 5 subunit alpha	SCN5A
Rhizoma C	MOL004193	Clarkeanidine	Muscarinic acetylcholine receptor M5	CHRM5
Rhizoma C	MOL004193	Clarkeanidine	Prostaglandin G/H synthase 2	PTGS2
Rhizoma C	MOL004193	Clarkeanidine	Alpha-2C adrenergic receptor	ADRA2C
Rhizoma C	MOL004193	Clarkeanidine	Muscarinic acetylcholine receptor M4	CHRM4
Rhizoma C	MOL004193	Clarkeanidine	Retinoic acid receptor RXR-alpha	RXRA
Rhizoma C	MOL004193	Clarkeanidine	Delta-type opioid receptor	OPRD1
Rhizoma C	MOL004193	Clarkeanidine	Sodium-dependent noradrenaline transporter	SLC6A2
Rhizoma C	MOL004193	Clarkeanidine	Alpha-1A adrenergic receptor	ADRA1A
Rhizoma C	MOL004193	Clarkeanidine	Muscarinic acetylcholine receptor M2	CHRM2
Rhizoma C	MOL004193	Clarkeanidine	Alpha-2B adrenergic receptor	ADRA2B

Rhizoma C	MOL004193	Clarkeanidine	Alpha-1B adrenergic receptor	ADRA1B
Rhizoma C	MOL004193	Clarkeanidine	Sodium-dependent dopamine transporter	SLC6A3
Rhizoma C	MOL004193	Clarkeanidine	Beta-2 adrenergic receptor	ADRB2
Rhizoma C	MOL004193	Clarkeanidine	Alpha-1D adrenergic receptor	ADRA1D
Rhizoma C	MOL004193	Clarkeanidine	Sodium-dependent serotonin transporter	SLC6A4
Rhizoma C	MOL004193	Clarkeanidine	Mu-type opioid receptor	OPRM1
Rhizoma C	MOL004195	CORYDALINE	Muscarinic acetylcholine receptor M3	CHRM3
Rhizoma C	MOL004195	CORYDALINE	Potassium voltage-gated channel subfamily H member 2	KCNH2
Rhizoma C	MOL004195	CORYDALINE	Muscarinic acetylcholine receptor M1	CHRM1
Rhizoma C	MOL004195	CORYDALINE	Sodium channel protein type 5 subunit alpha	SCN5A
Rhizoma C	MOL004195	CORYDALINE	Muscarinic acetylcholine receptor M5	CHRM5
Rhizoma C	MOL004195	CORYDALINE	Prostaglandin G/H synthase 2	PTGS2

Rhizoma C	MOL004195	CORY DALIN E	Muscarinic acetylcholine receptor M4	CHRM4
Rhizoma C	MOL004195	CORY DALIN E	Retinoic acid receptor RXR-alpha	RXRA
Rhizoma C	MOL004195	CORY DALIN E	Delta-type opioid receptor	OPRD1
Rhizoma C	MOL004195	CORY DALIN E	Alpha-2B adrenergic receptor	ADRA2 B
Rhizoma C	MOL004195	CORY DALIN E	Alpha-1B adrenergic receptor	ADRA1 B
Rhizoma C	MOL004195	CORY DALIN E	Sodium-dependent dopamine transporter	SLC6A3
Rhizoma C	MOL004195	CORY DALIN E	Beta-2 adrenergic receptor	ADRB2
Rhizoma C	MOL004195	CORY DALIN E	Alpha-1D adrenergic receptor	ADRA1 D
Rhizoma C	MOL004195	CORY DALIN E	Sodium-dependent serotonin transporter	SLC6A4
Rhizoma C	MOL004195	CORY DALIN E	D(2) dopamine receptor	DRD2

Rhizoma C	MOL004195	CORY DALIN E	Mu-type opioid receptor	OPRM1
Rhizoma C	MOL004195	CORY DALIN E	Retinoic acid receptor RXR-beta	RXRB
Rhizoma C	MOL004196	Corydal mine	Prostaglandin G/H synthase 1	PTGS1
Rhizoma C	MOL004196	Corydal mine	Muscarinic acetylcholine receptor M3	CHRM3
Rhizoma C	MOL004196	Corydal mine	Potassium voltage-gated channel subfamily H member 2	KCNH2
Rhizoma C	MOL004196	Corydal mine	Muscarinic acetylcholine receptor M1	CHRM1
Rhizoma C	MOL004196	Corydal mine	Estrogen receptor	ESR1
Rhizoma C	MOL004196	Corydal mine	Sodium channel protein type 5 subunit alpha	SCN5A
Rhizoma C	MOL004196	Corydal mine	Peroxisome proliferator activated receptor gamma	PPARG
Rhizoma C	MOL004196	Corydal mine	Muscarinic acetylcholine receptor M5	CHRM5
Rhizoma C	MOL004196	Corydal mine	Prostaglandin G/H synthase 2	PTGS2
Rhizoma C	MOL004196	Corydal mine	Alpha-2C adrenergic receptor	ADRA2 C
Rhizoma C	MOL004196	Corydal mine	Muscarinic acetylcholine receptor M4	CHRM4

Rhizoma C	MOL004196	Corydal mine	Retinoic acid receptor RXR-alpha	RXRA
Rhizoma C	MOL004196	Corydal mine	Delta-type opioid receptor	OPRD1
Rhizoma C	MOL004196	Corydal mine	Acetylcholinesterase	ACHE
Rhizoma C	MOL004196	Corydal mine	Alpha-1B adrenergic receptor	ADRA1 B
Rhizoma C	MOL004196	Corydal mine	Beta-2 adrenergic receptor	ADRB2
Rhizoma C	MOL004196	Corydal mine	Alpha-1D adrenergic receptor	ADRA1 D
Rhizoma C	MOL004196	Corydal mine	Mu-type opioid receptor	OPRM1
Rhizoma C	MOL004196	Corydal mine	Retinoic acid receptor RXR-beta	RXRB
Rhizoma C	MOL004196	Corydal mine	Nuclear receptor coactivator 2	NCOA2
Rhizoma C	MOL004196	Corydal mine	Nuclear receptor coactivator 1	NCOA1
Rhizoma C	MOL004197	Corydin e	Prostaglandin G/H synthase 1	PTGS1
Rhizoma C	MOL004197	Corydin e	Muscarinic acetylcholine receptor M3	CHRM3
Rhizoma C	MOL004197	Corydin e	Potassium voltage-gated channel subfamily H member 2	KCNH2
Rhizoma C	MOL004197	Corydin e	Muscarinic acetylcholine receptor M1	CHRM1

Rhizoma C	MOL004197	Corydin e	Androgen receptor	AR
Rhizoma C	MOL004197	Corydin e	Sodium channel protein type 5 subunit alpha	SCN5A
Rhizoma C	MOL004197	Corydin e	Muscarinic acetylcholine receptor M5	CHRM5
Rhizoma C	MOL004197	Corydin e	Prostaglandin G/H synthase 2	PTGS2
Rhizoma C	MOL004197	Corydin e	Retinoic acid receptor RXR-alpha	RXRA
Rhizoma C	MOL004197	Corydin e	Delta-type opioid receptor	OPRD1
Rhizoma C	MOL004197	Corydin e	Acetylcholinesterase	ACHE
Rhizoma C	MOL004197	Corydin e	Alpha-1B adrenergic receptor	ADRA1 B
Rhizoma C	MOL004197	Corydin e	Beta-2 adrenergic receptor	ADRB2
Rhizoma C	MOL004197	Corydin e	Alpha-1D adrenergic receptor	ADRA1 D
Rhizoma C	MOL004197	Corydin e	Sodium-dependent serotonin transporter	SLC6A4
Rhizoma C	MOL004197	Corydin e	Mu-type opioid receptor	OPRM1
Rhizoma C	MOL004198	18797-79-0	Prostaglandin G/H synthase 1	PTGS1
Rhizoma C	MOL004198	18797-79-0	Muscarinic acetylcholine receptor M3	CHRM3
Rhizoma C	MOL004198	18797-79-0	Muscarinic acetylcholine receptor M1	CHRM1

Rhizoma C	MOL004198	18797-79-0	Sodium channel protein type 5 subunit alpha	SCN5A
Rhizoma C	MOL004198	18797-79-0	Muscarinic acetylcholine receptor M5	CHRM5
Rhizoma C	MOL004198	18797-79-0	Prostaglandin G/H synthase 2	PTGS2
Rhizoma C	MOL004198	18797-79-0	Delta-type opioid receptor	OPRD1
Rhizoma C	MOL004198	18797-79-0	Alpha-1B adrenergic receptor	ADRA1B
Rhizoma C	MOL004198	18797-79-0	Mu-type opioid receptor	OPRM1
Rhizoma C	MOL004199	Corynoloxine	Prostaglandin G/H synthase 1	PTGS1
Rhizoma C	MOL004199	Corynoloxine	Androgen receptor	AR
Rhizoma C	MOL004199	Corynoloxine	Sodium channel protein type 5 subunit alpha	SCN5A
Rhizoma C	MOL004199	Corynoloxine	Prostaglandin G/H synthase 2	PTGS2
Rhizoma C	MOL004199	Corynoloxine	Delta-type opioid receptor	OPRD1
Rhizoma C	MOL004199	Corynoloxine	Acetylcholinesterase	ACHE
Rhizoma C	MOL004199	Corynoloxine	Mu-type opioid receptor	OPRM1

Rhizoma C	MOL004200	methyl-[2-(3,4,6,7-tetramethoxy-1-phenanthryl)ethyl]amine	Prostaglandin G/H synthase 1	PTGS1
Rhizoma C	MOL004200	methyl-[2-(3,4,6,7-tetramethoxy-1-phenanthryl)ethyl]amine	Potassium voltage-gated channel subfamily H member 2	KCNH2
Rhizoma C	MOL004200	methyl-[2-(3,4,6,7-tetramethoxy-1-phenanthryl)ethyl]amine	Sodium channel protein type 5 subunit alpha	SCN5A

Rhizoma C	MOL004200	methyl-[2-(3,4,6,7-tetramethoxy-1-phenanthryl)ethyl]amine	Prostaglandin G/H synthase 2	PTGS2
Rhizoma C	MOL004200	methyl-[2-(3,4,6,7-tetramethoxy-1-phenanthryl)ethyl]amine	Retinoic acid receptor RXR-alpha	RXRA
Rhizoma C	MOL004200	methyl-[2-(3,4,6,7-tetramethoxy-1-phenanthryl)ethyl]amine	Nuclear receptor coactivator 2	NCOA2
Rhizoma C	MOL004202	dehydrocavidine	Prostaglandin G/H synthase 1	PTGS1
Rhizoma C	MOL004202	dehydrocavidine	Muscarinic acetylcholine receptor M3	CHRM3

Rhizoma C	MOL004202	dehydro cavidine	Potassium voltage-gated channel subfamily H member 2	KCNH2
Rhizoma C	MOL004202	dehydro cavidine	Muscarinic acetylcholine receptor M1	CHRM1
Rhizoma C	MOL004202	dehydro cavidine	Sodium channel protein type 5 subunit alpha	SCN5A
Rhizoma C	MOL004202	dehydro cavidine	Muscarinic acetylcholine receptor M5	CHRM5
Rhizoma C	MOL004202	dehydro cavidine	Prostaglandin G/H synthase 2	PTGS2
Rhizoma C	MOL004202	dehydro cavidine	Coagulation factor VII	F7
Rhizoma C	MOL004202	dehydro cavidine	Muscarinic acetylcholine receptor M4	CHRM4
Rhizoma C	MOL004202	dehydro cavidine	Retinoic acid receptor RXR-alpha	RXRA
Rhizoma C	MOL004202	dehydro cavidine	Delta-type opioid receptor	OPRD1
Rhizoma C	MOL004202	dehydro cavidine	Muscarinic acetylcholine receptor M2	CHRM2
Rhizoma C	MOL004202	dehydro cavidine	Alpha-1B adrenergic receptor	ADRA1B
Rhizoma C	MOL004202	dehydro cavidine	Beta-2 adrenergic receptor	ADRB2
Rhizoma C	MOL004202	dehydro cavidine	Alpha-1D adrenergic receptor	ADRA1D
Rhizoma C	MOL004202	dehydro cavidine	Mu-type opioid receptor	OPRM1

Rhizoma C	MOL004203	Dehydro corybulb ine	Nitric oxide synthase, inducible	NOS2
Rhizoma C	MOL004203	Dehydro corybulb ine	Prostaglandin G/H synthase 1	PTGS1
Rhizoma C	MOL004203	Dehydro corybulb ine	Potassium voltage-gated channel subfamily H member 2	KCNH2
Rhizoma C	MOL004203	Dehydro corybulb ine	Estrogen receptor	ESR1
Rhizoma C	MOL004203	Dehydro corybulb ine	Androgen receptor	AR
Rhizoma C	MOL004203	Dehydro corybulb ine	Sodium channel protein type 5 subunit alpha	SCN5A
Rhizoma C	MOL004203	Dehydro corybulb ine	Prostaglandin G/H synthase 2	PTGS2
Rhizoma C	MOL004203	Dehydro corybulb ine	Retinoic acid receptor RXR-alpha	RXRA
Rhizoma C	MOL004203	Dehydro corybulb ine	Mitogen-activated protein kinase 14	MAPK1 4
Rhizoma C	MOL004203	Dehydro corybulb ine	Serine/threonine-protein kinase Chk1	CHEK1

Rhizoma C	MOL004203	Dehydrocorybulbine	Trypsin-1	PRSS1
Rhizoma C	MOL004203	Dehydrocorybulbine	Nuclear receptor coactivator 2	NCOA2
Rhizoma C	MOL004204	dehydrocorydaline	Nitric oxide synthase, inducible	NOS2
Rhizoma C	MOL004204	dehydrocorydaline	Prostaglandin G/H synthase 1	PTGS1
Rhizoma C	MOL004204	dehydrocorydaline	Potassium voltage-gated channel subfamily H member 2	KCNH2
Rhizoma C	MOL004204	dehydrocorydaline	Estrogen receptor	ESR1
Rhizoma C	MOL004204	dehydrocorydaline	Androgen receptor	AR
Rhizoma C	MOL004204	dehydrocorydaline	Sodium channel protein type 5 subunit alpha	SCN5A
Rhizoma C	MOL004204	dehydrocorydaline	Prostaglandin G/H synthase 2	PTGS2
Rhizoma C	MOL004204	dehydrocorydaline	Retinoic acid receptor RXR-alpha	RXRA

Rhizoma C	MOL004204	dehydro corydali ne	Mitogen-activated protein kinase 14	MAPK1 4
Rhizoma C	MOL004204	dehydro corydali ne	Serine/threonine-protein kinase Chk1	CHEK1
Rhizoma C	MOL004204	dehydro corydali ne	Retinoic acid receptor RXR-beta	RXRB
Rhizoma C	MOL004204	dehydro corydali ne	Trypsin-1	PRSS1
Rhizoma C	MOL004204	dehydro corydali ne	Nuclear receptor coactivator 2	NCOA2
Rhizoma C	MOL004204	dehydro corydali ne	cAMP and cAMP-inhibited cGMP 3',5'-cyclic phosphodiesterase 10A	PDE10A
Rhizoma C	MOL004205	Dehydro corydal mine	Nitric oxide synthase, inducible	NOS2
Rhizoma C	MOL004205	Dehydro corydal mine	Prostaglandin G/H synthase 1	PTGS1
Rhizoma C	MOL004205	Dehydro corydal mine	Potassium voltage-gated channel subfamily H member 2	KCNH2
Rhizoma C	MOL004205	Dehydro corydal mine	Estrogen receptor	ESR1

Rhizoma C	MOL004205	Dehydro corydal mine	Androgen receptor	AR
Rhizoma C	MOL004205	Dehydro corydal mine	Sodium channel protein type 5 subunit alpha	SCN5A
Rhizoma C	MOL004205	Dehydro corydal mine	Prostaglandin G/H synthase 2	PTGS2
Rhizoma C	MOL004205	Dehydro corydal mine	Coagulation factor VII	F7
Rhizoma C	MOL004205	Dehydro corydal mine	Retinoic acid receptor RXR-alpha	RXRA
Rhizoma C	MOL004205	Dehydro corydal mine	Beta-2 adrenergic receptor	ADRB2
Rhizoma C	MOL004205	Dehydro corydal mine	Estrogen receptor beta	ESR2
Rhizoma C	MOL004205	Dehydro corydal mine	Trypsin-1	PRSS1
Rhizoma C	MOL004205	Dehydro corydal mine	Cyclin-A2	CCNA2
Rhizoma C	MOL004205	Dehydro corydal mine	Nuclear receptor coactivator 2	NCOA2

Rhizoma C	MOL004208	demethylcorydalmatine	Prostaglandin G/H synthase 1	PTGS1
Rhizoma C	MOL004208	demethylcorydalmatine	Muscarinic acetylcholine receptor M3	CHRM3
Rhizoma C	MOL004208	demethylcorydalmatine	Potassium voltage-gated channel subfamily H member 2	KCNH2
Rhizoma C	MOL004208	demethylcorydalmatine	Muscarinic acetylcholine receptor M1	CHRM1
Rhizoma C	MOL004208	demethylcorydalmatine	Sodium channel protein type 5 subunit alpha	SCN5A
Rhizoma C	MOL004208	demethylcorydalmatine	Muscarinic acetylcholine receptor M5	CHRM5
Rhizoma C	MOL004208	demethylcorydalmatine	Prostaglandin G/H synthase 2	PTGS2
Rhizoma C	MOL004208	demethylcorydalmatine	Alpha-2C adrenergic receptor	ADRA2C
Rhizoma C	MOL004208	demethylcorydalmatine	Muscarinic acetylcholine receptor M4	CHRM4
Rhizoma C	MOL004208	demethylcorydalmatine	Delta-type opioid receptor	OPRD1

Rhizoma C	MOL004208	demethylcorydalmatine	Alpha-1B adrenergic receptor	ADRA1B
Rhizoma C	MOL004208	demethylcorydalmatine	Sodium-dependent dopamine transporter	SLC6A3
Rhizoma C	MOL004208	demethylcorydalmatine	Beta-2 adrenergic receptor	ADRB2
Rhizoma C	MOL004208	demethylcorydalmatine	Alpha-1D adrenergic receptor	ADRA1D
Rhizoma C	MOL004208	demethylcorydalmatine	Sodium-dependent serotonin transporter	SLC6A4
Rhizoma C	MOL004208	demethylcorydalmatine	Mu-type opioid receptor	OPRM1
Rhizoma C	MOL004209	13-methyldehydrocorydalmine	Nitric oxide synthase, inducible	NOS2
Rhizoma C	MOL004209	13-methyldehydrocorydalmine	Prostaglandin G/H synthase 1	PTGS1

Rhizoma (	MOL004209	13-methyldehydrocorydalmine	Potassium voltage-gated channel subfamily H member 2	KCNH2
Rhizoma (	MOL004209	13-methyldehydrocorydalmine	Estrogen receptor	ESR1
Rhizoma (	MOL004209	13-methyldehydrocorydalmine	Androgen receptor	AR
Rhizoma (	MOL004209	13-methyldehydrocorydalmine	Prostaglandin G/H synthase 2	PTGS2
Rhizoma (	MOL004209	13-methyldehydrocorydalmine	Retinoic acid receptor RXR-alpha	RXRA
Rhizoma (	MOL004209	13-methyldehydrocorydalmine	Mitogen-activated protein kinase 14	MAPK14

Rhizoma C	MOL004209	13-methyldehydrocorydalmine	Serine/threonine-protein kinase Chk1	CHEK1
Rhizoma C	MOL004209	13-methyldehydrocorydalmine	Trypsin-1	PRSS1
Rhizoma C	MOL004209	13-methyldehydrocorydalmine	Nuclear receptor coactivator 2	NCOA2

Rhizoma C	MOL004210	(1S,8'R)-6,7-dimethoxy-2-methylspiro[3,4-dihydroisoquinoline-1,7'-6,8-dihydrocyclopenta[g][1,3]benzodioxole]-8'-ol	Prostaglandin G/H synthase 1	PTGS1

Rhizoma C	MOL004210	(1S,8'R)-6,7-dimethoxy-2-methylspiro[3,4-dihydroisoquinoline-1,7'-6,8-dihydrocyclopenta[g][1,3]benzodioxole]-8'-ol	Muscarinic acetylcholine receptor M3	CHRM3

Rhizoma C	MOL004210	(1S,8'R)-6,7-dimethoxy-2-methylspiro[3,4-dihydroisoquinoline-1,7'-6,8-dihydrocyclopenta[g][1,3]benzodioxole]-8'-ol	Sodium channel protein type 5 subunit alpha	SCN5A

Rhizoma C	MOL004210	(1S,8'R)-6,7-dimethoxy-2-methylspiro[3,4-dihydroisoquinoline-1,7'-6,8-dihydrocyclopenta[g][1,3]benzodioxole]-8'-ol	Muscarinic acetylcholine receptor M5	CHRM5

Rhizoma C	MOL004210	(1S,8'R)-6,7-dimethoxy-2-methylspiro[3,4-dihydroisoquinoline-1,7'-6,8-dihydrocyclopenta[g][1,3]benzodioxole]-8'-ol	Prostaglandin G/H synthase 2	PTGS2

Rhizoma C	MOL004210	(1S,8'R)-6,7-dimethoxy-2-methylspiro[3,4-dihydroisoquinoline-1,7'-6,8-dihydrocyclopenta[g][1,3]benzodioxole]-8'-ol	Delta-type opioid receptor	OPRD1

Rhizoma C	MOL004210	(1S,8'R)-6,7-dimethoxy-2-methylspiro[3,4-dihydroisoquinoline-1,7'-6,8-dihydrocyclopenta[g][1,3]benzodioxole]-8'-ol	Alpha-1B adrenergic receptor	ADRA1B

Rhizoma C	MOL004210	(1S,8'R)-6,7-dimethoxy-2-methylspiro[3,4-dihydroisoquinoline-1,7'-6,8-dihydrocyclopenta[g][1,3]benzodioxole]-8'-ol	Alpha-1D adrenergic receptor	ADRA1D

Rhizoma C	MOL004210	(1S,8'R)-6,7-dimethoxy-2-methylspiro[3,4-dihydroisoquinoline-1,7'-6,8-dihydrocyclopenta[g][1,3]benzodioxole]-8'-ol	Mu-type opioid receptor	OPRM1

Rhizoma C	MOL004210	(1S,8'R)-6,7-dimethoxy-2-methylspiro[3,4-dihydroisoquinoline-1,7'-6,8-dihydrocyclopenta[g][1,3]benzodioxole]-8'-ol	Nuclear receptor coactivator 1	NCOA1
Rhizoma C	MOL004763	Izoteolin	Prostaglandin G/H synthase 1	PTGS1
Rhizoma C	MOL004763	Izoteolin	Muscarinic acetylcholine receptor M3	CHRM3
Rhizoma C	MOL004763	Izoteolin	Muscarinic acetylcholine receptor M1	CHRM1
Rhizoma C	MOL004763	Izoteolin	Androgen receptor	AR
Rhizoma C	MOL004763	Izoteolin	Sodium channel protein type 5 subunit alpha	SCN5A
Rhizoma C	MOL004763	Izoteolin	Muscarinic acetylcholine receptor M5	CHRM5
Rhizoma C	MOL004763	Izoteolin	Prostaglandin G/H synthase 2	PTGS2

Rhizoma C	MOL004763	Izoteolin	Muscarinic acetylcholine receptor M4	CHRM4
Rhizoma C	MOL004763	Izoteolin	Retinoic acid receptor RXR-alpha	RXRA
Rhizoma C	MOL004763	Izoteolin	Delta-type opioid receptor	OPRD1
Rhizoma C	MOL004763	Izoteolin	Acetylcholinesterase	ACHE
Rhizoma C	MOL004763	Izoteolin	Alpha-1B adrenergic receptor	ADRA1 B
Rhizoma C	MOL004763	Izoteolin	Sodium-dependent dopamine transporter	SLC6A3
Rhizoma C	MOL004763	Izoteolin	Beta-2 adrenergic receptor	ADRB2
Rhizoma C	MOL004763	Izoteolin	Alpha-1D adrenergic receptor	ADRA1 D
Rhizoma C	MOL004763	Izoteolin	Sodium-dependent serotonin transporter	SLC6A4
Rhizoma C	MOL004763	Izoteolin	Mu-type opioid receptor	OPRM1
Rhizoma C	MOL004763	Izoteolin	Alpha-1A adrenergic receptor	ADRA1 A
Rhizoma C	MOL004214	isocoryb ulbine	Prostaglandin G/H synthase 1	PTGS1
Rhizoma C	MOL004214	isocoryb ulbine	Muscarinic acetylcholine receptor M3	CHRM3
Rhizoma C	MOL004214	isocoryb ulbine	Potassium voltage-gated channel subfamily H member 2	KCNH2
Rhizoma C	MOL004214	isocoryb ulbine	Estrogen receptor	ESR1
Rhizoma C	MOL004214	isocoryb ulbine	Sodium channel protein type 5 subunit alpha	SCN5A

Rhizoma C	MOL004214	isocorybulbine	Prostaglandin G/H synthase 2	PTGS2
Rhizoma C	MOL004214	isocorybulbine	Retinoic acid receptor RXR-alpha	RXRA
Rhizoma C	MOL004214	isocorybulbine	Delta-type opioid receptor	OPRD1
Rhizoma C	MOL004214	isocorybulbine	Acetylcholinesterase	ACHE
Rhizoma C	MOL004214	isocorybulbine	Alpha-1B adrenergic receptor	ADRA1B
Rhizoma C	MOL004214	isocorybulbine	Beta-2 adrenergic receptor	ADRB2
Rhizoma C	MOL004214	isocorybulbine	Alpha-1D adrenergic receptor	ADRA1D
Rhizoma C	MOL004214	isocorybulbine	Mu-type opioid receptor	OPRM1
Rhizoma C	MOL004214	isocorybulbine	Retinoic acid receptor RXR-beta	RXRB
Rhizoma C	MOL004214	isocorybulbine	Nuclear receptor coactivator 2	NCOA2
Rhizoma C	MOL004214	isocorybulbine	Nuclear receptor coactivator 1	NCOA1
Rhizoma C	MOL004215	leonticine	Prostaglandin G/H synthase 1	PTGS1
Rhizoma C	MOL004215	leonticine	Muscarinic acetylcholine receptor M3	CHRM3
Rhizoma C	MOL004215	leonticine	Potassium voltage-gated channel subfamily H member 2	KCNH2

Rhizoma C	MOL004215	leonticin e	Muscarinic acetylcholine receptor M1	CHRM1
Rhizoma C	MOL004215	leonticin e	D(1B) dopamine receptor	DRD5
Rhizoma C	MOL004215	leonticin e	Beta-1 adrenergic receptor	ADRB1
Rhizoma C	MOL004215	leonticin e	Sodium channel protein type 5 subunit alpha	SCN5A
Rhizoma C	MOL004215	leonticin e	Muscarinic acetylcholine receptor M5	CHRM5
Rhizoma C	MOL004215	leonticin e	Prostaglandin G/H synthase 2	PTGS2
Rhizoma C	MOL004215	leonticin e	Alpha-2A adrenergic receptor	ADRA2 A
Rhizoma C	MOL004215	leonticin e	Alpha-2C adrenergic receptor	ADRA2 C
Rhizoma C	MOL004215	leonticin e	Muscarinic acetylcholine receptor M4	CHRM4
Rhizoma C	MOL004215	leonticin e	Retinoic acid receptor RXR-alpha	RXRA
Rhizoma C	MOL004215	leonticin e	Delta-type opioid receptor	OPRD1
Rhizoma C	MOL004215	leonticin e	Sodium-dependent noradrenaline transporter	SLC6A2
Rhizoma C	MOL004215	leonticin e	Alpha-1A adrenergic receptor	ADRA1 A
Rhizoma C	MOL004215	leonticin e	Muscarinic acetylcholine receptor M2	CHRM2
Rhizoma C	MOL004215	leonticin e	Alpha-2B adrenergic receptor	ADRA2 B

Rhizoma C	MOL004215	leonticine	Alpha-1B adrenergic receptor	ADRA1B
Rhizoma C	MOL004215	leonticine	Sodium-dependent dopamine transporter	SLC6A3
Rhizoma C	MOL004215	leonticine	Beta-2 adrenergic receptor	ADRB2
Rhizoma C	MOL004215	leonticine	Alpha-1D adrenergic receptor	ADRA1D
Rhizoma C	MOL004215	leonticine	Sodium-dependent serotonin transporter	SLC6A4
Rhizoma C	MOL004215	leonticine	D(2) dopamine receptor	DRD2
Rhizoma C	MOL004215	leonticine	Mu-type opioid receptor	OPRM1
Rhizoma C	MOL004215	leonticine	Gamma-aminobutyric acid receptor subunit alpha-1	GABRA1
Rhizoma C	MOL004216	13-methylpalmatrubine	Nitric oxide synthase, inducible	NOS2
Rhizoma C	MOL004216	13-methylpalmatrubine	Prostaglandin G/H synthase 1	PTGS1
Rhizoma C	MOL004216	13-methylpalmatrubine	Potassium voltage-gated channel subfamily H member 2	KCNH2

Rhizoma C	MOL004216	13-methylpalmatrubine	Estrogen receptor	ESR1
Rhizoma C	MOL004216	13-methylpalmatrubine	Androgen receptor	AR
Rhizoma C	MOL004216	13-methylpalmatrubine	Sodium channel protein type 5 subunit alpha	SCN5A
Rhizoma C	MOL004216	13-methylpalmatrubine	Prostaglandin G/H synthase 2	PTGS2
Rhizoma C	MOL004216	13-methylpalmatrubine	Retinoic acid receptor RXR-alpha	RXRA
Rhizoma C	MOL004216	13-methylpalmatrubine	Mitogen-activated protein kinase 14	MAPK14
Rhizoma C	MOL004216	13-methylpalmatrubine	Serine/threonine-protein kinase Chk1	CHEK1

Rhizoma C	MOL004216	13-methylpalmatrubine	Trypsin-1	PRSS1
Rhizoma C	MOL004216	13-methylpalmatrubine	Nuclear receptor coactivator 2	NCOA2
Rhizoma C	MOL004220	N-methyllaurotetanine	Prostaglandin G/H synthase 1	PTGS1
Rhizoma C	MOL004220	N-methyllaurotetanine	Muscarinic acetylcholine receptor M3	CHRM3
Rhizoma C	MOL004220	N-methyllaurotetanine	Muscarinic acetylcholine receptor M1	CHRM1
Rhizoma C	MOL004220	N-methyllaurotetanine	Androgen receptor	AR
Rhizoma C	MOL004220	N-methyllaurotetanine	Sodium channel protein type 5 subunit alpha	SCN5A

Rhizoma C	MOL004220	N-methyllaurotetanine	Muscarinic acetylcholine receptor M5	CHRM5
Rhizoma C	MOL004220	N-methyllaurotetanine	Prostaglandin G/H synthase 2	PTGS2
Rhizoma C	MOL004220	N-methyllaurotetanine	Retinoic acid receptor RXR-alpha	RXRA
Rhizoma C	MOL004220	N-methyllaurotetanine	Delta-type opioid receptor	OPRD1
Rhizoma C	MOL004220	N-methyllaurotetanine	Alpha-1B adrenergic receptor	ADRA1B
Rhizoma C	MOL004220	N-methyllaurotetanine	Beta-2 adrenergic receptor	ADRB2
Rhizoma C	MOL004220	N-methyllaurotetanine	Alpha-1D adrenergic receptor	ADRA1D

Rhizoma C	MOL004220	N-methyllaurotetanine	Mu-type opioid receptor	OPRM1
Rhizoma C	MOL004221	norglaucing	Prostaglandin G/H synthase 1	PTGS1
Rhizoma C	MOL004221	norglaucing	Muscarinic acetylcholine receptor M3	CHRM3
Rhizoma C	MOL004221	norglaucing	Potassium voltage-gated channel subfamily H member 2	KCNH2
Rhizoma C	MOL004221	norglaucing	Muscarinic acetylcholine receptor M1	CHRM1
Rhizoma C	MOL004221	norglaucing	Androgen receptor	AR
Rhizoma C	MOL004221	norglaucing	Sodium channel protein type 5 subunit alpha	SCN5A
Rhizoma C	MOL004221	norglaucing	Muscarinic acetylcholine receptor M5	CHRM5
Rhizoma C	MOL004221	norglaucing	Prostaglandin G/H synthase 2	PTGS2
Rhizoma C	MOL004221	norglaucing	Retinoic acid receptor RXR-alpha	RXRA
Rhizoma C	MOL004221	norglaucing	Delta-type opioid receptor	OPRD1
Rhizoma C	MOL004221	norglaucing	Acetylcholinesterase	ACHE
Rhizoma C	MOL004221	norglaucing	Alpha-1B adrenergic receptor	ADRA1B

Rhizoma C	MOL004221	norglauc ing	Beta-2 adrenergic receptor	ADRB2
Rhizoma C	MOL004221	norglauc ing	Alpha-1D adrenergic receptor	ADRA1 D
Rhizoma C	MOL004221	norglauc ing	Mu-type opioid receptor	OPRM1
Rhizoma C	MOL004221	norglauc ing	Retinoic acid receptor RXR-beta	RXRB
Rhizoma C	MOL004221	norglauc ing	Nuclear receptor coactivator 1	NCOA1
Rhizoma C	MOL004224	ponteve drine	Prostaglandin G/H synthase 1	PTGS1
Rhizoma C	MOL004224	ponteve drine	Potassium voltage-gated channel subfamily H member 2	KCNH2
Rhizoma C	MOL004224	ponteve drine	Prostaglandin G/H synthase 2	PTGS2
Rhizoma C	MOL004224	ponteve drine	Nuclear receptor coactivator 2	NCOA2
Rhizoma C	MOL004225	pseudoc optisine	Nitric oxide synthase, inducible	NOS2
Rhizoma C	MOL004225	pseudoc optisine	Prostaglandin G/H synthase 1	PTGS1
Rhizoma C	MOL004225	pseudoc optisine	Estrogen receptor	ESR1
Rhizoma C	MOL004225	pseudoc optisine	Androgen receptor	AR
Rhizoma C	MOL004225	pseudoc optisine	Prostaglandin G/H synthase 2	PTGS2

Rhizoma C	MOL004225	pseudoc optisine	Trypsin-1	PRSS1
Rhizoma C	MOL004226	24240-05-9	Prostaglandin G/H synthase 1	PTGS1
Rhizoma C	MOL004226	24240-05-9	Muscarinic acetylcholine receptor M3	CHRM3
Rhizoma C	MOL004226	24240-05-9	Potassium voltage-gated channel subfamily H member 2	KCNH2
Rhizoma C	MOL004226	24240-05-9	Muscarinic acetylcholine receptor M1	CHRM1
Rhizoma C	MOL004226	24240-05-9	Sodium channel protein type 5 subunit alpha	SCN5A
Rhizoma C	MOL004226	24240-05-9	Muscarinic acetylcholine receptor M5	CHRM5
Rhizoma C	MOL004226	24240-05-9	Prostaglandin G/H synthase 2	PTGS2
Rhizoma C	MOL004226	24240-05-9	5-hydroxytryptamine receptor 3A	HTR3A
Rhizoma C	MOL004226	24240-05-9	Coagulation factor VII	F7
Rhizoma C	MOL004226	24240-05-9	Delta-type opioid receptor	OPRD1
Rhizoma C	MOL004226	24240-05-9	Alpha-1B adrenergic receptor	ADRA1B
Rhizoma C	MOL004226	24240-05-9	Alpha-1D adrenergic receptor	ADRA1D
Rhizoma C	MOL004226	24240-05-9	Mu-type opioid receptor	OPRM1

Rhizoma C	MOL004228	saulatine	Muscarinic acetylcholine receptor M3	CHRM3
Rhizoma C	MOL004228	saulatine	Potassium voltage-gated channel subfamily H member 2	KCNH2
Rhizoma C	MOL004228	saulatine	Sodium channel protein type 5 subunit alpha	SCN5A
Rhizoma C	MOL004228	saulatine	Muscarinic acetylcholine receptor M5	CHRM5
Rhizoma C	MOL004228	saulatine	Prostaglandin G/H synthase 2	PTGS2
Rhizoma C	MOL004228	saulatine	Alpha-1B adrenergic receptor	ADRA1B
Rhizoma C	MOL004230	stylopine	Prostaglandin G/H synthase 1	PTGS1
Rhizoma C	MOL004230	stylopine	Muscarinic acetylcholine receptor M3	CHRM3
Rhizoma C	MOL004230	stylopine	Muscarinic acetylcholine receptor M1	CHRM1
Rhizoma C	MOL004230	stylopine	Sodium channel protein type 5 subunit alpha	SCN5A
Rhizoma C	MOL004230	stylopine	Muscarinic acetylcholine receptor M5	CHRM5
Rhizoma C	MOL004230	stylopine	Prostaglandin G/H synthase 2	PTGS2
Rhizoma C	MOL004230	stylopine	5-hydroxytryptamine receptor 3A	HTR3A
Rhizoma C	MOL004230	stylopine	Muscarinic acetylcholine receptor M4	CHRM4

Rhizoma C	MOL004230	stylopine	Delta-type opioid receptor	OPRD1
Rhizoma C	MOL004230	stylopine	Alpha-1B adrenergic receptor	ADRA1B
Rhizoma C	MOL004230	stylopine	Sodium-dependent dopamine transporter	SLC6A3
Rhizoma C	MOL004230	stylopine	Beta-2 adrenergic receptor	ADRB2
Rhizoma C	MOL004230	stylopine	Alpha-1D adrenergic receptor	ADRA1D
Rhizoma C	MOL004230	stylopine	Mu-type opioid receptor	OPRM1
Rhizoma C	MOL004230	stylopine	Retinoic acid receptor RXR-alpha	RXRA
Rhizoma C	MOL004231	Tetrahydrocorysamine	Prostaglandin G/H synthase 1	PTGS1
Rhizoma C	MOL004231	Tetrahydrocorysamine	Muscarinic acetylcholine receptor M3	CHRM3
Rhizoma C	MOL004231	Tetrahydrocorysamine	Potassium voltage-gated channel subfamily H member 2	KCNH2
Rhizoma C	MOL004231	Tetrahydrocorysamine	Muscarinic acetylcholine receptor M1	CHRM1
Rhizoma C	MOL004231	Tetrahydrocorysamine	Sodium channel protein type 5 subunit alpha	SCN5A

Rhizoma C	MOL004231	Tetrahydrocorysamine	Muscarinic acetylcholine receptor M5	CHRM5
Rhizoma C	MOL004231	Tetrahydrocorysamine	Prostaglandin G/H synthase 2	PTGS2
Rhizoma C	MOL004231	Tetrahydrocorysamine	Muscarinic acetylcholine receptor M4	CHRM4
Rhizoma C	MOL004231	Tetrahydrocorysamine	Retinoic acid receptor RXR-alpha	RXRA
Rhizoma C	MOL004231	Tetrahydrocorysamine	Delta-type opioid receptor	OPRD1
Rhizoma C	MOL004231	Tetrahydrocorysamine	Alpha-1B adrenergic receptor	ADRA1B
Rhizoma C	MOL004231	Tetrahydrocorysamine	Beta-2 adrenergic receptor	ADRB2
Rhizoma C	MOL004231	Tetrahydrocorysamine	Alpha-1D adrenergic receptor	ADRA1D
Rhizoma C	MOL004231	Tetrahydrocorysamine	Mu-type opioid receptor	OPRM1
Rhizoma C	MOL004232	tetrahydroprotopapaverine	Muscarinic acetylcholine receptor M3	CHRM3

Rhizoma C	MOL004232	tetrahydr oprotopa paverine	Potassium voltage-gated channel subfamily H member 2	KCNH2
Rhizoma C	MOL004232	tetrahydr oprotopa paverine	Muscarinic acetylcholine receptor M1	CHRM1
Rhizoma C	MOL004232	tetrahydr oprotopa paverine	Beta-1 adrenergic receptor	ADRB1
Rhizoma C	MOL004232	tetrahydr oprotopa paverine	Sodium channel protein type 5 subunit alpha	SCN5A
Rhizoma C	MOL004232	tetrahydr oprotopa paverine	Muscarinic acetylcholine receptor M5	CHRM5
Rhizoma C	MOL004232	tetrahydr oprotopa paverine	Prostaglandin G/H synthase 2	PTGS2
Rhizoma C	MOL004232	tetrahydr oprotopa paverine	Alpha-2C adrenergic receptor	ADRA2 C
Rhizoma C	MOL004232	tetrahydr oprotopa paverine	Muscarinic acetylcholine receptor M4	CHRM4
Rhizoma C	MOL004232	tetrahydr oprotopa paverine	Retinoic acid receptor RXR-alpha	RXRA
Rhizoma C	MOL004232	tetrahydr oprotopa paverine	Delta-type opioid receptor	OPRD1

Rhizoma C	MOL004232	tetrahydroprotopapaverine	Sodium-dependent noradrenaline transporter	SLC6A2
Rhizoma C	MOL004232	tetrahydroprotopapaverine	Alpha-1B adrenergic receptor	ADRA1B
Rhizoma C	MOL004232	tetrahydroprotopapaverine	Sodium-dependent dopamine transporter	SLC6A3
Rhizoma C	MOL004232	tetrahydroprotopapaverine	Beta-2 adrenergic receptor	ADRB2
Rhizoma C	MOL004232	tetrahydroprotopapaverine	Alpha-1D adrenergic receptor	ADRA1D
Rhizoma C	MOL004232	tetrahydroprotopapaverine	Sodium-dependent serotonin transporter	SLC6A4
Rhizoma C	MOL004232	tetrahydroprotopapaverine	Mu-type opioid receptor	OPRM1
Rhizoma C	MOL004233	ST057701	Prostaglandin G/H synthase 1	PTGS1
Rhizoma C	MOL004233	ST057701	Muscarinic acetylcholine receptor M3	CHRM3
Rhizoma C	MOL004233	ST057701	Potassium voltage-gated channel subfamily H member 2	KCNH2
Rhizoma C	MOL004233	ST057701	Muscarinic acetylcholine receptor M1	CHRM1

Rhizoma C	MOL004233	ST057701	Androgen receptor	AR
Rhizoma C	MOL004233	ST057701	Sodium channel protein type 5 subunit alpha	SCN5A
Rhizoma C	MOL004233	ST057701	Muscarinic acetylcholine receptor M5	CHRM5
Rhizoma C	MOL004233	ST057701	Prostaglandin G/H synthase 2	PTGS2
Rhizoma C	MOL004233	ST057701	Muscarinic acetylcholine receptor M4	CHRM4
Rhizoma C	MOL004233	ST057701	Retinoic acid receptor RXR-alpha	RXRA
Rhizoma C	MOL004233	ST057701	Delta-type opioid receptor	OPRD1
Rhizoma C	MOL004233	ST057701	Alpha-1B adrenergic receptor	ADRA1B
Rhizoma C	MOL004233	ST057701	Sodium-dependent dopamine transporter	SLC6A3
Rhizoma C	MOL004233	ST057701	Beta-2 adrenergic receptor	ADRB2
Rhizoma C	MOL004233	ST057701	Alpha-1D adrenergic receptor	ADRA1D
Rhizoma C	MOL004233	ST057701	Sodium-dependent serotonin transporter	SLC6A4
Rhizoma C	MOL004233	ST057701	Mu-type opioid receptor	OPRM1
Rhizoma C	MOL004233	ST057701	Trypsin-1	PRSS1

Rhizoma C	MOL004234	2,3,9,10-tetramethoxy-13-methyl-5,6-dihydroisoquinolino[2,1-b]isoquinolin-8-one	Nitric oxide synthase, inducible	NOS2
Rhizoma C	MOL004234	2,3,9,10-tetramethoxy-13-methyl-5,6-dihydroisoquinolino[2,1-b]isoquinolin-8-one	Prostaglandin G/H synthase 1	PTGS1

Rhizoma C	MOL004234	2,3,9,10-tetramethoxy-13-methyl-5,6-dihydroisoquinolino[2,1-b]isoquinolin-8-one	Potassium voltage-gated channel subfamily H member 2	KCNH2
Rhizoma C	MOL004234	2,3,9,10-tetramethoxy-13-methyl-5,6-dihydroisoquinolino[2,1-b]isoquinolin-8-one	Androgen receptor	AR

Rhizoma C	MOL004234	2,3,9,10-tetramethoxy-13-methyl-5,6-dihydroisoquinolino[2,1-b]isoquinolin-8-one	Sodium channel protein type 5 subunit alpha	SCN5A
Rhizoma C	MOL004234	2,3,9,10-tetramethoxy-13-methyl-5,6-dihydroisoquinolino[2,1-b]isoquinolin-8-one	Prostaglandin G/H synthase 2	PTGS2

Rhizoma C	MOL004234	2,3,9,10-tetramethoxy-13-methyl-5,6-dihydroisoquinolino[2,1-b]isoquinolin-8-one	Serine/threonine-protein kinase Chk1	CHEK1
Rhizoma C	MOL004234	2,3,9,10-tetramethoxy-13-methyl-5,6-dihydroisoquinolino[2,1-b]isoquinolin-8-one	Trypsin-1	PRSS1

Rhizoma C	MOL004234	2,3,9,10-tetramethoxy-13-methyl-5,6-dihydroisoquinolino[2,1-b]isoquinolin-8-one	Nuclear receptor coactivator 2	NCOA2
Rhizoma C	MOL000449	Stigmasterol	Progesterone receptor	PGR
Rhizoma C	MOL000449	Stigmasterol	Mineralocorticoid receptor	NR3C2
Rhizoma C	MOL000449	Stigmasterol	Nuclear receptor coactivator 2	NCOA2
Rhizoma C	MOL000449	Stigmasterol	Alcohol dehydrogenase 1C	ADH1C
Rhizoma C	MOL000449	Stigmasterol	Retinoic acid receptor RXR-alpha	RXRA
Rhizoma C	MOL000449	Stigmasterol	Nuclear receptor coactivator 1	NCOA1
Rhizoma C	MOL000449	Stigmasterol	Prostaglandin G/H synthase 1	PTGS1
Rhizoma C	MOL000449	Stigmasterol	Prostaglandin G/H synthase 2	PTGS2

Rhizoma C	MOL000449	Stigmasterol	Alpha-2A adrenergic receptor	ADRA2A
Rhizoma C	MOL000449	Stigmasterol	Sodium-dependent noradrenaline transporter	SLC6A2
Rhizoma C	MOL000449	Stigmasterol	Sodium-dependent dopamine transporter	SLC6A3
Rhizoma C	MOL000449	Stigmasterol	Beta-2 adrenergic receptor	ADRB2
Rhizoma C	MOL000449	Stigmasterol	Aldose reductase	AKR1B1
Rhizoma C	MOL000449	Stigmasterol	Urokinase-type plasminogen activator	PLAU
Rhizoma C	MOL000449	Stigmasterol	Leukotriene A-4 hydrolase	LTA4H
Rhizoma C	MOL000449	Stigmasterol	Amine oxidase [flavin-containing] B	MAOB
Rhizoma C	MOL000449	Stigmasterol	Amine oxidase [flavin-containing] A	MAOA
Rhizoma C	MOL000449	Stigmasterol	Chymotrypsinogen B	CTRB1
Rhizoma C	MOL000449	Stigmasterol	Muscarinic acetylcholine receptor M3	CHRM3
Rhizoma C	MOL000449	Stigmasterol	Muscarinic acetylcholine receptor M1	CHRM1
Rhizoma C	MOL000449	Stigmasterol	Beta-1 adrenergic receptor	ADRB1
Rhizoma C	MOL000449	Stigmasterol	Sodium channel protein type 5 subunit alpha	SCN5A
Rhizoma C	MOL000449	Stigmasterol	Alpha-1A adrenergic receptor	ADRA1A

Rhizoma C	MOL000449	Stigmasterol	Muscarinic acetylcholine receptor M2	CHRM2
Rhizoma C	MOL000449	Stigmasterol	Alpha-1B adrenergic receptor	ADRA1B
Rhizoma C	MOL000449	Stigmasterol	Gamma-aminobutyric acid receptor subunit alpha-1	GABRA1
Rhizoma C	MOL000785	palmatine	Nitric oxide synthase, inducible	NOS2
Rhizoma C	MOL000785	palmatine	Prostaglandin G/H synthase 1	PTGS1
Rhizoma C	MOL000785	palmatine	Potassium voltage-gated channel subfamily H member 2	KCNH2
Rhizoma C	MOL000785	palmatine	Estrogen receptor	ESR1
Rhizoma C	MOL000785	palmatine	Androgen receptor	AR
Rhizoma C	MOL000785	palmatine	Sodium channel protein type 5 subunit alpha	SCN5A
Rhizoma C	MOL000785	palmatine	Prostaglandin G/H synthase 2	PTGS2
Rhizoma C	MOL000785	palmatine	Retinoic acid receptor RXR-alpha	RXRA
Rhizoma C	MOL000785	palmatine	Beta-2 adrenergic receptor	ADRB2
Rhizoma C	MOL000785	palmatine	Estrogen receptor beta	ESR2
Rhizoma C	MOL000785	palmatine	Trypsin-1	PRSS1

Rhizoma C	MOL000785	palmatine	Nuclear receptor coactivator 2	NCOA2
Rhizoma C	MOL000785	palmatine	Coagulation factor VII	F7
Rhizoma C	MOL000787	Fumarine	Prostaglandin G/H synthase 1	PTGS1
Rhizoma C	MOL000787	Fumarine	Muscarinic acetylcholine receptor M3	CHRM3
Rhizoma C	MOL000787	Fumarine	Potassium voltage-gated channel subfamily H member 2	KCNH2
Rhizoma C	MOL000787	Fumarine	Muscarinic acetylcholine receptor M1	CHRM1
Rhizoma C	MOL000787	Fumarine	Sodium channel protein type 5 subunit alpha	SCN5A
Rhizoma C	MOL000787	Fumarine	Muscarinic acetylcholine receptor M5	CHRM5
Rhizoma C	MOL000787	Fumarine	Prostaglandin G/H synthase 2	PTGS2
Rhizoma C	MOL000787	Fumarine	5-hydroxytryptamine receptor 3A	HTR3A
Rhizoma C	MOL000787	Fumarine	Coagulation factor VII	F7
Rhizoma C	MOL000787	Fumarine	Muscarinic acetylcholine receptor M4	CHRM4
Rhizoma C	MOL000787	Fumarine	Delta-type opioid receptor	OPRD1
Rhizoma C	MOL000787	Fumarine	Alpha-1B adrenergic receptor	ADRA1B

Rhizoma C	MOL000787	Fumarine	Beta-2 adrenergic receptor	ADRB2
Rhizoma C	MOL000787	Fumarine	Alpha-1D adrenergic receptor	ADRA1D
Rhizoma C	MOL000787	Fumarine	Mu-type opioid receptor	OPRM1
Rhizoma C	MOL000787	Fumarine	Sodium-dependent serotonin transporter	SLC6A4
Rhizoma C	MOL000787	Fumarine	Voltage-dependent L-type calcium channel subunit alpha-1S	CACNA1S
Rhizoma C	MOL000787	Fumarine	Sodium-dependent dopamine transporter	SLC6A3
Rhizoma C	MOL000787	Fumarine	Vascular endothelial growth factor receptor 2	KDR
Rhizoma C	MOL000790	Isocorypalmine	Prostaglandin G/H synthase 1	PTGS1
Rhizoma C	MOL000790	Isocorypalmine	Muscarinic acetylcholine receptor M3	CHRM3
Rhizoma C	MOL000790	Isocorypalmine	Potassium voltage-gated channel subfamily H member 2	KCNH2
Rhizoma C	MOL000790	Isocorypalmine	Muscarinic acetylcholine receptor M1	CHRM1
Rhizoma C	MOL000790	Isocorypalmine	D(1B) dopamine receptor	DRD5
Rhizoma C	MOL000790	Isocorypalmine	Sodium channel protein type 5 subunit alpha	SCN5A
Rhizoma C	MOL000790	Isocorypalmine	Muscarinic acetylcholine receptor M5	CHRM5

Rhizoma C	MOL000790	Isocorypalmine	Prostaglandin G/H synthase 2	PTGS2
Rhizoma C	MOL000790	Isocorypalmine	5-hydroxytryptamine receptor 3A	HTR3A
Rhizoma C	MOL000790	Isocorypalmine	Alpha-2C adrenergic receptor	ADRA2C
Rhizoma C	MOL000790	Isocorypalmine	Muscarinic acetylcholine receptor M4	CHRM4
Rhizoma C	MOL000790	Isocorypalmine	Retinoic acid receptor RXR-alpha	RXRA
Rhizoma C	MOL000790	Isocorypalmine	Delta-type opioid receptor	OPRD1
Rhizoma C	MOL000790	Isocorypalmine	Sodium-dependent noradrenaline transporter	SLC6A2
Rhizoma C	MOL000790	Isocorypalmine	Alpha-1A adrenergic receptor	ADRA1A
Rhizoma C	MOL000790	Isocorypalmine	Muscarinic acetylcholine receptor M2	CHRM2
Rhizoma C	MOL000790	Isocorypalmine	Alpha-2B adrenergic receptor	ADRA2B
Rhizoma C	MOL000790	Isocorypalmine	Alpha-1B adrenergic receptor	ADRA1B
Rhizoma C	MOL000790	Isocorypalmine	D(3) dopamine receptor	DRD3
Rhizoma C	MOL000790	Isocorypalmine	Sodium-dependent dopamine transporter	SLC6A3
Rhizoma C	MOL000790	Isocorypalmine	Beta-2 adrenergic receptor	ADRB2
Rhizoma C	MOL000790	Isocorypalmine	Alpha-1D adrenergic receptor	ADRA1D

Rhizoma C	MOL000790	Isocorypalmine	Sodium-dependent serotonin transporter	SLC6A4
Rhizoma C	MOL000790	Isocorypalmine	Mu-type opioid receptor	OPRM1
Rhizoma C	MOL000790	Isocorypalmine	Retinoic acid receptor RXR-beta	RXRB
Rhizoma C	MOL000790	Isocorypalmine	Nuclear receptor coactivator 1	NCOA1
Rhizoma C	MOL000791	bicuculline	Prostaglandin G/H synthase 1	PTGS1
Rhizoma C	MOL000791	bicuculline	Potassium voltage-gated channel subfamily H member 2	KCNH2
Rhizoma C	MOL000791	bicuculline	Androgen receptor	AR
Rhizoma C	MOL000791	bicuculline	Sodium channel protein type 5 subunit alpha	SCN5A
Rhizoma C	MOL000791	bicuculline	Prostaglandin G/H synthase 2	PTGS2
Rhizoma C	MOL000791	bicuculline	Vascular endothelial growth factor receptor 2	KDR
Rhizoma C	MOL000791	bicuculline	Acetylcholinesterase	ACHE
Rhizoma C	MOL000791	bicuculline	Proto-oncogene c-Fos	FOS
Rhizoma C	MOL000791	bicuculline	Gap junction alpha-1 protein	GJA1
Rhizoma C	MOL000791	bicuculline	Gamma-aminobutyric acid type B receptor subunit 1	GABBR1

Rhizoma C	MOL000791	bicuculline	Bone morphogenetic protein receptor type-2	BMPR2
Rhizoma C	MOL000791	bicuculline	Metabotropic glutamate receptor 5	GRM5
Rhizoma C	MOL000791	bicuculline	Progonadoliberin-1	GNRH1
Rhizoma C	MOL000791	bicuculline	Aldehyde dehydrogenase, dimeric NADP-preferring	ALDH3A1
Rhizoma C	MOL000791	bicuculline	Gonadotropin-releasing hormone receptor	GNRHR
Rhizoma C	MOL000791	bicuculline	Corticoliberin	CRH
Rhizoma C	MOL000791	bicuculline	Sodium-dependent noradrenaline transporter	SLC6A2
Rhizoma C	MOL000791	bicuculline	Gap junction beta-1 protein	GJB1
Rhizoma C	MOL000791	bicuculline	Metabotropic glutamate receptor 1	GRM1
Rhizoma C	MOL000791	bicuculline	Transitional endoplasmic reticulum ATPase	VCP
Rhizoma C	MOL000793	C09367	Prostaglandin G/H synthase 1	PTGS1
Rhizoma C	MOL000793	C09367	Muscarinic acetylcholine receptor M3	CHRM3
Rhizoma C	MOL000793	C09367	Potassium voltage-gated channel subfamily H member 2	KCNH2
Rhizoma C	MOL000793	C09367	Muscarinic acetylcholine receptor M1	CHRM1
Rhizoma C	MOL000793	C09367	Androgen receptor	AR

Rhizoma C	MOL000793	C09367	Sodium channel protein type 5 subunit alpha	SCN5A
Rhizoma C	MOL000793	C09367	Muscarinic acetylcholine receptor M5	CHRM5
Rhizoma C	MOL000793	C09367	Prostaglandin G/H synthase 2	PTGS2
Rhizoma C	MOL000793	C09367	Retinoic acid receptor RXR-alpha	RXRA
Rhizoma C	MOL000793	C09367	Delta-type opioid receptor	OPRD1
Rhizoma C	MOL000793	C09367	Alpha-1B adrenergic receptor	ADRA1B
Rhizoma C	MOL000793	C09367	Beta-2 adrenergic receptor	ADRB2
Rhizoma C	MOL000793	C09367	Alpha-1D adrenergic receptor	ADRA1D
Rhizoma C	MOL000793	C09367	Mu-type opioid receptor	OPRM1
Rhizoma C	MOL000793	C09367	Muscarinic acetylcholine receptor M4	CHRM4
Rhizoma C	MOL000098	quercetin	Prostaglandin G/H synthase 1	PTGS1
Rhizoma C	MOL000098	quercetin	Androgen receptor	AR
Rhizoma C	MOL000098	quercetin	Peroxisome proliferator activated receptor gamma	PPARG
Rhizoma C	MOL000098	quercetin	Prostaglandin G/H synthase 2	PTGS2
Rhizoma C	MOL000098	quercetin	Nuclear receptor coactivator 2	NCOA2
Rhizoma C	MOL000098	quercetin	Aldose reductase	AKR1B1

Rhizoma C	MOL000098	quercetin	Trypsin-1	PRSS1
Rhizoma C	MOL000098	quercetin	Potassium voltage-gated channel subfamily H member 2	KCNH2
Rhizoma C	MOL000098	quercetin	Sodium channel protein type 5 subunit alpha	SCN5A
Rhizoma C	MOL000098	quercetin	Beta-2 adrenergic receptor	ADRB2
Rhizoma C	MOL000098	quercetin	Stromelysin-1	MMP3
Rhizoma C	MOL000098	quercetin	Coagulation factor VII	F7
Rhizoma C	MOL000098	quercetin	Retinoic acid receptor RXR-alpha	RXRA
Rhizoma C	MOL000098	quercetin	Acetylcholinesterase	ACHE
Rhizoma C	MOL000098	quercetin	Gamma-aminobutyric acid receptor subunit alpha-1	GABRA1
Rhizoma C	MOL000098	quercetin	Amine oxidase [flavin-containing] B	MAOB
Rhizoma C	MOL000098	quercetin	Transcription factor p65	RELA
Rhizoma C	MOL000098	quercetin	Epidermal growth factor receptor	EGFR
Rhizoma C	MOL000098	quercetin	RAC-alpha serine/threonine-protein kinase	AKT1
Rhizoma C	MOL000098	quercetin	Vascular endothelial growth factor A	VEGFA

Rhizoma C	MOL000098	querceti n	G1/S-specific cyclin-D1	CCND1
Rhizoma C	MOL000098	querceti n	Apoptosis regulator Bcl-2	BCL2
Rhizoma C	MOL000098	querceti n	Bcl-2-like protein 1	BCL2L1
Rhizoma C	MOL000098	querceti n	Proto-oncogene c-Fos	FOS
Rhizoma C	MOL000098	querceti n	Cyclin-dependent kinase inhibitor 1	CDKN1 A
Rhizoma C	MOL000098	querceti n	Eukaryotic translation initiation factor 6	EIF6
Rhizoma C	MOL000098	querceti n	Apoptosis regulator BAX	BAX
Rhizoma C	MOL000098	querceti n	Caspase-9	CASP9
Rhizoma C	MOL000098	querceti n	Urokinase-type plasminogen activator	PLAU
Rhizoma C	MOL000098	querceti n	72 kDa type IV collagenase	MMP2
Rhizoma C	MOL000098	querceti n	Matrix metalloproteinase-9	MMP9
Rhizoma C	MOL000098	querceti n	Mitogen-activated protein kinase 1	MAPK1
Rhizoma C	MOL000098	querceti n	Interleukin-10	IL10
Rhizoma C	MOL000098	querceti n	Pro-epidermal growth factor	EGF
Rhizoma C	MOL000098	querceti n	Retinoblastoma-associated protein	RB1

Rhizoma C	MOL000098	querceti n	Tumor necrosis factor	TNFSF1 5
Rhizoma C	MOL000098	querceti n	Transcription factor AP-1	JUN
Rhizoma C	MOL000098	querceti n	Interleukin-6	IL6
Rhizoma C	MOL000098	querceti n	Activator of 90 kDa heat shock protein ATPase homolog 1	AHSA1
Rhizoma C	MOL000098	querceti n	Caspase-3	CASP3
Rhizoma C	MOL000098	querceti n	Cellular tumor antigen p53	TP63
Rhizoma C	MOL000098	querceti n	ETS domain-containing protein Elk-1	ELK1
Rhizoma C	MOL000098	querceti n	NF-kappa-B inhibitor alpha	NFKBI A
Rhizoma C	MOL000098	querceti n	NADPH--cytochrome P450 reductase	POR
Rhizoma C	MOL000098	querceti n	Ornithine decarboxylase	ODC1
Rhizoma C	MOL000098	querceti n	Caspase-8	CASP8
Rhizoma C	MOL000098	querceti n	DNA topoisomerase 1	TOP1
Rhizoma C	MOL000098	querceti n	RAF proto-oncogene serine/threonine-protein kinase	RAF1
Rhizoma C	MOL000098	querceti n	Superoxide dismutase [Cu-Zn]	SOD1

Rhizoma C	MOL000098	quercetin	Protein kinase C alpha type	PRKCA
Rhizoma C	MOL000098	quercetin	Interstitial collagenase	MMP1
Rhizoma C	MOL000098	quercetin	Hypoxia-inducible factor 1-alpha	HIF1A
Rhizoma C	MOL000098	quercetin	Signal transducer and activator of transcription 1-alpha/beta	STAT1
Rhizoma C	MOL000098	quercetin	Protein CBFA2T1	RUNX1T1
Rhizoma C	MOL000098	quercetin	Receptor tyrosine-protein kinase erbB-2	ERBB2
Rhizoma C	MOL000098	quercetin	Peroxisome proliferator-activated receptor gamma	PPARG
Rhizoma C	MOL000098	quercetin	Acetyl-CoA carboxylase 1	ACACA
Rhizoma C	MOL000098	quercetin	Heme oxygenase 1	HMOX1
Rhizoma C	MOL000098	quercetin	Cytochrome P450 3A4	CYP3A4
Rhizoma C	MOL000098	quercetin	Cytochrome P450 1A2	CYP1A2
Rhizoma C	MOL000098	quercetin	Caveolin-1	CAV1
Rhizoma C	MOL000098	quercetin	Myc proto-oncogene protein	MYC
Rhizoma C	MOL000098	quercetin	Tissue factor	F3

Rhizoma C	MOL000098	querceti n	Gap junction alpha-1 protein	GJA1
Rhizoma C	MOL000098	querceti n	Cytochrome P450 1A1	CYP1A 1
Rhizoma C	MOL000098	querceti n	Intercellular adhesion molecule 1	ICAM1
Rhizoma C	MOL000098	querceti n	Interleukin-1 beta	IL1B
Rhizoma C	MOL000098	querceti n	C-C motif chemokine 2	CCL2
Rhizoma C	MOL000098	querceti n	E-selectin	SELE
Rhizoma C	MOL000098	querceti n	Vascular cell adhesion protein 1	VCAM1
Rhizoma C	MOL000098	querceti n	Prostaglandin E2 receptor EP3 subtype	PTGER3
Rhizoma C	MOL000098	querceti n	Interleukin-8	CXCL8
Rhizoma C	MOL000098	querceti n	Protein kinase C beta type	PRKCB
Rhizoma C	MOL000098	querceti n	Baculoviral IAP repeat-containing protein 5	BIRC5
Rhizoma C	MOL000098	querceti n	Dual oxidase 2	DUOX2
Rhizoma C	MOL000098	querceti n	Nitric oxide synthase, endothelial	NOS3
Rhizoma C	MOL000098	querceti n	Heat shock protein beta-1	HSPB1
Rhizoma C	MOL000098	querceti n	Estrogen sulfotransferase	SULT1E 1

Rhizoma C	MOL000098	querceti n	Maltase-glucoamylase, intestinal	MGAM
Rhizoma C	MOL000098	querceti n	Interleukin-2	IL2
Rhizoma C	MOL000098	querceti n	Nuclear receptor subfamily 1 group I member 2	NR1I2
Rhizoma C	MOL000098	querceti n	Cytochrome P450 1B1	CYP1B1
Rhizoma C	MOL000098	querceti n	G2/mitotic-specific cyclin-B1	CCNB1
Rhizoma C	MOL000098	querceti n	Tissue-type plasminogen activator	PLAT
Rhizoma C	MOL000098	querceti n	Thrombomodulin	THBD
Rhizoma C	MOL000098	querceti n	Plasminogen activator inhibitor 1	SERPIN E1
Rhizoma C	MOL000098	querceti n	Collagen alpha-1(I) chain	COL1A 1
Rhizoma C	MOL000098	querceti n	Interferon gamma	IFNG
Rhizoma C	MOL000098	querceti n	Arachidonate 5-lipoxygenase	ALOX5
Rhizoma C	MOL000098	querceti n	Interleukin-1 alpha	IL1A
Rhizoma C	MOL000098	querceti n	Myeloperoxidase	MPO
Rhizoma C	MOL000098	querceti n	DNA topoisomerase 2-alpha	TOP2A
Rhizoma C	MOL000098	querceti n	Neutrophil cytosol factor 1	NCF1

Rhizoma C	MOL000098	querceti n	ATP-binding cassette sub-family G member 2	ABCG2
Rhizoma C	MOL000098	querceti n	Hyaluronan synthase 2	HAS2
Rhizoma C	MOL000098	querceti n	Glutathione S-transferase P	GSTP1
Rhizoma C	MOL000098	querceti n	Nuclear factor erythroid 2-related factor 2	NFE2L2
Rhizoma C	MOL000098	querceti n	NAD(P)H dehydrogenase [quinone] 1	NQO1
Rhizoma C	MOL000098	querceti n	Poly [ADP-ribose] polymerase 1	PARP1
Rhizoma C	MOL000098	querceti n	Aryl hydrocarbon receptor	AHR
Rhizoma C	MOL000098	querceti n	26S proteasome non-ATPase regulatory subunit 3	PSMD3
Rhizoma C	MOL000098	querceti n	Solute carrier family 2, facilitated glucose transporter member 4	SLC2A4
Rhizoma C	MOL000098	querceti n	Collagen alpha-1(III) chain	COL3A 1
Rhizoma C	MOL000098	querceti n	C-X-C motif chemokine 11	CXCL11
Rhizoma C	MOL000098	querceti n	C-X-C motif chemokine 2	CXCL2
Rhizoma C	MOL000098	querceti n	DDB1- and CUL4-associated factor 5	DCAF5
Rhizoma C	MOL000098	querceti n	Nuclear receptor subfamily 1 group I member 3	NR1I3

Rhizoma C	MOL000098	querceti n	Serine/threonine-protein kinase Chk2	CHEK2
Rhizoma C	MOL000098	querceti n	Insulin receptor	INSR
Rhizoma C	MOL000098	querceti n	Claudin-4	CLDN4
Rhizoma C	MOL000098	querceti n	Peroxisome proliferator-activated receptor alpha	PPARA
Rhizoma C	MOL000098	querceti n	Peroxisome proliferator-activated receptor delta	PPARD
Rhizoma C	MOL000098	querceti n	Heat shock factor protein 1	HSF1
Rhizoma C	MOL000098	querceti n	C-reactive protein	CRP
Rhizoma C	MOL000098	querceti n	C-X-C motif chemokine 10	CXCL10
Rhizoma C	MOL000098	querceti n	Inhibitor of nuclear factor kappa-B kinase subunit alpha	CHUK
Rhizoma C	MOL000098	querceti n	Osteopontin	SPP1
Rhizoma C	MOL000098	querceti n	Runt-related transcription factor 2	RUNX2
Rhizoma C	MOL000098	querceti n	Ras association domain-containing protein 1	RASSF1
Rhizoma C	MOL000098	querceti n	Transcription factor E2F1	E2F1
Rhizoma C	MOL000098	querceti n	Transcription factor E2F2	E2F2

Rhizoma C	MOL000098	quercetin	Prostatic acid phosphatase	ACPP
Rhizoma C	MOL000098	quercetin	Cathepsin D	CTSD
Rhizoma C	MOL000098	quercetin	Insulin-like growth factor-binding protein 3	IGFBP3
Rhizoma C	MOL000098	quercetin	Insulin-like growth factor II	IGF2
Rhizoma C	MOL000098	quercetin	CD40 ligand	CD40LG
Rhizoma C	MOL000098	quercetin	Interferon regulatory factor 1	IRF1
Rhizoma C	MOL000098	quercetin	Receptor tyrosine-protein kinase erbB-3	ERBB3
Rhizoma C	MOL000098	quercetin	Serum paraoxonase/arylesterase 1	PON1
Rhizoma C	MOL000098	quercetin	Type I iodothyronine deiodinase	DIO1
Rhizoma C	MOL000098	quercetin	Procollagen C-endopeptidase enhancer 1	PCOLCE
Rhizoma C	MOL000098	quercetin	Puromycin-sensitive aminopeptidase	NPEPPS
Rhizoma C	MOL000098	quercetin	Hexokinase-2	HK2
Rhizoma C	MOL000098	quercetin	Ras GTPase-activating protein 1	RASA1
Rhizoma C	MOL000098	quercetin	Glutathione S-transferase Mu 1	GSTM1
Rhizoma C	MOL000098	quercetin	Glutathione S-transferase Mu 2	GSTM2

own therapeutic targets for KOA

Known therapeutic targets from		Known therapeutic targets from Genecards						
ne/Locus MIM numl	Gene/Locus	Gene Symbol	Description	Category	Gifts	GC Id	Relevance score	GeneCards Link
602109	MATN3, EDM5, HOA, OS2, SEMDBCD	**COL2A1**	Collagen Type II Alpha 1 Chain	Protein Coding	48	GC12M047972	69.95	https://www.genecards.org/cgi-bin/carddisp.pl?gene=COL2A1
602109	MATN3, EDM5, HOA, OS2, SEMDBCD	**ACAN**	Aggrecan	Protein Coding	45	GC15P088813	52.54	https://www.genecards.org/cgi-bin/carddisp.pl?gene=ACAN
602109	MATN3, EDM5, HOA, OS2, SEMDBCD	**COMP**	Cartilage Oligomeric Matrix Protein	Protein Coding	47	GC19M018783	50.96	https://www.genecards.org/cgi-bin/carddisp.pl?gene=COMP
605083	FRZB, FRZB1, SRFP3, OS1	**MATN3**	Matrilin 3	Protein Coding	43	GC02M019992	47.76	https://www.genecards.org/cgi-bin/carddisp.pl?gene=MATN3
610839	OS4, GOA1	**SMAD3**	SMAD Family Member 3	Protein Coding	49	GC15P067063	46.5	https://www.genecards.org/cgi-bin/carddisp.pl?gene=SMAD3
612401	OS6	**GDF5**	Growth Differentiation Factor 5	Protein Coding	47	GC20M035433	42.64	https://www.genecards.org/cgi-bin/carddisp.pl?gene=GDF5
600668	CCAL1	**COL9A1**	Collagen Type IX Alpha 1 Chain	Protein Coding	43	GC06M070215	41.89	https://www.genecards.org/cgi-bin/carddisp.pl?gene=COL9A1

608135	ASPN, PLAP1, OS3	**FRZB**	Frizzled Related Protein	Protein Coding	41	GC02M182 833	40.88	https://www.genecards.org/cgi-bin/carddisp.pl?gene=FRZB
608135	ASPN, PLAP1, OS3	**COL11A2**	Collagen Type XI Alpha 2 Chain	Protein Coding	44	GC06M033 162	37.8	https://www.genecards.org/cgi-bin/carddisp.pl?gene=COL11A2
120140	COL2A1	**MMP13**	Matrix Metallopeptidase 13	Protein Coding	50	GC11M102 942	36.94	https://www.genecards.org/cgi-bin/carddisp.pl?gene=MMP13
120140	COL2A1	**ASPN**	Asporin	Protein Coding	39	GC09M092 458	31.79	https://www.genecards.org/cgi-bin/carddisp.pl?gene=ASPN
120140	COL2A1	**COL9A2**	Collagen Type IX Alpha 2 Chain	Protein Coding	42	GC01M040 300	29.9	https://www.genecards.org/cgi-bin/carddisp.pl?gene=COL9A2
120140	COL2A1	**COL9A3**	Collagen Type IX Alpha 3 Chain	Protein Coding	42	GC20P062 816	29.2	https://www.genecards.org/cgi-bin/carddisp.pl?gene=COL9A3
120140	COL2A1	**FBN1**	Fibrillin 1	Protein Coding	45	GC15M048 408	28.06	https://www.genecards.org/cgi-bin/carddisp.pl?gene=FBN1
120140	COL2A1	**TGFB3**	Transforming Growth Factor Beta 3	Protein Coding	47	GC14M075 958	26.63	https://www.genecards.org/cgi-bin/carddisp.pl?gene=TGFB3

120140	COL2A1	**TGFB2**	Transforming Growth Factor Beta 2	Protein Coding	50	GC01P218345	25.06	https://www.genecards.org/cgi-bin/carddisp.pl?gene=TGFB2
120140	COL2A1	**TGFBR1**	Transforming Growth Factor Beta Receptor 1	Protein Coding	52	GC09P099104	24.63	https://www.genecards.org/cgi-bin/carddisp.pl?gene=TGFBR1
120140	COL2A1	**SLC26A2**	Solute Carrier Family 26 Member 2	Protein Coding	44	GC05P149944	24.54	https://www.genecards.org/cgi-bin/carddisp.pl?gene=SLC26A2
120140	COL2A1	**PTGS2**	Prostaglandin-Endoperoxide Synthase 2	Protein Coding	48	GC01M186640	24.2	https://www.genecards.org/cgi-bin/carddisp.pl?gene=PTGS2
120140	COL2A1	**TGFBR2**	Transforming Growth Factor Beta Receptor 2	Protein Coding	51	GC03P030623	23.79	https://www.genecards.org/cgi-bin/carddisp.pl?gene=TGFBR2
120140	COL2A1	**IL1B**	Interleukin 1 Beta	Protein Coding	48	GC02M112829	23.57	https://www.genecards.org/cgi-bin/carddisp.pl?gene=IL1B
120140	COL2A1	**TNF**	Tumor Necrosis Factor	Protein Coding	51	GC06P047305	22.77	https://www.genecards.org/cgi-bin/carddisp.pl?gene=TNF
120140	COL2A1	**COL5A2**	Collagen Type V Alpha 2 Chain	Protein Coding	41	GC02M189031	22.09	https://www.genecards.org/cgi-bin/carddisp.pl?gene=COL5A2
120140	COL2A1	**IL6**	Interleukin 6	Protein Coding	50	GC07P022765	21.79	https://www.genecards.org/cgi-bin/carddisp.pl?gene=IL6

155760	ACAN, AGC1, CSPG1, MSK16, SEDK, SSOAOD	**COL11A1**	Collagen Type XI Alpha 1 Chain	Protein Coding	43	GC01M102876	21.67	https://www.genecards.org/cgi-bin/carddisp.pl?gene=COL11A1
155760	ACAN, AGC1, CSPG1, MSK16, SEDK, SSOAOD	**UFSP2**	UFM1 Specific Peptidase 2	Protein Coding	40	GC04M185399	21.31	https://www.genecards.org/cgi-bin/carddisp.pl?gene=UFSP2
155760	ACAN, AGC1, CSPG1, MSK16, SEDK, SSOAOD	**MMP3**	Matrix Metallopeptidase 3	Protein Coding	51	GC11M102835	21.24	https://www.genecards.org/cgi-bin/carddisp.pl?gene=MMP3
601146	GDF5, CDMP1, SYNS2, OS5, BDA1C, SYM1B, DUPANS	**TGFB1**	Transforming Growth Factor Beta 1	Protein Coding	52	GC19M041301	21.02	https://www.genecards.org/cgi-bin/carddisp.pl?gene=TGFB1
601146	GDF5, CDMP1, SYNS2, OS5, BDA1C, SYM1B, DUPANS	**COL1A1**	Collagen Type I Alpha 1 Chain	Protein Coding	50	GC17M050183	20.65	https://www.genecards.org/cgi-bin/carddisp.pl?gene=COL1A1

601146	GDF5, CDMP1, SYNS2, OS5, BDA1C, SYM1B, DUPANS	**ADAMTS5**	ADAM Metallopeptidase With Thrombospondin Type 1 Motif 5	Protein Coding	42	GC21M026918	20.52	https://www.genecards.org/cgi-bin/carddisp.pl?gene=ADAMTS5
601146	GDF5, CDMP1, SYNS2, OS5, BDA1C, SYM1B, DUPANS	**IL10**	Interleukin 10	Protein Coding	47	GC01M206767	19.85	https://www.genecards.org/cgi-bin/carddisp.pl?gene=IL10
601146	GDF5, CDMP1, SYNS2, OS5, BDA1C, SYM1B, DUPANS	**MMP1**	Matrix Metallopeptidase 1	Protein Coding	51	GC11M102810	19.8	https://www.genecards.org/cgi-bin/carddisp.pl?gene=MMP1
601146	GDF5, CDMP1, SYNS2, OS5, BDA1C, SYM1B, DUPANS	**LOC109461476**	GDF5 Promoter Region	Biological Region	1	GC20P035437	19.36	https://www.genecards.org/cgi-bin/carddisp.pl?gene=LOC109461476
601146	GDF5, CDMP1, SYNS2, OS5, BDA1C, SYM1B, DUPANS	**CILP**	Cartilage Intermediate Layer Protein	Protein Coding	40	GC15M065194	18.9	https://www.genecards.org/cgi-bin/carddisp.pl?gene=CILP

601146	GDF5, CDMP1, SYNS2, OS5, BDA1C, SYM1B, DUPANS	**IL1RN**	Interleukin 1 Receptor Antagonist	Protein Coding	48	GC02P115307	18.79	https://www.genecards.org/cgi-bin/carddisp.pl?gene=IL1RN
601146	GDF5, CDMP1, SYNS2, OS5, BDA1C, SYM1B, DUPANS	**BGLAP**	Bone Gamma-Carboxyglutamate Protein	Protein Coding	40	GC01P156242	18.48	https://www.genecards.org/cgi-bin/carddisp.pl?gene=BGLAP
		**TRPV4**	Transient Receptor Potential Cation Channel Subfamily V Member 4	Protein Coding	49	GC12M109783	18.06	https://www.genecards.org/cgi-bin/carddisp.pl?gene=TRPV4
		**CXCL8**	C-X-C Motif Chemokine Ligand 8	Protein Coding	41	GC04P073740	17.98	https://www.genecards.org/cgi-bin/carddisp.pl?gene=CXCL8
		**ADAMTS4**	ADAM Metallopeptidase With Thrombospondin Type 1 Motif 4	Protein Coding	42	GC01M161184	17.94	https://www.genecards.org/cgi-bin/carddisp.pl?gene=ADAMTS4
		**TRAPPC2**	Trafficking Protein Particle Complex 2	Protein Coding	38	GC0XM013712	17.74	https://www.genecards.org/cgi-bin/carddisp.pl?gene=TRAPPC2
		**IL17A**	Interleukin 17A	Protein Coding	42	GC06P052186	17.68	https://www.genecards.org/cgi-bin/carddisp.pl?gene=IL17A

**TNFSF11**	TNF Superfamily Member 11	Protein Coding	47	GC13P042562	17.68	https://www.genecards.org/cgi-bin/carddisp.pl?gene=TNFSF11
**TNFRSF11B**	TNF Receptor Superfamily Member 11b	Protein Coding	47	GC08M118923	17.15	https://www.genecards.org/cgi-bin/carddisp.pl?gene=TNFRSF11B
**CRP**	C-Reactive Protein	Protein Coding	46	GC01M159716	16.99	https://www.genecards.org/cgi-bin/carddisp.pl?gene=CRP
**TIMP1**	TIMP Metallopeptidase Inhibitor 1	Protein Coding	45	GC0XP047583	16.83	https://www.genecards.org/cgi-bin/carddisp.pl?gene=TIMP1
**DDR2**	Discoidin Domain Receptor Tyrosine Kinase 2	Protein Coding	51	GC01P162631	16.72	https://www.genecards.org/cgi-bin/carddisp.pl?gene=DDR2
**IL1A**	Interleukin 1 Alpha	Protein Coding	44	GC02M112773	16.66	https://www.genecards.org/cgi-bin/carddisp.pl?gene=IL1A
**MMP9**	Matrix Metallopeptidase 9	Protein Coding	52	GC20P046008	16.5	https://www.genecards.org/cgi-bin/carddisp.pl?gene=MMP9
**MATN1**	Matrilin 1	Protein Coding	39	GC01M030711	16.37	https://www.genecards.org/cgi-bin/carddisp.pl?gene=MATN1

**MGP**	Matrix Gla Protein	Protein Coding	42	GC12M014881	16.16	https://www.genecards.org/cgi-bin/carddisp.pl?gene=MGP
**BMP5**	Bone Morphogenetic Protein 5	Protein Coding	41	GC06M055728	16.09	https://www.genecards.org/cgi-bin/carddisp.pl?gene=BMP5
**PTGS1**	Prostaglandin-Endoperoxide Synthase 1	Protein Coding	46	GC09P122370	15.95	https://www.genecards.org/cgi-bin/carddisp.pl?gene=PTGS1
**IGF1**	Insulin Like Growth Factor 1	Protein Coding	50	GC12M102395	15.84	https://www.genecards.org/cgi-bin/carddisp.pl?gene=IGF1
**RUNX2**	RUNX Family Transcription Factor 2	Protein Coding	47	GC06P047549	15.69	https://www.genecards.org/cgi-bin/carddisp.pl?gene=RUNX2
**IL1R1**	Interleukin 1 Receptor Type 1	Protein Coding	45	GC02P102136	15.63	https://www.genecards.org/cgi-bin/carddisp.pl?gene=IL1R1
**SOX9**	SRY-Box Transcription Factor 9	Protein Coding	47	GC17P072121	15.49	https://www.genecards.org/cgi-bin/carddisp.pl?gene=SOX9
**LRCH1**	Leucine Rich Repeats And Calponin Homology Domain Containing 1	Protein Coding	36	GC13P046553	15.44	https://www.genecards.org/cgi-bin/carddisp.pl?gene=LRCH1

**MCF2L**	MCF.2 Cell Line Derived Transforming Sequence Like	Protein Coding	42	GC13P112894	15.25	https://www.genecards.org/cgi-bin/carddisp.pl?gene=MCF2L
**CHI3L1**	Chitinase 3 Like 1	Protein Coding	43	GC01M203148	15.22	https://www.genecards.org/cgi-bin/carddisp.pl?gene=CHI3L1
**UCMA**	Upper Zone Of Growth Plate And Cartilage Matrix Associated	Protein Coding	32	GC10M013222	15.18	https://www.genecards.org/cgi-bin/carddisp.pl?gene=UCMA
**TNFAIP6**	TNF Alpha Induced Protein 6	Protein Coding	40	GC02P151357	15.06	https://www.genecards.org/cgi-bin/carddisp.pl?gene=TNFAIP6
**ADAM10**	ADAM Metallopeptidase Domain 10	Protein Coding	52	GC15M058588	15.04	https://www.genecards.org/cgi-bin/carddisp.pl?gene=ADAM10
**DCN**	Decorin	Protein Coding	47	GC12M091140	15.02	https://www.genecards.org/cgi-bin/carddisp.pl?gene=DCN
**COL3A1**	Collagen Type III Alpha 1 Chain	Protein Coding	47	GC02P188974	15.01	https://www.genecards.org/cgi-bin/carddisp.pl?gene=COL3A1
**COL5A1**	Collagen Type V Alpha 1 Chain	Protein Coding	45	GC09P134641	14.74	https://www.genecards.org/cgi-bin/carddisp.pl?gene=COL5A1

**BMP2**	Bone Morphogenetic Protein 2	Protein Coding	47	GC20P006696	14.72	https://www.genecards.org/cgi-bin/carddisp.pl?gene=BMP2
**COL10A1**	Collagen Type X Alpha 1 Chain	Protein Coding	43	GC06M116118	14.43	https://www.genecards.org/cgi-bin/carddisp.pl?gene=COL10A1
**ADAMTS14**	ADAM Metallopeptidase With Thrombospondin Type 1 Motif 14	Protein Coding	37	GC10P070672	14.37	https://www.genecards.org/cgi-bin/carddisp.pl?gene=ADAMTS14
**HAPLN1**	Hyaluronan And Proteoglycan Link Protein 1	Protein Coding	42	GC05M083637	14.24	https://www.genecards.org/cgi-bin/carddisp.pl?gene=HAPLN1
**CCN6**	Cellular Communication Network Factor 6	Protein Coding	33	GC06P112053	14.2	https://www.genecards.org/cgi-bin/carddisp.pl?gene=CCN6
**MEFV**	MEFV Innate Immuity Regulator, Pyrin	Protein Coding	43	GC16M003281	14	https://www.genecards.org/cgi-bin/carddisp.pl?gene=MEFV
**ALB**	Albumin	Protein Coding	50	GC04P073397	13.92	https://www.genecards.org/cgi-bin/carddisp.pl?gene=ALB
**CLCN7**	Chloride Voltage-Gated Channel 7	Protein Coding	45	GC16M001444	13.83	https://www.genecards.org/cgi-bin/carddisp.pl?gene=CLCN7

**PRG4**	Proteoglycan 4	Protein Coding	39	GC01P186296	13.48	https://www.genecards.org/cgi-bin/carddisp.pl?gene=PRG4
**AEBP1**	AE Binding Protein 1	Protein Coding	39	GC07P044106	13.2	https://www.genecards.org/cgi-bin/carddisp.pl?gene=AEBP1
**ANKH**	ANKH Inorganic Pyrophosphate Transport Regulator	Protein Coding	40	GC05M014706	13.19	https://www.genecards.org/cgi-bin/carddisp.pl?gene=ANKH
**CCAL1**	Chondrocalcinosis 1 (Calcium Pyrophosphate-Deposition Disease, Early Onset Osteoarthritis)	Genetic Locus	3	GC08U990009	13.18	https://www.genecards.org/cgi-bin/carddisp.pl?gene=CCAL1
**HLA-B**	Major Histocompatibility Complex, Class I, B	Protein Coding	45	GC06M031315	13.17	https://www.genecards.org/cgi-bin/carddisp.pl?gene=HLA-B
**COL1A2**	Collagen Type I Alpha 2 Chain	Protein Coding	47	GC07P094394	13.01	https://www.genecards.org/cgi-bin/carddisp.pl?gene=COL1A2
**KIF22**	Kinesin Family Member 22	Protein Coding	43	GC16P029802	12.8	https://www.genecards.org/cgi-bin/carddisp.pl?gene=KIF22
**H19**	H19 Imprinted Maternally Expressed Transcript	RNA Gene	28	GC11M001995	12.31	https://www.genecards.org/cgi-bin/carddisp.pl?gene=H19

**SMAD2**	SMAD Family Member 2	Protein Coding	47	GC18M047809	12.27	https://www.genecards.org/cgi-bin/carddisp.pl?gene=SMAD2
**PHEX**	Phosphate Regulating Endopeptidase Homolog X-Linked	Protein Coding	40	GC0XP022032	12.21	https://www.genecards.org/cgi-bin/carddisp.pl?gene=PHEX
**HOTAIR**	HOX Transcript Antisense RNA	RNA Gene	25	GC12M053962	12.08	https://www.genecards.org/cgi-bin/carddisp.pl?gene=HOTAIR
**CREB3L1**	CAMP Responsive Element Binding Protein 3 Like 1	Protein Coding	41	GC11P046299	12.03	https://www.genecards.org/cgi-bin/carddisp.pl?gene=CREB3L1
**MEG3**	Maternally Expressed 3	RNA Gene	29	GC14P104771	11.9	https://www.genecards.org/cgi-bin/carddisp.pl?gene=MEG3
**TMSB4X**	Thymosin Beta 4 X-Linked	Protein Coding	40	GC0XP012975	11.77	https://www.genecards.org/cgi-bin/carddisp.pl?gene=TMSB4X
**EFEMP2**	EGF Containing Fibulin Extracellular Matrix Protein 2	Protein Coding	43	GC11M065867	11.63	https://www.genecards.org/cgi-bin/carddisp.pl?gene=EFEMP2
**KCNK15-AS1**	KCNK15 And WISP2 Antisense RNA 1	RNA Gene	13	GC20M044694	11.6	https://www.genecards.org/cgi-bin/carddisp.pl?gene=KCNK15-AS1

**TLR4**	Toll Like Receptor 4	Protein Coding	51	GC09P117704	11.49	https://www.genecards.org/cgi-bin/carddisp.pl?gene=TLR4
**GAS5**	Growth Arrest Specific 5	RNA Gene	23	GC01M173947	11.46	https://www.genecards.org/cgi-bin/carddisp.pl?gene=GAS5
**PCGEM1**	PCGEM1 Prostate-Specific Transcript	RNA Gene	18	GC02P192749	11.34	https://www.genecards.org/cgi-bin/carddisp.pl?gene=PCGEM1
**UFC1**	Ubiquitin-Fold Modifier Conjugating Enzyme 1	Protein Coding	37	GC01P161142	11.17	https://www.genecards.org/cgi-bin/carddisp.pl?gene=UFC1
**EXT1**	Exostosin Glycosyltransferase 1	Protein Coding	48	GC08M117798	11.16	https://www.genecards.org/cgi-bin/carddisp.pl?gene=EXT1
**FGFR3**	Fibroblast Growth Factor Receptor 3	Protein Coding	55	GC04P001795	11.14	https://www.genecards.org/cgi-bin/carddisp.pl?gene=FGFR3
**PACERR**	PTGS2 Antisense NFKB1 Complex-Mediated Expression Regulator RNA	RNA Gene	12	GC01P186680	11.04	https://www.genecards.org/cgi-bin/carddisp.pl?gene=PACERR
**HGD**	Homogentisate 1,2-Dioxygenase	Protein Coding	44	GC03M120628	10.95	https://www.genecards.org/cgi-bin/carddisp.pl?gene=HGD

**MYLK**	Myosin Light Chain Kinase	Protein Coding	52	GC03M123610	10.7	https://www.genecards.org/cgi-bin/carddisp.pl?gene=MYLK
**ACTA2**	Actin Alpha 2, Smooth Muscle	Protein Coding	48	GC10M088935	10.7	https://www.genecards.org/cgi-bin/carddisp.pl?gene=ACTA2
**MYH11**	Myosin Heavy Chain 11	Protein Coding	45	GC16M015704	10.7	https://www.genecards.org/cgi-bin/carddisp.pl?gene=MYH11
**SMAD4**	SMAD Family Member 4	Protein Coding	50	GC18P051028	10.69	https://www.genecards.org/cgi-bin/carddisp.pl?gene=SMAD4
**SMAD6**	SMAD Family Member 6	Protein Coding	46	GC15P066702	10.69	https://www.genecards.org/cgi-bin/carddisp.pl?gene=SMAD6
**ACP5**	Acid Phosphatase 5, Tartrate Resistant	Protein Coding	47	GC19M011574	10.5	https://www.genecards.org/cgi-bin/carddisp.pl?gene=ACP5
**SLC2A10**	Solute Carrier Family 2 Member 10	Protein Coding	44	GC20P046709	10.42	https://www.genecards.org/cgi-bin/carddisp.pl?gene=SLC2A10
**PMS2P2**	PMS1 Homolog 2, Mismatch Repair System Component Pseudogene 2	Pseudogene	18	GC07M075343	10.25	https://www.genecards.org/cgi-bin/carddisp.pl?gene=PMS2P2

**F9**	Coagulation Factor IX	Protein Coding	46	GC0XP139530	10.25	https://www.genecards.org/cgi-bin/carddisp.pl?gene=F9
**XYLT1**	Xylosyltransferase 1	Protein Coding	43	GC16M017101	10.15	https://www.genecards.org/cgi-bin/carddisp.pl?gene=XYLT1
**KCNJ5**	Potassium Inwardly Rectifying Channel Subfamily J Member 5	Protein Coding	47	GC11P128891	10.12	https://www.genecards.org/cgi-bin/carddisp.pl?gene=KCNJ5
**KCTD13**	Potassium Channel Tetramerization Domain Containing 13	Protein Coding	35	GC16M029905	10.12	https://www.genecards.org/cgi-bin/carddisp.pl?gene=KCTD13
**SPP1**	Secreted Phosphoprotein 1	Protein Coding	46	GC04P087975	10.1	https://www.genecards.org/cgi-bin/carddisp.pl?gene=SPP1
**IL18**	Interleukin 18	Protein Coding	44	GC11M112143	9.99	https://www.genecards.org/cgi-bin/carddisp.pl?gene=IL18
**FMOD**	Fibromodulin	Protein Coding	40	GC01M203340	9.85	https://www.genecards.org/cgi-bin/carddisp.pl?gene=FMOD
**GNAS**	GNAS Complex Locus	Protein Coding	50	GC20P058839	9.85	https://www.genecards.org/cgi-bin/carddisp.pl?gene=GNAS
**TRPS1**	Transcriptional Repressor GATA Binding 1	Protein Coding	45	GC08M115408	9.85	https://www.genecards.org/cgi-bin/carddisp.pl?gene=TRPS1

**CANT1**	Calcium Activated Nucleotidase 1	Protein Coding	44	GC17M078992	9.85	https://www.genecards.org/cgi-bin/carddisp.pl?gene=CANT1
**CCL2**	C-C Motif Chemokine Ligand 2	Protein Coding	48	GC17P034255	9.84	https://www.genecards.org/cgi-bin/carddisp.pl?gene=CCL2
**TYR**	Tyrosinase	Protein Coding	47	GC11P089177	9.82	https://www.genecards.org/cgi-bin/carddisp.pl?gene=TYR
**DDH2**	Developmental Dysplasia Of The Hip 2	Genetic Locus	2	GC03U902136	9.82	https://www.genecards.org/cgi-bin/carddisp.pl?gene=DDH2
**TP53**	Tumor Protein P53	Protein Coding	54	GC17M007661	9.82	https://www.genecards.org/cgi-bin/carddisp.pl?gene=TP53
**GHR**	Growth Hormone Receptor	Protein Coding	47	GC05P042429	9.69	https://www.genecards.org/cgi-bin/carddisp.pl?gene=GHR
**IL4**	Interleukin 4	Protein Coding	46	GC05P132673	9.41	https://www.genecards.org/cgi-bin/carddisp.pl?gene=IL4
**ADIPOQ**	Adiponectin, C1Q And Collagen Domain Containing	Protein Coding	45	GC03P186842	9.38	https://www.genecards.org/cgi-bin/carddisp.pl?gene=ADIPOQ
**VEGFA**	Vascular Endothelial Growth Factor A	Protein Coding	48	GC06P043770	9.3	https://www.genecards.org/cgi-bin/carddisp.pl?gene=VEGFA

**PTH**	Parathyroid Hormone	Protein Coding	47	GC11M013492	9.29	https://www.genecards.org/cgi-bin/carddisp.pl?gene=PTH
**HFE**	Homeostatic Iron Regulator	Protein Coding	43	GC06P026087	8.83	https://www.genecards.org/cgi-bin/carddisp.pl?gene=HFE
**MMP2**	Matrix Metallopeptidase 2	Protein Coding	53	GC16P055390	8.78	https://www.genecards.org/cgi-bin/carddisp.pl?gene=MMP2
**IFNG**	Interferon Gamma	Protein Coding	48	GC12M068064	8.74	https://www.genecards.org/cgi-bin/carddisp.pl?gene=IFNG
**LEP**	Leptin	Protein Coding	47	GC07P128241	8.69	https://www.genecards.org/cgi-bin/carddisp.pl?gene=LEP
**CD36**	CD36 Molecule	Protein Coding	48	GC07P080369	8.51	https://www.genecards.org/cgi-bin/carddisp.pl?gene=CD36
**VDR**	Vitamin D Receptor	Protein Coding	51	GC12M047841	8.43	https://www.genecards.org/cgi-bin/carddisp.pl?gene=VDR
**GBA**	Glucosylceramidase Beta	Protein Coding	47	GC01M155234	8.29	https://www.genecards.org/cgi-bin/carddisp.pl?gene=GBA
**EXT2**	Exostosin Glycosyltransferase 2	Protein Coding	46	GC11P044095	8.29	https://www.genecards.org/cgi-bin/carddisp.pl?gene=EXT2
**F8**	Coagulation Factor VIII	Protein Coding	45	GC0XM154835	8.29	https://www.genecards.org/cgi-bin/carddisp.pl?gene=F8

**SCARB2**	Scavenger Receptor Class B Member 2	Protein Coding	44	GC04M076158	8.29	https://www.genecards.org/cgi-bin/carddisp.pl?gene=SCARB2
**MIR146A**	MicroRNA 146a	RNA Gene	22	GC05P160485	8.16	https://www.genecards.org/cgi-bin/carddisp.pl?gene=MIR146A
**TNFRSF1A**	TNF Receptor Superfamily Member 1A	Protein Coding	49	GC12M006328	8.15	https://www.genecards.org/cgi-bin/carddisp.pl?gene=TNFRSF1A
**LRP5**	LDL Receptor Related Protein 5	Protein Coding	48	GC11P068298	8.15	https://www.genecards.org/cgi-bin/carddisp.pl?gene=LRP5
**CTSK**	Cathepsin K	Protein Coding	48	GC01M150796	8.14	https://www.genecards.org/cgi-bin/carddisp.pl?gene=CTSK
**CCL5**	C-C Motif Chemokine Ligand 5	Protein Coding	43	GC17M035871	8.14	https://www.genecards.org/cgi-bin/carddisp.pl?gene=CCL5
**PADI4**	Peptidyl Arginine Deiminase 4	Protein Coding	44	GC01P017308	8.1	https://www.genecards.org/cgi-bin/carddisp.pl?gene=PADI4
**TLR2**	Toll Like Receptor 2	Protein Coding	51	GC04P153684	8.08	https://www.genecards.org/cgi-bin/carddisp.pl?gene=TLR2

**CALCA**	Calcitonin Related Polypeptide Alpha	Protein Coding	43	GC11M014945	8.04	https://www.genecards.org/cgi-bin/carddisp.pl?gene=CALCA
**PTPN22**	Protein Tyrosine Phosphatase Non-Receptor Type 22	Protein Coding	46	GC01M113813	7.99	https://www.genecards.org/cgi-bin/carddisp.pl?gene=PTPN22
**IL13**	Interleukin 13	Protein Coding	44	GC05P132656	7.96	https://www.genecards.org/cgi-bin/carddisp.pl?gene=IL13
**CCL3**	C-C Motif Chemokine Ligand 3	Protein Coding	39	GC17M036088	7.91	https://www.genecards.org/cgi-bin/carddisp.pl?gene=CCL3
**PTHLH**	Parathyroid Hormone Like Hormone	Protein Coding	45	GC12M027959	7.84	https://www.genecards.org/cgi-bin/carddisp.pl?gene=PTHLH
**ICAM1**	Intercellular Adhesion Molecule 1	Protein Coding	50	GC19P010270	7.77	https://www.genecards.org/cgi-bin/carddisp.pl?gene=ICAM1
**UBA5**	Ubiquitin Like Modifier Activating Enzyme 5	Protein Coding	43	GC03P132654	7.76	https://www.genecards.org/cgi-bin/carddisp.pl?gene=UBA5
**UFM1**	Ubiquitin Fold Modifier 1	Protein Coding	40	GC13P038349	7.76	https://www.genecards.org/cgi-bin/carddisp.pl?gene=UFM1

**DDRGK1**	DDRGK Domain Containing 1	Protein Coding	37	GC20M003191	7.76	https://www.genecards.org/cgi-bin/carddisp.pl?gene=DDRGK1
**UFL1**	UFM1 Specific Ligase 1	Protein Coding	35	GC06P096521	7.76	https://www.genecards.org/cgi-bin/carddisp.pl?gene=UFL1
**UFSP1**	UFM1 Specific Peptidase 1 (Inactive)	Protein Coding	32	GC07M100888	7.76	https://www.genecards.org/cgi-bin/carddisp.pl?gene=UFSP1
**C4orf47**	Chromosome 4 Open Reading Frame 47	Protein Coding	25	GC04P185426	7.76	https://www.genecards.org/cgi-bin/carddisp.pl?gene=C4orf47
**CSF1**	Colony Stimulating Factor 1	Protein Coding	43	GC01P109911	7.76	https://www.genecards.org/cgi-bin/carddisp.pl?gene=CSF1
**IL15**	Interleukin 15	Protein Coding	40	GC04P141636	7.73	https://www.genecards.org/cgi-bin/carddisp.pl?gene=IL15
**ESR1**	Estrogen Receptor 1	Protein Coding	53	GC06P151656	7.54	https://www.genecards.org/cgi-bin/carddisp.pl?gene=ESR1
**HPGD**	15-Hydroxyprostaglandin Dehydrogenase	Protein Coding	48	GC04M174490	7.51	https://www.genecards.org/cgi-bin/carddisp.pl?gene=HPGD
**C1S**	Complement C1s	Protein Coding	45	GC12P008238	7.46	https://www.genecards.org/cgi-bin/carddisp.pl?gene=C1S

**CXCL12**	C-X-C Motif Chemokine Ligand 12	Protein Coding	45	GC10M044294	7.38	https://www.genecards.org/cgi-bin/carddisp.pl?gene=CXCL12
**MIA2**	MIA SH3 Domain ER Export Factor 2	Protein Coding	37	GC14P039230	7.37	https://www.genecards.org/cgi-bin/carddisp.pl?gene=MIA2
**HMGB1**	High Mobility Group Box 1	Protein Coding	44	GC13M030456	7.36	https://www.genecards.org/cgi-bin/carddisp.pl?gene=HMGB1
**ATP7B**	ATPase Copper Transporting Beta	Protein Coding	47	GC13M051930	7.35	https://www.genecards.org/cgi-bin/carddisp.pl?gene=ATP7B
**SEMA3A**	Semaphorin 3A	Protein Coding	45	GC07M083955	7.31	https://www.genecards.org/cgi-bin/carddisp.pl?gene=SEMA3A
**ENPP1**	Ectonucleotide Pyrophosphatase/Phosphodiesterase 1	Protein Coding	47	GC06P131808	7.29	https://www.genecards.org/cgi-bin/carddisp.pl?gene=ENPP1
**ALPL**	Alkaline Phosphatase, Biomineralization Associated	Protein Coding	50	GC01P021508	7.24	https://www.genecards.org/cgi-bin/carddisp.pl?gene=ALPL
**TNFRSF1B**	TNF Receptor Superfamily Member 1B	Protein Coding	47	GC01P012167	7.22	https://www.genecards.org/cgi-bin/carddisp.pl?gene=TNFRSF1B

**CAT**	Catalase	Protein Coding	50	GC11P034 460	7.19	https://www.genecards.org/cgi-bin/carddisp.pl?gene=CAT
**LMX1B**	LIM Homeobox Transcription Factor 1 Beta	Protein Coding	45	GC09P126 614	7.17	https://www.genecards.org/cgi-bin/carddisp.pl?gene=LMX1B
**ADAMTSL1**	ADAMTS Like 1	Protein Coding	41	GC09P017 906	7.16	https://www.genecards.org/cgi-bin/carddisp.pl?gene=ADAMTSL1
**JUN**	Jun Proto-Oncogene, AP-1 Transcription Factor Subunit	Protein Coding	49	GC01M058 780	7.15	https://www.genecards.org/cgi-bin/carddisp.pl?gene=JUN
**TIMP2**	TIMP Metallopeptidase Inhibitor 2	Protein Coding	44	GC17M078 852	7.12	https://www.genecards.org/cgi-bin/carddisp.pl?gene=TIMP2
**IL2**	Interleukin 2	Protein Coding	45	GC04M122 451	7.08	https://www.genecards.org/cgi-bin/carddisp.pl?gene=IL2
**VCAM1**	Vascular Cell Adhesion Molecule 1	Protein Coding	45	GC01P100 719	7.07	https://www.genecards.org/cgi-bin/carddisp.pl?gene=VCAM1
**ELN**	Elastin	Protein Coding	44	GC07P074 027	7.06	https://www.genecards.org/cgi-bin/carddisp.pl?gene=ELN
**FAS**	Fas Cell Surface Death Receptor	Protein Coding	50	GC10P088 969	7	https://www.genecards.org/cgi-bin/carddisp.pl?gene=FAS
**FN1**	Fibronectin 1	Protein Coding	50	GC02M215 360	6.98	https://www.genecards.org/cgi-bin/carddisp.pl?gene=FN1

**SIRT1**	Sirtuin 1	Protein Coding	49	GC10P067884	6.96	https://www.genecards.org/cgi-bin/carddisp.pl?gene=SIRT1
**GNA11**	G Protein Subunit Alpha 11	Protein Coding	47	GC19P003094	6.94	https://www.genecards.org/cgi-bin/carddisp.pl?gene=GNA11
**CIITA**	Class II Major Histocompatibility Complex Transactivator	Protein Coding	45	GC16P010879	6.94	https://www.genecards.org/cgi-bin/carddisp.pl?gene=CIITA
**AKR1C1**	Aldo-Keto Reductase Family 1 Member C1	Protein Coding	43	GC10P004963	6.94	https://www.genecards.org/cgi-bin/carddisp.pl?gene=AKR1C1
**PLOD1**	Procollagen-Lysine,2-Oxoglutarate 5-Dioxygenase 1	Protein Coding	41	GC01P011934	6.94	https://www.genecards.org/cgi-bin/carddisp.pl?gene=PLOD1
**TECTB**	Tectorin Beta	Protein Coding	31	GC10P112283	6.94	https://www.genecards.org/cgi-bin/carddisp.pl?gene=TECTB
**IL1RAPL2**	Interleukin 1 Receptor Accessory Protein Like 2	Protein Coding	37	GC0XP104566	6.94	https://www.genecards.org/cgi-bin/carddisp.pl?gene=IL1RAPL2
**SERPINA3**	Serpin Family A Member 3	Protein Coding	43	GC14P094612	6.89	https://www.genecards.org/cgi-bin/carddisp.pl?gene=SERPINA3

**CCND1**	Cyclin D1	Protein Coding	52	GC11P069641	6.89	https://www.genecards.org/cgi-bin/carddisp.pl?gene=CCND1
**MIR155**	MicroRNA 155	RNA Gene	18	GC21P025573	6.86	https://www.genecards.org/cgi-bin/carddisp.pl?gene=MIR155
**MMP8**	Matrix Metallopeptidase 8	Protein Coding	47	GC11M102617	6.85	https://www.genecards.org/cgi-bin/carddisp.pl?gene=MMP8
**NOS2**	Nitric Oxide Synthase 2	Protein Coding	49	GC17M027756	6.83	https://www.genecards.org/cgi-bin/carddisp.pl?gene=NOS2
**SAA1**	Serum Amyloid A1	Protein Coding	42	GC11P018267	6.83	https://www.genecards.org/cgi-bin/carddisp.pl?gene=SAA1
**FGF2**	Fibroblast Growth Factor 2	Protein Coding	47	GC04P122826	6.75	https://www.genecards.org/cgi-bin/carddisp.pl?gene=FGF2
**B2M**	Beta-2-Microglobulin	Protein Coding	48	GC15P044711	6.75	https://www.genecards.org/cgi-bin/carddisp.pl?gene=B2M
**CCR6**	C-C Motif Chemokine Receptor 6	Protein Coding	44	GC06P167111	6.75	https://www.genecards.org/cgi-bin/carddisp.pl?gene=CCR6

**LMNA**	Lamin A/C	Protein Coding	47	GC01P156082	6.65	https://www.genecards.org/cgi-bin/carddisp.pl?gene=LMNA
**TRAPPC2B**	Trafficking Protein Particle Complex 2B	Protein Coding	18	GC19P057364	6.63	https://www.genecards.org/cgi-bin/carddisp.pl?gene=TRAPPC2B
**CASR**	Calcium Sensing Receptor	Protein Coding	50	GC03P122183	6.55	https://www.genecards.org/cgi-bin/carddisp.pl?gene=CASR
**TNFRSF11A**	TNF Receptor Superfamily Member 11a	Protein Coding	45	GC18P062325	6.55	https://www.genecards.org/cgi-bin/carddisp.pl?gene=TNFRSF11A
**CD44**	CD44 Molecule (Indian Blood Group)	Protein Coding	47	GC11P035139	6.52	https://www.genecards.org/cgi-bin/carddisp.pl?gene=CD44
**THBS1**	Thrombospondin 1	Protein Coding	44	GC15P039581	6.42	https://www.genecards.org/cgi-bin/carddisp.pl?gene=THBS1
**DKK 1.00**	Dickkopf WNT Signaling Pathway Inhibitor 1	Protein Coding	45	GC10P052314	6.42	https://www.genecards.org/cgi-bin/carddisp.pl?gene=DKK1
**IGFBP3**	Insulin Like Growth Factor Binding Protein 3	Protein Coding	45	GC07M045912	6.41	https://www.genecards.org/cgi-bin/carddisp.pl?gene=IGFBP3

**MIR15A**	MicroRNA 15a	RNA Gene	16	GC13M050049	6.39	https://www.genecards.org/cgi-bin/carddisp.pl?gene=MIR15A
**PPARG**	Peroxisome Proliferator Activated Receptor Gamma	Protein Coding	52	GC03P012287	6.39	https://www.genecards.org/cgi-bin/carddisp.pl?gene=PPARG
**AIP**	Aryl Hydrocarbon Receptor Interacting Protein	Protein Coding	44	GC11P067468	6.38	https://www.genecards.org/cgi-bin/carddisp.pl?gene=AIP
**OSM**	Oncostatin M	Protein Coding	43	GC22M030262	6.33	https://www.genecards.org/cgi-bin/carddisp.pl?gene=OSM
**MAPK8**	Mitogen-Activated Protein Kinase 8	Protein Coding	50	GC10P048306	6.31	https://www.genecards.org/cgi-bin/carddisp.pl?gene=MAPK8
**SOST**	Sclerostin	Protein Coding	44	GC17M043753	6.28	https://www.genecards.org/cgi-bin/carddisp.pl?gene=SOST
**ACE**	Angiotensin I Converting Enzyme	Protein Coding	49	GC17P063477	6.28	https://www.genecards.org/cgi-bin/carddisp.pl?gene=ACE
**HSPA5**	Heat Shock Protein Family A (Hsp70) Member 5	Protein Coding	47	GC09M125234	6.27	https://www.genecards.org/cgi-bin/carddisp.pl?gene=HSPA5
**SLC40A1**	Solute Carrier Family 40 Member 1	Protein Coding	46	GC02M189560	6.25	https://www.genecards.org/cgi-bin/carddisp.pl?gene=SLC40A1

**ZMPSTE24**	Zinc Metallopeptidase STE24	Protein Coding	43	GC01P040258	6.25	https://www.genecards.org/cgi-bin/carddisp.pl?gene=ZMPSTE24
**GPR101**	G Protein-Coupled Receptor 101	Protein Coding	39	GC0XM137030	6.25	https://www.genecards.org/cgi-bin/carddisp.pl?gene=GPR101
**KIF7**	Kinesin Family Member 7	Protein Coding	39	GC15M089608	6.25	https://www.genecards.org/cgi-bin/carddisp.pl?gene=KIF7
**ZNF687**	Zinc Finger Protein 687	Protein Coding	37	GC01P151281	6.25	https://www.genecards.org/cgi-bin/carddisp.pl?gene=ZNF687
**RETN**	Resistin	Protein Coding	43	GC19P007669	6.24	https://www.genecards.org/cgi-bin/carddisp.pl?gene=RETN
**HLA-DRB1**	Major Histocompatibility Complex, Class II, DR Beta 1	Protein Coding	46	GC06M032578	6.24	https://www.genecards.org/cgi-bin/carddisp.pl?gene=HLA-DRB1
**IL7**	Interleukin 7	Protein Coding	42	GC08M078689	6.21	https://www.genecards.org/cgi-bin/carddisp.pl?gene=IL7
**ADAMTS3**	ADAM Metallopeptidase With Thrombospondin Type 1 Motif 3	Protein Coding	41	GC04M072280	6.2	https://www.genecards.org/cgi-bin/carddisp.pl?gene=ADAMTS3
**IBSP**	Integrin Binding Sialoprotein	Protein Coding	37	GC04P087799	6.2	https://www.genecards.org/cgi-bin/carddisp.pl?gene=IBSP

**POMC**	Proopiomelanocortin	Protein Coding	48	GC02M025160	6.15	https://www.genecards.org/cgi-bin/carddisp.pl?gene=POMC
**CD40LG**	CD40 Ligand	Protein Coding	47	GC0XP136649	6.15	https://www.genecards.org/cgi-bin/carddisp.pl?gene=CD40LG
**RELN**	Reelin	Protein Coding	43	GC07M103471	6.12	https://www.genecards.org/cgi-bin/carddisp.pl?gene=RELN
**MIR181A2**	MicroRNA 181a-2	RNA Gene	20	GC09P124692	6.12	https://www.genecards.org/cgi-bin/carddisp.pl?gene=MIR181A2
**CXCL1**	C-X-C Motif Chemokine Ligand 1	Protein Coding	43	GC04P073869	6.12	https://www.genecards.org/cgi-bin/carddisp.pl?gene=CXCL1
**GALNS**	Galactosamine (N-Acetyl)-6-Sulfatase	Protein Coding	44	GC16M088813	6.1	https://www.genecards.org/cgi-bin/carddisp.pl?gene=GALNS
**IL11**	Interleukin 11	Protein Coding	41	GC19M055364	6.07	https://www.genecards.org/cgi-bin/carddisp.pl?gene=IL11
**SMAP2**	Small ArfGAP2	Protein Coding	37	GC01P040344	6.01	https://www.genecards.org/cgi-bin/carddisp.pl?gene=SMAP2

**MAPK1**	Mitogen-Activated Protein Kinase 1	Protein Coding	51	GC22M021754	5.98	https://www.genecards.org/cgi-bin/carddisp.pl?gene=MAPK1
**MIF**	Macrophage Migration Inhibitory Factor	Protein Coding	49	GC22P023894	5.97	https://www.genecards.org/cgi-bin/carddisp.pl?gene=MIF
**FOXP3**	Forkhead Box P3	Protein Coding	46	GC0XM049250	5.96	https://www.genecards.org/cgi-bin/carddisp.pl?gene=FOXP3
**EFEMP1**	EGF Containing Fibulin Extracellular Matrix Protein 1	Protein Coding	44	GC02M055865	5.96	https://www.genecards.org/cgi-bin/carddisp.pl?gene=EFEMP1
**CXCR4**	C-X-C Motif Chemokine Receptor 4	Protein Coding	52	GC02M136114	5.92	https://www.genecards.org/cgi-bin/carddisp.pl?gene=CXCR4
**HLA-DQB1**	Major Histocompatibility Complex, Class II, DQ Beta 1	Protein Coding	44	GC06M032804	5.91	https://www.genecards.org/cgi-bin/carddisp.pl?gene=HLA-DQB1
**KL**	Klotho	Protein Coding	44	GC13P033016	5.9	https://www.genecards.org/cgi-bin/carddisp.pl?gene=KL
**P4HB**	Prolyl 4-Hydroxylase Subunit Beta	Protein Coding	49	GC17M081843	5.89	https://www.genecards.org/cgi-bin/carddisp.pl?gene=P4HB
**HIF1A**	Hypoxia Inducible Factor 1 Subunit Alpha	Protein Coding	47	GC14P061695	5.89	https://www.genecards.org/cgi-bin/carddisp.pl?gene=HIF1A

**FASLG**	Fas Ligand	Protein Coding	47	GC01P172628	5.85	https://www.genecards.org/cgi-bin/carddisp.pl?gene=FASLG
**MEN1**	Menin 1	Protein Coding	46	GC11M064803	5.82	https://www.genecards.org/cgi-bin/carddisp.pl?gene=MEN1
**AP3D1**	Adaptor Related Protein Complex 3 Subunit Delta 1	Protein Coding	40	GC19M002101	5.82	https://www.genecards.org/cgi-bin/carddisp.pl?gene=AP3D1
**S100A9**	S100 Calcium Binding Protein A9	Protein Coding	43	GC01P153357	5.82	https://www.genecards.org/cgi-bin/carddisp.pl?gene=S100A9
**S100A12**	S100 Calcium Binding Protein A12	Protein Coding	39	GC01M153373	5.81	https://www.genecards.org/cgi-bin/carddisp.pl?gene=S100A12
**OPRM1**	Opioid Receptor Mu 1	Protein Coding	48	GC06P154075	5.8	https://www.genecards.org/cgi-bin/carddisp.pl?gene=OPRM1
**BMP7**	Bone Morphogenetic Protein 7	Protein Coding	45	GC20M057168	5.77	https://www.genecards.org/cgi-bin/carddisp.pl?gene=BMP7
**BMP6**	Bone Morphogenetic Protein 6	Protein Coding	43	GC06P007726	5.72	https://www.genecards.org/cgi-bin/carddisp.pl?gene=BMP6

**CRH**	Corticotropin Releasing Hormone	Protein Coding	44	GC08M066 176	5.7	https://www.genecards.org/cgi-bin/carddisp.pl?gene=CRH
**IL18R1**	Interleukin 18 Receptor 1	Protein Coding	43	GC02P102 311	5.69	https://www.genecards.org/cgi-bin/carddisp.pl?gene=IL18R1
**ADAM17**	ADAM Metallopeptidase Domain 17	Protein Coding	51	GC02M009 488	5.68	https://www.genecards.org/cgi-bin/carddisp.pl?gene=ADAM17
**OPTC**	Opticin	Protein Coding	39	GC01P203 464	5.66	https://www.genecards.org/cgi-bin/carddisp.pl?gene=OPTC
**MIR140**	MicroRNA 140	RNA Gene	22	GC16P069 934	5.65	https://www.genecards.org/cgi-bin/carddisp.pl?gene=MIR140
**NFKBIA**	NFKB Inhibitor Alpha	Protein Coding	50	GC14M035 401	5.65	https://www.genecards.org/cgi-bin/carddisp.pl?gene=NFKBIA
**MPO**	Myeloperoxidase	Protein Coding	50	GC17M058 269	5.63	https://www.genecards.org/cgi-bin/carddisp.pl?gene=MPO
**EGF**	Epidermal Growth Factor	Protein Coding	51	GC04P109 912	5.63	https://www.genecards.org/cgi-bin/carddisp.pl?gene=EGF
**CCL4**	C-C Motif Chemokine Ligand 4	Protein Coding	40	GC17P036 103	5.62	https://www.genecards.org/cgi-bin/carddisp.pl?gene=CCL4

**CSF2**	Colony Stimulating Factor 2	Protein Coding	44	GC05P132073	5.6	https://www.genecards.org/cgi-bin/carddisp.pl?gene=CSF2
**IHH**	Indian Hedgehog Signaling Molecule	Protein Coding	47	GC02M219054	5.57	https://www.genecards.org/cgi-bin/carddisp.pl?gene=IHH
**FOS**	Fos Proto-Oncogene, AP-1 Transcription Factor Subunit	Protein Coding	50	GC14P075278	5.56	https://www.genecards.org/cgi-bin/carddisp.pl?gene=FOS
**SOD1**	Superoxide Dismutase 1	Protein Coding	51	GC21P031659	5.55	https://www.genecards.org/cgi-bin/carddisp.pl?gene=SOD1
**IL23A**	Interleukin 23 Subunit Alpha	Protein Coding	39	GC12P056335	5.54	https://www.genecards.org/cgi-bin/carddisp.pl?gene=IL23A
**BCL2**	BCL2 Apoptosis Regulator	Protein Coding	51	GC18M063123	5.53	https://www.genecards.org/cgi-bin/carddisp.pl?gene=BCL2
**FGF23**	Fibroblast Growth Factor 23	Protein Coding	45	GC12M004368	5.52	https://www.genecards.org/cgi-bin/carddisp.pl?gene=FGF23
**FBLN2**	Fibulin 2	Protein Coding	43	GC03P013565	5.49	https://www.genecards.org/cgi-bin/carddisp.pl?gene=FBLN2
**CD86**	CD86 Molecule	Protein Coding	43	GC03P122055	5.49	https://www.genecards.org/cgi-bin/carddisp.pl?gene=CD86

**THBS3**	Thrombospondin 3	Protein Coding	41	GC01M155195	5.49	https://www.genecards.org/cgi-bin/carddisp.pl?gene=THBS3
**INA**	Internexin Neuronal Intermediate Filament Protein Alpha	Protein Coding	39	GC10P103277	5.49	https://www.genecards.org/cgi-bin/carddisp.pl?gene=INA
**TRAPPC3**	Trafficking Protein Particle Complex 3	Protein Coding	39	GC01M036136	5.49	https://www.genecards.org/cgi-bin/carddisp.pl?gene=TRAPPC3
**TRAPPC4**	Trafficking Protein Particle Complex 4	Protein Coding	39	GC11P119018	5.49	https://www.genecards.org/cgi-bin/carddisp.pl?gene=TRAPPC4
**TRAPPC9**	Trafficking Protein Particle Complex 9	Protein Coding	39	GC08M139728	5.49	https://www.genecards.org/cgi-bin/carddisp.pl?gene=TRAPPC9
**TRAPPC6B**	Trafficking Protein Particle Complex 6B	Protein Coding	39	GC14M039147	5.49	https://www.genecards.org/cgi-bin/carddisp.pl?gene=TRAPPC6B
**CREB3L3**	CAMP Responsive Element Binding Protein 3 Like 3	Protein Coding	37	GC19P004153	5.49	https://www.genecards.org/cgi-bin/carddisp.pl?gene=CREB3L3
**TRAPPC1**	Trafficking Protein Particle Complex 1	Protein Coding	37	GC17M007930	5.49	https://www.genecards.org/cgi-bin/carddisp.pl?gene=TRAPPC1

**TRAPPC10**	Trafficking Protein Particle Complex 10	Protein Coding	37	GC21P044012	5.49	https://www.genecards.org/cgi-bin/carddisp.pl?gene=TRAPPC10
**TRAPPC2L**	Trafficking Protein Particle Complex 2 Like	Protein Coding	37	GC16P088856	5.49	https://www.genecards.org/cgi-bin/carddisp.pl?gene=TRAPPC2L
**TRAPPC6A**	Trafficking Protein Particle Complex 6A	Protein Coding	37	GC19M045162	5.49	https://www.genecards.org/cgi-bin/carddisp.pl?gene=TRAPPC6A
**TMED2**	Transmembrane P24 Trafficking Protein 2	Protein Coding	36	GC12P123584	5.49	https://www.genecards.org/cgi-bin/carddisp.pl?gene=TMED2
**TRAPPC5**	Trafficking Protein Particle Complex 5	Protein Coding	35	GC19P007680	5.49	https://www.genecards.org/cgi-bin/carddisp.pl?gene=TRAPPC5
**TRAPPC8**	Trafficking Protein Particle Complex 8	Protein Coding	34	GC18M031829	5.49	https://www.genecards.org/cgi-bin/carddisp.pl?gene=TRAPPC8
**GPR33**	G Protein-Coupled Receptor 33	Protein Coding	25	GC14M031482	5.49	https://www.genecards.org/cgi-bin/carddisp.pl?gene=GPR33
**TRAPPC3L**	Trafficking Protein Particle Complex 3 Like	Protein Coding	25	GC06M116494	5.49	https://www.genecards.org/cgi-bin/carddisp.pl?gene=TRAPPC3L

**TLR9**	Toll Like Receptor 9	Protein Coding	45	GC03M052222	5.49	https://www.genecards.org/cgi-bin/carddisp.pl?gene=TLR9
**MIR181A1**	MicroRNA 181a-1	RNA Gene	18	GC01M198860	5.49	https://www.genecards.org/cgi-bin/carddisp.pl?gene=MIR181A1
**TTR**	Transthyretin	Protein Coding	49	GC18P031557	5.46	https://www.genecards.org/cgi-bin/carddisp.pl?gene=TTR
**TLR5**	Toll Like Receptor 5	Protein Coding	47	GC01M223109	5.42	https://www.genecards.org/cgi-bin/carddisp.pl?gene=TLR5
**CYP27B1**	Cytochrome P450 Family 27 Subfamily B Member 1	Protein Coding	47	GC12M057757	5.42	https://www.genecards.org/cgi-bin/carddisp.pl?gene=CYP27B1
**IL6R**	Interleukin 6 Receptor	Protein Coding	48	GC01P154405	5.42	https://www.genecards.org/cgi-bin/carddisp.pl?gene=IL6R
**CTNNB1**	Catenin Beta 1	Protein Coding	53	GC03P041236	5.4	https://www.genecards.org/cgi-bin/carddisp.pl?gene=CTNNB1
**CASP3**	Caspase 3	Protein Coding	50	GC04M184627	5.39	https://www.genecards.org/cgi-bin/carddisp.pl?gene=CASP3
**C1R**	Complement C1r	Protein Coding	46	GC12M007135	5.37	https://www.genecards.org/cgi-bin/carddisp.pl?gene=C1R

**HSP90B1**	Heat Shock Protein 90 Beta Family Member 1	Protein Coding	45	GC12P103930	5.37	https://www.genecards.org/cgi-bin/carddisp.pl?gene=HSP90B1
**PTPN11**	Protein Tyrosine Phosphatase Non-Receptor Type 11	Protein Coding	53	GC12P112418	5.31	https://www.genecards.org/cgi-bin/carddisp.pl?gene=PTPN11
**NOD2**	Nucleotide Binding Oligomerization Domain Containing 2	Protein Coding	48	GC16P050693	5.31	https://www.genecards.org/cgi-bin/carddisp.pl?gene=NOD2
**CANX**	Calnexin	Protein Coding	44	GC05P179678	5.31	https://www.genecards.org/cgi-bin/carddisp.pl?gene=CANX
**MEPE**	Matrix Extracellular Phosphoglycoprotein	Protein Coding	35	GC04P087821	5.31	https://www.genecards.org/cgi-bin/carddisp.pl?gene=MEPE
**MIR150**	MicroRNA 150	RNA Gene	21	GC19M049500	5.31	https://www.genecards.org/cgi-bin/carddisp.pl?gene=MIR150
**MIR19A**	MicroRNA 19a	RNA Gene	19	GC13P091433	5.31	https://www.genecards.org/cgi-bin/carddisp.pl?gene=MIR19A
**FOXM1**	Forkhead Box M1	Protein Coding	44	GC12M002857	5.3	https://www.genecards.org/cgi-bin/carddisp.pl?gene=FOXM1

**NLRP1**	NLR Family Pyrin Domain Containing 1	Protein Coding	44	GC17M005499	5.3	https://www.genecards.org/cgi-bin/carddisp.pl?gene=NLRP1
**DSPP**	Dentin Sialophosphoprotein	Protein Coding	37	GC04P087608	5.3	https://www.genecards.org/cgi-bin/carddisp.pl?gene=DSPP
**GAPDH**	Glyceraldehyde-3-Phosphate Dehydrogenase	Protein Coding	48	GC12P008161	5.29	https://www.genecards.org/cgi-bin/carddisp.pl?gene=GAPDH
**DMP1**	Dentin Matrix Acidic Phosphoprotein 1	Protein Coding	42	GC04P087650	5.28	https://www.genecards.org/cgi-bin/carddisp.pl?gene=DMP1
**FSTL1**	Follistatin Like 1	Protein Coding	41	GC03M120392	5.27	https://www.genecards.org/cgi-bin/carddisp.pl?gene=FSTL1
**COG5**	Component Of Oligomeric Golgi Complex 5	Protein Coding	39	GC07M107201	5.25	https://www.genecards.org/cgi-bin/carddisp.pl?gene=COG5
**IGF2**	Insulin Like Growth Factor 2	Protein Coding	48	GC11M002130	5.25	https://www.genecards.org/cgi-bin/carddisp.pl?gene=IGF2
**LTA**	Lymphotoxin Alpha	Protein Coding	42	GC06P047303	5.25	https://www.genecards.org/cgi-bin/carddisp.pl?gene=LTA

**CTSB**	Cathepsin B	Protein Coding	51	GC08M011842	5.23	https://www.genecards.org/cgi-bin/carddisp.pl?gene=CTSB
**BTNL2**	Butyrophilin Like 2	Protein Coding	39	GC06M032393	5.18	https://www.genecards.org/cgi-bin/carddisp.pl?gene=BTNL2
**CCL20**	C-C Motif Chemokine Ligand 20	Protein Coding	43	GC02P227813	5.18	https://www.genecards.org/cgi-bin/carddisp.pl?gene=CCL20
**SOD2**	Superoxide Dismutase 2	Protein Coding	51	GC06M159669	5.16	https://www.genecards.org/cgi-bin/carddisp.pl?gene=SOD2
**HTRA1**	HtrA Serine Peptidase 1	Protein Coding	43	GC10P122461	5.15	https://www.genecards.org/cgi-bin/carddisp.pl?gene=HTRA1
**PTGES**	Prostaglandin E Synthase	Protein Coding	41	GC09M129738	5.14	https://www.genecards.org/cgi-bin/carddisp.pl?gene=PTGES
**FLT1**	Fms Related Receptor Tyrosine Kinase 1	Protein Coding	51	GC13M028300	5.12	https://www.genecards.org/cgi-bin/carddisp.pl?gene=FLT1
**PTH1R**	Parathyroid Hormone 1 Receptor	Protein Coding	50	GC03P046877	5.12	https://www.genecards.org/cgi-bin/carddisp.pl?gene=PTH1R

**PEPD**	Peptidase D	Protein Coding	45	GC19M033386	5.12	https://www.genecards.org/cgi-bin/carddisp.pl?gene=PEPD
**TNFSF13B**	TNF Superfamily Member 13b	Protein Coding	45	GC13P108251	5.09	https://www.genecards.org/cgi-bin/carddisp.pl?gene=TNFSF13B
**RELA**	RELA Proto-Oncogene, NF-KB Subunit	Protein Coding	50	GC11M065653	5.09	https://www.genecards.org/cgi-bin/carddisp.pl?gene=RELA
**GREM1**	Gremlin 1, DAN Family BMP Antagonist	Protein Coding	44	GC15P032720	5.06	https://www.genecards.org/cgi-bin/carddisp.pl?gene=GREM1
**PLAUR**	Plasminogen Activator, Urokinase Receptor	Protein Coding	44	GC19M043646	5.06	https://www.genecards.org/cgi-bin/carddisp.pl?gene=PLAUR
**STAT3**	Signal Transducer And Activator Of Transcription 3	Protein Coding	52	GC17M042313	5.05	https://www.genecards.org/cgi-bin/carddisp.pl?gene=STAT3
**SLC22A4**	Solute Carrier Family 22 Member 4	Protein Coding	43	GC05P132294	5.04	https://www.genecards.org/cgi-bin/carddisp.pl?gene=SLC22A4
**TNXB**	Tenascin XB	Protein Coding	43	GC06M032635	5.04	https://www.genecards.org/cgi-bin/carddisp.pl?gene=TNXB

**MATN4**	Matrilin 4	Protein Coding	36	GC20M045293	5.04	https://www.genecards.org/cgi-bin/carddisp.pl?gene=MATN4
**NOTCH1**	Notch Receptor 1	Protein Coding	51	GC09M136602	5.04	https://www.genecards.org/cgi-bin/carddisp.pl?gene=NOTCH1
**CTSL**	Cathepsin L	Protein Coding	46	GC09P087725	5.04	https://www.genecards.org/cgi-bin/carddisp.pl?gene=CTSL
**ADAMTS9**	ADAM Metallopeptidase With Thrombospondin Type 1 Motif 9	Protein Coding	37	GC03M064501	5.03	https://www.genecards.org/cgi-bin/carddisp.pl?gene=ADAMTS9
**ALOX5**	Arachidonate 5-Lipoxygenase	Protein Coding	48	GC10P045374	5.01	https://www.genecards.org/cgi-bin/carddisp.pl?gene=ALOX5
**HGF**	Hepatocyte Growth Factor	Protein Coding	52	GC07M081699	5.01	https://www.genecards.org/cgi-bin/carddisp.pl?gene=HGF
**COX5A**	Cytochrome C Oxidase Subunit 5A	Protein Coding	43	GC15M074919	5	https://www.genecards.org/cgi-bin/carddisp.pl?gene=COX5A
**ANXA1**	Annexin A1	Protein Coding	49	GC09P073151	4.98	https://www.genecards.org/cgi-bin/carddisp.pl?gene=ANXA1

**BMP4**	Bone Morphogenetic Protein 4	Protein Coding	49	GC14M053949	4.98	https://www.genecards.org/cgi-bin/carddisp.pl?gene=BMP4
**FGFR1**	Fibroblast Growth Factor Receptor 1	Protein Coding	55	GC08M038400	4.95	https://www.genecards.org/cgi-bin/carddisp.pl?gene=FGFR1
**BRAF**	B-Raf Proto-Oncogene, Serine/Threonine Kinase	Protein Coding	54	GC07M140719	4.91	https://www.genecards.org/cgi-bin/carddisp.pl?gene=BRAF
**CDK4**	Cyclin Dependent Kinase 4	Protein Coding	54	GC12M057743	4.91	https://www.genecards.org/cgi-bin/carddisp.pl?gene=CDK4
**RET**	Ret Proto-Oncogene	Protein Coding	53	GC10P043081	4.91	https://www.genecards.org/cgi-bin/carddisp.pl?gene=RET
**HRAS**	HRas Proto-Oncogene, GTPase	Protein Coding	52	GC11M000635	4.91	https://www.genecards.org/cgi-bin/carddisp.pl?gene=HRAS
**IDH1**	Isocitrate Dehydrogenase (NADP(+)) 1	Protein Coding	52	GC02M208236	4.91	https://www.genecards.org/cgi-bin/carddisp.pl?gene=IDH1
**IDH2**	Isocitrate Dehydrogenase (NADP(+)) 2	Protein Coding	52	GC15M090083	4.91	https://www.genecards.org/cgi-bin/carddisp.pl?gene=IDH2

**KRAS**	KRAS Proto-Oncogene, GTPase	Protein Coding	51	GC12M025204	4.91	https://www.genecards.org/cgi-bin/carddisp.pl?gene=KRAS
**NRAS**	NRAS Proto-Oncogene, GTPase	Protein Coding	50	GC01M114704	4.91	https://www.genecards.org/cgi-bin/carddisp.pl?gene=NRAS
**BRCA2**	BRCA2 DNA Repair Associated	Protein Coding	49	GC13P032315	4.91	https://www.genecards.org/cgi-bin/carddisp.pl?gene=BRCA2
**CPT2**	Carnitine Palmitoyltransferase 2	Protein Coding	48	GC01P053196	4.91	https://www.genecards.org/cgi-bin/carddisp.pl?gene=CPT2
**IDS**	Iduronate 2-Sulfatase	Protein Coding	48	GC0XM149476	4.91	https://www.genecards.org/cgi-bin/carddisp.pl?gene=IDS
**LIG4**	DNA Ligase 4	Protein Coding	48	GC13M108207	4.91	https://www.genecards.org/cgi-bin/carddisp.pl?gene=LIG4
**LYZ**	Lysozyme	Protein Coding	47	GC12P069348	4.91	https://www.genecards.org/cgi-bin/carddisp.pl?gene=LYZ
**SLC11A1**	Solute Carrier Family 11 Member 1	Protein Coding	47	GC02P218382	4.91	https://www.genecards.org/cgi-bin/carddisp.pl?gene=SLC11A1
**ERCC4**	ERCC Excision Repair 4, Endonuclease Catalytic Subunit	Protein Coding	45	GC16P013920	4.91	https://www.genecards.org/cgi-bin/carddisp.pl?gene=ERCC4

**TCIRG1**	T Cell Immune Regulator 1, ATPase H+ Transporting V0 Subunit A3	Protein Coding	45	GC11P068038	4.91	https://www.genecards.org/cgi-bin/carddisp.pl?gene=TCIRG1
**ARID1B**	AT-Rich Interaction Domain 1B	Protein Coding	44	GC06P156777	4.91	https://www.genecards.org/cgi-bin/carddisp.pl?gene=ARID1B
**LTBP2**	Latent Transforming Growth Factor Beta Binding Protein 2	Protein Coding	43	GC14M074498	4.91	https://www.genecards.org/cgi-bin/carddisp.pl?gene=LTBP2
**FBLN1**	Fibulin 1	Protein Coding	43	GC22P045502	4.91	https://www.genecards.org/cgi-bin/carddisp.pl?gene=FBLN1
**CD244**	CD244 Molecule	Protein Coding	43	GC01M160830	4.91	https://www.genecards.org/cgi-bin/carddisp.pl?gene=CD244
**TNR**	Tenascin R	Protein Coding	41	GC01M175291	4.91	https://www.genecards.org/cgi-bin/carddisp.pl?gene=TNR
**KMT2D**	Lysine Methyltransferase 2D	Protein Coding	41	GC12M049018	4.91	https://www.genecards.org/cgi-bin/carddisp.pl?gene=KMT2D
**FKBP14**	FKBP Prolyl Isomerase 14	Protein Coding	40	GC07M030010	4.91	https://www.genecards.org/cgi-bin/carddisp.pl?gene=FKBP14

**COG4**	Component Of Oligomeric Golgi Complex 4	Protein Coding	40	GC16M070482	4.91	https://www.genecards.org/cgi-bin/carddisp.pl?gene=COG4
**MICA**	MHC Class I Polypeptide-Related Sequence A	Protein Coding	39	GC06P031399	4.91	https://www.genecards.org/cgi-bin/carddisp.pl?gene=MICA
**HNRNPL**	Heterogeneous Nuclear Ribonucleoprotein L	Protein Coding	39	GC19M038836	4.91	https://www.genecards.org/cgi-bin/carddisp.pl?gene=HNRNPL
**TECTA**	Tectorin Alpha	Protein Coding	39	GC11P121101	4.91	https://www.genecards.org/cgi-bin/carddisp.pl?gene=TECTA
**P3H1**	Prolyl 3-Hydroxylase 1	Protein Coding	39	GC01M042746	4.91	https://www.genecards.org/cgi-bin/carddisp.pl?gene=P3H1
**NFKBIL1**	NFKB Inhibitor Like 1	Protein Coding	37	GC06P047302	4.91	https://www.genecards.org/cgi-bin/carddisp.pl?gene=NFKBIL1
**NINL**	Ninein Like	Protein Coding	36	GC20M025452	4.91	https://www.genecards.org/cgi-bin/carddisp.pl?gene=NINL
**STRC**	Stereocilin	Protein Coding	35	GC15M043599	4.91	https://www.genecards.org/cgi-bin/carddisp.pl?gene=STRC

**CEACAM16**	CEA Cell Adhesion Molecule 16, Tectorial Membrane Component	Protein Coding	33	GC19P044699	4.91	https://www.genecards.org/cgi-bin/carddisp.pl?gene=CEACAM16
**OTOG**	Otogelin	Protein Coding	33	GC11P017530	4.91	https://www.genecards.org/cgi-bin/carddisp.pl?gene=OTOG
**H2AC18**	H2A Clustered Histone 18	Protein Coding	26	GC01M149961	4.91	https://www.genecards.org/cgi-bin/carddisp.pl?gene=H2AC18
**MIR132**	MicroRNA 132	RNA Gene	21	GC17M002049	4.91	https://www.genecards.org/cgi-bin/carddisp.pl?gene=MIR132
**MIR106B**	MicroRNA 106b	RNA Gene	21	GC07M100284	4.91	https://www.genecards.org/cgi-bin/carddisp.pl?gene=MIR106B
**MIR25**	MicroRNA 25	RNA Gene	20	GC07M100093	4.91	https://www.genecards.org/cgi-bin/carddisp.pl?gene=MIR25
**MIR93**	MicroRNA 93	RNA Gene	20	GC07M100282	4.91	https://www.genecards.org/cgi-bin/carddisp.pl?gene=MIR93
**MIR32**	MicroRNA 32	RNA Gene	19	GC09M109046	4.91	https://www.genecards.org/cgi-bin/carddisp.pl?gene=MIR32

**SPDT**	Spondyloepiphyseal Dysplasia Tarda, Autosomal Dominant	Genetic Locus	2	GC12U902501	4.91	https://www.genecards.org/cgi-bin/carddisp.pl?gene=SPDT
**CD14**	CD14 Molecule	Protein Coding	44	GC05M140631	4.91	https://www.genecards.org/cgi-bin/carddisp.pl?gene=CD14
**MMP12**	Matrix Metallopeptidase 12	Protein Coding	44	GC11M102862	4.91	https://www.genecards.org/cgi-bin/carddisp.pl?gene=MMP12
**TRAF6**	TNF Receptor Associated Factor 6	Protein Coding	47	GC11M036467	4.89	https://www.genecards.org/cgi-bin/carddisp.pl?gene=TRAF6
**ENG**	Endoglin	Protein Coding	46	GC09M127815	4.88	https://www.genecards.org/cgi-bin/carddisp.pl?gene=ENG
**CD40**	CD40 Molecule	Protein Coding	48	GC20P046118	4.88	https://www.genecards.org/cgi-bin/carddisp.pl?gene=CD40
**IL17RA**	Interleukin 17 Receptor A	Protein Coding	44	GC22P017086	4.88	https://www.genecards.org/cgi-bin/carddisp.pl?gene=IL17RA
**SERPINA1**	Serpin Family A Member 1	Protein Coding	49	GC14M094376	4.87	https://www.genecards.org/cgi-bin/carddisp.pl?gene=SERPINA1

**SERPINE1**	Serpin Family E Member 1	Protein Coding	50	GC07P101127	4.86	https://www.genecards.org/cgi-bin/carddisp.pl?gene=SERPINE1
**LTBP1**	Latent Transforming Growth Factor Beta Binding Protein 1	Protein Coding	43	GC02P032915	4.85	https://www.genecards.org/cgi-bin/carddisp.pl?gene=LTBP1
**ITGAM**	Integrin Subunit Alpha M	Protein Coding	46	GC16P031550	4.85	https://www.genecards.org/cgi-bin/carddisp.pl?gene=ITGAM
**CCL18**	C-C Motif Chemokine Ligand 18	Protein Coding	36	GC17P036064	4.85	https://www.genecards.org/cgi-bin/carddisp.pl?gene=CCL18
**IDUA**	Alpha-L-Iduronidase	Protein Coding	42	GC04P000986	4.84	https://www.genecards.org/cgi-bin/carddisp.pl?gene=IDUA
**LEPR**	Leptin Receptor	Protein Coding	49	GC01P065421	4.82	https://www.genecards.org/cgi-bin/carddisp.pl?gene=LEPR
**TLR8**	Toll Like Receptor 8	Protein Coding	47	GC0XP012924	4.82	https://www.genecards.org/cgi-bin/carddisp.pl?gene=TLR8
**CSF1R**	Colony Stimulating Factor 1 Receptor	Protein Coding	52	GC05M150053	4.82	https://www.genecards.org/cgi-bin/carddisp.pl?gene=CSF1R

**LOX**	Lysyl Oxidase	Protein Coding	44	GC05M122063	4.79	https://www.genecards.org/cgi-bin/carddisp.pl?gene=LOX
**MMP17**	Matrix Metallopeptidase 17	Protein Coding	44	GC12P131828	4.79	https://www.genecards.org/cgi-bin/carddisp.pl?gene=MMP17
**FOSL1**	FOS Like 1, AP-1 Transcription Factor Subunit	Protein Coding	44	GC11M065909	4.79	https://www.genecards.org/cgi-bin/carddisp.pl?gene=FOSL1
**TRPV1**	Transient Receptor Potential Cation Channel Subfamily V Member 1	Protein Coding	46	GC17M003565	4.78	https://www.genecards.org/cgi-bin/carddisp.pl?gene=TRPV1
**CCR2**	C-C Motif Chemokine Receptor 2	Protein Coding	45	GC03P046356	4.76	https://www.genecards.org/cgi-bin/carddisp.pl?gene=CCR2
**SELL**	Selectin L	Protein Coding	42	GC01M169690	4.75	https://www.genecards.org/cgi-bin/carddisp.pl?gene=SELL
**MAPK14**	Mitogen-Activated Protein Kinase 14	Protein Coding	51	GC06P047451	4.75	https://www.genecards.org/cgi-bin/carddisp.pl?gene=MAPK14
**TGIF1**	TGFB Induced Factor Homeobox 1	Protein Coding	45	GC18P003411	4.73	https://www.genecards.org/cgi-bin/carddisp.pl?gene=TGIF1

**CTLA4**	Cytotoxic T-Lymphocyte Associated Protein 4	Protein Coding	45	GC02P203867	4.71	https://www.genecards.org/cgi-bin/carddisp.pl?gene=CTLA4
**TLR8-AS1**	TLR8 Antisense RNA 1	RNA Gene	13	GC0XM012922	4.69	https://www.genecards.org/cgi-bin/carddisp.pl?gene=TLR8-AS1
**CDKN2A**	Cyclin Dependent Kinase Inhibitor 2A	Protein Coding	51	GC09M021967	4.69	https://www.genecards.org/cgi-bin/carddisp.pl?gene=CDKN2A
**PIK3CG**	Phosphatidylinositol-4,5-Bisphosphate 3-Kinase Catalytic Subunit Gamma	Protein Coding	48	GC07P106865	4.69	https://www.genecards.org/cgi-bin/carddisp.pl?gene=PIK3CG
**IRF5**	Interferon Regulatory Factor 5	Protein Coding	48	GC07P128937	4.69	https://www.genecards.org/cgi-bin/carddisp.pl?gene=IRF5
**MIR9-1**	MicroRNA 9-1	RNA Gene	20	GC01M156420	4.69	https://www.genecards.org/cgi-bin/carddisp.pl?gene=MIR9-1
**MIR139**	MicroRNA 139	RNA Gene	19	GC11M072615	4.69	https://www.genecards.org/cgi-bin/carddisp.pl?gene=MIR139
**MTHFR**	Methylenetetrahydrofolate Reductase	Protein Coding	47	GC01M011785	4.68	https://www.genecards.org/cgi-bin/carddisp.pl?gene=MTHFR

**MIR26A1**	MicroRNA 26a-1	RNA Gene	20	GC03P037969	4.67	https://www.genecards.org/cgi-bin/carddisp.pl?gene=MIR26A1
**NLRP3**	NLR Family Pyrin Domain Containing 3	Protein Coding	47	GC01P247415	4.66	https://www.genecards.org/cgi-bin/carddisp.pl?gene=NLRP3
**LIF**	LIF Interleukin 6 Family Cytokine	Protein Coding	43	GC22M030240	4.66	https://www.genecards.org/cgi-bin/carddisp.pl?gene=LIF
**NGF**	Nerve Growth Factor	Protein Coding	50	GC01M115285	4.66	https://www.genecards.org/cgi-bin/carddisp.pl?gene=NGF
**GGT1**	Gamma-Glutamyltransferase 1	Protein Coding	46	GC22P024927	4.65	https://www.genecards.org/cgi-bin/carddisp.pl?gene=GGT1
**SERPINH1**	Serpin Family H Member 1	Protein Coding	45	GC11P075562	4.65	https://www.genecards.org/cgi-bin/carddisp.pl?gene=SERPINH1
**CD80**	CD80 Molecule	Protein Coding	41	GC03M119524	4.65	https://www.genecards.org/cgi-bin/carddisp.pl?gene=CD80
**SCN9A**	Sodium Voltage-Gated Channel Alpha Subunit 9	Protein Coding	47	GC02M166195	4.65	https://www.genecards.org/cgi-bin/carddisp.pl?gene=SCN9A
**HSPD1**	Heat Shock Protein Family D (Hsp60) Member 1	Protein Coding	47	GC02M197486	4.65	https://www.genecards.org/cgi-bin/carddisp.pl?gene=HSPD1

**ELANE**	Elastase, Neutrophil Expressed	Protein Coding	46	GC19P000854	4.64	https://www.genecards.org/cgi-bin/carddisp.pl?gene=ELANE
**CCR5**	C-C Motif Chemokine Receptor 5	Protein Coding	46	GC03P046383	4.64	https://www.genecards.org/cgi-bin/carddisp.pl?gene=CCR5
**CASP8**	Caspase 8	Protein Coding	52	GC02P201233	4.58	https://www.genecards.org/cgi-bin/carddisp.pl?gene=CASP8
**HLA-DQA1**	Major Histocompatibility Complex, Class II, DQ Alpha 1	Protein Coding	42	GC06P047340	4.57	https://www.genecards.org/cgi-bin/carddisp.pl?gene=HLA-DQA1
**MALAT1**	Metastasis Associated Lung Adenocarcinoma Transcript 1	RNA Gene	24	GC11P065806	4.57	https://www.genecards.org/cgi-bin/carddisp.pl?gene=MALAT1
**MIR145**	MicroRNA 145	RNA Gene	21	GC05P149430	4.53	https://www.genecards.org/cgi-bin/carddisp.pl?gene=MIR145
**SFRP4**	Secreted Frizzled Related Protein 4	Protein Coding	43	GC07M037912	4.52	https://www.genecards.org/cgi-bin/carddisp.pl?gene=SFRP4
**SRC**	SRC Proto-Oncogene, Non-Receptor Tyrosine Kinase	Protein Coding	51	GC20P037344	4.52	https://www.genecards.org/cgi-bin/carddisp.pl?gene=SRC

**STAT4**	Signal Transducer And Activator Of Transcription 4	Protein Coding	45	GC02M191029	4.52	https://www.genecards.org/cgi-bin/carddisp.pl?gene=STAT4
**ADA**	Adenosine Deaminase	Protein Coding	51	GC20M044620	4.44	https://www.genecards.org/cgi-bin/carddisp.pl?gene=ADA
**ITGB5**	Integrin Subunit Beta 5	Protein Coding	45	GC03M124761	4.44	https://www.genecards.org/cgi-bin/carddisp.pl?gene=ITGB5
**MRC1**	Mannose Receptor C-Type 1	Protein Coding	39	GC10P017809	4.42	https://www.genecards.org/cgi-bin/carddisp.pl?gene=MRC1
**HLA-DQA2**	Major Histocompatibility Complex, Class II, DQ Alpha 2	Protein Coding	37	GC06P032741	4.42	https://www.genecards.org/cgi-bin/carddisp.pl?gene=HLA-DQA2
**IL16**	Interleukin 16	Protein Coding	42	GC15P081159	4.41	https://www.genecards.org/cgi-bin/carddisp.pl?gene=IL16
**IL5**	Interleukin 5	Protein Coding	44	GC05M132541	4.41	https://www.genecards.org/cgi-bin/carddisp.pl?gene=IL5
**CX3CL1**	C-X3-C Motif Chemokine Ligand 1	Protein Coding	42	GC16P057372	4.41	https://www.genecards.org/cgi-bin/carddisp.pl?gene=CX3CL1
**EDEM2**	ER Degradation Enhancing Alpha-Mannosidase Like Protein 2	Protein Coding	40	GC20M035115	4.39	https://www.genecards.org/cgi-bin/carddisp.pl?gene=EDEM2

**LINC00976**	Long Intergenic Non-Protein Coding RNA 976	RNA Gene	12	GC08M128634	4.39	https://www.genecards.org/cgi-bin/carddisp.pl?gene=LINC00976
**CD28**	CD28 Molecule	Protein Coding	47	GC02P203706	4.38	https://www.genecards.org/cgi-bin/carddisp.pl?gene=CD28
**CASP1**	Caspase 1	Protein Coding	50	GC11M105025	4.38	https://www.genecards.org/cgi-bin/carddisp.pl?gene=CASP1
**MIR335**	MicroRNA 335	RNA Gene	18	GC07P130496	4.36	https://www.genecards.org/cgi-bin/carddisp.pl?gene=MIR335
**BDNF**	Brain Derived Neurotrophic Factor	Protein Coding	47	GC11M027654	4.34	https://www.genecards.org/cgi-bin/carddisp.pl?gene=BDNF
**CXCL6**	C-X-C Motif Chemokine Ligand 6	Protein Coding	40	GC04P073837	4.31	https://www.genecards.org/cgi-bin/carddisp.pl?gene=CXCL6
**SELE**	Selectin E	Protein Coding	44	GC01M169722	4.3	https://www.genecards.org/cgi-bin/carddisp.pl?gene=SELE
**SOX5**	SRY-Box Transcription Factor 5	Protein Coding	46	GC12M023529	4.29	https://www.genecards.org/cgi-bin/carddisp.pl?gene=SOX5

**VIP**	Vasoactive Intestinal Peptide	Protein Coding	44	GC06P152750	4.29	https://www.genecards.org/cgi-bin/carddisp.pl?gene=VIP
**TLR3**	Toll Like Receptor 3	Protein Coding	52	GC04P186059	4.28	https://www.genecards.org/cgi-bin/carddisp.pl?gene=TLR3
**ANXA5**	Annexin A5	Protein Coding	46	GC04M121667	4.26	https://www.genecards.org/cgi-bin/carddisp.pl?gene=ANXA5
**PRKAR1A**	Protein Kinase CAMP-Dependent Type I Regulatory Subunit Alpha	Protein Coding	51	GC17P068414	4.25	https://www.genecards.org/cgi-bin/carddisp.pl?gene=PRKAR1A
**CDH1**	Cadherin 1	Protein Coding	50	GC16P068737	4.25	https://www.genecards.org/cgi-bin/carddisp.pl?gene=CDH1
**ACTB**	Actin Beta	Protein Coding	49	GC07M005527	4.25	https://www.genecards.org/cgi-bin/carddisp.pl?gene=ACTB
**CD4**	CD4 Molecule	Protein Coding	49	GC12P006786	4.25	https://www.genecards.org/cgi-bin/carddisp.pl?gene=CD4
**CDKN1B**	Cyclin Dependent Kinase Inhibitor 1B	Protein Coding	48	GC12P012716	4.25	https://www.genecards.org/cgi-bin/carddisp.pl?gene=CDKN1B
**VWF**	Von Willebrand Factor	Protein Coding	48	GC12M005917	4.25	https://www.genecards.org/cgi-bin/carddisp.pl?gene=VWF

**ARSA**	Arylsulfatase A	Protein Coding	47	GC22M050622	4.25	https://www.genecards.org/cgi-bin/carddisp.pl?gene=ARSA
**MITF**	Melanocyte Inducing Transcription Factor	Protein Coding	47	GC03P069788	4.25	https://www.genecards.org/cgi-bin/carddisp.pl?gene=MITF
**IKZF1**	IKAROS Family Zinc Finger 1	Protein Coding	47	GC07P050303	4.25	https://www.genecards.org/cgi-bin/carddisp.pl?gene=IKZF1
**GPT2**	Glutamic--Pyruvic Transaminase 2	Protein Coding	46	GC16P046885	4.25	https://www.genecards.org/cgi-bin/carddisp.pl?gene=GPT2
**REL**	REL Proto-Oncogene, NF-KB Subunit	Protein Coding	46	GC02P060881	4.25	https://www.genecards.org/cgi-bin/carddisp.pl?gene=REL
**PRODH**	Proline Dehydrogenase 1	Protein Coding	45	GC22M018912	4.25	https://www.genecards.org/cgi-bin/carddisp.pl?gene=PRODH
**HAMP**	Hepcidin Antimicrobial Peptide	Protein Coding	44	GC19P038216	4.25	https://www.genecards.org/cgi-bin/carddisp.pl?gene=HAMP
**SLC34A1**	Solute Carrier Family 34 Member 1	Protein Coding	44	GC05P177380	4.25	https://www.genecards.org/cgi-bin/carddisp.pl?gene=SLC34A1

**FMO1**	Flavin Containing Dimethylaniline Monoxygenase 1	Protein Coding	43	GC01P171248	4.25	https://www.genecards.org/cgi-bin/carddisp.pl?gene=FMO1
**FAM20C**	FAM20C Golgi Associated Secretory Pathway Kinase	Protein Coding	41	GC07P000192	4.25	https://www.genecards.org/cgi-bin/carddisp.pl?gene=FAM20C
**FBN2**	Fibrillin 2	Protein Coding	41	GC05M128257	4.25	https://www.genecards.org/cgi-bin/carddisp.pl?gene=FBN2
**SLC34A3**	Solute Carrier Family 34 Member 3	Protein Coding	41	GC09P137230	4.25	https://www.genecards.org/cgi-bin/carddisp.pl?gene=SLC34A3
**PIGB**	Phosphatidylinositol Glycan Anchor Biosynthesis Class B	Protein Coding	40	GC15P055318	4.25	https://www.genecards.org/cgi-bin/carddisp.pl?gene=PIGB
**HPS5**	HPS5 Biogenesis Of Lysosomal Organelles Complex 2 Subunit 2	Protein Coding	39	GC11M018278	4.25	https://www.genecards.org/cgi-bin/carddisp.pl?gene=HPS5
**ICOSLG**	Inducible T Cell Costimulator Ligand	Protein Coding	39	GC21M044222	4.25	https://www.genecards.org/cgi-bin/carddisp.pl?gene=ICOSLG
**SMYD1**	SET And MYND Domain Containing 1	Protein Coding	39	GC02P088068	4.25	https://www.genecards.org/cgi-bin/carddisp.pl?gene=SMYD1

**GBP4**	Guanylate Binding Protein 4	Protein Coding	37	GC01M089181	4.25	https://www.genecards.org/cgi-bin/carddisp.pl?gene=GBP4
**MRAP**	Melanocortin 2 Receptor Accessory Protein	Protein Coding	37	GC21P032291	4.25	https://www.genecards.org/cgi-bin/carddisp.pl?gene=MRAP
**PDZD11**	PDZ Domain Containing 11	Protein Coding	37	GC0XM070286	4.25	https://www.genecards.org/cgi-bin/carddisp.pl?gene=PDZD11
**PALM**	Paralemmin	Protein Coding	36	GC19P000708	4.25	https://www.genecards.org/cgi-bin/carddisp.pl?gene=PALM
**PALMD**	Palmdelphin	Protein Coding	34	GC01P099574	4.25	https://www.genecards.org/cgi-bin/carddisp.pl?gene=PALMD
**PRDM10**	PR/SET Domain 10	Protein Coding	34	GC11M129899	4.25	https://www.genecards.org/cgi-bin/carddisp.pl?gene=PRDM10
**H3-2**	H3.2 Histone (Putative)	Protein Coding	17	GC01M143894	4.25	https://www.genecards.org/cgi-bin/carddisp.pl?gene=H3-2
**LOC102723692**	Uncharacterized LOC102723692	RNA Gene	8	GC16P017134	4.25	https://www.genecards.org/cgi-bin/carddisp.pl?gene=LOC102723692

**LOC101448202**	Uncharacterized LOC101448202	RNA Gene	8	GC09M134819	4.25	https://www.genecards.org/cgi-bin/carddisp.pl?gene=LOC101448202
**LOC113939944**	Sharpr-MPRA Regulatory Region 9539	Biological Region	1	GC15P048520	4.25	https://www.genecards.org/cgi-bin/carddisp.pl?gene=LOC113939944
**CD163**	CD163 Molecule	Protein Coding	42	GC12M007471	4.24	https://www.genecards.org/cgi-bin/carddisp.pl?gene=CD163
**CCR7**	C-C Motif Chemokine Receptor 7	Protein Coding	45	GC17M040556	4.24	https://www.genecards.org/cgi-bin/carddisp.pl?gene=CCR7
**PRL**	Prolactin	Protein Coding	44	GC06M022230	4.23	https://www.genecards.org/cgi-bin/carddisp.pl?gene=PRL
**CDKN1A**	Cyclin Dependent Kinase Inhibitor 1A	Protein Coding	50	GC06P047460	4.23	https://www.genecards.org/cgi-bin/carddisp.pl?gene=CDKN1A
**LEPQTL1**	Leptin, Serum Levels Of	Genetic Locus	3	GC02U903086	4.2	https://www.genecards.org/cgi-bin/carddisp.pl?gene=LEPQTL1
**PLA2G2A**	Phospholipase A2 Group IIA	Protein Coding	45	GC01M019975	4.19	https://www.genecards.org/cgi-bin/carddisp.pl?gene=PLA2G2A

**CXCR3**	C-X-C Motif Chemokine Receptor 3	Protein Coding	44	GC0XM071615	4.18	https://www.genecards.org/cgi-bin/carddisp.pl?gene=CXCR3
**CYCS**	Cytochrome C, Somatic	Protein Coding	48	GC07M025118	4.18	https://www.genecards.org/cgi-bin/carddisp.pl?gene=CYCS
**MIR27A**	MicroRNA 27a	RNA Gene	22	GC19M014010	4.17	https://www.genecards.org/cgi-bin/carddisp.pl?gene=MIR27A
**F3**	Coagulation Factor III, Tissue Factor	Protein Coding	45	GC01M094530	4.17	https://www.genecards.org/cgi-bin/carddisp.pl?gene=F3
**ANGPT1**	Angiopoietin 1	Protein Coding	45	GC08M107246	4.17	https://www.genecards.org/cgi-bin/carddisp.pl?gene=ANGPT1
**CSF3**	Colony Stimulating Factor 3	Protein Coding	40	GC17P040015	4.17	https://www.genecards.org/cgi-bin/carddisp.pl?gene=CSF3
**CXCL10**	C-X-C Motif Chemokine Ligand 10	Protein Coding	44	GC04M076021	4.16	https://www.genecards.org/cgi-bin/carddisp.pl?gene=CXCL10
**IKBKB**	Inhibitor Of Nuclear Factor Kappa B Kinase Subunit Beta	Protein Coding	52	GC08P042271	4.15	https://www.genecards.org/cgi-bin/carddisp.pl?gene=IKBKB

**ITGB2**	Integrin Subunit Beta 2	Protein Coding	50	GC21M044885	4.15	https://www.genecards.org/cgi-bin/carddisp.pl?gene=ITGB2
**CXCL5**	C-X-C Motif Chemokine Ligand 5	Protein Coding	40	GC04M073995	4.15	https://www.genecards.org/cgi-bin/carddisp.pl?gene=CXCL5
**TNFRSF10A**	TNF Receptor Superfamily Member 10a	Protein Coding	45	GC08M023190	4.12	https://www.genecards.org/cgi-bin/carddisp.pl?gene=TNFRSF10A
**MMP14**	Matrix Metallopeptidase 14	Protein Coding	51	GC14P025277	4.11	https://www.genecards.org/cgi-bin/carddisp.pl?gene=MMP14
**MIR21**	MicroRNA 21	RNA Gene	24	GC17P059841	4.1	https://www.genecards.org/cgi-bin/carddisp.pl?gene=MIR21
**MTOR**	Mechanistic Target Of Rapamycin Kinase	Protein Coding	54	GC01M011106	4.09	https://www.genecards.org/cgi-bin/carddisp.pl?gene=MTOR
**F2**	Coagulation Factor II, Thrombin	Protein Coding	48	GC11P046720	4.08	https://www.genecards.org/cgi-bin/carddisp.pl?gene=F2
**GHRHR**	Growth Hormone Releasing Hormone Receptor	Protein Coding	43	GC07P030978	4.08	https://www.genecards.org/cgi-bin/carddisp.pl?gene=GHRHR

**IGF1R**	Insulin Like Growth Factor 1 Receptor	Protein Coding	54	GC15P098648	4.08	https://www.genecards.org/cgi-bin/carddisp.pl?gene=IGF1R
**OPRD1**	Opioid Receptor Delta 1	Protein Coding	44	GC01P028812	4.07	https://www.genecards.org/cgi-bin/carddisp.pl?gene=OPRD1
**TNFRSF13C**	TNF Receptor Superfamily Member 13C	Protein Coding	44	GC22M045411	4.06	https://www.genecards.org/cgi-bin/carddisp.pl?gene=TNFRSF13C
**CXCL2**	C-X-C Motif Chemokine Ligand 2	Protein Coding	40	GC04M074097	4.06	https://www.genecards.org/cgi-bin/carddisp.pl?gene=CXCL2
**CP**	Ceruloplasmin	Protein Coding	47	GC03M149162	4.04	https://www.genecards.org/cgi-bin/carddisp.pl?gene=CP
**SPARC**	Secreted Protein Acidic And Cysteine Rich	Protein Coding	50	GC05M151639	4.04	https://www.genecards.org/cgi-bin/carddisp.pl?gene=SPARC
**IGFBP2**	Insulin Like Growth Factor Binding Protein 2	Protein Coding	43	GC02P216632	4.04	https://www.genecards.org/cgi-bin/carddisp.pl?gene=IGFBP2
**MYC**	MYC Proto-Oncogene, BHLH Transcription Factor	Protein Coding	51	GC08P127735	4.04	https://www.genecards.org/cgi-bin/carddisp.pl?gene=MYC

**MET**	MET Proto-Oncogene, Receptor Tyrosine Kinase	Protein Coding	54	GC07P116672	4.02	https://www.genecards.org/cgi-bin/carddisp.pl?gene=MET
**IL2RA**	Interleukin 2 Receptor Subunit Alpha	Protein Coding	50	GC10M006010	4.02	https://www.genecards.org/cgi-bin/carddisp.pl?gene=IL2RA
**TLR10**	Toll Like Receptor 10	Protein Coding	40	GC04M038773	4.02	https://www.genecards.org/cgi-bin/carddisp.pl?gene=TLR10
**ATM**	ATM Serine/Threonine Kinase	Protein Coding	54	GC11P108222	4.01	https://www.genecards.org/cgi-bin/carddisp.pl?gene=ATM
**VCAN**	Versican	Protein Coding	47	GC05P083471	4	https://www.genecards.org/cgi-bin/carddisp.pl?gene=VCAN
**AKT1**	AKT Serine/Threonine Kinase 1	Protein Coding	54	GC14M104769	4	https://www.genecards.org/cgi-bin/carddisp.pl?gene=AKT1
**HAS1**	Hyaluronan Synthase 1	Protein Coding	39	GC19M051714	4	https://www.genecards.org/cgi-bin/carddisp.pl?gene=HAS1
**CXCL9**	C-X-C Motif Chemokine Ligand 9	Protein Coding	39	GC04M076001	3.97	https://www.genecards.org/cgi-bin/carddisp.pl?gene=CXCL9

**MIR221**	MicroRNA 221	RNA Gene	20	GC0XM045746	3.97	https://www.genecards.org/cgi-bin/carddisp.pl?gene=MIR221
**IL22**	Interleukin 22	Protein Coding	41	GC12M068248	3.96	https://www.genecards.org/cgi-bin/carddisp.pl?gene=IL22
**ADAM12**	ADAM Metallopeptidase Domain 12	Protein Coding	45	GC10M126012	3.96	https://www.genecards.org/cgi-bin/carddisp.pl?gene=ADAM12
**COMT**	Catechol-O-Methyltransferase	Protein Coding	51	GC22P019941	3.94	https://www.genecards.org/cgi-bin/carddisp.pl?gene=COMT
**PTK2B**	Protein Tyrosine Kinase 2 Beta	Protein Coding	49	GC08P027311	3.94	https://www.genecards.org/cgi-bin/carddisp.pl?gene=PTK2B
**TRAF1**	TNF Receptor Associated Factor 1	Protein Coding	43	GC09M120902	3.94	https://www.genecards.org/cgi-bin/carddisp.pl?gene=TRAF1
**CTSD**	Cathepsin D	Protein Coding	52	GC11M001752	3.94	https://www.genecards.org/cgi-bin/carddisp.pl?gene=CTSD
**CALCR**	Calcitonin Receptor	Protein Coding	48	GC07M093424	3.93	https://www.genecards.org/cgi-bin/carddisp.pl?gene=CALCR

**SERPINC1**	Serpin Family C Member 1	Protein Coding	48	GC01M174153	3.93	https://www.genecards.org/cgi-bin/carddisp.pl?gene=SERPINC1
**TNFSF13**	TNF Superfamily Member 13	Protein Coding	44	GC17P007558	3.93	https://www.genecards.org/cgi-bin/carddisp.pl?gene=TNFSF13
**HSPA4**	Heat Shock Protein Family A (Hsp70) Member 4	Protein Coding	41	GC05P133051	3.93	https://www.genecards.org/cgi-bin/carddisp.pl?gene=HSPA4
**SAA4**	Serum Amyloid A4, Constitutive	Protein Coding	39	GC11M018234	3.93	https://www.genecards.org/cgi-bin/carddisp.pl?gene=SAA4
**IL32**	Interleukin 32	Protein Coding	40	GC16P004242	3.92	https://www.genecards.org/cgi-bin/carddisp.pl?gene=IL32
**FGF1**	Fibroblast Growth Factor 1	Protein Coding	48	GC05M142555	3.9	https://www.genecards.org/cgi-bin/carddisp.pl?gene=FGF1
**PROCR**	Protein C Receptor	Protein Coding	42	GC20P035182	3.88	https://www.genecards.org/cgi-bin/carddisp.pl?gene=PROCR
**NR3C1**	Nuclear Receptor Subfamily 3 Group C Member 1	Protein Coding	50	GC05M143277	3.88	https://www.genecards.org/cgi-bin/carddisp.pl?gene=NR3C1

**PTPRC**	Protein Tyrosine Phosphatase Receptor Type C	Protein Coding	51	GC01P198607	3.87	https://www.genecards.org/cgi-bin/carddisp.pl?gene=PTPRC
**TEK**	TEK Receptor Tyrosine Kinase	Protein Coding	50	GC09P027109	3.87	https://www.genecards.org/cgi-bin/carddisp.pl?gene=TEK
**LRRK2**	Leucine Rich Repeat Kinase 2	Protein Coding	49	GC12P040196	3.87	https://www.genecards.org/cgi-bin/carddisp.pl?gene=LRRK2
**XIAP**	X-Linked Inhibitor Of Apoptosis	Protein Coding	49	GC0XP123859	3.87	https://www.genecards.org/cgi-bin/carddisp.pl?gene=XIAP
**KDM1A**	Lysine Demethylase 1A	Protein Coding	47	GC01P023019	3.87	https://www.genecards.org/cgi-bin/carddisp.pl?gene=KDM1A
**HPSE**	Heparanase	Protein Coding	44	GC04M083292	3.87	https://www.genecards.org/cgi-bin/carddisp.pl?gene=HPSE
**CR1**	Complement C3b/C4b Receptor 1 (Knops Blood Group)	Protein Coding	44	GC01P207496	3.87	https://www.genecards.org/cgi-bin/carddisp.pl?gene=CR1
**IGFBP1**	Insulin Like Growth Factor Binding Protein 1	Protein Coding	43	GC07P046552	3.87	https://www.genecards.org/cgi-bin/carddisp.pl?gene=IGFBP1
**STEAP4**	STEAP4 Metalloreductase	Protein Coding	40	GC07M088288	3.87	https://www.genecards.org/cgi-bin/carddisp.pl?gene=STEAP4

**OLAH**	Oleoyl-ACP Hydrolase	Protein Coding	35	GC10P015032	3.87	https://www.genecards.org/cgi-bin/carddisp.pl?gene=OLAH
**CCR1**	C-C Motif Chemokine Receptor 1	Protein Coding	45	GC03M046218	3.87	https://www.genecards.org/cgi-bin/carddisp.pl?gene=CCR1
**NPY**	Neuropeptide Y	Protein Coding	45	GC07P024290	3.86	https://www.genecards.org/cgi-bin/carddisp.pl?gene=NPY
**IFNB1**	Interferon Beta 1	Protein Coding	41	GC09M021077	3.86	https://www.genecards.org/cgi-bin/carddisp.pl?gene=IFNB1
**PLAU**	Plasminogen Activator, Urokinase	Protein Coding	51	GC10P073909	3.86	https://www.genecards.org/cgi-bin/carddisp.pl?gene=PLAU
**TMPO**	Thymopoietin	Protein Coding	45	GC12P098515	3.85	https://www.genecards.org/cgi-bin/carddisp.pl?gene=TMPO
**ITGB3**	Integrin Subunit Beta 3	Protein Coding	49	GC17P047254	3.83	https://www.genecards.org/cgi-bin/carddisp.pl?gene=ITGB3
**IL10RA**	Interleukin 10 Receptor Subunit Alpha	Protein Coding	45	GC11P117987	3.83	https://www.genecards.org/cgi-bin/carddisp.pl?gene=IL10RA
**HP**	Haptoglobin	Protein Coding	44	GC16P072089	3.83	https://www.genecards.org/cgi-bin/carddisp.pl?gene=HP

**IL3**	Interleukin 3	Protein Coding	44	GC05P132060	3.83	https://www.genecards.org/cgi-bin/carddisp.pl?gene=IL3
**MIRLET7E**	MicroRNA Let-7e	RNA Gene	20	GC19P051718	3.83	https://www.genecards.org/cgi-bin/carddisp.pl?gene=MIRLET7E
**PTEN**	Phosphatase And Tensin Homolog	Protein Coding	52	GC10P087863	3.8	https://www.genecards.org/cgi-bin/carddisp.pl?gene=PTEN
**CD38**	CD38 Molecule	Protein Coding	45	GC04P015779	3.8	https://www.genecards.org/cgi-bin/carddisp.pl?gene=CD38
**IL37**	Interleukin 37	Protein Coding	37	GC02P115304	3.8	https://www.genecards.org/cgi-bin/carddisp.pl?gene=IL37
**SDC1**	Syndecan 1	Protein Coding	43	GC02M020200	3.78	https://www.genecards.org/cgi-bin/carddisp.pl?gene=SDC1
**SST**	Somatostatin	Protein Coding	42	GC03M187668	3.78	https://www.genecards.org/cgi-bin/carddisp.pl?gene=SST
**CXCR5**	C-X-C Motif Chemokine Receptor 5	Protein Coding	41	GC11P118898	3.75	https://www.genecards.org/cgi-bin/carddisp.pl?gene=CXCR5
**PLOD2**	Procollagen-Lysine,2-Oxoglutarate 5-Dioxygenase 2	Protein Coding	45	GC03M146069	3.75	https://www.genecards.org/cgi-bin/carddisp.pl?gene=PLOD2

**BSG**	Basigin (Ok Blood Group)	Protein Coding	44	GC19P000571	3.75	https://www.genecards.org/cgi-bin/carddisp.pl?gene=BSG
**TUG1**	Taurine Up-Regulated 1	RNA Gene	22	GC22P030969	3.74	https://www.genecards.org/cgi-bin/carddisp.pl?gene=TUG1
**STAT5B**	Signal Transducer And Activator Of Transcription 5B	Protein Coding	49	GC17M042199	3.74	https://www.genecards.org/cgi-bin/carddisp.pl?gene=STAT5B
**HSP90AA1**	Heat Shock Protein 90 Alpha Family Class A Member 1	Protein Coding	48	GC14M102080	3.74	https://www.genecards.org/cgi-bin/carddisp.pl?gene=HSP90AA1
**APOH**	Apolipoprotein H	Protein Coding	44	GC17M066212	3.74	https://www.genecards.org/cgi-bin/carddisp.pl?gene=APOH
**FCGR3A**	Fc Fragment Of IgG Receptor IIIa	Protein Coding	44	GC01M161541	3.74	https://www.genecards.org/cgi-bin/carddisp.pl?gene=FCGR3A
**PNOC**	Prepronociceptin	Protein Coding	37	GC08P028316	3.74	https://www.genecards.org/cgi-bin/carddisp.pl?gene=PNOC
**ADAMTS15**	ADAM Metallopeptidase With Thrombospondin Type 1 Motif 15	Protein Coding	36	GC11P130448	3.74	https://www.genecards.org/cgi-bin/carddisp.pl?gene=ADAMTS15
**GRN**	Granulin Precursor	Protein Coding	47	GC17P044345	3.71	https://www.genecards.org/cgi-bin/carddisp.pl?gene=GRN

**MAF**	MAF BZIP Transcription Factor	Protein Coding	45	GC16M079204	3.71	https://www.genecards.org/cgi-bin/carddisp.pl?gene=MAF
**CAST**	Calpastatin	Protein Coding	45	GC05P096525	3.71	https://www.genecards.org/cgi-bin/carddisp.pl?gene=CAST
**TACR1**	Tachykinin Receptor 1	Protein Coding	45	GC02M075010	3.71	https://www.genecards.org/cgi-bin/carddisp.pl?gene=TACR1
**UQCC1**	Ubiquinol-Cytochrome C Reductase Complex Assembly Factor 1	Protein Coding	33	GC20M035302	3.71	https://www.genecards.org/cgi-bin/carddisp.pl?gene=UQCC1
**XBP1**	X-Box Binding Protein 1	Protein Coding	45	GC22M028794	3.69	https://www.genecards.org/cgi-bin/carddisp.pl?gene=XBP1
**FTO**	FTO Alpha-Ketoglutarate Dependent Dioxygenase	Protein Coding	44	GC16P053737	3.67	https://www.genecards.org/cgi-bin/carddisp.pl?gene=FTO
**CA2**	Carbonic Anhydrase 2	Protein Coding	51	GC08P085463	3.67	https://www.genecards.org/cgi-bin/carddisp.pl?gene=CA2
**SIRT6**	Sirtuin 6	Protein Coding	45	GC19M004174	3.67	https://www.genecards.org/cgi-bin/carddisp.pl?gene=SIRT6
**OPRK1**	Opioid Receptor Kappa 1	Protein Coding	45	GC08M053227	3.67	https://www.genecards.org/cgi-bin/carddisp.pl?gene=OPRK1

**LY96**	Lymphocyte Antigen 96	Protein Coding	42	GC08P073991	3.67	https://www.genecards.org/cgi-bin/carddisp.pl?gene=LY96
**SYVN1**	Synoviolin 1	Protein Coding	40	GC11M065122	3.67	https://www.genecards.org/cgi-bin/carddisp.pl?gene=SYVN1
**MIR30A**	MicroRNA 30a	RNA Gene	20	GC06M071403	3.67	https://www.genecards.org/cgi-bin/carddisp.pl?gene=MIR30A
**MIR675**	MicroRNA 675	RNA Gene	17	GC11M001997	3.67	https://www.genecards.org/cgi-bin/carddisp.pl?gene=MIR675
**CD79A**	CD79a Molecule	Protein Coding	46	GC19P041877	3.66	https://www.genecards.org/cgi-bin/carddisp.pl?gene=CD79A
**PRTN3**	Proteinase 3	Protein Coding	44	GC19P000840	3.66	https://www.genecards.org/cgi-bin/carddisp.pl?gene=PRTN3
**FCGR3B**	Fc Fragment Of IgG Receptor IIIb	Protein Coding	42	GC01M161623	3.66	https://www.genecards.org/cgi-bin/carddisp.pl?gene=FCGR3B
**EP300**	E1A Binding Protein P300	Protein Coding	50	GC22P041091	3.64	https://www.genecards.org/cgi-bin/carddisp.pl?gene=EP300

**CXADR**	CXADR Ig-Like Cell Adhesion Molecule	Protein Coding	43	GC21P017512	3.63	https://www.genecards.org/cgi-bin/carddisp.pl?gene=CXADR
**OS4**	Osteoarthritis, Generalized, Without Dysplasia, Susceptibility To	Genetic Locus	2	GC02U903030	3.63	https://www.genecards.org/cgi-bin/carddisp.pl?gene=OS4
**OS6**	Osteoarthritis Susceptibility 6	Genetic Locus	1	GC03U901212	3.63	https://www.genecards.org/cgi-bin/carddisp.pl?gene=OS6
**MC1R**	Melanocortin 1 Receptor	Protein Coding	47	GC16P089912	3.61	https://www.genecards.org/cgi-bin/carddisp.pl?gene=MC1R
**HABP2**	Hyaluronan Binding Protein 2	Protein Coding	44	GC10P113550	3.61	https://www.genecards.org/cgi-bin/carddisp.pl?gene=HABP2
**IL6ST**	Interleukin 6 Signal Transducer	Protein Coding	45	GC05M055935	3.6	https://www.genecards.org/cgi-bin/carddisp.pl?gene=IL6ST
**IL23R**	Interleukin 23 Receptor	Protein Coding	44	GC01P067138	3.6	https://www.genecards.org/cgi-bin/carddisp.pl?gene=IL23R
**CHIT1**	Chitinase 1	Protein Coding	43	GC01M203181	3.6	https://www.genecards.org/cgi-bin/carddisp.pl?gene=CHIT1
**EZH2**	Enhancer Of Zeste 2 Polycomb Repressive Complex 2 Subunit	Protein Coding	54	GC07M148807	3.6	https://www.genecards.org/cgi-bin/carddisp.pl?gene=EZH2

**RAC1**	Rac Family Small GTPase 1	Protein Coding	49	GC07P006380	3.6	https://www.genecards.org/cgi-bin/carddisp.pl?gene=RAC1
**GPT**	Glutamic--Pyruvic Transaminase	Protein Coding	41	GC08P144502	3.6	https://www.genecards.org/cgi-bin/carddisp.pl?gene=GPT
**FGFR2**	Fibroblast Growth Factor Receptor 2	Protein Coding	54	GC10M121478	3.56	https://www.genecards.org/cgi-bin/carddisp.pl?gene=FGFR2
**CD247**	CD247 Molecule	Protein Coding	49	GC01M167399	3.56	https://www.genecards.org/cgi-bin/carddisp.pl?gene=CD247
**TRPV6**	Transient Receptor Potential Cation Channel Subfamily V Member 6	Protein Coding	46	GC07M142871	3.56	https://www.genecards.org/cgi-bin/carddisp.pl?gene=TRPV6
**BRD4**	Bromodomain Containing 4	Protein Coding	44	GC19M015236	3.56	https://www.genecards.org/cgi-bin/carddisp.pl?gene=BRD4
**GNAS-AS1**	GNAS Antisense RNA 1	RNA Gene	22	GC20M058846	3.56	https://www.genecards.org/cgi-bin/carddisp.pl?gene=GNAS-AS1
**MIR483**	MicroRNA 483	RNA Gene	18	GC11M002188	3.56	https://www.genecards.org/cgi-bin/carddisp.pl?gene=MIR483

**ADRB2**	Adrenoceptor Beta 2	Protein Coding	48	GC05P148825	3.5	https://www.genecards.org/cgi-bin/carddisp.pl?gene=ADRB2
**TRAF3**	TNF Receptor Associated Factor 3	Protein Coding	47	GC14P104639	3.5	https://www.genecards.org/cgi-bin/carddisp.pl?gene=TRAF3
**HLA-A**	Major Histocompatibility Complex, Class I, A	Protein Coding	46	GC06P047265	3.5	https://www.genecards.org/cgi-bin/carddisp.pl?gene=HLA-A
**BCL2L11**	BCL2 Like 11	Protein Coding	45	GC02P111119	3.5	https://www.genecards.org/cgi-bin/carddisp.pl?gene=BCL2L11
**NCAM1**	Neural Cell Adhesion Molecule 1	Protein Coding	45	GC11P112961	3.5	https://www.genecards.org/cgi-bin/carddisp.pl?gene=NCAM1
**BRD2**	Bromodomain Containing 2	Protein Coding	44	GC06P047345	3.5	https://www.genecards.org/cgi-bin/carddisp.pl?gene=BRD2
**LOXL2**	Lysyl Oxidase Like 2	Protein Coding	44	GC08M023296	3.5	https://www.genecards.org/cgi-bin/carddisp.pl?gene=LOXL2
**OCRL**	OCRL Inositol Polyphosphate-5-Phosphatase	Protein Coding	44	GC0XP129539	3.5	https://www.genecards.org/cgi-bin/carddisp.pl?gene=OCRL

**NR1I3**	Nuclear Receptor Subfamily 1 Group I Member 3	Protein Coding	44	GC01M161 229	3.5	https://www.genecards.org/cgi-bin/carddisp.pl?gene=NR1I3
**TSPO**	Translocator Protein	Protein Coding	43	GC22P043 151	3.5	https://www.genecards.org/cgi-bin/carddisp.pl?gene=TSPO
**SOX6**	SRY-Box Transcription Factor 6	Protein Coding	42	GC11M015 949	3.5	https://www.genecards.org/cgi-bin/carddisp.pl?gene=SOX6
**PREP**	Prolyl Endopeptidase	Protein Coding	42	GC06M105 277	3.5	https://www.genecards.org/cgi-bin/carddisp.pl?gene=PREP
**TFEB**	Transcription Factor EB	Protein Coding	42	GC06M042 295	3.5	https://www.genecards.org/cgi-bin/carddisp.pl?gene=TFEB
**FOLR2**	Folate Receptor Beta	Protein Coding	41	GC11P072 216	3.5	https://www.genecards.org/cgi-bin/carddisp.pl?gene=FOLR2
**CHST11**	Carbohydrate Sulfotransferase 11	Protein Coding	41	GC12P104 455	3.5	https://www.genecards.org/cgi-bin/carddisp.pl?gene=CHST11
**SHOX**	Short Stature Homeobox	Protein Coding	38	GC0XP000 624	3.5	https://www.genecards.org/cgi-bin/carddisp.pl?gene=SHOX

**SPON1**	Spondin 1	Protein Coding	36	GC11P013940	3.5	https://www.genecards.org/cgi-bin/carddisp.pl?gene=SPON1
**PVT1**	Pvt1 Oncogene	RNA Gene	25	GC08P127804	3.5	https://www.genecards.org/cgi-bin/carddisp.pl?gene=PVT1
**ZFAS1**	ZNFX1 Antisense RNA 1	RNA Gene	18	GC20P049276	3.5	https://www.genecards.org/cgi-bin/carddisp.pl?gene=ZFAS1
**SMO**	Smoothened, Frizzled Class Receptor	Protein Coding	48	GC07P129463	3.5	https://www.genecards.org/cgi-bin/carddisp.pl?gene=SMO
**FGF18**	Fibroblast Growth Factor 18	Protein Coding	41	GC05P171419	3.5	https://www.genecards.org/cgi-bin/carddisp.pl?gene=FGF18
**NFKB2**	Nuclear Factor Kappa B Subunit 2	Protein Coding	52	GC10P102394	3.47	https://www.genecards.org/cgi-bin/carddisp.pl?gene=NFKB2
**BLK**	BLK Proto-Oncogene, Src Family Tyrosine Kinase	Protein Coding	51	GC08P011486	3.47	https://www.genecards.org/cgi-bin/carddisp.pl?gene=BLK
**PRKG1**	Protein Kinase CGMP-Dependent 1	Protein Coding	51	GC10P050991	3.47	https://www.genecards.org/cgi-bin/carddisp.pl?gene=PRKG1

**APOE**	Apolipoprotein E	Protein Coding	50	GC19P044906	3.47	https://www.genecards.org/cgi-bin/carddisp.pl?gene=APOE
**DRD2**	Dopamine Receptor D2	Protein Coding	50	GC11M113409	3.47	https://www.genecards.org/cgi-bin/carddisp.pl?gene=DRD2
**SNCA**	Synuclein Alpha	Protein Coding	50	GC04M089724	3.47	https://www.genecards.org/cgi-bin/carddisp.pl?gene=SNCA
**GLA**	Galactosidase Alpha	Protein Coding	48	GC0XM101393	3.47	https://www.genecards.org/cgi-bin/carddisp.pl?gene=GLA
**TFRC**	Transferrin Receptor	Protein Coding	48	GC03M196027	3.47	https://www.genecards.org/cgi-bin/carddisp.pl?gene=TFRC
**ZEB1**	Zinc Finger E-Box Binding Homeobox 1	Protein Coding	48	GC10P031318	3.47	https://www.genecards.org/cgi-bin/carddisp.pl?gene=ZEB1
**ZEB2**	Zinc Finger E-Box Binding Homeobox 2	Protein Coding	48	GC02M144384	3.47	https://www.genecards.org/cgi-bin/carddisp.pl?gene=ZEB2
**F7**	Coagulation Factor VII	Protein Coding	47	GC13P113105	3.47	https://www.genecards.org/cgi-bin/carddisp.pl?gene=F7
**ADCY3**	Adenylate Cyclase 3	Protein Coding	47	GC02M024819	3.47	https://www.genecards.org/cgi-bin/carddisp.pl?gene=ADCY3

**GAA**	Glucosidase Alpha, Acid	Protein Coding	47	GC17P080101	3.47	https://www.genecards.org/cgi-bin/carddisp.pl?gene=GAA
**PKLR**	Pyruvate Kinase L/R	Protein Coding	47	GC01M155289	3.47	https://www.genecards.org/cgi-bin/carddisp.pl?gene=PKLR
**SLC11A2**	Solute Carrier Family 11 Member 2	Protein Coding	47	GC12M050952	3.47	https://www.genecards.org/cgi-bin/carddisp.pl?gene=SLC11A2
**PSAP**	Prosaposin	Protein Coding	46	GC10M071816	3.47	https://www.genecards.org/cgi-bin/carddisp.pl?gene=PSAP
**NR4A2**	Nuclear Receptor Subfamily 4 Group A Member 2	Protein Coding	46	GC02M156324	3.47	https://www.genecards.org/cgi-bin/carddisp.pl?gene=NR4A2
**TYRP1**	Tyrosinase Related Protein 1	Protein Coding	46	GC09P012683	3.47	https://www.genecards.org/cgi-bin/carddisp.pl?gene=TYRP1
**AGA**	Aspartylglucosaminidase	Protein Coding	45	GC04M177430	3.47	https://www.genecards.org/cgi-bin/carddisp.pl?gene=AGA
**GALNT3**	Polypeptide N-Acetylgalactosaminyltransferase 3	Protein Coding	45	GC02M165747	3.47	https://www.genecards.org/cgi-bin/carddisp.pl?gene=GALNT3

**HPD**	4-Hydroxyphenylpyruvate Dioxygenase	Protein Coding	45	GC12M121839	3.47	https://www.genecards.org/cgi-bin/carddisp.pl?gene=HPD
**ERCC6**	ERCC Excision Repair 6, Chromatin Remodeling Factor	Protein Coding	45	GC10M049454	3.47	https://www.genecards.org/cgi-bin/carddisp.pl?gene=ERCC6
**GUCY2C**	Guanylate Cyclase 2C	Protein Coding	45	GC12M014612	3.47	https://www.genecards.org/cgi-bin/carddisp.pl?gene=GUCY2C
**SLC26A3**	Solute Carrier Family 26 Member 3	Protein Coding	45	GC07M107765	3.47	https://www.genecards.org/cgi-bin/carddisp.pl?gene=SLC26A3
**TAC3**	Tachykinin Precursor 3	Protein Coding	45	GC12M057009	3.47	https://www.genecards.org/cgi-bin/carddisp.pl?gene=TAC3
**RELB**	RELB Proto-Oncogene, NF-KB Subunit	Protein Coding	45	GC19P045002	3.47	https://www.genecards.org/cgi-bin/carddisp.pl?gene=RELB
**PSTPIP1**	Proline-Serine-Threonine Phosphatase Interacting Protein 1	Protein Coding	45	GC15P076993	3.47	https://www.genecards.org/cgi-bin/carddisp.pl?gene=PSTPIP1
**GH1**	Growth Hormone 1	Protein Coding	44	GC17M063917	3.47	https://www.genecards.org/cgi-bin/carddisp.pl?gene=GH1
**FBLN5**	Fibulin 5	Protein Coding	44	GC14M091869	3.47	https://www.genecards.org/cgi-bin/carddisp.pl?gene=FBLN5

**FMR1**	FMRP Translational Regulator 1	Protein Coding	44	GC0XP147913	3.47	https://www.genecards.org/cgi-bin/carddisp.pl?gene=FMR1
**GSTA3**	Glutathione S-Transferase Alpha 3	Protein Coding	44	GC06M052896	3.47	https://www.genecards.org/cgi-bin/carddisp.pl?gene=GSTA3
**CRYAA**	Crystallin Alpha A	Protein Coding	44	GC21P043169	3.47	https://www.genecards.org/cgi-bin/carddisp.pl?gene=CRYAA
**MAFB**	MAF BZIP Transcription Factor B	Protein Coding	43	GC20M040685	3.47	https://www.genecards.org/cgi-bin/carddisp.pl?gene=MAFB
**SLC6A13**	Solute Carrier Family 6 Member 13	Protein Coding	43	GC12M000200	3.47	https://www.genecards.org/cgi-bin/carddisp.pl?gene=SLC6A13
**SLC25A6**	Solute Carrier Family 25 Member 6	Protein Coding	43	GC0XM001386	3.47	https://www.genecards.org/cgi-bin/carddisp.pl?gene=SLC25A6
**SLC26A4**	Solute Carrier Family 26 Member 4	Protein Coding	43	GC07P107660	3.47	https://www.genecards.org/cgi-bin/carddisp.pl?gene=SLC26A4
**SLC26A5**	Solute Carrier Family 26 Member 5	Protein Coding	43	GC07M103352	3.47	https://www.genecards.org/cgi-bin/carddisp.pl?gene=SLC26A5

**SLC39A13**	Solute Carrier Family 39 Member 13	Protein Coding	43	GC11P047430	3.47	https://www.genecards.org/cgi-bin/carddisp.pl?gene=SLC39A13
**LAMP1**	Lysosomal Associated Membrane Protein 1	Protein Coding	43	GC13P113297	3.47	https://www.genecards.org/cgi-bin/carddisp.pl?gene=LAMP1
**UGCG**	UDP-Glucose Ceramide Glucosyltransferase	Protein Coding	43	GC09P111896	3.47	https://www.genecards.org/cgi-bin/carddisp.pl?gene=UGCG
**CHST3**	Carbohydrate Sulfotransferase 3	Protein Coding	42	GC10P071964	3.47	https://www.genecards.org/cgi-bin/carddisp.pl?gene=CHST3
**PECR**	Peroxisomal Trans-2-Enoyl-CoA Reductase	Protein Coding	42	GC02M215996	3.47	https://www.genecards.org/cgi-bin/carddisp.pl?gene=PECR
**GBA2**	Glucosylceramidase Beta 2	Protein Coding	41	GC09M035726	3.47	https://www.genecards.org/cgi-bin/carddisp.pl?gene=GBA2
**CCT3**	Chaperonin Containing TCP1 Subunit 3	Protein Coding	41	GC01M156308	3.47	https://www.genecards.org/cgi-bin/carddisp.pl?gene=CCT3
**GLYAT**	Glycine-N-Acyltransferase	Protein Coding	41	GC11M061239	3.47	https://www.genecards.org/cgi-bin/carddisp.pl?gene=GLYAT

**ATG3**	Autophagy Related 3	Protein Coding	41	GC03M112532	3.47	https://www.genecards.org/cgi-bin/carddisp.pl?gene=ATG3
**CA3**	Carbonic Anhydrase 3	Protein Coding	41	GC08P085373	3.47	https://www.genecards.org/cgi-bin/carddisp.pl?gene=CA3
**MFAP5**	Microfibril Associated Protein 5	Protein Coding	41	GC12M008637	3.47	https://www.genecards.org/cgi-bin/carddisp.pl?gene=MFAP5
**SLC26A8**	Solute Carrier Family 26 Member 8	Protein Coding	41	GC06M042225	3.47	https://www.genecards.org/cgi-bin/carddisp.pl?gene=SLC26A8
**SLC6A11**	Solute Carrier Family 6 Member 11	Protein Coding	41	GC03P010858	3.47	https://www.genecards.org/cgi-bin/carddisp.pl?gene=SLC6A11
**HOXD10**	Homeobox D10	Protein Coding	40	GC02P176108	3.47	https://www.genecards.org/cgi-bin/carddisp.pl?gene=HOXD10
**SLC35D1**	Solute Carrier Family 35 Member D1	Protein Coding	40	GC01M066999	3.47	https://www.genecards.org/cgi-bin/carddisp.pl?gene=SLC35D1
**SLC26A1**	Solute Carrier Family 26 Member 1	Protein Coding	40	GC04M000979	3.47	https://www.genecards.org/cgi-bin/carddisp.pl?gene=SLC26A1

**SLC38A4**	Solute Carrier Family 38 Member 4	Protein Coding	40	GC12M046764	3.47	https://www.genecards.org/cgi-bin/carddisp.pl?gene=SLC38A4
**TSHZ1**	Teashirt Zinc Finger Homeobox 1	Protein Coding	40	GC18P075210	3.47	https://www.genecards.org/cgi-bin/carddisp.pl?gene=TSHZ1
**TMEM43**	Transmembrane Protein 43	Protein Coding	40	GC03P014124	3.47	https://www.genecards.org/cgi-bin/carddisp.pl?gene=TMEM43
**GGA3**	Golgi Associated, Gamma Adaptin Ear Containing, ARF Binding Protein 3	Protein Coding	39	GC17M075225	3.47	https://www.genecards.org/cgi-bin/carddisp.pl?gene=GGA3
**GGCT**	Gamma-Glutamylcyclotransferase	Protein Coding	39	GC07M030496	3.47	https://www.genecards.org/cgi-bin/carddisp.pl?gene=GGCT
**BLZF1**	Basic Leucine Zipper Nuclear Factor 1	Protein Coding	39	GC01P169367	3.47	https://www.genecards.org/cgi-bin/carddisp.pl?gene=BLZF1
**ACOT11**	Acyl-CoA Thioesterase 11	Protein Coding	39	GC01P054542	3.47	https://www.genecards.org/cgi-bin/carddisp.pl?gene=ACOT11
**HPS6**	HPS6 Biogenesis Of Lysosomal Organelles Complex 2 Subunit 3	Protein Coding	39	GC10P102065	3.47	https://www.genecards.org/cgi-bin/carddisp.pl?gene=HPS6

**MTX1**	Metaxin 1	Protein Coding	39	GC01P155208	3.47	https://www.genecards.org/cgi-bin/carddisp.pl?gene=MTX1
**SLC26A6**	Solute Carrier Family 26 Member 6	Protein Coding	39	GC03M048625	3.47	https://www.genecards.org/cgi-bin/carddisp.pl?gene=SLC26A6
**SRSF7**	Serine And Arginine Rich Splicing Factor 7	Protein Coding	39	GC02M038709	3.47	https://www.genecards.org/cgi-bin/carddisp.pl?gene=SRSF7
**TTC3**	Tetratricopeptide Repeat Domain 3	Protein Coding	39	GC21P037073	3.47	https://www.genecards.org/cgi-bin/carddisp.pl?gene=TTC3
**CCL1**	C-C Motif Chemokine Ligand 1	Protein Coding	38	GC17M034402	3.47	https://www.genecards.org/cgi-bin/carddisp.pl?gene=CCL1
**LZTS1**	Leucine Zipper Tumor Suppressor 1	Protein Coding	38	GC08M020246	3.47	https://www.genecards.org/cgi-bin/carddisp.pl?gene=LZTS1
**SLC7A3**	Solute Carrier Family 7 Member 3	Protein Coding	38	GC0XM070926	3.47	https://www.genecards.org/cgi-bin/carddisp.pl?gene=SLC7A3
**PLEKHA7**	Pleckstrin Homology Domain Containing A7	Protein Coding	38	GC11M016778	3.47	https://www.genecards.org/cgi-bin/carddisp.pl?gene=PLEKHA7

**SLC26A11**	Solute Carrier Family 26 Member 11	Protein Coding	38	GC17P080219	3.47	https://www.genecards.org/cgi-bin/carddisp.pl?gene=SLC26A11
**OTULIN**	OTU Deubiquitinase With Linear Linkage Specificity	Protein Coding	38	GC05P014667	3.47	https://www.genecards.org/cgi-bin/carddisp.pl?gene=OTULIN
**DOCK11**	Dedicator Of Cytokinesis 11	Protein Coding	37	GC0XP118496	3.47	https://www.genecards.org/cgi-bin/carddisp.pl?gene=DOCK11
**STARD10**	StAR Related Lipid Transfer Domain Containing 10	Protein Coding	37	GC11M072761	3.47	https://www.genecards.org/cgi-bin/carddisp.pl?gene=STARD10
**SYNJ2BP**	Synaptojanin 2 Binding Protein	Protein Coding	37	GC14M070366	3.47	https://www.genecards.org/cgi-bin/carddisp.pl?gene=SYNJ2BP
**FCRL3**	Fc Receptor Like 3	Protein Coding	36	GC01M157674	3.47	https://www.genecards.org/cgi-bin/carddisp.pl?gene=FCRL3
**SUGCT**	Succinyl-CoA:Glutarate-CoA Transferase	Protein Coding	36	GC07P040134	3.47	https://www.genecards.org/cgi-bin/carddisp.pl?gene=SUGCT
**PCMTD1**	Protein-L-Isoaspartate (D-Aspartate) O-Methyltransferase Domain Containing 1	Protein Coding	36	GC08M051817	3.47	https://www.genecards.org/cgi-bin/carddisp.pl?gene=PCMTD1

**CAGE1**	Cancer Antigen 1	Protein Coding	35	GC06M007326	3.47	https://www.genecards.org/cgi-bin/carddisp.pl?gene=CAGE1
**EMP1**	Epithelial Membrane Protein 1	Protein Coding	35	GC12P013196	3.47	https://www.genecards.org/cgi-bin/carddisp.pl?gene=EMP1
**ARSH**	Arylsulfatase Family Member H	Protein Coding	34	GC0XP003006	3.47	https://www.genecards.org/cgi-bin/carddisp.pl?gene=ARSH
**TAAR9**	Trace Amine Associated Receptor 9	Protein Coding	32	GC06P132538	3.47	https://www.genecards.org/cgi-bin/carddisp.pl?gene=TAAR9
**GGT2**	Gamma-Glutamyltransferase 2	Protein Coding	31	GC22M021207	3.47	https://www.genecards.org/cgi-bin/carddisp.pl?gene=GGT2
**EMSY**	EMSY Transcriptional Repressor, BRCA2 Interacting	Protein Coding	31	GC11P076445	3.47	https://www.genecards.org/cgi-bin/carddisp.pl?gene=EMSY
**OR1L1**	Olfactory Receptor Family 1 Subfamily L Member 1	Protein Coding	27	GC09P122661	3.47	https://www.genecards.org/cgi-bin/carddisp.pl?gene=OR1L1
**KRTAP10-1**	Keratin Associated Protein 10-1	Protein Coding	27	GC21M044538	3.47	https://www.genecards.org/cgi-bin/carddisp.pl?gene=KRTAP10-1

**MACIR**	Macrophage Immunometabolism Regulator	Protein Coding	25	GC05P103259	3.47	https://www.genecards.org/cgi-bin/carddisp.pl?gene=MACIR
**UCA1**	Urothelial Cancer Associated 1	RNA Gene	24	GC19P015828	3.47	https://www.genecards.org/cgi-bin/carddisp.pl?gene=UCA1
**MIR10B**	MicroRNA 10b	RNA Gene	21	GC02P176150	3.47	https://www.genecards.org/cgi-bin/carddisp.pl?gene=MIR10B
**MIR222**	MicroRNA 222	RNA Gene	21	GC0XM045747	3.47	https://www.genecards.org/cgi-bin/carddisp.pl?gene=MIR222
**GGTLC3**	Gamma-Glutamyltransferase Light Chain Family Member 3	Protein Coding	20	GC22M018516	3.47	https://www.genecards.org/cgi-bin/carddisp.pl?gene=GGTLC3
**MIR212**	MicroRNA 212	RNA Gene	19	GC17M002050	3.47	https://www.genecards.org/cgi-bin/carddisp.pl?gene=MIR212
**MIR103A1**	MicroRNA 103a-1	RNA Gene	17	GC05M168560	3.47	https://www.genecards.org/cgi-bin/carddisp.pl?gene=MIR103A1
**RNF217-AS1**	RNF217 Antisense RNA 1 (Head To Head)	RNA Gene	14	GC06M124563	3.47	https://www.genecards.org/cgi-bin/carddisp.pl?gene=RNF217-AS1

**SNORA3A**	Small Nucleolar RNA, H/ACA Box 3A	RNA Gene	14	GC11P008718	3.47	https://www.genecards.org/cgi-bin/carddisp.pl?gene=SNORA3A
**TMPO-AS1**	TMPO Antisense RNA 1	RNA Gene	13	GC12M098512	3.47	https://www.genecards.org/cgi-bin/carddisp.pl?gene=TMPO-AS1
**TGFB2-AS1**	TGFB2 Antisense RNA 1 (Head To Head)	RNA Gene	12	GC01M218344	3.47	https://www.genecards.org/cgi-bin/carddisp.pl?gene=TGFB2-AS1
**TGFB2-OT1**	TGFB2 Overlapping Transcript 1	RNA Gene	10	GC01P218442	3.47	https://www.genecards.org/cgi-bin/carddisp.pl?gene=TGFB2-OT1
**MIR6069**	MicroRNA 6069	RNA Gene	8	GC22M035336	3.47	https://www.genecards.org/cgi-bin/carddisp.pl?gene=MIR6069
**ENSG00000259039**		RNA Gene	6	GC14P057578	3.47	https://www.genecards.org/cgi-bin/carddisp.pl?gene=ENSG00000259039
**CHUK**	Component Of Inhibitor Of Nuclear Factor Kappa B Kinase Complex	Protein Coding	52	GC10M100188	3.43	https://www.genecards.org/cgi-bin/carddisp.pl?gene=CHUK
**CFLAR**	CASP8 And FADD Like Apoptosis Regulator	Protein Coding	46	GC02P201117	3.43	https://www.genecards.org/cgi-bin/carddisp.pl?gene=CFLAR

**GNL3**	G Protein Nucleolar 3	Protein Coding	40	GC03P052681	3.39	https://www.genecards.org/cgi-bin/carddisp.pl?gene=GNL3
**CA10**	Carbonic Anhydrase 10	Protein Coding	39	GC17M051630	3.37	https://www.genecards.org/cgi-bin/carddisp.pl?gene=CA10
**CSK**	C-Terminal Src Kinase	Protein Coding	48	GC15P074782	3.37	https://www.genecards.org/cgi-bin/carddisp.pl?gene=CSK
**ATP6V0A2**	ATPase H+ Transporting V0 Subunit A2	Protein Coding	44	GC12P123712	3.37	https://www.genecards.org/cgi-bin/carddisp.pl?gene=ATP6V0A2
**CRBN**	Cereblon	Protein Coding	43	GC03M003144	3.37	https://www.genecards.org/cgi-bin/carddisp.pl?gene=CRBN
**HLA-DPB1**	Major Histocompatibility Complex, Class II, DP Beta 1	Protein Coding	43	GC06P047346	3.37	https://www.genecards.org/cgi-bin/carddisp.pl?gene=HLA-DPB1
**MAEA**	Macrophage Erythroblast Attacher, E3 Ubiquitin Ligase	Protein Coding	38	GC04P001289	3.37	https://www.genecards.org/cgi-bin/carddisp.pl?gene=MAEA
**SLBP**	Stem-Loop Binding Protein	Protein Coding	35	GC04M001692	3.37	https://www.genecards.org/cgi-bin/carddisp.pl?gene=SLBP

**NSD2**	Nuclear Receptor Binding SET Domain Protein 2	Protein Coding	35	GC04P001 872	3.37	https://www.genecards.org/cgi-bin/carddisp.pl?gene=NSD2
**USP50**	Ubiquitin Specific Peptidase 50	Protein Coding	33	GC15M050 496	3.37	https://www.genecards.org/cgi-bin/carddisp.pl?gene=USP50
**MMP10**	Matrix Metallopeptidase 10	Protein Coding	46	GC11M102 770	3.34	https://www.genecards.org/cgi-bin/carddisp.pl?gene=MMP10
**CENPP**	Centromere Protein P	Protein Coding	35	GC09P092 325	3.32	https://www.genecards.org/cgi-bin/carddisp.pl?gene=CENPP
**LOC10192822 2**	Uncharacterized LOC101928222	RNA Gene	8	GC02P019 990	3.32	https://www.genecards.org/cgi-bin/carddisp.pl?gene=LOC101928222
**HDAC1**	Histone Deacetylase 1	Protein Coding	49	GC01P032 292	3.29	https://www.genecards.org/cgi-bin/carddisp.pl?gene=HDAC1
**BGN**	Biglycan	Protein Coding	43	GC0XP153 494	3.26	https://www.genecards.org/cgi-bin/carddisp.pl?gene=BGN
**NAMPT**	Nicotinamide Phosphoribosyltransferase	Protein Coding	48	GC07M106 248	3.25	https://www.genecards.org/cgi-bin/carddisp.pl?gene=NAMPT

**COL6A4P1**	Collagen Type VI Alpha 4 Pseudogene 1	Pseudogene	12	GC03M015151	3.22	https://www.genecards.org/cgi-bin/carddisp.pl?gene=COL6A4P1
**KDR**	Kinase Insert Domain Receptor	Protein Coding	53	GC04M055078	3.22	https://www.genecards.org/cgi-bin/carddisp.pl?gene=KDR
**TNFRSF10B**	TNF Receptor Superfamily Member 10b	Protein Coding	50	GC08M023020	3.22	https://www.genecards.org/cgi-bin/carddisp.pl?gene=TNFRSF10B
**ITGA4**	Integrin Subunit Alpha 4	Protein Coding	48	GC02P181456	3.19	https://www.genecards.org/cgi-bin/carddisp.pl?gene=ITGA4
**ENO1**	Enolase 1	Protein Coding	47	GC01M008861	3.19	https://www.genecards.org/cgi-bin/carddisp.pl?gene=ENO1
**MAP2K7**	Mitogen-Activated Protein Kinase Kinase 7	Protein Coding	45	GC19P007903	3.19	https://www.genecards.org/cgi-bin/carddisp.pl?gene=MAP2K7
**DOT1L**	DOT1 Like Histone Lysine Methyltransferase	Protein Coding	44	GC19P002164	3.18	https://www.genecards.org/cgi-bin/carddisp.pl?gene=DOT1L
**CTSG**	Cathepsin G	Protein Coding	44	GC14M024573	3.16	https://www.genecards.org/cgi-bin/carddisp.pl?gene=CTSG

**STAT1**	Signal Transducer And Activator Of Transcription 1	Protein Coding	53	GC02M190908	3.15	https://www.genecards.org/cgi-bin/carddisp.pl?gene=STAT1
**CFI**	Complement Factor I	Protein Coding	46	GC04M109740	3.15	https://www.genecards.org/cgi-bin/carddisp.pl?gene=CFI
**TNFSF10**	TNF Superfamily Member 10	Protein Coding	46	GC03M172505	3.15	https://www.genecards.org/cgi-bin/carddisp.pl?gene=TNFSF10
**VTN**	Vitronectin	Protein Coding	44	GC17M029955	3.15	https://www.genecards.org/cgi-bin/carddisp.pl?gene=VTN
**DNMT1**	DNA Methyltransferase 1	Protein Coding	50	GC19M010133	3.11	https://www.genecards.org/cgi-bin/carddisp.pl?gene=DNMT1
**SELP**	Selectin P	Protein Coding	45	GC01M169558	3.11	https://www.genecards.org/cgi-bin/carddisp.pl?gene=SELP
**NT5E**	5'-Nucleotidase Ecto	Protein Coding	51	GC06P085449	3.1	https://www.genecards.org/cgi-bin/carddisp.pl?gene=NT5E
**CX3CR1**	C-X3-C Motif Chemokine Receptor 1	Protein Coding	44	GC03M039279	3.1	https://www.genecards.org/cgi-bin/carddisp.pl?gene=CX3CR1
**ERBB2**	Erb-B2 Receptor Tyrosine Kinase 2	Protein Coding	54	GC17P039687	3.07	https://www.genecards.org/cgi-bin/carddisp.pl?gene=ERBB2

**INS**	Insulin	Protein Coding	48	GC11M002159	3.07	https://www.genecards.org/cgi-bin/carddisp.pl?gene=INS
**JAM3**	Junctional Adhesion Molecule 3	Protein Coding	43	GC11P134068	3.07	https://www.genecards.org/cgi-bin/carddisp.pl?gene=JAM3
**IL18BP**	Interleukin 18 Binding Protein	Protein Coding	39	GC11P071998	3.07	https://www.genecards.org/cgi-bin/carddisp.pl?gene=IL18BP
**NOG**	Noggin	Protein Coding	47	GC17P056593	3.05	https://www.genecards.org/cgi-bin/carddisp.pl?gene=NOG
**THBD**	Thrombomodulin	Protein Coding	44	GC20M023026	3.05	https://www.genecards.org/cgi-bin/carddisp.pl?gene=THBD
**MAPK3**	Mitogen-Activated Protein Kinase 3	Protein Coding	49	GC16M030117	3.02	https://www.genecards.org/cgi-bin/carddisp.pl?gene=MAPK3
**HSD11B1**	Hydroxysteroid 11-Beta Dehydrogenase 1	Protein Coding	49	GC01P209686	3.02	https://www.genecards.org/cgi-bin/carddisp.pl?gene=HSD11B1
**PDCD1**	Programmed Cell Death 1	Protein Coding	48	GC02M241849	3.02	https://www.genecards.org/cgi-bin/carddisp.pl?gene=PDCD1

**LRP1**	LDL Receptor Related Protein 1	Protein Coding	47	GC12P057128	3.02	https://www.genecards.org/cgi-bin/carddisp.pl?gene=LRP1
**GPI**	Glucose-6-Phosphate Isomerase	Protein Coding	46	GC19P034359	3.02	https://www.genecards.org/cgi-bin/carddisp.pl?gene=GPI
**PPARA**	Peroxisome Proliferator Activated Receptor Alpha	Protein Coding	45	GC22P046150	3.02	https://www.genecards.org/cgi-bin/carddisp.pl?gene=PPARA
**CARD16**	Caspase Recruitment Domain Family Member 16	Protein Coding	36	GC11M105041	3.02	https://www.genecards.org/cgi-bin/carddisp.pl?gene=CARD16
**MIR17**	MicroRNA 17	RNA Gene	21	GC13P091350	3.02	https://www.genecards.org/cgi-bin/carddisp.pl?gene=MIR17
**MIR199A1**	MicroRNA 199a-1	RNA Gene	18	GC19M010792	3.02	https://www.genecards.org/cgi-bin/carddisp.pl?gene=MIR199A1
**CCN2**	Cellular Communication Network Factor 2	Protein Coding	39	GC06M131948	3.02	https://www.genecards.org/cgi-bin/carddisp.pl?gene=CCN2
**TGM2**	Transglutaminase 2	Protein Coding	48	GC20M038127	2.99	https://www.genecards.org/cgi-bin/carddisp.pl?gene=TGM2

**BCL2L1**	BCL2 Like 1	Protein Coding	47	GC20M031664	2.99	https://www.genecards.org/cgi-bin/carddisp.pl?gene=BCL2L1
**THY1**	Thy-1 Cell Surface Antigen	Protein Coding	44	GC11M119417	2.97	https://www.genecards.org/cgi-bin/carddisp.pl?gene=THY1
**PYCARD**	PYD And CARD Domain Containing	Protein Coding	43	GC16M031201	2.97	https://www.genecards.org/cgi-bin/carddisp.pl?gene=PYCARD
**RECK**	Reversion Inducing Cysteine Rich Protein With Kazal Motifs	Protein Coding	40	GC09P036036	2.97	https://www.genecards.org/cgi-bin/carddisp.pl?gene=RECK
**F2RL1**	F2R Like Trypsin Receptor 1	Protein Coding	45	GC05P076818	2.97	https://www.genecards.org/cgi-bin/carddisp.pl?gene=F2RL1
**TGFA**	Transforming Growth Factor Alpha	Protein Coding	46	GC02M070447	2.96	https://www.genecards.org/cgi-bin/carddisp.pl?gene=TGFA
**CASP9**	Caspase 9	Protein Coding	48	GC01M015491	2.95	https://www.genecards.org/cgi-bin/carddisp.pl?gene=CASP9
**IL2RB**	Interleukin 2 Receptor Subunit Beta	Protein Coding	48	GC22M037125	2.95	https://www.genecards.org/cgi-bin/carddisp.pl?gene=IL2RB

**CCK**	Cholecystokinin	Protein Coding	41	GC03M042274	2.95	https://www.genecards.org/cgi-bin/carddisp.pl?gene=CCK
**TREM1**	Triggering Receptor Expressed On Myeloid Cells 1	Protein Coding	41	GC06M041267	2.95	https://www.genecards.org/cgi-bin/carddisp.pl?gene=TREM1
**NFKB1**	Nuclear Factor Kappa B Subunit 1	Protein Coding	52	GC04P102501	2.95	https://www.genecards.org/cgi-bin/carddisp.pl?gene=NFKB1
**ESR2**	Estrogen Receptor 2	Protein Coding	49	GC14M064084	2.95	https://www.genecards.org/cgi-bin/carddisp.pl?gene=ESR2
**IL17F**	Interleukin 17F	Protein Coding	41	GC06M052209	2.93	https://www.genecards.org/cgi-bin/carddisp.pl?gene=IL17F
**JAK2**	Janus Kinase 2	Protein Coding	54	GC09P004985	2.91	https://www.genecards.org/cgi-bin/carddisp.pl?gene=JAK2
**APP**	Amyloid Beta Precursor Protein	Protein Coding	51	GC21M025880	2.91	https://www.genecards.org/cgi-bin/carddisp.pl?gene=APP
**SYK**	Spleen Associated Tyrosine Kinase	Protein Coding	50	GC09P091171	2.91	https://www.genecards.org/cgi-bin/carddisp.pl?gene=SYK
**LPL**	Lipoprotein Lipase	Protein Coding	49	GC08P019901	2.91	https://www.genecards.org/cgi-bin/carddisp.pl?gene=LPL

**GRB2**	Growth Factor Receptor Bound Protein 2	Protein Coding	49	GC17M075318	2.91	https://www.genecards.org/cgi-bin/carddisp.pl?gene=GRB2
**ITGAL**	Integrin Subunit Alpha L	Protein Coding	45	GC16P030472	2.91	https://www.genecards.org/cgi-bin/carddisp.pl?gene=ITGAL
**GHRL**	Ghrelin And Obestatin Prepropeptide	Protein Coding	43	GC03M010285	2.91	https://www.genecards.org/cgi-bin/carddisp.pl?gene=GHRL
**IL19**	Interleukin 19	Protein Coding	40	GC01P206770	2.91	https://www.genecards.org/cgi-bin/carddisp.pl?gene=IL19
**IL22RA1**	Interleukin 22 Receptor Subunit Alpha 1	Protein Coding	39	GC01M024119	2.91	https://www.genecards.org/cgi-bin/carddisp.pl?gene=IL22RA1
**AGTR1**	Angiotensin II Receptor Type 1	Protein Coding	51	GC03P148697	2.86	https://www.genecards.org/cgi-bin/carddisp.pl?gene=AGTR1
**ALK**	ALK Receptor Tyrosine Kinase	Protein Coding	51	GC02M029156	2.86	https://www.genecards.org/cgi-bin/carddisp.pl?gene=ALK
**ABCB1**	ATP Binding Cassette Subfamily B Member 1	Protein Coding	51	GC07M087504	2.86	https://www.genecards.org/cgi-bin/carddisp.pl?gene=ABCB1
**JAK1**	Janus Kinase 1	Protein Coding	50	GC01M064833	2.86	https://www.genecards.org/cgi-bin/carddisp.pl?gene=JAK1

**TF**	Transferrin	Protein Coding	49	GC03P133666	2.86	https://www.genecards.org/cgi-bin/carddisp.pl?gene=TF
**MAP3K8**	Mitogen-Activated Protein Kinase Kinase Kinase 8	Protein Coding	47	GC10P030458	2.86	https://www.genecards.org/cgi-bin/carddisp.pl?gene=MAP3K8
**FCGR2B**	Fc Fragment Of IgG Receptor IIb	Protein Coding	47	GC01P161663	2.86	https://www.genecards.org/cgi-bin/carddisp.pl?gene=FCGR2B
**HTR2A**	5-Hydroxytryptamine Receptor 2A	Protein Coding	47	GC13M046831	2.86	https://www.genecards.org/cgi-bin/carddisp.pl?gene=HTR2A
**BMP1**	Bone Morphogenetic Protein 1	Protein Coding	45	GC08P022164	2.86	https://www.genecards.org/cgi-bin/carddisp.pl?gene=BMP1
**PRF1**	Perforin 1	Protein Coding	45	GC10M070597	2.86	https://www.genecards.org/cgi-bin/carddisp.pl?gene=PRF1
**USP14**	Ubiquitin Specific Peptidase 14	Protein Coding	45	GC18P000158	2.86	https://www.genecards.org/cgi-bin/carddisp.pl?gene=USP14
**GNS**	Glucosamine (N-Acetyl)-6-Sulfatase	Protein Coding	44	GC12M064713	2.86	https://www.genecards.org/cgi-bin/carddisp.pl?gene=GNS
**GAL**	Galanin And GMAP Prepropeptide	Protein Coding	44	GC11P068684	2.86	https://www.genecards.org/cgi-bin/carddisp.pl?gene=GAL

**CD274**	CD274 Molecule	Protein Coding	44	GC09P005450	2.86	https://www.genecards.org/cgi-bin/carddisp.pl?gene=CD274
**SNAI1**	Snail Family Transcriptional Repressor 1	Protein Coding	44	GC20P049982	2.86	https://www.genecards.org/cgi-bin/carddisp.pl?gene=SNAI1
**TNFRSF17**	TNF Receptor Superfamily Member 17	Protein Coding	44	GC16P011965	2.86	https://www.genecards.org/cgi-bin/carddisp.pl?gene=TNFRSF17
**FGF7**	Fibroblast Growth Factor 7	Protein Coding	41	GC15P049423	2.86	https://www.genecards.org/cgi-bin/carddisp.pl?gene=FGF7
**P2RX3**	Purinergic Receptor P2X 3	Protein Coding	41	GC11P057356	2.86	https://www.genecards.org/cgi-bin/carddisp.pl?gene=P2RX3
**SRF**	Serum Response Factor	Protein Coding	41	GC06P043171	2.86	https://www.genecards.org/cgi-bin/carddisp.pl?gene=SRF
**PRKN**	Parkin RBR E3 Ubiquitin Protein Ligase	Protein Coding	40	GC06M161348	2.86	https://www.genecards.org/cgi-bin/carddisp.pl?gene=PRKN
**SHBG**	Sex Hormone Binding Globulin	Protein Coding	40	GC17P007613	2.86	https://www.genecards.org/cgi-bin/carddisp.pl?gene=SHBG

**LECT2**	Leukocyte Cell Derived Chemotaxin 2	Protein Coding	36	GC05M135922	2.86	https://www.genecards.org/cgi-bin/carddisp.pl?gene=LECT2
**IL26**	Interleukin 26	Protein Coding	34	GC12M068201	2.86	https://www.genecards.org/cgi-bin/carddisp.pl?gene=IL26
**KIR2DS4**	Killer Cell Immunoglobulin Like Receptor, Two Ig Domains And Short Cytoplasmic Tail 4	Protein Coding	32	GC19P055443	2.86	https://www.genecards.org/cgi-bin/carddisp.pl?gene=KIR2DS4
**MIR142**	MicroRNA 142	RNA Gene	20	GC17M058331	2.86	https://www.genecards.org/cgi-bin/carddisp.pl?gene=MIR142
**ALDH1A2**	Aldehyde Dehydrogenase 1 Family Member A2	Protein Coding	47	GC15M060613	2.85	https://www.genecards.org/cgi-bin/carddisp.pl?gene=ALDH1A2
**GLT8D1**	Glycosyltransferase 8 Domain Containing 1	Protein Coding	38	GC03M052704	2.76	https://www.genecards.org/cgi-bin/carddisp.pl?gene=GLT8D1
**TNC**	Tenascin C	Protein Coding	48	GC09M115019	2.75	https://www.genecards.org/cgi-bin/carddisp.pl?gene=TNC
**SUPT3H**	SPT3 Homolog, SAGA And STAGA Complex Component	Protein Coding	40	GC06M044807	2.74	https://www.genecards.org/cgi-bin/carddisp.pl?gene=SUPT3H

**NCOA3**	Nuclear Receptor Coactivator 3	Protein Coding	45	GC20P047501	2.73	https://www.genecards.org/cgi-bin/carddisp.pl?gene=NCOA3
**FGA**	Fibrinogen Alpha Chain	Protein Coding	47	GC04M154583	2.73	https://www.genecards.org/cgi-bin/carddisp.pl?gene=FGA
**MBL2**	Mannose Binding Lectin 2	Protein Coding	47	GC10M052760	2.73	https://www.genecards.org/cgi-bin/carddisp.pl?gene=MBL2
**SMPD1**	Sphingomyelin Phosphodiesterase 1	Protein Coding	47	GC11P006390	2.73	https://www.genecards.org/cgi-bin/carddisp.pl?gene=SMPD1
**LBR**	Lamin B Receptor	Protein Coding	47	GC01M225401	2.73	https://www.genecards.org/cgi-bin/carddisp.pl?gene=LBR
**F5**	Coagulation Factor V	Protein Coding	45	GC01M169511	2.73	https://www.genecards.org/cgi-bin/carddisp.pl?gene=F5
**IGF2R**	Insulin Like Growth Factor 2 Receptor	Protein Coding	45	GC06P159969	2.73	https://www.genecards.org/cgi-bin/carddisp.pl?gene=IGF2R
**GZMB**	Granzyme B	Protein Coding	45	GC14M024630	2.73	https://www.genecards.org/cgi-bin/carddisp.pl?gene=GZMB
**FGF3**	Fibroblast Growth Factor 3	Protein Coding	44	GC11M069811	2.73	https://www.genecards.org/cgi-bin/carddisp.pl?gene=FGF3

**GDF6**	Growth Differentiation Factor 6	Protein Coding	43	GC08M096142	2.73	https://www.genecards.org/cgi-bin/carddisp.pl?gene=GDF6
**CD34**	CD34 Molecule	Protein Coding	43	GC01M207880	2.73	https://www.genecards.org/cgi-bin/carddisp.pl?gene=CD34
**EPO**	Erythropoietin	Protein Coding	41	GC07P100720	2.73	https://www.genecards.org/cgi-bin/carddisp.pl?gene=EPO
**DDB1**	Damage Specific DNA Binding Protein 1	Protein Coding	41	GC11M061563	2.73	https://www.genecards.org/cgi-bin/carddisp.pl?gene=DDB1
**SP7**	Sp7 Transcription Factor	Protein Coding	41	GC12M053326	2.73	https://www.genecards.org/cgi-bin/carddisp.pl?gene=SP7
**SIGLEC1**	Sialic Acid Binding Ig Like Lectin 1	Protein Coding	40	GC20M003686	2.73	https://www.genecards.org/cgi-bin/carddisp.pl?gene=SIGLEC1
**PRRC2A**	Proline Rich Coiled-Coil 2A	Protein Coding	33	GC06P047307	2.73	https://www.genecards.org/cgi-bin/carddisp.pl?gene=PRRC2A
**GGT3P**	Gamma-Glutamyltransferase 3 Pseudogene	Pseudogene	18	GC22M018773	2.73	https://www.genecards.org/cgi-bin/carddisp.pl?gene=GGT3P
**MDM2**	MDM2 Proto-Oncogene	Protein Coding	52	GC12P068808	2.68	https://www.genecards.org/cgi-bin/carddisp.pl?gene=MDM2

**EPAS1**	Endothelial PAS Domain Protein 1	Protein Coding	48	GC02P046293	2.66	https://www.genecards.org/cgi-bin/carddisp.pl?gene=EPAS1
**KIT**	KIT Proto-Oncogene, Receptor Tyrosine Kinase	Protein Coding	53	GC04P054657	2.64	https://www.genecards.org/cgi-bin/carddisp.pl?gene=KIT
**NR1H4**	Nuclear Receptor Subfamily 1 Group H Member 4	Protein Coding	48	GC12P100473	2.64	https://www.genecards.org/cgi-bin/carddisp.pl?gene=NR1H4
**LRP2**	LDL Receptor Related Protein 2	Protein Coding	45	GC02M169127	2.64	https://www.genecards.org/cgi-bin/carddisp.pl?gene=LRP2
**CDK1**	Cyclin Dependent Kinase 1	Protein Coding	45	GC10P060772	2.64	https://www.genecards.org/cgi-bin/carddisp.pl?gene=CDK1
**SERPINF1**	Serpin Family F Member 1	Protein Coding	44	GC17P001761	2.64	https://www.genecards.org/cgi-bin/carddisp.pl?gene=SERPINF1
**PF4**	Platelet Factor 4	Protein Coding	41	GC04M073980	2.64	https://www.genecards.org/cgi-bin/carddisp.pl?gene=PF4
**TNIP1**	TNFAIP3 Interacting Protein 1	Protein Coding	41	GC05M151029	2.64	https://www.genecards.org/cgi-bin/carddisp.pl?gene=TNIP1
**ARL13B**	ADP Ribosylation Factor Like GTPase 13B	Protein Coding	37	GC03P093980	2.64	https://www.genecards.org/cgi-bin/carddisp.pl?gene=ARL13B

**MIR126**	MicroRNA 126	RNA Gene	22	GC09P136670	2.64	https://www.genecards.org/cgi-bin/carddisp.pl?gene=MIR126
**EGFR**	Epidermal Growth Factor Receptor	Protein Coding	54	GC07P055019	2.59	https://www.genecards.org/cgi-bin/carddisp.pl?gene=EGFR
**CDK2**	Cyclin Dependent Kinase 2	Protein Coding	52	GC12P055966	2.59	https://www.genecards.org/cgi-bin/carddisp.pl?gene=CDK2
**HNF4A**	Hepatocyte Nuclear Factor 4 Alpha	Protein Coding	50	GC20P044355	2.59	https://www.genecards.org/cgi-bin/carddisp.pl?gene=HNF4A
**CYLD**	CYLD Lysine 63 Deubiquitinase	Protein Coding	48	GC16P050742	2.59	https://www.genecards.org/cgi-bin/carddisp.pl?gene=CYLD
**IRAK3**	Interleukin 1 Receptor Associated Kinase 3	Protein Coding	48	GC12P066188	2.59	https://www.genecards.org/cgi-bin/carddisp.pl?gene=IRAK3
**EIF2AK3**	Eukaryotic Translation Initiation Factor 2 Alpha Kinase 3	Protein Coding	48	GC02M088637	2.59	https://www.genecards.org/cgi-bin/carddisp.pl?gene=EIF2AK3
**LMNB1**	Lamin B1	Protein Coding	47	GC05P126776	2.59	https://www.genecards.org/cgi-bin/carddisp.pl?gene=LMNB1

**FCGR2A**	Fc Fragment Of IgG Receptor IIa	Protein Coding	45	GC01P161505	2.59	https://www.genecards.org/cgi-bin/carddisp.pl?gene=FCGR2A
**HELLS**	Helicase, Lymphoid Specific	Protein Coding	45	GC10P094501	2.59	https://www.genecards.org/cgi-bin/carddisp.pl?gene=HELLS
**WRN**	WRN RecQ Like Helicase	Protein Coding	45	GC08P031033	2.59	https://www.genecards.org/cgi-bin/carddisp.pl?gene=WRN
**ESD**	Esterase D	Protein Coding	44	GC13M046771	2.59	https://www.genecards.org/cgi-bin/carddisp.pl?gene=ESD
**CPB2**	Carboxypeptidase B2	Protein Coding	44	GC13M046053	2.59	https://www.genecards.org/cgi-bin/carddisp.pl?gene=CPB2
**SERPINF2**	Serpin Family F Member 2	Protein Coding	44	GC17P001742	2.59	https://www.genecards.org/cgi-bin/carddisp.pl?gene=SERPINF2
**CHIA**	Chitinase Acidic	Protein Coding	42	GC01P111291	2.59	https://www.genecards.org/cgi-bin/carddisp.pl?gene=CHIA
**BST1**	Bone Marrow Stromal Cell Antigen 1	Protein Coding	41	GC04P015704	2.59	https://www.genecards.org/cgi-bin/carddisp.pl?gene=BST1

**BRD3**	Bromodomain Containing 3	Protein Coding	41	GC09M134030	2.59	https://www.genecards.org/cgi-bin/carddisp.pl?gene=BRD3
**FLG**	Filaggrin	Protein Coding	40	GC01M152274	2.59	https://www.genecards.org/cgi-bin/carddisp.pl?gene=FLG
**LTB**	Lymphotoxin Beta	Protein Coding	40	GC06M032590	2.59	https://www.genecards.org/cgi-bin/carddisp.pl?gene=LTB
**CTRL**	Chymotrypsin Like	Protein Coding	40	GC16M067927	2.59	https://www.genecards.org/cgi-bin/carddisp.pl?gene=CTRL
**NPPC**	Natriuretic Peptide C	Protein Coding	40	GC02M231921	2.59	https://www.genecards.org/cgi-bin/carddisp.pl?gene=NPPC
**HLA-DRB5**	Major Histocompatibility Complex, Class II, DR Beta 5	Protein Coding	40	GC06M032683	2.59	https://www.genecards.org/cgi-bin/carddisp.pl?gene=HLA-DRB5
**GPBAR1**	G Protein-Coupled Bile Acid Receptor 1	Protein Coding	39	GC02P218259	2.59	https://www.genecards.org/cgi-bin/carddisp.pl?gene=GPBAR1
**BTLA**	B And T Lymphocyte Associated	Protein Coding	39	GC03M112463	2.59	https://www.genecards.org/cgi-bin/carddisp.pl?gene=BTLA
**IFNA1**	Interferon Alpha 1	Protein Coding	39	GC09P021494	2.59	https://www.genecards.org/cgi-bin/carddisp.pl?gene=IFNA1

**CASC3**	CASC3 Exon Junction Complex Subunit	Protein Coding	35	GC17P040140	2.59	https://www.genecards.org/cgi-bin/carddisp.pl?gene=CASC3
**MIR186**	MicroRNA 186	RNA Gene	18	GC01M071067	2.59	https://www.genecards.org/cgi-bin/carddisp.pl?gene=MIR186
**CALM1**	Calmodulin 1	Protein Coding	45	GC14P090396	2.56	https://www.genecards.org/cgi-bin/carddisp.pl?gene=CALM1
**AGER**	Advanced Glycosylation End-Product Specific Receptor	Protein Coding	44	GC06M032180	2.55	https://www.genecards.org/cgi-bin/carddisp.pl?gene=AGER
**BAX**	BCL2 Associated X, Apoptosis Regulator	Protein Coding	48	GC19P048954	2.54	https://www.genecards.org/cgi-bin/carddisp.pl?gene=BAX
**PLA2G4A**	Phospholipase A2 Group IVA	Protein Coding	49	GC01P186798	2.46	https://www.genecards.org/cgi-bin/carddisp.pl?gene=PLA2G4A
**CDK6**	Cyclin Dependent Kinase 6	Protein Coding	54	GC07M092604	2.46	https://www.genecards.org/cgi-bin/carddisp.pl?gene=CDK6
**BTK**	Bruton Tyrosine Kinase	Protein Coding	53	GC0XM101349	2.46	https://www.genecards.org/cgi-bin/carddisp.pl?gene=BTK
**FLT3**	Fms Related Receptor Tyrosine Kinase 3	Protein Coding	52	GC13M028003	2.46	https://www.genecards.org/cgi-bin/carddisp.pl?gene=FLT3

**ABL1**	ABL Proto-Oncogene 1, Non-Receptor Tyrosine Kinase	Protein Coding	52	GC09P130713	2.46	https://www.genecards.org/cgi-bin/carddisp.pl?gene=ABL1
**ACVR1**	Activin A Receptor Type 1	Protein Coding	51	GC02M157736	2.46	https://www.genecards.org/cgi-bin/carddisp.pl?gene=ACVR1
**FGFR4**	Fibroblast Growth Factor Receptor 4	Protein Coding	51	GC05P177086	2.46	https://www.genecards.org/cgi-bin/carddisp.pl?gene=FGFR4
**HDAC6**	Histone Deacetylase 6	Protein Coding	51	GC0XP048801	2.46	https://www.genecards.org/cgi-bin/carddisp.pl?gene=HDAC6
**CCND2**	Cyclin D2	Protein Coding	50	GC12P008103	2.46	https://www.genecards.org/cgi-bin/carddisp.pl?gene=CCND2
**MME**	Membrane Metalloendopeptidase	Protein Coding	50	GC03P155024	2.46	https://www.genecards.org/cgi-bin/carddisp.pl?gene=MME
**GRM1**	Glutamate Metabotropic Receptor 1	Protein Coding	50	GC06P145973	2.46	https://www.genecards.org/cgi-bin/carddisp.pl?gene=GRM1
**DBH**	Dopamine Beta-Hydroxylase	Protein Coding	50	GC09P133636	2.46	https://www.genecards.org/cgi-bin/carddisp.pl?gene=DBH
**PPIB**	Peptidylprolyl Isomerase B	Protein Coding	50	GC15M064155	2.46	https://www.genecards.org/cgi-bin/carddisp.pl?gene=PPIB

**ROCK1**	Rho Associated Coiled-Coil Containing Protein Kinase 1	Protein Coding	50	GC18M020946	2.46	https://www.genecards.org/cgi-bin/carddisp.pl?gene=ROCK1
**FLNA**	Filamin A	Protein Coding	49	GC0XM154348	2.46	https://www.genecards.org/cgi-bin/carddisp.pl?gene=FLNA
**BUB1B**	BUB1 Mitotic Checkpoint Serine/Threonine Kinase B	Protein Coding	49	GC15P040161	2.46	https://www.genecards.org/cgi-bin/carddisp.pl?gene=BUB1B
**RIPK1**	Receptor Interacting Serine/Threonine Kinase 1	Protein Coding	49	GC06P003064	2.46	https://www.genecards.org/cgi-bin/carddisp.pl?gene=RIPK1
**GNAQ**	G Protein Subunit Alpha Q	Protein Coding	49	GC09M077716	2.46	https://www.genecards.org/cgi-bin/carddisp.pl?gene=GNAQ
**PBX1**	PBX Homeobox 1	Protein Coding	49	GC01P164524	2.46	https://www.genecards.org/cgi-bin/carddisp.pl?gene=PBX1
**PRKDC**	Protein Kinase, DNA-Activated, Catalytic Subunit	Protein Coding	49	GC08M047773	2.46	https://www.genecards.org/cgi-bin/carddisp.pl?gene=PRKDC
**RPS6KB1**	Ribosomal Protein S6 Kinase B1	Protein Coding	49	GC17P059893	2.46	https://www.genecards.org/cgi-bin/carddisp.pl?gene=RPS6KB1

**CD19**	CD19 Molecule	Protein Coding	49	GC16P029083	2.46	https://www.genecards.org/cgi-bin/carddisp.pl?gene=CD19
**KCNQ1**	Potassium Voltage-Gated Channel Subfamily Q Member 1	Protein Coding	49	GC11P002444	2.46	https://www.genecards.org/cgi-bin/carddisp.pl?gene=KCNQ1
**CBS**	Cystathionine Beta-Synthase	Protein Coding	48	GC21M043053	2.46	https://www.genecards.org/cgi-bin/carddisp.pl?gene=CBS
**F10**	Coagulation Factor X	Protein Coding	48	GC13P113122	2.46	https://www.genecards.org/cgi-bin/carddisp.pl?gene=F10
**ASS1**	Argininosuccinate Synthase 1	Protein Coding	48	GC09P130444	2.46	https://www.genecards.org/cgi-bin/carddisp.pl?gene=ASS1
**MVK**	Mevalonate Kinase	Protein Coding	48	GC12P109573	2.46	https://www.genecards.org/cgi-bin/carddisp.pl?gene=MVK
**GRIN2D**	Glutamate Ionotropic Receptor NMDA Type Subunit 2D	Protein Coding	48	GC19P048394	2.46	https://www.genecards.org/cgi-bin/carddisp.pl?gene=GRIN2D
**IKBKG**	Inhibitor Of Nuclear Factor Kappa B Kinase Regulatory Subunit Gamma	Protein Coding	48	GC0XP154541	2.46	https://www.genecards.org/cgi-bin/carddisp.pl?gene=IKBKG
**PAH**	Phenylalanine Hydroxylase	Protein Coding	48	GC12M102836	2.46	https://www.genecards.org/cgi-bin/carddisp.pl?gene=PAH

**PRLR**	Prolactin Receptor	Protein Coding	48	GC05M035048	2.46	https://www.genecards.org/cgi-bin/carddisp.pl?gene=PRLR
**SIRT2**	Sirtuin 2	Protein Coding	48	GC19M038878	2.46	https://www.genecards.org/cgi-bin/carddisp.pl?gene=SIRT2
**SIRT3**	Sirtuin 3	Protein Coding	48	GC11M000215	2.46	https://www.genecards.org/cgi-bin/carddisp.pl?gene=SIRT3
**GHSR**	Growth Hormone Secretagogue Receptor	Protein Coding	47	GC03M172443	2.46	https://www.genecards.org/cgi-bin/carddisp.pl?gene=GHSR
**GJB2**	Gap Junction Protein Beta 2	Protein Coding	47	GC13M020187	2.46	https://www.genecards.org/cgi-bin/carddisp.pl?gene=GJB2
**GK**	Glycerol Kinase	Protein Coding	47	GC0XP030671	2.46	https://www.genecards.org/cgi-bin/carddisp.pl?gene=GK
**FAH**	Fumarylacetoacetate Hydrolase	Protein Coding	47	GC15P080152	2.46	https://www.genecards.org/cgi-bin/carddisp.pl?gene=FAH
**AK1**	Adenylate Kinase 1	Protein Coding	47	GC09M127866	2.46	https://www.genecards.org/cgi-bin/carddisp.pl?gene=AK1
**MCL1**	MCL1 Apoptosis Regulator, BCL2 Family Member	Protein Coding	47	GC01M150707	2.46	https://www.genecards.org/cgi-bin/carddisp.pl?gene=MCL1

**CCND3**	Cyclin D3	Protein Coding	47	GC06M041934	2.46	https://www.genecards.org/cgi-bin/carddisp.pl?gene=CCND3
**HLA-DRA**	Major Histocompatibility Complex, Class II, DR Alpha	Protein Coding	47	GC06P032439	2.46	https://www.genecards.org/cgi-bin/carddisp.pl?gene=HLA-DRA
**DIABLO**	Diablo IAP-Binding Mitochondrial Protein	Protein Coding	47	GC12M122208	2.46	https://www.genecards.org/cgi-bin/carddisp.pl?gene=DIABLO
**MUC1**	Mucin 1, Cell Surface Associated	Protein Coding	47	GC01M155185	2.46	https://www.genecards.org/cgi-bin/carddisp.pl?gene=MUC1
**GPX4**	Glutathione Peroxidase 4	Protein Coding	47	GC19P001103	2.46	https://www.genecards.org/cgi-bin/carddisp.pl?gene=GPX4
**EIF4EBP1**	Eukaryotic Translation Initiation Factor 4E Binding Protein 1	Protein Coding	47	GC08P038007	2.46	https://www.genecards.org/cgi-bin/carddisp.pl?gene=EIF4EBP1
**SH2D1A**	SH2 Domain Containing 1A	Protein Coding	47	GC0XP124227	2.46	https://www.genecards.org/cgi-bin/carddisp.pl?gene=SH2D1A
**NPC1**	NPC Intracellular Cholesterol Transporter 1	Protein Coding	47	GC18M023506	2.46	https://www.genecards.org/cgi-bin/carddisp.pl?gene=NPC1

**PDE6A**	Phosphodiesterase 6A	Protein Coding	47	GC05M149857	2.46	https://www.genecards.org/cgi-bin/carddisp.pl?gene=PDE6A
**PRNP**	Prion Protein	Protein Coding	47	GC20P004686	2.46	https://www.genecards.org/cgi-bin/carddisp.pl?gene=PRNP
**SLC6A4**	Solute Carrier Family 6 Member 4	Protein Coding	47	GC17M030194	2.46	https://www.genecards.org/cgi-bin/carddisp.pl?gene=SLC6A4
**NPR 2.00**	Natriuretic Peptide Receptor 2	Protein Coding	47	GC09P035782	2.46	https://www.genecards.org/cgi-bin/carddisp.pl?gene=NPR2
**SSTR2**	Somatostatin Receptor 2	Protein Coding	47	GC17P073165	2.46	https://www.genecards.org/cgi-bin/carddisp.pl?gene=SSTR2
**TAP1**	Transporter 1, ATP Binding Cassette Subfamily B Member	Protein Coding	47	GC06M032865	2.46	https://www.genecards.org/cgi-bin/carddisp.pl?gene=TAP1
**TLR1**	Toll Like Receptor 1	Protein Coding	47	GC04M038797	2.46	https://www.genecards.org/cgi-bin/carddisp.pl?gene=TLR1
**OTC**	Ornithine Carbamoyltransferase	Protein Coding	47	GC0XP038353	2.46	https://www.genecards.org/cgi-bin/carddisp.pl?gene=OTC

**HTR1A**	5-Hydroxytryptamine Receptor 1A	Protein Coding	47	GC05M063960	2.46	https://www.genecards.org/cgi-bin/carddisp.pl?gene=HTR1A
**KCNJ1**	Potassium Inwardly Rectifying Channel Subfamily J Member 1	Protein Coding	47	GC11M128741	2.46	https://www.genecards.org/cgi-bin/carddisp.pl?gene=KCNJ1
**USP7**	Ubiquitin Specific Peptidase 7	Protein Coding	47	GC16M008892	2.46	https://www.genecards.org/cgi-bin/carddisp.pl?gene=USP7
**GNAI3**	G Protein Subunit Alpha I3	Protein Coding	46	GC01P109548	2.46	https://www.genecards.org/cgi-bin/carddisp.pl?gene=GNAI3
**BIRC3**	Baculoviral IAP Repeat Containing 3	Protein Coding	46	GC11P102317	2.46	https://www.genecards.org/cgi-bin/carddisp.pl?gene=BIRC3
**ACTN4**	Actinin Alpha 4	Protein Coding	46	GC19P038647	2.46	https://www.genecards.org/cgi-bin/carddisp.pl?gene=ACTN4
**MTR**	5-Methyltetrahydrofolate-Homocysteine Methyltransferase	Protein Coding	46	GC01P236795	2.46	https://www.genecards.org/cgi-bin/carddisp.pl?gene=MTR
**CD8A**	CD8a Molecule	Protein Coding	46	GC02M086784	2.46	https://www.genecards.org/cgi-bin/carddisp.pl?gene=CD8A

**PAX2**	Paired Box 2	Protein Coding	46	GC10P100735	2.46	https://www.genecards.org/cgi-bin/carddisp.pl?gene=PAX2
**PAX5**	Paired Box 5	Protein Coding	46	GC09M036828	2.46	https://www.genecards.org/cgi-bin/carddisp.pl?gene=PAX5
**PSMB9**	Proteasome 20S Subunit Beta 9	Protein Coding	46	GC06P047342	2.46	https://www.genecards.org/cgi-bin/carddisp.pl?gene=PSMB9
**ILK**	Integrin Linked Kinase	Protein Coding	46	GC11P006581	2.46	https://www.genecards.org/cgi-bin/carddisp.pl?gene=ILK
**TNFRSF13B**	TNF Receptor Superfamily Member 13B	Protein Coding	46	GC17M016929	2.46	https://www.genecards.org/cgi-bin/carddisp.pl?gene=TNFRSF13B
**LFNG**	LFNG O-Fucosylpeptide 3-Beta-N-Acetylglucosaminyltransferase	Protein Coding	46	GC07P002512	2.46	https://www.genecards.org/cgi-bin/carddisp.pl?gene=LFNG
**TK1**	Thymidine Kinase 1	Protein Coding	46	GC17M078175	2.46	https://www.genecards.org/cgi-bin/carddisp.pl?gene=TK1
**PRPS1**	Phosphoribosyl Pyrophosphate Synthetase 1	Protein Coding	46	GC0XP107628	2.46	https://www.genecards.org/cgi-bin/carddisp.pl?gene=PRPS1
**WNT1**	Wnt Family Member 1	Protein Coding	46	GC12P049053	2.46	https://www.genecards.org/cgi-bin/carddisp.pl?gene=WNT1

**CACNA1F**	Calcium Voltage-Gated Channel Subunit Alpha1 F	Protein Coding	45	GC0XM049205	2.46	https://www.genecards.org/cgi-bin/carddisp.pl?gene=CACNA1F
**APEX1**	Apurinic/Apyrimidinic Endodeoxyribonuclease 1	Protein Coding	45	GC14P020455	2.46	https://www.genecards.org/cgi-bin/carddisp.pl?gene=APEX1
**F11**	Coagulation Factor XI	Protein Coding	45	GC04P186265	2.46	https://www.genecards.org/cgi-bin/carddisp.pl?gene=F11
**ATP7A**	ATPase Copper Transporting Alpha	Protein Coding	45	GC0XP077927	2.46	https://www.genecards.org/cgi-bin/carddisp.pl?gene=ATP7A
**ATIC**	5-Aminoimidazole-4-Carboxamide Ribonucleotide Formyltransferase/IMP Cyclohydrolase	Protein Coding	45	GC02P215311	2.46	https://www.genecards.org/cgi-bin/carddisp.pl?gene=ATIC
**FDPS**	Farnesyl Diphosphate Synthase	Protein Coding	45	GC01P155308	2.46	https://www.genecards.org/cgi-bin/carddisp.pl?gene=FDPS
**ERCC1**	ERCC Excision Repair 1, Endonuclease Non-Catalytic Subunit	Protein Coding	45	GC19M045409	2.46	https://www.genecards.org/cgi-bin/carddisp.pl?gene=ERCC1
**ERN 1.00**	Endoplasmic Reticulum To Nucleus Signaling 1	Protein Coding	45	GC17M064039	2.46	https://www.genecards.org/cgi-bin/carddisp.pl?gene=ERN1

**BCL6**	BCL6 Transcription Repressor	Protein Coding	45	GC03M187721	2.46	https://www.genecards.org/cgi-bin/carddisp.pl?gene=BCL6
**BIRC2**	Baculoviral IAP Repeat Containing 2	Protein Coding	45	GC11P102347	2.46	https://www.genecards.org/cgi-bin/carddisp.pl?gene=BIRC2
**ADAMTS13**	ADAM Metallopeptidase With Thrombospondin Type 1 Motif 13	Protein Coding	45	GC09P133414	2.46	https://www.genecards.org/cgi-bin/carddisp.pl?gene=ADAMTS13
**CDK9**	Cyclin Dependent Kinase 9	Protein Coding	45	GC09P127814	2.46	https://www.genecards.org/cgi-bin/carddisp.pl?gene=CDK9
**HEXA**	Hexosaminidase Subunit Alpha	Protein Coding	45	GC15M072340	2.46	https://www.genecards.org/cgi-bin/carddisp.pl?gene=HEXA
**DHODH**	Dihydroorotate Dehydrogenase (Quinone)	Protein Coding	45	GC16P072008	2.46	https://www.genecards.org/cgi-bin/carddisp.pl?gene=DHODH
**GRIK1**	Glutamate Ionotropic Receptor Kainate Type Subunit 1	Protein Coding	45	GC21M029536	2.46	https://www.genecards.org/cgi-bin/carddisp.pl?gene=GRIK1
**CD22**	CD22 Molecule	Protein Coding	45	GC19P035319	2.46	https://www.genecards.org/cgi-bin/carddisp.pl?gene=CD22

**CD27**	CD27 Molecule	Protein Coding	45	GC12P008144	2.46	https://www.genecards.org/cgi-bin/carddisp.pl?gene=CD27
**RFC1**	Replication Factor C Subunit 1	Protein Coding	45	GC04M039291	2.46	https://www.genecards.org/cgi-bin/carddisp.pl?gene=RFC1
**EMD**	Emerin	Protein Coding	45	GC0XP154379	2.46	https://www.genecards.org/cgi-bin/carddisp.pl?gene=EMD
**NPHS1**	NPHS1 Adhesion Molecule, Nephrin	Protein Coding	45	GC19M035825	2.46	https://www.genecards.org/cgi-bin/carddisp.pl?gene=NPHS1
**HTR3A**	5-Hydroxytryptamine Receptor 3A	Protein Coding	45	GC11P113974	2.46	https://www.genecards.org/cgi-bin/carddisp.pl?gene=HTR3A
**PPP1CB**	Protein Phosphatase 1 Catalytic Subunit Beta	Protein Coding	45	GC02P028752	2.46	https://www.genecards.org/cgi-bin/carddisp.pl?gene=PPP1CB
**PPP1CC**	Protein Phosphatase 1 Catalytic Subunit Gamma	Protein Coding	45	GC12M110709	2.46	https://www.genecards.org/cgi-bin/carddisp.pl?gene=PPP1CC
**PRDM1**	PR/SET Domain 1	Protein Coding	45	GC06P105993	2.46	https://www.genecards.org/cgi-bin/carddisp.pl?gene=PRDM1

**PKD1**	Polycystin 1, Transient Receptor Potential Channel Interacting	Protein Coding	45	GC16M002348	2.46	https://www.genecards.org/cgi-bin/carddisp.pl?gene=PKD1
**PLOD3**	Procollagen-Lysine,2-Oxoglutarate 5-Dioxygenase 3	Protein Coding	45	GC07M101205	2.46	https://www.genecards.org/cgi-bin/carddisp.pl?gene=PLOD3
**SIRT5**	Sirtuin 5	Protein Coding	45	GC06P013574	2.46	https://www.genecards.org/cgi-bin/carddisp.pl?gene=SIRT5
**SLC19A1**	Solute Carrier Family 19 Member 1	Protein Coding	45	GC21M045493	2.46	https://www.genecards.org/cgi-bin/carddisp.pl?gene=SLC19A1
**SSTR3**	Somatostatin Receptor 3	Protein Coding	45	GC22M037204	2.46	https://www.genecards.org/cgi-bin/carddisp.pl?gene=SSTR3
**LAMA1**	Laminin Subunit Alpha 1	Protein Coding	45	GC18M006941	2.46	https://www.genecards.org/cgi-bin/carddisp.pl?gene=LAMA1
**TUBB4A**	Tubulin Beta 4A Class IVa	Protein Coding	45	GC19M006496	2.46	https://www.genecards.org/cgi-bin/carddisp.pl?gene=TUBB4A
**RAN**	RAN, Member RAS Oncogene Family	Protein Coding	45	GC12P130871	2.46	https://www.genecards.org/cgi-bin/carddisp.pl?gene=RAN

**STEAP3**	STEAP3 Metalloreductase	Protein Coding	45	GC02P119222	2.46	https://www.genecards.org/cgi-bin/carddisp.pl?gene=STEAP3
**STAT5A**	Signal Transducer And Activator Of Transcription 5A	Protein Coding	45	GC17P042287	2.46	https://www.genecards.org/cgi-bin/carddisp.pl?gene=STAT5A
**TRPM6**	Transient Receptor Potential Cation Channel Subfamily M Member 6	Protein Coding	45	GC09M074725	2.46	https://www.genecards.org/cgi-bin/carddisp.pl?gene=TRPM6
**XPO1**	Exportin 1	Protein Coding	45	GC02M061445	2.46	https://www.genecards.org/cgi-bin/carddisp.pl?gene=XPO1
**XPA**	XPA, DNA Damage Recognition And Repair Factor	Protein Coding	45	GC09M097635	2.46	https://www.genecards.org/cgi-bin/carddisp.pl?gene=XPA
**GFM1**	G Elongation Factor Mitochondrial 1	Protein Coding	44	GC03P158644	2.46	https://www.genecards.org/cgi-bin/carddisp.pl?gene=GFM1
**CARTPT**	CART Prepropeptide	Protein Coding	44	GC05P071719	2.46	https://www.genecards.org/cgi-bin/carddisp.pl?gene=CARTPT
**CBX5**	Chromobox 5	Protein Coding	44	GC12M054230	2.46	https://www.genecards.org/cgi-bin/carddisp.pl?gene=CBX5

**ARSB**	Arylsulfatase B	Protein Coding	44	GC05M078777	2.46	https://www.genecards.org/cgi-bin/carddisp.pl?gene=ARSB
**ANK3**	Ankyrin 3	Protein Coding	44	GC10M060026	2.46	https://www.genecards.org/cgi-bin/carddisp.pl?gene=ANK3
**BCL2L2**	BCL2 Like 2	Protein Coding	44	GC14P025306	2.46	https://www.genecards.org/cgi-bin/carddisp.pl?gene=BCL2L2
**MCOLN1**	Mucolipin 1	Protein Coding	44	GC19P007523	2.46	https://www.genecards.org/cgi-bin/carddisp.pl?gene=MCOLN1
**ACO1**	Aconitase 1	Protein Coding	44	GC09P032374	2.46	https://www.genecards.org/cgi-bin/carddisp.pl?gene=ACO1
**ADH4**	Alcohol Dehydrogenase 4 (Class II), Pi Polypeptide	Protein Coding	44	GC04M099123	2.46	https://www.genecards.org/cgi-bin/carddisp.pl?gene=ADH4
**FRK**	Fyn Related Src Family Tyrosine Kinase	Protein Coding	44	GC06M115931	2.46	https://www.genecards.org/cgi-bin/carddisp.pl?gene=FRK
**CKM**	Creatine Kinase, M-Type	Protein Coding	44	GC19M045306	2.46	https://www.genecards.org/cgi-bin/carddisp.pl?gene=CKM

**CLCNKB**	Chloride Voltage-Gated Channel Kb	Protein Coding	44	GC01P016043	2.46	https://www.genecards.org/cgi-bin/carddisp.pl?gene=CLCNKB
**GPNMB**	Glycoprotein Nmb	Protein Coding	44	GC07P023238	2.46	https://www.genecards.org/cgi-bin/carddisp.pl?gene=GPNMB
**COL4A3**	Collagen Type IV Alpha 3 Chain	Protein Coding	44	GC02P227164	2.46	https://www.genecards.org/cgi-bin/carddisp.pl?gene=COL4A3
**SGSH**	N-Sulfoglucosamine Sulfohydrolase	Protein Coding	44	GC17M080206	2.46	https://www.genecards.org/cgi-bin/carddisp.pl?gene=SGSH
**SEC24D**	SEC24 Homolog D, COPII Coat Complex Component	Protein Coding	44	GC04M118722	2.46	https://www.genecards.org/cgi-bin/carddisp.pl?gene=SEC24D
**IGFALS**	Insulin Like Growth Factor Binding Protein Acid Labile Subunit	Protein Coding	44	GC16M001790	2.46	https://www.genecards.org/cgi-bin/carddisp.pl?gene=IGFALS
**PLA2G10**	Phospholipase A2 Group X	Protein Coding	44	GC16M014672	2.46	https://www.genecards.org/cgi-bin/carddisp.pl?gene=PLA2G10
**SLC9A3R1**	SLC9A3 Regulator 1	Protein Coding	44	GC17P074749	2.46	https://www.genecards.org/cgi-bin/carddisp.pl?gene=SLC9A3R1

**PLCE1**	Phospholipase C Epsilon 1	Protein Coding	44	GC10P093993	2.46	https://www.genecards.org/cgi-bin/carddisp.pl?gene=PLCE1
**SLC22A12**	Solute Carrier Family 22 Member 12	Protein Coding	44	GC11P064609	2.46	https://www.genecards.org/cgi-bin/carddisp.pl?gene=SLC22A12
**TNFRSF9**	TNF Receptor Superfamily Member 9	Protein Coding	44	GC01M007915	2.46	https://www.genecards.org/cgi-bin/carddisp.pl?gene=TNFRSF9
**TMPRSS6**	Transmembrane Serine Protease 6	Protein Coding	44	GC22M037066	2.46	https://www.genecards.org/cgi-bin/carddisp.pl?gene=TMPRSS6
**LAMP2**	Lysosomal Associated Membrane Protein 2	Protein Coding	44	GC0XM120426	2.46	https://www.genecards.org/cgi-bin/carddisp.pl?gene=LAMP2
**LIG3**	DNA Ligase 3	Protein Coding	44	GC17P034980	2.46	https://www.genecards.org/cgi-bin/carddisp.pl?gene=LIG3
**STS**	Steroid Sulfatase	Protein Coding	44	GC0XP007146	2.46	https://www.genecards.org/cgi-bin/carddisp.pl?gene=STS
**TAP2**	Transporter 2, ATP Binding Cassette Subfamily B Member	Protein Coding	44	GC06M032821	2.46	https://www.genecards.org/cgi-bin/carddisp.pl?gene=TAP2
**TAT**	Tyrosine Aminotransferase	Protein Coding	44	GC16M071565	2.46	https://www.genecards.org/cgi-bin/carddisp.pl?gene=TAT

**PSMB5**	Proteasome 20S Subunit Beta 5	Protein Coding	44	GC14M023016	2.46	https://www.genecards.org/cgi-bin/carddisp.pl?gene=PSMB5
**TP53BP1**	Tumor Protein P53 Binding Protein 1	Protein Coding	44	GC15M043403	2.46	https://www.genecards.org/cgi-bin/carddisp.pl?gene=TP53BP1
**RPE65**	Retinoid Isomerohydrolase RPE65	Protein Coding	44	GC01M068428	2.46	https://www.genecards.org/cgi-bin/carddisp.pl?gene=RPE65
**PSMB10**	Proteasome 20S Subunit Beta 10	Protein Coding	44	GC16M067937	2.46	https://www.genecards.org/cgi-bin/carddisp.pl?gene=PSMB10
**TFR2**	Transferrin Receptor 2	Protein Coding	44	GC07M100620	2.46	https://www.genecards.org/cgi-bin/carddisp.pl?gene=TFR2
**XRCC5**	X-Ray Repair Cross Complementing 5	Protein Coding	44	GC02P216107	2.46	https://www.genecards.org/cgi-bin/carddisp.pl?gene=XRCC5
**ATN1**	Atrophin 1	Protein Coding	43	GC12P008222	2.46	https://www.genecards.org/cgi-bin/carddisp.pl?gene=ATN1
**M6PR**	Mannose-6-Phosphate Receptor, Cation Dependent	Protein Coding	43	GC12M008955	2.46	https://www.genecards.org/cgi-bin/carddisp.pl?gene=M6PR

**MBTPS2**	Membrane Bound Transcription Factor Peptidase, Site 2	Protein Coding	43	GC0XP021839	2.46	https://www.genecards.org/cgi-bin/carddisp.pl?gene=MBTPS2
**ADH1B**	Alcohol Dehydrogenase 1B (Class I), Beta Polypeptide	Protein Coding	43	GC04M099304	2.46	https://www.genecards.org/cgi-bin/carddisp.pl?gene=ADH1B
**GALC**	Galactosylceramidase	Protein Coding	43	GC14M087837	2.46	https://www.genecards.org/cgi-bin/carddisp.pl?gene=GALC
**APBB1**	Amyloid Beta Precursor Protein Binding Family B Member 1	Protein Coding	43	GC11M006396	2.46	https://www.genecards.org/cgi-bin/carddisp.pl?gene=APBB1
**LOXL1**	Lysyl Oxidase Like 1	Protein Coding	43	GC15P073925	2.46	https://www.genecards.org/cgi-bin/carddisp.pl?gene=LOXL1
**ERAP1**	Endoplasmic Reticulum Aminopeptidase 1	Protein Coding	43	GC05M096760	2.46	https://www.genecards.org/cgi-bin/carddisp.pl?gene=ERAP1
**CLCN4**	Chloride Voltage-Gated Channel 4	Protein Coding	43	GC0XP010085	2.46	https://www.genecards.org/cgi-bin/carddisp.pl?gene=CLCN4
**ROBO3**	Roundabout Guidance Receptor 3	Protein Coding	43	GC11P124865	2.46	https://www.genecards.org/cgi-bin/carddisp.pl?gene=ROBO3

**CD226**	CD226 Molecule	Protein Coding	43	GC18M069831	2.46	https://www.genecards.org/cgi-bin/carddisp.pl?gene=CD226
**CD2AP**	CD2 Associated Protein	Protein Coding	43	GC06P047777	2.46	https://www.genecards.org/cgi-bin/carddisp.pl?gene=CD2AP
**CD33**	CD33 Molecule	Protein Coding	43	GC19P051212	2.46	https://www.genecards.org/cgi-bin/carddisp.pl?gene=CD33
**COL4A4**	Collagen Type IV Alpha 4 Chain	Protein Coding	43	GC02M226967	2.46	https://www.genecards.org/cgi-bin/carddisp.pl?gene=COL4A4
**SPG7**	SPG7 Matrix AAA Peptidase Subunit, Paraplegin	Protein Coding	43	GC16P089492	2.46	https://www.genecards.org/cgi-bin/carddisp.pl?gene=SPG7
**SI**	Sucrase-Isomaltase	Protein Coding	43	GC03M164978	2.46	https://www.genecards.org/cgi-bin/carddisp.pl?gene=SI
**SKP1**	S-Phase Kinase Associated Protein 1	Protein Coding	43	GC05M134148	2.46	https://www.genecards.org/cgi-bin/carddisp.pl?gene=SKP1
**PAX1**	Paired Box 1	Protein Coding	43	GC20P021705	2.46	https://www.genecards.org/cgi-bin/carddisp.pl?gene=PAX1

**SLC29A3**	Solute Carrier Family 29 Member 3	Protein Coding	43	GC10P071320	2.46	https://www.genecards.org/cgi-bin/carddisp.pl?gene=SLC29A3
**OCA2**	OCA2 Melanosomal Transmembrane Protein	Protein Coding	43	GC15M027754	2.46	https://www.genecards.org/cgi-bin/carddisp.pl?gene=OCA2
**PSMD14**	Proteasome 26S Subunit, Non-ATPase 14	Protein Coding	43	GC02P161308	2.46	https://www.genecards.org/cgi-bin/carddisp.pl?gene=PSMD14
**SPTA1**	Spectrin Alpha, Erythrocytic 1	Protein Coding	43	GC01M158610	2.46	https://www.genecards.org/cgi-bin/carddisp.pl?gene=SPTA1
**SSTR1**	Somatostatin Receptor 1	Protein Coding	43	GC14P038207	2.46	https://www.genecards.org/cgi-bin/carddisp.pl?gene=SSTR1
**TRHR**	Thyrotropin Releasing Hormone Receptor	Protein Coding	43	GC08P109084	2.46	https://www.genecards.org/cgi-bin/carddisp.pl?gene=TRHR
**IRF4**	Interferon Regulatory Factor 4	Protein Coding	43	GC06P000391	2.46	https://www.genecards.org/cgi-bin/carddisp.pl?gene=IRF4
**RBBP4**	RB Binding Protein 4, Chromatin Remodeling Factor	Protein Coding	43	GC01P032651	2.46	https://www.genecards.org/cgi-bin/carddisp.pl?gene=RBBP4

**TAPBP**	TAP Binding Protein	Protein Coding	43	GC06M033299	2.46	https://www.genecards.org/cgi-bin/carddisp.pl?gene=TAPBP
**SUMF1**	Sulfatase Modifying Factor 1	Protein Coding	43	GC03M003700	2.46	https://www.genecards.org/cgi-bin/carddisp.pl?gene=SUMF1
**MTRR**	5-Methyltetrahydrofolate-Homocysteine Methyltransferase Reductase	Protein Coding	43	GC05P007851	2.46	https://www.genecards.org/cgi-bin/carddisp.pl?gene=MTRR
**NAGLU**	N-Acetyl-Alpha-Glucosaminidase	Protein Coding	43	GC17P042535	2.46	https://www.genecards.org/cgi-bin/carddisp.pl?gene=NAGLU
**NARS2**	Asparaginyl-TRNA Synthetase 2, Mitochondrial	Protein Coding	43	GC11M078435	2.46	https://www.genecards.org/cgi-bin/carddisp.pl?gene=NARS2
**SUV39H1**	Suppressor Of Variegation 3-9 Homolog 1	Protein Coding	43	GC0XP048698	2.46	https://www.genecards.org/cgi-bin/carddisp.pl?gene=SUV39H1
**TFE3**	Transcription Factor Binding To IGHM Enhancer 3	Protein Coding	43	GC0XM049028	2.46	https://www.genecards.org/cgi-bin/carddisp.pl?gene=TFE3
**VAV3**	Vav Guanine Nucleotide Exchange Factor 3	Protein Coding	43	GC01M107571	2.46	https://www.genecards.org/cgi-bin/carddisp.pl?gene=VAV3

**LMNB2**	Lamin B2	Protein Coding	43	GC19M002439	2.46	https://www.genecards.org/cgi-bin/carddisp.pl?gene=LMNB2
**CCS**	Copper Chaperone For Superoxide Dismutase	Protein Coding	42	GC11P066593	2.46	https://www.genecards.org/cgi-bin/carddisp.pl?gene=CCS
**BANF1**	BAF Nuclear Assembly Factor 1	Protein Coding	42	GC11P066002	2.46	https://www.genecards.org/cgi-bin/carddisp.pl?gene=BANF1
**MEOX1**	Mesenchyme Homeobox 1	Protein Coding	42	GC17M043640	2.46	https://www.genecards.org/cgi-bin/carddisp.pl?gene=MEOX1
**ADH1A**	Alcohol Dehydrogenase 1A (Class I), Alpha Polypeptide	Protein Coding	42	GC04M099276	2.46	https://www.genecards.org/cgi-bin/carddisp.pl?gene=ADH1A
**ACTC1**	Actin Alpha Cardiac Muscle 1	Protein Coding	42	GC15M034788	2.46	https://www.genecards.org/cgi-bin/carddisp.pl?gene=ACTC1
**BRDT**	Bromodomain Testis Associated	Protein Coding	42	GC01P091949	2.46	https://www.genecards.org/cgi-bin/carddisp.pl?gene=BRDT
**CLCN5**	Chloride Voltage-Gated Channel 5	Protein Coding	42	GC0XP049922	2.46	https://www.genecards.org/cgi-bin/carddisp.pl?gene=CLCN5

**COMMD1**	Copper Metabolism Domain Containing 1	Protein Coding	42	GC02P061888	2.46	https://www.genecards.org/cgi-bin/carddisp.pl?gene=COMMD1
**MOCOS**	Molybdenum Cofactor Sulfurase	Protein Coding	42	GC18P036187	2.46	https://www.genecards.org/cgi-bin/carddisp.pl?gene=MOCOS
**HPGDS**	Hematopoietic Prostaglandin D Synthase	Protein Coding	42	GC04M094298	2.46	https://www.genecards.org/cgi-bin/carddisp.pl?gene=HPGDS
**RPS26**	Ribosomal Protein S26	Protein Coding	42	GC12P056043	2.46	https://www.genecards.org/cgi-bin/carddisp.pl?gene=RPS26
**SLC31A1**	Solute Carrier Family 31 Member 1	Protein Coding	42	GC09P113221	2.46	https://www.genecards.org/cgi-bin/carddisp.pl?gene=SLC31A1
**TNFRSF8**	TNF Receptor Superfamily Member 8	Protein Coding	42	GC01P012063	2.46	https://www.genecards.org/cgi-bin/carddisp.pl?gene=TNFRSF8
**UCHL5**	Ubiquitin C-Terminal Hydrolase L5	Protein Coding	42	GC01M193012	2.46	https://www.genecards.org/cgi-bin/carddisp.pl?gene=UCHL5
**TRH**	Thyrotropin Releasing Hormone	Protein Coding	42	GC03P129974	2.46	https://www.genecards.org/cgi-bin/carddisp.pl?gene=TRH

**RBX1**	Ring-Box 1	Protein Coding	42	GC22P040951	2.46	https://www.genecards.org/cgi-bin/carddisp.pl?gene=RBX1
**TMEM38B**	Transmembrane Protein 38B	Protein Coding	42	GC09P105694	2.46	https://www.genecards.org/cgi-bin/carddisp.pl?gene=TMEM38B
**CD1D**	CD1d Molecule	Protein Coding	42	GC01P158178	2.46	https://www.genecards.org/cgi-bin/carddisp.pl?gene=CD1D
**STX16**	Syntaxin 16	Protein Coding	42	GC20P058652	2.46	https://www.genecards.org/cgi-bin/carddisp.pl?gene=STX16
**GDF3**	Growth Differentiation Factor 3	Protein Coding	41	GC12M007656	2.46	https://www.genecards.org/cgi-bin/carddisp.pl?gene=GDF3
**GCM2**	Glial Cells Missing Transcription Factor 2	Protein Coding	41	GC06M010873	2.46	https://www.genecards.org/cgi-bin/carddisp.pl?gene=GCM2
**GNRH1**	Gonadotropin Releasing Hormone 1	Protein Coding	41	GC08M025419	2.46	https://www.genecards.org/cgi-bin/carddisp.pl?gene=GNRH1
**GNA15**	G Protein Subunit Alpha 15	Protein Coding	41	GC19P003154	2.46	https://www.genecards.org/cgi-bin/carddisp.pl?gene=GNA15

**MICB**	MHC Class I Polypeptide-Related Sequence B	Protein Coding	41	GC06P047300	2.46	https://www.genecards.org/cgi-bin/carddisp.pl?gene=MICB
**LOXL3**	Lysyl Oxidase Like 3	Protein Coding	41	GC02M074532	2.46	https://www.genecards.org/cgi-bin/carddisp.pl?gene=LOXL3
**AP2S1**	Adaptor Related Protein Complex 2 Subunit Sigma 1	Protein Coding	41	GC19M046838	2.46	https://www.genecards.org/cgi-bin/carddisp.pl?gene=AP2S1
**ASAH2**	N-Acylsphingosine Amidohydrolase 2	Protein Coding	41	GC10M050182	2.46	https://www.genecards.org/cgi-bin/carddisp.pl?gene=ASAH2
**AFF2**	AF4/FMR2 Family Member 2	Protein Coding	41	GC0XP148500	2.46	https://www.genecards.org/cgi-bin/carddisp.pl?gene=AFF2
**FOXC1**	Forkhead Box C1	Protein Coding	41	GC06P001610	2.46	https://www.genecards.org/cgi-bin/carddisp.pl?gene=FOXC1
**FCAR**	Fc Fragment Of IgA Receptor	Protein Coding	41	GC19P055445	2.46	https://www.genecards.org/cgi-bin/carddisp.pl?gene=FCAR
**ERCC8**	ERCC Excision Repair 8, CSA Ubiquitin Ligase Complex Subunit	Protein Coding	41	GC05M060868	2.46	https://www.genecards.org/cgi-bin/carddisp.pl?gene=ERCC8

**CD5**	CD5 Molecule	Protein Coding	41	GC11P061114	2.46	https://www.genecards.org/cgi-bin/carddisp.pl?gene=CD5
**GSTZ1**	Glutathione S-Transferase Zeta 1	Protein Coding	41	GC14P077320	2.46	https://www.genecards.org/cgi-bin/carddisp.pl?gene=GSTZ1
**CNN1**	Calponin 1	Protein Coding	41	GC19P011539	2.46	https://www.genecards.org/cgi-bin/carddisp.pl?gene=CNN1
**CKS1B**	CDC28 Protein Kinase Regulatory Subunit 1B	Protein Coding	41	GC01P154974	2.46	https://www.genecards.org/cgi-bin/carddisp.pl?gene=CKS1B
**CLDN16**	Claudin 16	Protein Coding	41	GC03P190290	2.46	https://www.genecards.org/cgi-bin/carddisp.pl?gene=CLDN16
**CHST14**	Carbohydrate Sulfotransferase 14	Protein Coding	41	GC15P040470	2.46	https://www.genecards.org/cgi-bin/carddisp.pl?gene=CHST14
**HCCS**	Holocytochrome C Synthase	Protein Coding	41	GC0XP011111	2.46	https://www.genecards.org/cgi-bin/carddisp.pl?gene=HCCS
**GPR143**	G Protein-Coupled Receptor 143	Protein Coding	41	GC0XM009725	2.46	https://www.genecards.org/cgi-bin/carddisp.pl?gene=GPR143

**MPG**	N-Methylpurine DNA Glycosylase	Protein Coding	41	GC16P001366	2.46	https://www.genecards.org/cgi-bin/carddisp.pl?gene=MPG
**NEDD8**	NEDD8 Ubiquitin Like Modifier	Protein Coding	41	GC14M024216	2.46	https://www.genecards.org/cgi-bin/carddisp.pl?gene=NEDD8
**RPL34**	Ribosomal Protein L34	Protein Coding	41	GC04P108620	2.46	https://www.genecards.org/cgi-bin/carddisp.pl?gene=RPL34
**HLA-DMB**	Major Histocompatibility Complex, Class II, DM Beta	Protein Coding	41	GC06M032934	2.46	https://www.genecards.org/cgi-bin/carddisp.pl?gene=HLA-DMB
**IKZF3**	IKAROS Family Zinc Finger 3	Protein Coding	41	GC17M039759	2.46	https://www.genecards.org/cgi-bin/carddisp.pl?gene=IKZF3
**NPHS2**	NPHS2 Stomatin Family Member, Podocin	Protein Coding	41	GC01M179519	2.46	https://www.genecards.org/cgi-bin/carddisp.pl?gene=NPHS2
**ICMT**	Isoprenylcysteine Carboxyl Methyltransferase	Protein Coding	41	GC01M006222	2.46	https://www.genecards.org/cgi-bin/carddisp.pl?gene=ICMT
**SNX10**	Sorting Nexin 10	Protein Coding	41	GC07P026291	2.46	https://www.genecards.org/cgi-bin/carddisp.pl?gene=SNX10

**PCLO**	Piccolo Presynaptic Cytomatrix Protein	Protein Coding	41	GC07M082754	2.46	https://www.genecards.org/cgi-bin/carddisp.pl?gene=PCLO
**PNPLA3**	Patatin Like Phospholipase Domain Containing 3	Protein Coding	41	GC22P043923	2.46	https://www.genecards.org/cgi-bin/carddisp.pl?gene=PNPLA3
**PLEKHM1**	Pleckstrin Homology And RUN Domain Containing M1	Protein Coding	41	GC17M045435	2.46	https://www.genecards.org/cgi-bin/carddisp.pl?gene=PLEKHM1
**PLS3**	Plastin 3	Protein Coding	41	GC0XP115560	2.46	https://www.genecards.org/cgi-bin/carddisp.pl?gene=PLS3
**SIRT7**	Sirtuin 7	Protein Coding	41	GC17M081911	2.46	https://www.genecards.org/cgi-bin/carddisp.pl?gene=SIRT7
**SLC24A5**	Solute Carrier Family 24 Member 5	Protein Coding	41	GC15P048120	2.46	https://www.genecards.org/cgi-bin/carddisp.pl?gene=SLC24A5
**SLAMF7**	SLAM Family Member 7	Protein Coding	41	GC01P160709	2.46	https://www.genecards.org/cgi-bin/carddisp.pl?gene=SLAMF7
**RPS3A**	Ribosomal Protein S3A	Protein Coding	41	GC04P151099	2.46	https://www.genecards.org/cgi-bin/carddisp.pl?gene=RPS3A

**SSTR4**	Somatostatin Receptor 4	Protein Coding	41	GC20P023035	2.46	https://www.genecards.org/cgi-bin/carddisp.pl?gene=SSTR4
**SSTR5**	Somatostatin Receptor 5	Protein Coding	41	GC16P001072	2.46	https://www.genecards.org/cgi-bin/carddisp.pl?gene=SSTR5
**TWIST2**	Twist Family BHLH Transcription Factor 2	Protein Coding	41	GC02P238848	2.46	https://www.genecards.org/cgi-bin/carddisp.pl?gene=TWIST2
**U2AF1**	U2 Small Nuclear RNA Auxiliary Factor 1	Protein Coding	41	GC21M043092	2.46	https://www.genecards.org/cgi-bin/carddisp.pl?gene=U2AF1
**LEMD3**	LEM Domain Containing 3	Protein Coding	41	GC12P065169	2.46	https://www.genecards.org/cgi-bin/carddisp.pl?gene=LEMD3
**SYNE1**	Spectrin Repeat Containing Nuclear Envelope Protein 1	Protein Coding	41	GC06M152121	2.46	https://www.genecards.org/cgi-bin/carddisp.pl?gene=SYNE1
**SUCLG2**	Succinate-CoA Ligase GDP-Forming Subunit Beta	Protein Coding	41	GC03M067358	2.46	https://www.genecards.org/cgi-bin/carddisp.pl?gene=SUCLG2
**TKTL1**	Transketolase Like 1	Protein Coding	41	GC0XP154295	2.46	https://www.genecards.org/cgi-bin/carddisp.pl?gene=TKTL1

**UBA7**	Ubiquitin Like Modifier Activating Enzyme 7	Protein Coding	41	GC03M049805	2.46	https://www.genecards.org/cgi-bin/carddisp.pl?gene=UBA7
**CAPZB**	Capping Actin Protein Of Muscle Z-Line Subunit Beta	Protein Coding	40	GC01M019339	2.46	https://www.genecards.org/cgi-bin/carddisp.pl?gene=CAPZB
**GHRH**	Growth Hormone Releasing Hormone	Protein Coding	40	GC20M037251	2.46	https://www.genecards.org/cgi-bin/carddisp.pl?gene=GHRH
**ATOX1**	Antioxidant 1 Copper Chaperone	Protein Coding	40	GC05M151743	2.46	https://www.genecards.org/cgi-bin/carddisp.pl?gene=ATOX1
**FKBP10**	FKBP Prolyl Isomerase 10	Protein Coding	40	GC17P041812	2.46	https://www.genecards.org/cgi-bin/carddisp.pl?gene=FKBP10
**BSND**	Barttin CLCNK Type Accessory Subunit Beta	Protein Coding	40	GC01P054998	2.46	https://www.genecards.org/cgi-bin/carddisp.pl?gene=BSND
**CYBRD1**	Cytochrome B Reductase 1	Protein Coding	40	GC02P171522	2.46	https://www.genecards.org/cgi-bin/carddisp.pl?gene=CYBRD1
**CLN8**	CLN8 Transmembrane ER And ERGIC Protein	Protein Coding	40	GC08P001755	2.46	https://www.genecards.org/cgi-bin/carddisp.pl?gene=CLN8

**DNAJB11**	DnaJ Heat Shock Protein Family (Hsp40) Member B11	Protein Coding	40	GC03P186567	2.46	https://www.genecards.org/cgi-bin/carddisp.pl?gene=DNAJB11
**DIS3**	DIS3 Homolog, Exosome Endoribonuclease And 3'-5' Exoribonuclease	Protein Coding	40	GC13M072752	2.46	https://www.genecards.org/cgi-bin/carddisp.pl?gene=DIS3
**MOCS1**	Molybdenum Cofactor Synthesis 1	Protein Coding	40	GC06M039899	2.46	https://www.genecards.org/cgi-bin/carddisp.pl?gene=MOCS1
**CD69**	CD69 Molecule	Protein Coding	40	GC12M013857	2.46	https://www.genecards.org/cgi-bin/carddisp.pl?gene=CD69
**HLA-DMA**	Major Histocompatibility Complex, Class II, DM Alpha	Protein Coding	40	GC06M032950	2.46	https://www.genecards.org/cgi-bin/carddisp.pl?gene=HLA-DMA
**HLA-DOA**	Major Histocompatibility Complex, Class II, DO Alpha	Protein Coding	40	GC06M033004	2.46	https://www.genecards.org/cgi-bin/carddisp.pl?gene=HLA-DOA
**CRTAP**	Cartilage Associated Protein	Protein Coding	40	GC03P033113	2.46	https://www.genecards.org/cgi-bin/carddisp.pl?gene=CRTAP
**NPSR1**	Neuropeptide S Receptor 1	Protein Coding	40	GC07P034664	2.46	https://www.genecards.org/cgi-bin/carddisp.pl?gene=NPSR1

**PROP1**	PROP Paired-Like Homeobox 1	Protein Coding	40	GC05M177992	2.46	https://www.genecards.org/cgi-bin/carddisp.pl?gene=PROP1
**PLEK**	Pleckstrin	Protein Coding	40	GC02P068365	2.46	https://www.genecards.org/cgi-bin/carddisp.pl?gene=PLEK
**SIRT4**	Sirtuin 4	Protein Coding	40	GC12P120291	2.46	https://www.genecards.org/cgi-bin/carddisp.pl?gene=SIRT4
**SLC45A2**	Solute Carrier Family 45 Member 2	Protein Coding	40	GC05M033944	2.46	https://www.genecards.org/cgi-bin/carddisp.pl?gene=SLC45A2
**ONECUT1**	One Cut Homeobox 1	Protein Coding	40	GC15M060601	2.46	https://www.genecards.org/cgi-bin/carddisp.pl?gene=ONECUT1
**NUP153**	Nucleoporin 153	Protein Coding	40	GC06M017615	2.46	https://www.genecards.org/cgi-bin/carddisp.pl?gene=NUP153
**SLAMF1**	Signaling Lymphocytic Activation Molecule Family Member 1	Protein Coding	40	GC01M160608	2.46	https://www.genecards.org/cgi-bin/carddisp.pl?gene=SLAMF1
**PIGT**	Phosphatidylinositol Glycan Anchor Biosynthesis Class T	Protein Coding	40	GC20P045416	2.46	https://www.genecards.org/cgi-bin/carddisp.pl?gene=PIGT

**KLRC1**	Killer Cell Lectin Like Receptor C1	Protein Coding	40	GC12M013870	2.46	https://www.genecards.org/cgi-bin/carddisp.pl?gene=KLRC1
**KLRK1**	Killer Cell Lectin Like Receptor K1	Protein Coding	40	GC12M013865	2.46	https://www.genecards.org/cgi-bin/carddisp.pl?gene=KLRK1
**INF2**	Inverted Formin 2	Protein Coding	40	GC14P104788	2.46	https://www.genecards.org/cgi-bin/carddisp.pl?gene=INF2
**KDM4C**	Lysine Demethylase 4C	Protein Coding	40	GC09P006720	2.46	https://www.genecards.org/cgi-bin/carddisp.pl?gene=KDM4C
**CARD8**	Caspase Recruitment Domain Family Member 8	Protein Coding	39	GC19M048183	2.46	https://www.genecards.org/cgi-bin/carddisp.pl?gene=CARD8
**GNPTG**	N-Acetylglucosamine-1-Phosphate Transferase Subunit Gamma	Protein Coding	39	GC16P001351	2.46	https://www.genecards.org/cgi-bin/carddisp.pl?gene=GNPTG
**CCRL2**	C-C Motif Chemokine Receptor Like 2	Protein Coding	39	GC03P046407	2.46	https://www.genecards.org/cgi-bin/carddisp.pl?gene=CCRL2
**ALX1**	ALX Homeobox 1	Protein Coding	39	GC12P085279	2.46	https://www.genecards.org/cgi-bin/carddisp.pl?gene=ALX1

**ALX4**	ALX Homeobox 4	Protein Coding	39	GC11M044238	2.46	https://www.genecards.org/cgi-bin/carddisp.pl?gene=ALX4
**FUZ**	Fuzzy Planar Cell Polarity Protein	Protein Coding	39	GC19M049806	2.46	https://www.genecards.org/cgi-bin/carddisp.pl?gene=FUZ
**FRMD7**	FERM Domain Containing 7	Protein Coding	39	GC0XM132077	2.46	https://www.genecards.org/cgi-bin/carddisp.pl?gene=FRMD7
**HPS4**	HPS4 Biogenesis Of Lysosomal Organelles Complex 3 Subunit 2	Protein Coding	39	GC22M026443	2.46	https://www.genecards.org/cgi-bin/carddisp.pl?gene=HPS4
**HEPH**	Hephaestin	Protein Coding	39	GC0XP066162	2.46	https://www.genecards.org/cgi-bin/carddisp.pl?gene=HEPH
**CELSR3**	Cadherin EGF LAG Seven-Pass G-Type Receptor 3	Protein Coding	39	GC03M048641	2.46	https://www.genecards.org/cgi-bin/carddisp.pl?gene=CELSR3
**HGSNAT**	Heparan-Alpha-Glucosaminide N-Acetyltransferase	Protein Coding	39	GC08P043140	2.46	https://www.genecards.org/cgi-bin/carddisp.pl?gene=HGSNAT
**MLANA**	Melan-A	Protein Coding	39	GC09P005846	2.46	https://www.genecards.org/cgi-bin/carddisp.pl?gene=MLANA

**MYOM2**	Myomesin 2	Protein Coding	39	GC08P002045	2.46	https://www.genecards.org/cgi-bin/carddisp.pl?gene=MYOM2
**GPRC6A**	G Protein-Coupled Receptor Class C Group 6 Member A	Protein Coding	39	GC06M116793	2.46	https://www.genecards.org/cgi-bin/carddisp.pl?gene=GPRC6A
**DDX39B**	DExD-Box Helicase 39B	Protein Coding	39	GC06M031530	2.46	https://www.genecards.org/cgi-bin/carddisp.pl?gene=DDX39B
**NCR2**	Natural Cytotoxicity Triggering Receptor 2	Protein Coding	39	GC06P047497	2.46	https://www.genecards.org/cgi-bin/carddisp.pl?gene=NCR2
**NCR3**	Natural Cytotoxicity Triggering Receptor 3	Protein Coding	39	GC06M031588	2.46	https://www.genecards.org/cgi-bin/carddisp.pl?gene=NCR3
**CPNE3**	Copine 3	Protein Coding	39	GC08P086514	2.46	https://www.genecards.org/cgi-bin/carddisp.pl?gene=CPNE3
**COPE**	COPI Coat Complex Subunit Epsilon	Protein Coding	39	GC19M018899	2.46	https://www.genecards.org/cgi-bin/carddisp.pl?gene=COPE
**PDLIM5**	PDZ And LIM Domain 5	Protein Coding	39	GC04P094451	2.46	https://www.genecards.org/cgi-bin/carddisp.pl?gene=PDLIM5

**PRSS2**	Serine Protease 2	Protein Coding	39	GC07P144959	2.46	https://www.genecards.org/cgi-bin/carddisp.pl?gene=PRSS2
**NIT2**	Nitrilase Family Member 2	Protein Coding	39	GC03P100334	2.46	https://www.genecards.org/cgi-bin/carddisp.pl?gene=NIT2
**PMAIP1**	Phorbol-12-Myristate-13-Acetate-Induced Protein 1	Protein Coding	39	GC18P059899	2.46	https://www.genecards.org/cgi-bin/carddisp.pl?gene=PMAIP1
**PIPOX**	Pipecolic Acid And Sarcosine Oxidase	Protein Coding	39	GC17P028951	2.46	https://www.genecards.org/cgi-bin/carddisp.pl?gene=PIPOX
**LEMD2**	LEM Domain Nuclear Envelope Protein 2	Protein Coding	39	GC06M033772	2.46	https://www.genecards.org/cgi-bin/carddisp.pl?gene=LEMD2
**SYNE2**	Spectrin Repeat Containing Nuclear Envelope Protein 2	Protein Coding	39	GC14P063761	2.46	https://www.genecards.org/cgi-bin/carddisp.pl?gene=SYNE2
**TBX6**	T-Box Transcription Factor 6	Protein Coding	39	GC16M030085	2.46	https://www.genecards.org/cgi-bin/carddisp.pl?gene=TBX6
**LDB1**	LIM Domain Binding 1	Protein Coding	39	GC10M102106	2.46	https://www.genecards.org/cgi-bin/carddisp.pl?gene=LDB1

**NAT10**	N-Acetyltransferase 10	Protein Coding	39	GC11P034105	2.46	https://www.genecards.org/cgi-bin/carddisp.pl?gene=NAT10
**KRT86**	Keratin 86	Protein Coding	39	GC12P052249	2.46	https://www.genecards.org/cgi-bin/carddisp.pl?gene=KRT86
**LRRK1**	Leucine Rich Repeat Kinase 1	Protein Coding	38	GC15P100919	2.46	https://www.genecards.org/cgi-bin/carddisp.pl?gene=LRRK1
**ASAP2**	ArfGAP With SH3 Domain, Ankyrin Repeat And PH Domain 2	Protein Coding	38	GC02P009206	2.46	https://www.genecards.org/cgi-bin/carddisp.pl?gene=ASAP2
**COL15A1**	Collagen Type XV Alpha 1 Chain	Protein Coding	38	GC09P098943	2.46	https://www.genecards.org/cgi-bin/carddisp.pl?gene=COL15A1
**MYO18B**	Myosin XVIIIB	Protein Coding	38	GC22P025742	2.46	https://www.genecards.org/cgi-bin/carddisp.pl?gene=MYO18B
**DBP**	D-Box Binding PAR BZIP Transcription Factor	Protein Coding	38	GC19M048630	2.46	https://www.genecards.org/cgi-bin/carddisp.pl?gene=DBP
**NYX**	Nyctalopin	Protein Coding	38	GC0XP041447	2.46	https://www.genecards.org/cgi-bin/carddisp.pl?gene=NYX

**KIAA0319L**	KIAA0319 Like	Protein Coding	38	GC01M035433	2.46	https://www.genecards.org/cgi-bin/carddisp.pl?gene=KIAA0319L
**LDB2**	LIM Domain Binding 2	Protein Coding	38	GC04M016445	2.46	https://www.genecards.org/cgi-bin/carddisp.pl?gene=LDB2
**OSTM1**	Osteoclastogenesis Associated Transmembrane Protein 1	Protein Coding	38	GC06M108041	2.46	https://www.genecards.org/cgi-bin/carddisp.pl?gene=OSTM1
**RCE1**	Ras Converting CAAX Endopeptidase 1	Protein Coding	38	GC11P066842	2.46	https://www.genecards.org/cgi-bin/carddisp.pl?gene=RCE1
**ZNF23**	Zinc Finger Protein 23	Protein Coding	38	GC16M071463	2.46	https://www.genecards.org/cgi-bin/carddisp.pl?gene=ZNF23
**FXYD1**	FXYD Domain Containing Ion Transport Regulator 1	Protein Coding	37	GC19P038214	2.46	https://www.genecards.org/cgi-bin/carddisp.pl?gene=FXYD1
**CABP4**	Calcium Binding Protein 4	Protein Coding	37	GC11P067453	2.46	https://www.genecards.org/cgi-bin/carddisp.pl?gene=CABP4
**CLEC16A**	C-Type Lectin Domain Containing 16A	Protein Coding	37	GC16P010944	2.46	https://www.genecards.org/cgi-bin/carddisp.pl?gene=CLEC16A

**HES7**	Hes Family BHLH Transcription Factor 7	Protein Coding	37	GC17M008120	2.46	https://www.genecards.org/cgi-bin/carddisp.pl?gene=HES7
**RIPPLY2**	Ripply Transcriptional Repressor 2	Protein Coding	37	GC06P083854	2.46	https://www.genecards.org/cgi-bin/carddisp.pl?gene=RIPPLY2
**CFHR2**	Complement Factor H Related 2	Protein Coding	37	GC01P196943	2.46	https://www.genecards.org/cgi-bin/carddisp.pl?gene=CFHR2
**NAA50**	N-Alpha-Acetyltransferase 50, NatE Catalytic Subunit	Protein Coding	37	GC03M113716	2.46	https://www.genecards.org/cgi-bin/carddisp.pl?gene=NAA50
**GPS1**	G Protein Pathway Suppressor 1	Protein Coding	37	GC17P082050	2.46	https://www.genecards.org/cgi-bin/carddisp.pl?gene=GPS1
**DAZAP2**	DAZ Associated Protein 2	Protein Coding	37	GC12P051238	2.46	https://www.genecards.org/cgi-bin/carddisp.pl?gene=DAZAP2
**SPECC1L**	Sperm Antigen With Calponin Homology And Coiled-Coil Domains 1 Like	Protein Coding	37	GC22P024837	2.46	https://www.genecards.org/cgi-bin/carddisp.pl?gene=SPECC1L
**SIAE**	Sialic Acid Acetylesterase	Protein Coding	37	GC11M124633	2.46	https://www.genecards.org/cgi-bin/carddisp.pl?gene=SIAE

**PAPPA2**	Pappalysin 2	Protein Coding	37	GC01P176463	2.46	https://www.genecards.org/cgi-bin/carddisp.pl?gene=PAPPA2
**IGDCC3**	Immunoglobulin Superfamily DCC Subclass Member 3	Protein Coding	37	GC15M065327	2.46	https://www.genecards.org/cgi-bin/carddisp.pl?gene=IGDCC3
**PACSIN3**	Protein Kinase C And Casein Kinase Substrate In Neurons 3	Protein Coding	37	GC11M061115	2.46	https://www.genecards.org/cgi-bin/carddisp.pl?gene=PACSIN3
**IFT43**	Intraflagellar Transport 43	Protein Coding	37	GC14P075902	2.46	https://www.genecards.org/cgi-bin/carddisp.pl?gene=IFT43
**SCFD1**	Sec1 Family Domain Containing 1	Protein Coding	37	GC14P030622	2.46	https://www.genecards.org/cgi-bin/carddisp.pl?gene=SCFD1
**NT5C1B**	5'-Nucleotidase, Cytosolic IB	Protein Coding	37	GC02M018562	2.46	https://www.genecards.org/cgi-bin/carddisp.pl?gene=NT5C1B
**PGGT1B**	Protein Geranylgeranyltransferase Type I Subunit Beta	Protein Coding	37	GC05M115210	2.46	https://www.genecards.org/cgi-bin/carddisp.pl?gene=PGGT1B
**LCOR**	Ligand Dependent Nuclear Receptor Corepressor	Protein Coding	37	GC10P096832	2.46	https://www.genecards.org/cgi-bin/carddisp.pl?gene=LCOR

**TSR2**	TSR2 Ribosome Maturation Factor	Protein Coding	37	GC0XP054483	2.46	https://www.genecards.org/cgi-bin/carddisp.pl?gene=TSR2
**TAGAP**	T Cell Activation RhoGTPase Activating Protein	Protein Coding	37	GC06M159034	2.46	https://www.genecards.org/cgi-bin/carddisp.pl?gene=TAGAP
**SUN1**	Sad1 And UNC84 Domain Containing 1	Protein Coding	37	GC07P000857	2.46	https://www.genecards.org/cgi-bin/carddisp.pl?gene=SUN1
**SUN2**	Sad1 And UNC84 Domain Containing 2	Protein Coding	37	GC22M045390	2.46	https://www.genecards.org/cgi-bin/carddisp.pl?gene=SUN2
**DNAH8**	Dynein Axonemal Heavy Chain 8	Protein Coding	37	GC06P047481	2.46	https://www.genecards.org/cgi-bin/carddisp.pl?gene=DNAH8
**NAA30**	N-Alpha-Acetyltransferase 30, NatC Catalytic Subunit	Protein Coding	37	GC14P057390	2.46	https://www.genecards.org/cgi-bin/carddisp.pl?gene=NAA30
**SAMM50**	SAMM50 Sorting And Assembly Machinery Component	Protein Coding	37	GC22P043955	2.46	https://www.genecards.org/cgi-bin/carddisp.pl?gene=SAMM50
**TGDS**	TDP-Glucose 4,6-Dehydratase	Protein Coding	37	GC13M094574	2.46	https://www.genecards.org/cgi-bin/carddisp.pl?gene=TGDS

**ZNF354A**	Zinc Finger Protein 354A	Protein Coding	37	GC05M178711	2.46	https://www.genecards.org/cgi-bin/carddisp.pl?gene=ZNF354A
**VSX1**	Visual System Homeobox 1	Protein Coding	37	GC20M025070	2.46	https://www.genecards.org/cgi-bin/carddisp.pl?gene=VSX1
**GATB**	Glutamyl-TRNA Amidotransferase Subunit B	Protein Coding	36	GC04M151670	2.46	https://www.genecards.org/cgi-bin/carddisp.pl?gene=GATB
**GBA3**	Glucosylceramidase Beta 3 (Gene/Pseudogene)	Protein Coding	36	GC04P022694	2.46	https://www.genecards.org/cgi-bin/carddisp.pl?gene=GBA3
**MAGEA3**	MAGE Family Member A3	Protein Coding	36	GC0XP152698	2.46	https://www.genecards.org/cgi-bin/carddisp.pl?gene=MAGEA3
**FBN3**	Fibrillin 3	Protein Coding	36	GC19M008065	2.46	https://www.genecards.org/cgi-bin/carddisp.pl?gene=FBN3
**EPDR1**	Ependymin Related 1	Protein Coding	36	GC07P037688	2.46	https://www.genecards.org/cgi-bin/carddisp.pl?gene=EPDR1
**BLOC1S1**	Biogenesis Of Lysosomal Organelles Complex 1 Subunit 1	Protein Coding	36	GC12P055718	2.46	https://www.genecards.org/cgi-bin/carddisp.pl?gene=BLOC1S1

**COG1**	Component Of Oligomeric Golgi Complex 1	Protein Coding	36	GC17P073193	2.46	https://www.genecards.org/cgi-bin/carddisp.pl?gene=COG1
**PPP1R12C**	Protein Phosphatase 1 Regulatory Subunit 12C	Protein Coding	36	GC19M055110	2.46	https://www.genecards.org/cgi-bin/carddisp.pl?gene=PPP1R12C
**SAA2**	Serum Amyloid A2	Protein Coding	36	GC11M018238	2.46	https://www.genecards.org/cgi-bin/carddisp.pl?gene=SAA2
**PLB1**	Phospholipase B1	Protein Coding	36	GC02P028460	2.46	https://www.genecards.org/cgi-bin/carddisp.pl?gene=PLB1
**LIN54**	Lin-54 DREAM MuvB Core Complex Component	Protein Coding	36	GC04M082909	2.46	https://www.genecards.org/cgi-bin/carddisp.pl?gene=LIN54
**TTI2**	TELO2 Interacting Protein 2	Protein Coding	36	GC08M033473	2.46	https://www.genecards.org/cgi-bin/carddisp.pl?gene=TTI2
**TBL1X**	Transducin Beta Like 1 X-Linked	Protein Coding	36	GC0XP009463	2.46	https://www.genecards.org/cgi-bin/carddisp.pl?gene=TBL1X
**SUPT20H**	SPT20 Homolog, SAGA Complex Component	Protein Coding	36	GC13M037009	2.46	https://www.genecards.org/cgi-bin/carddisp.pl?gene=SUPT20H

**P3H2**	Prolyl 3-Hydroxylase 2	Protein Coding	36	GC03M189956	2.46	https://www.genecards.org/cgi-bin/carddisp.pl?gene=P3H2
**THSD4**	Thrombospondin Type 1 Domain Containing 4	Protein Coding	36	GC15P071096	2.46	https://www.genecards.org/cgi-bin/carddisp.pl?gene=THSD4
**ZNF346**	Zinc Finger Protein 346	Protein Coding	36	GC05P177022	2.46	https://www.genecards.org/cgi-bin/carddisp.pl?gene=ZNF346
**ZSWIM6**	Zinc Finger SWIM-Type Containing 6	Protein Coding	36	GC05P061332	2.46	https://www.genecards.org/cgi-bin/carddisp.pl?gene=ZSWIM6
**VIT**	Vitrin	Protein Coding	36	GC02P036696	2.46	https://www.genecards.org/cgi-bin/carddisp.pl?gene=VIT
**FAM135A**	Family With Sequence Similarity 135 Member A	Protein Coding	35	GC06P070412	2.46	https://www.genecards.org/cgi-bin/carddisp.pl?gene=FAM135A
**MESP2**	Mesoderm Posterior BHLH Transcription Factor 2	Protein Coding	35	GC15P089764	2.46	https://www.genecards.org/cgi-bin/carddisp.pl?gene=MESP2
**MBD6**	Methyl-CpG Binding Domain Protein 6	Protein Coding	35	GC12P057520	2.46	https://www.genecards.org/cgi-bin/carddisp.pl?gene=MBD6

**ADCK2**	AarF Domain Containing Kinase 2	Protein Coding	35	GC07P140672	2.46	https://www.genecards.org/cgi-bin/carddisp.pl?gene=ADCK2
**GPR180**	G Protein-Coupled Receptor 180	Protein Coding	35	GC13P094601	2.46	https://www.genecards.org/cgi-bin/carddisp.pl?gene=GPR180
**CEP350**	Centrosomal Protein 350	Protein Coding	35	GC01P179923	2.46	https://www.genecards.org/cgi-bin/carddisp.pl?gene=CEP350
**HJV**	Hemojuvelin BMP Co-Receptor	Protein Coding	35	GC01M146018	2.46	https://www.genecards.org/cgi-bin/carddisp.pl?gene=HJV
**H2AX**	H2A.X Variant Histone	Protein Coding	35	GC11M119097	2.46	https://www.genecards.org/cgi-bin/carddisp.pl?gene=H2AX
**IFITM5**	Interferon Induced Transmembrane Protein 5	Protein Coding	35	GC11M000298	2.46	https://www.genecards.org/cgi-bin/carddisp.pl?gene=IFITM5
**ONECUT2**	One Cut Homeobox 2	Protein Coding	35	GC18P057436	2.46	https://www.genecards.org/cgi-bin/carddisp.pl?gene=ONECUT2
**SHROOM2**	Shroom Family Member 2	Protein Coding	35	GC0XP009786	2.46	https://www.genecards.org/cgi-bin/carddisp.pl?gene=SHROOM2

**SSPN**	Sarcospan	Protein Coding	35	GC12P026118	2.46	https://www.genecards.org/cgi-bin/carddisp.pl?gene=SSPN
**LACC1**	Laccase Domain Containing 1	Protein Coding	35	GC13P043879	2.46	https://www.genecards.org/cgi-bin/carddisp.pl?gene=LACC1
**SRCIN1**	SRC Kinase Signaling Inhibitor 1	Protein Coding	35	GC17M038530	2.46	https://www.genecards.org/cgi-bin/carddisp.pl?gene=SRCIN1
**ZNF334**	Zinc Finger Protein 334	Protein Coding	35	GC20M046465	2.46	https://www.genecards.org/cgi-bin/carddisp.pl?gene=ZNF334
**ZNF664**	Zinc Finger Protein 664	Protein Coding	35	GC12P123971	2.46	https://www.genecards.org/cgi-bin/carddisp.pl?gene=ZNF664
**YIPF3**	Yip1 Domain Family Member 3	Protein Coding	35	GC06M043479	2.46	https://www.genecards.org/cgi-bin/carddisp.pl?gene=YIPF3
**F8A1**	Coagulation Factor VIII Associated 1	Protein Coding	34	GC0XP154886	2.46	https://www.genecards.org/cgi-bin/carddisp.pl?gene=F8A1
**ATAT1**	Alpha Tubulin Acetyltransferase 1	Protein Coding	34	GC06P030626	2.46	https://www.genecards.org/cgi-bin/carddisp.pl?gene=ATAT1

**ALX3**	ALX Homeobox 3	Protein Coding	34	GC01M110059	2.46	https://www.genecards.org/cgi-bin/carddisp.pl?gene=ALX3
**EPGN**	Epithelial Mitogen	Protein Coding	34	GC04P074309	2.46	https://www.genecards.org/cgi-bin/carddisp.pl?gene=EPGN
**PDF**	Peptide Deformylase, Mitochondrial	Protein Coding	34	GC16M069563	2.46	https://www.genecards.org/cgi-bin/carddisp.pl?gene=PDF
**TMEM222**	Transmembrane Protein 222	Protein Coding	34	GC01P027326	2.46	https://www.genecards.org/cgi-bin/carddisp.pl?gene=TMEM222
**NARS1**	Asparaginyl-TRNA Synthetase 1	Protein Coding	34	GC18M057601	2.46	https://www.genecards.org/cgi-bin/carddisp.pl?gene=NARS1
**SYNE3**	Spectrin Repeat Containing Nuclear Envelope Family Member 3	Protein Coding	34	GC14M095408	2.46	https://www.genecards.org/cgi-bin/carddisp.pl?gene=SYNE3
**ZNF469**	Zinc Finger Protein 469	Protein Coding	34	GC16P088382	2.46	https://www.genecards.org/cgi-bin/carddisp.pl?gene=ZNF469
**FAM102A**	Family With Sequence Similarity 102 Member A	Protein Coding	33	GC09M127941	2.46	https://www.genecards.org/cgi-bin/carddisp.pl?gene=FAM102A

**MAGEC1**	MAGE Family Member C1	Protein Coding	33	GC0XP141905	2.46	https://www.genecards.org/cgi-bin/carddisp.pl?gene=MAGEC1
**CNTD1**	Cyclin N-Terminal Domain Containing 1	Protein Coding	33	GC17P042798	2.46	https://www.genecards.org/cgi-bin/carddisp.pl?gene=CNTD1
**LAMP5**	Lysosomal Associated Membrane Protein Family Member 5	Protein Coding	33	GC20P009514	2.46	https://www.genecards.org/cgi-bin/carddisp.pl?gene=LAMP5
**LARP6**	La Ribonucleoprotein 6, Translational Regulator	Protein Coding	33	GC15M070829	2.46	https://www.genecards.org/cgi-bin/carddisp.pl?gene=LARP6
**KIAA0895**	KIAA0895	Protein Coding	33	GC07M036324	2.46	https://www.genecards.org/cgi-bin/carddisp.pl?gene=KIAA0895
**ADIRF**	Adipogenesis Regulatory Factor	Protein Coding	32	GC10P086968	2.46	https://www.genecards.org/cgi-bin/carddisp.pl?gene=ADIRF
**CARS1**	Cysteinyl-TRNA Synthetase 1	Protein Coding	32	GC11M003000	2.46	https://www.genecards.org/cgi-bin/carddisp.pl?gene=CARS1
**FAM189B**	Family With Sequence Similarity 189 Member B	Protein Coding	32	GC01M155248	2.46	https://www.genecards.org/cgi-bin/carddisp.pl?gene=FAM189B

**ANKAR**	Ankyrin And Armadillo Repeat Containing	Protein Coding	32	GC02P189674	2.46	https://www.genecards.org/cgi-bin/carddisp.pl?gene=ANKAR
**CTAG1B**	Cancer/Testis Antigen 1B	Protein Coding	32	GC0XM154617	2.46	https://www.genecards.org/cgi-bin/carddisp.pl?gene=CTAG1B
**MT-CO1**	Mitochondrially Encoded Cytochrome C Oxidase I	Protein Coding	32	GCMTP005906	2.46	https://www.genecards.org/cgi-bin/carddisp.pl?gene=MT-CO1
**OR2D2**	Olfactory Receptor Family 2 Subfamily D Member 2	Protein Coding	32	GC11M006869	2.46	https://www.genecards.org/cgi-bin/carddisp.pl?gene=OR2D2
**OFCC1**	Orofacial Cleft 1 Candidate 1	Protein Coding	32	GC06M009596	2.46	https://www.genecards.org/cgi-bin/carddisp.pl?gene=OFCC1
**SYNE4**	Spectrin Repeat Containing Nuclear Envelope Family Member 4	Protein Coding	32	GC19M036003	2.46	https://www.genecards.org/cgi-bin/carddisp.pl?gene=SYNE4
**P3H4**	Prolyl 3-Hydroxylase Family Member 4 (Inactive)	Protein Coding	32	GC17M041801	2.46	https://www.genecards.org/cgi-bin/carddisp.pl?gene=P3H4
**RBM33**	RNA Binding Motif Protein 33	Protein Coding	32	GC07P155644	2.46	https://www.genecards.org/cgi-bin/carddisp.pl?gene=RBM33

**IGLL5**	Immunoglobulin Lambda Like Polypeptide 5	Protein Coding	31	GC22P024570	2.46	https://www.genecards.org/cgi-bin/carddisp.pl?gene=IGLL5
**STH**	Saitohin	Protein Coding	30	GC17P045999	2.46	https://www.genecards.org/cgi-bin/carddisp.pl?gene=STH
**MT-CO3**	Mitochondrially Encoded Cytochrome C Oxidase III	Protein Coding	30	GCMTP009209	2.46	https://www.genecards.org/cgi-bin/carddisp.pl?gene=MT-CO3
**LRMDA**	Leucine Rich Melanocyte Differentiation Associated	Protein Coding	29	GC10P075432	2.46	https://www.genecards.org/cgi-bin/carddisp.pl?gene=LRMDA
**CCDC154**	Coiled-Coil Domain Containing 154	Protein Coding	29	GC16M001434	2.46	https://www.genecards.org/cgi-bin/carddisp.pl?gene=CCDC154
**RIPPLY1**	Ripply Transcriptional Repressor 1	Protein Coding	29	GC0XM106900	2.46	https://www.genecards.org/cgi-bin/carddisp.pl?gene=RIPPLY1
**TMSB15A**	Thymosin Beta 15a	Protein Coding	29	GC0XM102515	2.46	https://www.genecards.org/cgi-bin/carddisp.pl?gene=TMSB15A
**CTAG1A**	Cancer/Testis Antigen 1A	Protein Coding	28	GC0XP154585	2.46	https://www.genecards.org/cgi-bin/carddisp.pl?gene=CTAG1A

**TENT5C**	Terminal Nucleotidyltransferase 5C	Protein Coding	28	GC01P117606	2.46	https://www.genecards.org/cgi-bin/carddisp.pl?gene=TENT5C
**PLPPR3**	Phospholipid Phosphatase Related 3	Protein Coding	27	GC19M000814	2.46	https://www.genecards.org/cgi-bin/carddisp.pl?gene=PLPPR3
**KRTAP4-11**	Keratin Associated Protein 4-11	Protein Coding	27	GC17M041117	2.46	https://www.genecards.org/cgi-bin/carddisp.pl?gene=KRTAP4-11
**KCNJ18**	Potassium Inwardly Rectifying Channel Subfamily J Member 18	Protein Coding	26	GC17P026827	2.46	https://www.genecards.org/cgi-bin/carddisp.pl?gene=KCNJ18
**DIRC1**	Disrupted In Renal Carcinoma 1	RNA Gene	25	GC02M188733	2.46	https://www.genecards.org/cgi-bin/carddisp.pl?gene=DIRC1
**FRG2C**	FSHD Region Gene 2 Family Member C	Protein Coding	24	GC03P075646	2.46	https://www.genecards.org/cgi-bin/carddisp.pl?gene=FRG2C
**LEXM**	Lymphocyte Expansion Molecule	Protein Coding	24	GC01P054807	2.46	https://www.genecards.org/cgi-bin/carddisp.pl?gene=LEXM
**NEMP2**	Nuclear Envelope Integral Membrane Protein 2	Protein Coding	23	GC02M190455	2.46	https://www.genecards.org/cgi-bin/carddisp.pl?gene=NEMP2

**TP73-AS1**	TP73 Antisense RNA 1	RNA Gene	22	GC01M003 735	2.46	https://www.genecards.org/cgi-bin/carddisp.pl?gene=TP73-AS1
**MIR125A**	MicroRNA 125a	RNA Gene	21	GC19P051 720	2.46	https://www.genecards.org/cgi-bin/carddisp.pl?gene=MIR125A
**MIR135A1**	MicroRNA 135a-1	RNA Gene	20	GC03M052 296	2.46	https://www.genecards.org/cgi-bin/carddisp.pl?gene=MIR135A1
**MIRLET7B**	MicroRNA Let-7b	RNA Gene	20	GC22P046 119	2.46	https://www.genecards.org/cgi-bin/carddisp.pl?gene=MIRLET7B
**MIR196A1**	MicroRNA 196a-1	RNA Gene	19	GC17M048 632	2.46	https://www.genecards.org/cgi-bin/carddisp.pl?gene=MIR196A1
**MIR342**	MicroRNA 342	RNA Gene	19	GC14P100 109	2.46	https://www.genecards.org/cgi-bin/carddisp.pl?gene=MIR342
**MIR28**	MicroRNA 28	RNA Gene	19	GC03P188 688	2.46	https://www.genecards.org/cgi-bin/carddisp.pl?gene=MIR28
**FBXL19-AS1**	FBXL19 Antisense RNA 1	RNA Gene	19	GC16M030 932	2.46	https://www.genecards.org/cgi-bin/carddisp.pl?gene=FBXL19-AS1

**MIR331**	MicroRNA 331	RNA Gene	18	GC12P095308	2.46	https://www.genecards.org/cgi-bin/carddisp.pl?gene=MIR331
**MIR99B**	MicroRNA 99b	RNA Gene	18	GC19P051692	2.46	https://www.genecards.org/cgi-bin/carddisp.pl?gene=MIR99B
**SNHG28**	Small Nucleolar RNA Host Gene 28	RNA Gene	18	GC01M159834	2.46	https://www.genecards.org/cgi-bin/carddisp.pl?gene=SNHG28
**SNHG29**	Small Nucleolar RNA Host Gene 29	RNA Gene	18	GC17P016469	2.46	https://www.genecards.org/cgi-bin/carddisp.pl?gene=SNHG29
**MIR485**	MicroRNA 485	RNA Gene	17	GC14P104810	2.46	https://www.genecards.org/cgi-bin/carddisp.pl?gene=MIR485
**RNU105C**	RNA, U105C Small Nucleolar	RNA Gene	17	GC08P054330	2.46	https://www.genecards.org/cgi-bin/carddisp.pl?gene=RNU105C
**PCAT1**	Prostate Cancer Associated Transcript 1	RNA Gene	17	GC08P126553	2.46	https://www.genecards.org/cgi-bin/carddisp.pl?gene=PCAT1
**MIR561**	MicroRNA 561	RNA Gene	16	GC02P188297	2.46	https://www.genecards.org/cgi-bin/carddisp.pl?gene=MIR561

**SCARNA22**	Small Cajal Body-Specific RNA 22	RNA Gene	16	GC04P001981	2.46	https://www.genecards.org/cgi-bin/carddisp.pl?gene=SCARNA22
**MIR363**	MicroRNA 363	RNA Gene	15	GC0XM134205	2.46	https://www.genecards.org/cgi-bin/carddisp.pl?gene=MIR363
**ST3GAL6-AS1**	ST3GAL6 Antisense RNA 1	RNA Gene	15	GC03M098714	2.46	https://www.genecards.org/cgi-bin/carddisp.pl?gene=ST3GAL6-AS1
**CCAT1**	Colon Cancer Associated Transcript 1	RNA Gene	14	GC08M127207	2.46	https://www.genecards.org/cgi-bin/carddisp.pl?gene=CCAT1
**HCG14**	HLA Complex Group 14	RNA Gene	14	GC06P028896	2.46	https://www.genecards.org/cgi-bin/carddisp.pl?gene=HCG14
**LAMA5-AS1**	LAMA5 Antisense RNA 1	RNA Gene	13	GC20P062352	2.46	https://www.genecards.org/cgi-bin/carddisp.pl?gene=LAMA5-AS1
**PDIA3P1**	Protein Disulfide Isomerase Family A Member 3 Pseudogene 1	Pseudogene	12	GC01P147182	2.46	https://www.genecards.org/cgi-bin/carddisp.pl?gene=PDIA3P1
**MIR4497**	MicroRNA 4497	RNA Gene	11	GC12P109833	2.46	https://www.genecards.org/cgi-bin/carddisp.pl?gene=MIR4497

**LOC100130744**	Uncharacterized LOC100130744	RNA Gene	8	GC05P014856	2.46	https://www.genecards.org/cgi-bin/carddisp.pl?gene=LOC100130744
**IGHV4-38-2**	Immunoglobulin Heavy Variable 4-38-2	Protein Coding	8	GC14U901616	2.46	https://www.genecards.org/cgi-bin/carddisp.pl?gene=IGHV4-38-2
**GPR143P**	G Protein-Coupled Receptor 143 Pseudogene	Pseudogene	6	GC0YM007101	2.46	https://www.genecards.org/cgi-bin/carddisp.pl?gene=GPR143P
**CNC2**	Carney Complex Type 2, Multiple Neoplasia And Lentiginosis	Genetic Locus	4	GC02U990267	2.46	https://www.genecards.org/cgi-bin/carddisp.pl?gene=CNC2
**OASD**	Ocular Albinism And Sensorineural Deafness	Genetic Locus	3	GC0XU990171	2.46	https://www.genecards.org/cgi-bin/carddisp.pl?gene=OASD
**OA3**	Ocular Albinism 3 (Autosomal Recessive)	Uncategorized	2	GC06U990090	2.46	https://www.genecards.org/cgi-bin/carddisp.pl?gene=OA3
**LOC106050102**	IDS Recombination Region	Biological Region	1	GC0XP149488	2.46	https://www.genecards.org/cgi-bin/carddisp.pl?gene=LOC106050102
**LOC106050103**	IDSP1 Recombination Region	Biological Region	1	GC0XP149526	2.46	https://www.genecards.org/cgi-bin/carddisp.pl?gene=LOC106050103

**LOC106146143**	Int1h-1 Recombination Region	Biological Region	1	GC0XP155006	2.46	https://www.genecards.org/cgi-bin/carddisp.pl?gene=LOC106146143
**LOC106146144**	Int1h-2 Recombination Region	Biological Region	1	GC0XP155147	2.46	https://www.genecards.org/cgi-bin/carddisp.pl?gene=LOC106146144
**LOC106146150**	Int22h-1 Recombination Region	Biological Region	1	GC0XP154880	2.46	https://www.genecards.org/cgi-bin/carddisp.pl?gene=LOC106146150
**LOC106146152**	Int22h-3 Recombination Region	Biological Region	1	GC0XP155454	2.46	https://www.genecards.org/cgi-bin/carddisp.pl?gene=LOC106146152
**LOC106627981**	GBA Recombination Region	Biological Region	1	GC01P155233	2.46	https://www.genecards.org/cgi-bin/carddisp.pl?gene=LOC106627981
**LOC106627982**	GBAP1 Recombination Region	Biological Region	1	GC01P155224	2.46	https://www.genecards.org/cgi-bin/carddisp.pl?gene=LOC106627982
**TIMP3**	TIMP Metallopeptidase Inhibitor 3	Protein Coding	45	GC22P032800	2.45	https://www.genecards.org/cgi-bin/carddisp.pl?gene=TIMP3
**MIR34A**	MicroRNA 34a	RNA Gene	22	GC01M009151	2.44	https://www.genecards.org/cgi-bin/carddisp.pl?gene=MIR34A

**IFRD1**	Interferon Related Developmental Regulator 1	Protein Coding	40	GC07P112 422	2.44	https://www.genecards.org/cgi-bin/carddisp.pl?gene=IFRD1
**PBRM1**	Polybromo 1	Protein Coding	44	GC03M052 545	2.37	https://www.genecards.org/cgi-bin/carddisp.pl?gene=PBRM1
**CDC5L**	Cell Division Cycle 5 Like	Protein Coding	39	GC06P044 387	2.37	https://www.genecards.org/cgi-bin/carddisp.pl?gene=CDC5L
**DIO2**	Iodothyronine Deiodinase 2	Protein Coding	40	GC14M080 197	2.37	https://www.genecards.org/cgi-bin/carddisp.pl?gene=DIO2
**CHADL**	Chondroadherin Like	Protein Coding	35	GC22M045 407	2.34	https://www.genecards.org/cgi-bin/carddisp.pl?gene=CHADL
**IL4R**	Interleukin 4 Receptor	Protein Coding	47	GC16P027 325	2.33	https://www.genecards.org/cgi-bin/carddisp.pl?gene=IL4R
**S100A8**	S100 Calcium Binding Protein A8	Protein Coding	42	GC01M153 391	2.27	https://www.genecards.org/cgi-bin/carddisp.pl?gene=S100A8
**RHOB**	Ras Homolog Family Member B	Protein Coding	44	GC02P020 447	2.24	https://www.genecards.org/cgi-bin/carddisp.pl?gene=RHOB

**VIM**	Vimentin	Protein Coding	50	GC10P017 227	2.23	https://www.genecards.org/cgi-bin/carddisp.pl?gene=VIM
**CCN4**	Cellular Communication Network Factor 4	Protein Coding	31	GC08P133 192	2.23	https://www.genecards.org/cgi-bin/carddisp.pl?gene=CCN4
**MMP7**	Matrix Metallopeptidase 7	Protein Coding	48	GC11M102 425	2.18	https://www.genecards.org/cgi-bin/carddisp.pl?gene=MMP7
**BDKRB2**	Bradykinin Receptor B2	Protein Coding	44	GC14P096 205	2.16	https://www.genecards.org/cgi-bin/carddisp.pl?gene=BDKRB2
**NME8**	NME/NM23 Family Member 8	Protein Coding	40	GC07P037 889	2.15	https://www.genecards.org/cgi-bin/carddisp.pl?gene=NME8
**GSR**	Glutathione-Disulfide Reductase	Protein Coding	48	GC08M030 678	2.13	https://www.genecards.org/cgi-bin/carddisp.pl?gene=GSR
**HDAC9**	Histone Deacetylase 9	Protein Coding	46	GC07P018 086	2.13	https://www.genecards.org/cgi-bin/carddisp.pl?gene=HDAC9
**ERG**	ETS Transcription Factor ERG	Protein Coding	45	GC21M038 367	2.13	https://www.genecards.org/cgi-bin/carddisp.pl?gene=ERG
**NEAT1**	Nuclear Paraspeckle Assembly Transcript 1	RNA Gene	23	GC11P065 794	2.13	https://www.genecards.org/cgi-bin/carddisp.pl?gene=NEAT1

**HDAC4**	Histone Deacetylase 4	Protein Coding	51	GC02M239048	2.09	https://www.genecards.org/cgi-bin/carddisp.pl?gene=HDAC4
**NFE2L2**	Nuclear Factor, Erythroid 2 Like 2	Protein Coding	48	GC02M177227	2.09	https://www.genecards.org/cgi-bin/carddisp.pl?gene=NFE2L2
**PON1**	Paraoxonase 1	Protein Coding	45	GC07M095297	2.09	https://www.genecards.org/cgi-bin/carddisp.pl?gene=PON1
**GLIS3**	GLIS Family Zinc Finger 3	Protein Coding	39	GC09M003816	2.07	https://www.genecards.org/cgi-bin/carddisp.pl?gene=GLIS3
**APOA1**	Apolipoprotein A1	Protein Coding	48	GC11M116835	2.04	https://www.genecards.org/cgi-bin/carddisp.pl?gene=APOA1
**PTN**	Pleiotrophin	Protein Coding	43	GC07M137227	2.04	https://www.genecards.org/cgi-bin/carddisp.pl?gene=PTN
**PARP1**	Poly(ADP-Ribose) Polymerase 1	Protein Coding	49	GC01M226360	2.02	https://www.genecards.org/cgi-bin/carddisp.pl?gene=PARP1
**PRKCA**	Protein Kinase C Alpha	Protein Coding	50	GC17P066302	2.01	https://www.genecards.org/cgi-bin/carddisp.pl?gene=PRKCA

**ASTN2**	Astrotactin 2	Protein Coding	36	GC09M116425	2.01	https://www.genecards.org/cgi-bin/carddisp.pl?gene=ASTN2
**HMOX1**	Heme Oxygenase 1	Protein Coding	52	GC22P035380	1.99	https://www.genecards.org/cgi-bin/carddisp.pl?gene=HMOX1
**MIR29A**	MicroRNA 29a	RNA Gene	21	GC07M130876	1.98	https://www.genecards.org/cgi-bin/carddisp.pl?gene=MIR29A
**MIR29B1**	MicroRNA 29b-1	RNA Gene	21	GC07M130877	1.98	https://www.genecards.org/cgi-bin/carddisp.pl?gene=MIR29B1
**CD68**	CD68 Molecule	Protein Coding	40	GC17P007579	1.97	https://www.genecards.org/cgi-bin/carddisp.pl?gene=CD68
**CAMK2B**	Calcium/Calmodulin Dependent Protein Kinase II Beta	Protein Coding	50	GC07M044217	1.96	https://www.genecards.org/cgi-bin/carddisp.pl?gene=CAMK2B
**TGFBR3**	Transforming Growth Factor Beta Receptor 3	Protein Coding	45	GC01M091680	1.96	https://www.genecards.org/cgi-bin/carddisp.pl?gene=TGFBR3
**SLC39A8**	Solute Carrier Family 39 Member 8	Protein Coding	43	GC04M102252	1.96	https://www.genecards.org/cgi-bin/carddisp.pl?gene=SLC39A8

**XRCC4**	X-Ray Repair Cross Complementing 4	Protein Coding	43	GC05P083077	1.96	https://www.genecards.org/cgi-bin/carddisp.pl?gene=XRCC4
**MUS81**	MUS81 Structure-Specific Endonuclease Subunit	Protein Coding	42	GC11P065883	1.96	https://www.genecards.org/cgi-bin/carddisp.pl?gene=MUS81
**CRTC1**	CREB Regulated Transcription Coactivator 1	Protein Coding	42	GC19P023333	1.96	https://www.genecards.org/cgi-bin/carddisp.pl?gene=CRTC1
**PLEC**	Plectin	Protein Coding	42	GC08M143916	1.96	https://www.genecards.org/cgi-bin/carddisp.pl?gene=PLEC
**CSMD1**	CUB And Sushi Multiple Domains 1	Protein Coding	37	GC08M002953	1.96	https://www.genecards.org/cgi-bin/carddisp.pl?gene=CSMD1
**DUS4L**	Dihydrouridine Synthase 4 Like	Protein Coding	36	GC07P107563	1.96	https://www.genecards.org/cgi-bin/carddisp.pl?gene=DUS4L
**DNAH10**	Dynein Axonemal Heavy Chain 10	Protein Coding	35	GC12P123762	1.96	https://www.genecards.org/cgi-bin/carddisp.pl?gene=DNAH10
**SPCS1**	Signal Peptidase Complex Subunit 1	Protein Coding	35	GC03P052821	1.96	https://www.genecards.org/cgi-bin/carddisp.pl?gene=SPCS1

**PARD3B**	Par-3 Family Cell Polarity Regulator Beta	Protein Coding	35	GC02P204545	1.96	https://www.genecards.org/cgi-bin/carddisp.pl?gene=PARD3B
**NACA2**	Nascent Polypeptide Associated Complex Subunit Alpha 2	Protein Coding	34	GC17M061590	1.96	https://www.genecards.org/cgi-bin/carddisp.pl?gene=NACA2
**TMEM167A**	Transmembrane Protein 167A	Protein Coding	32	GC05M083052	1.96	https://www.genecards.org/cgi-bin/carddisp.pl?gene=TMEM167A
**SCARNA18**	Small Cajal Body-Specific RNA 18	RNA Gene	15	GC05M083064	1.96	https://www.genecards.org/cgi-bin/carddisp.pl?gene=SCARNA18
**TSBP1-AS1**	TSBP1 And BTNL2 Antisense RNA 1	RNA Gene	9	GC06P049132	1.96	https://www.genecards.org/cgi-bin/carddisp.pl?gene=TSBP1-AS1
**CDH2**	Cadherin 2	Protein Coding	50	GC18M027950	1.93	https://www.genecards.org/cgi-bin/carddisp.pl?gene=CDH2
**HAS2**	Hyaluronan Synthase 2	Protein Coding	40	GC08M121594	1.92	https://www.genecards.org/cgi-bin/carddisp.pl?gene=HAS2
**GJA1**	Gap Junction Protein Alpha 1	Protein Coding	50	GC06P121436	1.92	https://www.genecards.org/cgi-bin/carddisp.pl?gene=GJA1

**FOXO1**	Forkhead Box O1	Protein Coding	48	GC13M040555	1.92	https://www.genecards.org/cgi-bin/carddisp.pl?gene=FOXO1
**CCR3**	C-C Motif Chemokine Receptor 3	Protein Coding	47	GC03P046227	1.92	https://www.genecards.org/cgi-bin/carddisp.pl?gene=CCR3
**HAVCR2**	Hepatitis A Virus Cellular Receptor 2	Protein Coding	43	GC05M157063	1.92	https://www.genecards.org/cgi-bin/carddisp.pl?gene=HAVCR2
**TNFAIP3**	TNF Alpha Induced Protein 3	Protein Coding	48	GC06P137866	1.91	https://www.genecards.org/cgi-bin/carddisp.pl?gene=TNFAIP3
**CLEC3B**	C-Type Lectin Domain Family 3 Member B	Protein Coding	40	GC03P045357	1.89	https://www.genecards.org/cgi-bin/carddisp.pl?gene=CLEC3B
**PLA2G3**	Phospholipase A2 Group III	Protein Coding	38	GC22M031135	1.85	https://www.genecards.org/cgi-bin/carddisp.pl?gene=PLA2G3
**MIR149**	MicroRNA 149	RNA Gene	21	GC02P240456	1.85	https://www.genecards.org/cgi-bin/carddisp.pl?gene=MIR149
**MIR223**	MicroRNA 223	RNA Gene	21	GC0XP066018	1.85	https://www.genecards.org/cgi-bin/carddisp.pl?gene=MIR223

**POSTN**	Periostin	Protein Coding	43	GC13M037562	1.84	https://www.genecards.org/cgi-bin/carddisp.pl?gene=POSTN
**PLAT**	Plasminogen Activator, Tissue Type	Protein Coding	49	GC08M042174	1.81	https://www.genecards.org/cgi-bin/carddisp.pl?gene=PLAT
**INHBA**	Inhibin Subunit Beta A	Protein Coding	45	GC07M041668	1.81	https://www.genecards.org/cgi-bin/carddisp.pl?gene=INHBA
**IGFBP5**	Insulin Like Growth Factor Binding Protein 5	Protein Coding	43	GC02M216672	1.81	https://www.genecards.org/cgi-bin/carddisp.pl?gene=IGFBP5
**CXCL13**	C-X-C Motif Chemokine Ligand 13	Protein Coding	41	GC04P077511	1.81	https://www.genecards.org/cgi-bin/carddisp.pl?gene=CXCL13
**MIR210**	MicroRNA 210	RNA Gene	21	GC11M000674	1.81	https://www.genecards.org/cgi-bin/carddisp.pl?gene=MIR210
**ITGB1**	Integrin Subunit Beta 1	Protein Coding	50	GC10M032900	1.8	https://www.genecards.org/cgi-bin/carddisp.pl?gene=ITGB1
**LGALS1**	Galectin 1	Protein Coding	43	GC22P037675	1.8	https://www.genecards.org/cgi-bin/carddisp.pl?gene=LGALS1

**NOX4**	NADPH Oxidase 4	Protein Coding	42	GC11M089324	1.8	https://www.genecards.org/cgi-bin/carddisp.pl?gene=NOX4
**CREB1**	CAMP Responsive Element Binding Protein 1	Protein Coding	48	GC02P207529	1.78	https://www.genecards.org/cgi-bin/carddisp.pl?gene=CREB1
**PITX1**	Paired Like Homeodomain 1	Protein Coding	45	GC05M135027	1.78	https://www.genecards.org/cgi-bin/carddisp.pl?gene=PITX1
**LRP6**	LDL Receptor Related Protein 6	Protein Coding	48	GC12M013893	1.78	https://www.genecards.org/cgi-bin/carddisp.pl?gene=LRP6
**SP1**	Sp1 Transcription Factor	Protein Coding	44	GC12P053380	1.78	https://www.genecards.org/cgi-bin/carddisp.pl?gene=SP1
**OLR1**	Oxidized Low Density Lipoprotein Receptor 1	Protein Coding	44	GC12M013864	1.78	https://www.genecards.org/cgi-bin/carddisp.pl?gene=OLR1
**MIR671**	MicroRNA 671	RNA Gene	17	GC07P151238	1.77	https://www.genecards.org/cgi-bin/carddisp.pl?gene=MIR671
**MIR98**	MicroRNA 98	RNA Gene	17	GC0XM053582	1.75	https://www.genecards.org/cgi-bin/carddisp.pl?gene=MIR98

**EDN1**	Endothelin 1	Protein Coding	47	GC06P012 290	1.75	https://www.genecards.org/cgi-bin/carddisp.pl?gene=EDN1
**ADAMTS1**	ADAM Metallopeptidase With Thrombospondin Type 1 Motif 1	Protein Coding	44	GC21M026 835	1.74	https://www.genecards.org/cgi-bin/carddisp.pl?gene=ADAMTS1
**IL21**	Interleukin 21	Protein Coding	43	GC04M122 612	1.73	https://www.genecards.org/cgi-bin/carddisp.pl?gene=IL21
**UCN**	Urocortin	Protein Coding	37	GC02M027 308	1.73	https://www.genecards.org/cgi-bin/carddisp.pl?gene=UCN
**FOXO3**	Forkhead Box O3	Protein Coding	44	GC06P108 559	1.72	https://www.genecards.org/cgi-bin/carddisp.pl?gene=FOXO3
**XDH**	Xanthine Dehydrogenase	Protein Coding	47	GC02M031 294	1.7	https://www.genecards.org/cgi-bin/carddisp.pl?gene=XDH
**IGFBP7**	Insulin Like Growth Factor Binding Protein 7	Protein Coding	45	GC04M057 030	1.7	https://www.genecards.org/cgi-bin/carddisp.pl?gene=IGFBP7
**RARRES2**	Retinoic Acid Receptor Responder 2	Protein Coding	39	GC07M150 333	1.7	https://www.genecards.org/cgi-bin/carddisp.pl?gene=RARRES2
**CLU**	Clusterin	Protein Coding	46	GC08M027 596	1.7	https://www.genecards.org/cgi-bin/carddisp.pl?gene=CLU

**MIR130A**	MicroRNA 130a	RNA Gene	20	GC11P057641	1.69	https://www.genecards.org/cgi-bin/carddisp.pl?gene=MIR130A
**MIR195**	MicroRNA 195	RNA Gene	19	GC17M007018	1.69	https://www.genecards.org/cgi-bin/carddisp.pl?gene=MIR195
**ALOX15**	Arachidonate 15-Lipoxygenase	Protein Coding	44	GC17M004630	1.68	https://www.genecards.org/cgi-bin/carddisp.pl?gene=ALOX15
**WNT5A**	Wnt Family Member 5A	Protein Coding	49	GC03M055465	1.67	https://www.genecards.org/cgi-bin/carddisp.pl?gene=WNT5A
**IL1RAP**	Interleukin 1 Receptor Accessory Protein	Protein Coding	43	GC03P190514	1.64	https://www.genecards.org/cgi-bin/carddisp.pl?gene=IL1RAP
**YAP1**	Yes1 Associated Transcriptional Regulator	Protein Coding	47	GC11P102110	1.64	https://www.genecards.org/cgi-bin/carddisp.pl?gene=YAP1
**IL34**	Interleukin 34	Protein Coding	39	GC16P070613	1.64	https://www.genecards.org/cgi-bin/carddisp.pl?gene=IL34
**TH**	Tyrosine Hydroxylase	Protein Coding	51	GC11M002163	1.64	https://www.genecards.org/cgi-bin/carddisp.pl?gene=TH
**PECAM1**	Platelet And Endothelial Cell Adhesion Molecule 1	Protein Coding	40	GC17M064319	1.62	https://www.genecards.org/cgi-bin/carddisp.pl?gene=PECAM1

**ANPEP**	Alanyl Aminopeptidase, Membrane	Protein Coding	48	GC15M089784	1.62	https://www.genecards.org/cgi-bin/carddisp.pl?gene=ANPEP
**IL1R2**	Interleukin 1 Receptor Type 2	Protein Coding	45	GC02P101991	1.61	https://www.genecards.org/cgi-bin/carddisp.pl?gene=IL1R2
**IL9**	Interleukin 9	Protein Coding	43	GC05M135891	1.61	https://www.genecards.org/cgi-bin/carddisp.pl?gene=IL9
**MAZ**	MYC Associated Zinc Finger Protein	Protein Coding	40	GC16P029806	1.61	https://www.genecards.org/cgi-bin/carddisp.pl?gene=MAZ
**LGALS3**	Galectin 3	Protein Coding	44	GC14P055124	1.6	https://www.genecards.org/cgi-bin/carddisp.pl?gene=LGALS3
**TWIST1**	Twist Family BHLH Transcription Factor 1	Protein Coding	45	GC07M019020	1.57	https://www.genecards.org/cgi-bin/carddisp.pl?gene=TWIST1
**DEAF1**	DEAF1 Transcription Factor	Protein Coding	40	GC11M000644	1.57	https://www.genecards.org/cgi-bin/carddisp.pl?gene=DEAF1
**OLIG3**	Oligodendrocyte Transcription Factor 3	Protein Coding	36	GC06M137492	1.57	https://www.genecards.org/cgi-bin/carddisp.pl?gene=OLIG3

**GPR22**	G Protein-Coupled Receptor 22	Protein Coding	35	GC07P107470	1.57	https://www.genecards.org/cgi-bin/carddisp.pl?gene=GPR22
**CAV1**	Caveolin 1	Protein Coding	48	GC07P116524	1.56	https://www.genecards.org/cgi-bin/carddisp.pl?gene=CAV1
**CXCR2**	C-X-C Motif Chemokine Receptor 2	Protein Coding	48	GC02P218125	1.54	https://www.genecards.org/cgi-bin/carddisp.pl?gene=CXCR2
**ATF2**	Activating Transcription Factor 2	Protein Coding	45	GC02M175072	1.53	https://www.genecards.org/cgi-bin/carddisp.pl?gene=ATF2
**HRH2**	Histamine Receptor H2	Protein Coding	44	GC05P175659	1.53	https://www.genecards.org/cgi-bin/carddisp.pl?gene=HRH2
**PDPN**	Podoplanin	Protein Coding	40	GC01P013583	1.53	https://www.genecards.org/cgi-bin/carddisp.pl?gene=PDPN
**PIK3R1**	Phosphoinositide-3-Kinase Regulatory Subunit 1	Protein Coding	51	GC05P068215	1.52	https://www.genecards.org/cgi-bin/carddisp.pl?gene=PIK3R1
**ACVRL1**	Activin A Receptor Like Type 1	Protein Coding	50	GC12P051906	1.52	https://www.genecards.org/cgi-bin/carddisp.pl?gene=ACVRL1

**LPAR1**	Lysophosphatidic Acid Receptor 1	Protein Coding	45	GC09M110873	1.52	https://www.genecards.org/cgi-bin/carddisp.pl?gene=LPAR1
**TREH**	Trehalase	Protein Coding	43	GC11M118657	1.52	https://www.genecards.org/cgi-bin/carddisp.pl?gene=TREH
**ADCYAP1**	Adenylate Cyclase Activating Polypeptide 1	Protein Coding	41	GC18P000895	1.52	https://www.genecards.org/cgi-bin/carddisp.pl?gene=ADCYAP1
**IL1RL2**	Interleukin 1 Receptor Like 2	Protein Coding	41	GC02P102186	1.52	https://www.genecards.org/cgi-bin/carddisp.pl?gene=IL1RL2
**DANCR**	Differentiation Antagonizing Non-Protein Coding RNA	RNA Gene	21	GC04P052712	1.52	https://www.genecards.org/cgi-bin/carddisp.pl?gene=DANCR
**VIM2P**	Vimentin 2, Pseudogene	Pseudogene	4	GC06M126602	1.52	https://www.genecards.org/cgi-bin/carddisp.pl?gene=VIM2P
**CXCR1**	C-X-C Motif Chemokine Receptor 1	Protein Coding	43	GC02M218162	1.51	https://www.genecards.org/cgi-bin/carddisp.pl?gene=CXCR1
**SOCS1**	Suppressor Of Cytokine Signaling 1	Protein Coding	43	GC16M011255	1.51	https://www.genecards.org/cgi-bin/carddisp.pl?gene=SOCS1

**ADIPOR1**	Adiponectin Receptor 1	Protein Coding	44	GC01M202940	1.49	https://www.genecards.org/cgi-bin/carddisp.pl?gene=ADIPOR1
**WWP2**	WW Domain Containing E3 Ubiquitin Protein Ligase 2	Protein Coding	43	GC16P069796	1.48	https://www.genecards.org/cgi-bin/carddisp.pl?gene=WWP2
**NR1D1**	Nuclear Receptor Subfamily 1 Group D Member 1	Protein Coding	47	GC17M040092	1.48	https://www.genecards.org/cgi-bin/carddisp.pl?gene=NR1D1
**TYMP**	Thymidine Phosphorylase	Protein Coding	46	GC22M050525	1.48	https://www.genecards.org/cgi-bin/carddisp.pl?gene=TYMP
**CRYAB**	Crystallin Alpha B	Protein Coding	45	GC11M111908	1.48	https://www.genecards.org/cgi-bin/carddisp.pl?gene=CRYAB
**SERPINB2**	Serpin Family B Member 2	Protein Coding	44	GC18P063871	1.48	https://www.genecards.org/cgi-bin/carddisp.pl?gene=SERPINB2
**CCL11**	C-C Motif Chemokine Ligand 11	Protein Coding	43	GC17P034285	1.48	https://www.genecards.org/cgi-bin/carddisp.pl?gene=CCL11
**SREBF2**	Sterol Regulatory Element Binding Transcription Factor 2	Protein Coding	43	GC22P041833	1.48	https://www.genecards.org/cgi-bin/carddisp.pl?gene=SREBF2

**ITLN1**	Intelectin 1	Protein Coding	39	GC01M160876	1.48	https://www.genecards.org/cgi-bin/carddisp.pl?gene=ITLN1
**RBFOX1**	RNA Binding Fox-1 Homolog 1	Protein Coding	37	GC16P005240	1.48	https://www.genecards.org/cgi-bin/carddisp.pl?gene=RBFOX1
**XIST**	X Inactive Specific Transcript	RNA Gene	24	GC0XM073820	1.48	https://www.genecards.org/cgi-bin/carddisp.pl?gene=XIST
**MIR204**	MicroRNA 204	RNA Gene	21	GC09M070809	1.48	https://www.genecards.org/cgi-bin/carddisp.pl?gene=MIR204
**MIR26B**	MicroRNA 26b	RNA Gene	21	GC02P218402	1.48	https://www.genecards.org/cgi-bin/carddisp.pl?gene=MIR26B
**MIR27B**	MicroRNA 27b	RNA Gene	21	GC09P095097	1.48	https://www.genecards.org/cgi-bin/carddisp.pl?gene=MIR27B
**FOXD2-AS1**	FOXD2 Adjacent Opposite Strand RNA 1	RNA Gene	16	GC01M047432	1.48	https://www.genecards.org/cgi-bin/carddisp.pl?gene=FOXD2-AS1
**MIR488**	MicroRNA 488	RNA Gene	16	GC01M177029	1.48	https://www.genecards.org/cgi-bin/carddisp.pl?gene=MIR488

**IL33**	Interleukin 33	Protein Coding	40	GC09P006206	1.46	https://www.genecards.org/cgi-bin/carddisp.pl?gene=IL33
**SNORD19**	Small Nucleolar RNA, C/D Box 19	RNA Gene	14	GC03P052819	1.46	https://www.genecards.org/cgi-bin/carddisp.pl?gene=SNORD19
**DPP4**	Dipeptidyl Peptidase 4	Protein Coding	50	GC02M161992	1.45	https://www.genecards.org/cgi-bin/carddisp.pl?gene=DPP4
**MAP2K4**	Mitogen-Activated Protein Kinase Kinase 4	Protein Coding	46	GC17P012020	1.45	https://www.genecards.org/cgi-bin/carddisp.pl?gene=MAP2K4
**CCL21**	C-C Motif Chemokine Ligand 21	Protein Coding	43	GC09M034709	1.45	https://www.genecards.org/cgi-bin/carddisp.pl?gene=CCL21
**LUM**	Lumican	Protein Coding	41	GC12M091102	1.45	https://www.genecards.org/cgi-bin/carddisp.pl?gene=LUM
**MCAM**	Melanoma Cell Adhesion Molecule	Protein Coding	40	GC11M119308	1.45	https://www.genecards.org/cgi-bin/carddisp.pl?gene=MCAM
**ROR2**	Receptor Tyrosine Kinase Like Orphan Receptor 2	Protein Coding	48	GC09M091564	1.44	https://www.genecards.org/cgi-bin/carddisp.pl?gene=ROR2
**SLPI**	Secretory Leukocyte Peptidase Inhibitor	Protein Coding	39	GC20M045252	1.44	https://www.genecards.org/cgi-bin/carddisp.pl?gene=SLPI

**AQP1**	Aquaporin 1 (Colton Blood Group)	Protein Coding	45	GC07P030911	1.44	https://www.genecards.org/cgi-bin/carddisp.pl?gene=AQP1
**NUCB2**	Nucleobindin 2	Protein Coding	38	GC11P017221	1.43	https://www.genecards.org/cgi-bin/carddisp.pl?gene=NUCB2
**HMMR**	Hyaluronan Mediated Motility Receptor	Protein Coding	42	GC05P163480	1.41	https://www.genecards.org/cgi-bin/carddisp.pl?gene=HMMR
**CYP19A1**	Cytochrome P450 Family 19 Subfamily A Member 1	Protein Coding	48	GC15M051208	1.41	https://www.genecards.org/cgi-bin/carddisp.pl?gene=CYP19A1
**PTGER4**	Prostaglandin E Receptor 4	Protein Coding	46	GC05P040679	1.41	https://www.genecards.org/cgi-bin/carddisp.pl?gene=PTGER4
**ANP32A**	Acidic Nuclear Phosphoprotein 32 Family Member A	Protein Coding	43	GC15M068778	1.41	https://www.genecards.org/cgi-bin/carddisp.pl?gene=ANP32A
**GZMA**	Granzyme A	Protein Coding	42	GC05P055102	1.41	https://www.genecards.org/cgi-bin/carddisp.pl?gene=GZMA
**TCF7L1**	Transcription Factor 7 Like 1	Protein Coding	41	GC02P085133	1.41	https://www.genecards.org/cgi-bin/carddisp.pl?gene=TCF7L1

**CHI3L2**	Chitinase 3 Like 2	Protein Coding	39	GC01P111201	1.41	https://www.genecards.org/cgi-bin/carddisp.pl?gene=CHI3L2
**PDCD5**	Programmed Cell Death 5	Protein Coding	39	GC19P032581	1.4	https://www.genecards.org/cgi-bin/carddisp.pl?gene=PDCD5
**BMPR1A**	Bone Morphogenetic Protein Receptor Type 1A	Protein Coding	51	GC10P086756	1.38	https://www.genecards.org/cgi-bin/carddisp.pl?gene=BMPR1A
**BIRC5**	Baculoviral IAP Repeat Containing 5	Protein Coding	47	GC17P078214	1.38	https://www.genecards.org/cgi-bin/carddisp.pl?gene=BIRC5
**ADIPOR2**	Adiponectin Receptor 2	Protein Coding	43	GC12P001670	1.38	https://www.genecards.org/cgi-bin/carddisp.pl?gene=ADIPOR2
**CCL8**	C-C Motif Chemokine Ligand 8	Protein Coding	39	GC17P034319	1.38	https://www.genecards.org/cgi-bin/carddisp.pl?gene=CCL8
**CLEC4A**	C-Type Lectin Domain Family 4 Member A	Protein Coding	37	GC12P008267	1.38	https://www.genecards.org/cgi-bin/carddisp.pl?gene=CLEC4A
**MIR320A**	MicroRNA 320a	RNA Gene	20	GC08M022246	1.38	https://www.genecards.org/cgi-bin/carddisp.pl?gene=MIR320A

**TPSAB1**	Tryptase Alpha/Beta 1	Protein Coding	43	GC16P001 240	1.38	https://www.genecards.org/cgi-bin/carddisp.pl?gene=TPSAB1
**ADAMTS8**	ADAM Metallopeptidase With Thrombospondin Type 1 Motif 8	Protein Coding	39	GC11M130 308	1.38	https://www.genecards.org/cgi-bin/carddisp.pl?gene=ADAMTS8
**MIR454**	MicroRNA 454	RNA Gene	18	GC17M059 137	1.38	https://www.genecards.org/cgi-bin/carddisp.pl?gene=MIR454
**MIR885**	MicroRNA 885	RNA Gene	17	GC03M010 413	1.38	https://www.genecards.org/cgi-bin/carddisp.pl?gene=MIR885
**UGDH**	UDP-Glucose 6-Dehydrogenase	Protein Coding	45	GC04M039 502	1.37	https://www.genecards.org/cgi-bin/carddisp.pl?gene=UGDH
**ALPP**	Alkaline Phosphatase, Placental	Protein Coding	46	GC02P232 378	1.37	https://www.genecards.org/cgi-bin/carddisp.pl?gene=ALPP
**MAP2K6**	Mitogen-Activated Protein Kinase Kinase 6	Protein Coding	47	GC17P069 414	1.37	https://www.genecards.org/cgi-bin/carddisp.pl?gene=MAP2K6
**PRELP**	Proline And Arginine Rich End Leucine Rich Repeat Protein	Protein Coding	40	GC01P203 444	1.37	https://www.genecards.org/cgi-bin/carddisp.pl?gene=PRELP

**CHAD**	Chondroadherin	Protein Coding	39	GC17M050464	1.37	https://www.genecards.org/cgi-bin/carddisp.pl?gene=CHAD
**MYD88**	MYD88 Innate Immune Signal Transduction Adaptor	Protein Coding	50	GC03P038179	1.35	https://www.genecards.org/cgi-bin/carddisp.pl?gene=MYD88
**APLN**	Apelin	Protein Coding	37	GC0XM129645	1.35	https://www.genecards.org/cgi-bin/carddisp.pl?gene=APLN
**GSK3B**	Glycogen Synthase Kinase 3 Beta	Protein Coding	50	GC03M119821	1.34	https://www.genecards.org/cgi-bin/carddisp.pl?gene=GSK3B
**KNG1**	Kininogen 1	Protein Coding	44	GC03P186717	1.34	https://www.genecards.org/cgi-bin/carddisp.pl?gene=KNG1
**HBEGF**	Heparin Binding EGF Like Growth Factor	Protein Coding	43	GC05M140332	1.34	https://www.genecards.org/cgi-bin/carddisp.pl?gene=HBEGF
**PGF**	Placental Growth Factor	Protein Coding	43	GC14M074941	1.34	https://www.genecards.org/cgi-bin/carddisp.pl?gene=PGF
**PCSK6**	Proprotein Convertase Subtilisin/Kexin Type 6	Protein Coding	40	GC15M101297	1.34	https://www.genecards.org/cgi-bin/carddisp.pl?gene=PCSK6

**SOD3**	Superoxide Dismutase 3	Protein Coding	40	GC04P024798	1.34	https://www.genecards.org/cgi-bin/carddisp.pl?gene=SOD3
**KLF2**	Kruppel Like Factor 2	Protein Coding	40	GC19P023268	1.34	https://www.genecards.org/cgi-bin/carddisp.pl?gene=KLF2
**MMP28**	Matrix Metallopeptidase 28	Protein Coding	37	GC17M035756	1.34	https://www.genecards.org/cgi-bin/carddisp.pl?gene=MMP28
**WNT3A**	Wnt Family Member 3A	Protein Coding	47	GC01P228127	1.32	https://www.genecards.org/cgi-bin/carddisp.pl?gene=WNT3A
**SMURF2**	SMAD Specific E3 Ubiquitin Protein Ligase 2	Protein Coding	43	GC17M064542	1.32	https://www.genecards.org/cgi-bin/carddisp.pl?gene=SMURF2
**MIR365A**	MicroRNA 365a	RNA Gene	17	GC16P014309	1.32	https://www.genecards.org/cgi-bin/carddisp.pl?gene=MIR365A
**MIR320C1**	MicroRNA 320c-1	RNA Gene	16	GC18P021683	1.32	https://www.genecards.org/cgi-bin/carddisp.pl?gene=MIR320C1
**NOS1**	Nitric Oxide Synthase 1	Protein Coding	49	GC12M117208	1.32	https://www.genecards.org/cgi-bin/carddisp.pl?gene=NOS1

**NOD1**	Nucleotide Binding Oligomerization Domain Containing 1	Protein Coding	44	GC07M030424	1.31	https://www.genecards.org/cgi-bin/carddisp.pl?gene=NOD1
**AQP9**	Aquaporin 9	Protein Coding	43	GC15P058138	1.31	https://www.genecards.org/cgi-bin/carddisp.pl?gene=AQP9
**MAP1LC3A**	Microtubule Associated Protein 1 Light Chain 3 Alpha	Protein Coding	43	GC20P034546	1.31	https://www.genecards.org/cgi-bin/carddisp.pl?gene=MAP1LC3A
**ETS2**	ETS Proto-Oncogene 2, Transcription Factor	Protein Coding	42	GC21P038805	1.31	https://www.genecards.org/cgi-bin/carddisp.pl?gene=ETS2
**STAB1**	Stabilin 1	Protein Coding	39	GC03P052495	1.31	https://www.genecards.org/cgi-bin/carddisp.pl?gene=STAB1
**SCUBE1**	Signal Peptide, CUB Domain And EGF Like Domain Containing 1	Protein Coding	36	GC22M043197	1.31	https://www.genecards.org/cgi-bin/carddisp.pl?gene=SCUBE1
**TPI1**	Triosephosphate Isomerase 1	Protein Coding	48	GC12P008208	1.31	https://www.genecards.org/cgi-bin/carddisp.pl?gene=TPI1
**NLRP12**	NLR Family Pyrin Domain Containing 12	Protein Coding	44	GC19M053793	1.31	https://www.genecards.org/cgi-bin/carddisp.pl?gene=NLRP12

**FKBP5**	FKBP Prolyl Isomerase 5	Protein Coding	45	GC06M042217	1.3	https://www.genecards.org/cgi-bin/carddisp.pl?gene=FKBP5
**PAPSS2**	3'-Phosphoadenosine 5'-Phosphosulfate Synthase 2	Protein Coding	44	GC10P087659	1.3	https://www.genecards.org/cgi-bin/carddisp.pl?gene=PAPSS2
**JUND**	JunD Proto-Oncogene, AP-1 Transcription Factor Subunit	Protein Coding	41	GC19M018279	1.3	https://www.genecards.org/cgi-bin/carddisp.pl?gene=JUND
**CXCL11**	C-X-C Motif Chemokine Ligand 11	Protein Coding	40	GC04M076033	1.3	https://www.genecards.org/cgi-bin/carddisp.pl?gene=CXCL11
**LOC111365141**	NOS2 5' Regulatory Region	Biological Region	1	GC17P027800	1.3	https://www.genecards.org/cgi-bin/carddisp.pl?gene=LOC111365141
**L3MBTL2**	L3MBTL Histone Methyl-Lysine Binding Protein 2	Protein Coding	36	GC22P041205	1.29	https://www.genecards.org/cgi-bin/carddisp.pl?gene=L3MBTL2
**L13304-025**		RNA Gene	4	GC22M045502	1.29	https://www.genecards.org/cgi-bin/carddisp.pl?gene=L13304-025
**AB372731**		RNA Gene	3	GC22M045464	1.29	https://www.genecards.org/cgi-bin/carddisp.pl?gene=AB372731

**DUSP1**	Dual Specificity Phosphatase 1	Protein Coding	47	GC05M172768	1.29	https://www.genecards.org/cgi-bin/carddisp.pl?gene=DUSP1
**S100B**	S100 Calcium Binding Protein B	Protein Coding	45	GC21M047431	1.29	https://www.genecards.org/cgi-bin/carddisp.pl?gene=S100B
**NGFR**	Nerve Growth Factor Receptor	Protein Coding	45	GC17P049495	1.29	https://www.genecards.org/cgi-bin/carddisp.pl?gene=NGFR
**MMP16**	Matrix Metallopeptidase 16	Protein Coding	44	GC08M088032	1.29	https://www.genecards.org/cgi-bin/carddisp.pl?gene=MMP16
**ARNTL**	Aryl Hydrocarbon Receptor Nuclear Translocator Like	Protein Coding	42	GC11P013276	1.29	https://www.genecards.org/cgi-bin/carddisp.pl?gene=ARNTL
**CSGALNACT1**	Chondroitin Sulfate N-Acetylgalactosaminyltransferase 1	Protein Coding	40	GC08M019404	1.29	https://www.genecards.org/cgi-bin/carddisp.pl?gene=CSGALNACT1
**CCL22**	C-C Motif Chemokine Ligand 22	Protein Coding	38	GC16P057359	1.29	https://www.genecards.org/cgi-bin/carddisp.pl?gene=CCL22
**MIR455**	MicroRNA 455	RNA Gene	18	GC09P114209	1.29	https://www.genecards.org/cgi-bin/carddisp.pl?gene=MIR455

**SDC4**	Syndecan 4	Protein Coding	44	GC20M045325	1.29	https://www.genecards.org/cgi-bin/carddisp.pl?gene=SDC4
**DIO3**	Iodothyronine Deiodinase 3	Protein Coding	40	GC14P104662	1.29	https://www.genecards.org/cgi-bin/carddisp.pl?gene=DIO3
**LNCRNA-ATB**	Long Noncoding RNA Activated By TGF-Beta	RNA Gene	4	GC14P019509	1.29	https://www.genecards.org/cgi-bin/carddisp.pl?gene=LNCRNA-ATB
**ATF3**	Activating Transcription Factor 3	Protein Coding	44	GC01P212565	1.26	https://www.genecards.org/cgi-bin/carddisp.pl?gene=ATF3
**MIR30C1**	MicroRNA 30c-1	RNA Gene	21	GC01P040757	1.26	https://www.genecards.org/cgi-bin/carddisp.pl?gene=MIR30C1
**B3GAT3**	Beta-1,3-Glucuronyltransferase 3	Protein Coding	45	GC11M063429	1.26	https://www.genecards.org/cgi-bin/carddisp.pl?gene=B3GAT3
**AHSG**	Alpha 2-HS Glycoprotein	Protein Coding	44	GC03P186612	1.26	https://www.genecards.org/cgi-bin/carddisp.pl?gene=AHSG
**CST3**	Cystatin C	Protein Coding	44	GC20M023608	1.26	https://www.genecards.org/cgi-bin/carddisp.pl?gene=CST3

**HMGA2**	High Mobility Group AT-Hook 2	Protein Coding	44	GC12P065824	1.26	https://www.genecards.org/cgi-bin/carddisp.pl?gene=HMGA2
**SFRP1**	Secreted Frizzled Related Protein 1	Protein Coding	43	GC08M041238	1.26	https://www.genecards.org/cgi-bin/carddisp.pl?gene=SFRP1
**HMGB2**	High Mobility Group Box 2	Protein Coding	42	GC04M173331	1.26	https://www.genecards.org/cgi-bin/carddisp.pl?gene=HMGB2
**GPD1L**	Glycerol-3-Phosphate Dehydrogenase 1 Like	Protein Coding	42	GC03P032123	1.26	https://www.genecards.org/cgi-bin/carddisp.pl?gene=GPD1L
**CABIN1**	Calcineurin Binding Protein 1	Protein Coding	41	GC22P024011	1.26	https://www.genecards.org/cgi-bin/carddisp.pl?gene=CABIN1
**IL1RL1**	Interleukin 1 Receptor Like 1	Protein Coding	41	GC02P102294	1.26	https://www.genecards.org/cgi-bin/carddisp.pl?gene=IL1RL1
**LGALS8**	Galectin 8	Protein Coding	41	GC01P236518	1.26	https://www.genecards.org/cgi-bin/carddisp.pl?gene=LGALS8
**HYAL2**	Hyaluronidase 2	Protein Coding	41	GC03M050317	1.26	https://www.genecards.org/cgi-bin/carddisp.pl?gene=HYAL2

**MRC2**	Mannose Receptor C Type 2	Protein Coding	40	GC17P062627	1.26	https://www.genecards.org/cgi-bin/carddisp.pl?gene=MRC2
**CIRBP**	Cold Inducible RNA Binding Protein	Protein Coding	38	GC19P001259	1.26	https://www.genecards.org/cgi-bin/carddisp.pl?gene=CIRBP
**CYTL1**	Cytokine Like 1	Protein Coding	33	GC04M005016	1.26	https://www.genecards.org/cgi-bin/carddisp.pl?gene=CYTL1
**CNR2**	Cannabinoid Receptor 2	Protein Coding	45	GC01M023870	1.24	https://www.genecards.org/cgi-bin/carddisp.pl?gene=CNR2
**TIMP4**	TIMP Metallopeptidase Inhibitor 4	Protein Coding	40	GC03M012153	1.24	https://www.genecards.org/cgi-bin/carddisp.pl?gene=TIMP4
**BAG6**	BAG Cochaperone 6	Protein Coding	35	GC06M031639	1.24	https://www.genecards.org/cgi-bin/carddisp.pl?gene=BAG6
**THRA**	Thyroid Hormone Receptor Alpha	Protein Coding	48	GC17P040058	1.24	https://www.genecards.org/cgi-bin/carddisp.pl?gene=THRA
**ATF6**	Activating Transcription Factor 6	Protein Coding	47	GC01P161766	1.24	https://www.genecards.org/cgi-bin/carddisp.pl?gene=ATF6

**KCNJ11**	Potassium Inwardly Rectifying Channel Subfamily J Member 11	Protein Coding	47	GC11M017364	1.24	https://www.genecards.org/cgi-bin/carddisp.pl?gene=KCNJ11
**HMGCR**	3-Hydroxy-3-Methylglutaryl-CoA Reductase	Protein Coding	45	GC05P075336	1.24	https://www.genecards.org/cgi-bin/carddisp.pl?gene=HMGCR
**GLRX**	Glutaredoxin	Protein Coding	44	GC05M095752	1.24	https://www.genecards.org/cgi-bin/carddisp.pl?gene=GLRX
**MUTYH**	MutY DNA Glycosylase	Protein Coding	44	GC01M045329	1.24	https://www.genecards.org/cgi-bin/carddisp.pl?gene=MUTYH
**ORAI1**	ORAI Calcium Release-Activated Calcium Modulator 1	Protein Coding	44	GC12P122835	1.24	https://www.genecards.org/cgi-bin/carddisp.pl?gene=ORAI1
**UTS2**	Urotensin 2	Protein Coding	41	GC01M007843	1.24	https://www.genecards.org/cgi-bin/carddisp.pl?gene=UTS2
**USP33**	Ubiquitin Specific Peptidase 33	Protein Coding	39	GC01M077695	1.24	https://www.genecards.org/cgi-bin/carddisp.pl?gene=USP33
**COL6A4P2**	Collagen Type VI Alpha 4 Pseudogene 2	Pseudogene	12	GC03P130212	1.24	https://www.genecards.org/cgi-bin/carddisp.pl?gene=COL6A4P2

**DNMT3A**	DNA Methyltransferase 3 Alpha	Protein Coding	51	GC02M025228	1.21	https://www.genecards.org/cgi-bin/carddisp.pl?gene=DNMT3A
**NOS3**	Nitric Oxide Synthase 3	Protein Coding	51	GC07P150990	1.21	https://www.genecards.org/cgi-bin/carddisp.pl?gene=NOS3
**MAP3K5**	Mitogen-Activated Protein Kinase Kinase Kinase 5	Protein Coding	47	GC06M136557	1.21	https://www.genecards.org/cgi-bin/carddisp.pl?gene=MAP3K5
**CYBA**	Cytochrome B-245 Alpha Chain	Protein Coding	47	GC16M088643	1.21	https://www.genecards.org/cgi-bin/carddisp.pl?gene=CYBA
**CNR1**	Cannabinoid Receptor 1	Protein Coding	46	GC06M088139	1.21	https://www.genecards.org/cgi-bin/carddisp.pl?gene=CNR1
**PPARGC1A**	PPARG Coactivator 1 Alpha	Protein Coding	46	GC04M023755	1.21	https://www.genecards.org/cgi-bin/carddisp.pl?gene=PPARGC1A
**CCR4**	C-C Motif Chemokine Receptor 4	Protein Coding	45	GC03P032951	1.21	https://www.genecards.org/cgi-bin/carddisp.pl?gene=CCR4
**HRH4**	Histamine Receptor H4	Protein Coding	45	GC18P024460	1.21	https://www.genecards.org/cgi-bin/carddisp.pl?gene=HRH4

**SERPINB5**	Serpin Family B Member 5	Protein Coding	43	GC18P063476	1.21	https://www.genecards.org/cgi-bin/carddisp.pl?gene=SERPINB5
**PTGES3**	Prostaglandin E Synthase 3	Protein Coding	43	GC12M056667	1.21	https://www.genecards.org/cgi-bin/carddisp.pl?gene=PTGES3
**LCN2**	Lipocalin 2	Protein Coding	43	GC09P128149	1.21	https://www.genecards.org/cgi-bin/carddisp.pl?gene=LCN2
**PPBP**	Pro-Platelet Basic Protein	Protein Coding	43	GC04M073986	1.21	https://www.genecards.org/cgi-bin/carddisp.pl?gene=PPBP
**FAP**	Fibroblast Activation Protein Alpha	Protein Coding	42	GC02M162170	1.21	https://www.genecards.org/cgi-bin/carddisp.pl?gene=FAP
**SP3**	Sp3 Transcription Factor	Protein Coding	42	GC02M173882	1.21	https://www.genecards.org/cgi-bin/carddisp.pl?gene=SP3
**SERPINA4**	Serpin Family A Member 4	Protein Coding	41	GC14P094561	1.21	https://www.genecards.org/cgi-bin/carddisp.pl?gene=SERPINA4
**SERPINE2**	Serpin Family E Member 2	Protein Coding	41	GC02M223975	1.21	https://www.genecards.org/cgi-bin/carddisp.pl?gene=SERPINE2
**TFF3**	Trefoil Factor 3	Protein Coding	41	GC21M042311	1.21	https://www.genecards.org/cgi-bin/carddisp.pl?gene=TFF3

**ADAMTS7**	ADAM Metallopeptidase With Thrombospondin Type 1 Motif 7	Protein Coding	40	GC15M078759	1.21	https://www.genecards.org/cgi-bin/carddisp.pl?gene=ADAMTS7
**IL18RAP**	Interleukin 18 Receptor Accessory Protein	Protein Coding	39	GC02P102418	1.21	https://www.genecards.org/cgi-bin/carddisp.pl?gene=IL18RAP
**PITRM1**	Pitrilysin Metallopeptidase 1	Protein Coding	39	GC10M003138	1.21	https://www.genecards.org/cgi-bin/carddisp.pl?gene=PITRM1
**LGALS9**	Galectin 9	Protein Coding	39	GC17P027629	1.21	https://www.genecards.org/cgi-bin/carddisp.pl?gene=LGALS9
**CCL13**	C-C Motif Chemokine Ligand 13	Protein Coding	38	GC17P034356	1.21	https://www.genecards.org/cgi-bin/carddisp.pl?gene=CCL13
**IL27**	Interleukin 27	Protein Coding	38	GC16M028511	1.21	https://www.genecards.org/cgi-bin/carddisp.pl?gene=IL27
**IFNL1**	Interferon Lambda 1	Protein Coding	35	GC19P039296	1.21	https://www.genecards.org/cgi-bin/carddisp.pl?gene=IFNL1
**MIR125B1**	MicroRNA 125b-1	RNA Gene	21	GC11M122100	1.21	https://www.genecards.org/cgi-bin/carddisp.pl?gene=MIR125B1

**MIR125B2**	MicroRNA 125b-2	RNA Gene	21	GC21P016590	1.21	https://www.genecards.org/cgi-bin/carddisp.pl?gene=MIR125B2
**MIR206**	MicroRNA 206	RNA Gene	20	GC06P052144	1.21	https://www.genecards.org/cgi-bin/carddisp.pl?gene=MIR206
**ERVK-18**	Endogenous Retrovirus Group K Member 18	Protein Coding	9	GC01U903332	1.21	https://www.genecards.org/cgi-bin/carddisp.pl?gene=ERVK-18
**PTGER2**	Prostaglandin E Receptor 2	Protein Coding	48	GC14P052314	1.21	https://www.genecards.org/cgi-bin/carddisp.pl?gene=PTGER2
**POR**	Cytochrome P450 Oxidoreductase	Protein Coding	48	GC07P075899	1.21	https://www.genecards.org/cgi-bin/carddisp.pl?gene=POR
**CYP1A1**	Cytochrome P450 Family 1 Subfamily A Member 1	Protein Coding	47	GC15M074719	1.21	https://www.genecards.org/cgi-bin/carddisp.pl?gene=CYP1A1
**TCF4**	Transcription Factor 4	Protein Coding	46	GC18M055222	1.21	https://www.genecards.org/cgi-bin/carddisp.pl?gene=TCF4
**NCALD**	Neurocalcin Delta	Protein Coding	42	GC08M101704	1.21	https://www.genecards.org/cgi-bin/carddisp.pl?gene=NCALD

**HHIP**	Hedgehog Interacting Protein	Protein Coding	41	GC04P144645	1.21	https://www.genecards.org/cgi-bin/carddisp.pl?gene=HHIP
**NRCAM**	Neuronal Cell Adhesion Molecule	Protein Coding	41	GC07M108147	1.21	https://www.genecards.org/cgi-bin/carddisp.pl?gene=NRCAM
**SIK3**	SIK Family Kinase 3	Protein Coding	41	GC11M116843	1.21	https://www.genecards.org/cgi-bin/carddisp.pl?gene=SIK3
**LRG1**	Leucine Rich Alpha-2-Glycoprotein 1	Protein Coding	39	GC19M004538	1.21	https://www.genecards.org/cgi-bin/carddisp.pl?gene=LRG1
**MIR4435-2HG**	MIR4435-2 Host Gene	RNA Gene	17	GC02M111037	1.21	https://www.genecards.org/cgi-bin/carddisp.pl?gene=MIR4435-2HG
**MELTF-AS1**	MELTF Antisense RNA 1	RNA Gene	12	GC03P197000	1.21	https://www.genecards.org/cgi-bin/carddisp.pl?gene=MELTF-AS1
**MIR4435-2**	MicroRNA 4435-2	RNA Gene	12	GC02M111322	1.21	https://www.genecards.org/cgi-bin/carddisp.pl?gene=MIR4435-2
**COL27A1**	Collagen Type XXVII Alpha 1 Chain	Protein Coding	40	GC09P114827	1.21	https://www.genecards.org/cgi-bin/carddisp.pl?gene=COL27A1

**ITIH1**	Inter-Alpha-Trypsin Inhibitor Heavy Chain 1	Protein Coding	39	GC03P052777	1.18	https://www.genecards.org/cgi-bin/carddisp.pl?gene=ITIH1
**ILF3**	Interleukin Enhancer Binding Factor 3	Protein Coding	38	GC19P010625	1.18	https://www.genecards.org/cgi-bin/carddisp.pl?gene=ILF3
**CEACAM4**	CEA Cell Adhesion Molecule 4	Protein Coding	32	GC19M042520	1.18	https://www.genecards.org/cgi-bin/carddisp.pl?gene=CEACAM4
**DSP**	Desmoplakin	Protein Coding	49	GC06P007541	1.18	https://www.genecards.org/cgi-bin/carddisp.pl?gene=DSP
**PRDX5**	Peroxiredoxin 5	Protein Coding	46	GC11P064317	1.18	https://www.genecards.org/cgi-bin/carddisp.pl?gene=PRDX5
**ADM**	Adrenomedullin	Protein Coding	44	GC11P010304	1.18	https://www.genecards.org/cgi-bin/carddisp.pl?gene=ADM
**CSTA**	Cystatin A	Protein Coding	44	GC03P122325	1.18	https://www.genecards.org/cgi-bin/carddisp.pl?gene=CSTA
**TGIF2**	TGFB Induced Factor Homeobox 2	Protein Coding	39	GC20P036573	1.18	https://www.genecards.org/cgi-bin/carddisp.pl?gene=TGIF2
**FOXD1**	Forkhead Box D1	Protein Coding	36	GC05M073444	1.18	https://www.genecards.org/cgi-bin/carddisp.pl?gene=FOXD1

**JAG1**	Jagged Canonical Notch Ligand 1	Protein Coding	50	GC20M010637	1.16	https://www.genecards.org/cgi-bin/carddisp.pl?gene=JAG1
**AHR**	Aryl Hydrocarbon Receptor	Protein Coding	48	GC07P016916	1.16	https://www.genecards.org/cgi-bin/carddisp.pl?gene=AHR
**YY1**	YY1 Transcription Factor	Protein Coding	48	GC14P100238	1.16	https://www.genecards.org/cgi-bin/carddisp.pl?gene=YY1
**GALE**	UDP-Galactose-4-Epimerase	Protein Coding	45	GC01M023795	1.16	https://www.genecards.org/cgi-bin/carddisp.pl?gene=GALE
**SLC17A5**	Solute Carrier Family 17 Member 5	Protein Coding	44	GC06M073593	1.16	https://www.genecards.org/cgi-bin/carddisp.pl?gene=SLC17A5
**KLRB1**	Killer Cell Lectin Like Receptor B1	Protein Coding	41	GC12M013855	1.16	https://www.genecards.org/cgi-bin/carddisp.pl?gene=KLRB1
**WNT16**	Wnt Family Member 16	Protein Coding	41	GC07P121325	1.16	https://www.genecards.org/cgi-bin/carddisp.pl?gene=WNT16
**HIF3A**	Hypoxia Inducible Factor 3 Subunit Alpha	Protein Coding	39	GC19P046297	1.16	https://www.genecards.org/cgi-bin/carddisp.pl?gene=HIF3A
**FIP1L1**	Factor Interacting With PAPOLA And CPSF1	Protein Coding	38	GC04P053386	1.16	https://www.genecards.org/cgi-bin/carddisp.pl?gene=FIP1L1

**HBP1**	HMG-Box Transcription Factor 1	Protein Coding	38	GC07P107168	1.16	https://www.genecards.org/cgi-bin/carddisp.pl?gene=HBP1
**CHRDL2**	Chordin Like 2	Protein Coding	35	GC11M074696	1.16	https://www.genecards.org/cgi-bin/carddisp.pl?gene=CHRDL2
**DCSTAMP**	Dendrocyte Expressed Seven Transmembrane Protein	Protein Coding	35	GC08P104339	1.16	https://www.genecards.org/cgi-bin/carddisp.pl?gene=DCSTAMP
**TENT5A**	Terminal Nucleotidyltransferase 5A	Protein Coding	29	GC06M081495	1.16	https://www.genecards.org/cgi-bin/carddisp.pl?gene=TENT5A
**PRNCR1**	Prostate Cancer Associated Non-Coding RNA 1	RNA Gene	14	GC08P127079	1.16	https://www.genecards.org/cgi-bin/carddisp.pl?gene=PRNCR1
**NR1H3**	Nuclear Receptor Subfamily 1 Group H Member 3	Protein Coding	47	GC11P047248	1.14	https://www.genecards.org/cgi-bin/carddisp.pl?gene=NR1H3
**IL12A**	Interleukin 12A	Protein Coding	44	GC03P159988	1.14	https://www.genecards.org/cgi-bin/carddisp.pl?gene=IL12A
**C5AR1**	Complement C5a Receptor 1	Protein Coding	43	GC19P047290	1.14	https://www.genecards.org/cgi-bin/carddisp.pl?gene=C5AR1

**SLC23A2**	Solute Carrier Family 23 Member 2	Protein Coding	42	GC20M004852	1.14	https://www.genecards.org/cgi-bin/carddisp.pl?gene=SLC23A2
**BBC3**	BCL2 Binding Component 3	Protein Coding	41	GC19M047220	1.14	https://www.genecards.org/cgi-bin/carddisp.pl?gene=BBC3
**GSTM1**	Glutathione S-Transferase Mu 1	Protein Coding	41	GC01P109687	1.14	https://www.genecards.org/cgi-bin/carddisp.pl?gene=GSTM1
**FGF21**	Fibroblast Growth Factor 21	Protein Coding	40	GC19P048766	1.14	https://www.genecards.org/cgi-bin/carddisp.pl?gene=FGF21
**SCG2**	Secretogranin II	Protein Coding	39	GC02M223596	1.14	https://www.genecards.org/cgi-bin/carddisp.pl?gene=SCG2
**GSTT1**	Glutathione S-Transferase Theta 1	Protein Coding	32	GC22Mi00270	1.14	https://www.genecards.org/cgi-bin/carddisp.pl?gene=GSTT1
**MIR29B2**	MicroRNA 29b-2	RNA Gene	18	GC01M207806	1.14	https://www.genecards.org/cgi-bin/carddisp.pl?gene=MIR29B2
**MIR29C**	MicroRNA 29c	RNA Gene	18	GC01M207802	1.14	https://www.genecards.org/cgi-bin/carddisp.pl?gene=MIR29C

**MIR338**	MicroRNA 338	RNA Gene	17	GC17M081126	1.14	https://www.genecards.org/cgi-bin/carddisp.pl?gene=MIR338
**BMPR2**	Bone Morphogenetic Protein Receptor Type 2	Protein Coding	50	GC02P202376	1.1	https://www.genecards.org/cgi-bin/carddisp.pl?gene=BMPR2
**CTSS**	Cathepsin S	Protein Coding	45	GC01M150730	1.1	https://www.genecards.org/cgi-bin/carddisp.pl?gene=CTSS
**MSTN**	Myostatin	Protein Coding	45	GC02M190055	1.1	https://www.genecards.org/cgi-bin/carddisp.pl?gene=MSTN
**ULK1**	Unc-51 Like Autophagy Activating Kinase 1	Protein Coding	45	GC12P131894	1.1	https://www.genecards.org/cgi-bin/carddisp.pl?gene=ULK1
**KPNA2**	Karyopherin Subunit Alpha 2	Protein Coding	45	GC17P068035	1.1	https://www.genecards.org/cgi-bin/carddisp.pl?gene=KPNA2
**XYLT2**	Xylosyltransferase 2	Protein Coding	45	GC17P050347	1.1	https://www.genecards.org/cgi-bin/carddisp.pl?gene=XYLT2
**B4GALT1**	Beta-1,4-Galactosyltransferase 1	Protein Coding	44	GC09M033100	1.1	https://www.genecards.org/cgi-bin/carddisp.pl?gene=B4GALT1

**CS**	Citrate Synthase	Protein Coding	44	GC12M056271	1.1	https://www.genecards.org/cgi-bin/carddisp.pl?gene=CS
**SFTPD**	Surfactant Protein D	Protein Coding	44	GC10M079937	1.1	https://www.genecards.org/cgi-bin/carddisp.pl?gene=SFTPD
**SFTPB**	Surfactant Protein B	Protein Coding	43	GC02M085657	1.1	https://www.genecards.org/cgi-bin/carddisp.pl?gene=SFTPB
**SFTPC**	Surfactant Protein C	Protein Coding	43	GC08P022156	1.1	https://www.genecards.org/cgi-bin/carddisp.pl?gene=SFTPC
**PTH2R**	Parathyroid Hormone 2 Receptor	Protein Coding	43	GC02P208359	1.1	https://www.genecards.org/cgi-bin/carddisp.pl?gene=PTH2R
**DNTT**	DNA Nucleotidylexotransferase	Protein Coding	42	GC10P096304	1.1	https://www.genecards.org/cgi-bin/carddisp.pl?gene=DNTT
**SFTPA1**	Surfactant Protein A1	Protein Coding	41	GC10P084171	1.1	https://www.genecards.org/cgi-bin/carddisp.pl?gene=SFTPA1
**MFAP4**	Microfibril Associated Protein 4	Protein Coding	40	GC17M019383	1.1	https://www.genecards.org/cgi-bin/carddisp.pl?gene=MFAP4

**CMKLR1**	Chemerin Chemokine-Like Receptor 1	Protein Coding	40	GC12M108288	1.1	https://www.genecards.org/cgi-bin/carddisp.pl?gene=CMKLR1
**SLC25A27**	Solute Carrier Family 25 Member 27	Protein Coding	38	GC06P046652	1.1	https://www.genecards.org/cgi-bin/carddisp.pl?gene=SLC25A27
**MIR23A**	MicroRNA 23a	RNA Gene	20	GC19M014011	1.1	https://www.genecards.org/cgi-bin/carddisp.pl?gene=MIR23A
**HOTAIRM1**	HOXA Transcript Antisense RNA, Myeloid-Specific 1	RNA Gene	18	GC07P027095	1.1	https://www.genecards.org/cgi-bin/carddisp.pl?gene=HOTAIRM1
**MIR940**	MicroRNA 940	RNA Gene	15	GC16P002271	1.1	https://www.genecards.org/cgi-bin/carddisp.pl?gene=MIR940
**MIR300**	MicroRNA 300	RNA Gene	13	GC14P104781	1.1	https://www.genecards.org/cgi-bin/carddisp.pl?gene=MIR300
**OAP**	Osteoarthrosis, Precocious	Uncategorized	2	GC11U900760	1.1	https://www.genecards.org/cgi-bin/carddisp.pl?gene=OAP
**LOC106728418**	LEP 5' Regulatory Region	Biological Region	1	GC07P128238	1.1	https://www.genecards.org/cgi-bin/carddisp.pl?gene=LOC106728418
**AR**	Androgen Receptor	Protein Coding	53	GC0XP067544	1.05	https://www.genecards.org/cgi-bin/carddisp.pl?gene=AR

**PPP3CA**	Protein Phosphatase 3 Catalytic Subunit Alpha	Protein Coding	52	GC04M101024	1.05	https://www.genecards.org/cgi-bin/carddisp.pl?gene=PPP3CA
**ESRRB**	Estrogen Related Receptor Beta	Protein Coding	50	GC14P076310	1.05	https://www.genecards.org/cgi-bin/carddisp.pl?gene=ESRRB
**ACTG1**	Actin Gamma 1	Protein Coding	50	GC17M081509	1.05	https://www.genecards.org/cgi-bin/carddisp.pl?gene=ACTG1
**GSTP1**	Glutathione S-Transferase Pi 1	Protein Coding	50	GC11P067583	1.05	https://www.genecards.org/cgi-bin/carddisp.pl?gene=GSTP1
**CYP2D6**	Cytochrome P450 Family 2 Subfamily D Member 6	Protein Coding	48	GC22M042126	1.05	https://www.genecards.org/cgi-bin/carddisp.pl?gene=CYP2D6
**GLB1**	Galactosidase Beta 1	Protein Coding	48	GC03M033013	1.05	https://www.genecards.org/cgi-bin/carddisp.pl?gene=GLB1
**PDPK1**	3-Phosphoinositide Dependent Protein Kinase 1	Protein Coding	48	GC16P002537	1.05	https://www.genecards.org/cgi-bin/carddisp.pl?gene=PDPK1
**TP63**	Tumor Protein P63	Protein Coding	48	GC03P189598	1.05	https://www.genecards.org/cgi-bin/carddisp.pl?gene=TP63

**KEAP1**	Kelch Like ECH Associated Protein 1	Protein Coding	48	GC19M010486	1.05	https://www.genecards.org/cgi-bin/carddisp.pl?gene=KEAP1
**FAAH**	Fatty Acid Amide Hydrolase	Protein Coding	47	GC01P046394	1.05	https://www.genecards.org/cgi-bin/carddisp.pl?gene=FAAH
**CD3D**	CD3d Molecule	Protein Coding	47	GC11M118338	1.05	https://www.genecards.org/cgi-bin/carddisp.pl?gene=CD3D
**PRKAA1**	Protein Kinase AMP-Activated Catalytic Subunit Alpha 1	Protein Coding	47	GC05M040759	1.05	https://www.genecards.org/cgi-bin/carddisp.pl?gene=PRKAA1
**SOX2**	SRY-Box Transcription Factor 2	Protein Coding	47	GC03P181711	1.05	https://www.genecards.org/cgi-bin/carddisp.pl?gene=SOX2
**ENO2**	Enolase 2	Protein Coding	47	GC12P006913	1.05	https://www.genecards.org/cgi-bin/carddisp.pl?gene=ENO2
**PIK3CB**	Phosphatidylinositol-4,5-Bisphosphate 3-Kinase Catalytic Subunit Beta	Protein Coding	47	GC03M138652	1.05	https://www.genecards.org/cgi-bin/carddisp.pl?gene=PIK3CB
**BECN1**	Beclin 1	Protein Coding	46	GC17M042810	1.05	https://www.genecards.org/cgi-bin/carddisp.pl?gene=BECN1

**C5**	Complement C5	Protein Coding	46	GC09M120952	1.05	https://www.genecards.org/cgi-bin/carddisp.pl?gene=C5
**RIPK2**	Receptor Interacting Serine/Threonine Kinase 2	Protein Coding	46	GC08P089758	1.05	https://www.genecards.org/cgi-bin/carddisp.pl?gene=RIPK2
**HCFC1**	Host Cell Factor C1	Protein Coding	46	GC0XM153947	1.05	https://www.genecards.org/cgi-bin/carddisp.pl?gene=HCFC1
**APOB**	Apolipoprotein B	Protein Coding	45	GC02M020956	1.05	https://www.genecards.org/cgi-bin/carddisp.pl?gene=APOB
**MECP2**	Methyl-CpG Binding Protein 2	Protein Coding	45	GC0XM154021	1.05	https://www.genecards.org/cgi-bin/carddisp.pl?gene=MECP2
**HDAC7**	Histone Deacetylase 7	Protein Coding	45	GC12M047782	1.05	https://www.genecards.org/cgi-bin/carddisp.pl?gene=HDAC7
**DVL2**	Dishevelled Segment Polarity Protein 2	Protein Coding	45	GC17M007225	1.05	https://www.genecards.org/cgi-bin/carddisp.pl?gene=DVL2
**PKM**	Pyruvate Kinase M1/2	Protein Coding	45	GC15M072199	1.05	https://www.genecards.org/cgi-bin/carddisp.pl?gene=PKM

**DLL1**	Delta Like Canonical Notch Ligand 1	Protein Coding	45	GC06M170282	1.05	https://www.genecards.org/cgi-bin/carddisp.pl?gene=DLL1
**HYAL1**	Hyaluronidase 1	Protein Coding	45	GC03M050299	1.05	https://www.genecards.org/cgi-bin/carddisp.pl?gene=HYAL1
**TARDBP**	TAR DNA Binding Protein	Protein Coding	45	GC01P011013	1.05	https://www.genecards.org/cgi-bin/carddisp.pl?gene=TARDBP
**CALM2**	Calmodulin 2	Protein Coding	44	GC02M047124	1.05	https://www.genecards.org/cgi-bin/carddisp.pl?gene=CALM2
**ACSL4**	Acyl-CoA Synthetase Long Chain Family Member 4	Protein Coding	44	GC0XM109624	1.05	https://www.genecards.org/cgi-bin/carddisp.pl?gene=ACSL4
**MFGE8**	Milk Fat Globule EGF And Factor V/VIII Domain Containing	Protein Coding	44	GC15M088898	1.05	https://www.genecards.org/cgi-bin/carddisp.pl?gene=MFGE8
**GANAB**	Glucosidase II Alpha Subunit	Protein Coding	44	GC11M063430	1.05	https://www.genecards.org/cgi-bin/carddisp.pl?gene=GANAB
**HLA-G**	Major Histocompatibility Complex, Class I, G	Protein Coding	44	GC06P047256	1.05	https://www.genecards.org/cgi-bin/carddisp.pl?gene=HLA-G

**GPX3**	Glutathione Peroxidase 3	Protein Coding	44	GC05P150997	1.05	https://www.genecards.org/cgi-bin/carddisp.pl?gene=GPX3
**DDIT4**	DNA Damage Inducible Transcript 4	Protein Coding	44	GC10P072273	1.05	https://www.genecards.org/cgi-bin/carddisp.pl?gene=DDIT4
**PFKFB3**	6-Phosphofructo-2-Kinase/Fructose-2,6-Biphosphatase 3	Protein Coding	44	GC10P006144	1.05	https://www.genecards.org/cgi-bin/carddisp.pl?gene=PFKFB3
**SHMT2**	Serine Hydroxymethyltransferase 2	Protein Coding	44	GC12P057229	1.05	https://www.genecards.org/cgi-bin/carddisp.pl?gene=SHMT2
**PDE5A**	Phosphodiesterase 5A	Protein Coding	44	GC04M119494	1.05	https://www.genecards.org/cgi-bin/carddisp.pl?gene=PDE5A
**NFATC4**	Nuclear Factor Of Activated T Cells 4	Protein Coding	44	GC14P024365	1.05	https://www.genecards.org/cgi-bin/carddisp.pl?gene=NFATC4
**NTN1**	Netrin 1	Protein Coding	44	GC17P009021	1.05	https://www.genecards.org/cgi-bin/carddisp.pl?gene=NTN1
**PTPRD**	Protein Tyrosine Phosphatase Receptor Type D	Protein Coding	44	GC09M008307	1.05	https://www.genecards.org/cgi-bin/carddisp.pl?gene=PTPRD

**TRAF2**	TNF Receptor Associated Factor 2	Protein Coding	44	GC09P136881	1.05	https://www.genecards.org/cgi-bin/carddisp.pl?gene=TRAF2
**STT3B**	STT3 Oligosaccharyltransferase Complex Catalytic Subunit B	Protein Coding	44	GC03P031550	1.05	https://www.genecards.org/cgi-bin/carddisp.pl?gene=STT3B
**SULT2A1**	Sulfotransferase Family 2A Member 1	Protein Coding	44	GC19M047870	1.05	https://www.genecards.org/cgi-bin/carddisp.pl?gene=SULT2A1
**PRKAR2B**	Protein Kinase CAMP-Dependent Type II Regulatory Subunit Beta	Protein Coding	44	GC07P107044	1.05	https://www.genecards.org/cgi-bin/carddisp.pl?gene=PRKAR2B
**PDE4A**	Phosphodiesterase 4A	Protein Coding	44	GC19P010416	1.05	https://www.genecards.org/cgi-bin/carddisp.pl?gene=PDE4A
**KLF5**	Kruppel Like Factor 5	Protein Coding	44	GC13P073054	1.05	https://www.genecards.org/cgi-bin/carddisp.pl?gene=KLF5
**TFF1**	Trefoil Factor 1	Protein Coding	44	GC21M042362	1.05	https://www.genecards.org/cgi-bin/carddisp.pl?gene=TFF1
**BAG1**	BAG Cochaperone 1	Protein Coding	43	GC09M033245	1.05	https://www.genecards.org/cgi-bin/carddisp.pl?gene=BAG1

**HES1**	Hes Family BHLH Transcription Factor 1	Protein Coding	43	GC03P194136	1.05	https://www.genecards.org/cgi-bin/carddisp.pl?gene=HES1
**RIPK4**	Receptor Interacting Serine/Threonine Kinase 4	Protein Coding	43	GC21M041739	1.05	https://www.genecards.org/cgi-bin/carddisp.pl?gene=RIPK4
**NCOR2**	Nuclear Receptor Corepressor 2	Protein Coding	43	GC12M124324	1.05	https://www.genecards.org/cgi-bin/carddisp.pl?gene=NCOR2
**CSPG4**	Chondroitin Sulfate Proteoglycan 4	Protein Coding	43	GC15M075674	1.05	https://www.genecards.org/cgi-bin/carddisp.pl?gene=CSPG4
**SPHK2**	Sphingosine Kinase 2	Protein Coding	43	GC19P048619	1.05	https://www.genecards.org/cgi-bin/carddisp.pl?gene=SPHK2
**PDGFC**	Platelet Derived Growth Factor C	Protein Coding	43	GC04M156760	1.05	https://www.genecards.org/cgi-bin/carddisp.pl?gene=PDGFC
**PRDX4**	Peroxiredoxin 4	Protein Coding	43	GC0XP023665	1.05	https://www.genecards.org/cgi-bin/carddisp.pl?gene=PRDX4
**RSPO2**	R-Spondin 2	Protein Coding	43	GC08M107899	1.05	https://www.genecards.org/cgi-bin/carddisp.pl?gene=RSPO2

**PTGES2**	Prostaglandin E Synthase 2	Protein Coding	43	GC09M128120	1.05	https://www.genecards.org/cgi-bin/carddisp.pl?gene=PTGES2
**LGR5**	Leucine Rich Repeat Containing G Protein-Coupled Receptor 5	Protein Coding	43	GC12P071439	1.05	https://www.genecards.org/cgi-bin/carddisp.pl?gene=LGR5
**TAGLN**	Transgelin	Protein Coding	43	GC11P117199	1.05	https://www.genecards.org/cgi-bin/carddisp.pl?gene=TAGLN
**ZFP36L1**	ZFP36 Ring Finger Protein Like 1	Protein Coding	43	GC14M068787	1.05	https://www.genecards.org/cgi-bin/carddisp.pl?gene=ZFP36L1
**CBX4**	Chromobox 4	Protein Coding	42	GC17M079833	1.05	https://www.genecards.org/cgi-bin/carddisp.pl?gene=CBX4
**APOL1**	Apolipoprotein L1	Protein Coding	42	GC22P036253	1.05	https://www.genecards.org/cgi-bin/carddisp.pl?gene=APOL1
**EIF4G2**	Eukaryotic Translation Initiation Factor 4 Gamma 2	Protein Coding	42	GC11M010866	1.05	https://www.genecards.org/cgi-bin/carddisp.pl?gene=EIF4G2
**SECISBP2**	SECIS Binding Protein 2	Protein Coding	42	GC09P089318	1.05	https://www.genecards.org/cgi-bin/carddisp.pl?gene=SECISBP2

**VPS4B**	Vacuolar Protein Sorting 4 Homolog B	Protein Coding	42	GC18M063389	1.05	https://www.genecards.org/cgi-bin/carddisp.pl?gene=VPS4B
**CCL7**	C-C Motif Chemokine Ligand 7	Protein Coding	41	GC17P034270	1.05	https://www.genecards.org/cgi-bin/carddisp.pl?gene=CCL7
**LPA**	Lipoprotein(A)	Protein Coding	41	GC06M160531	1.05	https://www.genecards.org/cgi-bin/carddisp.pl?gene=LPA
**CRLF1**	Cytokine Receptor Like Factor 1	Protein Coding	41	GC19M018572	1.05	https://www.genecards.org/cgi-bin/carddisp.pl?gene=CRLF1
**CHKA**	Choline Kinase Alpha	Protein Coding	41	GC11M068052	1.05	https://www.genecards.org/cgi-bin/carddisp.pl?gene=CHKA
**NOX1**	NADPH Oxidase 1	Protein Coding	41	GC0XM100843	1.05	https://www.genecards.org/cgi-bin/carddisp.pl?gene=NOX1
**SDCBP**	Syndecan Binding Protein	Protein Coding	41	GC08P058539	1.05	https://www.genecards.org/cgi-bin/carddisp.pl?gene=SDCBP
**SMN1**	Survival Of Motor Neuron 1, Telomeric	Protein Coding	41	GC05P070924	1.05	https://www.genecards.org/cgi-bin/carddisp.pl?gene=SMN1

**KLK11**	Kallikrein Related Peptidase 11	Protein Coding	41	GC19M051023	1.05	https://www.genecards.org/cgi-bin/carddisp.pl?gene=KLK11
**SOX11**	SRY-Box Transcription Factor 11	Protein Coding	41	GC02P005703	1.05	https://www.genecards.org/cgi-bin/carddisp.pl?gene=SOX11
**KHDRBS1**	KH RNA Binding Domain Containing, Signal Transduction Associated 1	Protein Coding	41	GC01P031981	1.05	https://www.genecards.org/cgi-bin/carddisp.pl?gene=KHDRBS1
**TFF2**	Trefoil Factor 2	Protein Coding	41	GC21M042346	1.05	https://www.genecards.org/cgi-bin/carddisp.pl?gene=TFF2
**BOK**	BCL2 Family Apoptosis Regulator BOK	Protein Coding	40	GC02P241558	1.05	https://www.genecards.org/cgi-bin/carddisp.pl?gene=BOK
**ABCD2**	ATP Binding Cassette Subfamily D Member 2	Protein Coding	40	GC12M039530	1.05	https://www.genecards.org/cgi-bin/carddisp.pl?gene=ABCD2
**FUT1**	Fucosyltransferase 1 (H Blood Group)	Protein Coding	40	GC19M048748	1.05	https://www.genecards.org/cgi-bin/carddisp.pl?gene=FUT1
**HS6ST2**	Heparan Sulfate 6-O-Sulfotransferase 2	Protein Coding	40	GC0XM132626	1.05	https://www.genecards.org/cgi-bin/carddisp.pl?gene=HS6ST2

**HGFAC**	HGF Activator	Protein Coding	40	GC04P003443	1.05	https://www.genecards.org/cgi-bin/carddisp.pl?gene=HGFAC
**CPEB1**	Cytoplasmic Polyadenylation Element Binding Protein 1	Protein Coding	40	GC15M082543	1.05	https://www.genecards.org/cgi-bin/carddisp.pl?gene=CPEB1
**DGCR8**	DGCR8 Microprocessor Complex Subunit	Protein Coding	40	GC22P020080	1.05	https://www.genecards.org/cgi-bin/carddisp.pl?gene=DGCR8
**DKK 3.00**	Dickkopf WNT Signaling Pathway Inhibitor 3	Protein Coding	40	GC11M011962	1.05	https://www.genecards.org/cgi-bin/carddisp.pl?gene=DKK3
**DPP7**	Dipeptidyl Peptidase 7	Protein Coding	40	GC09M137111	1.05	https://www.genecards.org/cgi-bin/carddisp.pl?gene=DPP7
**IL17RC**	Interleukin 17 Receptor C	Protein Coding	40	GC03P009917	1.05	https://www.genecards.org/cgi-bin/carddisp.pl?gene=IL17RC
**TSC22D3**	TSC22 Domain Family Member 3	Protein Coding	40	GC0XM107713	1.05	https://www.genecards.org/cgi-bin/carddisp.pl?gene=TSC22D3
**UNC5C**	Unc-5 Netrin Receptor C	Protein Coding	40	GC04M095162	1.05	https://www.genecards.org/cgi-bin/carddisp.pl?gene=UNC5C

**CD160**	CD160 Molecule	Protein Coding	39	GC01P145719	1.05	https://www.genecards.org/cgi-bin/carddisp.pl?gene=CD160
**FIS1**	Fission, Mitochondrial 1	Protein Coding	39	GC07M101239	1.05	https://www.genecards.org/cgi-bin/carddisp.pl?gene=FIS1
**DUSP19**	Dual Specificity Phosphatase 19	Protein Coding	39	GC02P183078	1.05	https://www.genecards.org/cgi-bin/carddisp.pl?gene=DUSP19
**DSTN**	Destrin, Actin Depolymerizing Factor	Protein Coding	39	GC20P017550	1.05	https://www.genecards.org/cgi-bin/carddisp.pl?gene=DSTN
**OXA1L**	OXA1L Mitochondrial Inner Membrane Protein	Protein Coding	39	GC14P022766	1.05	https://www.genecards.org/cgi-bin/carddisp.pl?gene=OXA1L
**TUFT1**	Tuftelin 1	Protein Coding	39	GC01P151513	1.05	https://www.genecards.org/cgi-bin/carddisp.pl?gene=TUFT1
**LGR4**	Leucine Rich Repeat Containing G Protein-Coupled Receptor 4	Protein Coding	39	GC11M027345	1.05	https://www.genecards.org/cgi-bin/carddisp.pl?gene=LGR4
**CCL16**	C-C Motif Chemokine Ligand 16	Protein Coding	38	GC17M035976	1.05	https://www.genecards.org/cgi-bin/carddisp.pl?gene=CCL16

**FFAR4**	Free Fatty Acid Receptor 4	Protein Coding	38	GC10P093566	1.05	https://www.genecards.org/cgi-bin/carddisp.pl?gene=FFAR4
**FOXP4**	Forkhead Box P4	Protein Coding	38	GC06P047499	1.05	https://www.genecards.org/cgi-bin/carddisp.pl?gene=FOXP4
**CREB3**	CAMP Responsive Element Binding Protein 3	Protein Coding	38	GC09P035722	1.05	https://www.genecards.org/cgi-bin/carddisp.pl?gene=CREB3
**EN1**	Engrailed Homeobox 1	Protein Coding	38	GC02M118842	1.05	https://www.genecards.org/cgi-bin/carddisp.pl?gene=EN1
**CRTAC1**	Cartilage Acidic Protein 1	Protein Coding	37	GC10M097865	1.05	https://www.genecards.org/cgi-bin/carddisp.pl?gene=CRTAC1
**CKLF**	Chemokine Like Factor	Protein Coding	37	GC16P066552	1.05	https://www.genecards.org/cgi-bin/carddisp.pl?gene=CKLF
**NID2**	Nidogen 2	Protein Coding	37	GC14M052004	1.05	https://www.genecards.org/cgi-bin/carddisp.pl?gene=NID2
**LAG3**	Lymphocyte Activating 3	Protein Coding	37	GC12P008203	1.05	https://www.genecards.org/cgi-bin/carddisp.pl?gene=LAG3

**UNC5B**	Unc-5 Netrin Receptor B	Protein Coding	37	GC10P071212	1.05	https://www.genecards.org/cgi-bin/carddisp.pl?gene=UNC5B
**RACK1**	Receptor For Activated C Kinase 1	Protein Coding	37	GC05M181310	1.05	https://www.genecards.org/cgi-bin/carddisp.pl?gene=RACK1
**SUB1**	SUB1 Regulator Of Transcription	Protein Coding	37	GC05P032533	1.05	https://www.genecards.org/cgi-bin/carddisp.pl?gene=SUB1
**CCL14**	C-C Motif Chemokine Ligand 14	Protein Coding	36	GC17M036610	1.05	https://www.genecards.org/cgi-bin/carddisp.pl?gene=CCL14
**CCAR2**	Cell Cycle And Apoptosis Regulator 2	Protein Coding	36	GC08P022604	1.05	https://www.genecards.org/cgi-bin/carddisp.pl?gene=CCAR2
**BCAP29**	B Cell Receptor Associated Protein 29	Protein Coding	36	GC07P107579	1.05	https://www.genecards.org/cgi-bin/carddisp.pl?gene=BCAP29
**FUT4**	Fucosyltransferase 4	Protein Coding	36	GC11P094544	1.05	https://www.genecards.org/cgi-bin/carddisp.pl?gene=FUT4
**CORT**	Cortistatin	Protein Coding	36	GC01P010449	1.05	https://www.genecards.org/cgi-bin/carddisp.pl?gene=CORT

**NKX3-2**	NK3 Homeobox 2	Protein Coding	36	GC04M013542	1.05	https://www.genecards.org/cgi-bin/carddisp.pl?gene=NKX3-2
**CCL15**	C-C Motif Chemokine Ligand 15	Protein Coding	35	GC17M035996	1.05	https://www.genecards.org/cgi-bin/carddisp.pl?gene=CCL15
**MICAL3**	Microtubule Associated Monooxygenase, Calponin And LIM Domain Containing 3	Protein Coding	35	GC22M017788	1.05	https://www.genecards.org/cgi-bin/carddisp.pl?gene=MICAL3
**CILP2**	Cartilage Intermediate Layer Protein 2	Protein Coding	35	GC19P019538	1.05	https://www.genecards.org/cgi-bin/carddisp.pl?gene=CILP2
**SHROOM1**	Shroom Family Member 1	Protein Coding	35	GC05M132822	1.05	https://www.genecards.org/cgi-bin/carddisp.pl?gene=SHROOM1
**HSH2D**	Hematopoietic SH2 Domain Containing	Protein Coding	35	GC19P023265	1.05	https://www.genecards.org/cgi-bin/carddisp.pl?gene=HSH2D
**AKNA**	AT-Hook Transcription Factor	Protein Coding	34	GC09M114334	1.05	https://www.genecards.org/cgi-bin/carddisp.pl?gene=AKNA
**FNDC5**	Fibronectin Type III Domain Containing 5	Protein Coding	34	GC01M032864	1.05	https://www.genecards.org/cgi-bin/carddisp.pl?gene=FNDC5

**GRINA**	Glutamate Ionotropic Receptor NMDA Type Subunit Associated Protein 1	Protein Coding	34	GC08P143990	1.05	https://www.genecards.org/cgi-bin/carddisp.pl?gene=GRINA
**TM2D3**	TM2 Domain Containing 3	Protein Coding	33	GC15M101621	1.05	https://www.genecards.org/cgi-bin/carddisp.pl?gene=TM2D3
**XCL2**	X-C Motif Chemokine Ligand 2	Protein Coding	31	GC01M168510	1.05	https://www.genecards.org/cgi-bin/carddisp.pl?gene=XCL2
**TUT7**	Terminal Uridylyl Transferase 7	Protein Coding	29	GC09M086288	1.05	https://www.genecards.org/cgi-bin/carddisp.pl?gene=TUT7
**MIR16-1**	MicroRNA 16-1	RNA Gene	21	GC13M050048	1.05	https://www.genecards.org/cgi-bin/carddisp.pl?gene=MIR16-1
**MIR196A2**	MicroRNA 196a-2	RNA Gene	21	GC12P054200	1.05	https://www.genecards.org/cgi-bin/carddisp.pl?gene=MIR196A2
**MIR152**	MicroRNA 152	RNA Gene	20	GC17M048037	1.05	https://www.genecards.org/cgi-bin/carddisp.pl?gene=MIR152
**MIR138-2**	MicroRNA 138-2	RNA Gene	20	GC16P056859	1.05	https://www.genecards.org/cgi-bin/carddisp.pl?gene=MIR138-2

**MIR370**	MicroRNA 370	RNA Gene	20	GC14P104575	1.05	https://www.genecards.org/cgi-bin/carddisp.pl?gene=MIR370
**MIR373**	MicroRNA 373	RNA Gene	20	GC19P054634	1.05	https://www.genecards.org/cgi-bin/carddisp.pl?gene=MIR373
**MIR200B**	MicroRNA 200b	RNA Gene	20	GC01P001167	1.05	https://www.genecards.org/cgi-bin/carddisp.pl?gene=MIR200B
**MIR23B**	MicroRNA 23b	RNA Gene	20	GC09P095085	1.05	https://www.genecards.org/cgi-bin/carddisp.pl?gene=MIR23B
**MIR30B**	MicroRNA 30b	RNA Gene	20	GC08M134800	1.05	https://www.genecards.org/cgi-bin/carddisp.pl?gene=MIR30B
**MIR22**	MicroRNA 22	RNA Gene	20	GC17M001713	1.05	https://www.genecards.org/cgi-bin/carddisp.pl?gene=MIR22
**MIR449A**	MicroRNA 449a	RNA Gene	20	GC05M055171	1.05	https://www.genecards.org/cgi-bin/carddisp.pl?gene=MIR449A
**MIR181C**	MicroRNA 181c	RNA Gene	20	GC19P013876	1.05	https://www.genecards.org/cgi-bin/carddisp.pl?gene=MIR181C

**MIR138-1**	MicroRNA 138-1	RNA Gene	19	GC03P044115	1.05	https://www.genecards.org/cgi-bin/carddisp.pl?gene=MIR138-1
**MIR107**	MicroRNA 107	RNA Gene	19	GC10M089600	1.05	https://www.genecards.org/cgi-bin/carddisp.pl?gene=MIR107
**MIR128-1**	MicroRNA 128-1	RNA Gene	19	GC02P135665	1.05	https://www.genecards.org/cgi-bin/carddisp.pl?gene=MIR128-1
**MIR9-3**	MicroRNA 9-3	RNA Gene	19	GC15P089363	1.05	https://www.genecards.org/cgi-bin/carddisp.pl?gene=MIR9-3
**SNHG5**	Small Nucleolar RNA Host Gene 5	RNA Gene	19	GC06M085650	1.05	https://www.genecards.org/cgi-bin/carddisp.pl?gene=SNHG5
**MIR16-2**	MicroRNA 16-2	RNA Gene	18	GC03P160413	1.05	https://www.genecards.org/cgi-bin/carddisp.pl?gene=MIR16-2
**MIR106A**	MicroRNA 106a	RNA Gene	18	GC0XM134219	1.05	https://www.genecards.org/cgi-bin/carddisp.pl?gene=MIR106A
**MIR211**	MicroRNA 211	RNA Gene	18	GC15M031065	1.05	https://www.genecards.org/cgi-bin/carddisp.pl?gene=MIR211

**MIR24-1**	MicroRNA 24-1	RNA Gene	18	GC09P095086	1.05	https://www.genecards.org/cgi-bin/carddisp.pl?gene=MIR24-1
**MIR24-2**	MicroRNA 24-2	RNA Gene	18	GC19M014008	1.05	https://www.genecards.org/cgi-bin/carddisp.pl?gene=MIR24-2
**MIR301A**	MicroRNA 301a	RNA Gene	18	GC17M059151	1.05	https://www.genecards.org/cgi-bin/carddisp.pl?gene=MIR301A
**MIR324**	MicroRNA 324	RNA Gene	18	GC17M007223	1.05	https://www.genecards.org/cgi-bin/carddisp.pl?gene=MIR324
**MIR9-2**	MicroRNA 9-2	RNA Gene	18	GC05M088666	1.05	https://www.genecards.org/cgi-bin/carddisp.pl?gene=MIR9-2
**MIR337**	MicroRNA 337	RNA Gene	18	GC14P104787	1.05	https://www.genecards.org/cgi-bin/carddisp.pl?gene=MIR337
**MIR33B**	MicroRNA 33b	RNA Gene	18	GC17M017813	1.05	https://www.genecards.org/cgi-bin/carddisp.pl?gene=MIR33B
**MIR451A**	MicroRNA 451a	RNA Gene	17	GC17M028861	1.05	https://www.genecards.org/cgi-bin/carddisp.pl?gene=MIR451A

**MIR381**	MicroRNA 381	RNA Gene	17	GC14P104797	1.05	https://www.genecards.org/cgi-bin/carddisp.pl?gene=MIR381
**MIR361**	MicroRNA 361	RNA Gene	16	GC0XM085903	1.05	https://www.genecards.org/cgi-bin/carddisp.pl?gene=MIR361
**MIR577**	MicroRNA 577	RNA Gene	16	GC04P114656	1.05	https://www.genecards.org/cgi-bin/carddisp.pl?gene=MIR577
**MIR486-1**	MicroRNA 486-1	RNA Gene	16	GC08M041660	1.05	https://www.genecards.org/cgi-bin/carddisp.pl?gene=MIR486-1
**MIR1236**	MicroRNA 1236	RNA Gene	14	GC06M032637	1.05	https://www.genecards.org/cgi-bin/carddisp.pl?gene=MIR1236
**MIR1246**	MicroRNA 1246	RNA Gene	13	GC02M176600	1.05	https://www.genecards.org/cgi-bin/carddisp.pl?gene=MIR1246
**MIR1277**	MicroRNA 1277	RNA Gene	13	GC0XP118386	1.05	https://www.genecards.org/cgi-bin/carddisp.pl?gene=MIR1277
**MIR634**	MicroRNA 634	RNA Gene	13	GC17P066787	1.05	https://www.genecards.org/cgi-bin/carddisp.pl?gene=MIR634

**MIR105-1**	MicroRNA 105-1	RNA Gene	13	GC0XM152392	1.05	https://www.genecards.org/cgi-bin/carddisp.pl?gene=MIR105-1
**TP53COR1**	Tumor Protein P53 Pathway Corepressor 1	RNA Gene	8	GC06U903133	1.05	https://www.genecards.org/cgi-bin/carddisp.pl?gene=TP53COR1
**LOC112543491**	Linc-UFC1	RNA Gene	5	GC01P161141	1.05	https://www.genecards.org/cgi-bin/carddisp.pl?gene=LOC112543491
**HPBP**	Phosphate Binding Apolipoprotein	RNA Gene	2	GC00U922525	1.05	https://www.genecards.org/cgi-bin/carddisp.pl?gene=HPBP
**LOC110283621**	SPP1 5' Regulatory Region	Biological Region	1	GC04P087973	1.05	https://www.genecards.org/cgi-bin/carddisp.pl?gene=LOC110283621
**MAPT**	Microtubule Associated Protein Tau	Protein Coding	50	GC17P045894	1.04	https://www.genecards.org/cgi-bin/carddisp.pl?gene=MAPT
**USP8**	Ubiquitin Specific Peptidase 8	Protein Coding	48	GC15P050424	1.04	https://www.genecards.org/cgi-bin/carddisp.pl?gene=USP8
**ABT1**	Activator Of Basal Transcription 1	Protein Coding	35	GC06P028762	1.04	https://www.genecards.org/cgi-bin/carddisp.pl?gene=ABT1

**MIR499A**	MicroRNA 499a	RNA Gene	21	GC20P034990	1.04	https://www.genecards.org/cgi-bin/carddisp.pl?gene=MIR499A
**CEACAM8**	CEA Cell Adhesion Molecule 8	Protein Coding	37	GC19M042580	1.03	https://www.genecards.org/cgi-bin/carddisp.pl?gene=CEACAM8
**CEBPB**	CCAAT Enhancer Binding Protein Beta	Protein Coding	43	GC20P050190	1.01	https://www.genecards.org/cgi-bin/carddisp.pl?gene=CEBPB
**MAPK10**	Mitogen-Activated Protein Kinase 10	Protein Coding	51	GC04M085990	0.96	https://www.genecards.org/cgi-bin/carddisp.pl?gene=MAPK10
**TNFRSF6B**	TNF Receptor Superfamily Member 6b	Protein Coding	43	GC20P063696	0.93	https://www.genecards.org/cgi-bin/carddisp.pl?gene=TNFRSF6B
**WNT7A**	Wnt Family Member 7A	Protein Coding	49	GC03M015784	0.92	https://www.genecards.org/cgi-bin/carddisp.pl?gene=WNT7A
**SETD2**	SET Domain Containing 2, Histone Lysine Methyltransferase	Protein Coding	47	GC03M047033	0.92	https://www.genecards.org/cgi-bin/carddisp.pl?gene=SETD2
**LDHB**	Lactate Dehydrogenase B	Protein Coding	46	GC12M021635	0.92	https://www.genecards.org/cgi-bin/carddisp.pl?gene=LDHB

**P4HA2**	Prolyl 4-Hydroxylase Subunit Alpha 2	Protein Coding	45	GC05M132191	0.92	https://www.genecards.org/cgi-bin/carddisp.pl?gene=P4HA2
**TNKS**	Tankyrase	Protein Coding	43	GC08P009555	0.92	https://www.genecards.org/cgi-bin/carddisp.pl?gene=TNKS
**SETD1A**	SET Domain Containing 1A, Histone Lysine Methyltransferase	Protein Coding	40	GC16P030980	0.92	https://www.genecards.org/cgi-bin/carddisp.pl?gene=SETD1A
**P4HA1**	Prolyl 4-Hydroxylase Subunit Alpha 1	Protein Coding	40	GC10M073007	0.92	https://www.genecards.org/cgi-bin/carddisp.pl?gene=P4HA1
**HES5**	Hes Family BHLH Transcription Factor 5	Protein Coding	34	GC01M002528	0.92	https://www.genecards.org/cgi-bin/carddisp.pl?gene=HES5
**MIR146B**	MicroRNA 146b	RNA Gene	19	GC10P102436	0.92	https://www.genecards.org/cgi-bin/carddisp.pl?gene=MIR146B
**BHD**	Beukes Familial Hip Dysplasia	Genetic Locus	1	GC00U936447	0.92	https://www.genecards.org/cgi-bin/carddisp.pl?gene=BHD
**PCSK9**	Proprotein Convertase Subtilisin/Kexin Type 9	Protein Coding	51	GC01P055039	0.91	https://www.genecards.org/cgi-bin/carddisp.pl?gene=PCSK9

**NPM1**	Nucleophosmin 1	Protein Coding	50	GC05P171387	0.91	https://www.genecards.org/cgi-bin/carddisp.pl?gene=NPM1
**CYP11A1**	Cytochrome P450 Family 11 Subfamily A Member 1	Protein Coding	48	GC15M074337	0.91	https://www.genecards.org/cgi-bin/carddisp.pl?gene=CYP11A1
**NF1**	Neurofibromin 1	Protein Coding	48	GC17P031094	0.91	https://www.genecards.org/cgi-bin/carddisp.pl?gene=NF1
**SLC25A13**	Solute Carrier Family 25 Member 13	Protein Coding	46	GC07M096120	0.91	https://www.genecards.org/cgi-bin/carddisp.pl?gene=SLC25A13
**GPD2**	Glycerol-3-Phosphate Dehydrogenase 2	Protein Coding	46	GC02P156435	0.91	https://www.genecards.org/cgi-bin/carddisp.pl?gene=GPD2
**KAT5**	Lysine Acetyltransferase 5	Protein Coding	46	GC11P065711	0.91	https://www.genecards.org/cgi-bin/carddisp.pl?gene=KAT5
**AP3B1**	Adaptor Related Protein Complex 3 Subunit Beta 1	Protein Coding	45	GC05M078000	0.91	https://www.genecards.org/cgi-bin/carddisp.pl?gene=AP3B1
**TBX2**	T-Box Transcription Factor 2	Protein Coding	45	GC17P061399	0.91	https://www.genecards.org/cgi-bin/carddisp.pl?gene=TBX2

**HERC2**	HECT And RLD Domain Containing E3 Ubiquitin Protein Ligase 2	Protein Coding	45	GC15M028111	0.91	https://www.genecards.org/cgi-bin/carddisp.pl?gene=HERC2
**ADD1**	Adducin 1	Protein Coding	44	GC04P002855	0.91	https://www.genecards.org/cgi-bin/carddisp.pl?gene=ADD1
**ATP2B1**	ATPase Plasma Membrane Ca2+ Transporting 1	Protein Coding	44	GC12M089588	0.91	https://www.genecards.org/cgi-bin/carddisp.pl?gene=ATP2B1
**GAK**	Cyclin G Associated Kinase	Protein Coding	44	GC04M000849	0.91	https://www.genecards.org/cgi-bin/carddisp.pl?gene=GAK
**CRADD**	CASP2 And RIPK1 Domain Containing Adaptor With Death Domain	Protein Coding	44	GC12P093677	0.91	https://www.genecards.org/cgi-bin/carddisp.pl?gene=CRADD
**CDC7**	Cell Division Cycle 7	Protein Coding	44	GC01P091500	0.91	https://www.genecards.org/cgi-bin/carddisp.pl?gene=CDC7
**PAX7**	Paired Box 7	Protein Coding	44	GC01P018631	0.91	https://www.genecards.org/cgi-bin/carddisp.pl?gene=PAX7
**MMP24**	Matrix Metallopeptidase 24	Protein Coding	43	GC20P035226	0.91	https://www.genecards.org/cgi-bin/carddisp.pl?gene=MMP24

**GRK4**	G Protein-Coupled Receptor Kinase 4	Protein Coding	43	GC04P002963	0.91	https://www.genecards.org/cgi-bin/carddisp.pl?gene=GRK4
**EIF2B1**	Eukaryotic Translation Initiation Factor 2B Subunit Alpha	Protein Coding	43	GC12M123620	0.91	https://www.genecards.org/cgi-bin/carddisp.pl?gene=EIF2B1
**HTT**	Huntingtin	Protein Coding	43	GC04P003041	0.91	https://www.genecards.org/cgi-bin/carddisp.pl?gene=HTT
**SOCS2**	Suppressor Of Cytokine Signaling 2	Protein Coding	43	GC12P093569	0.91	https://www.genecards.org/cgi-bin/carddisp.pl?gene=SOCS2
**SLC39A4**	Solute Carrier Family 39 Member 4	Protein Coding	43	GC08M144409	0.91	https://www.genecards.org/cgi-bin/carddisp.pl?gene=SLC39A4
**LCT**	Lactase	Protein Coding	43	GC02M135787	0.91	https://www.genecards.org/cgi-bin/carddisp.pl?gene=LCT
**TACC3**	Transforming Acidic Coiled-Coil Containing Protein 3	Protein Coding	43	GC04P001723	0.91	https://www.genecards.org/cgi-bin/carddisp.pl?gene=TACC3
**USP24**	Ubiquitin Specific Peptidase 24	Protein Coding	43	GC01M055066	0.91	https://www.genecards.org/cgi-bin/carddisp.pl?gene=USP24
**ANXA3**	Annexin A3	Protein Coding	42	GC04P078551	0.91	https://www.genecards.org/cgi-bin/carddisp.pl?gene=ANXA3

**DPEP1**	Dipeptidase 1	Protein Coding	42	GC16P089613	0.91	https://www.genecards.org/cgi-bin/carddisp.pl?gene=DPEP1
**NFAT5**	Nuclear Factor Of Activated T Cells 5	Protein Coding	42	GC16P069565	0.91	https://www.genecards.org/cgi-bin/carddisp.pl?gene=NFAT5
**SLC30A10**	Solute Carrier Family 30 Member 10	Protein Coding	42	GC01M219685	0.91	https://www.genecards.org/cgi-bin/carddisp.pl?gene=SLC30A10
**ITIH4**	Inter-Alpha-Trypsin Inhibitor Heavy Chain 4	Protein Coding	42	GC03M052812	0.91	https://www.genecards.org/cgi-bin/carddisp.pl?gene=ITIH4
**LTBP3**	Latent Transforming Growth Factor Beta Binding Protein 3	Protein Coding	41	GC11M065538	0.91	https://www.genecards.org/cgi-bin/carddisp.pl?gene=LTBP3
**MCAT**	Malonyl-CoA-Acyl Carrier Protein Transacylase	Protein Coding	41	GC22M043132	0.91	https://www.genecards.org/cgi-bin/carddisp.pl?gene=MCAT
**PAPPA**	Pappalysin 1	Protein Coding	41	GC09P116179	0.91	https://www.genecards.org/cgi-bin/carddisp.pl?gene=PAPPA
**NISCH**	Nischarin	Protein Coding	41	GC03P052455	0.91	https://www.genecards.org/cgi-bin/carddisp.pl?gene=NISCH

**SMYD3**	SET And MYND Domain Containing 3	Protein Coding	41	GC01M245749	0.91	https://www.genecards.org/cgi-bin/carddisp.pl?gene=SMYD3
**SPTBN2**	Spectrin Beta, Non-Erythrocytic 2	Protein Coding	41	GC11M066684	0.91	https://www.genecards.org/cgi-bin/carddisp.pl?gene=SPTBN2
**JPH3**	Junctophilin 3	Protein Coding	41	GC16P087601	0.91	https://www.genecards.org/cgi-bin/carddisp.pl?gene=JPH3
**RASGRP3**	RAS Guanyl Releasing Protein 3	Protein Coding	41	GC02P033436	0.91	https://www.genecards.org/cgi-bin/carddisp.pl?gene=RASGRP3
**TBX4**	T-Box Transcription Factor 4	Protein Coding	41	GC17P061451	0.91	https://www.genecards.org/cgi-bin/carddisp.pl?gene=TBX4
**PUF60**	Poly(U) Binding Splicing Factor 60	Protein Coding	41	GC08M143816	0.91	https://www.genecards.org/cgi-bin/carddisp.pl?gene=PUF60
**CYP2A13**	Cytochrome P450 Family 2 Subfamily A Member 13	Protein Coding	41	GC19P041088	0.91	https://www.genecards.org/cgi-bin/carddisp.pl?gene=CYP2A13
**SCYL1**	SCY1 Like Pseudokinase 1	Protein Coding	41	GC11P065525	0.91	https://www.genecards.org/cgi-bin/carddisp.pl?gene=SCYL1

**PTPRG**	Protein Tyrosine Phosphatase Receptor Type G	Protein Coding	41	GC03P061522	0.91	https://www.genecards.org/cgi-bin/carddisp.pl?gene=PTPRG
**ABCB9**	ATP Binding Cassette Subfamily B Member 9	Protein Coding	40	GC12M122920	0.91	https://www.genecards.org/cgi-bin/carddisp.pl?gene=ABCB9
**MAT2B**	Methionine Adenosyltransferase 2B	Protein Coding	40	GC05P163523	0.91	https://www.genecards.org/cgi-bin/carddisp.pl?gene=MAT2B
**GALNT5**	Polypeptide N-Acetylgalactosaminyltransferase 5	Protein Coding	40	GC02P157258	0.91	https://www.genecards.org/cgi-bin/carddisp.pl?gene=GALNT5
**CACNG5**	Calcium Voltage-Gated Channel Auxiliary Subunit Gamma 5	Protein Coding	40	GC17P066835	0.91	https://www.genecards.org/cgi-bin/carddisp.pl?gene=CACNG5
**B9D2**	B9 Domain Containing 2	Protein Coding	40	GC19M041354	0.91	https://www.genecards.org/cgi-bin/carddisp.pl?gene=B9D2
**FAR2**	Fatty Acyl-CoA Reductase 2	Protein Coding	40	GC12P029145	0.91	https://www.genecards.org/cgi-bin/carddisp.pl?gene=FAR2
**MAGI1**	Membrane Associated Guanylate Kinase, WW And PDZ Domain Containing 1	Protein Coding	40	GC03M065330	0.91	https://www.genecards.org/cgi-bin/carddisp.pl?gene=MAGI1

**AAGAB**	Alpha And Gamma Adaptin Binding Protein	Protein Coding	40	GC15M067200	0.91	https://www.genecards.org/cgi-bin/carddisp.pl?gene=AAGAB
**CEACAM3**	CEA Cell Adhesion Molecule 3	Protein Coding	40	GC19P041796	0.91	https://www.genecards.org/cgi-bin/carddisp.pl?gene=CEACAM3
**CEP250**	Centrosomal Protein 250	Protein Coding	40	GC20P035455	0.91	https://www.genecards.org/cgi-bin/carddisp.pl?gene=CEP250
**RNF4**	Ring Finger Protein 4	Protein Coding	40	GC04P002462	0.91	https://www.genecards.org/cgi-bin/carddisp.pl?gene=RNF4
**NEK4**	NIMA Related Kinase 4	Protein Coding	40	GC03M052708	0.91	https://www.genecards.org/cgi-bin/carddisp.pl?gene=NEK4
**EIF6**	Eukaryotic Translation Initiation Factor 6	Protein Coding	40	GC20M035278	0.91	https://www.genecards.org/cgi-bin/carddisp.pl?gene=EIF6
**HLA-DPA1**	Major Histocompatibility Complex, Class II, DP Alpha 1	Protein Coding	40	GC06M033064	0.91	https://www.genecards.org/cgi-bin/carddisp.pl?gene=HLA-DPA1
**CPNE1**	Copine 1	Protein Coding	40	GC20M035626	0.91	https://www.genecards.org/cgi-bin/carddisp.pl?gene=CPNE1

**SMG6**	SMG6 Nonsense Mediated MRNA Decay Factor	Protein Coding	40	GC17M002059	0.91	https://www.genecards.org/cgi-bin/carddisp.pl?gene=SMG6
**PRDM2**	PR/SET Domain 2	Protein Coding	40	GC01P013702	0.91	https://www.genecards.org/cgi-bin/carddisp.pl?gene=PRDM2
**SCRIB**	Scribble Planar Cell Polarity Protein	Protein Coding	40	GC08M143822	0.91	https://www.genecards.org/cgi-bin/carddisp.pl?gene=SCRIB
**SART1**	Spliceosome Associated Factor 1, Recruiter Of U4/U6.U5 Tri-SnRNP	Protein Coding	40	GC11P065979	0.91	https://www.genecards.org/cgi-bin/carddisp.pl?gene=SART1
**SLC44A2**	Solute Carrier Family 44 Member 2	Protein Coding	40	GC19P010602	0.91	https://www.genecards.org/cgi-bin/carddisp.pl?gene=SLC44A2
**POLA2**	DNA Polymerase Alpha 2, Accessory Subunit	Protein Coding	40	GC11P065279	0.91	https://www.genecards.org/cgi-bin/carddisp.pl?gene=POLA2
**STRA6**	Signaling Receptor And Transporter Of Retinol STRA6	Protein Coding	40	GC15M074179	0.91	https://www.genecards.org/cgi-bin/carddisp.pl?gene=STRA6
**DYNC1I1**	Dynein Cytoplasmic 1 Intermediate Chain 1	Protein Coding	40	GC07P095772	0.91	https://www.genecards.org/cgi-bin/carddisp.pl?gene=DYNC1I1

**SH3BP2**	SH3 Domain Binding Protein 2	Protein Coding	40	GC04P002794	0.91	https://www.genecards.org/cgi-bin/carddisp.pl?gene=SH3BP2
**YKT6**	YKT6 V-SNARE Homolog	Protein Coding	40	GC07P044200	0.91	https://www.genecards.org/cgi-bin/carddisp.pl?gene=YKT6
**GGT7**	Gamma-Glutamyltransferase 7	Protein Coding	39	GC20M034844	0.91	https://www.genecards.org/cgi-bin/carddisp.pl?gene=GGT7
**AGAP1**	ArfGAP With GTPase Domain, Ankyrin Repeat And PH Domain 1	Protein Coding	39	GC02P235494	0.91	https://www.genecards.org/cgi-bin/carddisp.pl?gene=AGAP1
**BMP2K**	BMP2 Inducible Kinase	Protein Coding	39	GC04P078776	0.91	https://www.genecards.org/cgi-bin/carddisp.pl?gene=BMP2K
**CPSF6**	Cleavage And Polyadenylation Specific Factor 6	Protein Coding	39	GC12P069239	0.91	https://www.genecards.org/cgi-bin/carddisp.pl?gene=CPSF6
**BTN3A1**	Butyrophilin Subfamily 3 Member A1	Protein Coding	39	GC06P026402	0.91	https://www.genecards.org/cgi-bin/carddisp.pl?gene=BTN3A1
**RFX3**	Regulatory Factor X3	Protein Coding	39	GC09M003214	0.91	https://www.genecards.org/cgi-bin/carddisp.pl?gene=RFX3

**RPGRIP1L**	RPGRIP1 Like	Protein Coding	39	GC16M053597	0.91	https://www.genecards.org/cgi-bin/carddisp.pl?gene=RPGRIP1L
**DDX10**	DEAD-Box Helicase 10	Protein Coding	39	GC11P108569	0.91	https://www.genecards.org/cgi-bin/carddisp.pl?gene=DDX10
**NCAM2**	Neural Cell Adhesion Molecule 2	Protein Coding	39	GC21P020998	0.91	https://www.genecards.org/cgi-bin/carddisp.pl?gene=NCAM2
**PLXNC1**	Plexin C1	Protein Coding	39	GC12P094150	0.91	https://www.genecards.org/cgi-bin/carddisp.pl?gene=PLXNC1
**TCTN2**	Tectonic Family Member 2	Protein Coding	39	GC12P123671	0.91	https://www.genecards.org/cgi-bin/carddisp.pl?gene=TCTN2
**RAD54L2**	RAD54 Like 2	Protein Coding	39	GC03P051538	0.91	https://www.genecards.org/cgi-bin/carddisp.pl?gene=RAD54L2
**TIPIN**	TIMELESS Interacting Protein	Protein Coding	39	GC15M066336	0.91	https://www.genecards.org/cgi-bin/carddisp.pl?gene=TIPIN
**NCOA6**	Nuclear Receptor Coactivator 6	Protein Coding	39	GC20M034696	0.91	https://www.genecards.org/cgi-bin/carddisp.pl?gene=NCOA6

**RNASEH2C**	Ribonuclease H2 Subunit C	Protein Coding	39	GC11M065714	0.91	https://www.genecards.org/cgi-bin/carddisp.pl?gene=RNASEH2C
**KCNIP4**	Potassium Voltage-Gated Channel Interacting Protein 4	Protein Coding	39	GC04M020728	0.91	https://www.genecards.org/cgi-bin/carddisp.pl?gene=KCNIP4
**EYA2**	EYA Transcriptional Coactivator And Phosphatase 2	Protein Coding	38	GC20P046894	0.91	https://www.genecards.org/cgi-bin/carddisp.pl?gene=EYA2
**ACER3**	Alkaline Ceramidase 3	Protein Coding	38	GC11P076860	0.91	https://www.genecards.org/cgi-bin/carddisp.pl?gene=ACER3
**CDK2AP1**	Cyclin Dependent Kinase 2 Associated Protein 1	Protein Coding	38	GC12M123260	0.91	https://www.genecards.org/cgi-bin/carddisp.pl?gene=CDK2AP1
**RFT1**	RFT1 Homolog	Protein Coding	38	GC03M053071	0.91	https://www.genecards.org/cgi-bin/carddisp.pl?gene=RFT1
**RPL29**	Ribosomal Protein L29	Protein Coding	38	GC03M052005	0.91	https://www.genecards.org/cgi-bin/carddisp.pl?gene=RPL29
**SF1**	Splicing Factor 1	Protein Coding	38	GC11M064764	0.91	https://www.genecards.org/cgi-bin/carddisp.pl?gene=SF1

**TNIP2**	TNFAIP3 Interacting Protein 2	Protein Coding	38	GC04M002741	0.91	https://www.genecards.org/cgi-bin/carddisp.pl?gene=TNIP2
**SF3B2**	Splicing Factor 3b Subunit 2	Protein Coding	38	GC11P066050	0.91	https://www.genecards.org/cgi-bin/carddisp.pl?gene=SF3B2
**ZMYND8**	Zinc Finger MYND-Type Containing 8	Protein Coding	38	GC20M047209	0.91	https://www.genecards.org/cgi-bin/carddisp.pl?gene=ZMYND8
**FRMD4A**	FERM Domain Containing 4A	Protein Coding	37	GC10M013643	0.91	https://www.genecards.org/cgi-bin/carddisp.pl?gene=FRMD4A
**LRIG3**	Leucine Rich Repeats And Immunoglobulin Like Domains 3	Protein Coding	37	GC12M058872	0.91	https://www.genecards.org/cgi-bin/carddisp.pl?gene=LRIG3
**EXOSC4**	Exosome Component 4	Protein Coding	37	GC08P144079	0.91	https://www.genecards.org/cgi-bin/carddisp.pl?gene=EXOSC4
**FAM136A**	Family With Sequence Similarity 136 Member A	Protein Coding	37	GC02M070296	0.91	https://www.genecards.org/cgi-bin/carddisp.pl?gene=FAM136A
**LYPLAL1**	Lysophospholipase Like 1	Protein Coding	37	GC01P219173	0.91	https://www.genecards.org/cgi-bin/carddisp.pl?gene=LYPLAL1

**ANKRD6**	Ankyrin Repeat Domain 6	Protein Coding	37	GC06P089433	0.91	https://www.genecards.org/cgi-bin/carddisp.pl?gene=ANKRD6
**BTN2A1**	Butyrophilin Subfamily 2 Member A1	Protein Coding	37	GC06P026457	0.91	https://www.genecards.org/cgi-bin/carddisp.pl?gene=BTN2A1
**FOXL1**	Forkhead Box L1	Protein Coding	37	GC16P086576	0.91	https://www.genecards.org/cgi-bin/carddisp.pl?gene=FOXL1
**BTN2A2**	Butyrophilin Subfamily 2 Member A2	Protein Coding	37	GC06P026382	0.91	https://www.genecards.org/cgi-bin/carddisp.pl?gene=BTN2A2
**CHD9**	Chromodomain Helicase DNA Binding Protein 9	Protein Coding	37	GC16P053041	0.91	https://www.genecards.org/cgi-bin/carddisp.pl?gene=CHD9
**DLGAP2**	DLG Associated Protein 2	Protein Coding	37	GC08P000739	0.91	https://www.genecards.org/cgi-bin/carddisp.pl?gene=DLGAP2
**HNRNPUL1**	Heterogeneous Nuclear Ribonucleoprotein U Like 1	Protein Coding	37	GC19P041262	0.91	https://www.genecards.org/cgi-bin/carddisp.pl?gene=HNRNPUL1
**PARP10**	Poly(ADP-Ribose) Polymerase Family Member 10	Protein Coding	37	GC08M143977	0.91	https://www.genecards.org/cgi-bin/carddisp.pl?gene=PARP10

**SENP6**	SUMO Specific Peptidase 6	Protein Coding	37	GC06P075601	0.91	https://www.genecards.org/cgi-bin/carddisp.pl?gene=SENP6
**IRX3**	Iroquois Homeobox 3	Protein Coding	37	GC16M054283	0.91	https://www.genecards.org/cgi-bin/carddisp.pl?gene=IRX3
**TNFSF8**	TNF Superfamily Member 8	Protein Coding	37	GC09M114893	0.91	https://www.genecards.org/cgi-bin/carddisp.pl?gene=TNFSF8
**TAS1R2**	Taste 1 Receptor Member 2	Protein Coding	37	GC01M018839	0.91	https://www.genecards.org/cgi-bin/carddisp.pl?gene=TAS1R2
**RBM39**	RNA Binding Motif Protein 39	Protein Coding	37	GC20M035703	0.91	https://www.genecards.org/cgi-bin/carddisp.pl?gene=RBM39
**RBM12**	RNA Binding Motif Protein 12	Protein Coding	37	GC20M035648	0.91	https://www.genecards.org/cgi-bin/carddisp.pl?gene=RBM12
**SNX32**	Sorting Nexin 32	Protein Coding	37	GC11P065833	0.91	https://www.genecards.org/cgi-bin/carddisp.pl?gene=SNX32
**NDST4**	N-Deacetylase And N-Sulfotransferase 4	Protein Coding	37	GC04M114827	0.91	https://www.genecards.org/cgi-bin/carddisp.pl?gene=NDST4

**MRPL11**	Mitochondrial Ribosomal Protein L11	Protein Coding	37	GC11M066436	0.91	https://www.genecards.org/cgi-bin/carddisp.pl?gene=MRPL11
**LRRN1**	Leucine Rich Repeat Neuronal 1	Protein Coding	36	GC03P003799	0.91	https://www.genecards.org/cgi-bin/carddisp.pl?gene=LRRN1
**ATF7IP**	Activating Transcription Factor 7 Interacting Protein	Protein Coding	36	GC12P014365	0.91	https://www.genecards.org/cgi-bin/carddisp.pl?gene=ATF7IP
**ERGIC3**	ERGIC And Golgi 3	Protein Coding	36	GC20P035542	0.91	https://www.genecards.org/cgi-bin/carddisp.pl?gene=ERGIC3
**ERP27**	Endoplasmic Reticulum Protein 27	Protein Coding	36	GC12M014914	0.91	https://www.genecards.org/cgi-bin/carddisp.pl?gene=ERP27
**ABHD14B**	Abhydrolase Domain Containing 14B	Protein Coding	36	GC03M051968	0.91	https://www.genecards.org/cgi-bin/carddisp.pl?gene=ABHD14B
**BTN3A2**	Butyrophilin Subfamily 3 Member A2	Protein Coding	36	GC06P026365	0.91	https://www.genecards.org/cgi-bin/carddisp.pl?gene=BTN3A2
**COLGALT2**	Collagen Beta(1-O)Galactosyltransferase 2	Protein Coding	36	GC01M183899	0.91	https://www.genecards.org/cgi-bin/carddisp.pl?gene=COLGALT2

**NELFA**	Negative Elongation Factor Complex Member A	Protein Coding	36	GC04M001985	0.91	https://www.genecards.org/cgi-bin/carddisp.pl?gene=NELFA
**SF3A2**	Splicing Factor 3a Subunit 2	Protein Coding	36	GC19P002236	0.91	https://www.genecards.org/cgi-bin/carddisp.pl?gene=SF3A2
**PPM1H**	Protein Phosphatase, Mg2+/Mn2+ Dependent 1H	Protein Coding	36	GC12M062643	0.91	https://www.genecards.org/cgi-bin/carddisp.pl?gene=PPM1H
**SBNO1**	Strawberry Notch Homolog 1	Protein Coding	36	GC12M123289	0.91	https://www.genecards.org/cgi-bin/carddisp.pl?gene=SBNO1
**RPRD2**	Regulation Of Nuclear Pre-MRNA Domain Containing 2	Protein Coding	36	GC01P150363	0.91	https://www.genecards.org/cgi-bin/carddisp.pl?gene=RPRD2
**POGK**	Pogo Transposable Element Derived With KRAB Domain	Protein Coding	36	GC01P166809	0.91	https://www.genecards.org/cgi-bin/carddisp.pl?gene=POGK
**ISLR**	Immunoglobulin Superfamily Containing Leucine Rich Repeat	Protein Coding	36	GC15P074173	0.91	https://www.genecards.org/cgi-bin/carddisp.pl?gene=ISLR
**RAPH1**	Ras Association (RalGDS/AF-6) And Pleckstrin Homology Domains 1	Protein Coding	36	GC02M203394	0.91	https://www.genecards.org/cgi-bin/carddisp.pl?gene=RAPH1

**KIAA1217**	KIAA1217	Protein Coding	36	GC10P023695	0.91	https://www.genecards.org/cgi-bin/carddisp.pl?gene=KIAA1217
**KNTC1**	Kinetochore Associated 1	Protein Coding	36	GC12P122527	0.91	https://www.genecards.org/cgi-bin/carddisp.pl?gene=KNTC1
**KIF26B**	Kinesin Family Member 26B	Protein Coding	36	GC01P245154	0.91	https://www.genecards.org/cgi-bin/carddisp.pl?gene=KIF26B
**RBM6**	RNA Binding Motif Protein 6	Protein Coding	36	GC03P049940	0.91	https://www.genecards.org/cgi-bin/carddisp.pl?gene=RBM6
**TMEM91**	Transmembrane Protein 91	Protein Coding	36	GC19P041351	0.91	https://www.genecards.org/cgi-bin/carddisp.pl?gene=TMEM91
**LMAN1L**	Lectin, Mannose Binding 1 Like	Protein Coding	36	GC15P074812	0.91	https://www.genecards.org/cgi-bin/carddisp.pl?gene=LMAN1L
**FSTL5**	Follistatin Like 5	Protein Coding	35	GC04M161383	0.91	https://www.genecards.org/cgi-bin/carddisp.pl?gene=FSTL5
**CCDC91**	Coiled-Coil Domain Containing 91	Protein Coding	35	GC12P028133	0.91	https://www.genecards.org/cgi-bin/carddisp.pl?gene=CCDC91

**APOBEC4**	Apolipoprotein B MRNA Editing Enzyme Catalytic Polypeptide Like 4	Protein Coding	35	GC01M183646	0.91	https://www.genecards.org/cgi-bin/carddisp.pl?gene=APOBEC4
**ANAPC4**	Anaphase Promoting Complex Subunit 4	Protein Coding	35	GC04P025379	0.91	https://www.genecards.org/cgi-bin/carddisp.pl?gene=ANAPC4
**BBX**	BBX High Mobility Group Box Domain Containing	Protein Coding	35	GC03P107522	0.91	https://www.genecards.org/cgi-bin/carddisp.pl?gene=BBX
**AAR2**	AAR2 Splicing Factor	Protein Coding	35	GC20P036236	0.91	https://www.genecards.org/cgi-bin/carddisp.pl?gene=AAR2
**BTN3A3**	Butyrophilin Subfamily 3 Member A3	Protein Coding	35	GC06P028714	0.91	https://www.genecards.org/cgi-bin/carddisp.pl?gene=BTN3A3
**MON2**	MON2 Homolog, Regulator Of Endosome-To-Golgi Trafficking	Protein Coding	35	GC12P062466	0.91	https://www.genecards.org/cgi-bin/carddisp.pl?gene=MON2
**DDX55**	DEAD-Box Helicase 55	Protein Coding	35	GC12P123602	0.91	https://www.genecards.org/cgi-bin/carddisp.pl?gene=DDX55
**MOB3B**	MOB Kinase Activator 3B	Protein Coding	35	GC09M027332	0.91	https://www.genecards.org/cgi-bin/carddisp.pl?gene=MOB3B

**RWDD2B**	RWD Domain Containing 2B	Protein Coding	35	GC21M029004	0.91	https://www.genecards.org/cgi-bin/carddisp.pl?gene=RWDD2B
**RRP9**	Ribosomal RNA Processing 9, U3 Small Nucleolar RNA Binding Protein	Protein Coding	35	GC03M051943	0.91	https://www.genecards.org/cgi-bin/carddisp.pl?gene=RRP9
**NT5DC2**	5'-Nucleotidase Domain Containing 2	Protein Coding	35	GC03M052524	0.91	https://www.genecards.org/cgi-bin/carddisp.pl?gene=NT5DC2
**ISLR2**	Immunoglobulin Superfamily Containing Leucine Rich Repeat 2	Protein Coding	35	GC15P074100	0.91	https://www.genecards.org/cgi-bin/carddisp.pl?gene=ISLR2
**TTLL12**	Tubulin Tyrosine Ligase Like 12	Protein Coding	35	GC22M045425	0.91	https://www.genecards.org/cgi-bin/carddisp.pl?gene=TTLL12
**TSKU**	Tsukushi, Small Leucine Rich Proteoglycan	Protein Coding	35	GC11P076782	0.91	https://www.genecards.org/cgi-bin/carddisp.pl?gene=TSKU
**KIF12**	Kinesin Family Member 12	Protein Coding	35	GC09M114086	0.91	https://www.genecards.org/cgi-bin/carddisp.pl?gene=KIF12
**ZFYVE28**	Zinc Finger FYVE-Type Containing 28	Protein Coding	35	GC04M002299	0.91	https://www.genecards.org/cgi-bin/carddisp.pl?gene=ZFYVE28

**ZCCHC8**	Zinc Finger CCHC-Type Containing 8	Protein Coding	35	GC12M122472	0.91	https://www.genecards.org/cgi-bin/carddisp.pl?gene=ZCCHC8
**ZWILCH**	Zwilch Kinetochore Protein	Protein Coding	35	GC15P066504	0.91	https://www.genecards.org/cgi-bin/carddisp.pl?gene=ZWILCH
**UVSSA**	UV Stimulated Scaffold Protein A	Protein Coding	35	GC04P001341	0.91	https://www.genecards.org/cgi-bin/carddisp.pl?gene=UVSSA
**VPS51**	VPS51 Subunit Of GARP Complex	Protein Coding	35	GC11P065089	0.91	https://www.genecards.org/cgi-bin/carddisp.pl?gene=VPS51
**FILIP1**	Filamin A Interacting Protein 1	Protein Coding	34	GC06M075291	0.91	https://www.genecards.org/cgi-bin/carddisp.pl?gene=FILIP1
**EPPK1**	Epiplakin 1	Protein Coding	34	GC08M143857	0.91	https://www.genecards.org/cgi-bin/carddisp.pl?gene=EPPK1
**C11orf80**	Chromosome 11 Open Reading Frame 80	Protein Coding	34	GC11P066744	0.91	https://www.genecards.org/cgi-bin/carddisp.pl?gene=C11orf80
**HECW1**	HECT, C2 And WW Domain Containing E3 Ubiquitin Protein Ligase 1	Protein Coding	34	GC07P043112	0.91	https://www.genecards.org/cgi-bin/carddisp.pl?gene=HECW1

**CCDC33**	Coiled-Coil Domain Containing 33	Protein Coding	34	GC15P074217	0.91	https://www.genecards.org/cgi-bin/carddisp.pl?gene=CCDC33
**MPHOSPH9**	M-Phase Phosphoprotein 9	Protein Coding	34	GC12M123153	0.91	https://www.genecards.org/cgi-bin/carddisp.pl?gene=MPHOSPH9
**NRBP2**	Nuclear Receptor Binding Protein 2	Protein Coding	34	GC08M143832	0.91	https://www.genecards.org/cgi-bin/carddisp.pl?gene=NRBP2
**SRBD1**	S1 RNA Binding Domain 1	Protein Coding	34	GC02M045388	0.91	https://www.genecards.org/cgi-bin/carddisp.pl?gene=SRBD1
**TENM2**	Teneurin Transmembrane Protein 2	Protein Coding	34	GC05P166979	0.91	https://www.genecards.org/cgi-bin/carddisp.pl?gene=TENM2
**THSD7B**	Thrombospondin Type 1 Domain Containing 7B	Protein Coding	34	GC02P136765	0.91	https://www.genecards.org/cgi-bin/carddisp.pl?gene=THSD7B
**GALNT18**	Polypeptide N-Acetylgalactosaminyltransferase 18	Protein Coding	33	GC11M011293	0.91	https://www.genecards.org/cgi-bin/carddisp.pl?gene=GALNT18
**CCDC62**	Coiled-Coil Domain Containing 62	Protein Coding	33	GC12P122863	0.91	https://www.genecards.org/cgi-bin/carddisp.pl?gene=CCDC62

**H1-4**	H1.4 Linker Histone, Cluster Member	Protein Coding	33	GC06P028871	0.91	https://www.genecards.org/cgi-bin/carddisp.pl?gene=H1-4
**HMGN4**	High Mobility Group Nucleosomal Binding Domain 4	Protein Coding	33	GC06P026538	0.91	https://www.genecards.org/cgi-bin/carddisp.pl?gene=HMGN4
**MLXIP**	MLX Interacting Protein	Protein Coding	33	GC12P122078	0.91	https://www.genecards.org/cgi-bin/carddisp.pl?gene=MLXIP
**HAUS3**	HAUS Augmin Like Complex Subunit 3	Protein Coding	33	GC04M002079	0.91	https://www.genecards.org/cgi-bin/carddisp.pl?gene=HAUS3
**EIF1AD**	Eukaryotic Translation Initiation Factor 1A Domain Containing	Protein Coding	33	GC11M065996	0.91	https://www.genecards.org/cgi-bin/carddisp.pl?gene=EIF1AD
**SPATC1**	Spermatogenesis And Centriole Associated 1	Protein Coding	33	GC08P144012	0.91	https://www.genecards.org/cgi-bin/carddisp.pl?gene=SPATC1
**IQCH**	IQ Motif Containing H	Protein Coding	33	GC15P067254	0.91	https://www.genecards.org/cgi-bin/carddisp.pl?gene=IQCH
**IZUMO4**	IZUMO Family Member 4	Protein Coding	33	GC19P002096	0.91	https://www.genecards.org/cgi-bin/carddisp.pl?gene=IZUMO4

**KAZN**	Kazrin, Periplakin Interacting Protein	Protein Coding	33	GC01P013893	0.91	https://www.genecards.org/cgi-bin/carddisp.pl?gene=KAZN
**ZFPL1**	Zinc Finger Protein Like 1	Protein Coding	33	GC11P065084	0.91	https://www.genecards.org/cgi-bin/carddisp.pl?gene=ZFPL1
**ZC3H3**	Zinc Finger CCCH-Type Containing 3	Protein Coding	33	GC08M143437	0.91	https://www.genecards.org/cgi-bin/carddisp.pl?gene=ZC3H3
**FAM53A**	Family With Sequence Similarity 53 Member A	Protein Coding	32	GC04M001610	0.91	https://www.genecards.org/cgi-bin/carddisp.pl?gene=FAM53A
**LURAP1L**	Leucine Rich Adaptor Protein 1 Like	Protein Coding	32	GC09P012775	0.91	https://www.genecards.org/cgi-bin/carddisp.pl?gene=LURAP1L
**FAM83C**	Family With Sequence Similarity 83 Member C	Protein Coding	32	GC20M035285	0.91	https://www.genecards.org/cgi-bin/carddisp.pl?gene=FAM83C
**BRINP1**	BMP/Retinoic Acid Inducible Neural Specific 1	Protein Coding	32	GC09M119153	0.91	https://www.genecards.org/cgi-bin/carddisp.pl?gene=BRINP1
**BTNL8**	Butyrophilin Like 8	Protein Coding	32	GC05P181617	0.91	https://www.genecards.org/cgi-bin/carddisp.pl?gene=BTNL8

**GSDMC**	Gasdermin C	Protein Coding	32	GC08M129705	0.91	https://www.genecards.org/cgi-bin/carddisp.pl?gene=GSDMC
**CEACAM21**	CEA Cell Adhesion Molecule 21	Protein Coding	32	GC19P041550	0.91	https://www.genecards.org/cgi-bin/carddisp.pl?gene=CEACAM21
**H1-2**	H1.2 Linker Histone, Cluster Member	Protein Coding	32	GC06M026056	0.91	https://www.genecards.org/cgi-bin/carddisp.pl?gene=H1-2
**CCDC97**	Coiled-Coil Domain Containing 97	Protein Coding	32	GC19P041310	0.91	https://www.genecards.org/cgi-bin/carddisp.pl?gene=CCDC97
**RSRC2**	Arginine And Serine Rich Coiled-Coil 2	Protein Coding	32	GC12M122503	0.91	https://www.genecards.org/cgi-bin/carddisp.pl?gene=RSRC2
**NKAIN1**	Sodium/Potassium Transporting ATPase Interacting 1	Protein Coding	32	GC01M031179	0.91	https://www.genecards.org/cgi-bin/carddisp.pl?gene=NKAIN1
**POLR2M**	RNA Polymerase II Subunit M	Protein Coding	32	GC15P057706	0.91	https://www.genecards.org/cgi-bin/carddisp.pl?gene=POLR2M
**KIAA1755**	KIAA1755	Protein Coding	32	GC20M038210	0.91	https://www.genecards.org/cgi-bin/carddisp.pl?gene=KIAA1755

**KLHL42**	Kelch Like Family Member 42	Protein Coding	32	GC12P027780	0.91	https://www.genecards.org/cgi-bin/carddisp.pl?gene=KLHL42
**ZBED5**	Zinc Finger BED-Type Containing 5	Protein Coding	32	GC11M010861	0.91	https://www.genecards.org/cgi-bin/carddisp.pl?gene=ZBED5
**FAM193A**	Family With Sequence Similarity 193 Member A	Protein Coding	31	GC04P002536	0.91	https://www.genecards.org/cgi-bin/carddisp.pl?gene=FAM193A
**FAM98A**	Family With Sequence Similarity 98 Member A	Protein Coding	31	GC02M033583	0.91	https://www.genecards.org/cgi-bin/carddisp.pl?gene=FAM98A
**H3C3**	H3 Clustered Histone 3	Protein Coding	31	GC06P028673	0.91	https://www.genecards.org/cgi-bin/carddisp.pl?gene=H3C3
**CCDC54**	Coiled-Coil Domain Containing 54	Protein Coding	31	GC03P107377	0.91	https://www.genecards.org/cgi-bin/carddisp.pl?gene=CCDC54
**TMEM241**	Transmembrane Protein 241	Protein Coding	31	GC18M023197	0.91	https://www.genecards.org/cgi-bin/carddisp.pl?gene=TMEM241
**LSMEM1**	Leucine Rich Single-Pass Membrane Protein 1	Protein Coding	30	GC07P112480	0.91	https://www.genecards.org/cgi-bin/carddisp.pl?gene=LSMEM1

**C12orf60**	Chromosome 12 Open Reading Frame 60	Protein Coding	30	GC12P014568	0.91	https://www.genecards.org/cgi-bin/carddisp.pl?gene=C12orf60
**EQTN**	Equatorin	Protein Coding	30	GC09M027284	0.91	https://www.genecards.org/cgi-bin/carddisp.pl?gene=EQTN
**H1-3**	H1.3 Linker Histone, Cluster Member	Protein Coding	30	GC06M027296	0.91	https://www.genecards.org/cgi-bin/carddisp.pl?gene=H1-3
**TMEM129**	Transmembrane Protein 129, E3 Ubiquitin Ligase	Protein Coding	30	GC04M001715	0.91	https://www.genecards.org/cgi-bin/carddisp.pl?gene=TMEM129
**LRRC43**	Leucine Rich Repeat Containing 43	Protein Coding	29	GC12P122167	0.91	https://www.genecards.org/cgi-bin/carddisp.pl?gene=LRRC43
**CNBD2**	Cyclic Nucleotide Binding Domain Containing 2	Protein Coding	29	GC20P035954	0.91	https://www.genecards.org/cgi-bin/carddisp.pl?gene=CNBD2
**H2BC4**	H2B Clustered Histone 4	Protein Coding	29	GC06M027315	0.91	https://www.genecards.org/cgi-bin/carddisp.pl?gene=H2BC4
**H2BC9**	H2B Clustered Histone 9	Protein Coding	29	GC06P028898	0.91	https://www.genecards.org/cgi-bin/carddisp.pl?gene=H2BC9

**C11orf87**	Chromosome 11 Open Reading Frame 87	Protein Coding	28	GC11P109421	0.91	https://www.genecards.org/cgi-bin/carddisp.pl?gene=C11orf87
**H2AC6**	H2A Clustered Histone 6	Protein Coding	28	GC06P028875	0.91	https://www.genecards.org/cgi-bin/carddisp.pl?gene=H2AC6
**TMEM61**	Transmembrane Protein 61	Protein Coding	28	GC01P054980	0.91	https://www.genecards.org/cgi-bin/carddisp.pl?gene=TMEM61
**ZNF345**	Zinc Finger Protein 345	Protein Coding	28	GC19P036850	0.91	https://www.genecards.org/cgi-bin/carddisp.pl?gene=ZNF345
**ZNRD2**	Zinc Ribbon Domain Containing 2	Protein Coding	28	GC11P065813	0.91	https://www.genecards.org/cgi-bin/carddisp.pl?gene=ZNRD2
**C8orf82**	Chromosome 8 Open Reading Frame 82	Protein Coding	27	GC08M144525	0.91	https://www.genecards.org/cgi-bin/carddisp.pl?gene=C8orf82
**ATP23**	ATP23 Metallopeptidase And ATP Synthase Assembly Factor Homolog	Protein Coding	27	GC12P057942	0.91	https://www.genecards.org/cgi-bin/carddisp.pl?gene=ATP23
**CSNK2A3**	Casein Kinase 2 Alpha 3	Protein Coding	27	GC11M011351	0.91	https://www.genecards.org/cgi-bin/carddisp.pl?gene=CSNK2A3

**PLPP6**	Phospholipid Phosphatase 6	Protein Coding	27	GC09P004663	0.91	https://www.genecards.org/cgi-bin/carddisp.pl?gene=PLPP6
**SMIM4**	Small Integral Membrane Protein 4	Protein Coding	27	GC03P052534	0.91	https://www.genecards.org/cgi-bin/carddisp.pl?gene=SMIM4
**CCDC26**	CCDC26 Long Non-Coding RNA	RNA Gene	26	GC08M128636	0.91	https://www.genecards.org/cgi-bin/carddisp.pl?gene=CCDC26
**C16orf95**	Chromosome 16 Open Reading Frame 95	Protein Coding	26	GC16M087119	0.91	https://www.genecards.org/cgi-bin/carddisp.pl?gene=C16orf95
**MS4A13**	Membrane Spanning 4-Domains A13	Protein Coding	26	GC11P060515	0.91	https://www.genecards.org/cgi-bin/carddisp.pl?gene=MS4A13
**PCNX3**	Pecanex 3	Protein Coding	25	GC11P065830	0.91	https://www.genecards.org/cgi-bin/carddisp.pl?gene=PCNX3
**MANSC4**	MANSC Domain Containing 4	Protein Coding	24	GC12M027762	0.91	https://www.genecards.org/cgi-bin/carddisp.pl?gene=MANSC4
**DELEC1**	Deleted In Esophageal Cancer 1	RNA Gene	24	GC09P114945	0.91	https://www.genecards.org/cgi-bin/carddisp.pl?gene=DELEC1

**RFLNA**	Refilin A	Protein Coding	24	GC12P123979	0.91	https://www.genecards.org/cgi-bin/carddisp.pl?gene=RFLNA
**STIMATE**	STIM Activating Enhancer	Protein Coding	24	GC03M052837	0.91	https://www.genecards.org/cgi-bin/carddisp.pl?gene=STIMATE
**MINAR1**	Membrane Integral NOTCH2 Associated Receptor 1	Protein Coding	23	GC15P079414	0.91	https://www.genecards.org/cgi-bin/carddisp.pl?gene=MINAR1
**BTN2A3P**	Butyrophilin Subfamily 2 Member A3, Pseudogene	Pseudogene	23	GC06P028713	0.91	https://www.genecards.org/cgi-bin/carddisp.pl?gene=BTN2A3P
**LINC02875**	Long Intergenic Non-Protein Coding RNA 2875	RNA Gene	23	GC17P061412	0.91	https://www.genecards.org/cgi-bin/carddisp.pl?gene=LINC02875
**SMIM23**	Small Integral Membrane Protein 23	Protein Coding	22	GC05P171785	0.91	https://www.genecards.org/cgi-bin/carddisp.pl?gene=SMIM23
**LINC01006**	Long Intergenic Non-Protein Coding RNA 1006	RNA Gene	21	GC07M156388	0.91	https://www.genecards.org/cgi-bin/carddisp.pl?gene=LINC01006
**DNAH10OS**	Dynein Axonemal Heavy Chain 10 Opposite Strand	Protein Coding	19	GC12M123927	0.91	https://www.genecards.org/cgi-bin/carddisp.pl?gene=DNAH10OS

**LINC00114**	Long Intergenic Non-Protein Coding RNA 114	RNA Gene	19	GC21M038738	0.91	https://www.genecards.org/cgi-bin/carddisp.pl?gene=LINC00114
**NOP14-AS1**	NOP14 Antisense RNA 1	RNA Gene	18	GC04P002936	0.91	https://www.genecards.org/cgi-bin/carddisp.pl?gene=NOP14-AS1
**GDF5-AS1**	GDF5 Antisense RNA 1	RNA Gene	17	GC20P035435	0.91	https://www.genecards.org/cgi-bin/carddisp.pl?gene=GDF5-AS1
**CTBP1-DT**	CTBP1 Divergent Transcript	RNA Gene	17	GC04P001250	0.91	https://www.genecards.org/cgi-bin/carddisp.pl?gene=CTBP1-DT
**MIR661**	MicroRNA 661	RNA Gene	17	GC08M143945	0.91	https://www.genecards.org/cgi-bin/carddisp.pl?gene=MIR661
**HCG11**	HLA Complex Group 11	RNA Gene	17	GC06P028718	0.91	https://www.genecards.org/cgi-bin/carddisp.pl?gene=HCG11
**GUSBP2**	GUSB Pseudogene 2	Pseudogene	16	GC06M026871	0.91	https://www.genecards.org/cgi-bin/carddisp.pl?gene=GUSBP2
**LINC01088**	Long Intergenic Non-Protein Coding RNA 1088	RNA Gene	16	GC04P078971	0.91	https://www.genecards.org/cgi-bin/carddisp.pl?gene=LINC01088

**FAM83C-AS1**	FAM83C Antisense RNA 1	RNA Gene	14	GC20P035285	0.91	https://www.genecards.org/cgi-bin/carddisp.pl?gene=FAM83C-AS1
**HCG23**	HLA Complex Group 23	RNA Gene	14	GC06P047338	0.91	https://www.genecards.org/cgi-bin/carddisp.pl?gene=HCG23
**NR2F2-AS1**	NR2F2 Antisense RNA 1	RNA Gene	14	GC15M103900	0.91	https://www.genecards.org/cgi-bin/carddisp.pl?gene=NR2F2-AS1
**SNORD19B**	Small Nucleolar RNA, C/D Box 19B	RNA Gene	14	GC03P052820	0.91	https://www.genecards.org/cgi-bin/carddisp.pl?gene=SNORD19B
**SNORD16**	Small Nucleolar RNA, C/D Box 16	RNA Gene	14	GC15M066574	0.91	https://www.genecards.org/cgi-bin/carddisp.pl?gene=SNORD16
**IQCH-AS1**	IQCH Antisense RNA 1	RNA Gene	14	GC15M067290	0.91	https://www.genecards.org/cgi-bin/carddisp.pl?gene=IQCH-AS1
**SAMMSON**	Survival Associated Mitochondrial Melanoma Specific Oncogenic Non-Coding RNA	RNA Gene	14	GC03P069999	0.91	https://www.genecards.org/cgi-bin/carddisp.pl?gene=SAMMSON
**ZC3H11B**	Zinc Finger CCCH-Type Containing 11B	Protein Coding	14	GC01M219607	0.91	https://www.genecards.org/cgi-bin/carddisp.pl?gene=ZC3H11B

**LINC00977**	Long Intergenic Non-Protein Coding RNA 977	RNA Gene	14	GC08M128635	0.91	https://www.genecards.org/cgi-bin/carddisp.pl?gene=LINC00977
**MMP24OS**	MMP24 Opposite Strand	Protein Coding	13	GC20M035202	0.91	https://www.genecards.org/cgi-bin/carddisp.pl?gene=MMP24OS
**ILF3-DT**	ILF3 Divergent Transcript	RNA Gene	13	GC19M010652	0.91	https://www.genecards.org/cgi-bin/carddisp.pl?gene=ILF3-DT
**LINC01094**	Long Intergenic Non-Protein Coding RNA 1094	RNA Gene	13	GC04P078645	0.91	https://www.genecards.org/cgi-bin/carddisp.pl?gene=LINC01094
**LINC01507**	Long Intergenic Non-Protein Coding RNA 1507	RNA Gene	13	GC09P079824	0.91	https://www.genecards.org/cgi-bin/carddisp.pl?gene=LINC01507
**MIR1289-1**	MicroRNA 1289-1	RNA Gene	12	GC20M035453	0.91	https://www.genecards.org/cgi-bin/carddisp.pl?gene=MIR1289-1
**GARS1-DT**	GARS1 Divergent Transcript	RNA Gene	12	GC07M030385	0.91	https://www.genecards.org/cgi-bin/carddisp.pl?gene=GARS1-DT
**LYPLAL1-AS1**	LYPLAL1 Antisense RNA 1	RNA Gene	12	GC01M219411	0.91	https://www.genecards.org/cgi-bin/carddisp.pl?gene=LYPLAL1-AS1

**ATP2B1-AS1**	ATP2B1 Antisense RNA 1	RNA Gene	12	GC12P089709	0.91	https://www.genecards.org/cgi-bin/carddisp.pl?gene=ATP2B1-AS1
**ASTN2-AS1**	ASTN2 Antisense RNA 1	RNA Gene	12	GC09P116515	0.91	https://www.genecards.org/cgi-bin/carddisp.pl?gene=ASTN2-AS1
**MDFIC2**	MyoD Family Inhibitor Domain Containing 2	Protein Coding	12	GC03M070197	0.91	https://www.genecards.org/cgi-bin/carddisp.pl?gene=MDFIC2
**DUBR**	DPPA2 Upstream Binding RNA	RNA Gene	12	GC03P107242	0.91	https://www.genecards.org/cgi-bin/carddisp.pl?gene=DUBR
**LINC01574**	Long Intergenic Non-Protein Coding RNA 1574	RNA Gene	11	GC05P176743	0.91	https://www.genecards.org/cgi-bin/carddisp.pl?gene=LINC01574
**LOC101928731**	Uncharacterized LOC101928731	RNA Gene	10	GC12M093707	0.91	https://www.genecards.org/cgi-bin/carddisp.pl?gene=LOC101928731
**ENSG00000255224**		RNA Gene	10	GC08M144078	0.91	https://www.genecards.org/cgi-bin/carddisp.pl?gene=ENSG00000255224
**ROCR**	Regulator Of Chondrogenesis RNA	RNA Gene	10	GC17M072023	0.91	https://www.genecards.org/cgi-bin/carddisp.pl?gene=ROCR

**ZNRD2-AS1**	ZNRD2 Antisense RNA 1 (Head To Head)	RNA Gene	10	GC11M065571	0.91	https://www.genecards.org/cgi-bin/carddisp.pl?gene=ZNRD2-AS1
**LSP1P3**	LSP1 Pseudogene 3	Pseudogene	9	GC05P028986	0.91	https://www.genecards.org/cgi-bin/carddisp.pl?gene=LSP1P3
**LOC101928540**	Uncharacterized LOC101928540	RNA Gene	9	GC06P075383	0.91	https://www.genecards.org/cgi-bin/carddisp.pl?gene=LOC101928540
**SBNO1-AS1**	SBNO1 Antisense RNA 1	RNA Gene	9	GC12P123365	0.91	https://www.genecards.org/cgi-bin/carddisp.pl?gene=SBNO1-AS1
**KIF26B-AS1**	KIF26B Antisense RNA 1	RNA Gene	9	GC01M245208	0.91	https://www.genecards.org/cgi-bin/carddisp.pl?gene=KIF26B-AS1
**ZNF664-RFLNA**	ZNF664-RFLNA Readthrough	Protein Coding	9	GC12P123983	0.91	https://www.genecards.org/cgi-bin/carddisp.pl?gene=ZNF664-RFLNA
**LINC01754**	Long Intergenic Non-Protein Coding RNA 1754	RNA Gene	9	GC20M047384	0.91	https://www.genecards.org/cgi-bin/carddisp.pl?gene=LINC01754
**LINC01338**	Long Intergenic Non-Protein Coding RNA 1338	RNA Gene	9	GC05M082850	0.91	https://www.genecards.org/cgi-bin/carddisp.pl?gene=LINC01338

**LINC02399**	Long Intergenic Non-Protein Coding RNA 2399	RNA Gene	9	GC12P089949	0.91	https://www.genecards.org/cgi-bin/carddisp.pl?gene=LINC02399
**HOMER3-AS1**	HOMER3 Antisense RNA 1	RNA Gene	8	GC19P023518	0.91	https://www.genecards.org/cgi-bin/carddisp.pl?gene=HOMER3-AS1
**MPPED2-AS1**	MPPED2 Antisense RNA 1	RNA Gene	8	GC11P030585	0.91	https://www.genecards.org/cgi-bin/carddisp.pl?gene=MPPED2-AS1
**RPL32P1**	Ribosomal Protein L32 Pseudogene 1	Pseudogene	8	GC06P047347	0.91	https://www.genecards.org/cgi-bin/carddisp.pl?gene=RPL32P1
**ENSG00000243224**		Uncategorized	8	GC03P052241	0.91	https://www.genecards.org/cgi-bin/carddisp.pl?gene=ENSG00000243224
**ENSG00000237174**		RNA Gene	8	GC06P075357	0.91	https://www.genecards.org/cgi-bin/carddisp.pl?gene=ENSG00000237174
**RPL35AP**	Ribosomal Protein L35a Pseudogene	Pseudogene	8	GC20M047478	0.91	https://www.genecards.org/cgi-bin/carddisp.pl?gene=RPL35AP
**ENSG00000256546**		Uncategorized	8	GC12P122872	0.91	https://www.genecards.org/cgi-bin/carddisp.pl?gene=ENSG00000256546

**ENSG00000255730**	Protein Coding	8	GC19P041350	0.91	https://www.genecards.org/cgi-bin/carddisp.pl?gene=ENSG00000255730
**ENSG00000249592**	Uncategorized	8	GC04M000760	0.91	https://www.genecards.org/cgi-bin/carddisp.pl?gene=ENSG00000249592
**ENSG00000254461**	RNA Gene	8	GC11M066259	0.91	https://www.genecards.org/cgi-bin/carddisp.pl?gene=ENSG00000254461
**ENSG00000255320**	RNA Gene	8	GC11M066245	0.91	https://www.genecards.org/cgi-bin/carddisp.pl?gene=ENSG00000255320
**ENSG00000255038**	RNA Gene	8	GC11M066067	0.91	https://www.genecards.org/cgi-bin/carddisp.pl?gene=ENSG00000255038
**ENSG00000254855**	RNA Gene	8	GC11M066264	0.91	https://www.genecards.org/cgi-bin/carddisp.pl?gene=ENSG00000254855
**ENSG00000254632**	RNA Gene	8	GC11M076759	0.91	https://www.genecards.org/cgi-bin/carddisp.pl?gene=ENSG00000254632
**ENSG00000245156**	RNA Gene	8	GC11M066269	0.91	https://www.genecards.org/cgi-bin/carddisp.pl?gene=ENSG00000245156

**DUS4L-BCAP29**	DUS4L-BCAP29 Readthrough	Protein Coding	8	GC07P107564	0.91	https://www.genecards.org/cgi-bin/carddisp.pl?gene=DUS4L-BCAP29
**ENSG00000253926**		RNA Gene	8	GC08P129298	0.91	https://www.genecards.org/cgi-bin/carddisp.pl?gene=ENSG00000253926
**ENSG00000261384**		RNA Gene	8	GC15P074303	0.91	https://www.genecards.org/cgi-bin/carddisp.pl?gene=ENSG00000261384
**ENSG00000261324**		RNA Gene	8	GC12M014762	0.91	https://www.genecards.org/cgi-bin/carddisp.pl?gene=ENSG00000261324
**ENSG00000267122**		RNA Gene	8	GC19M002214	0.91	https://www.genecards.org/cgi-bin/carddisp.pl?gene=ENSG00000267122
**LINC02234**	Long Intergenic Non-Protein Coding RNA 2234	RNA Gene	8	GC05P097882	0.91	https://www.genecards.org/cgi-bin/carddisp.pl?gene=LINC02234
**RNA5SP301**	RNA, 5S Ribosomal Pseudogene 301	Pseudogene	7	GC10P013728	0.91	https://www.genecards.org/cgi-bin/carddisp.pl?gene=RNA5SP301
**MTND4P14**	MT-ND4 Pseudogene 14	Pseudogene	7	GC09P005690	0.91	https://www.genecards.org/cgi-bin/carddisp.pl?gene=MTND4P14

**NPM1P14**	Nucleophosmin 1 Pseudogene 14	Pseudogene	7	GC07P112520	0.91	https://www.genecards.org/cgi-bin/carddisp.pl?gene=NPM1P14
**ENSG00000227554**		RNA Gene	7	GC01M183723	0.91	https://www.genecards.org/cgi-bin/carddisp.pl?gene=ENSG00000227554
**ENSG00000234352**		Uncategorized	7	GC07M136687	0.91	https://www.genecards.org/cgi-bin/carddisp.pl?gene=ENSG00000234352
**ENSG00000234091**		RNA Gene	7	GC10P013771	0.91	https://www.genecards.org/cgi-bin/carddisp.pl?gene=ENSG00000234091
**ENSG00000231612**		RNA Gene	7	GC01M245675	0.91	https://www.genecards.org/cgi-bin/carddisp.pl?gene=ENSG00000231612
**ENSG00000254094**		RNA Gene	7	GC04P001356	0.91	https://www.genecards.org/cgi-bin/carddisp.pl?gene=ENSG00000254094
**ENSG00000258216**		RNA Gene	7	GC12P089712	0.91	https://www.genecards.org/cgi-bin/carddisp.pl?gene=ENSG00000258216
**ENSG00000248538**		Uncategorized	7	GC08P009175	0.91	https://www.genecards.org/cgi-bin/carddisp.pl?gene=ENSG00000248538

**ENSG00000228322**		RNA Gene	7	GC09P004299	0.91	https://www.genecards.org/cgi-bin/carddisp.pl?gene=ENSG00000228322
**ENSG00000270195**		RNA Gene	7	GC04P001714	0.91	https://www.genecards.org/cgi-bin/carddisp.pl?gene=ENSG00000270195
**ENSG00000272462**		RNA Gene	7	GC06P025992	0.91	https://www.genecards.org/cgi-bin/carddisp.pl?gene=ENSG00000272462
**ENSG00000261578**		RNA Gene	7	GC11P076800	0.91	https://www.genecards.org/cgi-bin/carddisp.pl?gene=ENSG00000261578
**LINC02742**	Long Intergenic Non-Protein Coding RNA 2742	RNA Gene	7	GC11P028703	0.91	https://www.genecards.org/cgi-bin/carddisp.pl?gene=LINC02742
**LINC02650**	Long Intergenic Non-Protein Coding RNA 2650	RNA Gene	7	GC10M083674	0.91	https://www.genecards.org/cgi-bin/carddisp.pl?gene=LINC02650
**H2AC5P**	H2A Clustered Histone 5, Pseudogene	Pseudogene	6	GC06P026043	0.91	https://www.genecards.org/cgi-bin/carddisp.pl?gene=H2AC5P
**RPL26P20**	Ribosomal Protein L26 Pseudogene 20	Pseudogene	6	GC06P075499	0.91	https://www.genecards.org/cgi-bin/carddisp.pl?gene=RPL26P20

**SOD2P1**	Superoxide Dismutase 2 Pseudogene 1	Pseudogene	6	GC01M103100	0.91	https://www.genecards.org/cgi-bin/carddisp.pl?gene=SOD2P1
**ENSG00000225884**		Uncategorized	6	GC02P191793	0.91	https://www.genecards.org/cgi-bin/carddisp.pl?gene=ENSG00000225884
**PPIAP1**	Peptidylprolyl Isomerase A Pseudogene 1	Pseudogene	6	GC21M020827	0.91	https://www.genecards.org/cgi-bin/carddisp.pl?gene=PPIAP1
**ENSG00000237530**		RNA Gene	6	GC06P047544	0.91	https://www.genecards.org/cgi-bin/carddisp.pl?gene=ENSG00000237530
**ENSG00000238224**		RNA Gene	6	GC01M245614	0.91	https://www.genecards.org/cgi-bin/carddisp.pl?gene=ENSG00000238224
**ENSG00000223727**		RNA Gene	6	GC03M003292	0.91	https://www.genecards.org/cgi-bin/carddisp.pl?gene=ENSG00000223727
**ENSG00000225050**		RNA Gene	6	GC09M118645	0.91	https://www.genecards.org/cgi-bin/carddisp.pl?gene=ENSG00000225050
**ENSG00000207009**		RNA Gene	6	GC04M001736	0.91	https://www.genecards.org/cgi-bin/carddisp.pl?gene=ENSG00000207009

**ENSG00000207189**		RNA Gene	6	GC12M123348	0.91	https://www.genecards.org/cgi-bin/carddisp.pl?gene=ENSG00000207189
**ENSG00000200998**		RNA Gene	6	GC04M078632	0.91	https://www.genecards.org/cgi-bin/carddisp.pl?gene=ENSG00000200998
**ENSG00000234892**		RNA Gene	6	GC22P043232	0.91	https://www.genecards.org/cgi-bin/carddisp.pl?gene=ENSG00000234892
**LOC105373944**	Translation Initiation Factor IF-2-Like	Protein Coding	6	GC02M236161	0.91	https://www.genecards.org/cgi-bin/carddisp.pl?gene=LOC105373944
**ENSG00000248112**		RNA Gene	6	GC05M082907	0.91	https://www.genecards.org/cgi-bin/carddisp.pl?gene=ENSG00000248112
**ENSG00000257042**		RNA Gene	6	GC12P027958	0.91	https://www.genecards.org/cgi-bin/carddisp.pl?gene=ENSG00000257042
**TPT1P9**	Tumor Protein, Translationally-Controlled 1 Pseudogene 9	Pseudogene	6	GC09M118082	0.91	https://www.genecards.org/cgi-bin/carddisp.pl?gene=TPT1P9
**ENSG00000250098**		RNA Gene	6	GC04M012949	0.91	https://www.genecards.org/cgi-bin/carddisp.pl?gene=ENSG00000250098

**ENSG00000250453**		RNA Gene	6	GC05M028488	0.91	https://www.genecards.org/cgi-bin/carddisp.pl?gene=ENSG00000250453
**ENSG00000254614**		Uncategorized	6	GC11M065180	0.91	https://www.genecards.org/cgi-bin/carddisp.pl?gene=ENSG00000254614
**ENSG00000241956**		Uncategorized	6	GC05P164297	0.91	https://www.genecards.org/cgi-bin/carddisp.pl?gene=ENSG00000241956
**RNA5SP349**	RNA, 5S Ribosomal Pseudogene 349	Pseudogene	6	GC11M109120	0.91	https://www.genecards.org/cgi-bin/carddisp.pl?gene=RNA5SP349
**RNA5SP355**	RNA, 5S Ribosomal Pseudogene 355	Pseudogene	6	GC12P028505	0.91	https://www.genecards.org/cgi-bin/carddisp.pl?gene=RNA5SP355
**ENSG00000275443**		RNA Gene	6	GC15P095990	0.91	https://www.genecards.org/cgi-bin/carddisp.pl?gene=ENSG00000275443
**ENSG00000260773**		RNA Gene	6	GC15M066314	0.91	https://www.genecards.org/cgi-bin/carddisp.pl?gene=ENSG00000260773
**LINC02752**	Long Intergenic Non-Protein Coding RNA 2752	RNA Gene	6	GC11P011074	0.91	https://www.genecards.org/cgi-bin/carddisp.pl?gene=LINC02752

**ENSG00000282246**		Protein Coding	6	GC10P013613	0.91	https://www.genecards.org/cgi-bin/carddisp.pl?gene=ENSG00000282246
**RF00017-5093**		RNA Gene	5	GC05M171223	0.91	https://www.genecards.org/cgi-bin/carddisp.pl?gene=RF00017-5093
**ENSG00000287373**		RNA Gene	5	GC11P030773	0.91	https://www.genecards.org/cgi-bin/carddisp.pl?gene=ENSG00000287373
**ENSG00000286177**		RNA Gene	5	GC19M042500	0.91	https://www.genecards.org/cgi-bin/carddisp.pl?gene=ENSG00000286177
**ENSG00000285108**		RNA Gene	5	GC08M129685	0.91	https://www.genecards.org/cgi-bin/carddisp.pl?gene=ENSG00000285108
**lnc-TNIP2-1**		RNA Gene	5	GC04M002832	0.91	https://www.genecards.org/cgi-bin/carddisp.pl?gene=lnc-TNIP2-1
**CNN2P10**	Calponin 2 Pseudogene 10	Pseudogene	5	GC01P166796	0.91	https://www.genecards.org/cgi-bin/carddisp.pl?gene=CNN2P10
**MRPS33P3**	Mitochondrial Ribosomal Protein S33 Pseudogene 3	Pseudogene	5	GC04P115143	0.91	https://www.genecards.org/cgi-bin/carddisp.pl?gene=MRPS33P3

**lnc-GLIS3-2**		RNA Gene	5	GC09M003824	0.91	https://www.genecards.org/cgi-bin/carddisp.pl?gene=lnc-GLIS3-2
**LOC101928268**	Uncharacterized LOC101928268	Protein Coding	5	GC07P030460	0.91	https://www.genecards.org/cgi-bin/carddisp.pl?gene=LOC101928268
**CSP2**	CS Pseudogene 2	Pseudogene	5	GC03M107329	0.91	https://www.genecards.org/cgi-bin/carddisp.pl?gene=CSP2
**NPAP1P4**	Nuclear Pore Associated Protein 1 Pseudogene 4	Pseudogene	5	GC09M080357	0.91	https://www.genecards.org/cgi-bin/carddisp.pl?gene=NPAP1P4
**ENSG00000238156**		RNA Gene	5	GC06P075455	0.91	https://www.genecards.org/cgi-bin/carddisp.pl?gene=ENSG00000238156
**RPL21P34**	Ribosomal Protein L21 Pseudogene 34	Pseudogene	5	GC02P128217	0.91	https://www.genecards.org/cgi-bin/carddisp.pl?gene=RPL21P34
**lnc-KCNK7-4**		RNA Gene	5	GC11M065547	0.91	https://www.genecards.org/cgi-bin/carddisp.pl?gene=lnc-KCNK7-4
**ENSG00000173727**		Pseudogene	5	GC11P065799	0.91	https://www.genecards.org/cgi-bin/carddisp.pl?gene=ENSG00000173727

**ENSG00000279093**		Uncategorized	5	GC11P065561	0.91	https://www.genecards.org/cgi-bin/carddisp.pl?gene=ENSG00000279093
**ENSG00000273179**		RNA Gene	5	GC04P001161	0.91	https://www.genecards.org/cgi-bin/carddisp.pl?gene=ENSG00000273179
**ENSG00000273771**		RNA Gene	5	GC15M096080	0.91	https://www.genecards.org/cgi-bin/carddisp.pl?gene=ENSG00000273771
**LINC02680**	Long Intergenic Non-Protein Coding RNA 2680	RNA Gene	5	GC10P027542	0.91	https://www.genecards.org/cgi-bin/carddisp.pl?gene=LINC02680
**ENSG00000272588**		RNA Gene	5	GC04M000803	0.91	https://www.genecards.org/cgi-bin/carddisp.pl?gene=ENSG00000272588
**lnc-TIMM29-1**		RNA Gene	4	GC19P010385	0.91	https://www.genecards.org/cgi-bin/carddisp.pl?gene=lnc-TIMM29-1
**MARK2P15**	Microtubule Affinity Regulating Kinase 2 Pseudogene 15	Pseudogene	4	GC10M083311	0.91	https://www.genecards.org/cgi-bin/carddisp.pl?gene=MARK2P15
**RF00017-7373**		RNA Gene	4	GC08P143777	0.91	https://www.genecards.org/cgi-bin/carddisp.pl?gene=RF00017-7373

**RF00017-7375**	RNA Gene	4	GC08P143902	0.91	https://www.genecards.org/cgi-bin/carddisp.pl?gene=RF00017-7375
**lnc-CD47-7**	RNA Gene	4	GC03M107248	0.91	https://www.genecards.org/cgi-bin/carddisp.pl?gene=lnc-CD47-7
**piR-40096**	RNA Gene	4	GC06M027401	0.91	https://www.genecards.org/cgi-bin/carddisp.pl?gene=piR-40096
**ENSG00000288096**	RNA Gene	4	GC08P143888	0.91	https://www.genecards.org/cgi-bin/carddisp.pl?gene=ENSG00000288096
**lnc-BRD2-2**	RNA Gene	4	GC06P047354	0.91	https://www.genecards.org/cgi-bin/carddisp.pl?gene=lnc-BRD2-2
**lnc-BRD2-3**	RNA Gene	4	GC06P047355	0.91	https://www.genecards.org/cgi-bin/carddisp.pl?gene=lnc-BRD2-3
**piR-39858-250**	RNA Gene	4	GC16M053820	0.91	https://www.genecards.org/cgi-bin/carddisp.pl?gene=piR-39858-250
**ENSG00000287632**	RNA Gene	4	GC04M078669	0.91	https://www.genecards.org/cgi-bin/carddisp.pl?gene=ENSG00000287632

**RF00017-5095**	RNA Gene	4	GC05M171476	0.91	https://www.genecards.org/cgi-bin/carddisp.pl?gene=RF00017-5095
**lnc-SSSCA1-1**	RNA Gene	4	GC11P065812	0.91	https://www.genecards.org/cgi-bin/carddisp.pl?gene=lnc-SSSCA1-1
**FJ601676**	RNA Gene	4	GC09M115076	0.91	https://www.genecards.org/cgi-bin/carddisp.pl?gene=FJ601676
**lnc-SOCS2-6**	RNA Gene	4	GC12P093788	0.91	https://www.genecards.org/cgi-bin/carddisp.pl?gene=lnc-SOCS2-6
**ENSG00000285144**	RNA Gene	4	GC20P038268	0.91	https://www.genecards.org/cgi-bin/carddisp.pl?gene=ENSG00000285144
**ENSG00000285577**	RNA Gene	4	GC02M033274	0.91	https://www.genecards.org/cgi-bin/carddisp.pl?gene=ENSG00000285577
**ENSG00000285914**	RNA Gene	4	GC05M171724	0.91	https://www.genecards.org/cgi-bin/carddisp.pl?gene=ENSG00000285914
**ENSG00000286910**	RNA Gene	4	GC08P008665	0.91	https://www.genecards.org/cgi-bin/carddisp.pl?gene=ENSG00000286910

**lnc-APOBEC4-5**	RNA Gene	4	GC01M183931	0.91	https://www.genecards.org/cgi-bin/carddisp.pl?gene=lnc-APOBEC4-5
**piR-31751**	RNA Gene	4	GC06P029097	0.91	https://www.genecards.org/cgi-bin/carddisp.pl?gene=piR-31751
**lnc-B9D2-2**	RNA Gene	4	GC19M041332	0.91	https://www.genecards.org/cgi-bin/carddisp.pl?gene=lnc-B9D2-2
**lnc-CTDSP2-7**	RNA Gene	4	GC12M058885	0.91	https://www.genecards.org/cgi-bin/carddisp.pl?gene=lnc-CTDSP2-7
**lnc-TACC3-2**	RNA Gene	4	GC04P001691	0.91	https://www.genecards.org/cgi-bin/carddisp.pl?gene=lnc-TACC3-2
**lnc-STAB1-1**	RNA Gene	4	GC03P052535	0.91	https://www.genecards.org/cgi-bin/carddisp.pl?gene=lnc-STAB1-1
**RF00017-7406**	RNA Gene	4	GC09P004280	0.91	https://www.genecards.org/cgi-bin/carddisp.pl?gene=RF00017-7406
**lnc-ZMYND8-10**	RNA Gene	4	GC20M047439	0.91	https://www.genecards.org/cgi-bin/carddisp.pl?gene=lnc-ZMYND8-10

**piR-34822-068**	RNA Gene	4	GC12P058 911	0.91	https://www.genecards.org/cgi-bin/carddisp.pl?gene=piR-34822-068
**lnc-YKT6-2**	RNA Gene	4	GC07P044 321	0.91	https://www.genecards.org/cgi-bin/carddisp.pl?gene=lnc-YKT6-2
**lnc-ETS2-8**	RNA Gene	4	GC21P038 760	0.91	https://www.genecards.org/cgi-bin/carddisp.pl?gene=lnc-ETS2-8
**piR-42694-043**	RNA Gene	4	GC12P122 940	0.91	https://www.genecards.org/cgi-bin/carddisp.pl?gene=piR-42694-043
**lnc-DYNC1I1-4**	RNA Gene	4	GC07P096 113	0.91	https://www.genecards.org/cgi-bin/carddisp.pl?gene=lnc-DYNC1I1-4
**piR-43098**	RNA Gene	4	GC02M156 453	0.91	https://www.genecards.org/cgi-bin/carddisp.pl?gene=piR-43098
**lnc-TTLL12-1**	RNA Gene	4	GC22M045 625	0.91	https://www.genecards.org/cgi-bin/carddisp.pl?gene=lnc-TTLL12-1
**piR-50208**	RNA Gene	4	GC15P073 696	0.91	https://www.genecards.org/cgi-bin/carddisp.pl?gene=piR-50208

**lnc-IQCH-7**	RNA Gene	4	GC15P073029	0.91	https://www.genecards.org/cgi-bin/carddisp.pl?gene=lnc-IQCH-7
**lnc-FAM83C-2**	RNA Gene	4	GC20M035345	0.91	https://www.genecards.org/cgi-bin/carddisp.pl?gene=lnc-FAM83C-2
**lnc-FAM83C-3**	RNA Gene	4	GC20M035303	0.91	https://www.genecards.org/cgi-bin/carddisp.pl?gene=lnc-FAM83C-3
**HSALNG0123133**	RNA Gene	4	GC19M002173	0.91	https://www.genecards.org/cgi-bin/carddisp.pl?gene=HSALNG0123133
**ENSG00000238232**	RNA Gene	4	GC01P219557	0.91	https://www.genecards.org/cgi-bin/carddisp.pl?gene=ENSG00000238232
**ENSG00000222658**	RNA Gene	4	GC09P012877	0.91	https://www.genecards.org/cgi-bin/carddisp.pl?gene=ENSG00000222658
**NONHSAG011935.2**	RNA Gene	4	GC12P093773	0.91	https://www.genecards.org/cgi-bin/carddisp.pl?gene=NONHSAG011935.2
**ENSG00000219682**	Pseudogene	4	GC06P025140	0.91	https://www.genecards.org/cgi-bin/carddisp.pl?gene=ENSG00000219682

**lnc-KIF12-2**		RNA Gene	4	GC09M114145	0.91	https://www.genecards.org/cgi-bin/carddisp.pl?gene=lnc-KIF12-2
**lnc-KIF12-3**		RNA Gene	4	GC09M114159	0.91	https://www.genecards.org/cgi-bin/carddisp.pl?gene=lnc-KIF12-3
**SNODB718**		RNA Gene	4	GC04P078641	0.91	https://www.genecards.org/cgi-bin/carddisp.pl?gene=SNODB718
**LOC107987014**	Uncharacterized LOC107987014	Protein Coding	4	GC09P116565	0.91	https://www.genecards.org/cgi-bin/carddisp.pl?gene=LOC107987014
**RF00017-2809**		RNA Gene	4	GC19P010621	0.91	https://www.genecards.org/cgi-bin/carddisp.pl?gene=RF00017-2809
**lnc-PRPF18-2**		RNA Gene	4	GC10P013683	0.91	https://www.genecards.org/cgi-bin/carddisp.pl?gene=lnc-PRPF18-2
**ENSG00000251446**		RNA Gene	4	GC05M176735	0.91	https://www.genecards.org/cgi-bin/carddisp.pl?gene=ENSG00000251446
**lnc-PLEC-4**		RNA Gene	4	GC08M143901	0.91	https://www.genecards.org/cgi-bin/carddisp.pl?gene=lnc-PLEC-4

**L13304-005**	RNA Gene	4	GC11M065320	0.91	https://www.genecards.org/cgi-bin/carddisp.pl?gene=L13304-005
**ENSG00000239793**	Pseudogene	4	GC04P078769	0.91	https://www.genecards.org/cgi-bin/carddisp.pl?gene=ENSG00000239793
**piR-50446**	RNA Gene	4	GC06P029236	0.91	https://www.genecards.org/cgi-bin/carddisp.pl?gene=piR-50446
**lnc-NCOA3-18**	RNA Gene	4	GC20P047472	0.91	https://www.genecards.org/cgi-bin/carddisp.pl?gene=lnc-NCOA3-18
**ENSG00000277135**	RNA Gene	4	GC15M096139	0.91	https://www.genecards.org/cgi-bin/carddisp.pl?gene=ENSG00000277135
**ENSG00000277749**	RNA Gene	4	GC15M074311	0.91	https://www.genecards.org/cgi-bin/carddisp.pl?gene=ENSG00000277749
**ENSG00000277829**	RNA Gene	4	GC20M038233	0.91	https://www.genecards.org/cgi-bin/carddisp.pl?gene=ENSG00000277829
**ENSG00000273254**	RNA Gene	4	GC21M029024	0.91	https://www.genecards.org/cgi-bin/carddisp.pl?gene=ENSG00000273254

**ENSG00000264997**	RNA Gene	4	GC11M109263	0.91	https://www.genecards.org/cgi-bin/carddisp.pl?gene=ENSG00000264997
**lnc-ADD1-6**	RNA Gene	4	GC04P002793	0.91	https://www.genecards.org/cgi-bin/carddisp.pl?gene=lnc-ADD1-6
**lnc-ADD2-2**	RNA Gene	4	GC02M070456	0.91	https://www.genecards.org/cgi-bin/carddisp.pl?gene=lnc-ADD2-2
**ENSG00000280904**	RNA Gene	4	GC03P052817	0.91	https://www.genecards.org/cgi-bin/carddisp.pl?gene=ENSG00000280904
**lnc-SMYD3-5**	RNA Gene	3	GC01M245591	0.91	https://www.genecards.org/cgi-bin/carddisp.pl?gene=lnc-SMYD3-5
**piR-32559-090**	RNA Gene	3	GC12P062759	0.91	https://www.genecards.org/cgi-bin/carddisp.pl?gene=piR-32559-090
**lnc-RPGRIP1L-2**	RNA Gene	3	GC16M053746	0.91	https://www.genecards.org/cgi-bin/carddisp.pl?gene=lnc-RPGRIP1L-2
**RF00017-596**	RNA Gene	3	GC10P013694	0.91	https://www.genecards.org/cgi-bin/carddisp.pl?gene=RF00017-596

**5MWI_A-023**		RNA Gene	3	GC11M065574	0.91	https://www.genecards.org/cgi-bin/carddisp.pl?gene=5MWI_A-023
**ENSG00000283271**		RNA Gene	3	GC04P013051	0.91	https://www.genecards.org/cgi-bin/carddisp.pl?gene=ENSG00000283271
**ENSG00000283517**		RNA Gene	3	GC17M071829	0.91	https://www.genecards.org/cgi-bin/carddisp.pl?gene=ENSG00000283517
**RF00017-3161**		RNA Gene	3	GC02P033230	0.91	https://www.genecards.org/cgi-bin/carddisp.pl?gene=RF00017-3161
**lnc-COLGALT2-6**		RNA Gene	3	GC01M183929	0.91	https://www.genecards.org/cgi-bin/carddisp.pl?gene=lnc-COLGALT2-6
**lnc-GGCT-3**		RNA Gene	3	GC07M030470	0.91	https://www.genecards.org/cgi-bin/carddisp.pl?gene=lnc-GGCT-3
**piR-43105-319**		RNA Gene	3	GC19P010651	0.91	https://www.genecards.org/cgi-bin/carddisp.pl?gene=piR-43105-319
**H3P27**	H3 Histone Pseudogene 27	Pseudogene	3	GC06P075588	0.91	https://www.genecards.org/cgi-bin/carddisp.pl?gene=H3P27

**lnc-HFM1-3**		RNA Gene	3	GC01M091682	0.91	https://www.genecards.org/cgi-bin/carddisp.pl?gene=lnc-HFM1-3
**MS4A19P**	Membrane Spanning 4-Domains A19, Pseudogene	Pseudogene	3	GC11P060578	0.91	https://www.genecards.org/cgi-bin/carddisp.pl?gene=MS4A19P
**LOC100288365**	Activating Transcription Factor 7 Interacting Protein 2 Pseudogene	Pseudogene	3	GC11P011138	0.91	https://www.genecards.org/cgi-bin/carddisp.pl?gene=LOC100288365
**RPL14P6**	RPL14 Pseudogene 6	Pseudogene	3	GC02P128206	0.91	https://www.genecards.org/cgi-bin/carddisp.pl?gene=RPL14P6
**HSALNG0059706**		RNA Gene	3	GC07M095971	0.91	https://www.genecards.org/cgi-bin/carddisp.pl?gene=HSALNG0059706
**HSALNG0026174**		RNA Gene	3	GC03M052679	0.91	https://www.genecards.org/cgi-bin/carddisp.pl?gene=HSALNG0026174
**MN308750**		RNA Gene	3	GC01P091719	0.91	https://www.genecards.org/cgi-bin/carddisp.pl?gene=MN308750
**LOC105372401**	Uncharacterized LOC105372401	RNA Gene	3	GC19M042510	0.91	https://www.genecards.org/cgi-bin/carddisp.pl?gene=LOC105372401

**lnc-KLHL42-2**		RNA Gene	3	GC12P027870	0.91	https://www.genecards.org/cgi-bin/carddisp.pl?gene=lnc-KLHL42-2
**NONHSAG001562.2**		RNA Gene	3	GC01P055065	0.91	https://www.genecards.org/cgi-bin/carddisp.pl?gene=NONHSAG001562.2
**piR-36756-038**		RNA Gene	3	GC02P033175	0.91	https://www.genecards.org/cgi-bin/carddisp.pl?gene=piR-36756-038
**SMC4P1**	Structural Maintenance Of Chromosomes 4 Pseudogene 1	Pseudogene	3	GC02P136546	0.91	https://www.genecards.org/cgi-bin/carddisp.pl?gene=SMC4P1
**lnc-NT5DC2-1**		RNA Gene	3	GC03M052536	0.91	https://www.genecards.org/cgi-bin/carddisp.pl?gene=lnc-NT5DC2-1
**LOC107986989**	Uncharacterized LOC107986989	RNA Gene	3	GC09P004301	0.91	https://www.genecards.org/cgi-bin/carddisp.pl?gene=LOC107986989
**ENSG00000256378**		Pseudogene	3	GC12M027935	0.91	https://www.genecards.org/cgi-bin/carddisp.pl?gene=ENSG00000256378
**lnc-PBRM1-2**		RNA Gene	3	GC03M052528	0.91	https://www.genecards.org/cgi-bin/carddisp.pl?gene=lnc-PBRM1-2

**RF00017-1169**	RNA Gene	3	GC12M062799	0.91	https://www.genecards.org/cgi-bin/carddisp.pl?gene=RF00017-1169
**piR-43107-151**	RNA Gene	3	GC02M033229	0.91	https://www.genecards.org/cgi-bin/carddisp.pl?gene=piR-43107-151
**piR-57133-366**	RNA Gene	2	GC20P035444	0.91	https://www.genecards.org/cgi-bin/carddisp.pl?gene=piR-57133-366
**piR-57137-006**	RNA Gene	2	GC11M011046	0.91	https://www.genecards.org/cgi-bin/carddisp.pl?gene=piR-57137-006
**piR-49204-003**	RNA Gene	2	GC12P123897	0.91	https://www.genecards.org/cgi-bin/carddisp.pl?gene=piR-49204-003
**RF00017-5583**	RNA Gene	2	GC06P049105	0.91	https://www.genecards.org/cgi-bin/carddisp.pl?gene=RF00017-5583
**lnc-BRD2-4**	RNA Gene	2	GC06P033079	0.91	https://www.genecards.org/cgi-bin/carddisp.pl?gene=lnc-BRD2-4
**piR-39858-279**	RNA Gene	2	GC17M066596	0.91	https://www.genecards.org/cgi-bin/carddisp.pl?gene=piR-39858-279

**piR-56497-029**	RNA Gene	2	GC12P027826	0.91	https://www.genecards.org/cgi-bin/carddisp.pl?gene=piR-56497-029
**FJ601684-189**	RNA Gene	2	GC17P066527	0.91	https://www.genecards.org/cgi-bin/carddisp.pl?gene=FJ601684-189
**lnc-ARHGDIB-2**	RNA Gene	2	GC12M014916	0.91	https://www.genecards.org/cgi-bin/carddisp.pl?gene=lnc-ARHGDIB-2
**ENSG00000286953**	RNA Gene	2	GC05M097799	0.91	https://www.genecards.org/cgi-bin/carddisp.pl?gene=ENSG00000286953
**piR-56368**	RNA Gene	2	GC15P074341	0.91	https://www.genecards.org/cgi-bin/carddisp.pl?gene=piR-56368
**AB372616**	RNA Gene	2	GC12P122868	0.91	https://www.genecards.org/cgi-bin/carddisp.pl?gene=AB372616
**piR-32023-046**	RNA Gene	2	GC11P076791	0.91	https://www.genecards.org/cgi-bin/carddisp.pl?gene=piR-32023-046
**ENSG00000286417**	RNA Gene	2	GC06P047588	0.91	https://www.genecards.org/cgi-bin/carddisp.pl?gene=ENSG00000286417

**MF281437-018**	RNA Gene	2	GC12P028506	0.91	https://www.genecards.org/cgi-bin/carddisp.pl?gene=MF281437-018
**lnc-TACC3-3**	RNA Gene	2	GC04P001683	0.91	https://www.genecards.org/cgi-bin/carddisp.pl?gene=lnc-TACC3-3
**lnc-TNC-7**	RNA Gene	2	GC09M116502	0.91	https://www.genecards.org/cgi-bin/carddisp.pl?gene=lnc-TNC-7
**HG984098**	RNA Gene	2	GC02M070438	0.91	https://www.genecards.org/cgi-bin/carddisp.pl?gene=HG984098
**MN297574**	RNA Gene	2	GC19P023704	0.91	https://www.genecards.org/cgi-bin/carddisp.pl?gene=MN297574
**lnc-EFCAB2-7**	RNA Gene	2	GC01P245584	0.91	https://www.genecards.org/cgi-bin/carddisp.pl?gene=lnc-EFCAB2-7
**piR-60429**	RNA Gene	2	GC04M001683	0.91	https://www.genecards.org/cgi-bin/carddisp.pl?gene=piR-60429
**piR-52471-007**	RNA Gene	2	GC04M001737	0.91	https://www.genecards.org/cgi-bin/carddisp.pl?gene=piR-52471-007

**RF00100-076**		RNA Gene	2	GC20M035382	0.91	https://www.genecards.org/cgi-bin/carddisp.pl?gene=RF00100-076
**RF00951-040**		RNA Gene	2	GC15M061438	0.91	https://www.genecards.org/cgi-bin/carddisp.pl?gene=RF00951-040
**HSALNG0117900**		RNA Gene	2	GC17M061574	0.91	https://www.genecards.org/cgi-bin/carddisp.pl?gene=HSALNG0117900
**lnc-NEK4-1**		RNA Gene	2	GC03M052785	0.91	https://www.genecards.org/cgi-bin/carddisp.pl?gene=lnc-NEK4-1
**piR-46368**		RNA Gene	2	GC20M035435	0.91	https://www.genecards.org/cgi-bin/carddisp.pl?gene=piR-46368
**LOC105369890**	Uncharacterized LOC105369890	RNA Gene	2	GC12P089920	0.91	https://www.genecards.org/cgi-bin/carddisp.pl?gene=LOC105369890
**LOC101929770**	Uncharacterized LOC101929770	RNA Gene	2	GC06P047545	0.91	https://www.genecards.org/cgi-bin/carddisp.pl?gene=LOC101929770
**piR-62029**		RNA Gene	2	GC07P044213	0.91	https://www.genecards.org/cgi-bin/carddisp.pl?gene=piR-62029

**lnc-NACA2-9**		RNA Gene	2	GC17M061567	0.91	https://www.genecards.org/cgi-bin/carddisp.pl?gene=lnc-NACA2-9
**piR-61945-087**		RNA Gene	2	GC12M014892	0.91	https://www.genecards.org/cgi-bin/carddisp.pl?gene=piR-61945-087
**LOC105373943**	Uncharacterized LOC105373943	RNA Gene	2	GC02M235676	0.91	https://www.genecards.org/cgi-bin/carddisp.pl?gene=LOC105373943
**ENSG00000244712**		Pseudogene	2	GC12M028564	0.91	https://www.genecards.org/cgi-bin/carddisp.pl?gene=ENSG00000244712
**RF00001-077**		RNA Gene	2	GC12P028507	0.91	https://www.genecards.org/cgi-bin/carddisp.pl?gene=RF00001-077
**LOC107986000**	Uncharacterized LOC107986000	RNA Gene	2	GC02P235730	0.91	https://www.genecards.org/cgi-bin/carddisp.pl?gene=LOC107986000
**TRUND-NNN9-1**	TRNA-Undetermined (NNN) 9-1	Pseudogene	2	GC02M070439	0.91	https://www.genecards.org/cgi-bin/carddisp.pl?gene=TRUND-NNN9-1
**piR-30534**		RNA Gene	2	GC08M143920	0.91	https://www.genecards.org/cgi-bin/carddisp.pl?gene=piR-30534

**lnc-LYPLAL1-11**	RNA Gene	2	GC01P219575	0.91	https://www.genecards.org/cgi-bin/carddisp.pl?gene=lnc-LYPLAL1-11
**NONHSAG012622.2**	RNA Gene	2	GC12P123850	0.91	https://www.genecards.org/cgi-bin/carddisp.pl?gene=NONHSAG012622.2
**RF00004-011**	RNA Gene	2	GC01P219603	0.91	https://www.genecards.org/cgi-bin/carddisp.pl?gene=RF00004-011
**ENSG00000278892**	Uncategorized	2	GC11P093071	0.91	https://www.genecards.org/cgi-bin/carddisp.pl?gene=ENSG00000278892
**ENSG00000279304**	Uncategorized	2	GC11P093133	0.91	https://www.genecards.org/cgi-bin/carddisp.pl?gene=ENSG00000279304
**ENSG00000274075**	RNA Gene	2	GC12M027824	0.91	https://www.genecards.org/cgi-bin/carddisp.pl?gene=ENSG00000274075
**lnc-ADAM10-10**	RNA Gene	2	GC15M060611	0.91	https://www.genecards.org/cgi-bin/carddisp.pl?gene=lnc-ADAM10-10
**piR-56497-036**	RNA Gene	1	GC12M123357	0.91	https://www.genecards.org/cgi-bin/carddisp.pl?gene=piR-56497-036

**LOC105377720**	Uncharacterized LOC105377720	RNA Gene	1	GC05P171494	0.91	https://www.genecards.org/cgi-bin/carddisp.pl?gene=LOC105377720
**LOC105377721**	Uncharacterized LOC105377721	RNA Gene	1	GC05M171469	0.91	https://www.genecards.org/cgi-bin/carddisp.pl?gene=LOC105377721
**LOC107986600**	Uncharacterized LOC107986600	RNA Gene	1	GC06P047873	0.91	https://www.genecards.org/cgi-bin/carddisp.pl?gene=LOC107986600
**LOC107985895**	Uncharacterized LOC107985895	RNA Gene	1	GC02M070479	0.91	https://www.genecards.org/cgi-bin/carddisp.pl?gene=LOC107985895
**ENSG00000259493**		Pseudogene	1	GC15M079559	0.91	https://www.genecards.org/cgi-bin/carddisp.pl?gene=ENSG00000259493
**TNFSF14**	TNF Superfamily Member 14	Protein Coding	41	GC19M006663	0.9	https://www.genecards.org/cgi-bin/carddisp.pl?gene=TNFSF14
**ITGAV**	Integrin Subunit Alpha V	Protein Coding	46	GC02P186589	0.86	https://www.genecards.org/cgi-bin/carddisp.pl?gene=ITGAV
**EGR1**	Early Growth Response 1	Protein Coding	44	GC05P138465	0.84	https://www.genecards.org/cgi-bin/carddisp.pl?gene=EGR1

**SULF1**	Sulfatase 1	Protein Coding	41	GC08P069466	0.84	https://www.genecards.org/cgi-bin/carddisp.pl?gene=SULF1
**ADAM15**	ADAM Metallopeptidase Domain 15	Protein Coding	41	GC01P155023	0.83	https://www.genecards.org/cgi-bin/carddisp.pl?gene=ADAM15
**SLC2A1**	Solute Carrier Family 2 Member 1	Protein Coding	52	GC01M042925	0.8	https://www.genecards.org/cgi-bin/carddisp.pl?gene=SLC2A1
**SOCS3**	Suppressor Of Cytokine Signaling 3	Protein Coding	44	GC17M078356	0.8	https://www.genecards.org/cgi-bin/carddisp.pl?gene=SOCS3
**TSLP**	Thymic Stromal Lymphopoietin	Protein Coding	38	GC05P111070	0.8	https://www.genecards.org/cgi-bin/carddisp.pl?gene=TSLP
**HDAC2**	Histone Deacetylase 2	Protein Coding	51	GC06M113933	0.79	https://www.genecards.org/cgi-bin/carddisp.pl?gene=HDAC2
**NTRK1**	Neurotrophic Receptor Tyrosine Kinase 1	Protein Coding	48	GC01P156786	0.79	https://www.genecards.org/cgi-bin/carddisp.pl?gene=NTRK1
**ATG7**	Autophagy Related 7	Protein Coding	43	GC03P011273	0.78	https://www.genecards.org/cgi-bin/carddisp.pl?gene=ATG7

**MIR136**	MicroRNA 136	RNA Gene	19	GC14P104582	0.78	https://www.genecards.org/cgi-bin/carddisp.pl?gene=MIR136
**KLF3-AS1**	KLF3 Antisense RNA 1	RNA Gene	17	GC04M038618	0.78	https://www.genecards.org/cgi-bin/carddisp.pl?gene=KLF3-AS1
**S100A4**	S100 Calcium Binding Protein A4	Protein Coding	44	GC01M153543	0.78	https://www.genecards.org/cgi-bin/carddisp.pl?gene=S100A4
**PRKCZ**	Protein Kinase C Zeta	Protein Coding	48	GC01P002050	0.77	https://www.genecards.org/cgi-bin/carddisp.pl?gene=PRKCZ
**ANG**	Angiogenin	Protein Coding	45	GC14P020830	0.77	https://www.genecards.org/cgi-bin/carddisp.pl?gene=ANG
**GC**	GC Vitamin D Binding Protein	Protein Coding	43	GC04M071741	0.77	https://www.genecards.org/cgi-bin/carddisp.pl?gene=GC
**JUNB**	JunB Proto-Oncogene, AP-1 Transcription Factor Subunit	Protein Coding	41	GC19P012791	0.77	https://www.genecards.org/cgi-bin/carddisp.pl?gene=JUNB
**PADI2**	Peptidyl Arginine Deiminase 2	Protein Coding	41	GC01M017066	0.76	https://www.genecards.org/cgi-bin/carddisp.pl?gene=PADI2

**S100A11**	S100 Calcium Binding Protein A11	Protein Coding	41	GC01M152032	0.76	https://www.genecards.org/cgi-bin/carddisp.pl?gene=S100A11
**CD55**	CD55 Molecule (Cromer Blood Group)	Protein Coding	47	GC01P207321	0.73	https://www.genecards.org/cgi-bin/carddisp.pl?gene=CD55
**DEFB4A**	Defensin Beta 4A	Protein Coding	37	GC08P007895	0.73	https://www.genecards.org/cgi-bin/carddisp.pl?gene=DEFB4A
**MIR92A1**	MicroRNA 92a-1	RNA Gene	19	GC13P091431	0.73	https://www.genecards.org/cgi-bin/carddisp.pl?gene=MIR92A1
**CXCL16**	C-X-C Motif Chemokine Ligand 16	Protein Coding	40	GC17M004733	0.73	https://www.genecards.org/cgi-bin/carddisp.pl?gene=CXCL16
**ALOX5AP**	Arachidonate 5-Lipoxygenase Activating Protein	Protein Coding	44	GC13P030713	0.73	https://www.genecards.org/cgi-bin/carddisp.pl?gene=ALOX5AP
**TNFSF12**	TNF Superfamily Member 12	Protein Coding	40	GC17P008027	0.73	https://www.genecards.org/cgi-bin/carddisp.pl?gene=TNFSF12
**CCN1**	Cellular Communication Network Factor 1	Protein Coding	31	GC01P085581	0.73	https://www.genecards.org/cgi-bin/carddisp.pl?gene=CCN1

**CPOX**	Coproporphyrinogen Oxidase	Protein Coding	43	GC03M098576	0.7	https://www.genecards.org/cgi-bin/carddisp.pl?gene=CPOX
**PDGFRA**	Platelet Derived Growth Factor Receptor Alpha	Protein Coding	55	GC04P054229	0.7	https://www.genecards.org/cgi-bin/carddisp.pl?gene=PDGFRA
**MAP3K7**	Mitogen-Activated Protein Kinase Kinase Kinase 7	Protein Coding	51	GC06M090513	0.7	https://www.genecards.org/cgi-bin/carddisp.pl?gene=MAP3K7
**MAP2K3**	Mitogen-Activated Protein Kinase Kinase 3	Protein Coding	49	GC17P026749	0.7	https://www.genecards.org/cgi-bin/carddisp.pl?gene=MAP2K3
**ANXA2**	Annexin A2	Protein Coding	48	GC15M060347	0.7	https://www.genecards.org/cgi-bin/carddisp.pl?gene=ANXA2
**ELF3**	E74 Like ETS Transcription Factor 3	Protein Coding	39	GC01P202007	0.7	https://www.genecards.org/cgi-bin/carddisp.pl?gene=ELF3
**F2R**	Coagulation Factor II Thrombin Receptor	Protein Coding	46	GC05P076716	0.68	https://www.genecards.org/cgi-bin/carddisp.pl?gene=F2R
**MIR193B**	MicroRNA 193b	RNA Gene	20	GC16P014307	0.68	https://www.genecards.org/cgi-bin/carddisp.pl?gene=MIR193B

**CD151**	CD151 Molecule (Raph Blood Group)	Protein Coding	45	GC11P000895	0.67	https://www.genecards.org/cgi-bin/carddisp.pl?gene=CD151
**BMP3**	Bone Morphogenetic Protein 3	Protein Coding	42	GC04P081030	0.66	https://www.genecards.org/cgi-bin/carddisp.pl?gene=BMP3
**CAPN2**	Calpain 2	Protein Coding	47	GC01P223701	0.65	https://www.genecards.org/cgi-bin/carddisp.pl?gene=CAPN2
**NRP1**	Neuropilin 1	Protein Coding	47	GC10M033177	0.65	https://www.genecards.org/cgi-bin/carddisp.pl?gene=NRP1
**FST**	Follistatin	Protein Coding	46	GC05P053480	0.65	https://www.genecards.org/cgi-bin/carddisp.pl?gene=FST
**HSD11B2**	Hydroxysteroid 11-Beta Dehydrogenase 2	Protein Coding	45	GC16P067433	0.65	https://www.genecards.org/cgi-bin/carddisp.pl?gene=HSD11B2
**E2F2**	E2F Transcription Factor 2	Protein Coding	42	GC01M023527	0.65	https://www.genecards.org/cgi-bin/carddisp.pl?gene=E2F2
**AMBP**	Alpha-1-Microglobulin/Bikunin Precursor	Protein Coding	41	GC09M114060	0.65	https://www.genecards.org/cgi-bin/carddisp.pl?gene=AMBP

**GADD45B**	Growth Arrest And DNA Damage Inducible Beta	Protein Coding	39	GC19P002476	0.65	https://www.genecards.org/cgi-bin/carddisp.pl?gene=GADD45B
**FZD1**	Frizzled Class Receptor 1	Protein Coding	45	GC07P091264	0.65	https://www.genecards.org/cgi-bin/carddisp.pl?gene=FZD1
**GUSB**	Glucuronidase Beta	Protein Coding	47	GC07M065960	0.65	https://www.genecards.org/cgi-bin/carddisp.pl?gene=GUSB
**LTF**	Lactotransferrin	Protein Coding	43	GC03M046435	0.65	https://www.genecards.org/cgi-bin/carddisp.pl?gene=LTF
**IL24**	Interleukin 24	Protein Coding	42	GC01P206897	0.65	https://www.genecards.org/cgi-bin/carddisp.pl?gene=IL24
**CD58**	CD58 Molecule	Protein Coding	40	GC01M116514	0.65	https://www.genecards.org/cgi-bin/carddisp.pl?gene=CD58
**IL20**	Interleukin 20	Protein Coding	39	GC01P206866	0.65	https://www.genecards.org/cgi-bin/carddisp.pl?gene=IL20
**ANGPTL2**	Angiopoietin Like 2	Protein Coding	36	GC09M127087	0.65	https://www.genecards.org/cgi-bin/carddisp.pl?gene=ANGPTL2
**IKBKE**	Inhibitor Of Nuclear Factor Kappa B Kinase Subunit Epsilon	Protein Coding	45	GC01P206470	0.62	https://www.genecards.org/cgi-bin/carddisp.pl?gene=IKBKE

**FAM215A**	Family With Sequence Similarity 215 Member A	RNA Gene	22	GC17P043917	0.62	https://www.genecards.org/cgi-bin/carddisp.pl?gene=FAM215A
**MIR448**	MicroRNA 448	RNA Gene	13	GC0XP114823	0.62	https://www.genecards.org/cgi-bin/carddisp.pl?gene=MIR448
**PDGFB**	Platelet Derived Growth Factor Subunit B	Protein Coding	50	GC22M045657	0.61	https://www.genecards.org/cgi-bin/carddisp.pl?gene=PDGFB
**PGK 1.00**	Phosphoglycerate Kinase 1	Protein Coding	48	GC0XP077928	0.61	https://www.genecards.org/cgi-bin/carddisp.pl?gene=PGK1
**CA1**	Carbonic Anhydrase 1	Protein Coding	47	GC08M085327	0.61	https://www.genecards.org/cgi-bin/carddisp.pl?gene=CA1
**CD59**	CD59 Molecule (CD59 Blood Group)	Protein Coding	46	GC11M033704	0.61	https://www.genecards.org/cgi-bin/carddisp.pl?gene=CD59
**DDIT3**	DNA Damage Inducible Transcript 3	Protein Coding	45	GC12M057516	0.61	https://www.genecards.org/cgi-bin/carddisp.pl?gene=DDIT3
**CFP**	Complement Factor Properdin	Protein Coding	43	GC0XM047624	0.61	https://www.genecards.org/cgi-bin/carddisp.pl?gene=CFP
**ICAM3**	Intercellular Adhesion Molecule 3	Protein Coding	41	GC19M010335	0.61	https://www.genecards.org/cgi-bin/carddisp.pl?gene=ICAM3

**LGALS3BP**	Galectin 3 Binding Protein	Protein Coding	41	GC17M078971	0.61	https://www.genecards.org/cgi-bin/carddisp.pl?gene=LGALS3BP
**POMGNT2**	Protein O-Linked Mannose N-Acetylglucosaminyltransferase 2 (Beta 1,4-)	Protein Coding	36	GC03M043121	0.61	https://www.genecards.org/cgi-bin/carddisp.pl?gene=POMGNT2
**MYDGF**	Myeloid Derived Growth Factor	Protein Coding	34	GC19M004641	0.61	https://www.genecards.org/cgi-bin/carddisp.pl?gene=MYDGF
**CEMIP**	Cell Migration Inducing Hyaluronidase 1	Protein Coding	33	GC15P080779	0.61	https://www.genecards.org/cgi-bin/carddisp.pl?gene=CEMIP
**PROC**	Protein C, Inactivator Of Coagulation Factors Va And VIIIa	Protein Coding	49	GC02P127418	0.6	https://www.genecards.org/cgi-bin/carddisp.pl?gene=PROC
**HDC**	Histidine Decarboxylase	Protein Coding	44	GC15M050241	0.6	https://www.genecards.org/cgi-bin/carddisp.pl?gene=HDC
**TAC1**	Tachykinin Precursor 1	Protein Coding	43	GC07P097731	0.6	https://www.genecards.org/cgi-bin/carddisp.pl?gene=TAC1
**ITGA1**	Integrin Subunit Alpha 1	Protein Coding	41	GC05P052788	0.6	https://www.genecards.org/cgi-bin/carddisp.pl?gene=ITGA1

**PTX3**	Pentraxin 3	Protein Coding	41	GC03P157436	0.6	https://www.genecards.org/cgi-bin/carddisp.pl?gene=PTX3
**HAS3**	Hyaluronan Synthase 3	Protein Coding	40	GC16P069105	0.6	https://www.genecards.org/cgi-bin/carddisp.pl?gene=HAS3
**SOX4**	SRY-Box Transcription Factor 4	Protein Coding	43	GC06P021593	0.59	https://www.genecards.org/cgi-bin/carddisp.pl?gene=SOX4
**RNASE3**	Ribonuclease A Family Member 3	Protein Coding	40	GC14P020891	0.59	https://www.genecards.org/cgi-bin/carddisp.pl?gene=RNASE3
**EPHB4**	EPH Receptor B4	Protein Coding	52	GC07M100803	0.57	https://www.genecards.org/cgi-bin/carddisp.pl?gene=EPHB4
**CALR**	Calreticulin	Protein Coding	51	GC19P012938	0.57	https://www.genecards.org/cgi-bin/carddisp.pl?gene=CALR
**TERT**	Telomerase Reverse Transcriptase	Protein Coding	51	GC05M001253	0.57	https://www.genecards.org/cgi-bin/carddisp.pl?gene=TERT
**MAPK9**	Mitogen-Activated Protein Kinase 9	Protein Coding	48	GC05M180234	0.57	https://www.genecards.org/cgi-bin/carddisp.pl?gene=MAPK9

**GSN**	Gelsolin	Protein Coding	48	GC09P121201	0.57	https://www.genecards.org/cgi-bin/carddisp.pl?gene=GSN
**TAB2**	TGF-Beta Activated Kinase 1 (MAP3K7) Binding Protein 2	Protein Coding	47	GC06P149218	0.57	https://www.genecards.org/cgi-bin/carddisp.pl?gene=TAB2
**IRF1**	Interferon Regulatory Factor 1	Protein Coding	47	GC05M132481	0.57	https://www.genecards.org/cgi-bin/carddisp.pl?gene=IRF1
**FURIN**	Furin, Paired Basic Amino Acid Cleaving Enzyme	Protein Coding	46	GC15P090868	0.57	https://www.genecards.org/cgi-bin/carddisp.pl?gene=FURIN
**RHOA**	Ras Homolog Family Member A	Protein Coding	46	GC03M049359	0.57	https://www.genecards.org/cgi-bin/carddisp.pl?gene=RHOA
**CHRNA7**	Cholinergic Receptor Nicotinic Alpha 7 Subunit	Protein Coding	45	GC15P031923	0.57	https://www.genecards.org/cgi-bin/carddisp.pl?gene=CHRNA7
**ICAM2**	Intercellular Adhesion Molecule 2	Protein Coding	45	GC17M064002	0.57	https://www.genecards.org/cgi-bin/carddisp.pl?gene=ICAM2
**TRPA1**	Transient Receptor Potential Cation Channel Subfamily A Member 1	Protein Coding	45	GC08M072019	0.57	https://www.genecards.org/cgi-bin/carddisp.pl?gene=TRPA1
**MAP3K2**	Mitogen-Activated Protein Kinase Kinase Kinase 2	Protein Coding	44	GC02M127298	0.57	https://www.genecards.org/cgi-bin/carddisp.pl?gene=MAP3K2

**ANGPT2**	Angiopoietin 2	Protein Coding	44	GC08M006499	0.57	https://www.genecards.org/cgi-bin/carddisp.pl?gene=ANGPT2
**S1PR1**	Sphingosine-1-Phosphate Receptor 1	Protein Coding	44	GC01P101236	0.57	https://www.genecards.org/cgi-bin/carddisp.pl?gene=S1PR1
**TICAM1**	Toll Like Receptor Adaptor Molecule 1	Protein Coding	44	GC19M004815	0.57	https://www.genecards.org/cgi-bin/carddisp.pl?gene=TICAM1
**IGFBP4**	Insulin Like Growth Factor Binding Protein 4	Protein Coding	43	GC17P040443	0.57	https://www.genecards.org/cgi-bin/carddisp.pl?gene=IGFBP4
**SMAD7**	SMAD Family Member 7	Protein Coding	43	GC18M048919	0.57	https://www.genecards.org/cgi-bin/carddisp.pl?gene=SMAD7
**TRIB3**	Tribbles Pseudokinase 3	Protein Coding	43	GC20P000361	0.57	https://www.genecards.org/cgi-bin/carddisp.pl?gene=TRIB3
**CCL19**	C-C Motif Chemokine Ligand 19	Protein Coding	41	GC09M034692	0.57	https://www.genecards.org/cgi-bin/carddisp.pl?gene=CCL19
**FCGR1A**	Fc Fragment Of IgG Receptor Ia	Protein Coding	41	GC01P149754	0.57	https://www.genecards.org/cgi-bin/carddisp.pl?gene=FCGR1A

**BPI**	Bactericidal Permeability Increasing Protein	Protein Coding	41	GC20P038304	0.57	https://www.genecards.org/cgi-bin/carddisp.pl?gene=BPI
**IL17B**	Interleukin 17B	Protein Coding	41	GC05M149371	0.57	https://www.genecards.org/cgi-bin/carddisp.pl?gene=IL17B
**ACKR3**	Atypical Chemokine Receptor 3	Protein Coding	40	GC02P236537	0.57	https://www.genecards.org/cgi-bin/carddisp.pl?gene=ACKR3
**AIF1**	Allograft Inflammatory Factor 1	Protein Coding	39	GC06P047304	0.57	https://www.genecards.org/cgi-bin/carddisp.pl?gene=AIF1
**ADGRE5**	Adhesion G Protein-Coupled Receptor E5	Protein Coding	38	GC19P014381	0.57	https://www.genecards.org/cgi-bin/carddisp.pl?gene=ADGRE5
**ZC3H12A**	Zinc Finger CCCH-Type Containing 12A	Protein Coding	36	GC01P037474	0.57	https://www.genecards.org/cgi-bin/carddisp.pl?gene=ZC3H12A
**MIR31**	MicroRNA 31	RNA Gene	20	GC09M021513	0.57	https://www.genecards.org/cgi-bin/carddisp.pl?gene=MIR31
**MIR127**	MicroRNA 127	RNA Gene	20	GC14P104580	0.57	https://www.genecards.org/cgi-bin/carddisp.pl?gene=MIR127

**MIR199A2**	MicroRNA 199a-2	RNA Gene	19	GC01M172235	0.57	https://www.genecards.org/cgi-bin/carddisp.pl?gene=MIR199A2
**MIR148A**	MicroRNA 148a	RNA Gene	18	GC07M025993	0.57	https://www.genecards.org/cgi-bin/carddisp.pl?gene=MIR148A
**MIR33A**	MicroRNA 33a	RNA Gene	18	GC22P041900	0.57	https://www.genecards.org/cgi-bin/carddisp.pl?gene=MIR33A
**HDAC8**	Histone Deacetylase 8	Protein Coding	47	GC0XM072329	0.56	https://www.genecards.org/cgi-bin/carddisp.pl?gene=HDAC8
**TXN**	Thioredoxin	Protein Coding	45	GC09M110243	0.56	https://www.genecards.org/cgi-bin/carddisp.pl?gene=TXN
**LAIR1**	Leukocyte Associated Immunoglobulin Like Receptor 1	Protein Coding	41	GC19M054351	0.56	https://www.genecards.org/cgi-bin/carddisp.pl?gene=LAIR1
**CXCR6**	C-X-C Motif Chemokine Receptor 6	Protein Coding	40	GC03P045982	0.56	https://www.genecards.org/cgi-bin/carddisp.pl?gene=CXCR6
**MIR95**	MicroRNA 95	RNA Gene	16	GC04M008007	0.56	https://www.genecards.org/cgi-bin/carddisp.pl?gene=MIR95

**F2RL2**	Coagulation Factor II Thrombin Receptor Like 2	Protein Coding	41	GC05M076615	0.53	https://www.genecards.org/cgi-bin/carddisp.pl?gene=F2RL2
**C4BPA**	Complement Component 4 Binding Protein Alpha	Protein Coding	40	GC01P207105	0.53	https://www.genecards.org/cgi-bin/carddisp.pl?gene=C4BPA
**TYK2**	Tyrosine Kinase 2	Protein Coding	52	GC19M010350	0.51	https://www.genecards.org/cgi-bin/carddisp.pl?gene=TYK2
**ITGA2B**	Integrin Subunit Alpha 2b	Protein Coding	50	GC17M044388	0.51	https://www.genecards.org/cgi-bin/carddisp.pl?gene=ITGA2B
**STAT6**	Signal Transducer And Activator Of Transcription 6	Protein Coding	50	GC12M057095	0.51	https://www.genecards.org/cgi-bin/carddisp.pl?gene=STAT6
**MAP3K1**	Mitogen-Activated Protein Kinase Kinase Kinase 1	Protein Coding	49	GC05P056815	0.51	https://www.genecards.org/cgi-bin/carddisp.pl?gene=MAP3K1
**ITGA5**	Integrin Subunit Alpha 5	Protein Coding	48	GC12M054396	0.51	https://www.genecards.org/cgi-bin/carddisp.pl?gene=ITGA5
**ENTPD1**	Ectonucleoside Triphosphate Diphosphohydrolase 1	Protein Coding	46	GC10P095711	0.51	https://www.genecards.org/cgi-bin/carddisp.pl?gene=ENTPD1

**CD9**	CD9 Molecule	Protein Coding	44	GC12P008124	0.51	https://www.genecards.org/cgi-bin/carddisp.pl?gene=CD9
**TNFRSF12A**	TNF Receptor Superfamily Member 12A	Protein Coding	44	GC16P003018	0.51	https://www.genecards.org/cgi-bin/carddisp.pl?gene=TNFRSF12A
**EFNB2**	Ephrin B2	Protein Coding	44	GC13M106489	0.51	https://www.genecards.org/cgi-bin/carddisp.pl?gene=EFNB2
**DKK 2.00**	Dickkopf WNT Signaling Pathway Inhibitor 2	Protein Coding	43	GC04M106921	0.51	https://www.genecards.org/cgi-bin/carddisp.pl?gene=DKK2
**ADAM8**	ADAM Metallopeptidase Domain 8	Protein Coding	42	GC10M133262	0.51	https://www.genecards.org/cgi-bin/carddisp.pl?gene=ADAM8
**SLC3A2**	Solute Carrier Family 3 Member 2	Protein Coding	42	GC11P062856	0.51	https://www.genecards.org/cgi-bin/carddisp.pl?gene=SLC3A2
**THPO**	Thrombopoietin	Protein Coding	42	GC03M184371	0.51	https://www.genecards.org/cgi-bin/carddisp.pl?gene=THPO
**CD63**	CD63 Molecule	Protein Coding	41	GC12M055725	0.51	https://www.genecards.org/cgi-bin/carddisp.pl?gene=CD63

**CHRD**	Chordin	Protein Coding	41	GC03P184380	0.51	https://www.genecards.org/cgi-bin/carddisp.pl?gene=CHRD
**CD84**	CD84 Molecule	Protein Coding	41	GC01M160541	0.51	https://www.genecards.org/cgi-bin/carddisp.pl?gene=CD84
**SMAD5**	SMAD Family Member 5	Protein Coding	41	GC05P136132	0.51	https://www.genecards.org/cgi-bin/carddisp.pl?gene=SMAD5
**KERA**	Keratocan	Protein Coding	41	GC12M091050	0.51	https://www.genecards.org/cgi-bin/carddisp.pl?gene=KERA
**OGN**	Osteoglycin	Protein Coding	39	GC09M092383	0.51	https://www.genecards.org/cgi-bin/carddisp.pl?gene=OGN
**IL36A**	Interleukin 36 Alpha	Protein Coding	37	GC02P113005	0.51	https://www.genecards.org/cgi-bin/carddisp.pl?gene=IL36A
**TSPAN4**	Tetraspanin 4	Protein Coding	36	GC11P000894	0.51	https://www.genecards.org/cgi-bin/carddisp.pl?gene=TSPAN4
**DEFB103B**	Defensin Beta 103B	Protein Coding	30	GC08M007430	0.51	https://www.genecards.org/cgi-bin/carddisp.pl?gene=DEFB103B

**PRKCD**	Protein Kinase C Delta	Protein Coding	53	GC03P053156	0.5	https://www.genecards.org/cgi-bin/carddisp.pl?gene=PRKCD
**PCNA**	Proliferating Cell Nuclear Antigen	Protein Coding	51	GC20M005114	0.5	https://www.genecards.org/cgi-bin/carddisp.pl?gene=PCNA
**GRIN2B**	Glutamate Ionotropic Receptor NMDA Type Subunit 2B	Protein Coding	50	GC12M013437	0.5	https://www.genecards.org/cgi-bin/carddisp.pl?gene=GRIN2B
**YWHAE**	Tyrosine 3-Monooxygenase/Tryptophan 5-Monooxygenase Activation Protein Epsilon	Protein Coding	50	GC17M001346	0.5	https://www.genecards.org/cgi-bin/carddisp.pl?gene=YWHAE
**ABCA1**	ATP Binding Cassette Subfamily A Member 1	Protein Coding	48	GC09M104781	0.5	https://www.genecards.org/cgi-bin/carddisp.pl?gene=ABCA1
**MMP19**	Matrix Metallopeptidase 19	Protein Coding	47	GC12M055835	0.5	https://www.genecards.org/cgi-bin/carddisp.pl?gene=MMP19
**VEGFC**	Vascular Endothelial Growth Factor C	Protein Coding	47	GC04M176683	0.5	https://www.genecards.org/cgi-bin/carddisp.pl?gene=VEGFC
**PRDX2**	Peroxiredoxin 2	Protein Coding	46	GC19M012796	0.5	https://www.genecards.org/cgi-bin/carddisp.pl?gene=PRDX2

**ABCC4**	ATP Binding Cassette Subfamily C Member 4	Protein Coding	45	GC13M095019	0.5	https://www.genecards.org/cgi-bin/carddisp.pl?gene=ABCC4
**P2RX7**	Purinergic Receptor P2X 7	Protein Coding	45	GC12P122829	0.5	https://www.genecards.org/cgi-bin/carddisp.pl?gene=P2RX7
**TFDP1**	Transcription Factor Dp-1	Protein Coding	44	GC13P113584	0.5	https://www.genecards.org/cgi-bin/carddisp.pl?gene=TFDP1
**ANXA6**	Annexin A6	Protein Coding	43	GC05M151077	0.5	https://www.genecards.org/cgi-bin/carddisp.pl?gene=ANXA6
**E2F1**	E2F Transcription Factor 1	Protein Coding	43	GC20M033675	0.5	https://www.genecards.org/cgi-bin/carddisp.pl?gene=E2F1
**PRDX3**	Peroxiredoxin 3	Protein Coding	43	GC10M119167	0.5	https://www.genecards.org/cgi-bin/carddisp.pl?gene=PRDX3
**RAMP2**	Receptor Activity Modifying Protein 2	Protein Coding	41	GC17P042758	0.5	https://www.genecards.org/cgi-bin/carddisp.pl?gene=RAMP2
**MTDH**	Metadherin	Protein Coding	40	GC08P097643	0.5	https://www.genecards.org/cgi-bin/carddisp.pl?gene=MTDH

**ZFP36**	ZFP36 Ring Finger Protein	Protein Coding	39	GC19P039406	0.5	https://www.genecards.org/cgi-bin/carddisp.pl?gene=ZFP36
**CCL27**	C-C Motif Chemokine Ligand 27	Protein Coding	37	GC09M034662	0.5	https://www.genecards.org/cgi-bin/carddisp.pl?gene=CCL27
**SNTB1**	Syntrophin Beta 1	Protein Coding	37	GC08M120535	0.5	https://www.genecards.org/cgi-bin/carddisp.pl?gene=SNTB1
**CORIN**	Corin, Serine Peptidase	Protein Coding	44	GC04M047596	0.47	https://www.genecards.org/cgi-bin/carddisp.pl?gene=CORIN
**PDGFRB**	Platelet Derived Growth Factor Receptor Beta	Protein Coding	55	GC05M150113	0.46	https://www.genecards.org/cgi-bin/carddisp.pl?gene=PDGFRB
**MAP2K1**	Mitogen-Activated Protein Kinase Kinase 1	Protein Coding	54	GC15P066386	0.46	https://www.genecards.org/cgi-bin/carddisp.pl?gene=MAP2K1
**NTRK2**	Neurotrophic Receptor Tyrosine Kinase 2	Protein Coding	53	GC09P084668	0.46	https://www.genecards.org/cgi-bin/carddisp.pl?gene=NTRK2
**FLT4**	Fms Related Receptor Tyrosine Kinase 4	Protein Coding	52	GC05M180607	0.46	https://www.genecards.org/cgi-bin/carddisp.pl?gene=FLT4

**G6PD**	Glucose-6-Phosphate Dehydrogenase	Protein Coding	50	GC0XM154531	0.46	https://www.genecards.org/cgi-bin/carddisp.pl?gene=G6PD
**MSH6**	MutS Homolog 6	Protein Coding	50	GC02P047695	0.46	https://www.genecards.org/cgi-bin/carddisp.pl?gene=MSH6
**C3**	Complement C3	Protein Coding	47	GC19M006677	0.46	https://www.genecards.org/cgi-bin/carddisp.pl?gene=C3
**GFAP**	Glial Fibrillary Acidic Protein	Protein Coding	47	GC17M044905	0.46	https://www.genecards.org/cgi-bin/carddisp.pl?gene=GFAP
**CCNB1**	Cyclin B1	Protein Coding	47	GC05P069167	0.46	https://www.genecards.org/cgi-bin/carddisp.pl?gene=CCNB1
**HSPA8**	Heat Shock Protein Family A (Hsp70) Member 8	Protein Coding	47	GC11M123057	0.46	https://www.genecards.org/cgi-bin/carddisp.pl?gene=HSPA8
**IRS1**	Insulin Receptor Substrate 1	Protein Coding	47	GC02M226731	0.46	https://www.genecards.org/cgi-bin/carddisp.pl?gene=IRS1
**HSD3B2**	Hydroxy-Delta-5-Steroid Dehydrogenase, 3 Beta- And Steroid Delta-Isomerase 2	Protein Coding	46	GC01P119414	0.46	https://www.genecards.org/cgi-bin/carddisp.pl?gene=HSD3B2
**PTGER3**	Prostaglandin E Receptor 3	Protein Coding	46	GC01M070852	0.46	https://www.genecards.org/cgi-bin/carddisp.pl?gene=PTGER3

**FGF5**	Fibroblast Growth Factor 5	Protein Coding	45	GC04P080266	0.46	https://www.genecards.org/cgi-bin/carddisp.pl?gene=FGF5
**ANGPTL4**	Angiopoietin Like 4	Protein Coding	45	GC19P008363	0.46	https://www.genecards.org/cgi-bin/carddisp.pl?gene=ANGPTL4
**SEMA4A**	Semaphorin 4A	Protein Coding	45	GC01P156119	0.46	https://www.genecards.org/cgi-bin/carddisp.pl?gene=SEMA4A
**COL18A1**	Collagen Type XVIII Alpha 1 Chain	Protein Coding	45	GC21P045405	0.46	https://www.genecards.org/cgi-bin/carddisp.pl?gene=COL18A1
**POLG**	DNA Polymerase Gamma, Catalytic Subunit	Protein Coding	45	GC15M089316	0.46	https://www.genecards.org/cgi-bin/carddisp.pl?gene=POLG
**KLF4**	Kruppel Like Factor 4	Protein Coding	45	GC09M107484	0.46	https://www.genecards.org/cgi-bin/carddisp.pl?gene=KLF4
**CITED2**	Cbp/P300 Interacting Transactivator With Glu/Asp Rich Carboxy-Terminal Domain 2	Protein Coding	44	GC06M139371	0.46	https://www.genecards.org/cgi-bin/carddisp.pl?gene=CITED2
**ELK1**	ETS Transcription Factor ELK1	Protein Coding	44	GC0XM047635	0.46	https://www.genecards.org/cgi-bin/carddisp.pl?gene=ELK1

**IL12B**	Interleukin 12B	Protein Coding	44	GC05M159314	0.46	https://www.genecards.org/cgi-bin/carddisp.pl?gene=IL12B
**SKIL**	SKI Like Proto-Oncogene	Protein Coding	44	GC03P170357	0.46	https://www.genecards.org/cgi-bin/carddisp.pl?gene=SKIL
**ITGB7**	Integrin Subunit Beta 7	Protein Coding	44	GC12M053191	0.46	https://www.genecards.org/cgi-bin/carddisp.pl?gene=ITGB7
**PLA2G5**	Phospholipase A2 Group V	Protein Coding	44	GC01P020028	0.46	https://www.genecards.org/cgi-bin/carddisp.pl?gene=PLA2G5
**CD47**	CD47 Molecule	Protein Coding	43	GC03M108043	0.46	https://www.genecards.org/cgi-bin/carddisp.pl?gene=CD47
**CMA1**	Chymase 1	Protein Coding	43	GC14M024506	0.46	https://www.genecards.org/cgi-bin/carddisp.pl?gene=CMA1
**NTF3**	Neurotrophin 3	Protein Coding	43	GC12P005432	0.46	https://www.genecards.org/cgi-bin/carddisp.pl?gene=NTF3
**ID1**	Inhibitor Of DNA Binding 1, HLH Protein	Protein Coding	43	GC20P031605	0.46	https://www.genecards.org/cgi-bin/carddisp.pl?gene=ID1

**THBS4**	Thrombospondin 4	Protein Coding	43	GC05P079991	0.46	https://www.genecards.org/cgi-bin/carddisp.pl?gene=THBS4
**TIA1**	TIA1 Cytotoxic Granule Associated RNA Binding Protein	Protein Coding	43	GC02M070209	0.46	https://www.genecards.org/cgi-bin/carddisp.pl?gene=TIA1
**ADAMTS2**	ADAM Metallopeptidase With Thrombospondin Type 1 Motif 2	Protein Coding	42	GC05M179110	0.46	https://www.genecards.org/cgi-bin/carddisp.pl?gene=ADAMTS2
**MT2A**	Metallothionein 2A	Protein Coding	42	GC16P056627	0.46	https://www.genecards.org/cgi-bin/carddisp.pl?gene=MT2A
**LAMA5**	Laminin Subunit Alpha 5	Protein Coding	42	GC20M062307	0.46	https://www.genecards.org/cgi-bin/carddisp.pl?gene=LAMA5
**GRP**	Gastrin Releasing Peptide	Protein Coding	41	GC18P059220	0.46	https://www.genecards.org/cgi-bin/carddisp.pl?gene=GRP
**ELAVL1**	ELAV Like RNA Binding Protein 1	Protein Coding	41	GC19M007958	0.46	https://www.genecards.org/cgi-bin/carddisp.pl?gene=ELAVL1
**SEMA3C**	Semaphorin 3C	Protein Coding	41	GC07M080742	0.46	https://www.genecards.org/cgi-bin/carddisp.pl?gene=SEMA3C

**MARCO**	Macrophage Receptor With Collagenous Structure	Protein Coding	40	GC02P118942	0.46	https://www.genecards.org/cgi-bin/carddisp.pl?gene=MARCO
**HOXD9**	Homeobox D9	Protein Coding	40	GC02P176122	0.46	https://www.genecards.org/cgi-bin/carddisp.pl?gene=HOXD9
**HOMER1**	Homer Scaffold Protein 1	Protein Coding	40	GC05M079372	0.46	https://www.genecards.org/cgi-bin/carddisp.pl?gene=HOMER1
**AZU1**	Azurocidin 1	Protein Coding	39	GC19P000825	0.46	https://www.genecards.org/cgi-bin/carddisp.pl?gene=AZU1
**ADAMTS12**	ADAM Metallopeptidase With Thrombospondin Type 1 Motif 12	Protein Coding	39	GC05M033524	0.46	https://www.genecards.org/cgi-bin/carddisp.pl?gene=ADAMTS12
**C1QTNF3**	C1q And TNF Related 3	Protein Coding	38	GC05M034017	0.46	https://www.genecards.org/cgi-bin/carddisp.pl?gene=C1QTNF3
**RND1**	Rho Family GTPase 1	Protein Coding	38	GC12M048857	0.46	https://www.genecards.org/cgi-bin/carddisp.pl?gene=RND1
**CALCB**	Calcitonin Related Polypeptide Beta	Protein Coding	37	GC11P014904	0.46	https://www.genecards.org/cgi-bin/carddisp.pl?gene=CALCB

**A1BG**	Alpha-1-B Glycoprotein	Protein Coding	37	GC19M058345	0.46	https://www.genecards.org/cgi-bin/carddisp.pl?gene=A1BG
**IFI27**	Interferon Alpha Inducible Protein 27	Protein Coding	37	GC14P094104	0.46	https://www.genecards.org/cgi-bin/carddisp.pl?gene=IFI27
**OMD**	Osteomodulin	Protein Coding	37	GC09M092414	0.46	https://www.genecards.org/cgi-bin/carddisp.pl?gene=OMD
**ZBTB38**	Zinc Finger And BTB Domain Containing 38	Protein Coding	36	GC03P141324	0.46	https://www.genecards.org/cgi-bin/carddisp.pl?gene=ZBTB38
**VSIG4**	V-Set And Immunoglobulin Domain Containing 4	Protein Coding	36	GC0XM066021	0.46	https://www.genecards.org/cgi-bin/carddisp.pl?gene=VSIG4
**CPQ**	Carboxypeptidase Q	Protein Coding	35	GC08P096645	0.46	https://www.genecards.org/cgi-bin/carddisp.pl?gene=CPQ
**ITGBL1**	Integrin Subunit Beta Like 1	Protein Coding	35	GC13P101454	0.46	https://www.genecards.org/cgi-bin/carddisp.pl?gene=ITGBL1
**H2BC21**	H2B Clustered Histone 21	Protein Coding	31	GC01M149950	0.46	https://www.genecards.org/cgi-bin/carddisp.pl?gene=H2BC21

**SELENOS**	Selenoprotein S	Protein Coding	31	GC15M103936	0.46	https://www.genecards.org/cgi-bin/carddisp.pl?gene=SELENOS
**H3C14**	H3 Clustered Histone 14	Protein Coding	29	GC01M149963	0.46	https://www.genecards.org/cgi-bin/carddisp.pl?gene=H3C14
**MIR590**	MicroRNA 590	RNA Gene	18	GC07P074191	0.46	https://www.genecards.org/cgi-bin/carddisp.pl?gene=MIR590
**MIR582**	MicroRNA 582	RNA Gene	17	GC05M059703	0.46	https://www.genecards.org/cgi-bin/carddisp.pl?gene=MIR582
**MIR194-1**	MicroRNA 194-1	RNA Gene	16	GC01M220118	0.46	https://www.genecards.org/cgi-bin/carddisp.pl?gene=MIR194-1
**PIK3CD**	Phosphatidylinositol-4,5-Bisphosphate 3-Kinase Catalytic Subunit Delta	Protein Coding	53	GC01P009629	0.4	https://www.genecards.org/cgi-bin/carddisp.pl?gene=PIK3CD
**CSNK2A1**	Casein Kinase 2 Alpha 1	Protein Coding	51	GC20M000472	0.4	https://www.genecards.org/cgi-bin/carddisp.pl?gene=CSNK2A1
**SLC9A1**	Solute Carrier Family 9 Member A1	Protein Coding	51	GC01M027109	0.4	https://www.genecards.org/cgi-bin/carddisp.pl?gene=SLC9A1

**HSPB1**	Heat Shock Protein Family B (Small) Member 1	Protein Coding	51	GC07P076302	0.4	https://www.genecards.org/cgi-bin/carddisp.pl?gene=HSPB1
**ZAP70**	Zeta Chain Of T Cell Receptor Associated Protein Kinase 70	Protein Coding	51	GC02P097696	0.4	https://www.genecards.org/cgi-bin/carddisp.pl?gene=ZAP70
**BMPR1B**	Bone Morphogenetic Protein Receptor Type 1B	Protein Coding	50	GC04P094757	0.4	https://www.genecards.org/cgi-bin/carddisp.pl?gene=BMPR1B
**MYB**	MYB Proto-Oncogene, Transcription Factor	Protein Coding	50	GC06P135180	0.4	https://www.genecards.org/cgi-bin/carddisp.pl?gene=MYB
**PRKCH**	Protein Kinase C Eta	Protein Coding	50	GC14P061187	0.4	https://www.genecards.org/cgi-bin/carddisp.pl?gene=PRKCH
**RARB**	Retinoic Acid Receptor Beta	Protein Coding	50	GC03P024830	0.4	https://www.genecards.org/cgi-bin/carddisp.pl?gene=RARB
**ETS1**	ETS Proto-Oncogene 1, Transcription Factor	Protein Coding	49	GC11M128458	0.4	https://www.genecards.org/cgi-bin/carddisp.pl?gene=ETS1
**GRIN1**	Glutamate Ionotropic Receptor NMDA Type Subunit 1	Protein Coding	49	GC09P137138	0.4	https://www.genecards.org/cgi-bin/carddisp.pl?gene=GRIN1

**RRM2B**	Ribonucleotide Reductase Regulatory TP53 Inducible Subunit M2B	Protein Coding	49	GC08M102204	0.4	https://www.genecards.org/cgi-bin/carddisp.pl?gene=RRM2B
**KCNMA1**	Potassium Calcium-Activated Channel Subfamily M Alpha 1	Protein Coding	49	GC10M076869	0.4	https://www.genecards.org/cgi-bin/carddisp.pl?gene=KCNMA1
**ITGA6**	Integrin Subunit Alpha 6	Protein Coding	49	GC02P172427	0.4	https://www.genecards.org/cgi-bin/carddisp.pl?gene=ITGA6
**STK11**	Serine/Threonine Kinase 11	Protein Coding	49	GC19P001177	0.4	https://www.genecards.org/cgi-bin/carddisp.pl?gene=STK11
**ALDOA**	Aldolase, Fructose-Bisphosphate A	Protein Coding	48	GC16P030064	0.4	https://www.genecards.org/cgi-bin/carddisp.pl?gene=ALDOA
**CYP3A4**	Cytochrome P450 Family 3 Subfamily A Member 4	Protein Coding	48	GC07M099759	0.4	https://www.genecards.org/cgi-bin/carddisp.pl?gene=CYP3A4
**EEF2**	Eukaryotic Translation Elongation Factor 2	Protein Coding	48	GC19M003976	0.4	https://www.genecards.org/cgi-bin/carddisp.pl?gene=EEF2
**CDH11**	Cadherin 11	Protein Coding	48	GC16M064882	0.4	https://www.genecards.org/cgi-bin/carddisp.pl?gene=CDH11

**HPRT1**	Hypoxanthine Phosphoribosyltransferase 1	Protein Coding	48	GC0XP134460	0.4	https://www.genecards.org/cgi-bin/carddisp.pl?gene=HPRT1
**SCNN1B**	Sodium Channel Epithelial 1 Subunit Beta	Protein Coding	48	GC16P023278	0.4	https://www.genecards.org/cgi-bin/carddisp.pl?gene=SCNN1B
**SLC2A3**	Solute Carrier Family 2 Member 3	Protein Coding	48	GC12M007919	0.4	https://www.genecards.org/cgi-bin/carddisp.pl?gene=SLC2A3
**REN**	Renin	Protein Coding	48	GC01M204154	0.4	https://www.genecards.org/cgi-bin/carddisp.pl?gene=REN
**GDNF**	Glial Cell Derived Neurotrophic Factor	Protein Coding	47	GC05M037812	0.4	https://www.genecards.org/cgi-bin/carddisp.pl?gene=GDNF
**CAMK2G**	Calcium/Calmodulin Dependent Protein Kinase II Gamma	Protein Coding	47	GC10M073812	0.4	https://www.genecards.org/cgi-bin/carddisp.pl?gene=CAMK2G
**MAP3K3**	Mitogen-Activated Protein Kinase Kinase Kinase 3	Protein Coding	47	GC17P063622	0.4	https://www.genecards.org/cgi-bin/carddisp.pl?gene=MAP3K3
**ESRRA**	Estrogen Related Receptor Alpha	Protein Coding	47	GC11P064305	0.4	https://www.genecards.org/cgi-bin/carddisp.pl?gene=ESRRA

**ADAM9**	ADAM Metallopeptidase Domain 9	Protein Coding	47	GC08P038996	0.4	https://www.genecards.org/cgi-bin/carddisp.pl?gene=ADAM9
**EPHX1**	Epoxide Hydrolase 1	Protein Coding	47	GC01P225810	0.4	https://www.genecards.org/cgi-bin/carddisp.pl?gene=EPHX1
**CYBB**	Cytochrome B-245 Beta Chain	Protein Coding	47	GC0XP037780	0.4	https://www.genecards.org/cgi-bin/carddisp.pl?gene=CYBB
**EPHB1**	EPH Receptor B1	Protein Coding	47	GC03P134598	0.4	https://www.genecards.org/cgi-bin/carddisp.pl?gene=EPHB1
**CHAT**	Choline O-Acetyltransferase	Protein Coding	47	GC10P049609	0.4	https://www.genecards.org/cgi-bin/carddisp.pl?gene=CHAT
**DDX58**	DExD/H-Box Helicase 58	Protein Coding	47	GC09M032455	0.4	https://www.genecards.org/cgi-bin/carddisp.pl?gene=DDX58
**SPHK1**	Sphingosine Kinase 1	Protein Coding	47	GC17P076376	0.4	https://www.genecards.org/cgi-bin/carddisp.pl?gene=SPHK1
**SLC9A3**	Solute Carrier Family 9 Member A3	Protein Coding	47	GC05M000472	0.4	https://www.genecards.org/cgi-bin/carddisp.pl?gene=SLC9A3

**IL7R**	Interleukin 7 Receptor	Protein Coding	47	GC05P035852	0.4	https://www.genecards.org/cgi-bin/carddisp.pl?gene=IL7R
**TTN**	Titin	Protein Coding	47	GC02M178525	0.4	https://www.genecards.org/cgi-bin/carddisp.pl?gene=TTN
**TYRO3**	TYRO3 Protein Tyrosine Kinase	Protein Coding	47	GC15P041557	0.4	https://www.genecards.org/cgi-bin/carddisp.pl?gene=TYRO3
**LEF1**	Lymphoid Enhancer Binding Factor 1	Protein Coding	47	GC04M108047	0.4	https://www.genecards.org/cgi-bin/carddisp.pl?gene=LEF1
**TFAP2A**	Transcription Factor AP-2 Alpha	Protein Coding	47	GC06M010393	0.4	https://www.genecards.org/cgi-bin/carddisp.pl?gene=TFAP2A
**EFNB1**	Ephrin B1	Protein Coding	47	GC0XP068828	0.4	https://www.genecards.org/cgi-bin/carddisp.pl?gene=EFNB1
**GFPT1**	Glutamine--Fructose-6-Phosphate Transaminase 1	Protein Coding	46	GC02M069283	0.4	https://www.genecards.org/cgi-bin/carddisp.pl?gene=GFPT1
**MAP3K11**	Mitogen-Activated Protein Kinase Kinase Kinase 11	Protein Coding	46	GC11M065598	0.4	https://www.genecards.org/cgi-bin/carddisp.pl?gene=MAP3K11

**CD46**	CD46 Molecule	Protein Coding	46	GC01P207752	0.4	https://www.genecards.org/cgi-bin/carddisp.pl?gene=CD46
**HMGA1**	High Mobility Group AT-Hook 1	Protein Coding	46	GC06P047417	0.4	https://www.genecards.org/cgi-bin/carddisp.pl?gene=HMGA1
**MYCN**	MYCN Proto-Oncogene, BHLH Transcription Factor	Protein Coding	46	GC02P015949	0.4	https://www.genecards.org/cgi-bin/carddisp.pl?gene=MYCN
**PTPN2**	Protein Tyrosine Phosphatase Non-Receptor Type 2	Protein Coding	46	GC18M017325	0.4	https://www.genecards.org/cgi-bin/carddisp.pl?gene=PTPN2
**SUMO1**	Small Ubiquitin Like Modifier 1	Protein Coding	46	GC02M202206	0.4	https://www.genecards.org/cgi-bin/carddisp.pl?gene=SUMO1
**DAG1**	Dystroglycan 1	Protein Coding	46	GC03P049482	0.4	https://www.genecards.org/cgi-bin/carddisp.pl?gene=DAG1
**FPR2**	Formyl Peptide Receptor 2	Protein Coding	45	GC19P051752	0.4	https://www.genecards.org/cgi-bin/carddisp.pl?gene=FPR2
**FHL2**	Four And A Half LIM Domains 2	Protein Coding	45	GC02M105343	0.4	https://www.genecards.org/cgi-bin/carddisp.pl?gene=FHL2

**BAP1**	BRCA1 Associated Protein 1	Protein Coding	45	GC03M052401	0.4	https://www.genecards.org/cgi-bin/carddisp.pl?gene=BAP1
**AICDA**	Activation Induced Cytidine Deaminase	Protein Coding	45	GC12M008602	0.4	https://www.genecards.org/cgi-bin/carddisp.pl?gene=AICDA
**ACLY**	ATP Citrate Lyase	Protein Coding	45	GC17M041866	0.4	https://www.genecards.org/cgi-bin/carddisp.pl?gene=ACLY
**C1QA**	Complement C1q A Chain	Protein Coding	45	GC01P022636	0.4	https://www.genecards.org/cgi-bin/carddisp.pl?gene=C1QA
**MMP15**	Matrix Metallopeptidase 15	Protein Coding	45	GC16P058025	0.4	https://www.genecards.org/cgi-bin/carddisp.pl?gene=MMP15
**GRK6**	G Protein-Coupled Receptor Kinase 6	Protein Coding	45	GC05P177403	0.4	https://www.genecards.org/cgi-bin/carddisp.pl?gene=GRK6
**IDO1**	Indoleamine 2,3-Dioxygenase 1	Protein Coding	45	GC08P039891	0.4	https://www.genecards.org/cgi-bin/carddisp.pl?gene=IDO1
**SCARB1**	Scavenger Receptor Class B Member 1	Protein Coding	45	GC12M124776	0.4	https://www.genecards.org/cgi-bin/carddisp.pl?gene=SCARB1

**PTGDS**	Prostaglandin D2 Synthase	Protein Coding	45	GC09P136982	0.4	https://www.genecards.org/cgi-bin/carddisp.pl?gene=PTGDS
**PTPRB**	Protein Tyrosine Phosphatase Receptor Type B	Protein Coding	45	GC12M070516	0.4	https://www.genecards.org/cgi-bin/carddisp.pl?gene=PTPRB
**IRF8**	Interferon Regulatory Factor 8	Protein Coding	45	GC16P085898	0.4	https://www.genecards.org/cgi-bin/carddisp.pl?gene=IRF8
**TCF7L2**	Transcription Factor 7 Like 2	Protein Coding	45	GC10P112950	0.4	https://www.genecards.org/cgi-bin/carddisp.pl?gene=TCF7L2
**KCNA2**	Potassium Voltage-Gated Channel Subfamily A Member 2	Protein Coding	45	GC01M110519	0.4	https://www.genecards.org/cgi-bin/carddisp.pl?gene=KCNA2
**RAG1**	Recombination Activating 1	Protein Coding	45	GC11P036514	0.4	https://www.genecards.org/cgi-bin/carddisp.pl?gene=RAG1
**SHC1**	SHC Adaptor Protein 1	Protein Coding	45	GC01M154962	0.4	https://www.genecards.org/cgi-bin/carddisp.pl?gene=SHC1
**ITCH**	Itchy E3 Ubiquitin Protein Ligase	Protein Coding	45	GC20P034363	0.4	https://www.genecards.org/cgi-bin/carddisp.pl?gene=ITCH

**THBS2**	Thrombospondin 2	Protein Coding	45	GC06M169215	0.4	https://www.genecards.org/cgi-bin/carddisp.pl?gene=THBS2
**VIPR1**	Vasoactive Intestinal Peptide Receptor 1	Protein Coding	45	GC03P042490	0.4	https://www.genecards.org/cgi-bin/carddisp.pl?gene=VIPR1
**C1QB**	Complement C1q B Chain	Protein Coding	44	GC01P022652	0.4	https://www.genecards.org/cgi-bin/carddisp.pl?gene=C1QB
**C1QC**	Complement C1q C Chain	Protein Coding	44	GC01P022643	0.4	https://www.genecards.org/cgi-bin/carddisp.pl?gene=C1QC
**CCNA2**	Cyclin A2	Protein Coding	44	GC04M121816	0.4	https://www.genecards.org/cgi-bin/carddisp.pl?gene=CCNA2
**FABP4**	Fatty Acid Binding Protein 4	Protein Coding	44	GC08M081478	0.4	https://www.genecards.org/cgi-bin/carddisp.pl?gene=FABP4
**LTB4R**	Leukotriene B4 Receptor	Protein Coding	44	GC14P024311	0.4	https://www.genecards.org/cgi-bin/carddisp.pl?gene=LTB4R
**AGRN**	Agrin	Protein Coding	44	GC01P001020	0.4	https://www.genecards.org/cgi-bin/carddisp.pl?gene=AGRN

**FZD10**	Frizzled Class Receptor 10	Protein Coding	44	GC12P130162	0.4	https://www.genecards.org/cgi-bin/carddisp.pl?gene=FZD10
**CENPE**	Centromere Protein E	Protein Coding	44	GC04M103105	0.4	https://www.genecards.org/cgi-bin/carddisp.pl?gene=CENPE
**PDGFA**	Platelet Derived Growth Factor Subunit A	Protein Coding	44	GC07M000497	0.4	https://www.genecards.org/cgi-bin/carddisp.pl?gene=PDGFA
**RXFP1**	Relaxin Family Peptide Receptor 1	Protein Coding	44	GC04P158315	0.4	https://www.genecards.org/cgi-bin/carddisp.pl?gene=RXFP1
**SMAD1**	SMAD Family Member 1	Protein Coding	44	GC04P145481	0.4	https://www.genecards.org/cgi-bin/carddisp.pl?gene=SMAD1
**HSF1**	Heat Shock Transcription Factor 1	Protein Coding	44	GC08P144291	0.4	https://www.genecards.org/cgi-bin/carddisp.pl?gene=HSF1
**PLXNB1**	Plexin B1	Protein Coding	44	GC03M048403	0.4	https://www.genecards.org/cgi-bin/carddisp.pl?gene=PLXNB1
**TPH1**	Tryptophan Hydroxylase 1	Protein Coding	44	GC11M018040	0.4	https://www.genecards.org/cgi-bin/carddisp.pl?gene=TPH1

**LAMA4**	Laminin Subunit Alpha 4	Protein Coding	44	GC06M112107	0.4	https://www.genecards.org/cgi-bin/carddisp.pl?gene=LAMA4
**SREBF1**	Sterol Regulatory Element Binding Transcription Factor 1	Protein Coding	44	GC17M017810	0.4	https://www.genecards.org/cgi-bin/carddisp.pl?gene=SREBF1
**ID2**	Inhibitor Of DNA Binding 2	Protein Coding	44	GC02P008772	0.4	https://www.genecards.org/cgi-bin/carddisp.pl?gene=ID2
**VIPR2**	Vasoactive Intestinal Peptide Receptor 2	Protein Coding	44	GC07M159028	0.4	https://www.genecards.org/cgi-bin/carddisp.pl?gene=VIPR2
**B3GAT1**	Beta-1,3-Glucuronyltransferase 1	Protein Coding	43	GC11M134378	0.4	https://www.genecards.org/cgi-bin/carddisp.pl?gene=B3GAT1
**BATF**	Basic Leucine Zipper ATF-Like Transcription Factor	Protein Coding	43	GC14P075523	0.4	https://www.genecards.org/cgi-bin/carddisp.pl?gene=BATF
**MAP3K4**	Mitogen-Activated Protein Kinase Kinase Kinase 4	Protein Coding	43	GC06P160991	0.4	https://www.genecards.org/cgi-bin/carddisp.pl?gene=MAP3K4
**AGTR2**	Angiotensin II Receptor Type 2	Protein Coding	43	GC0XP116170	0.4	https://www.genecards.org/cgi-bin/carddisp.pl?gene=AGTR2
**ACD**	ACD Shelterin Complex Subunit And Telomerase Recruitment Factor	Protein Coding	43	GC16M067658	0.4	https://www.genecards.org/cgi-bin/carddisp.pl?gene=ACD

**GAB2**	GRB2 Associated Binding Protein 2	Protein Coding	43	GC11M078215	0.4	https://www.genecards.org/cgi-bin/carddisp.pl?gene=GAB2
**FOXO4**	Forkhead Box O4	Protein Coding	43	GC0XP071095	0.4	https://www.genecards.org/cgi-bin/carddisp.pl?gene=FOXO4
**FSCN1**	Fascin Actin-Bundling Protein 1	Protein Coding	43	GC07P005592	0.4	https://www.genecards.org/cgi-bin/carddisp.pl?gene=FSCN1
**GSTA4**	Glutathione S-Transferase Alpha 4	Protein Coding	43	GC06M052977	0.4	https://www.genecards.org/cgi-bin/carddisp.pl?gene=GSTA4
**CPA6**	Carboxypeptidase A6	Protein Coding	43	GC08M067422	0.4	https://www.genecards.org/cgi-bin/carddisp.pl?gene=CPA6
**HDAC10**	Histone Deacetylase 10	Protein Coding	43	GC22M050245	0.4	https://www.genecards.org/cgi-bin/carddisp.pl?gene=HDAC10
**DHRS3**	Dehydrogenase/Reductase 3	Protein Coding	43	GC01M012567	0.4	https://www.genecards.org/cgi-bin/carddisp.pl?gene=DHRS3
**EEF1A1**	Eukaryotic Translation Elongation Factor 1 Alpha 1	Protein Coding	43	GC06M073515	0.4	https://www.genecards.org/cgi-bin/carddisp.pl?gene=EEF1A1

**CD209**	CD209 Molecule	Protein Coding	43	GC19M007739	0.4	https://www.genecards.org/cgi-bin/carddisp.pl?gene=CD209
**SIRPA**	Signal Regulatory Protein Alpha	Protein Coding	43	GC20P001894	0.4	https://www.genecards.org/cgi-bin/carddisp.pl?gene=SIRPA
**PLTP**	Phospholipid Transfer Protein	Protein Coding	43	GC20M045898	0.4	https://www.genecards.org/cgi-bin/carddisp.pl?gene=PLTP
**NRP2**	Neuropilin 2	Protein Coding	43	GC02P205681	0.4	https://www.genecards.org/cgi-bin/carddisp.pl?gene=NRP2
**NR2C2**	Nuclear Receptor Subfamily 2 Group C Member 2	Protein Coding	43	GC03P014947	0.4	https://www.genecards.org/cgi-bin/carddisp.pl?gene=NR2C2
**INHA**	Inhibin Subunit Alpha	Protein Coding	43	GC02P219569	0.4	https://www.genecards.org/cgi-bin/carddisp.pl?gene=INHA
**PHOX2B**	Paired Like Homeobox 2B	Protein Coding	43	GC04M041746	0.4	https://www.genecards.org/cgi-bin/carddisp.pl?gene=PHOX2B
**TNFRSF14**	TNF Receptor Superfamily Member 14	Protein Coding	43	GC01P002555	0.4	https://www.genecards.org/cgi-bin/carddisp.pl?gene=TNFRSF14

**TRAP1**	TNF Receptor Associated Protein 1	Protein Coding	43	GC16M003652	0.4	https://www.genecards.org/cgi-bin/carddisp.pl?gene=TRAP1
**TNFRSF10D**	TNF Receptor Superfamily Member 10d	Protein Coding	43	GC08M023135	0.4	https://www.genecards.org/cgi-bin/carddisp.pl?gene=TNFRSF10D
**LILRB1**	Leukocyte Immunoglobulin Like Receptor B1	Protein Coding	43	GC19P055408	0.4	https://www.genecards.org/cgi-bin/carddisp.pl?gene=LILRB1
**RAMP1**	Receptor Activity Modifying Protein 1	Protein Coding	43	GC02P237858	0.4	https://www.genecards.org/cgi-bin/carddisp.pl?gene=RAMP1
**TIRAP**	TIR Domain Containing Adaptor Protein	Protein Coding	43	GC11P126284	0.4	https://www.genecards.org/cgi-bin/carddisp.pl?gene=TIRAP
**P2RX1**	Purinergic Receptor P2X 1	Protein Coding	43	GC17M003896	0.4	https://www.genecards.org/cgi-bin/carddisp.pl?gene=P2RX1
**PDYN**	Prodynorphin	Protein Coding	43	GC20M001978	0.4	https://www.genecards.org/cgi-bin/carddisp.pl?gene=PDYN
**ITGA9**	Integrin Subunit Alpha 9	Protein Coding	43	GC03P037468	0.4	https://www.genecards.org/cgi-bin/carddisp.pl?gene=ITGA9

**TIE1**	Tyrosine Kinase With Immunoglobulin Like And EGF Like Domains 1	Protein Coding	43	GC01P043300	0.4	https://www.genecards.org/cgi-bin/carddisp.pl?gene=TIE1
**LPIN2**	Lipin 2	Protein Coding	42	GC18M002906	0.4	https://www.genecards.org/cgi-bin/carddisp.pl?gene=LPIN2
**ALCAM**	Activated Leukocyte Cell Adhesion Molecule	Protein Coding	42	GC03P105366	0.4	https://www.genecards.org/cgi-bin/carddisp.pl?gene=ALCAM
**GRPR**	Gastrin Releasing Peptide Receptor	Protein Coding	42	GC0XP016141	0.4	https://www.genecards.org/cgi-bin/carddisp.pl?gene=GRPR
**CDKN3**	Cyclin Dependent Kinase Inhibitor 3	Protein Coding	42	GC14P054398	0.4	https://www.genecards.org/cgi-bin/carddisp.pl?gene=CDKN3
**CDO1**	Cysteine Dioxygenase Type 1	Protein Coding	42	GC05M115804	0.4	https://www.genecards.org/cgi-bin/carddisp.pl?gene=CDO1
**CHGA**	Chromogranin A	Protein Coding	42	GC14P092923	0.4	https://www.genecards.org/cgi-bin/carddisp.pl?gene=CHGA
**CDH13**	Cadherin 13	Protein Coding	42	GC16P082626	0.4	https://www.genecards.org/cgi-bin/carddisp.pl?gene=CDH13

**PADI3**	Peptidyl Arginine Deiminase 3	Protein Coding	42	GC01P017249	0.4	https://www.genecards.org/cgi-bin/carddisp.pl?gene=PADI3
**IFI16**	Interferon Gamma Inducible Protein 16	Protein Coding	42	GC01P158969	0.4	https://www.genecards.org/cgi-bin/carddisp.pl?gene=IFI16
**PRMT5**	Protein Arginine Methyltransferase 5	Protein Coding	42	GC14M022920	0.4	https://www.genecards.org/cgi-bin/carddisp.pl?gene=PRMT5
**TNFRSF18**	TNF Receptor Superfamily Member 18	Protein Coding	42	GC01M001203	0.4	https://www.genecards.org/cgi-bin/carddisp.pl?gene=TNFRSF18
**TREX1**	Three Prime Repair Exonuclease 1	Protein Coding	42	GC03P048466	0.4	https://www.genecards.org/cgi-bin/carddisp.pl?gene=TREX1
**KDM6B**	Lysine Demethylase 6B	Protein Coding	42	GC17P007834	0.4	https://www.genecards.org/cgi-bin/carddisp.pl?gene=KDM6B
**RAG2**	Recombination Activating 2	Protein Coding	42	GC11M036575	0.4	https://www.genecards.org/cgi-bin/carddisp.pl?gene=RAG2
**LTB4R2**	Leukotriene B4 Receptor 2	Protein Coding	41	GC14P025298	0.4	https://www.genecards.org/cgi-bin/carddisp.pl?gene=LTB4R2

**ATG12**	Autophagy Related 12	Protein Coding	41	GC05M115828	0.4	https://www.genecards.org/cgi-bin/carddisp.pl?gene=ATG12
**LTBR**	Lymphotoxin Beta Receptor	Protein Coding	41	GC12P006375	0.4	https://www.genecards.org/cgi-bin/carddisp.pl?gene=LTBR
**BCL3**	BCL3 Transcription Coactivator	Protein Coding	41	GC19P044747	0.4	https://www.genecards.org/cgi-bin/carddisp.pl?gene=BCL3
**CTHRC1**	Collagen Triple Helix Repeat Containing 1	Protein Coding	41	GC08P103371	0.4	https://www.genecards.org/cgi-bin/carddisp.pl?gene=CTHRC1
**CAMP**	Cathelicidin Antimicrobial Peptide	Protein Coding	41	GC03P048266	0.4	https://www.genecards.org/cgi-bin/carddisp.pl?gene=CAMP
**CSGALNACT2**	Chondroitin Sulfate N-Acetylgalactosaminyltransferase 2	Protein Coding	41	GC10P043138	0.4	https://www.genecards.org/cgi-bin/carddisp.pl?gene=CSGALNACT2
**IL15RA**	Interleukin 15 Receptor Subunit Alpha	Protein Coding	41	GC10M005943	0.4	https://www.genecards.org/cgi-bin/carddisp.pl?gene=IL15RA
**SETDB1**	SET Domain Bifurcated Histone Lysine Methyltransferase 1	Protein Coding	41	GC01P150926	0.4	https://www.genecards.org/cgi-bin/carddisp.pl?gene=SETDB1

**PCM1**	Pericentriolar Material 1	Protein Coding	41	GC08P017922	0.4	https://www.genecards.org/cgi-bin/carddisp.pl?gene=PCM1
**SEMA3F**	Semaphorin 3F	Protein Coding	41	GC03P050167	0.4	https://www.genecards.org/cgi-bin/carddisp.pl?gene=SEMA3F
**TAB3**	TGF-Beta Activated Kinase 1 (MAP3K7) Binding Protein 3	Protein Coding	41	GC0XM030845	0.4	https://www.genecards.org/cgi-bin/carddisp.pl?gene=TAB3
**KLF15**	Kruppel Like Factor 15	Protein Coding	41	GC03M126293	0.4	https://www.genecards.org/cgi-bin/carddisp.pl?gene=KLF15
**ID3**	Inhibitor Of DNA Binding 3, HLH Protein	Protein Coding	41	GC01M023557	0.4	https://www.genecards.org/cgi-bin/carddisp.pl?gene=ID3
**CCR9**	C-C Motif Chemokine Receptor 9	Protein Coding	40	GC03P045903	0.4	https://www.genecards.org/cgi-bin/carddisp.pl?gene=CCR9
**G3BP1**	G3BP Stress Granule Assembly Factor 1	Protein Coding	40	GC05P151771	0.4	https://www.genecards.org/cgi-bin/carddisp.pl?gene=G3BP1
**CAPZA1**	Capping Actin Protein Of Muscle Z-Line Subunit Alpha 1	Protein Coding	40	GC01P112619	0.4	https://www.genecards.org/cgi-bin/carddisp.pl?gene=CAPZA1

**CHRDL1**	Chordin Like 1	Protein Coding	40	GC0XM110674	0.4	https://www.genecards.org/cgi-bin/carddisp.pl?gene=CHRDL1
**EDIL3**	EGF Like Repeats And Discoidin Domains 3	Protein Coding	40	GC05M083940	0.4	https://www.genecards.org/cgi-bin/carddisp.pl?gene=EDIL3
**SLC9A2**	Solute Carrier Family 9 Member A2	Protein Coding	40	GC02P102694	0.4	https://www.genecards.org/cgi-bin/carddisp.pl?gene=SLC9A2
**TRAIP**	TRAF Interacting Protein	Protein Coding	40	GC03M049960	0.4	https://www.genecards.org/cgi-bin/carddisp.pl?gene=TRAIP
**TPST1**	Tyrosylprotein Sulfotransferase 1	Protein Coding	40	GC07P066206	0.4	https://www.genecards.org/cgi-bin/carddisp.pl?gene=TPST1
**TRIM2**	Tripartite Motif Containing 2	Protein Coding	40	GC04P153152	0.4	https://www.genecards.org/cgi-bin/carddisp.pl?gene=TRIM2
**KIR3DL1**	Killer Cell Immunoglobulin Like Receptor, Three Ig Domains And Long Cytoplasmic Tail 1	Protein Coding	40	GC19P055442	0.4	https://www.genecards.org/cgi-bin/carddisp.pl?gene=KIR3DL1
**TEP1**	Telomerase Associated Protein 1	Protein Coding	40	GC14M020365	0.4	https://www.genecards.org/cgi-bin/carddisp.pl?gene=TEP1

**TERF2**	Telomeric Repeat Binding Factor 2	Protein Coding	40	GC16M069355	0.4	https://www.genecards.org/cgi-bin/carddisp.pl?gene=TERF2
**RCOR1**	REST Corepressor 1	Protein Coding	40	GC14P102592	0.4	https://www.genecards.org/cgi-bin/carddisp.pl?gene=RCOR1
**OXT**	Oxytocin/Neurophysin I Prepropeptide	Protein Coding	40	GC20P003068	0.4	https://www.genecards.org/cgi-bin/carddisp.pl?gene=OXT
**SULF2**	Sulfatase 2	Protein Coding	40	GC20M047656	0.4	https://www.genecards.org/cgi-bin/carddisp.pl?gene=SULF2
**BHLHE41**	Basic Helix-Loop-Helix Family Member E41	Protein Coding	39	GC12M026120	0.4	https://www.genecards.org/cgi-bin/carddisp.pl?gene=BHLHE41
**ADAM28**	ADAM Metallopeptidase Domain 28	Protein Coding	39	GC08P024294	0.4	https://www.genecards.org/cgi-bin/carddisp.pl?gene=ADAM28
**HPX**	Hemopexin	Protein Coding	39	GC11M006435	0.4	https://www.genecards.org/cgi-bin/carddisp.pl?gene=HPX
**CD53**	CD53 Molecule	Protein Coding	39	GC01P110871	0.4	https://www.genecards.org/cgi-bin/carddisp.pl?gene=CD53
**CD5L**	CD5 Molecule Like	Protein Coding	39	GC01M157800	0.4	https://www.genecards.org/cgi-bin/carddisp.pl?gene=CD5L

**MKKS**	McKusick-Kaufman Syndrome	Protein Coding	39	GC20M010412	0.4	https://www.genecards.org/cgi-bin/carddisp.pl?gene=MKKS
**DEFB1**	Defensin Beta 1	Protein Coding	39	GC08M006870	0.4	https://www.genecards.org/cgi-bin/carddisp.pl?gene=DEFB1
**TINAGL1**	Tubulointerstitial Nephritis Antigen Like 1	Protein Coding	39	GC01P031576	0.4	https://www.genecards.org/cgi-bin/carddisp.pl?gene=TINAGL1
**TNFRSF10C**	TNF Receptor Superfamily Member 10c	Protein Coding	39	GC08P023102	0.4	https://www.genecards.org/cgi-bin/carddisp.pl?gene=TNFRSF10C
**TTF2**	Transcription Termination Factor 2	Protein Coding	39	GC01P117060	0.4	https://www.genecards.org/cgi-bin/carddisp.pl?gene=TTF2
**RASGRP4**	RAS Guanyl Releasing Protein 4	Protein Coding	39	GC19M038409	0.4	https://www.genecards.org/cgi-bin/carddisp.pl?gene=RASGRP4
**KIR2DL4**	Killer Cell Immunoglobulin Like Receptor, Two Ig Domains And Long Cytoplasmic Tail 4	Protein Coding	39	GC19P055441	0.4	https://www.genecards.org/cgi-bin/carddisp.pl?gene=KIR2DL4
**KCNIP3**	Potassium Voltage-Gated Channel Interacting Protein 3	Protein Coding	39	GC02P095326	0.4	https://www.genecards.org/cgi-bin/carddisp.pl?gene=KCNIP3

**IVNS1ABP**	Influenza Virus NS1A Binding Protein	Protein Coding	39	GC01M185295	0.4	https://www.genecards.org/cgi-bin/carddisp.pl?gene=IVNS1ABP
**FBLIM1**	Filamin Binding LIM Protein 1	Protein Coding	38	GC01P015756	0.4	https://www.genecards.org/cgi-bin/carddisp.pl?gene=FBLIM1
**DEFA5**	Defensin Alpha 5	Protein Coding	38	GC08M007057	0.4	https://www.genecards.org/cgi-bin/carddisp.pl?gene=DEFA5
**IL17D**	Interleukin 17D	Protein Coding	38	GC13P020702	0.4	https://www.genecards.org/cgi-bin/carddisp.pl?gene=IL17D
**PDIA2**	Protein Disulfide Isomerase Family A Member 2	Protein Coding	38	GC16P001384	0.4	https://www.genecards.org/cgi-bin/carddisp.pl?gene=PDIA2
**IER3**	Immediate Early Response 3	Protein Coding	38	GC06M030743	0.4	https://www.genecards.org/cgi-bin/carddisp.pl?gene=IER3
**NLRP6**	NLR Family Pyrin Domain Containing 6	Protein Coding	38	GC11P000269	0.4	https://www.genecards.org/cgi-bin/carddisp.pl?gene=NLRP6
**OSCAR**	Osteoclast Associated Ig-Like Receptor	Protein Coding	38	GC19M054094	0.4	https://www.genecards.org/cgi-bin/carddisp.pl?gene=OSCAR

**SRGN**	Serglycin	Protein Coding	38	GC10P069088	0.4	https://www.genecards.org/cgi-bin/carddisp.pl?gene=SRGN
**TNFSF18**	TNF Superfamily Member 18	Protein Coding	38	GC01M173009	0.4	https://www.genecards.org/cgi-bin/carddisp.pl?gene=TNFSF18
**C1D**	C1D Nuclear Receptor Corepressor	Protein Coding	37	GC02M068041	0.4	https://www.genecards.org/cgi-bin/carddisp.pl?gene=C1D
**BAIAP2L1**	BAR/IMD Domain Containing Adaptor Protein 2 Like 1	Protein Coding	37	GC07M098294	0.4	https://www.genecards.org/cgi-bin/carddisp.pl?gene=BAIAP2L1
**ATP6V0E2**	ATPase H+ Transporting V0 Subunit E2	Protein Coding	37	GC07P149873	0.4	https://www.genecards.org/cgi-bin/carddisp.pl?gene=ATP6V0E2
**ADPRH**	ADP-Ribosylarginine Hydrolase	Protein Coding	37	GC03P119579	0.4	https://www.genecards.org/cgi-bin/carddisp.pl?gene=ADPRH
**ADGRE2**	Adhesion G Protein-Coupled Receptor E2	Protein Coding	37	GC19M014733	0.4	https://www.genecards.org/cgi-bin/carddisp.pl?gene=ADGRE2
**CLEC4E**	C-Type Lectin Domain Family 4 Member E	Protein Coding	37	GC12M008535	0.4	https://www.genecards.org/cgi-bin/carddisp.pl?gene=CLEC4E

**DEFA6**	Defensin Alpha 6	Protein Coding	37	GC08M006924	0.4	https://www.genecards.org/cgi-bin/carddisp.pl?gene=DEFA6
**PRTG**	Protogenin	Protein Coding	37	GC15M055611	0.4	https://www.genecards.org/cgi-bin/carddisp.pl?gene=PRTG
**SAP30BP**	SAP30 Binding Protein	Protein Coding	37	GC17P075667	0.4	https://www.genecards.org/cgi-bin/carddisp.pl?gene=SAP30BP
**PIWIL2**	Piwi Like RNA-Mediated Gene Silencing 2	Protein Coding	37	GC08P022275	0.4	https://www.genecards.org/cgi-bin/carddisp.pl?gene=PIWIL2
**PIWIL4**	Piwi Like RNA-Mediated Gene Silencing 4	Protein Coding	37	GC11P094543	0.4	https://www.genecards.org/cgi-bin/carddisp.pl?gene=PIWIL4
**UCN3**	Urocortin 3	Protein Coding	37	GC10P005396	0.4	https://www.genecards.org/cgi-bin/carddisp.pl?gene=UCN3
**KIR3DL2**	Killer Cell Immunoglobulin Like Receptor, Three Ig Domains And Long Cytoplasmic Tail 2	Protein Coding	37	GC19P055444	0.4	https://www.genecards.org/cgi-bin/carddisp.pl?gene=KIR3DL2
**SPA17**	Sperm Autoantigenic Protein 17	Protein Coding	37	GC11P124673	0.4	https://www.genecards.org/cgi-bin/carddisp.pl?gene=SPA17

**MYCL**	MYCL Proto-Oncogene, BHLH Transcription Factor	Protein Coding	37	GC01M039895	0.4	https://www.genecards.org/cgi-bin/carddisp.pl?gene=MYCL
**TRIB1**	Tribbles Pseudokinase 1	Protein Coding	37	GC08P125430	0.4	https://www.genecards.org/cgi-bin/carddisp.pl?gene=TRIB1
**TRIB2**	Tribbles Pseudokinase 2	Protein Coding	37	GC02P012706	0.4	https://www.genecards.org/cgi-bin/carddisp.pl?gene=TRIB2
**TRIM38**	Tripartite Motif Containing 38	Protein Coding	37	GC06P025962	0.4	https://www.genecards.org/cgi-bin/carddisp.pl?gene=TRIM38
**XCL1**	X-C Motif Chemokine Ligand 1	Protein Coding	37	GC01P168576	0.4	https://www.genecards.org/cgi-bin/carddisp.pl?gene=XCL1
**VASH1**	Vasohibin 1	Protein Coding	37	GC14P076761	0.4	https://www.genecards.org/cgi-bin/carddisp.pl?gene=VASH1
**MKX**	Mohawk Homeobox	Protein Coding	36	GC10M027682	0.4	https://www.genecards.org/cgi-bin/carddisp.pl?gene=MKX
**DEFA3**	Defensin Alpha 3	Protein Coding	36	GC08M007015	0.4	https://www.genecards.org/cgi-bin/carddisp.pl?gene=DEFA3

**SNX19**	Sorting Nexin 19	Protein Coding	36	GC11M130868	0.4	https://www.genecards.org/cgi-bin/carddisp.pl?gene=SNX19
**KIR2DL1**	Killer Cell Immunoglobulin Like Receptor, Two Ig Domains And Long Cytoplasmic Tail 1	Protein Coding	36	GC19P055437	0.4	https://www.genecards.org/cgi-bin/carddisp.pl?gene=KIR2DL1
**KIR2DL3**	Killer Cell Immunoglobulin Like Receptor, Two Ig Domains And Long Cytoplasmic Tail 3	Protein Coding	36	GC19P055469	0.4	https://www.genecards.org/cgi-bin/carddisp.pl?gene=KIR2DL3
**KIR3DL3**	Killer Cell Immunoglobulin Like Receptor, Three Ig Domains And Long Cytoplasmic Tail 3	Protein Coding	36	GC19P055470	0.4	https://www.genecards.org/cgi-bin/carddisp.pl?gene=KIR3DL3
**PGPEP1**	Pyroglutamyl-Peptidase I	Protein Coding	36	GC19P023328	0.4	https://www.genecards.org/cgi-bin/carddisp.pl?gene=PGPEP1
**ZNF277**	Zinc Finger Protein 277	Protein Coding	36	GC07P112206	0.4	https://www.genecards.org/cgi-bin/carddisp.pl?gene=ZNF277
**GALNT15**	Polypeptide N-Acetylgalactosaminyltransferase 15	Protein Coding	35	GC03P016174	0.4	https://www.genecards.org/cgi-bin/carddisp.pl?gene=GALNT15

**GCNT4**	Glucosaminyl (N-Acetyl) Transferase 4	Protein Coding	35	GC05M075025	0.4	https://www.genecards.org/cgi-bin/carddisp.pl?gene=GCNT4
**LVRN**	Laeverin	Protein Coding	35	GC05P115962	0.4	https://www.genecards.org/cgi-bin/carddisp.pl?gene=LVRN
**HECTD2**	HECT Domain E3 Ubiquitin Protein Ligase 2	Protein Coding	35	GC10P091409	0.4	https://www.genecards.org/cgi-bin/carddisp.pl?gene=HECTD2
**PCOLCE2**	Procollagen C-Endopeptidase Enhancer 2	Protein Coding	35	GC03M142815	0.4	https://www.genecards.org/cgi-bin/carddisp.pl?gene=PCOLCE2
**TTC38**	Tetratricopeptide Repeat Domain 38	Protein Coding	35	GC22P046267	0.4	https://www.genecards.org/cgi-bin/carddisp.pl?gene=TTC38
**PTPA**	Protein Phosphatase 2 Phosphatase Activator	Protein Coding	35	GC09P129111	0.4	https://www.genecards.org/cgi-bin/carddisp.pl?gene=PTPA
**LBH**	LBH Regulator Of WNT Signaling Pathway	Protein Coding	35	GC02P030231	0.4	https://www.genecards.org/cgi-bin/carddisp.pl?gene=LBH
**EFS**	Embryonal Fyn-Associated Substrate	Protein Coding	35	GC14M023356	0.4	https://www.genecards.org/cgi-bin/carddisp.pl?gene=EFS
**CLEC5A**	C-Type Lectin Domain Containing 5A	Protein Coding	34	GC07M141927	0.4	https://www.genecards.org/cgi-bin/carddisp.pl?gene=CLEC5A

**ADM2**	Adrenomedullin 2	Protein Coding	33	GC22P050481	0.4	https://www.genecards.org/cgi-bin/carddisp.pl?gene=ADM2
**FNDC1**	Fibronectin Type III Domain Containing 1	Protein Coding	33	GC06P159379	0.4	https://www.genecards.org/cgi-bin/carddisp.pl?gene=FNDC1
**FAM122B**	Family With Sequence Similarity 122B	Protein Coding	33	GC0XM134770	0.4	https://www.genecards.org/cgi-bin/carddisp.pl?gene=FAM122B
**UCN2**	Urocortin 2	Protein Coding	33	GC03M048561	0.4	https://www.genecards.org/cgi-bin/carddisp.pl?gene=UCN2
**ZNF302**	Zinc Finger Protein 302	Protein Coding	33	GC19P034677	0.4	https://www.genecards.org/cgi-bin/carddisp.pl?gene=ZNF302
**H3C1**	H3 Clustered Histone 1	Protein Coding	32	GC06P026040	0.4	https://www.genecards.org/cgi-bin/carddisp.pl?gene=H3C1
**SFMBT2**	Scm Like With Four Mbt Domains 2	Protein Coding	32	GC10M007159	0.4	https://www.genecards.org/cgi-bin/carddisp.pl?gene=SFMBT2
**SAAL1**	Serum Amyloid A Like 1	Protein Coding	32	GC11M018069	0.4	https://www.genecards.org/cgi-bin/carddisp.pl?gene=SAAL1

**CNMD**	Chondromodulin	Protein Coding	29	GC13M052704	0.4	https://www.genecards.org/cgi-bin/carddisp.pl?gene=CNMD
**TERC**	Telomerase RNA Component	RNA Gene	28	GC03M169765	0.4	https://www.genecards.org/cgi-bin/carddisp.pl?gene=TERC
**RBIS**	Ribosomal Biogenesis Factor	Protein Coding	26	GC08M085215	0.4	https://www.genecards.org/cgi-bin/carddisp.pl?gene=RBIS
**KIR2DS2**	Killer Cell Immunoglobulin Like Receptor, Two Ig Domains And Short Cytoplasmic Tail 2	Protein Coding	24	GC19MR00122	0.4	https://www.genecards.org/cgi-bin/carddisp.pl?gene=KIR2DS2
**KIR2DS3**	Killer Cell Immunoglobulin Like Receptor, Two Ig Domains And Short Cytoplasmic Tail 3	Protein Coding	24	GC19MR00031	0.4	https://www.genecards.org/cgi-bin/carddisp.pl?gene=KIR2DS3
**KIR2DL2**	Killer Cell Immunoglobulin Like Receptor, Two Ig Domains And Long Cytoplasmic Tail 2	Protein Coding	23	GC19Mr00108	0.4	https://www.genecards.org/cgi-bin/carddisp.pl?gene=KIR2DL2
**MIR143**	MicroRNA 143	RNA Gene	22	GC05P149410	0.4	https://www.genecards.org/cgi-bin/carddisp.pl?gene=MIR143

**KIR2DL5A**	Killer Cell Immunoglobulin Like Receptor, Two Ig Domains And Long Cytoplasmic Tail 5A	Protein Coding	22	GC19MR00046	0.4	https://www.genecards.org/cgi-bin/carddisp.pl?gene=KIR2DL5A
**KIR2DS1**	Killer Cell Immunoglobulin Like Receptor, Two Ig Domains And Short Cytoplasmic Tail 1	Protein Coding	22	GC19Mr00063	0.4	https://www.genecards.org/cgi-bin/carddisp.pl?gene=KIR2DS1
**MIR124-1**	MicroRNA 124-1	RNA Gene	21	GC08M009903	0.4	https://www.genecards.org/cgi-bin/carddisp.pl?gene=MIR124-1
**KIR3DS1**	Killer Cell Immunoglobulin Like Receptor, Three Ig Domains And Short Cytoplasmic Tail 1	Protein Coding	21	GC19MR00058	0.4	https://www.genecards.org/cgi-bin/carddisp.pl?gene=KIR3DS1
**MIR20A**	MicroRNA 20a	RNA Gene	19	GC13P091434	0.4	https://www.genecards.org/cgi-bin/carddisp.pl?gene=MIR20A
**MIR216A**	MicroRNA 216a	RNA Gene	19	GC02M055988	0.4	https://www.genecards.org/cgi-bin/carddisp.pl?gene=MIR216A
**MCM3AP-AS1**	MCM3AP Antisense RNA 1	RNA Gene	17	GC21P046229	0.4	https://www.genecards.org/cgi-bin/carddisp.pl?gene=MCM3AP-AS1

**MIR19B1**	MicroRNA 19b-1	RNA Gene	17	GC13P091435	0.4	https://www.genecards.org/cgi-bin/carddisp.pl?gene=MIR19B1
**MIR608**	MicroRNA 608	RNA Gene	17	GC10P100974	0.4	https://www.genecards.org/cgi-bin/carddisp.pl?gene=MIR608
**MIR519B**	MicroRNA 519b	RNA Gene	16	GC19P053695	0.4	https://www.genecards.org/cgi-bin/carddisp.pl?gene=MIR519B
**MIR92A2**	MicroRNA 92a-2	RNA Gene	16	GC0XM134206	0.4	https://www.genecards.org/cgi-bin/carddisp.pl?gene=MIR92A2
**MIR382**	MicroRNA 382	RNA Gene	15	GC14P104799	0.4	https://www.genecards.org/cgi-bin/carddisp.pl?gene=MIR382
**MIR558**	MicroRNA 558	RNA Gene	14	GC02P032534	0.4	https://www.genecards.org/cgi-bin/carddisp.pl?gene=MIR558
**MIR602**	MicroRNA 602	RNA Gene	13	GC09P137838	0.4	https://www.genecards.org/cgi-bin/carddisp.pl?gene=MIR602
**KIR2DP1**	Killer Cell Immunoglobulin Like Receptor, Two Ig Domains Pseudogene 1	Pseudogene	9	GC19P055436	0.4	https://www.genecards.org/cgi-bin/carddisp.pl?gene=KIR2DP1

**CDR1-AS**	CDR1 Antisense RNA	RNA Gene	6	GC0XU902169	0.4	https://www.genecards.org/cgi-bin/carddisp.pl?gene=CDR1-AS
**TALDO1P1**	Transaldolase 1 Pseudogene 1	Pseudogene	5	GC01P054525	0.4	https://www.genecards.org/cgi-bin/carddisp.pl?gene=TALDO1P1
**LOC108021846**	SOX9 Promoter Region	Biological Region	1	GC17P072120	0.4	https://www.genecards.org/cgi-bin/carddisp.pl?gene=LOC108021846
**PLA2G6**	Phospholipase A2 Group VI	Protein Coding	47	GC22M046185	0.38	https://www.genecards.org/cgi-bin/carddisp.pl?gene=PLA2G6
**MMP11**	Matrix Metallopeptidase 11	Protein Coding	45	GC22P023768	0.38	https://www.genecards.org/cgi-bin/carddisp.pl?gene=MMP11
**SELENOP**	Selenoprotein P	Protein Coding	31	GC05M042800	0.38	https://www.genecards.org/cgi-bin/carddisp.pl?gene=SELENOP
**PTPN1**	Protein Tyrosine Phosphatase Non-Receptor Type 1	Protein Coding	51	GC20P050510	0.27	https://www.genecards.org/cgi-bin/carddisp.pl?gene=PTPN1
**CASP7**	Caspase 7	Protein Coding	50	GC10P113679	0.27	https://www.genecards.org/cgi-bin/carddisp.pl?gene=CASP7

**CSNK1D**	Casein Kinase 1 Delta	Protein Coding	50	GC17M082239	0.27	https://www.genecards.org/cgi-bin/carddisp.pl?gene=CSNK1D
**IFNGR1**	Interferon Gamma Receptor 1	Protein Coding	50	GC06M137197	0.27	https://www.genecards.org/cgi-bin/carddisp.pl?gene=IFNGR1
**FSHR**	Follicle Stimulating Hormone Receptor	Protein Coding	49	GC02M048953	0.27	https://www.genecards.org/cgi-bin/carddisp.pl?gene=FSHR
**MAPKAPK2**	MAPK Activated Protein Kinase 2	Protein Coding	48	GC01P206684	0.27	https://www.genecards.org/cgi-bin/carddisp.pl?gene=MAPKAPK2
**GPX1**	Glutathione Peroxidase 1	Protein Coding	48	GC03M049368	0.27	https://www.genecards.org/cgi-bin/carddisp.pl?gene=GPX1
**IDE**	Insulin Degrading Enzyme	Protein Coding	48	GC10M092451	0.27	https://www.genecards.org/cgi-bin/carddisp.pl?gene=IDE
**TPO**	Thyroid Peroxidase	Protein Coding	48	GC02P001374	0.27	https://www.genecards.org/cgi-bin/carddisp.pl?gene=TPO
**TRPC3**	Transient Receptor Potential Cation Channel Subfamily C Member 3	Protein Coding	48	GC04M121879	0.27	https://www.genecards.org/cgi-bin/carddisp.pl?gene=TRPC3
**RDX**	Radixin	Protein Coding	48	GC11M109864	0.27	https://www.genecards.org/cgi-bin/carddisp.pl?gene=RDX

**TRPC6**	Transient Receptor Potential Cation Channel Subfamily C Member 6	Protein Coding	48	GC11M101356	0.27	https://www.genecards.org/cgi-bin/carddisp.pl?gene=TRPC6
**GNB3**	G Protein Subunit Beta 3	Protein Coding	47	GC12P006839	0.27	https://www.genecards.org/cgi-bin/carddisp.pl?gene=GNB3
**FGF10**	Fibroblast Growth Factor 10	Protein Coding	47	GC05M044340	0.27	https://www.genecards.org/cgi-bin/carddisp.pl?gene=FGF10
**MC2R**	Melanocortin 2 Receptor	Protein Coding	47	GC18M017331	0.27	https://www.genecards.org/cgi-bin/carddisp.pl?gene=MC2R
**FZD5**	Frizzled Class Receptor 5	Protein Coding	47	GC02M207762	0.27	https://www.genecards.org/cgi-bin/carddisp.pl?gene=FZD5
**MSN**	Moesin	Protein Coding	47	GC0XP065588	0.27	https://www.genecards.org/cgi-bin/carddisp.pl?gene=MSN
**DLG4**	Discs Large MAGUK Scaffold Protein 4	Protein Coding	47	GC17M007189	0.27	https://www.genecards.org/cgi-bin/carddisp.pl?gene=DLG4
**CSNK2B**	Casein Kinase 2 Beta	Protein Coding	47	GC06P047308	0.27	https://www.genecards.org/cgi-bin/carddisp.pl?gene=CSNK2B

**PIK3C2A**	Phosphatidylinositol-4-Phosphate 3-Kinase Catalytic Subunit Type 2 Alpha	Protein Coding	47	GC11M017191	0.27	https://www.genecards.org/cgi-bin/carddisp.pl?gene=PIK3C2A
**ITGB6**	Integrin Subunit Beta 6	Protein Coding	47	GC02M160099	0.27	https://www.genecards.org/cgi-bin/carddisp.pl?gene=ITGB6
**RBPJ**	Recombination Signal Binding Protein For Immunoglobulin Kappa J Region	Protein Coding	47	GC04P026165	0.27	https://www.genecards.org/cgi-bin/carddisp.pl?gene=RBPJ
**AMPD2**	Adenosine Monophosphate Deaminase 2	Protein Coding	46	GC01P109616	0.27	https://www.genecards.org/cgi-bin/carddisp.pl?gene=AMPD2
**DCC**	DCC Netrin 1 Receptor	Protein Coding	46	GC18P052340	0.27	https://www.genecards.org/cgi-bin/carddisp.pl?gene=DCC
**SLC20A2**	Solute Carrier Family 20 Member 2	Protein Coding	46	GC08M042416	0.27	https://www.genecards.org/cgi-bin/carddisp.pl?gene=SLC20A2
**TXNRD2**	Thioredoxin Reductase 2	Protein Coding	46	GC22M019863	0.27	https://www.genecards.org/cgi-bin/carddisp.pl?gene=TXNRD2
**KLK3**	Kallikrein Related Peptidase 3	Protein Coding	46	GC19P050854	0.27	https://www.genecards.org/cgi-bin/carddisp.pl?gene=KLK3

**EZR**	Ezrin	Protein Coding	45	GC06M158765	0.27	https://www.genecards.org/cgi-bin/carddisp.pl?gene=EZR
**ADORA2A**	Adenosine A2a Receptor	Protein Coding	45	GC22P024417	0.27	https://www.genecards.org/cgi-bin/carddisp.pl?gene=ADORA2A
**HRH1**	Histamine Receptor H1	Protein Coding	45	GC03P011113	0.27	https://www.genecards.org/cgi-bin/carddisp.pl?gene=HRH1
**SMURF1**	SMAD Specific E3 Ubiquitin Protein Ligase 1	Protein Coding	45	GC07M099027	0.27	https://www.genecards.org/cgi-bin/carddisp.pl?gene=SMURF1
**OPRL1**	Opioid Related Nociceptin Receptor 1	Protein Coding	45	GC20P064080	0.27	https://www.genecards.org/cgi-bin/carddisp.pl?gene=OPRL1
**ITGA2**	Integrin Subunit Alpha 2	Protein Coding	45	GC05P052989	0.27	https://www.genecards.org/cgi-bin/carddisp.pl?gene=ITGA2
**HSPG2**	Heparan Sulfate Proteoglycan 2	Protein Coding	45	GC01M021822	0.27	https://www.genecards.org/cgi-bin/carddisp.pl?gene=HSPG2
**P2RY2**	Purinergic Receptor P2Y2	Protein Coding	45	GC11P073217	0.27	https://www.genecards.org/cgi-bin/carddisp.pl?gene=P2RY2

**FGF14**	Fibroblast Growth Factor 14	Protein Coding	44	GC13M101710	0.27	https://www.genecards.org/cgi-bin/carddisp.pl?gene=FGF14
**HGS**	Hepatocyte Growth Factor-Regulated Tyrosine Kinase Substrate	Protein Coding	44	GC17P081683	0.27	https://www.genecards.org/cgi-bin/carddisp.pl?gene=HGS
**ENPP2**	Ectonucleotide Pyrophosphatase/Phosphodiesterase 2	Protein Coding	44	GC08M119556	0.27	https://www.genecards.org/cgi-bin/carddisp.pl?gene=ENPP2
**SNCAIP**	Synuclein Alpha Interacting Protein	Protein Coding	44	GC05P122311	0.27	https://www.genecards.org/cgi-bin/carddisp.pl?gene=SNCAIP
**SLC20A1**	Solute Carrier Family 20 Member 1	Protein Coding	44	GC02P115301	0.27	https://www.genecards.org/cgi-bin/carddisp.pl?gene=SLC20A1
**ITGAX**	Integrin Subunit Alpha X	Protein Coding	44	GC16P031570	0.27	https://www.genecards.org/cgi-bin/carddisp.pl?gene=ITGAX
**REST**	RE1 Silencing Transcription Factor	Protein Coding	44	GC04P056907	0.27	https://www.genecards.org/cgi-bin/carddisp.pl?gene=REST
**LBP**	Lipopolysaccharide Binding Protein	Protein Coding	44	GC20P038346	0.27	https://www.genecards.org/cgi-bin/carddisp.pl?gene=LBP
**CBX3**	Chromobox 3	Protein Coding	43	GC07P026201	0.27	https://www.genecards.org/cgi-bin/carddisp.pl?gene=CBX3

**CD70**	CD70 Molecule	Protein Coding	43	GC19M006583	0.27	https://www.genecards.org/cgi-bin/carddisp.pl?gene=CD70
**PAPSS1**	3'-Phosphoadenosine 5'-Phosphosulfate Synthase 1	Protein Coding	43	GC04M107590	0.27	https://www.genecards.org/cgi-bin/carddisp.pl?gene=PAPSS1
**MC5R**	Melanocortin 5 Receptor	Protein Coding	42	GC18P013815	0.27	https://www.genecards.org/cgi-bin/carddisp.pl?gene=MC5R
**COPS5**	COP9 Signalosome Subunit 5	Protein Coding	42	GC08M067043	0.27	https://www.genecards.org/cgi-bin/carddisp.pl?gene=COPS5
**DDAH2**	Dimethylarginine Dimethylaminohydrolase 2	Protein Coding	42	GC06M031727	0.27	https://www.genecards.org/cgi-bin/carddisp.pl?gene=DDAH2
**ITGA11**	Integrin Subunit Alpha 11	Protein Coding	42	GC15M068296	0.27	https://www.genecards.org/cgi-bin/carddisp.pl?gene=ITGA11
**CENPJ**	Centromere Protein J	Protein Coding	42	GC13M024882	0.27	https://www.genecards.org/cgi-bin/carddisp.pl?gene=CENPJ
**MIP**	Major Intrinsic Protein Of Lens Fiber	Protein Coding	41	GC12M056449	0.27	https://www.genecards.org/cgi-bin/carddisp.pl?gene=MIP

**FGF13**	Fibroblast Growth Factor 13	Protein Coding	41	GC0XM138615	0.27	https://www.genecards.org/cgi-bin/carddisp.pl?gene=FGF13
**FOSB**	FosB Proto-Oncogene, AP-1 Transcription Factor Subunit	Protein Coding	41	GC19P045467	0.27	https://www.genecards.org/cgi-bin/carddisp.pl?gene=FOSB
**FOSL2**	FOS Like 2, AP-1 Transcription Factor Subunit	Protein Coding	41	GC02P028392	0.27	https://www.genecards.org/cgi-bin/carddisp.pl?gene=FOSL2
**BRD1**	Bromodomain Containing 1	Protein Coding	41	GC22M049773	0.27	https://www.genecards.org/cgi-bin/carddisp.pl?gene=BRD1
**PTGDR2**	Prostaglandin D2 Receptor 2	Protein Coding	41	GC11M060850	0.27	https://www.genecards.org/cgi-bin/carddisp.pl?gene=PTGDR2
**TRPC5**	Transient Receptor Potential Cation Channel Subfamily C Member 5	Protein Coding	41	GC0XM111774	0.27	https://www.genecards.org/cgi-bin/carddisp.pl?gene=TRPC5
**APOM**	Apolipoprotein M	Protein Coding	40	GC06P047315	0.27	https://www.genecards.org/cgi-bin/carddisp.pl?gene=APOM
**CD276**	CD276 Molecule	Protein Coding	40	GC15P073683	0.27	https://www.genecards.org/cgi-bin/carddisp.pl?gene=CD276

**GABPA**	GA Binding Protein Transcription Factor Subunit Alpha	Protein Coding	39	GC21P025734	0.27	https://www.genecards.org/cgi-bin/carddisp.pl?gene=GABPA
**RHOD**	Ras Homolog Family Member D	Protein Coding	39	GC11P067057	0.27	https://www.genecards.org/cgi-bin/carddisp.pl?gene=RHOD
**SFRP5**	Secreted Frizzled Related Protein 5	Protein Coding	39	GC10M097766	0.27	https://www.genecards.org/cgi-bin/carddisp.pl?gene=SFRP5
**SERPINA7**	Serpin Family A Member 7	Protein Coding	39	GC0XM106032	0.27	https://www.genecards.org/cgi-bin/carddisp.pl?gene=SERPINA7
**KCNN1**	Potassium Calcium-Activated Channel Subfamily N Member 1	Protein Coding	39	GC19P023319	0.27	https://www.genecards.org/cgi-bin/carddisp.pl?gene=KCNN1
**ITGA10**	Integrin Subunit Alpha 10	Protein Coding	39	GC01M145891	0.27	https://www.genecards.org/cgi-bin/carddisp.pl?gene=ITGA10
**ACKR1**	Atypical Chemokine Receptor 1 (Duffy Blood Group)	Protein Coding	37	GC01P159203	0.27	https://www.genecards.org/cgi-bin/carddisp.pl?gene=ACKR1
**MRPL17**	Mitochondrial Ribosomal Protein L17	Protein Coding	37	GC11M006659	0.27	https://www.genecards.org/cgi-bin/carddisp.pl?gene=MRPL17

**DPT**	Dermatopontin	Protein Coding	37	GC01M168664	0.27	https://www.genecards.org/cgi-bin/carddisp.pl?gene=DPT
**NOX5**	NADPH Oxidase 5	Protein Coding	37	GC15P072888	0.27	https://www.genecards.org/cgi-bin/carddisp.pl?gene=NOX5
**P4HTM**	Prolyl 4-Hydroxylase, Transmembrane	Protein Coding	36	GC03P049172	0.27	https://www.genecards.org/cgi-bin/carddisp.pl?gene=P4HTM
**NUPR1**	Nuclear Protein 1, Transcriptional Regulator	Protein Coding	35	GC16M028548	0.27	https://www.genecards.org/cgi-bin/carddisp.pl?gene=NUPR1
**ABHD16A**	Abhydrolase Domain Containing 16A, Phospholipase	Protein Coding	34	GC06M032614	0.27	https://www.genecards.org/cgi-bin/carddisp.pl?gene=ABHD16A
**GPANK1**	G-Patch Domain And Ankyrin Repeats 1	Protein Coding	33	GC06M032611	0.27	https://www.genecards.org/cgi-bin/carddisp.pl?gene=GPANK1
**DEFB104A**	Defensin Beta 104A	Protein Coding	33	GC08P007836	0.27	https://www.genecards.org/cgi-bin/carddisp.pl?gene=DEFB104A
**C6orf47**	Chromosome 6 Open Reading Frame 47	Protein Coding	29	GC06M032610	0.27	https://www.genecards.org/cgi-bin/carddisp.pl?gene=C6orf47

**CGAS**	Cyclic GMP-AMP Synthase	Protein Coding	29	GC06M073414	0.27	https://www.genecards.org/cgi-bin/carddisp.pl?gene=CGAS
**CIP2A**	Cellular Inhibitor Of PP2A	Protein Coding	28	GC03M108545	0.27	https://www.genecards.org/cgi-bin/carddisp.pl?gene=CIP2A
**DEFB104B**	Defensin Beta 104B	Protein Coding	28	GC08M007470	0.27	https://www.genecards.org/cgi-bin/carddisp.pl?gene=DEFB104B
**LY6G5C**	Lymphocyte Antigen 6 Family Member G5C	Protein Coding	27	GC06M031676	0.27	https://www.genecards.org/cgi-bin/carddisp.pl?gene=LY6G5C
**HOTTIP**	HOXA Distal Transcript Antisense RNA	RNA Gene	22	GC07P027198	0.27	https://www.genecards.org/cgi-bin/carddisp.pl?gene=HOTTIP
**MIR770**	MicroRNA 770	RNA Gene	16	GC14P104830	0.27	https://www.genecards.org/cgi-bin/carddisp.pl?gene=MIR770
**FRA1H**	Fragile Site, 5-Azacytidine Type, Common, Fra(1)(Q42)	Uncategorized	5	GC01U990057	0.27	https://www.genecards.org/cgi-bin/carddisp.pl?gene=FRA1H
**FADD**	Fas Associated Via Death Domain	Protein Coding	47	GC11P070203	0.26	https://www.genecards.org/cgi-bin/carddisp.pl?gene=FADD

**KITLG**	KIT Ligand	Protein Coding	44	GC12M088492	0.26	https://www.genecards.org/cgi-bin/carddisp.pl?gene=KITLG
**ERRFI1**	ERBB Receptor Feedback Inhibitor 1	Protein Coding	37	GC01M008004	0.26	https://www.genecards.org/cgi-bin/carddisp.pl?gene=ERRFI1
**CYP2C9**	Cytochrome P450 Family 2 Subfamily C Member 9	Protein Coding	48	GC10P094938	0.23	https://www.genecards.org/cgi-bin/carddisp.pl?gene=CYP2C9
**HNRNPA2B1**	Heterogeneous Nuclear Ribonucleoprotein A2/B1	Protein Coding	45	GC07M026174	0.23	https://www.genecards.org/cgi-bin/carddisp.pl?gene=HNRNPA2B1
**CALCRL**	Calcitonin Receptor Like Receptor	Protein Coding	43	GC02M187341	0.23	https://www.genecards.org/cgi-bin/carddisp.pl?gene=CALCRL
**USP3**	Ubiquitin Specific Peptidase 3	Protein Coding	39	GC15P063504	0.23	https://www.genecards.org/cgi-bin/carddisp.pl?gene=USP3
**ELP1**	Elongator Complex Protein 1	Protein Coding	33	GC09M108868	0.23	https://www.genecards.org/cgi-bin/carddisp.pl?gene=ELP1
**MAP3K4-AS1**	MAP3K4 Antisense RNA 1	RNA Gene	2	GC06U903870	0.23	https://www.genecards.org/cgi-bin/carddisp.pl?gene=MAP3K4-AS1

**MAP2K2**	Mitogen-Activated Protein Kinase Kinase 2	Protein Coding	53	GC19M004090	0.19	https://www.genecards.org/cgi-bin/carddisp.pl?gene=MAP2K2
**NR1H2**	Nuclear Receptor Subfamily 1 Group H Member 2	Protein Coding	48	GC19P050329	0.19	https://www.genecards.org/cgi-bin/carddisp.pl?gene=NR1H2
**SQSTM1**	Sequestosome 1	Protein Coding	48	GC05P179806	0.19	https://www.genecards.org/cgi-bin/carddisp.pl?gene=SQSTM1
**KRT18**	Keratin 18	Protein Coding	48	GC12P052948	0.19	https://www.genecards.org/cgi-bin/carddisp.pl?gene=KRT18
**PRKAB1**	Protein Kinase AMP-Activated Non-Catalytic Subunit Beta 1	Protein Coding	47	GC12P119632	0.19	https://www.genecards.org/cgi-bin/carddisp.pl?gene=PRKAB1
**PTGIS**	Prostaglandin I2 Synthase	Protein Coding	47	GC20M049503	0.19	https://www.genecards.org/cgi-bin/carddisp.pl?gene=PTGIS
**TXNRD1**	Thioredoxin Reductase 1	Protein Coding	47	GC12P104215	0.19	https://www.genecards.org/cgi-bin/carddisp.pl?gene=TXNRD1
**LIFR**	LIF Receptor Subunit Alpha	Protein Coding	47	GC05M038475	0.19	https://www.genecards.org/cgi-bin/carddisp.pl?gene=LIFR

**GNAI1**	G Protein Subunit Alpha I1	Protein Coding	46	GC07P079 769	0.19	https://www.genecards.org/cgi-bin/carddisp.pl?gene=GNAI1
**F13A1**	Coagulation Factor XIII A Chain	Protein Coding	45	GC06M006 144	0.19	https://www.genecards.org/cgi-bin/carddisp.pl?gene=F13A1
**A2M**	Alpha-2-Macroglobulin	Protein Coding	45	GC12M009 067	0.19	https://www.genecards.org/cgi-bin/carddisp.pl?gene=A2M
**CRHR1**	Corticotropin Releasing Hormone Receptor 1	Protein Coding	45	GC17P045 784	0.19	https://www.genecards.org/cgi-bin/carddisp.pl?gene=CRHR1
**TBX5**	T-Box Transcription Factor 5	Protein Coding	45	GC12M114 353	0.19	https://www.genecards.org/cgi-bin/carddisp.pl?gene=TBX5
**EIF2S1**	Eukaryotic Translation Initiation Factor 2 Subunit Alpha	Protein Coding	44	GC14P067 359	0.19	https://www.genecards.org/cgi-bin/carddisp.pl?gene=EIF2S1
**SNAI2**	Snail Family Transcriptional Repressor 2	Protein Coding	44	GC08M048 854	0.19	https://www.genecards.org/cgi-bin/carddisp.pl?gene=SNAI2
**ALPI**	Alkaline Phosphatase, Intestinal	Protein Coding	43	GC02P232 456	0.19	https://www.genecards.org/cgi-bin/carddisp.pl?gene=ALPI

**SERPINA6**	Serpin Family A Member 6	Protein Coding	43	GC14M094341	0.19	https://www.genecards.org/cgi-bin/carddisp.pl?gene=SERPINA6
**CNTF**	Ciliary Neurotrophic Factor	Protein Coding	41	GC11P058622	0.19	https://www.genecards.org/cgi-bin/carddisp.pl?gene=CNTF
**IL21R**	Interleukin 21 Receptor	Protein Coding	41	GC16P027413	0.19	https://www.genecards.org/cgi-bin/carddisp.pl?gene=IL21R
**DIO1**	Iodothyronine Deiodinase 1	Protein Coding	40	GC01P053891	0.19	https://www.genecards.org/cgi-bin/carddisp.pl?gene=DIO1
**GAST**	Gastrin	Protein Coding	40	GC17P041712	0.19	https://www.genecards.org/cgi-bin/carddisp.pl?gene=GAST
**MIR203A**	MicroRNA 203a	RNA Gene	19	GC14P104655	0.19	https://www.genecards.org/cgi-bin/carddisp.pl?gene=MIR203A
**AXL**	AXL Receptor Tyrosine Kinase	Protein Coding	51	GC19P041219	0.13	https://www.genecards.org/cgi-bin/carddisp.pl?gene=AXL
**ATR**	ATR Serine/Threonine Kinase	Protein Coding	51	GC03M142449	0.13	https://www.genecards.org/cgi-bin/carddisp.pl?gene=ATR
**CDC42**	Cell Division Cycle 42	Protein Coding	51	GC01P022057	0.13	https://www.genecards.org/cgi-bin/carddisp.pl?gene=CDC42

**NTRK3**	Neurotrophic Receptor Tyrosine Kinase 3	Protein Coding	51	GC15M087859	0.13	https://www.genecards.org/cgi-bin/carddisp.pl?gene=NTRK3
**JAK3**	Janus Kinase 3	Protein Coding	51	GC19M017824	0.13	https://www.genecards.org/cgi-bin/carddisp.pl?gene=JAK3
**PGR**	Progesterone Receptor	Protein Coding	50	GC11M100943	0.13	https://www.genecards.org/cgi-bin/carddisp.pl?gene=PGR
**IRAK1**	Interleukin 1 Receptor Associated Kinase 1	Protein Coding	50	GC0XM154010	0.13	https://www.genecards.org/cgi-bin/carddisp.pl?gene=IRAK1
**PLK1**	Polo Like Kinase 1	Protein Coding	49	GC16P023888	0.13	https://www.genecards.org/cgi-bin/carddisp.pl?gene=PLK1
**CASP10**	Caspase 10	Protein Coding	48	GC02P201182	0.13	https://www.genecards.org/cgi-bin/carddisp.pl?gene=CASP10
**MAPK13**	Mitogen-Activated Protein Kinase 13	Protein Coding	48	GC06P047452	0.13	https://www.genecards.org/cgi-bin/carddisp.pl?gene=MAPK13
**AXIN2**	Axin 2	Protein Coding	48	GC17M065528	0.13	https://www.genecards.org/cgi-bin/carddisp.pl?gene=AXIN2

**MEF2C**	Myocyte Enhancer Factor 2C	Protein Coding	48	GC05M088718	0.13	https://www.genecards.org/cgi-bin/carddisp.pl?gene=MEF2C
**RHEB**	Ras Homolog, MTORC1 Binding	Protein Coding	48	GC07M151466	0.13	https://www.genecards.org/cgi-bin/carddisp.pl?gene=RHEB
**IL2RG**	Interleukin 2 Receptor Subunit Gamma	Protein Coding	48	GC0XM071108	0.13	https://www.genecards.org/cgi-bin/carddisp.pl?gene=IL2RG
**PRMT1**	Protein Arginine Methyltransferase 1	Protein Coding	48	GC19P049675	0.13	https://www.genecards.org/cgi-bin/carddisp.pl?gene=PRMT1
**EGLN1**	Egl-9 Family Hypoxia Inducible Factor 1	Protein Coding	48	GC01M231363	0.13	https://www.genecards.org/cgi-bin/carddisp.pl?gene=EGLN1
**PLD1**	Phospholipase D1	Protein Coding	48	GC03M171600	0.13	https://www.genecards.org/cgi-bin/carddisp.pl?gene=PLD1
**IRAK4**	Interleukin 1 Receptor Associated Kinase 4	Protein Coding	48	GC12P043758	0.13	https://www.genecards.org/cgi-bin/carddisp.pl?gene=IRAK4
**VCP**	Valosin Containing Protein	Protein Coding	48	GC09M035056	0.13	https://www.genecards.org/cgi-bin/carddisp.pl?gene=VCP

**YWHAZ**	Tyrosine 3-Monooxygenase/Tryptophan 5-Monooxygenase Activation Protein Zeta	Protein Coding	48	GC08M100917	0.13	https://www.genecards.org/cgi-bin/carddisp.pl?gene=YWHAZ
**GLI1**	GLI Family Zinc Finger 1	Protein Coding	47	GC12P057460	0.13	https://www.genecards.org/cgi-bin/carddisp.pl?gene=GLI1
**DDR1**	Discoidin Domain Receptor Tyrosine Kinase 1	Protein Coding	47	GC06P047288	0.13	https://www.genecards.org/cgi-bin/carddisp.pl?gene=DDR1
**IFIH1**	Interferon Induced With Helicase C Domain 1	Protein Coding	47	GC02M162267	0.13	https://www.genecards.org/cgi-bin/carddisp.pl?gene=IFIH1
**PITX2**	Paired Like Homeodomain 2	Protein Coding	47	GC04M110617	0.13	https://www.genecards.org/cgi-bin/carddisp.pl?gene=PITX2
**PNLIP**	Pancreatic Lipase	Protein Coding	47	GC10P116545	0.13	https://www.genecards.org/cgi-bin/carddisp.pl?gene=PNLIP
**ODC1**	Ornithine Decarboxylase 1	Protein Coding	47	GC02M010432	0.13	https://www.genecards.org/cgi-bin/carddisp.pl?gene=ODC1
**PTGIR**	Prostaglandin I2 Receptor	Protein Coding	47	GC19M046611	0.13	https://www.genecards.org/cgi-bin/carddisp.pl?gene=PTGIR

**PIN1**	Peptidylprolyl Cis/Trans Isomerase, NIMA-Interacting 1	Protein Coding	47	GC19P009835	0.13	https://www.genecards.org/cgi-bin/carddisp.pl?gene=PIN1
**IRF3**	Interferon Regulatory Factor 3	Protein Coding	47	GC19M049659	0.13	https://www.genecards.org/cgi-bin/carddisp.pl?gene=IRF3
**PTK2**	Protein Tyrosine Kinase 2	Protein Coding	47	GC08M140657	0.13	https://www.genecards.org/cgi-bin/carddisp.pl?gene=PTK2
**ATF4**	Activating Transcription Factor 4	Protein Coding	46	GC22P039525	0.13	https://www.genecards.org/cgi-bin/carddisp.pl?gene=ATF4
**CLTC**	Clathrin Heavy Chain	Protein Coding	46	GC17P059619	0.13	https://www.genecards.org/cgi-bin/carddisp.pl?gene=CLTC
**NR4A1**	Nuclear Receptor Subfamily 4 Group A Member 1	Protein Coding	46	GC12P052022	0.13	https://www.genecards.org/cgi-bin/carddisp.pl?gene=NR4A1
**SDHA**	Succinate Dehydrogenase Complex Flavoprotein Subunit A	Protein Coding	46	GC05P000208	0.13	https://www.genecards.org/cgi-bin/carddisp.pl?gene=SDHA
**KDM6A**	Lysine Demethylase 6A	Protein Coding	46	GC0XP044873	0.13	https://www.genecards.org/cgi-bin/carddisp.pl?gene=KDM6A

**TLR7**	Toll Like Receptor 7	Protein Coding	46	GC0XP012867	0.13	https://www.genecards.org/cgi-bin/carddisp.pl?gene=TLR7
**PDE8B**	Phosphodiesterase 8B	Protein Coding	46	GC05P077180	0.13	https://www.genecards.org/cgi-bin/carddisp.pl?gene=PDE8B
**WEE1**	WEE1 G2 Checkpoint Kinase	Protein Coding	46	GC11P009573	0.13	https://www.genecards.org/cgi-bin/carddisp.pl?gene=WEE1
**CSF2RA**	Colony Stimulating Factor 2 Receptor Subunit Alpha	Protein Coding	45	GC0XP001270	0.13	https://www.genecards.org/cgi-bin/carddisp.pl?gene=CSF2RA
**S1PR2**	Sphingosine-1-Phosphate Receptor 2	Protein Coding	45	GC19M010223	0.13	https://www.genecards.org/cgi-bin/carddisp.pl?gene=S1PR2
**SMAD9**	SMAD Family Member 9	Protein Coding	45	GC13M036844	0.13	https://www.genecards.org/cgi-bin/carddisp.pl?gene=SMAD9
**TNFSF15**	TNF Superfamily Member 15	Protein Coding	45	GC09M114784	0.13	https://www.genecards.org/cgi-bin/carddisp.pl?gene=TNFSF15
**RASGRP2**	RAS Guanyl Releasing Protein 2	Protein Coding	45	GC11M064726	0.13	https://www.genecards.org/cgi-bin/carddisp.pl?gene=RASGRP2

**EIF2AK2**	Eukaryotic Translation Initiation Factor 2 Alpha Kinase 2	Protein Coding	45	GC02M037099	0.13	https://www.genecards.org/cgi-bin/carddisp.pl?gene=EIF2AK2
**TGFBI**	Transforming Growth Factor Beta Induced	Protein Coding	45	GC05P136027	0.13	https://www.genecards.org/cgi-bin/carddisp.pl?gene=TGFBI
**GP6**	Glycoprotein VI Platelet	Protein Coding	44	GC19M055013	0.13	https://www.genecards.org/cgi-bin/carddisp.pl?gene=GP6
**MAP3K14**	Mitogen-Activated Protein Kinase Kinase Kinase 14	Protein Coding	44	GC17M045263	0.13	https://www.genecards.org/cgi-bin/carddisp.pl?gene=MAP3K14
**CLEC7A**	C-Type Lectin Domain Containing 7A	Protein Coding	44	GC12M013863	0.13	https://www.genecards.org/cgi-bin/carddisp.pl?gene=CLEC7A
**CBFB**	Core-Binding Factor Subunit Beta	Protein Coding	44	GC16P067063	0.13	https://www.genecards.org/cgi-bin/carddisp.pl?gene=CBFB
**CISH**	Cytokine Inducible SH2 Containing Protein	Protein Coding	44	GC03M050618	0.13	https://www.genecards.org/cgi-bin/carddisp.pl?gene=CISH
**GAS6**	Growth Arrest Specific 6	Protein Coding	44	GC13M113820	0.13	https://www.genecards.org/cgi-bin/carddisp.pl?gene=GAS6

**IL10RB**	Interleukin 10 Receptor Subunit Beta	Protein Coding	44	GC21P033266	0.13	https://www.genecards.org/cgi-bin/carddisp.pl?gene=IL10RB
**NTF4**	Neurotrophin 4	Protein Coding	44	GC19M049073	0.13	https://www.genecards.org/cgi-bin/carddisp.pl?gene=NTF4
**NPPB**	Natriuretic Peptide B	Protein Coding	44	GC01M011858	0.13	https://www.genecards.org/cgi-bin/carddisp.pl?gene=NPPB
**UGT2B7**	UDP Glucuronosyltransferase Family 2 Member B7	Protein Coding	44	GC04P069051	0.13	https://www.genecards.org/cgi-bin/carddisp.pl?gene=UGT2B7
**SRD5A1**	Steroid 5 Alpha-Reductase 1	Protein Coding	44	GC05P006633	0.13	https://www.genecards.org/cgi-bin/carddisp.pl?gene=SRD5A1
**PDE4B**	Phosphodiesterase 4B	Protein Coding	44	GC01P065792	0.13	https://www.genecards.org/cgi-bin/carddisp.pl?gene=PDE4B
**EIF2AK4**	Eukaryotic Translation Initiation Factor 2 Alpha Kinase 4	Protein Coding	44	GC15P039934	0.13	https://www.genecards.org/cgi-bin/carddisp.pl?gene=EIF2AK4
**DLX5**	Distal-Less Homeobox 5	Protein Coding	44	GC07M097020	0.13	https://www.genecards.org/cgi-bin/carddisp.pl?gene=DLX5

**ABCB7**	ATP Binding Cassette Subfamily B Member 7	Protein Coding	43	GC0XM075053	0.13	https://www.genecards.org/cgi-bin/carddisp.pl?gene=ABCB7
**AREG**	Amphiregulin	Protein Coding	43	GC04P074445	0.13	https://www.genecards.org/cgi-bin/carddisp.pl?gene=AREG
**AMD 1.00**	Adenosylmethionine Decarboxylase 1	Protein Coding	43	GC06P110814	0.13	https://www.genecards.org/cgi-bin/carddisp.pl?gene=AMD1
**AIMP1**	Aminoacyl TRNA Synthetase Complex Interacting Multifunctional Protein 1	Protein Coding	43	GC04P106315	0.13	https://www.genecards.org/cgi-bin/carddisp.pl?gene=AIMP1
**AGRP**	Agouti Related Neuropeptide	Protein Coding	43	GC16M067482	0.13	https://www.genecards.org/cgi-bin/carddisp.pl?gene=AGRP
**MDM4**	MDM4 Regulator Of P53	Protein Coding	43	GC01P204516	0.13	https://www.genecards.org/cgi-bin/carddisp.pl?gene=MDM4
**RGS4**	Regulator Of G Protein Signaling 4	Protein Coding	43	GC01P163038	0.13	https://www.genecards.org/cgi-bin/carddisp.pl?gene=RGS4
**RGS10**	Regulator Of G Protein Signaling 10	Protein Coding	43	GC10M119499	0.13	https://www.genecards.org/cgi-bin/carddisp.pl?gene=RGS10

**COL4A5**	Collagen Type IV Alpha 5 Chain	Protein Coding	43	GC0XP108439	0.13	https://www.genecards.org/cgi-bin/carddisp.pl?gene=COL4A5
**CYSLTR1**	Cysteinyl Leukotriene Receptor 1	Protein Coding	43	GC0XM078271	0.13	https://www.genecards.org/cgi-bin/carddisp.pl?gene=CYSLTR1
**PCSK7**	Proprotein Convertase Subtilisin/Kexin Type 7	Protein Coding	43	GC11M117199	0.13	https://www.genecards.org/cgi-bin/carddisp.pl?gene=PCSK7
**ENPP3**	Ectonucleotide Pyrophosphatase/Phosphodiesterase 3	Protein Coding	43	GC06P131617	0.13	https://www.genecards.org/cgi-bin/carddisp.pl?gene=ENPP3
**PTPRK**	Protein Tyrosine Phosphatase Receptor Type K	Protein Coding	43	GC06M127949	0.13	https://www.genecards.org/cgi-bin/carddisp.pl?gene=PTPRK
**TRADD**	TNFRSF1A Associated Via Death Domain	Protein Coding	43	GC16M067154	0.13	https://www.genecards.org/cgi-bin/carddisp.pl?gene=TRADD
**TRAF3IP2**	TRAF3 Interacting Protein 2	Protein Coding	43	GC06M111555	0.13	https://www.genecards.org/cgi-bin/carddisp.pl?gene=TRAF3IP2
**UGT1A9**	UDP Glucuronosyltransferase Family 1 Member A9	Protein Coding	43	GC02P233671	0.13	https://www.genecards.org/cgi-bin/carddisp.pl?gene=UGT1A9

**TRIM32**	Tripartite Motif Containing 32	Protein Coding	43	GC09P116687	0.13	https://www.genecards.org/cgi-bin/carddisp.pl?gene=TRIM32
**ROM1**	Retinal Outer Segment Membrane Protein 1	Protein Coding	43	GC11P062611	0.13	https://www.genecards.org/cgi-bin/carddisp.pl?gene=ROM1
**HSPA1A**	Heat Shock Protein Family A (Hsp70) Member 1A	Protein Coding	43	GC06P047326	0.13	https://www.genecards.org/cgi-bin/carddisp.pl?gene=HSPA1A
**PLAA**	Phospholipase A2 Activating Protein	Protein Coding	43	GC09M026903	0.13	https://www.genecards.org/cgi-bin/carddisp.pl?gene=PLAA
**ANXA7**	Annexin A7	Protein Coding	42	GC10M073375	0.13	https://www.genecards.org/cgi-bin/carddisp.pl?gene=ANXA7
**BNIP3**	BCL2 Interacting Protein 3	Protein Coding	42	GC10M131966	0.13	https://www.genecards.org/cgi-bin/carddisp.pl?gene=BNIP3
**HNRNPD**	Heterogeneous Nuclear Ribonucleoprotein D	Protein Coding	42	GC04M082352	0.13	https://www.genecards.org/cgi-bin/carddisp.pl?gene=HNRNPD
**SEMA7A**	Semaphorin 7A (John Milton Hagen Blood Group)	Protein Coding	42	GC15M074409	0.13	https://www.genecards.org/cgi-bin/carddisp.pl?gene=SEMA7A

**PYY**	Peptide YY	Protein Coding	42	GC17M043952	0.13	https://www.genecards.org/cgi-bin/carddisp.pl?gene=PYY
**PDIA4**	Protein Disulfide Isomerase Family A Member 4	Protein Coding	42	GC07M149003	0.13	https://www.genecards.org/cgi-bin/carddisp.pl?gene=PDIA4
**GLRA3**	Glycine Receptor Alpha 3	Protein Coding	41	GC04M174636	0.13	https://www.genecards.org/cgi-bin/carddisp.pl?gene=GLRA3
**HNRNPC**	Heterogeneous Nuclear Ribonucleoprotein C	Protein Coding	41	GC14M021210	0.13	https://www.genecards.org/cgi-bin/carddisp.pl?gene=HNRNPC
**H6PD**	Hexose-6-Phosphate Dehydrogenase/Glucose 1-Dehydrogenase	Protein Coding	41	GC01P009234	0.13	https://www.genecards.org/cgi-bin/carddisp.pl?gene=H6PD
**DSE**	Dermatan Sulfate Epimerase	Protein Coding	41	GC06P116255	0.13	https://www.genecards.org/cgi-bin/carddisp.pl?gene=DSE
**RASGRF1**	Ras Protein Specific Guanine Nucleotide Releasing Factor 1	Protein Coding	41	GC15M078959	0.13	https://www.genecards.org/cgi-bin/carddisp.pl?gene=RASGRF1
**LIPF**	Lipase F, Gastric Type	Protein Coding	41	GC10P088664	0.13	https://www.genecards.org/cgi-bin/carddisp.pl?gene=LIPF
**RGS5**	Regulator Of G Protein Signaling 5	Protein Coding	40	GC01M163111	0.13	https://www.genecards.org/cgi-bin/carddisp.pl?gene=RGS5

**RGS7**	Regulator Of G Protein Signaling 7	Protein Coding	40	GC01M240775	0.13	https://www.genecards.org/cgi-bin/carddisp.pl?gene=RGS7
**HCRT**	Hypocretin Neuropeptide Precursor	Protein Coding	40	GC17M042185	0.13	https://www.genecards.org/cgi-bin/carddisp.pl?gene=HCRT
**EHF**	ETS Homologous Factor	Protein Coding	40	GC11P034621	0.13	https://www.genecards.org/cgi-bin/carddisp.pl?gene=EHF
**HSPB2**	Heat Shock Protein Family B (Small) Member 2	Protein Coding	40	GC11P111913	0.13	https://www.genecards.org/cgi-bin/carddisp.pl?gene=HSPB2
**IAPP**	Islet Amyloid Polypeptide	Protein Coding	40	GC12P021354	0.13	https://www.genecards.org/cgi-bin/carddisp.pl?gene=IAPP
**UXS1**	UDP-Glucuronate Decarboxylase 1	Protein Coding	40	GC02M106094	0.13	https://www.genecards.org/cgi-bin/carddisp.pl?gene=UXS1
**LMOD1**	Leiomodin 1	Protein Coding	40	GC01M201896	0.13	https://www.genecards.org/cgi-bin/carddisp.pl?gene=LMOD1
**MAPRE3**	Microtubule Associated Protein RP/EB Family Member 3	Protein Coding	39	GC02P026935	0.13	https://www.genecards.org/cgi-bin/carddisp.pl?gene=MAPRE3

**EBI3**	Epstein-Barr Virus Induced 3	Protein Coding	39	GC19P004232	0.13	https://www.genecards.org/cgi-bin/carddisp.pl?gene=EBI3
**CD83**	CD83 Molecule	Protein Coding	39	GC06P014117	0.13	https://www.genecards.org/cgi-bin/carddisp.pl?gene=CD83
**NELL1**	Neural EGFL Like 1	Protein Coding	39	GC11P020647	0.13	https://www.genecards.org/cgi-bin/carddisp.pl?gene=NELL1
**IL1F10**	Interleukin 1 Family Member 10	Protein Coding	39	GC02P113067	0.13	https://www.genecards.org/cgi-bin/carddisp.pl?gene=IL1F10
**SLC16A4**	Solute Carrier Family 16 Member 4	Protein Coding	39	GC01M110362	0.13	https://www.genecards.org/cgi-bin/carddisp.pl?gene=SLC16A4
**PHOSPHO1**	Phosphoethanolamine/Phosphocholine Phosphatase 1	Protein Coding	39	GC17M049223	0.13	https://www.genecards.org/cgi-bin/carddisp.pl?gene=PHOSPHO1
**LAMP3**	Lysosomal Associated Membrane Protein 3	Protein Coding	39	GC03M183122	0.13	https://www.genecards.org/cgi-bin/carddisp.pl?gene=LAMP3
**TXNDC5**	Thioredoxin Domain Containing 5	Protein Coding	39	GC06M007893	0.13	https://www.genecards.org/cgi-bin/carddisp.pl?gene=TXNDC5

**TSPAN9**	Tetraspanin 9	Protein Coding	39	GC12P003 078	0.13	https://www.genecards.org/cgi-bin/carddisp.pl?gene=TSPAN9
**SPAM1**	Sperm Adhesion Molecule 1	Protein Coding	39	GC07P123 925	0.13	https://www.genecards.org/cgi-bin/carddisp.pl?gene=SPAM1
**ITGAE**	Integrin Subunit Alpha E	Protein Coding	39	GC17M003 722	0.13	https://www.genecards.org/cgi-bin/carddisp.pl?gene=ITGAE
**CCL17**	C-C Motif Chemokine Ligand 17	Protein Coding	38	GC16P057 400	0.13	https://www.genecards.org/cgi-bin/carddisp.pl?gene=CCL17
**AP2A1**	Adaptor Related Protein Complex 2 Subunit Alpha 1	Protein Coding	38	GC19P049 766	0.13	https://www.genecards.org/cgi-bin/carddisp.pl?gene=AP2A1
**ADAMTS16**	ADAM Metallopeptidase With Thrombospondin Type 1 Motif 16	Protein Coding	38	GC05P005 140	0.13	https://www.genecards.org/cgi-bin/carddisp.pl?gene=ADAMTS16
**UGT1A3**	UDP Glucuronosyltransferase Family 1 Member A3	Protein Coding	38	GC02P233 729	0.13	https://www.genecards.org/cgi-bin/carddisp.pl?gene=UGT1A3
**TET1**	Tet Methylcytosine Dioxygenase 1	Protein Coding	38	GC10P068 560	0.13	https://www.genecards.org/cgi-bin/carddisp.pl?gene=TET1

**EBAG9**	Estrogen Receptor Binding Site Associated Antigen 9	Protein Coding	38	GC08P109536	0.13	https://www.genecards.org/cgi-bin/carddisp.pl?gene=EBAG9
**MADCAM1**	Mucosal Vascular Addressin Cell Adhesion Molecule 1	Protein Coding	37	GC19P000499	0.13	https://www.genecards.org/cgi-bin/carddisp.pl?gene=MADCAM1
**CHST4**	Carbohydrate Sulfotransferase 4	Protein Coding	37	GC16P071560	0.13	https://www.genecards.org/cgi-bin/carddisp.pl?gene=CHST4
**PIEZO1**	Piezo Type Mechanosensitive Ion Channel Component 1	Protein Coding	37	GC16M088715	0.13	https://www.genecards.org/cgi-bin/carddisp.pl?gene=PIEZO1
**LAIR2**	Leukocyte Associated Immunoglobulin Like Receptor 2	Protein Coding	37	GC19P055377	0.13	https://www.genecards.org/cgi-bin/carddisp.pl?gene=LAIR2
**IRX1**	Iroquois Homeobox 1	Protein Coding	37	GC05P003596	0.13	https://www.genecards.org/cgi-bin/carddisp.pl?gene=IRX1
**ARL 15.00**	ADP Ribosylation Factor Like GTPase 15	Protein Coding	36	GC05M053883	0.13	https://www.genecards.org/cgi-bin/carddisp.pl?gene=ARL15
**HIVEP3**	HIVEP Zinc Finger 3	Protein Coding	36	GC01M041506	0.13	https://www.genecards.org/cgi-bin/carddisp.pl?gene=HIVEP3

**TNIP3**	TNFAIP3 Interacting Protein 3	Protein Coding	36	GC04M121131	0.13	https://www.genecards.org/cgi-bin/carddisp.pl?gene=TNIP3
**LILRA5**	Leukocyte Immunoglobulin Like Receptor A5	Protein Coding	36	GC19M054307	0.13	https://www.genecards.org/cgi-bin/carddisp.pl?gene=LILRA5
**TP53AIP1**	Tumor Protein P53 Regulated Apoptosis Inducing Protein 1	Protein Coding	36	GC11M128934	0.13	https://www.genecards.org/cgi-bin/carddisp.pl?gene=TP53AIP1
**RNASE7**	Ribonuclease A Family Member 7	Protein Coding	35	GC14P021042	0.13	https://www.genecards.org/cgi-bin/carddisp.pl?gene=RNASE7
**CNN3**	Calponin 3	Protein Coding	35	GC01M094896	0.13	https://www.genecards.org/cgi-bin/carddisp.pl?gene=CNN3
**RNPC3**	RNA Binding Region (RNP1, RRM) Containing 3	Protein Coding	35	GC01P103525	0.13	https://www.genecards.org/cgi-bin/carddisp.pl?gene=RNPC3
**ENGASE**	Endo-Beta-N-Acetylglucosaminidase	Protein Coding	35	GC17P079071	0.13	https://www.genecards.org/cgi-bin/carddisp.pl?gene=ENGASE
**COL20A1**	Collagen Type XX Alpha 1 Chain	Protein Coding	35	GC20P063293	0.13	https://www.genecards.org/cgi-bin/carddisp.pl?gene=COL20A1

**PPARGC1B**	PPARG Coactivator 1 Beta	Protein Coding	35	GC05P149730	0.13	https://www.genecards.org/cgi-bin/carddisp.pl?gene=PPARGC1B
**IL36B**	Interleukin 36 Beta	Protein Coding	35	GC02M113022	0.13	https://www.genecards.org/cgi-bin/carddisp.pl?gene=IL36B
**CSN1S1**	Casein Alpha S1	Protein Coding	34	GC04P069932	0.13	https://www.genecards.org/cgi-bin/carddisp.pl?gene=CSN1S1
**JPH4**	Junctophilin 4	Protein Coding	34	GC14M023568	0.13	https://www.genecards.org/cgi-bin/carddisp.pl?gene=JPH4
**VEGFD**	Vascular Endothelial Growth Factor D	Protein Coding	34	GC0XM015345	0.13	https://www.genecards.org/cgi-bin/carddisp.pl?gene=VEGFD
**GCFC2**	GC-Rich Sequence DNA-Binding Factor 2	Protein Coding	33	GC02M075652	0.13	https://www.genecards.org/cgi-bin/carddisp.pl?gene=GCFC2
**CD24**	CD24 Molecule	Protein Coding	33	GC06M106969	0.13	https://www.genecards.org/cgi-bin/carddisp.pl?gene=CD24
**GDPD4**	Glycerophosphodiester Phosphodiesterase Domain Containing 4	Protein Coding	31	GC11M077216	0.13	https://www.genecards.org/cgi-bin/carddisp.pl?gene=GDPD4

**CCN5**	Cellular Communication Network Factor 5	Protein Coding	31	GC20P044715	0.13	https://www.genecards.org/cgi-bin/carddisp.pl?gene=CCN5
**CSRNP1**	Cysteine And Serine Rich Nuclear Protein 1	Protein Coding	31	GC03M039159	0.13	https://www.genecards.org/cgi-bin/carddisp.pl?gene=CSRNP1
**ZBED3**	Zinc Finger BED-Type Containing 3	Protein Coding	31	GC05M077072	0.13	https://www.genecards.org/cgi-bin/carddisp.pl?gene=ZBED3
**ERV3-1**	Endogenous Retrovirus Group 3 Member 1, Envelope	Protein Coding	29	GC07M065002	0.13	https://www.genecards.org/cgi-bin/carddisp.pl?gene=ERV3-1
**HEXD**	Hexosaminidase D	Protein Coding	26	GC17P082419	0.13	https://www.genecards.org/cgi-bin/carddisp.pl?gene=HEXD
**NAA80**	N-Alpha-Acetyltransferase 80, NatH Catalytic Subunit	Protein Coding	25	GC03M050310	0.13	https://www.genecards.org/cgi-bin/carddisp.pl?gene=NAA80
**TSBP1**	Testis Expressed Basic Protein 1	Protein Coding	23	GC06M032288	0.13	https://www.genecards.org/cgi-bin/carddisp.pl?gene=TSBP1
**MIR192**	MicroRNA 192	RNA Gene	21	GC11M064891	0.13	https://www.genecards.org/cgi-bin/carddisp.pl?gene=MIR192

**MIRLET7A1**	MicroRNA Let-7a-1	RNA Gene	21	GC09P094175	0.13	https://www.genecards.org/cgi-bin/carddisp.pl?gene=MIRLET7A1
**MIR155HG**	MIR155 Host Gene	RNA Gene	20	GC21P025561	0.13	https://www.genecards.org/cgi-bin/carddisp.pl?gene=MIR155HG
**MIR191**	MicroRNA 191	RNA Gene	20	GC03M049247	0.13	https://www.genecards.org/cgi-bin/carddisp.pl?gene=MIR191
**MIR181B1**	MicroRNA 181b-1	RNA Gene	19	GC01M198858	0.13	https://www.genecards.org/cgi-bin/carddisp.pl?gene=MIR181B1
**MIR302D**	MicroRNA 302d	RNA Gene	18	GC04M112648	0.13	https://www.genecards.org/cgi-bin/carddisp.pl?gene=MIR302D
**MIR302B**	MicroRNA 302b	RNA Gene	17	GC04M112681	0.13	https://www.genecards.org/cgi-bin/carddisp.pl?gene=MIR302B
**HAS2-AS1**	HAS2 Antisense RNA 1	RNA Gene	17	GC08P121639	0.13	https://www.genecards.org/cgi-bin/carddisp.pl?gene=HAS2-AS1
**SAA3P**	Serum Amyloid A3, Pseudogene	Pseudogene	14	GC11M018112	0.13	https://www.genecards.org/cgi-bin/carddisp.pl?gene=SAA3P

**GRASLND**	Glycosaminoglycan Regulatory Associated Long Non-Coding RNA	RNA Gene	11	GC02M006911	0.13	https://www.genecards.org/cgi-bin/carddisp.pl?gene=GRASLND
**UGT1A**	UDP Glucuronosyltransferase Family 1 Member A Complex Locus	Uncategorized	9	GC02P233586	0.13	https://www.genecards.org/cgi-bin/carddisp.pl?gene=UGT1A
**TRAP**	Triiodothyronine Receptor Auxiliary Protein	Protein Coding	4	GC00U923131	0.13	https://www.genecards.org/cgi-bin/carddisp.pl?gene=TRAP
**STQTL2**	Stature Quantitative Trait Locus 2	Genetic Locus	2	GC07U901815	0.13	https://www.genecards.org/cgi-bin/carddisp.pl?gene=STQTL2

Table S5 | The overlapping gene symbols between disease (osteoarthritis) and drug (GJ oral liquid).

Gene Symbol
PGR
PTGS1
PTGS2
ADRB2
SLC6A4
OPRM1
BCL2
BAX
CASP9
JUN
CASP3
CASP8
PRKCA
PON1
PLAU
OPRD1
F7
NOS2
AR
PPARG
RELA
IKBKB
AKT1
TNFSF15
MAPK8
MMP1
STAT1
HMOX1
CYP3A4
CYP1A1
ICAM1
SELE
VCAM1

ALOX5
HAS2
GSTP1
AHR
NR1I3
DIO1
PPP3CA
GSTM1
SLPI
ESR1
HTR3A
DRD2
NR3C1
CCNA2
EGFR
KDR
ESR2
MAPK14
GSK3B
MMP3
VEGFA
CCND1
BCL2L1
FOS
CDKN1A
EIF6
MMP2
MMP9
MAPK1
IL10
EGF
IL6
TP63
ELK1
NFKBIA
POR
ODC1

SOD1
HIF1A
ERBB2
CAV1
MYC
F3
GJA1
IL1B
CCL2
PTGER3
CXCL8
BIRC5
NOS3
HSPB1
IL2
CCNB1
PLAT
THBD
SERPINE1
COL1A1
IFNG
IL1A
MPO
NFE2L2
PARP1
COL3A1
CXCL11
CXCL2
PPARA
HSF1
CRP
CXCL10
CHUK
SPP1
RUNX2
E2F1
E2F2

CTSD
IGFBP3
IGF2
CD40LG
IRF1
ALB
CTNNB1
CASP7
MMP10
ADH1B
LYZ
OLR1
MET
IL4
HSD3B2
G6PD
SREBF1
NOX1
ACLY
ADH1A
HPSE
CD44
BMPR2
GNRH1
CRH
GRM1
VCP

Supplementary Table S6 | Details of the active ingredients and the gene symbols

Drug	Compound	ID	Target
	Jaranol	HQ1	NOS2<br>PTGS1
	hederagenin	HQ2	PGR<br>NCOA2
	(3S,8S,9S,10R,13R,14S,1	HQ3	PGR
	3,9-di-O-methylnissolin	HQ4	NOS2<br>PTGS1
Astragali	7-O-methylisomucronulato	HQ5	NOS2<br>PTGS1
	9,10-dimethoxypterocarpar	HQ6	PTGS2<br>NCOA2
	(6aR,11aR)-9,10-dimethox	HQ7	NOS2<br>PTGS1
	Bifendate	HQ8	PTGS2<br>KDR
	formononetin	HQ9	NOS2<br>PTGS1
	Calycosin	HQ10	NOS2<br>PTGS1
	40957-99-1	DZ1	KCNH2<br>SCN5A
	olivil	DZ2	PTGS2<br>NCOA2
	Erythraline	DZ3	PTGS1<br>CHRM3
	AIDS214634	DZ4	PTGS2<br>NCOA2
	ent-Epicatechin	DZ5	PTGS1<br>ESR1
	Yangambin	DZ6	KCNH2<br>SCN5A
	(+)-medioresinol	DZ7	KCNH2<br>SCN5A
	(-)-Tabernemontanine	DZ8	PTGS1<br>CHRM3
	Cyclopamine	DZ9	AR<br>NR3C2
Eucommiae cortex	Dehydrodiconiferyl alcoho	DZ10	PTGS1<br>KCNH2
	Cinchonan-9-al, 6'-methox	DZ11	PTGS1<br>CHRM3
	Helenalin	DZ12	GABRA1
	(+)-Eudesmin	DZ13	CHRM3<br>KCNH2
	4-[(2S,3R)-5-[(E)-3-hydro	DZ14	KCNH2<br>ESR1

	hirsutin_qt	DZ15	NOS2 PTGS1
	liriodendrin_qt	DZ16	NCOA2
	beta-carotene	DZ17	AKT1 VEGFA
	(E)-3-[4-[(1R,2R)-2-hydro	DZ18	NOS2 ESR1
	Syringetin	DZ19	NOS2 ESR1
	sesamin	TSZ1	PTGS2 SCN5A
	NSC63551	TSZ2	PGR
Semen Cuseutae	campest-5-en-3beta-ol	TSZ3	PGR
	Isofucosterol	TSZ4	PGR NR3C2
	matrine	TSZ5	RELA MMP2
	CLR	TSZ6	PGR NR3C2
	berberine	YHS1	NOS2 PTGS1
	coptisine	YHS2	NOS2 PTGS1
	Cryptopin	YHS3	PTGS1 CHRM3
	Dihydrochelerythrine	YHS4	NOS2 PTGS1
	Dihydrosanguinarine	YHS5	NOS2 PTGS1
	sanguinarine	YHS6	PTGS1 PTGS2
	(S)-Scoulerine	YHS7	PTGS1 CHRM3
	Cavidine	YHS8	PTGS1 CHRM3
	(R)-Canadine	YHS9	PTGS1 CHRM3
	sitosterol	YHS10	PGR NCOA2
	Hyndarin	YHS11	CHRM3 KCNH2
	(-)-alpha-N-methylcanadin	YHS12	CHRM3 KCNH2
	Capaurine	YHS13	PTGS1 CHRM3
	Clarkeanidine	YHS14	PTGS1 CHRM3
	CORYDALINE	YHS15	CHRM3 KCNH2
	Corydalmine	YHS16	PTGS1 CHRM3

Rhizoma Corydalis	Corydine	YHS17	PTGS1<br>CHRM3
	18797-79-0	YHS18	PTGS1<br>CHRM3
	Corynoloxine	YHS19	PTGS1<br>AR
	methyl-[2-(3,4,6,7-tetramet	YHS20	PTGS1<br>KCNH2
	dehydrocavidine	YHS21	PTGS1<br>CHRM3
	Dehydrocorybulbine	YHS22	NOS2<br>PTGS1
	dehydrocorydaline	YHS23	NOS2<br>PTGS1
	Dehydrocorydalmine	YHS24	NOS2<br>PTGS1
	demethylcorydalmatine	YHS25	PTGS1<br>CHRM3
	13-methyldehydrocorydaln	YHS26	NOS2<br>PTGS1
	(1S,8'R)-6,7-dimethoxy-2-	YHS27	PTGS1<br>CHRM3
	Izoteolin	YHS28	PTGS1<br>CHRM3
	isocorybulbine	YHS29	PTGS1<br>CHRM3
	leonticine	YHS30	PTGS1<br>CHRM3
	13-methylpalmatrubine	YHS31	PTGS1<br>KCNH2
	N-methyllaurotetanine	YHS32	PTGS1<br>CHRM3
	norglaucing	YHS33	PTGS1<br>CHRM3
	pontevedrine	YHS34	PTGS1<br>KCNH2
	pseudocoptisine	YHS35	NOS2<br>PTGS1
	24240-05-9	YHS36	PTGS1<br>CHRM3
	saulatine	YHS37	CHRM3<br>KCNH2
	stylopine	YHS38	PTGS1<br>CHRM3
	Tetrahydrocorysamine	YHS39	PTGS1<br>CHRM3
	tetrahydroprotopapaverine	YHS40	CHRM3<br>KCNH2
	ST057701	YHS41	PTGS1<br>CHRM3
	2,3,9,10-tetramethoxy-13-r	YHS42	NOS2<br>PTGS1
	palmatine	YHS43	NOS2<br>PTGS1

	Fumarine	YHS44	PTGS1<br>CHRM3
	Isocorypalmine	YHS45	PTGS1<br>CHRM3
	bicuculline	YHS46	PTGS1<br>KCNH2
	C09367	YHS47	PTGS1<br>CHRM3
Astragali, Eucommiae co	Mairin	A1	PGR
Astragali, Eucommiae co	kaempferol	B1	NOS2<br>PTGS1
Astragali, Eucommiae co	quercetin	C1	PTGS1<br>AR
Astragali, Semen Cuseuta	isorhamnetin	D1	NOS2<br>PTGS1
Salviae liguliobae, Salvia	3-beta-Hydroxymethyllene	E1	CHRM1<br>PTGS2
Salviae liguliobae, Angel	beta-sitosterol	F1	PGR<br>NCOA2
Angelicae Sinensis, Rhiz	Stigmasterol	G1	PGR<br>NR3C2

Supplementary Table S7. The top ten potentially effective compounds and the docking compoun

Pubchem ID	MolId	Compounds	Code name	Degree
5280343	MOL000098	quercetin	C1 (HQ, DZ,	548
5280863	MOL000422	kaempferol	B1 (HQ, DZ,	160
222284	MOL000358	beta-sitostero	F1 (DZ, DG,	81
5280794	MOL000449	Stigmasterol	G1	54
5281654	MOL000354	isorhamnetin	D1	50
15689652	MOL000378	7-O-methylis	HQ5	33
5280378	MOL000392	formononetir	HQ9	29
94175	MOL009031	Epiquinidine	DZ11	29
N/A	MOL009015	(-)-Tabernem	DZ8	29
439654	MOL000217	(S)-Scoulerir	YHS7	28
72307	MOL001558	sesamin	TSZ1	22
5318290	MOL007059	3-Hydroxym	E1 (DS, DZ)	22

ds in the prescription

Table S8 | Details of the PPI network.

#node1	node2	node1_string_id	node2_string_id	neighborhood_on_chromosome	gene_fusion	phylogenetic_cooccurrence	homology	coexpression	experimentally_determined_interaction	database_annotated	automated_textmining	combined_score
ACLY	MAPK1	9606.ENSP00000253792	9606.ENSP00000215832	0	0	0	0	0.061	0	0.9	0.124	0.91
ACLY	MPO	9606.ENSP00000253792	9606.ENSP00000225275	0	0	0	0	0	0	0.9	0.079	0.904
ACLY	MYC	9606.ENSP00000253792	9606.ENSP00000479618	0	0	0	0	0.063	0	0	0.404	0.418
ACLY	PPARG	9606.ENSP00000253792	9606.ENSP00000287820	0	0	0	0	0	0	0	0.425	0.425
ACLY	PPARA	9606.ENSP00000253792	9606.ENSP00000385523	0	0	0	0	0	0	0	0.45	0.45
ACLY	GSK3B	9606.ENSP00000253792	9606.ENSP00000324806	0	0	0	0	0	0.379	0	0.188	0.474
ACLY	AKT1	9606.ENSP00000253792	9606.ENSP00000451828	0	0	0	0	0	0	0	0.742	0.743

ACLY	G6PD	9606.ENSP000025 3792	9606.ENSP000003 77192	0	0	0	0	0.079	0	0	0.786	0.795
ACLY	SREBF1	9606.ENSP000025 3792	9606.ENSP000003 48069	0	0	0	0	0.063	0	0	0.848	0.852
ACLY	LYZ	9606.ENSP000025 3792	9606.ENSP000002 61267	0	0	0	0	0	0	0.9	0	0.9
ACLY	VCP	9606.ENSP000025 3792	9606.ENSP000003 51777	0	0	0	0	0.079	0	0.9	0.105	0.91
ADH1A	GSTM1	9606.ENSP000020 9668	9606.ENSP000003 11469	0	0	0	0	0.061	0	0.65	0.257	0.734
ADH1A	GSTP1	9606.ENSP000020 9668	9606.ENSP000003 81607	0	0	0	0	0	0	0.65	0.387	0.776
ADH1A	ADH1B	9606.ENSP000020 9668	9606.ENSP000003 06606	0	0	0.449	0.985	0.828	0	0.9	0.892	0.982
ADH1B	OPRD1	9606.ENSP000030 6606	9606.ENSP000002 34961	0	0	0	0	0	0	0	0.468	0.468
ADH1B	SLC6A4	9606.ENSP000030 6606	9606.ENSP000002 61707	0	0	0	0	0	0	0	0.411	0.41

ADH1B	DRD2	9606.ENSP0000306606	9606.ENSP00000354859	0	0	0	0	0	0	0	0.444	0.444
ADH1B	GSTP1	9606.ENSP0000306606	9606.ENSP00000381607	0	0	0	0	0	0	0.65	0.296	0.743
ADH1B	GSTM1	9606.ENSP0000306606	9606.ENSP00000311469	0	0	0	0	0.061	0	0.65	0.413	0.79
ADRB2	NR3C1	9606.ENSP0000305372	9606.ENSP00000231509	0	0	0	0	0	0	0	0.478	0.478
ADRB2	OPRD1	9606.ENSP0000305372	9606.ENSP00000234961	0	0	0	0.674	0	0.379	0	0.701	0.518
ADRB2	SLC6A4	9606.ENSP0000305372	9606.ENSP00000261707	0	0	0	0	0	0.073	0	0.511	0.527
ADRB2	IL1B	9606.ENSP0000305372	9606.ENSP00000263341	0	0	0	0	0.088	0.379	0	0.624	0.768
ADRB2	ICAM1	9606.ENSP0000305372	9606.ENSP00000264832	0	0	0	0	0	0	0	0.529	0.529
ADRB2	EGF	9606.ENSP0000305372	9606.ENSP00000265171	0	0	0	0	0	0.062	0.9	0.295	0.928

ADRB2	ERBB2	9606.ENSP00000305372	9606.ENSP00000269571	0	0	0	0	0	0.299	0	0.83	0.876
ADRB2	EGFR	9606.ENSP00000305372	9606.ENSP00000275493	0	0	0	0	0	0.186	0.9	0.293	0.937
ADRB2	CRH	9606.ENSP00000305372	9606.ENSP00000276571	0	0	0	0	0	0	0.9	0.512	0.949
ADRB2	PPARG	9606.ENSP00000305372	9606.ENSP00000287820	0	0	0	0	0	0	0	0.416	0.416
ADRB2	ALB	9606.ENSP00000305372	9606.ENSP00000295897	0	0	0	0	0	0	0	0.416	0.416
ADRB2	NOS3	9606.ENSP00000305372	9606.ENSP00000297494	0	0	0	0	0	0	0	0.539	0.539
ADRB2	PPP3CA	9606.ENSP00000305372	9606.ENSP00000378323	0	0	0	0	0	0.388	0	0.08	0.413
ADRB2	AKT1	9606.ENSP00000305372	9606.ENSP00000451828	0	0	0	0	0	0.072	0	0.44	0.458
ADRB2	IL6	9606.ENSP00000305372	9606.ENSP00000385675	0	0	0	0	0.063	0	0	0.447	0.459

ADRB2	FOS	9606.ENSP0000305372	9606.ENSP00000306245	0	0	0	0	0	0	0	0.473	0.473
ADRB2	HTR3A	9606.ENSP0000305372	9606.ENSP00000347754	0	0	0	0	0	0.047	0	0.615	0.617
AHR	HMOX1	9606.ENSP0000242057	9606.ENSP00000216117	0	0	0	0	0.061	0	0	0.475	0.485
AHR	CCL2	9606.ENSP0000242057	9606.ENSP00000225831	0	0	0	0	0	0	0	0.616	0.616
AHR	IL2	9606.ENSP0000242057	9606.ENSP00000226730	0	0	0	0	0	0	0	0.48	0.48
AHR	CCND1	9606.ENSP0000242057	9606.ENSP00000227507	0	0	0	0	0	0	0	0.403	0.403
AHR	IL4	9606.ENSP0000242057	9606.ENSP00000231449	0	0	0	0	0	0	0	0.477	0.477
AHR	NR3C1	9606.ENSP0000242057	9606.ENSP00000231509	0	0	0	0	0.059	0.05	0	0.582	0.594
AHR	MAPK8	9606.ENSP0000242057	9606.ENSP00000378974	0	0	0	0	0	0	0	0.401	0.4

AHR	JUN	9606.ENSP00000242057	9606.ENSP00000360266	0	0	0	0	0	0	0	0.43	0.43
AHR	AKT1	9606.ENSP00000242057	9606.ENSP00000451828	0	0	0	0	0	0	0	0.447	0.447
AHR	PGR	9606.ENSP00000242057	9606.ENSP00000325120	0	0	0	0	0	0.05	0	0.444	0.449
AHR	GSTM1	9606.ENSP00000242057	9606.ENSP00000311469	0	0	0	0	0	0	0	0.456	0.456
AHR	F3	9606.ENSP00000242057	9606.ENSP00000334145	0	0	0	0	0	0	0	0.492	0.493
AHR	ALB	9606.ENSP00000242057	9606.ENSP00000295897	0	0	0	0	0.061	0	0	0.487	0.498
AHR	PPARA	9606.ENSP00000242057	9606.ENSP00000385523	0	0	0	0	0.059	0.05	0	0.484	0.499
AHR	SPP1	9606.ENSP00000242057	9606.ENSP00000378517	0	0	0	0	0	0	0	0.501	0.501
AHR	NOS2	9606.ENSP00000242057	9606.ENSP00000327251	0	0	0	0	0	0	0	0.505	0.505

AHR	GSTP1	9606.ENSP000002 42057	9606.ENSP0000038 1607	0	0	0	0	0	0	0	0.51	0.51
AHR	MMP1	9606.ENSP000002 42057	9606.ENSP0000032 2788	0	0	0	0	0.069	0	0	0.503	0.518
AHR	ESR2	9606.ENSP000002 42057	9606.ENSP0000034 3925	0	0	0	0	0	0.05	0	0.514	0.519
AHR	CAV1	9606.ENSP000002 42057	9606.ENSP0000033 9191	0	0	0	0	0.064	0	0	0.546	0.557
AHR	MMP9	9606.ENSP000002 42057	9606.ENSP0000036 1405	0	0	0	0	0	0	0	0.56	0.56
AHR	IRF1	9606.ENSP000002 42057	9606.ENSP0000024 5414	0	0	0	0	0.065	0	0	0.557	0.569
AHR	CTNNB1	9606.ENSP000002 42057	9606.ENSP0000034 4456	0	0	0	0	0	0.379	0	0.341	0.573
AHR	ICAM1	9606.ENSP000002 42057	9606.ENSP0000026 4832	0	0	0	0	0.076	0	0	0.56	0.576
AHR	RELA	9606.ENSP000002 42057	9606.ENSP0000038 4273	0	0	0	0	0	0.379	0	0.378	0.597

AHR	MYC	9606.ENSP0000242057	9606.ENSP000004796 18	0	0	0	0	0	0	0	0.638	0.638
AHR	EGFR	9606.ENSP0000242057	9606.ENSP0000275493	0	0	0	0	0.061	0	0	0.635	0.643
AHR	IL1B	9606.ENSP0000242057	9606.ENSP0000263341	0	0	0	0	0.071	0	0	0.652	0.663
AHR	PPARG	9606.ENSP0000242057	9606.ENSP0000287820	0	0	0	0	0	0.05	0	0.674	0.677
AHR	PTGS2	9606.ENSP0000242057	9606.ENSP0000356438	0	0	0	0	0.098	0	0	0.671	0.69
AHR	CXCL8	9606.ENSP0000242057	9606.ENSP0000306512	0	0	0	0	0.098	0	0	0.671	0.691
AHR	AR	9606.ENSP0000242057	9606.ENSP0000363822	0	0	0	0	0	0.384	0	0.589	0.736
AHR	VEGFA	9606.ENSP0000242057	9606.ENSP0000478570	0	0	0	0	0	0	0	0.744	0.744
AHR	IL10	9606.ENSP0000242057	9606.ENSP0000412237	0	0	0	0	0	0	0	0.745	0.745

AHR	NR1I3	9606.ENSP0000242057	9606.ENSP000356959	0	0	0	0	0	0.05	0	0.768	0.771
AHR	IL6	9606.ENSP0000242057	9606.ENSP000385675	0	0	0	0	0.069	0	0	0.769	0.777
AHR	CYP3A4	9606.ENSP0000242057	9606.ENSP000337915	0	0	0	0	0.061	0	0	0.783	0.788
AHR	STAT1	9606.ENSP0000242057	9606.ENSP000354394	0	0	0	0	0.061	0.299	0	0.704	0.788
AHR	NFE2L2	9606.ENSP0000242057	9606.ENSP000380252	0	0	0	0	0	0.379	0	0.742	0.834
AHR	ESR1	9606.ENSP0000242057	9606.ENSP000405330	0	0	0	0	0	0.384	0	0.826	0.888
AHR	CYP1A1	9606.ENSP0000242057	9606.ENSP000369050	0	0	0	0	0.061	0	0.9	0.975	0.997
AKT1	MAPK1	9606.ENSP0000451828	9606.ENSP000215832	0	0	0.304	0.592	0.066	0.146	0.8	0.948	0.905
AKT1	HMOX1	9606.ENSP0000451828	9606.ENSP000216117	0	0	0	0	0	0.379	0	0.828	0.889

AKT1	NFKBIA	9606.ENSP0000451828	9606.ENSP00000216797	0	0	0	0	0.061	0.433	0	0.586	0.76
AKT1	MMP2	9606.ENSP0000451828	9606.ENSP00000219070	0	0	0	0	0.061	0	0	0.833	0.836
AKT1	SERPINE1	9606.ENSP0000451828	9606.ENSP00000223095	0	0	0	0	0	0	0	0.761	0.761
AKT1	MPO	9606.ENSP0000451828	9606.ENSP00000225275	0	0	0	0	0	0.058	0	0.559	0.567
AKT1	CCL2	9606.ENSP0000451828	9606.ENSP00000225831	0	0	0	0	0	0	0	0.794	0.794
AKT1	COL1A1	9606.ENSP0000451828	9606.ENSP00000225964	0	0	0	0	0.059	0.061	0	0.483	0.504
AKT1	IL2	9606.ENSP0000451828	9606.ENSP00000226730	0	0	0	0	0	0	0.9	0.793	0.978
AKT1	CCND1	9606.ENSP0000451828	9606.ENSP00000227507	0	0	0	0	0.061	0	0	0.941	0.943
AKT1	IFNG	9606.ENSP0000451828	9606.ENSP00000229135	0	0	0	0	0	0	0	0.53	0.53

AKT1	MAPK14	9606.ENSP0000451828	9606.ENSP00000229795	0	0	0.303	0.623	0.082	0.715	0.6	0.858	0.931
AKT1	IL4	9606.ENSP0000451828	9606.ENSP00000231449	0	0	0	0	0.062	0	0	0.656	0.664
AKT1	NR3C1	9606.ENSP0000451828	9606.ENSP00000231509	0	0	0	0	0	0.407	0	0.561	0.728
AKT1	ODC1	9606.ENSP0000451828	9606.ENSP00000234111	0	0	0	0	0.059	0.077	0	0.435	0.466
AKT1	CTSD	9606.ENSP0000451828	9606.ENSP00000236671	0	0	0	0	0.095	0.085	0	0.423	0.481
AKT1	IRF1	9606.ENSP0000451828	9606.ENSP00000245414	0	0	0	0	0	0.082	0	0.635	0.651
AKT1	HSPB1	9606.ENSP0000451828	9606.ENSP00000248553	0	0	0	0	0	0.698	0	0.864	0.957
AKT1	CRP	9606.ENSP0000451828	9606.ENSP00000255030	0	0	0	0	0	0	0	0.545	0.545
AKT1	CCNB1	9606.ENSP0000451828	9606.ENSP00000256442	0	0	0	0	0	0.138	0	0.804	0.824

AKT1	IL1B	9606. ENSP0 00004 51828	9606.E NSP00 00026 3341	0	0	0	0	0	0.059	0	0.669	0.675
AKT1	KDR	9606. ENSP0 00004 51828	9606.E NSP00 00026 3923	0	0	0	0.555	0	0.096	0	0.905	0.458
AKT1	ICAM1	9606. ENSP0 00004 51828	9606.E NSP00 00026 4832	0	0	0	0	0	0	0	0.818	0.818
AKT1	EGF	9606. ENSP0 00004 51828	9606.E NSP00 00026 5171	0	0	0	0	0	0.05	0	0.932	0.933
AKT1	ERBB2	9606. ENSP0 00004 51828	9606.E NSP00 00026 9571	0	0	0	0.556	0.053	0.543	0	0.943	0.737
AKT1	SOD1	9606. ENSP0 00004 51828	9606.E NSP00 00027 0142	0	0	0	0	0	0.064	0.9	0.64	0.963
AKT1	CCNA2	9606. ENSP0 00004 51828	9606.E NSP00 00027 4026	0	0	0	0	0	0.354	0	0.668	0.776
AKT1	EGFR	9606. ENSP0 00004 51828	9606.E NSP00 00027 5493	0	0	0	0.554	0	0.431	0	0.973	0.678
AKT1	GNRH1	9606. ENSP0 00004 51828	9606.E NSP00 00027 6414	0	0	0	0	0.062	0	0	0.644	0.652

AKT1	GJA1	9606.ENSP00000451828	9606.ENSP00000282561	0	0	0	0	0	0.053	0	0.815	0.818
AKT1	PPARG	9606.ENSP00000451828	9606.ENSP00000287820	0	0	0	0	0.061	0.085	0	0.674	0.695
AKT1	BAX	9606.ENSP00000451828	9606.ENSP00000293288	0	0	0	0	0.061	0.379	0	0.581	0.734
AKT1	VCAM1	9606.ENSP00000451828	9606.ENSP00000294728	0	0	0	0	0	0	0	0.791	0.791
AKT1	ALB	9606.ENSP00000451828	9606.ENSP00000295897	0	0	0	0	0	0	0	0.772	0.772
AKT1	NOS3	9606.ENSP00000451828	9606.ENSP00000297494	0	0	0	0	0.049	0.693	0.9	0.97	0.999
AKT1	MMP3	9606.ENSP00000451828	9606.ENSP00000299855	0	0	0	0	0	0	0	0.585	0.585
AKT1	BIRC5	9606.ENSP00000451828	9606.ENSP00000301633	0	0	0	0	0	0.108	0	0.448	0.486
AKT1	BCL2L1	9606.ENSP00000451828	9606.ENSP00000302564	0	0	0	0	0.073	0.32	0.9	0.902	0.993

AKT1	CXCL10	9606.ENSP00000451828	9606.ENSP00000305651	0	0	0	0	0	0	0	0.48	0.48
AKT1	FOS	9606.ENSP00000451828	9606.ENSP00000306245	0	0	0	0	0	0.08	0	0.806	0.815
AKT1	CXCL8	9606.ENSP00000451828	9606.ENSP00000306512	0	0	0	0	0	0	0	0.707	0.707
AKT1	OLR1	9606.ENSP00000451828	9606.ENSP00000309124	0	0	0	0	0	0.063	0	0.657	0.665
AKT1	CASP3	9606.ENSP00000451828	9606.ENSP00000311032	0	0	0	0	0.055	0.393	0	0.95	0.969
AKT1	MMP1	9606.ENSP00000451828	9606.ENSP00000322788	0	0	0	0	0	0	0	0.547	0.547
AKT1	GSK3B	9606.ENSP00000451828	9606.ENSP00000324806	0	0	0.218	0.576	0.049	0.995	0.9	0.918	0.999
AKT1	PGR	9606.ENSP00000451828	9606.ENSP00000325120	0	0	0	0	0.061	0.085	0	0.661	0.683
AKT1	NOS2	9606.ENSP00000451828	9606.ENSP00000327251	0	0	0	0	0.063	0.065	0.8	0.543	0.909

AKT1	CASP9	9606.ENSP0000451828	9606.ENSP000000330237	0	0	0	0	0	0.393	0.9	0.919	0.994
AKT1	SELE	9606.ENSP0000451828	9606.ENSP000000331736	0	0	0	0	0	0.063	0	0.53	0.54
AKT1	F3	9606.ENSP0000451828	9606.ENSP000000334145	0	0	0	0	0	0	0	0.478	0.478
AKT1	CAV1	9606.ENSP0000451828	9606.ENSP000000339191	0	0	0	0	0	0.057	0.9	0.815	0.981
AKT1	ESR2	9606.ENSP0000451828	9606.ENSP000000343925	0	0	0	0	0	0.407	0	0.722	0.828
AKT1	CTNNB1	9606.ENSP0000451828	9606.ENSP000000344456	0	0	0	0	0.083	0.444	0.9	0.959	0.997
AKT1	E2F1	9606.ENSP0000451828	9606.ENSP000000345571	0	0	0	0	0.091	0.124	0.9	0.481	0.953
AKT1	SREBF1	9606.ENSP0000451828	9606.ENSP000000348069	0	0	0	0	0.085	0.06	0	0.806	0.819
AKT1	PTGER3	9606.ENSP0000451828	9606.ENSP000000349003	0	0	0	0	0	0.072	0	0.4	0.419

AKT1	CASP8	9606.ENSP0000451828	9606.ENSP000003251273	0	0	0	0	0	0.064	0	0.74	0.746
AKT1	VCP	9606.ENSP0000451828	9606.ENSP000003251777	0	0	0	0	0.072	0.418	0	0.618	0.776
AKT1	STAT1	9606.ENSP0000451828	9606.ENSP000003254394	0	0	0	0	0	0.378	0	0.683	0.794
AKT1	DRD2	9606.ENSP0000451828	9606.ENSP000003254859	0	0	0	0	0	0.072	0	0.603	0.616
AKT1	GRM1	9606.ENSP0000451828	9606.ENSP000003254896	0	0	0	0	0	0.085	0	0.603	0.621
AKT1	E2F2	9606.ENSP0000451828	9606.ENSP000003255249	0	0	0	0	0.051	0.124	0	0.432	0.487
AKT1	PARP1	9606.ENSP0000451828	9606.ENSP000003255759	0	0	0	0	0.061	0.057	0	0.783	0.791
AKT1	PTGS2	9606.ENSP0000451828	9606.ENSP000003256438	0	0	0	0	0.062	0.058	0	0.848	0.854
AKT1	CASP7	9606.ENSP0000451828	9606.ENSP000003258327	0	0	0	0	0	0.064	0	0.664	0.672

AKT1	CHUK	9606.ENSP00000451828	9606.ENSP00000359424	0	0	0	0.593	0	0.682	0.9	0.673	0.975
AKT1	CD40LG	9606.ENSP00000451828	9606.ENSP00000359663	0	0	0	0	0	0.072	0.9	0.482	0.947
AKT1	JUN	9606.ENSP00000451828	9606.ENSP00000360266	0	0	0	0	0	0.072	0	0.91	0.913
AKT1	RUNX2	9606.ENSP00000451828	9606.ENSP00000360493	0	0	0	0	0	0	0	0.808	0.808
AKT1	MMP9	9606.ENSP00000451828	9606.ENSP00000361405	0	0	0	0	0.061	0	0	0.885	0.887
AKT1	PLAU	9606.ENSP00000451828	9606.ENSP00000361850	0	0	0	0	0	0.05	0	0.536	0.541
AKT1	NOX1	9606.ENSP00000451828	9606.ENSP00000362057	0	0	0	0	0	0.079	0	0.754	0.764
AKT1	ALOX5	9606.ENSP00000451828	9606.ENSP00000363512	0	0	0	0	0	0	0	0.429	0.429
AKT1	AR	9606.ENSP00000451828	9606.ENSP00000363822	0	0	0	0	0	0.407	0	0.89	0.932

AKT1	IGFBP3	9606.ENSP00000451828	9606.ENSP00000370473	0	0	0	0	0	0	0	0.743	0.743
AKT1	G6PD	9606.ENSP00000451828	9606.ENSP00000377192	0	0	0	0	0.087	0	0	0.428	0.455
AKT1	PPP3CA	9606.ENSP00000451828	9606.ENSP00000378323	0	0	0	0	0	0.409	0	0.361	0.606
AKT1	SPP1	9606.ENSP00000451828	9606.ENSP00000378517	0	0	0	0	0	0.079	0	0.774	0.783
AKT1	MAPK8	9606.ENSP00000451828	9606.ENSP00000378974	0	0	0.417	0.573	0.084	0.328	0	0.96	0.684
AKT1	NFE2L2	9606.ENSP00000451828	9606.ENSP00000380252	0	0	0	0	0	0.344	0	0.786	0.854
AKT1	BCL2	9606.ENSP00000451828	9606.ENSP00000381185	0	0	0	0	0.061	0	0.9	0.426	0.941
AKT1	GSTP1	9606.ENSP00000451828	9606.ENSP00000381607	0	0	0	0	0.049	0.064	0	0.388	0.407
AKT1	RELA	9606.ENSP00000451828	9606.ENSP00000384273	0	0	0	0	0.086	0	0	0.776	0.786

AKT1	CDKN1A	9606.ENSP00000451828	9606.ENSP00000384849	0	0	0	0	0	0.696	0.9	0.851	0.995
AKT1	PPARA	9606.ENSP00000451828	9606.ENSP00000385523	0	0	0	0	0	0.085	0	0.471	0.495
AKT1	IL6	9606.ENSP00000451828	9606.ENSP00000385675	0	0	0	0	0.055	0	0	0.886	0.888
AKT1	IGF2	9606.ENSP00000451828	9606.ENSP00000391826	0	0	0	0	0	0.262	0	0.794	0.841
AKT1	OPRM1	9606.ENSP00000451828	9606.ENSP00000394624	0	0	0	0	0	0.072	0	0.42	0.438
AKT1	CD44	9606.ENSP00000451828	9606.ENSP00000398632	0	0	0	0	0	0.153	0	0.702	0.737
AKT1	ESR1	9606.ENSP00000451828	9606.ENSP00000405330	0	0	0	0	0.055	0.407	0.9	0.867	0.991
AKT1	IL10	9606.ENSP00000451828	9606.ENSP00000412237	0	0	0	0	0	0.079	0	0.821	0.828
AKT1	CXCL2	9606.ENSP00000451828	9606.ENSP00000427279	0	0	0	0	0	0	0	0.412	0.412

AKT1	IKBKB	9606.ENSP000004518 28	9606.ENSP000004306 84	0	0	0	0.601	0	0.682	0.9	0.574	0.974
AKT1	HSF1	9606.ENSP000004518 28	9606.ENSP000004315 12	0	0	0	0	0.086	0.098	0	0.572	0.616
AKT1	HIF1A	9606.ENSP000004518 28	9606.ENSP000004379 55	0	0	0	0	0	0.362	0.9	0.827	0.988
AKT1	VEGFA	9606.ENSP000004518 28	9606.ENSP000004785 70	0	0	0	0	0.063	0.342	0	0.95	0.966
AKT1	MYC	9606.ENSP000004518 28	9606.ENSP000004796 18	0	0	0	0	0.052	0.156	0.9	0.915	0.992
ALB	MAPK1	9606.ENSP000002958 97	9606.ENSP000002158 32	0	0	0	0	0	0	0	0.687	0.687
ALB	HMOX1	9606.ENSP000002958 97	9606.ENSP000002161 17	0	0	0	0	0	0	0	0.656	0.656
ALB	MMP2	9606.ENSP000002958 97	9606.ENSP000002190 70	0	0	0	0	0.078	0	0	0.616	0.631
ALB	PLAT	9606.ENSP000002958 97	9606.ENSP000002208 09	0	0	0	0	0.054	0	0	0.537	0.543

ALB	PON1	9606.ENSP00000295897	9606.ENSP00000222381	0	0	0	0	0.077	0.379	0	0.53	0.707
ALB	SERPINE1	9606.ENSP00000295897	9606.ENSP00000223095	0	0	0	0	0.077	0	0.9	0.795	0.979
ALB	MPO	9606.ENSP00000295897	9606.ENSP00000225275	0	0	0	0	0	0	0	0.762	0.762
ALB	CCL2	9606.ENSP00000295897	9606.ENSP00000225831	0	0	0	0	0	0	0	0.815	0.815
ALB	COL1A1	9606.ENSP00000295897	9606.ENSP00000225964	0	0	0	0	0	0	0	0.488	0.488
ALB	IL2	9606.ENSP00000295897	9606.ENSP00000226730	0	0	0	0	0	0	0	0.739	0.739
ALB	CCND1	9606.ENSP00000295897	9606.ENSP00000227507	0	0	0	0	0	0	0	0.636	0.636
ALB	IFNG	9606.ENSP00000295897	9606.ENSP00000229135	0	0	0	0	0	0	0	0.657	0.657
ALB	MAPK14	9606.ENSP00000295897	9606.ENSP00000229795	0	0	0	0	0	0	0	0.593	0.593

ALB	IL4	9606.ENSP00000295897	9606.ENSP00000231449	0	0	0	0	0	0	0	0.687	0.687
ALB	NR3C1	9606.ENSP00000295897	9606.ENSP00000231509	0	0	0	0	0.052	0.165	0	0.556	0.617
ALB	CTSD	9606.ENSP00000295897	9606.ENSP00000236671	0	0	0	0	0.061	0	0	0.576	0.585
ALB	HSPB1	9606.ENSP00000295897	9606.ENSP00000248553	0	0	0	0	0	0	0	0.511	0.511
ALB	CRP	9606.ENSP00000295897	9606.ENSP00000255030	0	0	0	0	0.115	0	0	0.921	0.927
ALB	CCNB1	9606.ENSP00000295897	9606.ENSP00000256442	0	0	0	0	0	0	0	0.497	0.497
ALB	LYZ	9606.ENSP00000295897	9606.ENSP00000261267	0	0	0	0	0.092	0	0	0.583	0.605
ALB	SLC6A4	9606.ENSP00000295897	9606.ENSP00000261707	0	0	0	0	0.051	0	0	0.429	0.435
ALB	IL1A	9606.ENSP00000295897	9606.ENSP00000263339	0	0	0	0	0.062	0	0	0.412	0.424

ALB	IL1B	9606.ENSP0000295897	9606.ENSP00000263341	0	0	0	0	0	0	0	0.681	0.681
ALB	KDR	9606.ENSP0000295897	9606.ENSP00000263923	0	0	0	0	0.061	0	0	0.769	0.774
ALB	ICAM1	9606.ENSP0000295897	9606.ENSP00000264832	0	0	0	0	0	0	0	0.806	0.807
ALB	EGF	9606.ENSP0000295897	9606.ENSP00000265171	0	0	0	0	0	0	0.9	0.808	0.98
ALB	ERBB2	9606.ENSP0000295897	9606.ENSP00000269571	0	0	0	0	0	0	0	0.683	0.683
ALB	SOD1	9606.ENSP0000295897	9606.ENSP00000270142	0	0	0	0	0	0.126	0	0.573	0.611
ALB	CCNA2	9606.ENSP0000295897	9606.ENSP00000274026	0	0	0	0	0	0	0	0.447	0.447
ALB	EGFR	9606.ENSP0000295897	9606.ENSP00000275493	0	0	0	0	0.061	0	0	0.762	0.767
ALB	GNRH1	9606.ENSP0000295897	9606.ENSP00000276414	0	0	0	0	0	0	0	0.554	0.554

ALB	CRH	9606.ENSP0000295897	9606.ENSP00000276571	0	0	0	0	0	0	0	0.531	0.531
ALB	MMP10	9606.ENSP0000295897	9606.ENSP00000279441	0	0	0	0	0.055	0	0	0.46	0.468
ALB	GJA1	9606.ENSP0000295897	9606.ENSP00000282561	0	0	0	0	0	0.05	0	0.574	0.578
ALB	PPARG	9606.ENSP0000295897	9606.ENSP00000287820	0	0	0	0	0.056	0.074	0	0.566	0.588
ALB	BAX	9606.ENSP0000295897	9606.ENSP00000293288	0	0	0	0	0.049	0	0	0.397	0.402
ALB	VCAM1	9606.ENSP0000295897	9606.ENSP00000294728	0	0	0	0	0	0	0	0.788	0.788
ALB	SLPI	9606.ENSP0000295897	9606.ENSP00000342082	0	0	0	0	0.065	0	0	0.389	0.404
ALB	GSTP1	9606.ENSP0000295897	9606.ENSP00000381607	0	0	0	0	0	0	0	0.404	0.404
ALB	DRD2	9606.ENSP0000295897	9606.ENSP00000354859	0	0	0	0	0	0	0	0.411	0.41

ALB	HIF1A	9606.ENSP00000295897	9606.ENSP00000437955	0	0	0	0	0	0	0	0.414	0.414
ALB	CASP7	9606.ENSP00000295897	9606.ENSP00000358327	0	0	0	0	0.059	0	0	0.409	0.42
ALB	ESR2	9606.ENSP00000295897	9606.ENSP00000343925	0	0	0	0	0.052	0.074	0	0.399	0.426
ALB	GSK3B	9606.ENSP00000295897	9606.ENSP00000324806	0	0	0	0	0	0	0	0.432	0.432
ALB	RELA	9606.ENSP00000295897	9606.ENSP00000384273	0	0	0	0	0	0	0	0.433	0.433
ALB	CDKN1A	9606.ENSP00000295897	9606.ENSP00000384849	0	0	0	0	0	0	0	0.468	0.468
ALB	PPARA	9606.ENSP00000295897	9606.ENSP00000385523	0	0	0	0	0.064	0.074	0	0.438	0.47
ALB	OPRM1	9606.ENSP00000295897	9606.ENSP00000394624	0	0	0	0	0	0	0	0.472	0.472
ALB	GSTM1	9606.ENSP00000295897	9606.ENSP00000311469	0	0	0	0	0.062	0	0	0.468	0.48

ALB	THBD	9606.ENSP00000295897	9606.ENSP00000366307	0	0	0	0	0	0	0	0.497	0.497
ALB	NFE2L2	9606.ENSP00000295897	9606.ENSP00000380252	0	0	0	0	0	0	0	0.499	0.499
ALB	PLAU	9606.ENSP00000295897	9606.ENSP00000361850	0	0	0	0	0.054	0	0	0.495	0.502
ALB	SREBF1	9606.ENSP00000295897	9606.ENSP00000348069	0	0	0	0	0.061	0	0	0.498	0.508
ALB	MMP1	9606.ENSP00000295897	9606.ENSP00000322788	0	0	0	0	0.055	0	0	0.504	0.511
ALB	MET	9606.ENSP00000295897	9606.ENSP00000317272	0	0	0	0	0.062	0	0	0.513	0.524
ALB	PGR	9606.ENSP00000295897	9606.ENSP00000325120	0	0	0	0	0.052	0.074	0	0.506	0.529
ALB	CD40LG	9606.ENSP00000295897	9606.ENSP00000359663	0	0	0	0	0	0	0	0.536	0.536
ALB	NOS2	9606.ENSP00000295897	9606.ENSP00000327251	0	0	0	0	0	0	0	0.542	0.542

ALB	PARP1	9606.ENSP00000295897	9606.ENSP00000355759	0	0	0	0	0	0	0	0.542	0.542
ALB	CXCL10	9606.ENSP00000295897	9606.ENSP00000305651	0	0	0	0	0	0	0	0.545	0.545
ALB	CYP1A1	9606.ENSP00000295897	9606.ENSP00000369050	0	0	0	0	0.063	0	0	0.549	0.56
ALB	STAT1	9606.ENSP00000295897	9606.ENSP00000354394	0	0	0	0	0	0	0	0.561	0.561
ALB	RUNX2	9606.ENSP00000295897	9606.ENSP00000360493	0	0	0	0	0	0	0	0.57	0.57
ALB	CTNNB1	9606.ENSP00000295897	9606.ENSP00000344456	0	0	0	0	0	0	0	0.598	0.598
ALB	BCL2L1	9606.ENSP00000295897	9606.ENSP00000302564	0	0	0	0	0.049	0	0	0.6	0.604
ALB	CAV1	9606.ENSP00000295897	9606.ENSP00000339191	0	0	0	0	0	0	0	0.604	0.604
ALB	CASP8	9606.ENSP00000295897	9606.ENSP00000351273	0	0	0	0	0	0	0	0.605	0.605

ALB	SELE	9606.ENSP00000295897	9606.ENSP00000331736	0	0	0	0	0	0	0	0.606	0.606
ALB	MMP3	9606.ENSP00000295897	9606.ENSP00000299855	0	0	0	0	0.055	0	0	0.603	0.608
ALB	F7	9606.ENSP00000295897	9606.ENSP00000364731	0	0	0	0	0.187	0.043	0	0.551	0.62
ALB	AR	9606.ENSP00000295897	9606.ENSP00000363822	0	0	0	0	0.052	0.074	0	0.604	0.622
ALB	CASP9	9606.ENSP00000295897	9606.ENSP00000330237	0	0	0	0	0	0	0	0.633	0.633
ALB	FOS	9606.ENSP00000295897	9606.ENSP00000306245	0	0	0	0	0	0	0	0.64	0.64
ALB	JUN	9606.ENSP00000295897	9606.ENSP00000360266	0	0	0	0	0	0	0	0.649	0.649
ALB	G6PD	9606.ENSP00000295897	9606.ENSP00000377192	0	0	0	0	0	0	0	0.651	0.651
ALB	NOS3	9606.ENSP00000295897	9606.ENSP00000297494	0	0	0	0	0.059	0	0	0.671	0.677

ALB	CYP3A4	9606.ENSP00000295897	9606.ENSP00000337915	0	0	0	0	0.063	0	0	0.671	0.678
ALB	MAPK8	9606.ENSP00000295897	9606.ENSP00000378974	0	0	0	0	0	0	0	0.686	0.686
ALB	ESR1	9606.ENSP00000295897	9606.ENSP00000405330	0	0	0	0	0.061	0.074	0	0.682	0.7
ALB	F3	9606.ENSP00000295897	9606.ENSP00000334145	0	0	0	0	0	0	0	0.734	0.734
ALB	MMP9	9606.ENSP00000295897	9606.ENSP00000361405	0	0	0	0	0.061	0	0	0.732	0.738
ALB	IL10	9606.ENSP00000295897	9606.ENSP00000412237	0	0	0	0	0	0	0	0.776	0.776
ALB	PTGS2	9606.ENSP00000295897	9606.ENSP00000356438	0	0	0	0	0	0	0	0.797	0.797
ALB	MYC	9606.ENSP00000295897	9606.ENSP00000479618	0	0	0	0	0	0	0	0.798	0.798
ALB	CD44	9606.ENSP00000295897	9606.ENSP00000398632	0	0	0	0	0	0	0	0.798	0.798

ALB	CASP3	9606.ENSP00000295897	9606.ENSP00000311032	0	0	0	0	0	0	0	0.835	0.835
ALB	CXCL8	9606.ENSP00000295897	9606.ENSP00000306512	0	0	0	0	0	0	0	0.84	0.841
ALB	IGFBP3	9606.ENSP00000295897	9606.ENSP00000370473	0	0	0	0	0.098	0	0.9	0.473	0.948
ALB	IGF2	9606.ENSP00000295897	9606.ENSP00000391826	0	0	0	0	0.231	0	0.9	0.507	0.958
ALB	SPP1	9606.ENSP00000295897	9606.ENSP00000378517	0	0	0	0	0.076	0	0.9	0.789	0.978
ALB	IL6	9606.ENSP00000295897	9606.ENSP00000385675	0	0	0	0	0	0	0.9	0.841	0.983
ALB	VEGFA	9606.ENSP00000295897	9606.ENSP00000478570	0	0	0	0	0.062	0	0.9	0.871	0.986
ALOX5	MAPK1	9606.ENSP00000363512	9606.ENSP00000215832	0	0	0	0	0	0	0	0.594	0.594
ALOX5	HMOX1	9606.ENSP00000363512	9606.ENSP00000216117	0	0	0	0	0.06	0	0	0.389	0.401

ALOX5	MMP2	9606.ENSP00000363512	9606.ENSP00000219070	0	0	0	0	0	0	0	0.501	0.501
ALOX5	MPO	9606.ENSP00000363512	9606.ENSP00000225275	0	0	0	0	0.12	0	0	0.543	0.581
ALOX5	CCL2	9606.ENSP00000363512	9606.ENSP00000225831	0	0	0	0	0	0	0	0.446	0.446
ALOX5	MAPK14	9606.ENSP00000363512	9606.ENSP00000229795	0	0	0	0	0	0	0	0.478	0.478
ALOX5	IL4	9606.ENSP00000363512	9606.ENSP00000231449	0	0	0	0	0	0	0.9	0.473	0.945
ALOX5	IL1B	9606.ENSP00000363512	9606.ENSP00000263341	0	0	0	0	0.098	0	0	0.477	0.508
ALOX5	ICAM1	9606.ENSP00000363512	9606.ENSP00000264832	0	0	0	0	0.06	0	0	0.4	0.411
ALOX5	PPARG	9606.ENSP00000363512	9606.ENSP00000287820	0	0	0	0	0	0	0	0.421	0.42
ALOX5	CXCL8	9606.ENSP00000363512	9606.ENSP00000306512	0	0	0	0	0	0	0	0.492	0.491

ALOX5	CASP3	9606.ENSP00000363512	9606.ENSP00000311032	0	0	0	0	0	0	0	0.416	0.416
ALOX5	PTGER3	9606.ENSP00000363512	9606.ENSP00000349003	0	0	0	0	0	0	0	0.445	0.445
ALOX5	PTGS1	9606.ENSP00000363512	9606.ENSP00000354612	0	0	0	0	0.095	0	0.9	0.728	0.973
ALOX5	PTGS2	9606.ENSP00000363512	9606.ENSP00000356438	0	0	0	0	0.08	0	0.9	0.758	0.975
ALOX5	MMP9	9606.ENSP00000363512	9606.ENSP00000361405	0	0	0	0	0.159	0	0	0.423	0.494
ALOX5	RELA	9606.ENSP00000363512	9606.ENSP00000384273	0	0	0	0	0	0.379	0	0.189	0.474
ALOX5	CXCL2	9606.ENSP00000363512	9606.ENSP00000427279	0	0	0	0	0	0	0	0.5	0.499
ALOX5	IL10	9606.ENSP00000363512	9606.ENSP00000412237	0	0	0	0	0	0	0	0.51	0.51
ALOX5	IL6	9606.ENSP00000363512	9606.ENSP00000385675	0	0	0	0	0	0	0	0.563	0.563

ALOX5	MAPK8	9606.ENSP00000363512	9606.ENSP00000378974	0	0	0	0	0	0	0	0.65	0.65
ALOX5	VEGFA	9606.ENSP00000363512	9606.ENSP00000478570	0	0	0	0	0	0	0	0.658	0.658
AR	MAPK1	9606.ENSP00000363822	9606.ENSP00000215832	0	0	0	0	0	0.399	0	0.651	0.782
AR	MMP2	9606.ENSP00000363822	9606.ENSP00000219070	0	0	0	0	0.061	0	0	0.4	0.412
AR	CCND1	9606.ENSP00000363822	9606.ENSP00000227507	0	0	0	0	0.061	0.384	0.9	0.795	0.986
AR	MAPK14	9606.ENSP00000363822	9606.ENSP00000229795	0	0	0	0	0	0.073	0.9	0.462	0.945
AR	NR3C1	9606.ENSP00000363822	9606.ENSP00000231509	0	0	0	0.816	0	0.379	0.9	0.806	0.944
AR	ODC1	9606.ENSP00000363822	9606.ENSP00000234111	0	0	0	0	0	0	0	0.423	0.422
AR	HSPB1	9606.ENSP00000363822	9606.ENSP00000248553	0	0	0	0	0	0	0	0.443	0.443

AR	CCNB1	9606.ENSP00000363822	9606.ENSP00000256442	0	0	0	0	0	0.05	0	0.408	0.413
AR	KDR	9606.ENSP00000363822	9606.ENSP00000263923	0	0	0	0	0	0.051	0	0.414	0.42
AR	EGF	9606.ENSP00000363822	9606.ENSP00000265171	0	0	0	0	0	0	0	0.81	0.81
AR	ERBB2	9606.ENSP00000363822	9606.ENSP00000269571	0	0	0	0	0.067	0.077	0	0.829	0.84
AR	SOD1	9606.ENSP00000363822	9606.ENSP00000270142	0	0	0	0	0	0	0	0.625	0.625
AR	CCNA2	9606.ENSP00000363822	9606.ENSP00000274026	0	0	0	0	0	0	0	0.455	0.455
AR	EGFR	9606.ENSP00000363822	9606.ENSP00000275493	0	0	0	0	0.062	0.402	0	0.678	0.803
AR	GNRH1	9606.ENSP00000363822	9606.ENSP00000276414	0	0	0	0	0	0	0	0.849	0.849
AR	VCAM1	9606.ENSP00000363822	9606.ENSP00000294728	0	0	0	0	0	0	0	0.528	0.528

AR	BCL2L1	9606.ENSP00000363822	9606.ENSP00000302564	0	0	0	0	0	0.064	0	0.698	0.706
AR	FOS	9606.ENSP00000363822	9606.ENSP00000306245	0	0	0	0	0.055	0.266	0	0.532	0.647
AR	CXCL8	9606.ENSP00000363822	9606.ENSP00000306512	0	0	0	0	0	0	0	0.638	0.638
AR	CASP3	9606.ENSP00000363822	9606.ENSP00000311032	0	0	0	0	0	0.39	0	0.6	0.746
AR	MET	9606.ENSP00000363822	9606.ENSP00000317272	0	0	0	0	0	0.085	0	0.738	0.75
AR	MMP1	9606.ENSP00000363822	9606.ENSP00000322788	0	0	0	0	0.061	0	0	0.499	0.509
AR	GSK3B	9606.ENSP00000363822	9606.ENSP00000324806	0	0	0	0	0	0.407	0.9	0.49	0.967
AR	PGR	9606.ENSP00000363822	9606.ENSP00000325120	0	0	0	0.813	0.069	0	0.9	0.904	0.919
AR	CYP3A4	9606.ENSP00000363822	9606.ENSP00000337915	0	0	0	0	0	0.085	0	0.433	0.459

AR	CAV1	9606.ENSP000003 63822	9606.ENSP0000033 9191	0	0	0	0	0	0.4	0	0.665	0.791
AR	CTNNB1	9606.ENSP000003 63822	9606.ENSP0000034 4456	0	0	0	0	0	0.385	0.9	0.775	0.984
AR	E2F1	9606.ENSP000003 63822	9606.ENSP0000034 5571	0	0	0	0	0	0.305	0	0.58	0.696
AR	SREBF1	9606.ENSP000003 63822	9606.ENSP0000034 8069	0	0	0	0	0	0.379	0	0.63	0.76
AR	PTGER3	9606.ENSP000003 63822	9606.ENSP0000034 9003	0	0	0	0	0	0	0	0.463	0.463
AR	CASP8	9606.ENSP000003 63822	9606.ENSP0000035 1273	0	0	0	0	0	0.472	0.9	0.399	0.965
AR	VCP	9606.ENSP000003 63822	9606.ENSP0000035 1777	0	0	0	0	0	0.305	0	0.188	0.411
AR	PARP1	9606.ENSP000003 63822	9606.ENSP0000035 5759	0	0	0	0	0	0	0	0.445	0.445
AR	PTGS2	9606.ENSP000003 63822	9606.ENSP0000035 6438	0	0	0	0	0	0.05	0	0.437	0.442

AR	CASP7	9606.ENSP00000363822	9606.ENSP00000358327	0	0	0	0	0	0.325	0	0.228	0.456
AR	CHUK	9606.ENSP00000363822	9606.ENSP00000359424	0	0	0	0	0	0.085	0	0.514	0.537
AR	JUN	9606.ENSP00000363822	9606.ENSP00000360266	0	0	0	0	0	0.454	0	0.535	0.736
AR	RUNX2	9606.ENSP00000363822	9606.ENSP00000360493	0	0	0	0	0	0.472	0	0.655	0.81
AR	MMP9	9606.ENSP00000363822	9606.ENSP00000361405	0	0	0	0	0	0	0	0.716	0.717
AR	IGF2	9606.ENSP00000363822	9606.ENSP00000391826	0	0	0	0	0	0	0	0.423	0.422
AR	CYP1A1	9606.ENSP00000363822	9606.ENSP00000369050	0	0	0	0	0	0.085	0	0.417	0.444
AR	HSD3B2	9606.ENSP00000363822	9606.ENSP00000445122	0	0	0	0	0	0	0	0.476	0.476
AR	IGFBP3	9606.ENSP00000363822	9606.ENSP00000370473	0	0	0	0	0	0.107	0	0.453	0.49

AR	CD44	9606.ENSP00000363822	9606.ENSP00000398632	0	0	0	0	0	0	0	0.51	0.51
AR	CDKN1A	9606.ENSP00000363822	9606.ENSP00000384849	0	0	0	0	0	0	0	0.51	0.51
AR	G6PD	9606.ENSP00000363822	9606.ENSP00000377192	0	0	0	0	0	0	0	0.534	0.534
AR	ESR1	9606.ENSP00000363822	9606.ENSP00000405330	0	0	0	0.656	0.062	0.379	0	0.936	0.587
AR	HIF1A	9606.ENSP00000363822	9606.ENSP00000437955	0	0	0	0	0	0.413	0	0.378	0.619
AR	GSTP1	9606.ENSP00000363822	9606.ENSP00000381607	0	0	0	0	0	0.064	0	0.65	0.659
AR	VEGFA	9606.ENSP00000363822	9606.ENSP00000478570	0	0	0	0	0	0	0	0.742	0.742
AR	RELA	9606.ENSP00000363822	9606.ENSP00000384273	0	0	0	0	0	0.393	0	0.625	0.763
AR	IL6	9606.ENSP00000363822	9606.ENSP00000385675	0	0	0	0	0	0	0	0.787	0.787

AR	MYC	9606.ENSP0000363822	9606.ENSP000004796 18	0	0	0	0	0	0	0	0.853	0.853
AR	MAPK8	9606.ENSP0000363822	9606.ENSP000003789 74	0	0	0	0	0	0	0.9	0.479	0.945
BAX	HMOX1	9606.ENSP0000293288	9606.ENSP000002161 17	0	0	0	0	0.063	0	0	0.399	0.412
BAX	CCND1	9606.ENSP0000293288	9606.ENSP000002275 07	0	0	0	0	0	0	0	0.478	0.478
BAX	CCNB1	9606.ENSP0000293288	9606.ENSP000002564 42	0	0	0	0	0.061	0	0	0.389	0.401
BAX	TP63	9606.ENSP0000293288	9606.ENSP000002647 31	0	0	0	0	0	0	0.9	0.056	0.901
BAX	MYC	9606.ENSP0000293288	9606.ENSP000004796 18	0	0	0	0	0	0.064	0	0.412	0.426
BAX	VEGFA	9606.ENSP0000293288	9606.ENSP000004785 70	0	0	0	0	0	0	0	0.429	0.429
BAX	CDKN1A	9606.ENSP0000293288	9606.ENSP000003848 49	0	0	0	0	0.069	0	0	0.444	0.46

BAX	JUN	9606.ENSP000002 93288	9606.ENSP0000036 0266	0	0	0	0	0	0	0	0.474	0.474
BAX	GSK3B	9606.ENSP000002 93288	9606.ENSP0000032 4806	0	0	0	0	0.059	0.384	0	0.215	0.505
BAX	CASP7	9606.ENSP000002 93288	9606.ENSP0000035 8327	0	0	0	0	0	0.416	0	0.484	0.686
BAX	CASP8	9606.ENSP000002 93288	9606.ENSP0000035 1273	0	0	0	0	0	0.416	0	0.656	0.791
BAX	CASP9	9606.ENSP000002 93288	9606.ENSP0000033 0237	0	0	0	0	0	0.416	0	0.712	0.825
BAX	CASP3	9606.ENSP000002 93288	9606.ENSP0000031 1032	0	0	0	0	0	0.416	0	0.793	0.874
BAX	MAPK8	9606.ENSP000002 93288	9606.ENSP0000037 8974	0	0	0	0	0	0.053	0.9	0.525	0.951
BAX	BCL2	9606.ENSP000002 93288	9606.ENSP0000038 1185	0	0	0	0.645	0	0.58	0.9	0.734	0.967
BAX	BCL2L1	9606.ENSP000002 93288	9606.ENSP0000030 2564	0	0	0	0.643	0.047	0.982	0.9	0.677	0.998

BCL2	MAPK1	9606.ENSP000003 81185	9606.ENSP00000215832	0	0	0	0	0	0.379	0.9	0.198	0.945
BCL2	CCND1	9606.ENSP000003 81185	9606.ENSP00000227507	0	0	0	0	0	0	0	0.433	0.433
BCL2	MAPK14	9606.ENSP000003 81185	9606.ENSP00000229795	0	0	0	0	0	0.379	0	0.089	0.41
BCL2	CTSD	9606.ENSP000003 81185	9606.ENSP00000236671	0	0	0	0	0	0	0.6	0.087	0.619
BCL2	SOD1	9606.ENSP000003 81185	9606.ENSP00000270142	0	0	0	0	0	0.379	0.8	0.117	0.88
BCL2	BCL2L1	9606.ENSP000003 81185	9606.ENSP00000302564	0	0	0	0.898	0	0.912	0.9	0.633	0.991
BCL2	CASP3	9606.ENSP000003 81185	9606.ENSP00000311032	0	0	0	0	0	0.461	0	0.578	0.763
BCL2	CASP9	9606.ENSP000003 81185	9606.ENSP00000330237	0	0	0	0	0	0.168	0	0.512	0.577
BCL2	CASP8	9606.ENSP000003 81185	9606.ENSP00000351273	0	0	0	0	0	0.993	0	0.43	0.996

BCL2	PARP1	9606.ENSP00000381185	9606.ENSP00000355759	0	0	0	0	0	0.379	0	0.249	0.514
BCL2	CHUK	9606.ENSP00000381185	9606.ENSP00000359424	0	0	0	0	0	0.379	0	0.113	0.425
BCL2	PPP3CA	9606.ENSP00000381185	9606.ENSP00000378323	0	0	0	0	0	0.472	0	0.085	0.496
BCL2	MAPK8	9606.ENSP00000381185	9606.ENSP00000378974	0	0	0	0	0	0.386	0.9	0.29	0.952
BCL2	IKBKB	9606.ENSP00000381185	9606.ENSP00000430684	0	0	0	0	0	0.379	0	0.129	0.435
BCL2	PRKCA	9606.ENSP00000381185	9606.ENSP00000408695	0	0	0	0	0	0.379	0	0.139	0.442
BCL2	HIF1A	9606.ENSP00000381185	9606.ENSP00000437955	0	0	0	0	0	0.357	0	0.25	0.497
BCL2	ESR1	9606.ENSP00000381185	9606.ENSP00000405330	0	0	0	0	0.069	0.064	0.9	0.244	0.925
BCL2	MYC	9606.ENSP00000381185	9606.ENSP00000479618	0	0	0	0	0	0.456	0.9	0.407	0.965

BCL2L1	MAPK1	9606.ENSP00000302564	9606.ENSP00000215832	0	0	0	0	0	0	0.9	0.786	0.977
BCL2L1	HMOX1	9606.ENSP00000302564	9606.ENSP00000216117	0	0	0	0	0	0	0	0.559	0.559
BCL2L1	NFKBIA	9606.ENSP00000302564	9606.ENSP00000216797	0	0	0	0	0.061	0.185	0	0.55	0.625
BCL2L1	MMP2	9606.ENSP00000302564	9606.ENSP00000219070	0	0	0	0	0	0	0	0.518	0.518
BCL2L1	CCL2	9606.ENSP00000302564	9606.ENSP00000225831	0	0	0	0	0	0	0	0.428	0.428
BCL2L1	IL2	9606.ENSP00000302564	9606.ENSP00000226730	0	0	0	0	0	0	0	0.63	0.63
BCL2L1	CCND1	9606.ENSP00000302564	9606.ENSP00000227507	0	0	0	0	0	0	0	0.83	0.83
BCL2L1	IFNG	9606.ENSP00000302564	9606.ENSP00000229135	0	0	0	0	0.052	0	0	0.399	0.406
BCL2L1	MAPK14	9606.ENSP00000302564	9606.ENSP00000229795	0	0	0	0	0	0.348	0	0.703	0.798

BCL2L1	IL4	9606.ENSP0000302564	9606.ENSP000231449	0	0	0	0	0	0	0	0.511	0.511
BCL2L1	NR3C1	9606.ENSP0000302564	9606.ENSP000231509	0	0	0	0	0	0.186	0	0.349	0.447
BCL2L1	CTSD	9606.ENSP0000302564	9606.ENSP000236671	0	0	0	0	0.076	0	0.6	0.294	0.716
BCL2L1	HSPB1	9606.ENSP0000302564	9606.ENSP000248553	0	0	0	0	0.061	0	0	0.491	0.501
BCL2L1	CCNB1	9606.ENSP0000302564	9606.ENSP000256442	0	0	0	0	0.061	0	0	0.661	0.668
BCL2L1	IL1B	9606.ENSP0000302564	9606.ENSP000263341	0	0	0	0	0.053	0	0	0.43	0.437
BCL2L1	KDR	9606.ENSP0000302564	9606.ENSP000263923	0	0	0	0	0	0	0	0.473	0.473
BCL2L1	ICAM1	9606.ENSP0000302564	9606.ENSP000264832	0	0	0	0	0	0	0	0.478	0.478
BCL2L1	EGF	9606.ENSP0000302564	9606.ENSP000265171	0	0	0	0	0	0	0	0.754	0.754

BCL2L1	ERBB2	9606.ENSP0000302564	9606.ENSP00000269571	0	0	0	0	0	0	0	0.594	0.594
BCL2L1	SOD1	9606.ENSP0000302564	9606.ENSP00000270142	0	0	0	0	0	0	0	0.403	0.403
BCL2L1	CCNA2	9606.ENSP0000302564	9606.ENSP00000274026	0	0	0	0	0	0	0	0.58	0.58
BCL2L1	EGFR	9606.ENSP0000302564	9606.ENSP00000275493	0	0	0	0	0	0	0	0.676	0.676
BCL2L1	BIRC5	9606.ENSP0000302564	9606.ENSP00000301633	0	0	0	0	0	0.083	0	0.522	0.543
BCL2L1	MET	9606.ENSP0000302564	9606.ENSP00000317272	0	0	0	0	0	0	0	0.452	0.452
BCL2L1	IKBKB	9606.ENSP0000302564	9606.ENSP00000430684	0	0	0	0	0	0	0	0.458	0.457
BCL2L1	HIF1A	9606.ENSP0000302564	9606.ENSP00000437955	0	0	0	0	0	0.127	0	0.43	0.481
BCL2L1	CD40LG	9606.ENSP0000302564	9606.ENSP00000359663	0	0	0	0	0	0	0	0.534	0.534

BCL2L1	FOS	9606.ENSP00000302564	9606.ENSP00000306245	0	0	0	0	0.061	0	0	0.531	0.54
BCL2L1	ESR1	9606.ENSP00000302564	9606.ENSP00000405330	0	0	0	0	0	0.064	0	0.531	0.542
BCL2L1	IL10	9606.ENSP00000302564	9606.ENSP00000412237	0	0	0	0	0	0	0	0.557	0.557
BCL2L1	CD44	9606.ENSP00000302564	9606.ENSP00000398632	0	0	0	0	0	0	0	0.557	0.557
BCL2L1	PTGS2	9606.ENSP00000302564	9606.ENSP00000356438	0	0	0	0	0	0	0	0.58	0.58
BCL2L1	GSK3B	9606.ENSP00000302564	9606.ENSP00000324806	0	0	0	0	0	0.354	0	0.409	0.602
BCL2L1	PARP1	9606.ENSP00000302564	9606.ENSP00000355759	0	0	0	0	0	0	0	0.611	0.611
BCL2L1	CTNNB1	9606.ENSP00000302564	9606.ENSP00000344456	0	0	0	0	0.046	0.336	0	0.473	0.637
BCL2L1	MMP9	9606.ENSP00000302564	9606.ENSP00000361405	0	0	0	0	0	0	0	0.642	0.642

BCL2L1	CDKN1A	9606.ENSP000003O2564	9606.ENSP000003848 49	0	0	0	0	0.069	0	0	0.659	0.669
BCL2L1	VEGFA	9606.ENSP00000302564	9606.ENSP000004785 70	0	0	0	0	0	0	0	0.678	0.678
BCL2L1	JUN	9606.ENSP00000302564	9606.ENSP000003602 66	0	0	0	0	0.062	0	0	0.674	0.681
BCL2L1	PGR	9606.ENSP00000302564	9606.ENSP000003251 20	0	0	0	0	0	0.263	0	0.593	0.687
BCL2L1	CXCL8	9606.ENSP00000302564	9606.ENSP000003065 12	0	0	0	0	0	0	0	0.719	0.719
BCL2L1	STAT1	9606.ENSP00000302564	9606.ENSP000003543 94	0	0	0	0	0	0.084	0	0.725	0.737
BCL2L1	RELA	9606.ENSP00000302564	9606.ENSP000003842 73	0	0	0	0	0.082	0.336	0	0.63	0.754
BCL2L1	CASP7	9606.ENSP00000302564	9606.ENSP000003583 27	0	0	0	0	0	0.168	0	0.765	0.796
BCL2L1	MYC	9606.ENSP00000302564	9606.ENSP000004796 18	0	0	0	0	0	0.128	0	0.78	0.8

BCL2L1	IL6	9606.ENSP00000302564	9606.ENSP00000385675	0	0	0	0	0.049	0	0	0.797	0.8
BCL2L1	CASP3	9606.ENSP00000302564	9606.ENSP00000311032	0	0	0	0	0	0.168	0	0.927	0.937
BCL2L1	CASP9	9606.ENSP00000302564	9606.ENSP00000330237	0	0	0	0	0.055	0.729	0	0.897	0.971
BCL2L1	MAPK8	9606.ENSP00000302564	9606.ENSP00000378974	0	0	0	0	0	0.519	0.8	0.869	0.986
BCL2L1	CASP8	9606.ENSP00000302564	9606.ENSP00000351273	0	0	0	0	0	0.993	0	0.883	0.999
BIRC5	CCND1	9606.ENSP00000301633	9606.ENSP00000227507	0	0	0	0	0.085	0	0	0.563	0.583
BIRC5	CCNB1	9606.ENSP00000301633	9606.ENSP00000256442	0	0	0	0	0.888	0.185	0.9	0.651	0.996
BIRC5	ERBB2	9606.ENSP00000301633	9606.ENSP00000269571	0	0	0	0	0	0	0	0.475	0.475
BIRC5	CCNA2	9606.ENSP00000301633	9606.ENSP00000274026	0	0	0	0	0.921	0.157	0	0.522	0.965

BIRC5	EGFR	9606.ENSP0000301633	9606.ENSP000275493	0	0	0	0	0	0	0	0.474	0.474
BIRC5	E2F2	9606.ENSP0000301633	9606.ENSP000355249	0	0	0	0	0.178	0.084	0	0.293	0.421
BIRC5	ESR1	9606.ENSP0000301633	9606.ENSP000405330	0	0	0	0	0	0	0	0.44	0.44
BIRC5	CDKN1A	9606.ENSP0000301633	9606.ENSP000384849	0	0	0	0	0	0	0	0.465	0.465
BIRC5	CASP8	9606.ENSP0000301633	9606.ENSP000351273	0	0	0	0	0.06	0.181	0	0.428	0.521
BIRC5	E2F1	9606.ENSP0000301633	9606.ENSP000345571	0	0	0	0	0.306	0.084	0	0.36	0.558
BIRC5	MYC	9606.ENSP0000301633	9606.ENSP000479618	0	0	0	0	0.102	0.084	0	0.535	0.585
BIRC5	CTNNB1	9606.ENSP0000301633	9606.ENSP000344456	0	0	0	0	0.054	0.336	0	0.398	0.588
BIRC5	CASP7	9606.ENSP0000301633	9606.ENSP000358327	0	0	0	0	0.048	0.469	0	0.363	0.65

BIRC5	CASP3	9606.ENSP000003 01633	9606.ENSP0000031 1032	0	0	0	0	0.061	0.469	0	0.507	0.733
BIRC5	CASP9	9606.ENSP000003 01633	9606.ENSP0000033 0237	0	0	0	0	0	0.588	0	0.455	0.766
BMPR2	SLC6A4	9606.ENSP000003 63708	9606.ENSP0000026 1707	0	0	0	0	0.062	0	0	0.411	0.424
BMPR2	NOS3	9606.ENSP000003 63708	9606.ENSP0000029 7494	0	0	0	0	0	0.065	0	0.404	0.418
BMPR2	CAV1	9606.ENSP000003 63708	9606.ENSP0000033 9191	0	0	0	0	0	0.389	0	0.72	0.822
BMPR2	RUNX2	9606.ENSP000003 63708	9606.ENSP0000036 0493	0	0	0	0	0	0	0	0.494	0.494
BMPR2	NOX1	9606.ENSP000003 63708	9606.ENSP0000036 2057	0	0	0	0	0	0.079	0	0.426	0.448
BMPR2	VEGFA	9606.ENSP000003 63708	9606.ENSP0000047 8570	0	0	0	0	0	0	0	0.426	0.426
BMPR2	ESR1	9606.ENSP000003 63708	9606.ENSP0000040 5330	0	0	0	0	0	0.085	0	0.543	0.564

BMPR2	IL6	9606.ENSP0000363708	9606.ENSP00000385675	0	0	0	0	0	0	0	0.604	0.605
CASP3	MAPK1	9606.ENSP0000311032	9606.ENSP00000215832	0	0	0	0	0.048	0.261	0	0.757	0.814
CASP3	HMOX1	9606.ENSP0000311032	9606.ENSP00000216117	0	0	0	0	0	0	0	0.726	0.726
CASP3	NFKBIA	9606.ENSP0000311032	9606.ENSP00000216797	0	0	0	0	0	0.178	0	0.564	0.626
CASP3	MMP2	9606.ENSP0000311032	9606.ENSP00000219070	0	0	0	0	0	0	0	0.669	0.669
CASP3	SERPINE1	9606.ENSP0000311032	9606.ENSP00000223095	0	0	0	0	0	0	0	0.455	0.455
CASP3	MPO	9606.ENSP0000311032	9606.ENSP00000225275	0	0	0	0	0	0	0	0.699	0.699
CASP3	CCL2	9606.ENSP0000311032	9606.ENSP00000225831	0	0	0	0	0.05	0	0	0.63	0.633
CASP3	IL2	9606.ENSP0000311032	9606.ENSP00000226730	0	0	0	0	0	0	0	0.575	0.575

CASP3	CCND1	9606.ENSP00000311032	9606.ENSP00000227507	0	0	0	0	0.061	0	0	0.851	0.854
CASP3	IFNG	9606.ENSP00000311032	9606.ENSP00000229135	0	0	0	0	0	0	0	0.538	0.538
CASP3	MAPK14	9606.ENSP00000311032	9606.ENSP00000229795	0	0	0	0	0.061	0.521	0.9	0.793	0.989
CASP3	IL4	9606.ENSP00000311032	9606.ENSP00000231449	0	0	0	0	0	0	0	0.544	0.544
CASP3	NR3C1	9606.ENSP00000311032	9606.ENSP00000231509	0	0	0	0	0	0.058	0	0.415	0.425
CASP3	ODC1	9606.ENSP00000311032	9606.ENSP00000234111	0	0	0	0	0.061	0	0	0.4	0.412
CASP3	CTSD	9606.ENSP00000311032	9606.ENSP00000236671	0	0	0	0	0	0	0	0.556	0.556
CASP3	HSPB1	9606.ENSP00000311032	9606.ENSP00000248553	0	0	0	0	0	0.437	0	0.793	0.879
CASP3	CRP	9606.ENSP00000311032	9606.ENSP00000255030	0	0	0	0	0	0	0	0.471	0.471

CASP3	CCNB1	9606.ENSP00000311032	9606.ENSP00000256442	0	0	0	0	0.084	0	0	0.753	0.765
CASP3	IL1B	9606.ENSP00000311032	9606.ENSP00000263341	0	0	0	0	0	0.074	0	0.824	0.831
CASP3	KDR	9606.ENSP00000311032	9606.ENSP00000263923	0	0	0	0	0	0	0	0.594	0.594
CASP3	ICAM1	9606.ENSP00000311032	9606.ENSP00000264832	0	0	0	0	0	0	0	0.62	0.62
CASP3	EGF	9606.ENSP00000311032	9606.ENSP00000265171	0	0	0	0	0	0	0	0.698	0.698
CASP3	ERBB2	9606.ENSP00000311032	9606.ENSP00000269571	0	0	0	0	0	0.05	0	0.691	0.694
CASP3	SOD1	9606.ENSP00000311032	9606.ENSP00000270142	0	0	0	0	0	0.092	0	0.63	0.65
CASP3	CCNA2	9606.ENSP00000311032	9606.ENSP00000274026	0	0	0	0	0.084	0	0	0.67	0.684
CASP3	EGFR	9606.ENSP00000311032	9606.ENSP00000275493	0	0	0	0	0	0.05	0	0.748	0.751

CASP3	MMP10	9606.ENSP0000311032	9606.ENSP00000279441	0	0	0	0	0	0	0	0.435	0.435
CASP3	GJA1	9606.ENSP0000311032	9606.ENSP00000282561	0	0	0	0	0	0	0	0.501	0.501
CASP3	PPARG	9606.ENSP0000311032	9606.ENSP00000287820	0	0	0	0	0	0.058	0	0.571	0.578
CASP3	VCAM1	9606.ENSP0000311032	9606.ENSP00000294728	0	0	0	0	0.053	0	0	0.522	0.528
CASP3	NOS3	9606.ENSP0000311032	9606.ENSP00000297494	0	0	0	0	0	0	0	0.793	0.793
CASP3	MMP3	9606.ENSP0000311032	9606.ENSP00000299855	0	0	0	0	0	0	0	0.657	0.657
CASP3	CXCL10	9606.ENSP0000311032	9606.ENSP00000305651	0	0	0	0	0	0	0	0.434	0.434
CASP3	FOS	9606.ENSP0000311032	9606.ENSP00000306245	0	0	0	0	0	0	0	0.585	0.585
CASP3	CXCL8	9606.ENSP0000311032	9606.ENSP00000306512	0	0	0	0	0	0	0	0.649	0.649

CASP3	IGFBP3	9606.ENSP000003110 32	9606.ENSP000000370473	0	0	0	0	0	0	0	0.4	0.4
CASP3	E2F1	9606.ENSP000003110 32	9606.ENSP000000345571	0	0	0	0	0	0	0	0.408	0.408
CASP3	IGF2	9606.ENSP000003110 32	9606.ENSP000000391826	0	0	0	0	0	0	0	0.42	0.42
CASP3	CD40LG	9606.ENSP000003110 32	9606.ENSP000000359663	0	0	0	0	0	0.055	0	0.418	0.426
CASP3	SELE	9606.ENSP000003110 32	9606.ENSP000000331736	0	0	0	0	0	0	0	0.428	0.428
CASP3	RUNX2	9606.ENSP000003110 32	9606.ENSP000000360493	0	0	0	0	0	0	0	0.448	0.448
CASP3	ESR2	9606.ENSP000003110 32	9606.ENSP000000343925	0	0	0	0	0	0.058	0	0.443	0.452
CASP3	MMP1	9606.ENSP000003110 32	9606.ENSP000000322788	0	0	0	0	0	0	0	0.453	0.453
CASP3	CAV1	9606.ENSP000003110 32	9606.ENSP000000339191	0	0	0	0	0	0	0	0.454	0.454

CASP3	PGR	9606.ENSP00000311032	9606.ENSP000003255120	0	0	0	0	0	0.058	0	0.461	0.471
CASP3	PPP3CA	9606.ENSP00000311032	9606.ENSP000003788323	0	0	0	0	0	0.379	0	0.185	0.472
CASP3	NOX1	9606.ENSP00000311032	9606.ENSP000003622057	0	0	0	0	0	0	0	0.483	0.483
CASP3	IKBKB	9606.ENSP00000311032	9606.ENSP000004300684	0	0	0	0	0	0.071	0	0.474	0.49
CASP3	NOS2	9606.ENSP00000311032	9606.ENSP000003277251	0	0	0	0	0	0	0	0.533	0.533
CASP3	RELA	9606.ENSP00000311032	9606.ENSP000003844273	0	0	0	0	0	0	0	0.557	0.557
CASP3	HIF1A	9606.ENSP00000311032	9606.ENSP000004377955	0	0	0	0	0	0	0	0.559	0.559
CASP3	CD44	9606.ENSP00000311032	9606.ENSP000003988632	0	0	0	0	0	0	0	0.598	0.598
CASP3	SPP1	9606.ENSP00000311032	9606.ENSP000003788517	0	0	0	0	0	0.379	0	0.42	0.625

CASP3	GSK3B	9606.ENSP000003 11032	9606.ENSP0000032 4806	0	0	0	0	0.059	0.097	0	0.605	0.636
CASP3	ESR1	9606.ENSP000003 11032	9606.ENSP0000040 5330	0	0	0	0	0	0.058	0	0.659	0.665
CASP3	IL10	9606.ENSP000003 11032	9606.ENSP0000041 2237	0	0	0	0	0	0	0	0.669	0.669
CASP3	PTGS2	9606.ENSP000003 11032	9606.ENSP0000035 6438	0	0	0	0	0	0	0	0.685	0.685
CASP3	STAT1	9606.ENSP000003 11032	9606.ENSP0000035 4394	0	0	0	0	0.062	0.332	0	0.59	0.72
CASP3	NFE2L2	9606.ENSP000003 11032	9606.ENSP0000038 0252	0	0	0	0	0	0.379	0	0.571	0.722
CASP3	MMP9	9606.ENSP000003 11032	9606.ENSP0000036 1405	0	0	0	0	0.063	0	0	0.716	0.723
CASP3	VEGFA	9606.ENSP000003 11032	9606.ENSP0000047 8570	0	0	0	0	0	0	0	0.737	0.737
CASP3	MET	9606.ENSP000003 11032	9606.ENSP0000031 7272	0	0	0	0	0	0.524	0	0.524	0.764

CASP3	IL6	9606.ENSP00000311032	9606.ENSP00000385675	0	0	0	0	0	0	0	0.771	0.771
CASP3	MYC	9606.ENSP00000311032	9606.ENSP00000479618	0	0	0	0	0.061	0	0	0.77	0.775
CASP3	PRKCA	9606.ENSP00000311032	9606.ENSP00000408695	0	0	0	0	0	0	0.8	0.398	0.874
CASP3	JUN	9606.ENSP00000311032	9606.ENSP00000360266	0	0	0	0	0	0.069	0	0.87	0.874
CASP3	MAPK8	9606.ENSP00000311032	9606.ENSP00000378974	0	0	0	0	0.055	0.294	0	0.863	0.901
CASP3	CASP7	9606.ENSP00000311032	9606.ENSP00000358327	0	0	0	0.942	0.062	0.332	0.9	0.897	0.935
CASP3	SREBF1	9606.ENSP00000311032	9606.ENSP00000348069	0	0	0	0	0	0	0.9	0.385	0.935
CASP3	CASP9	9606.ENSP00000311032	9606.ENSP00000330237	0	0	0	0.833	0.061	0.682	0.9	0.971	0.972
CASP3	CDKN1A	9606.ENSP00000311032	9606.ENSP00000384849	0	0	0	0	0	0.682	0.8	0.718	0.98

CASP3	CTNNB1	9606.ENSP0000311032	9606.ENSP00000344456	0	0	0	0	0.062	0.542	0.9	0.648	0.982
CASP3	PARP1	9606.ENSP0000311032	9606.ENSP00000355759	0	0	0	0	0.062	0.472	0.9	0.839	0.99
CASP3	CASP8	9606.ENSP0000311032	9606.ENSP00000351273	0	0	0	0.835	0.061	0.993	0.9	0.978	0.999
CASP7	MAPK1	9606.ENSP0000358327	9606.ENSP00000215832	0	0	0	0	0	0.261	0	0.549	0.652
CASP7	NFKBIA	9606.ENSP0000358327	9606.ENSP00000216797	0	0	0	0	0	0.178	0	0.363	0.454
CASP7	CCND1	9606.ENSP0000358327	9606.ENSP00000227507	0	0	0	0	0	0	0	0.572	0.572
CASP7	MAPK14	9606.ENSP0000358327	9606.ENSP00000229795	0	0	0	0	0	0.261	0	0.413	0.548
CASP7	CCNB1	9606.ENSP0000358327	9606.ENSP00000256442	0	0	0	0	0	0	0	0.551	0.551
CASP7	ERBB2	9606.ENSP0000358327	9606.ENSP00000269571	0	0	0	0	0	0.063	0	0.431	0.444

CASP7	CCNA2	9606.<br>ENSP0<br>00003<br>58327	9606.E<br>NSP00<br>00027<br>4026	0	0	0	0	0	0	0	0.426	0.426
CASP7	EGFR	9606.<br>ENSP0<br>00003<br>58327	9606.E<br>NSP00<br>00027<br>5493	0	0	0	0	0	0.063	0	0.463	0.475
CASP7	CASP9	9606.<br>ENSP0<br>00003<br>58327	9606.E<br>NSP00<br>00033<br>0237	0	0	0	0.71	0	0.332	0.9	0.894	0.948
CASP7	E2F1	9606.<br>ENSP0<br>00003<br>58327	9606.E<br>NSP00<br>00034<br>5571	0	0	0	0	0	0.336	0	0.255	0.484
CASP7	CASP8	9606.<br>ENSP0<br>00003<br>58327	9606.E<br>NSP00<br>00035<br>1273	0	0	0	0.753	0.063	0.993	0.9	0.874	0.999
CASP7	STAT1	9606.<br>ENSP0<br>00003<br>58327	9606.E<br>NSP00<br>00035<br>4394	0	0	0	0	0.082	0.332	0	0.295	0.53
CASP7	PARP1	9606.<br>ENSP0<br>00003<br>58327	9606.E<br>NSP00<br>00035<br>5759	0	0	0	0	0.052	0.435	0.9	0.69	0.981
CASP7	CDKN1A	9606.<br>ENSP0<br>00003<br>58327	9606.E<br>NSP00<br>00038<br>4849	0	0	0	0	0.067	0	0	0.425	0.44
CASP7	MYC	9606.<br>ENSP0<br>00003<br>58327	9606.E<br>NSP00<br>00047<br>9618	0	0	0	0	0	0	0	0.442	0.442

CASP7	JUN	9606.ENSP0000358327	9606.ENSP00000360266	0	0	0	0	0	0.069	0	0.478	0.493
CASP7	MAPK8	9606.ENSP0000358327	9606.ENSP00000378974	0	0	0	0	0	0	0	0.689	0.689
CASP8	MAPK1	9606.ENSP0000351273	9606.ENSP00000215832	0	0	0	0	0	0.482	0.9	0.778	0.987
CASP8	HMOX1	9606.ENSP0000351273	9606.ENSP00000216117	0	0	0	0	0	0	0	0.43	0.43
CASP8	NFKBIA	9606.ENSP0000351273	9606.ENSP00000216797	0	0	0	0	0.061	0.178	0	0.51	0.588
CASP8	MMP2	9606.ENSP0000351273	9606.ENSP00000219070	0	0	0	0	0.061	0	0	0.445	0.456
CASP8	CCL2	9606.ENSP0000351273	9606.ENSP00000225831	0	0	0	0	0.061	0	0	0.44	0.451
CASP8	COL1A1	9606.ENSP0000351273	9606.ENSP00000225964	0	0	0	0	0	0	0.9	0.185	0.915
CASP8	IL2	9606.ENSP0000351273	9606.ENSP00000226730	0	0	0	0	0	0	0	0.532	0.532

CASP8	CCND1	9606.ENSP00000351273	9606.ENSP00000227507	0	0	0	0	0	0	0	0.66	0.66
CASP8	IFNG	9606.ENSP00000351273	9606.ENSP00000229135	0	0	0	0	0.061	0	0	0.687	0.694
CASP8	MAPK14	9606.ENSP00000351273	9606.ENSP00000229795	0	0	0	0	0	0.521	0	0.667	0.833
CASP8	IL4	9606.ENSP00000351273	9606.ENSP00000231449	0	0	0	0	0	0	0	0.42	0.42
CASP8	CTSD	9606.ENSP00000351273	9606.ENSP00000236671	0	0	0	0	0.052	0	0	0.42	0.426
CASP8	IRF1	9606.ENSP00000351273	9606.ENSP00000245414	0	0	0	0	0.129	0	0	0.637	0.67
CASP8	HSPB1	9606.ENSP00000351273	9606.ENSP00000248553	0	0	0	0	0	0.064	0	0.459	0.472
CASP8	CCNB1	9606.ENSP00000351273	9606.ENSP00000256442	0	0	0	0	0	0	0	0.595	0.595
CASP8	IL1B	9606.ENSP00000351273	9606.ENSP00000263341	0	0	0	0	0.063	0.268	0.9	0.568	0.966

CASP8	ICAM1	9606.ENSP0000351273	9606.ENSP00000264832	0	0	0	0	0.063	0	0	0.416	0.429
CASP8	EGF	9606.ENSP0000351273	9606.ENSP00000265171	0	0	0	0	0	0	0	0.519	0.519
CASP8	ERBB2	9606.ENSP0000351273	9606.ENSP00000269571	0	0	0	0	0	0.05	0	0.526	0.53
CASP8	SOD1	9606.ENSP0000351273	9606.ENSP00000270142	0	0	0	0	0	0.092	0	0.388	0.421
CASP8	CCNA2	9606.ENSP0000351273	9606.ENSP00000274026	0	0	0	0	0	0	0	0.512	0.512
CASP8	EGFR	9606.ENSP0000351273	9606.ENSP00000275493	0	0	0	0	0	0.05	0	0.733	0.736
CASP8	COL3A1	9606.ENSP0000351273	9606.ENSP00000304408	0	0	0	0	0	0	0.9	0.216	0.918
CASP8	FOS	9606.ENSP0000351273	9606.ENSP00000306245	0	0	0	0	0.058	0	0	0.44	0.449
CASP8	CXCL8	9606.ENSP0000351273	9606.ENSP00000306512	0	0	0	0	0.067	0	0	0.53	0.543

CASP8	CASP9	9606.ENSP0000351273	9606.ENSP00000330237	0	0	0	0.707	0	0.993	0	0.956	0.994
CASP8	CTNNB1	9606.ENSP0000351273	9606.ENSP00000344456	0	0	0	0	0	0.387	0	0.404	0.619
CASP8	CD40LG	9606.ENSP0000351273	9606.ENSP00000359663	0	0	0	0	0.084	0.055	0	0.399	0.434
CASP8	CD44	9606.ENSP0000351273	9606.ENSP00000398632	0	0	0	0	0.063	0	0	0.421	0.434
CASP8	STAT1	9606.ENSP0000351273	9606.ENSP00000354394	0	0	0	0	0.062	0	0	0.485	0.496
CASP8	ESR1	9606.ENSP0000351273	9606.ENSP00000405330	0	0	0	0	0	0.058	0	0.501	0.509
CASP8	MMP9	9606.ENSP0000351273	9606.ENSP00000361405	0	0	0	0	0	0	0	0.516	0.516
CASP8	SPP1	9606.ENSP0000351273	9606.ENSP00000378517	0	0	0	0	0	0.379	0	0.255	0.517
CASP8	PTGS2	9606.ENSP0000351273	9606.ENSP00000356438	0	0	0	0	0.057	0	0	0.509	0.518

CASP8	IL10	9606.ENSP000003 51273	9606.ENSP00000412237	0	0	0	0	0	0	0	0.523	0.523
CASP8	VEGFA	9606.ENSP000003 51273	9606.ENSP00000478570	0	0	0	0	0	0	0	0.559	0.559
CASP8	CDKN1A	9606.ENSP000003 51273	9606.ENSP00000384849	0	0	0	0	0	0	0	0.569	0.569
CASP8	MYC	9606.ENSP000003 51273	9606.ENSP00000479618	0	0	0	0	0.052	0	0	0.635	0.639
CASP8	IL6	9606.ENSP000003 51273	9606.ENSP00000385675	0	0	0	0	0.061	0	0	0.642	0.65
CASP8	JUN	9606.ENSP000003 51273	9606.ENSP00000360266	0	0	0	0	0	0.069	0	0.683	0.692
CASP8	PARP1	9606.ENSP000003 51273	9606.ENSP00000355759	0	0	0	0	0	0.394	0	0.715	0.82
CASP8	MAPK8	9606.ENSP000003 51273	9606.ENSP00000378974	0	0	0	0	0	0.48	0	0.752	0.865
CASP8	RELA	9606.ENSP000003 51273	9606.ENSP00000384273	0	0	0	0	0.061	0	0.9	0.542	0.953

CASP8	CHUK	9606.<br>ENSP0<br>00003<br>51273	9606.E<br>NSP00<br>00035<br>9424	0	0	0	0	0	0.374	0.9	0.43	0.961
CASP8	IKBKB	9606.<br>ENSP0<br>00003<br>51273	9606.E<br>NSP00<br>00043<br>0684	0	0	0	0	0.061	0.374	0.9	0.447	0.963
CASP9	MAPK1	9606.<br>ENSP0<br>00003<br>30237	9606.E<br>NSP00<br>00021<br>5832	0	0	0	0	0	0.455	0	0.674	0.815
CASP9	HMOX1	9606.<br>ENSP0<br>00003<br>30237	9606.E<br>NSP00<br>00021<br>6117	0	0	0	0	0	0	0	0.528	0.528
CASP9	NFKBIA	9606.<br>ENSP0<br>00003<br>30237	9606.E<br>NSP00<br>00021<br>6797	0	0	0	0	0.061	0.178	0	0.428	0.52
CASP9	MMP2	9606.<br>ENSP0<br>00003<br>30237	9606.E<br>NSP00<br>00021<br>9070	0	0	0	0	0	0	0	0.475	0.475
CASP9	IL2	9606.<br>ENSP0<br>00003<br>30237	9606.E<br>NSP00<br>00022<br>6730	0	0	0	0	0	0	0	0.414	0.414
CASP9	CCND1	9606.<br>ENSP0<br>00003<br>30237	9606.E<br>NSP00<br>00022<br>7507	0	0	0	0	0	0	0	0.737	0.737
CASP9	MAPK14	9606.<br>ENSP0<br>00003<br>30237	9606.E<br>NSP00<br>00022<br>9795	0	0	0	0	0	0.261	0	0.561	0.662

CASP9	HSPB1	9606.<br>ENSP0<br>00003<br>30237	9606.E<br>NSP00<br>00024<br>8553	0	0	0	0	0	0.064	0	0.553	0.564
CASP9	CCNB1	9606.<br>ENSP0<br>00003<br>30237	9606.E<br>NSP00<br>00025<br>6442	0	0	0	0	0	0	0	0.686	0.686
CASP9	IL1B	9606.<br>ENSP0<br>00003<br>30237	9606.E<br>NSP00<br>00026<br>3341	0	0	0	0	0	0.074	0	0.44	0.459
CASP9	EGF	9606.<br>ENSP0<br>00003<br>30237	9606.E<br>NSP00<br>00026<br>5171	0	0	0	0	0	0	0	0.51	0.51
CASP9	ERBB2	9606.<br>ENSP0<br>00003<br>30237	9606.E<br>NSP00<br>00026<br>9571	0	0	0	0	0	0.05	0	0.538	0.542
CASP9	SOD1	9606.<br>ENSP0<br>00003<br>30237	9606.E<br>NSP00<br>00027<br>0142	0	0	0	0	0	0.092	0	0.426	0.456
CASP9	CCNA2	9606.<br>ENSP0<br>00003<br>30237	9606.E<br>NSP00<br>00027<br>4026	0	0	0	0	0	0	0	0.581	0.581
CASP9	EGFR	9606.<br>ENSP0<br>00003<br>30237	9606.E<br>NSP00<br>00027<br>5493	0	0	0	0	0	0.05	0	0.601	0.605
CASP9	NOS3	9606.<br>ENSP0<br>00003<br>30237	9606.E<br>NSP00<br>00029<br>7494	0	0	0	0	0	0	0	0.433	0.433

CASP9	FOS	9606.ENSP000003 30237	9606.ENSP0000030 6245	0	0	0	0	0	0	0	0.414	0.414
CASP9	CXCL8	9606.ENSP000003 30237	9606.ENSP0000030 6512	0	0	0	0	0	0	0	0.443	0.443
CASP9	GSK3B	9606.ENSP000003 30237	9606.ENSP0000032 4806	0	0	0	0	0.052	0.097	0	0.597	0.625
CASP9	STAT1	9606.ENSP000003 30237	9606.ENSP0000035 4394	0	0	0	0	0	0	0	0.408	0.408
CASP9	RELA	9606.ENSP000003 30237	9606.ENSP0000038 4273	0	0	0	0	0	0	0	0.419	0.418
CASP9	CTNNB1	9606.ENSP000003 30237	9606.ENSP0000034 4456	0	0	0	0	0	0.054	0	0.418	0.426
CASP9	IL10	9606.ENSP000003 30237	9606.ENSP0000041 2237	0	0	0	0	0	0	0	0.427	0.427
CASP9	ESR1	9606.ENSP000003 30237	9606.ENSP0000040 5330	0	0	0	0	0	0.058	0	0.483	0.492
CASP9	PTGS2	9606.ENSP000003 30237	9606.ENSP0000035 6438	0	0	0	0	0	0	0	0.547	0.547

CASP9	MMP9	9606.<br>ENSP0<br>00003<br>30237	9606.E<br>NSP00<br>00036<br>1405	0	0	0	0	0	0	0	0.554	0.554
CASP9	IL6	9606.<br>ENSP0<br>00003<br>30237	9606.E<br>NSP00<br>00038<br>5675	0	0	0	0	0	0	0	0.578	0.578
CASP9	VEGFA	9606.<br>ENSP0<br>00003<br>30237	9606.E<br>NSP00<br>00047<br>8570	0	0	0	0	0	0	0	0.581	0.581
CASP9	CDKN1A	9606.<br>ENSP0<br>00003<br>30237	9606.E<br>NSP00<br>00038<br>4849	0	0	0	0	0	0	0	0.634	0.634
CASP9	MYC	9606.<br>ENSP0<br>00003<br>30237	9606.E<br>NSP00<br>00047<br>9618	0	0	0	0	0	0	0	0.649	0.649
CASP9	MAPK8	9606.<br>ENSP0<br>00003<br>30237	9606.E<br>NSP00<br>00037<br>8974	0	0	0	0	0	0	0	0.741	0.741
CASP9	JUN	9606.<br>ENSP0<br>00003<br>30237	9606.E<br>NSP00<br>00036<br>0266	0	0	0	0	0	0.472	0	0.671	0.818
CASP9	PARP1	9606.<br>ENSP0<br>00003<br>30237	9606.E<br>NSP00<br>00035<br>5759	0	0	0	0	0.061	0.126	0.8	0.743	0.952
CAV1	MAPK1	9606.<br>ENSP0<br>00003<br>39191	9606.E<br>NSP00<br>00021<br>5832	0	0	0	0	0	0.379	0	0.477	0.661

CAV1	HMOX1	9606.ENSP00000339191	9606.ENSP00000216117	0	0	0	0	0.055	0	0	0.633	0.638
CAV1	MMP2	9606.ENSP00000339191	9606.ENSP00000219070	0	0	0	0	0.23	0	0	0.473	0.576
CAV1	SERPINE1	9606.ENSP00000339191	9606.ENSP00000223095	0	0	0	0	0.314	0	0	0.422	0.586
CAV1	CCL2	9606.ENSP00000339191	9606.ENSP00000225831	0	0	0	0	0.085	0	0	0.429	0.455
CAV1	COL1A1	9606.ENSP00000339191	9606.ENSP00000225964	0	0	0	0	0.213	0	0	0.284	0.412
CAV1	CCND1	9606.ENSP00000339191	9606.ENSP00000227507	0	0	0	0	0.214	0	0	0.697	0.752
CAV1	NR3C1	9606.ENSP00000339191	9606.ENSP00000231509	0	0	0	0	0.061	0.4	0	0.215	0.519
CAV1	IL1B	9606.ENSP00000339191	9606.ENSP00000263341	0	0	0	0	0.055	0	0	0.594	0.6
CAV1	KDR	9606.ENSP00000339191	9606.ENSP00000263923	0	0	0	0	0.07	0.379	0	0.671	0.794

CAV1	ICAM1	9606.ENSP000003391 91	9606.ENSP00000264832	0	0	0	0	0	0	0	0.656	0.656
CAV1	EGF	9606.ENSP00000339191	9606.ENSP00000265171	0	0	0	0	0	0	0.9	0.746	0.973
CAV1	ERBB2	9606.ENSP00000339191	9606.ENSP00000269571	0	0	0	0	0.06	0.379	0	0.705	0.813
CAV1	EGFR	9606.ENSP00000339191	9606.ENSP00000275493	0	0	0	0	0.335	0.393	0.9	0.844	0.992
CAV1	GJA1	9606.ENSP00000339191	9606.ENSP00000282561	0	0	0	0	0.113	0.294	0	0.473	0.641
CAV1	PPARG	9606.ENSP00000339191	9606.ENSP00000287820	0	0	0	0	0.071	0.466	0	0.473	0.716
CAV1	VCAM1	9606.ENSP00000339191	9606.ENSP00000294728	0	0	0	0	0.068	0	0	0.442	0.458
CAV1	NOS3	9606.ENSP00000339191	9606.ENSP00000297494	0	0	0	0	0	0.379	0.9	0.915	0.994
CAV1	CXCL8	9606.ENSP00000339191	9606.ENSP00000306512	0	0	0	0	0	0	0	0.417	0.416

CAV1	MET	9606.ENSP000003 39191	9606.ENSP0000031 7272	0	0	0	0	0.214	0.057	0	0.657	0.723
CAV1	GSK3B	9606.ENSP000003 39191	9606.ENSP0000032 4806	0	0	0	0	0	0.057	0.9	0.282	0.926
CAV1	PGR	9606.ENSP000003 39191	9606.ENSP0000032 5120	0	0	0	0	0	0.074	0	0.381	0.402
CAV1	NOS2	9606.ENSP000003 39191	9606.ENSP0000032 7251	0	0	0	0	0	0.379	0	0.211	0.489
CAV1	ESR2	9606.ENSP000003 39191	9606.ENSP0000034 3925	0	0	0	0	0	0.074	0	0.398	0.418
CAV1	PLAU	9606.ENSP000003 39191	9606.ENSP0000036 1850	0	0	0	0	0.18	0	0	0.35	0.444
CAV1	PTGS1	9606.ENSP000003 39191	9606.ENSP0000035 4612	0	0	0	0	0.062	0.379	0	0.145	0.458
CAV1	MMP9	9606.ENSP000003 39191	9606.ENSP0000036 1405	0	0	0	0	0	0	0	0.493	0.493
CAV1	IL6	9606.ENSP000003 39191	9606.ENSP0000038 5675	0	0	0	0	0.088	0	0	0.477	0.503

CAV1	MYC	9606.ENSP0000339191	9606.ENSP00000479618	0	0	0	0	0.076	0	0	0.499	0.517
CAV1	CD44	9606.ENSP0000339191	9606.ENSP00000398632	0	0	0	0	0.114	0.313	0	0.448	0.634
CAV1	PRKCA	9606.ENSP0000339191	9606.ENSP00000408695	0	0	0	0	0	0	0	0.635	0.635
CAV1	SREBF1	9606.ENSP0000339191	9606.ENSP00000348069	0	0	0	0	0	0	0	0.643	0.643
CAV1	VCP	9606.ENSP0000339191	9606.ENSP00000351777	0	0	0	0	0	0.379	0	0.483	0.665
CAV1	MAPK8	9606.ENSP0000339191	9606.ENSP00000378974	0	0	0	0	0	0	0	0.671	0.671
CAV1	IGFBP3	9606.ENSP0000339191	9606.ENSP00000370473	0	0	0	0	0.3	0.379	0	0.348	0.691
CAV1	CTNNB1	9606.ENSP0000339191	9606.ENSP00000344456	0	0	0	0	0	0.379	0	0.655	0.776
CAV1	VEGFA	9606.ENSP0000339191	9606.ENSP00000478570	0	0	0	0	0.076	0	0	0.772	0.78

CAV1	PTGS2	9606.ENSP0000339191	9606.ENSP00000356438	0	0	0	0	0.084	0.379	0	0.669	0.795
CAV1	ESR1	9606.ENSP0000339191	9606.ENSP00000405330	0	0	0	0	0	0.4	0	0.734	0.834
CCL2	MAPK1	9606.ENSP0000225831	9606.ENSP00000215832	0	0	0	0	0	0	0	0.795	0.795
CCL2	HMOX1	9606.ENSP0000225831	9606.ENSP00000216117	0	0	0	0	0.094	0	0	0.81	0.821
CCL2	NFKBIA	9606.ENSP0000225831	9606.ENSP00000216797	0	0	0	0	0.063	0	0	0.571	0.58
CCL2	MMP2	9606.ENSP0000225831	9606.ENSP00000219070	0	0	0	0	0.155	0	0	0.802	0.825
CCL2	PLAT	9606.ENSP0000225831	9606.ENSP00000220809	0	0	0	0	0.073	0	0	0.409	0.428
CCL2	PON1	9606.ENSP0000225831	9606.ENSP00000222381	0	0	0	0	0	0	0	0.421	0.42
CCL2	SERPINE1	9606.ENSP0000225831	9606.ENSP00000223095	0	0	0	0	0.122	0	0	0.73	0.752

CCL2	MPO	9606.ENSP0000225831	9606.ENSP00000225275	0	0	0	0	0	0	0	0.702	0.702
CCL2	PTGS1	9606.ENSP0000225831	9606.ENSP00000354612	0	0	0	0	0.096	0	0	0.365	0.402
CCL2	NFE2L2	9606.ENSP0000225831	9606.ENSP00000380252	0	0	0	0	0	0	0	0.408	0.408
CCL2	ESR1	9606.ENSP0000225831	9606.ENSP00000405330	0	0	0	0	0.055	0	0	0.421	0.429
CCL2	NR3C1	9606.ENSP0000225831	9606.ENSP00000231509	0	0	0	0	0.055	0	0	0.426	0.434
CCL2	COL3A1	9606.ENSP0000225831	9606.ENSP00000304408	0	0	0	0	0.111	0	0	0.397	0.441
CCL2	SOD1	9606.ENSP0000225831	9606.ENSP00000270142	0	0	0	0	0	0	0	0.444	0.444
CCL2	IKBKB	9606.ENSP0000225831	9606.ENSP00000430684	0	0	0	0	0	0	0	0.492	0.491
CCL2	PLAU	9606.ENSP0000225831	9606.ENSP00000361850	0	0	0	0	0.12	0	0	0.452	0.497

CCL2	COL1A1	9606.ENSP0000225831	9606.ENSP0000225964	0	0	0	0	0.098	0	0	0.473	0.504
CCL2	THBD	9606.ENSP0000225831	9606.ENSP0000366307	0	0	0	0	0.095	0	0	0.475	0.504
CCL2	EGFR	9606.ENSP0000225831	9606.ENSP0000275493	0	0	0	0	0.062	0	0	0.499	0.509
CCL2	MMP10	9606.ENSP0000225831	9606.ENSP0000279441	0	0	0	0	0.076	0	0	0.491	0.51
CCL2	SREBF1	9606.ENSP0000225831	9606.ENSP0000348069	0	0	0	0	0	0	0	0.511	0.511
CCL2	IRF1	9606.ENSP0000225831	9606.ENSP0000245414	0	0	0	0	0.077	0	0	0.526	0.544
CCL2	KDR	9606.ENSP0000225831	9606.ENSP0000263923	0	0	0	0	0.065	0	0	0.534	0.545
CCL2	MAPK14	9606.ENSP0000225831	9606.ENSP0000229795	0	0	0	0	0	0	0	0.559	0.559
CCL2	CD44	9606.ENSP0000225831	9606.ENSP0000398632	0	0	0	0	0.097	0	0	0.555	0.581

CCL2	NOS2	9606.ENSP0000225831	9606.ENSP00000327251	0	0	0	0	0	0	0	0.636	0.636
CCL2	GJA1	9606.ENSP0000225831	9606.ENSP00000282561	0	0	0	0	0.086	0	0	0.623	0.641
CCL2	CCND1	9606.ENSP0000225831	9606.ENSP00000227507	0	0	0	0	0.065	0	0	0.638	0.647
CCL2	NOX1	9606.ENSP0000225831	9606.ENSP00000362057	0	0	0	0	0.061	0	0	0.642	0.65
CCL2	PPARA	9606.ENSP0000225831	9606.ENSP00000385523	0	0	0	0	0.055	0	0	0.654	0.659
CCL2	PPARG	9606.ENSP0000225831	9606.ENSP00000287820	0	0	0	0	0.055	0	0	0.66	0.664
CCL2	HIF1A	9606.ENSP0000225831	9606.ENSP00000437955	0	0	0	0	0.061	0	0	0.658	0.665
CCL2	MYC	9606.ENSP0000225831	9606.ENSP00000479618	0	0	0	0	0.061	0	0	0.66	0.667
CCL2	EGF	9606.ENSP0000225831	9606.ENSP00000265171	0	0	0	0	0	0	0	0.669	0.669

CCL2	NOS3	9606.ENSP0000225831	9606.ENSP00000297494	0	0	0	0	0	0	0	0.669	0.669
CCL2	IL1A	9606.ENSP0000225831	9606.ENSP00000263339	0	0	0	0	0.091	0	0	0.684	0.7
CCL2	OLR1	9606.ENSP0000225831	9606.ENSP00000309124	0	0	0	0	0.084	0	0	0.718	0.73
CCL2	CXCL11	9606.ENSP0000225831	9606.ENSP00000306884	0	0	0	0	0.107	0	0	0.725	0.744
CCL2	SELE	9606.ENSP0000225831	9606.ENSP00000331736	0	0	0	0	0.108	0	0	0.741	0.759
CCL2	F3	9606.ENSP0000225831	9606.ENSP00000334145	0	0	0	0	0.082	0	0	0.777	0.787
CCL2	CRP	9606.ENSP0000225831	9606.ENSP00000255030	0	0	0	0	0	0	0	0.789	0.789
CCL2	STAT1	9606.ENSP0000225831	9606.ENSP00000354394	0	0	0	0	0.063	0	0	0.787	0.793
CCL2	IL2	9606.ENSP0000225831	9606.ENSP00000226730	0	0	0	0	0	0	0	0.795	0.795

CCL2	CD40LG	9606.ENSP0000225831	9606.ENSP00000359663	0	0	0	0	0	0.059	0	0.793	0.797
CCL2	IFNG	9606.ENSP0000225831	9606.ENSP00000229135	0	0	0	0	0.063	0	0	0.794	0.798
CCL2	SPP1	9606.ENSP0000225831	9606.ENSP00000378517	0	0	0	0	0.118	0	0	0.792	0.809
CCL2	MAPK8	9606.ENSP0000225831	9606.ENSP00000378974	0	0	0	0	0	0	0	0.829	0.829
CCL2	MMP9	9606.ENSP0000225831	9606.ENSP00000361405	0	0	0	0	0.076	0	0	0.843	0.849
CCL2	PTGS2	9606.ENSP0000225831	9606.ENSP00000356438	0	0	0	0	0.117	0	0	0.847	0.859
CCL2	MMP3	9606.ENSP0000225831	9606.ENSP00000299855	0	0	0	0	0.107	0.379	0	0.795	0.876
CCL2	MMP1	9606.ENSP0000225831	9606.ENSP00000322788	0	0	0	0	0.102	0.379	0	0.806	0.882
CCL2	CXCL2	9606.ENSP0000225831	9606.ENSP00000427279	0	0	0	0	0.193	0	0	0.885	0.903

CCL2	VEGFA	9606.ENSP00000225831	9606.ENSP00000478570	0	0	0	0	0	0	0	0.914	0.914
CCL2	ICAM1	9606.ENSP00000225831	9606.ENSP00000264832	0	0	0	0	0.149	0	0	0.919	0.929
CCL2	RELA	9606.ENSP00000225831	9606.ENSP00000384273	0	0	0	0	0	0.379	0.8	0.555	0.939
CCL2	VCAM1	9606.ENSP00000225831	9606.ENSP00000294728	0	0	0	0	0.206	0	0	0.927	0.94
CCL2	CXCL10	9606.ENSP00000225831	9606.ENSP00000305651	0	0	0	0	0.158	0	0	0.933	0.941
CCL2	IL1B	9606.ENSP00000225831	9606.ENSP00000263341	0	0	0	0	0.126	0	0	0.945	0.95
CCL2	FOS	9606.ENSP00000225831	9606.ENSP00000306245	0	0	0	0	0	0	0.9	0.719	0.97
CCL2	JUN	9606.ENSP00000225831	9606.ENSP00000360266	0	0	0	0	0.062	0	0.9	0.792	0.978
CCL2	CXCL8	9606.ENSP00000225831	9606.ENSP00000306512	0	0	0	0	0.278	0.667	0	0.925	0.98

CCL2	IL6	9606.ENSP0000225831	9606.ENSP000385675	0	0	0	0	0.267	0	0	0.977	0.982
CCL2	IL4	9606.ENSP0000225831	9606.ENSP000231449	0	0	0	0	0	0	0.9	0.918	0.991
CCL2	IL10	9606.ENSP0000225831	9606.ENSP000412237	0	0	0	0	0	0	0.9	0.955	0.995
CCNA2	MAPK1	9606.ENSP0000274026	9606.ENSP000215832	0	0	0	0	0.063	0.085	0	0.526	0.558
CCNA2	IL2	9606.ENSP0000274026	9606.ENSP000226730	0	0	0	0	0	0	0	0.542	0.542
CCNA2	CCND1	9606.ENSP0000274026	9606.ENSP000227507	0	0	0	0.694	0.084	0.065	0.72	0.885	0.809
CCNA2	CCNB1	9606.ENSP0000274026	9606.ENSP000256442	0	0	0	0.738	0.971	0.707	0.72	0.924	0.998
CCNA2	EGF	9606.ENSP0000274026	9606.ENSP000265171	0	0	0	0	0.061	0	0	0.686	0.693
CCNA2	ERBB2	9606.ENSP0000274026	9606.ENSP000269571	0	0	0	0	0	0.085	0	0.511	0.533

CCNA2	MMP9	9606.ENSP0000274026	9606.ENSP000000361405	0	0	0	0	0	0	0	0.42	0.42
CCNA2	VEGFA	9606.ENSP0000274026	9606.ENSP000000478570	0	0	0	0	0	0	0	0.475	0.475
CCNA2	MAPK8	9606.ENSP0000274026	9606.ENSP000000378974	0	0	0	0	0	0	0	0.511	0.511
CCNA2	ESR1	9606.ENSP0000274026	9606.ENSP000000405330	0	0	0	0	0	0	0	0.528	0.528
CCNA2	FOS	9606.ENSP0000274026	9606.ENSP000000306245	0	0	0	0	0	0.089	0	0.511	0.535
CCNA2	EGFR	9606.ENSP0000274026	9606.ENSP000000275493	0	0	0	0	0	0.085	0	0.541	0.562
CCNA2	PGR	9606.ENSP0000274026	9606.ENSP000000325120	0	0	0	0	0	0.313	0	0.42	0.584
CCNA2	CTNNB1	9606.ENSP0000274026	9606.ENSP000000344456	0	0	0	0	0	0.358	0	0.398	0.597
CCNA2	JUN	9606.ENSP0000274026	9606.ENSP000000360266	0	0	0	0	0	0	0	0.711	0.711

CCNA2	MYC	9606.ENSP0000274026	9606.ENSP000479618	0	0	0	0	0.098	0	0	0.7	0.718
CCNA2	E2F2	9606.ENSP0000274026	9606.ENSP000355249	0	0	0	0	0.215	0.261	0.9	0.714	0.981
CCNA2	E2F1	9606.ENSP0000274026	9606.ENSP000345571	0	0	0	0	0.313	0.521	0.9	0.714	0.989
CCNA2	CDKN1A	9606.ENSP0000274026	9606.ENSP000384849	0	0	0	0	0.058	0.994	0.9	0.793	0.999
CCNB1	MAPK1	9606.ENSP0000256442	9606.ENSP000215832	0	0	0	0	0.062	0.111	0	0.555	0.597
CCNB1	MMP2	9606.ENSP0000256442	9606.ENSP000219070	0	0	0	0	0	0	0	0.65	0.65
CCNB1	CCND1	9606.ENSP0000256442	9606.ENSP000227507	0	0	0	0.65	0.096	0.064	0.9	0.861	0.935
CCNB1	MAPK14	9606.ENSP0000256442	9606.ENSP000229795	0	0	0	0	0.061	0.111	0	0.353	0.413
CCNB1	PTGS2	9606.ENSP0000256442	9606.ENSP000356438	0	0	0	0	0.061	0	0	0.398	0.41

CCNB1	CTNNB1	9606.ENSP00000256442	9606.ENSP00000344456	0	0	0	0	0	0.094	0	0.398	0.431
CCNB1	PGR	9606.ENSP00000256442	9606.ENSP00000325120	0	0	0	0	0	0.05	0	0.446	0.451
CCNB1	VEGFA	9606.ENSP00000256442	9606.ENSP00000478570	0	0	0	0	0	0	0	0.475	0.475
CCNB1	MMP9	9606.ENSP00000256442	9606.ENSP00000361405	0	0	0	0	0	0	0	0.485	0.485
CCNB1	EGF	9606.ENSP00000256442	9606.ENSP00000265171	0	0	0	0	0.063	0	0	0.473	0.485
CCNB1	RELA	9606.ENSP00000256442	9606.ENSP00000384273	0	0	0	0	0.048	0.379	0	0.269	0.53
CCNB1	JUN	9606.ENSP00000256442	9606.ENSP00000360266	0	0	0	0	0	0.108	0	0.497	0.532
CCNB1	ESR1	9606.ENSP00000256442	9606.ENSP00000405330	0	0	0	0	0	0.05	0	0.53	0.534
CCNB1	PARP1	9606.ENSP00000256442	9606.ENSP00000355759	0	0	0	0	0.201	0	0	0.458	0.549

CCNB1	MAPK8	9606.ENSP0000256442	9606.ENSP00000378974	0	0	0	0	0	0	0	0.574	0.574
CCNB1	ERBB2	9606.ENSP0000256442	9606.ENSP00000269571	0	0	0	0	0	0.085	0	0.567	0.587
CCNB1	E2F2	9606.ENSP0000256442	9606.ENSP00000355249	0	0	0	0	0.117	0.169	0	0.527	0.623
CCNB1	FOS	9606.ENSP0000256442	9606.ENSP00000306245	0	0	0	0	0	0.125	0	0.63	0.662
CCNB1	RUNX2	9606.ENSP0000256442	9606.ENSP00000360493	0	0	0	0	0.061	0.379	0	0.508	0.688
CCNB1	EGFR	9606.ENSP0000256442	9606.ENSP00000275493	0	0	0	0	0	0.085	0	0.722	0.735
CCNB1	MYC	9606.ENSP0000256442	9606.ENSP00000479618	0	0	0	0	0.096	0.354	0	0.685	0.8
CCNB1	E2F1	9606.ENSP0000256442	9606.ENSP00000345571	0	0	0	0	0.249	0.169	0.9	0.54	0.967
CCNB1	CDKN1A	9606.ENSP0000256442	9606.ENSP00000384849	0	0	0	0	0	0.774	0.9	0.744	0.993

CCND1	MAPK1	9606.ENSP00000227507	9606.ENSP00000215832	0	0	0	0	0	0.348	0	0.834	0.887
CCND1	HMOX1	9606.ENSP00000227507	9606.ENSP00000216117	0	0	0	0	0	0	0	0.423	0.422
CCND1	NFKBIA	9606.ENSP00000227507	9606.ENSP00000216797	0	0	0	0	0	0	0	0.475	0.475
CCND1	MMP2	9606.ENSP00000227507	9606.ENSP00000219070	0	0	0	0	0.139	0	0	0.676	0.709
CCND1	SERPINE1	9606.ENSP00000227507	9606.ENSP00000223095	0	0	0	0	0.135	0	0	0.42	0.476
CCND1	COL1A1	9606.ENSP00000227507	9606.ENSP00000225964	0	0	0	0	0.152	0	0	0.329	0.406
CCND1	IL2	9606.ENSP00000227507	9606.ENSP00000226730	0	0	0	0	0	0	0	0.43	0.43
CCND1	IL10	9606.ENSP00000227507	9606.ENSP00000412237	0	0	0	0	0	0	0	0.419	0.418
CCND1	GJA1	9606.ENSP00000227507	9606.ENSP00000282561	0	0	0	0	0.098	0	0	0.39	0.426

CCND1	HSPB1	9606.ENSP0000227507	9606.ENSP00000248553	0	0	0	0	0.088	0.133	0	0.355	0.445
CCND1	NR3C1	9606.ENSP0000227507	9606.ENSP00000231509	0	0	0	0	0	0.05	0	0.443	0.448
CCND1	SPP1	9606.ENSP0000227507	9606.ENSP00000378517	0	0	0	0	0.061	0	0	0.446	0.458
CCND1	ICAM1	9606.ENSP0000227507	9606.ENSP00000264832	0	0	0	0	0.061	0	0	0.453	0.465
CCND1	PLAU	9606.ENSP0000227507	9606.ENSP00000361850	0	0	0	0	0.11	0	0	0.425	0.466
CCND1	MMP1	9606.ENSP0000227507	9606.ENSP00000322788	0	0	0	0	0.104	0	0	0.439	0.476
CCND1	TP63	9606.ENSP0000227507	9606.ENSP00000264731	0	0	0	0	0.062	0	0	0.466	0.477
CCND1	MMP3	9606.ENSP0000227507	9606.ENSP00000299855	0	0	0	0	0.076	0	0	0.479	0.498
CCND1	ODC1	9606.ENSP0000227507	9606.ENSP00000234111	0	0	0	0	0.062	0	0	0.499	0.51

CCND1	RUNX2	9606.ENSP00000227507	9606.ENSP00000360493	0	0	0	0	0	0	0	0.515	0.515
CCND1	HIF1A	9606.ENSP00000227507	9606.ENSP00000437955	0	0	0	0	0	0	0	0.517	0.517
CCND1	PARP1	9606.ENSP00000227507	9606.ENSP00000355759	0	0	0	0	0	0	0	0.53	0.53
CCND1	CXCL8	9606.ENSP00000227507	9606.ENSP00000306512	0	0	0	0	0	0	0	0.544	0.544
CCND1	IGFBP3	9606.ENSP00000227507	9606.ENSP00000370473	0	0	0	0	0.141	0.132	0	0.444	0.549
CCND1	STAT1	9606.ENSP00000227507	9606.ENSP00000354394	0	0	0	0	0.049	0.067	0	0.535	0.551
CCND1	IRF1	9606.ENSP00000227507	9606.ENSP00000245414	0	0	0	0	0	0	0	0.558	0.558
CCND1	SREBF1	9606.ENSP00000227507	9606.ENSP00000348069	0	0	0	0	0.062	0	0	0.577	0.587
CCND1	CHUK	9606.ENSP00000227507	9606.ENSP00000359424	0	0	0	0	0	0	0	0.587	0.588

CCND1	KDR	9606.ENSP0000227507	9606.ENSP00000263923	0	0	0	0	0.074	0	0	0.581	0.595
CCND1	PPARG	9606.ENSP0000227507	9606.ENSP00000287820	0	0	0	0	0	0.05	0	0.601	0.605
CCND1	IL4	9606.ENSP0000227507	9606.ENSP00000231449	0	0	0	0	0	0	0	0.613	0.614
CCND1	IKBKB	9606.ENSP0000227507	9606.ENSP00000430684	0	0	0	0	0	0	0	0.637	0.637
CCND1	PTGS2	9606.ENSP0000227507	9606.ENSP00000356438	0	0	0	0	0.055	0	0	0.674	0.678
CCND1	ESR2	9606.ENSP0000227507	9606.ENSP00000343925	0	0	0	0	0	0.05	0	0.706	0.709
CCND1	MAPK14	9606.ENSP0000227507	9606.ENSP00000229795	0	0	0	0	0	0	0	0.741	0.742
CCND1	MMP9	9606.ENSP0000227507	9606.ENSP00000361405	0	0	0	0	0	0	0	0.765	0.765
CCND1	MET	9606.ENSP0000227507	9606.ENSP00000317272	0	0	0	0	0.139	0	0	0.749	0.776

CCND1	CD44	9606.ENSP00000227507	9606.ENSP00000398632	0	0	0	0	0	0	0	0.78	0.78
CCND1	VEGFA	9606.ENSP00000227507	9606.ENSP00000478570	0	0	0	0	0.069	0.17	0	0.743	0.784
CCND1	IL6	9606.ENSP00000227507	9606.ENSP00000385675	0	0	0	0	0	0	0	0.812	0.813
CCND1	MAPK8	9606.ENSP00000227507	9606.ENSP00000378974	0	0	0	0	0	0	0	0.818	0.818
CCND1	EGF	9606.ENSP00000227507	9606.ENSP00000265171	0	0	0	0	0	0	0	0.844	0.844
CCND1	ERBB2	9606.ENSP00000227507	9606.ENSP00000269571	0	0	0	0	0.098	0.43	0	0.799	0.888
CCND1	PGR	9606.ENSP00000227507	9606.ENSP00000325120	0	0	0	0	0	0.43	0	0.817	0.892
CCND1	IGF2	9606.ENSP00000227507	9606.ENSP00000391826	0	0	0	0	0.092	0	0	0.901	0.907
CCND1	EGFR	9606.ENSP00000227507	9606.ENSP00000275493	0	0	0	0	0.2	0.157	0	0.886	0.917

CCND1	MYC	9606.ENSP00000227507	9606.ENSP00000479618	0	0	0	0	0.069	0	0	0.963	0.964
CCND1	FOS	9606.ENSP00000227507	9606.ENSP00000306245	0	0	0	0	0	0	0.9	0.685	0.967
CCND1	RELA	9606.ENSP00000227507	9606.ENSP00000384273	0	0	0	0	0	0.313	0.9	0.559	0.967
CCND1	E2F2	9606.ENSP00000227507	9606.ENSP00000355249	0	0	0	0	0.041	0.132	0.9	0.689	0.97
CCND1	E2F1	9606.ENSP00000227507	9606.ENSP00000345571	0	0	0	0	0.064	0.132	0.9	0.713	0.973
CCND1	JUN	9606.ENSP00000227507	9606.ENSP00000360266	0	0	0	0	0	0	0.9	0.862	0.985
CCND1	GSK3B	9606.ENSP00000227507	9606.ENSP00000324806	0	0	0	0	0	0.526	0.9	0.822	0.99
CCND1	ESR1	9606.ENSP00000227507	9606.ENSP00000405330	0	0	0	0	0	0.384	0.9	0.858	0.99
CCND1	CTNNB1	9606.ENSP00000227507	9606.ENSP00000344456	0	0	0	0	0.081	0.472	0.9	0.921	0.995

CCND1	CDKN1A	9606.ENSP00000227507	9606.ENSP00000384849	0	0	0	0	0.084	0.993	0.9	0.857	0.999
CD40LG	MAPK1	9606.ENSP00000359663	9606.ENSP00000215832	0	0	0	0	0	0	0	0.42	0.42
CD40LG	NFKBIA	9606.ENSP00000359663	9606.ENSP00000216797	0	0	0	0	0	0.059	0.9	0.329	0.931
CD40LG	SERPINE1	9606.ENSP00000359663	9606.ENSP00000223095	0	0	0	0	0	0.051	0	0.473	0.478
CD40LG	MPO	9606.ENSP00000359663	9606.ENSP00000225275	0	0	0	0	0	0.056	0	0.513	0.52
CD40LG	IL2	9606.ENSP00000359663	9606.ENSP00000226730	0	0	0	0	0.076	0	0	0.878	0.883
CD40LG	IFNG	9606.ENSP00000359663	9606.ENSP00000229135	0	0	0	0	0.116	0	0	0.677	0.703
CD40LG	MAPK14	9606.ENSP00000359663	9606.ENSP00000229795	0	0	0	0	0	0	0.9	0.303	0.927
CD40LG	IL4	9606.ENSP00000359663	9606.ENSP00000231449	0	0	0	0	0	0	0	0.914	0.914

CD40LG	NR3C1	9606.ENSP0000359663	9606.ENSP00000231509	0	0	0	0	0	0	0	0.466	0.466
CD40LG	CRP	9606.ENSP0000359663	9606.ENSP00000255030	0	0	0	0	0	0	0	0.669	0.669
CD40LG	IL1B	9606.ENSP0000359663	9606.ENSP00000263341	0	0	0	0	0	0	0	0.568	0.568
CD40LG	ICAM1	9606.ENSP0000359663	9606.ENSP00000264832	0	0	0	0	0	0	0	0.816	0.816
CD40LG	EGF	9606.ENSP0000359663	9606.ENSP00000265171	0	0	0	0	0	0.062	0	0.4	0.413
CD40LG	VCAM1	9606.ENSP0000359663	9606.ENSP00000294728	0	0	0	0	0.085	0	0	0.845	0.852
CD40LG	CXCL10	9606.ENSP0000359663	9606.ENSP00000305651	0	0	0	0	0	0	0	0.588	0.588
CD40LG	CXCL8	9606.ENSP0000359663	9606.ENSP00000306512	0	0	0	0	0.061	0	0	0.669	0.676
CD40LG	CXCL11	9606.ENSP0000359663	9606.ENSP00000306884	0	0	0	0	0	0	0	0.423	0.422

CD40LG	SELE	9606.ENSP00000359663	9606.ENSP00000331736	0	0	0	0	0.055	0	0	0.754	0.757
CD40LG	F3	9606.ENSP00000359663	9606.ENSP00000334145	0	0	0	0	0	0	0	0.59	0.59
CD40LG	STAT1	9606.ENSP00000359663	9606.ENSP00000354394	0	0	0	0	0	0	0	0.496	0.495
CD40LG	PTGS2	9606.ENSP00000359663	9606.ENSP00000356438	0	0	0	0	0	0.056	0	0.399	0.408
CD40LG	JUN	9606.ENSP00000359663	9606.ENSP00000360266	0	0	0	0	0	0	0	0.417	0.416
CD40LG	CDKN1A	9606.ENSP00000359663	9606.ENSP00000384849	0	0	0	0	0	0	0	0.464	0.464
CD40LG	THBD	9606.ENSP00000359663	9606.ENSP00000366307	0	0	0	0	0	0	0	0.504	0.504
CD40LG	MMP9	9606.ENSP00000359663	9606.ENSP00000361405	0	0	0	0	0	0.064	0	0.511	0.522
CD40LG	VEGFA	9606.ENSP00000359663	9606.ENSP00000478570	0	0	0	0	0	0	0	0.556	0.556

CD40LG	CD44	9606.ENSP0000359663	9606.ENSP00000398632	0	0	0	0	0	0	0	0.571	0.571
CD40LG	IL6	9606.ENSP0000359663	9606.ENSP00000385675	0	0	0	0	0	0	0	0.741	0.741
CD40LG	IL10	9606.ENSP0000359663	9606.ENSP00000412237	0	0	0	0	0	0	0	0.802	0.802
CD40LG	RELA	9606.ENSP0000359663	9606.ENSP00000384273	0	0	0	0	0	0	0.9	0.477	0.945
CD40LG	MAPK8	9606.ENSP0000359663	9606.ENSP00000378974	0	0	0	0	0	0.076	0.9	0.473	0.947
CD44	MAPK1	9606.ENSP0000398632	9606.ENSP00000215832	0	0	0	0	0	0.185	0	0.485	0.562
CD44	MMP2	9606.ENSP0000398632	9606.ENSP00000219070	0	0	0	0	0.068	0	0.9	0.642	0.963
CD44	SERPINE1	9606.ENSP0000398632	9606.ENSP00000223095	0	0	0	0	0.123	0	0	0.42	0.469
CD44	COL1A1	9606.ENSP0000398632	9606.ENSP00000225964	0	0	0	0	0.084	0.379	0.6	0.441	0.855

CD44	IL2	9606.ENSP00000398632	9606.ENSP000002226730	0	0	0	0	0	0	0	0.741	0.741
CD44	IFNG	9606.ENSP00000398632	9606.ENSP000002229135	0	0	0	0	0	0	0	0.596	0.596
CD44	MAPK14	9606.ENSP00000398632	9606.ENSP000002229795	0	0	0	0	0	0.144	0	0.334	0.405
CD44	IL4	9606.ENSP00000398632	9606.ENSP000002231449	0	0	0	0	0	0	0	0.676	0.676
CD44	IRF1	9606.ENSP00000398632	9606.ENSP000002245414	0	0	0	0	0.071	0	0.9	0.247	0.924
CD44	IL1B	9606.ENSP00000398632	9606.ENSP000002263341	0	0	0	0	0.098	0	0	0.699	0.717
CD44	KDR	9606.ENSP00000398632	9606.ENSP000002263923	0	0	0	0	0	0	0	0.591	0.591
CD44	ICAM1	9606.ENSP00000398632	9606.ENSP000002264832	0	0	0	0	0.117	0	0	0.733	0.754
CD44	EGF	9606.ENSP00000398632	9606.ENSP000002265171	0	0	0	0	0	0	0	0.84	0.84

CD44	ERBB2	9606.<br>ENSP0<br>00003<br>98632	9606.E<br>NSP00<br>00026<br>9571	0	0	0	0	0	0.379	0.8	0.845	0.979
CD44	EGFR	9606.<br>ENSP0<br>00003<br>98632	9606.E<br>NSP00<br>00027<br>5493	0	0	0	0	0.099	0.379	0	0.871	0.921
CD44	GJA1	9606.<br>ENSP0<br>00003<br>98632	9606.E<br>NSP00<br>00028<br>2561	0	0	0	0	0.062	0	0	0.413	0.425
CD44	VCAM1	9606.<br>ENSP0<br>00003<br>98632	9606.E<br>NSP00<br>00029<br>4728	0	0	0	0	0.063	0	0	0.712	0.719
CD44	MMP3	9606.<br>ENSP0<br>00003<br>98632	9606.E<br>NSP00<br>00029<br>9855	0	0	0	0	0.097	0	0	0.547	0.574
CD44	CXCL10	9606.<br>ENSP0<br>00003<br>98632	9606.E<br>NSP00<br>00030<br>5651	0	0	0	0	0.084	0	0	0.449	0.474
CD44	FOS	9606.<br>ENSP0<br>00003<br>98632	9606.E<br>NSP00<br>00030<br>6245	0	0	0	0	0	0	0	0.416	0.416
CD44	CXCL8	9606.<br>ENSP0<br>00003<br>98632	9606.E<br>NSP00<br>00030<br>6512	0	0	0	0	0.139	0	0	0.598	0.639
CD44	HAS2	9606.<br>ENSP0<br>00003<br>98632	9606.E<br>NSP00<br>00030<br>6991	0	0	0	0	0.059	0	0	0.796	0.8

CD44	MET	9606.ENSP0000398632	9606.ENSP00000317272	0	0	0	0	0.075	0.316	0	0.784	0.851
CD44	MMP1	9606.ENSP0000398632	9606.ENSP00000322788	0	0	0	0	0.126	0.379	0	0.488	0.698
CD44	PGR	9606.ENSP0000398632	9606.ENSP00000325120	0	0	0	0	0	0	0	0.539	0.539
CD44	SELE	9606.ENSP0000398632	9606.ENSP00000331736	0	0	0	0	0	0.447	0.9	0.817	0.989
CD44	CTNNB1	9606.ENSP0000398632	9606.ENSP00000344456	0	0	0	0	0.044	0	0	0.663	0.664
CD44	STAT1	9606.ENSP0000398632	9606.ENSP00000354394	0	0	0	0	0.069	0	0	0.45	0.466
CD44	PTGS2	9606.ENSP0000398632	9606.ENSP00000356438	0	0	0	0	0.123	0	0	0.477	0.522
CD44	JUN	9606.ENSP0000398632	9606.ENSP00000360266	0	0	0	0	0.064	0	0	0.707	0.714
CD44	RUNX2	9606.ENSP0000398632	9606.ENSP00000360493	0	0	0	0	0.107	0	0	0.723	0.742

CD44	MMP9	9606.ENSP00000398632	9606.ENSP00000361405	0	0	0	0	0	0.379	0.9	0.817	0.987
CD44	PLAU	9606.ENSP00000398632	9606.ENSP00000361850	0	0	0	0	0.124	0	0	0.422	0.472
CD44	IGFBP3	9606.ENSP00000398632	9606.ENSP00000370473	0	0	0	0	0.076	0.379	0	0.297	0.561
CD44	SPP1	9606.ENSP00000398632	9606.ENSP00000378517	0	0	0	0	0.062	0.379	0.9	0.892	0.992
CD44	MAPK8	9606.ENSP00000398632	9606.ENSP00000378974	0	0	0	0	0	0	0	0.512	0.512
CD44	CDKN1A	9606.ENSP00000398632	9606.ENSP00000384849	0	0	0	0	0.088	0	0	0.418	0.446
CD44	IL6	9606.ENSP00000398632	9606.ENSP00000385675	0	0	0	0	0.088	0	0	0.68	0.696
CD44	IGF2	9606.ENSP00000398632	9606.ENSP00000391826	0	0	0	0	0.055	0	0	0.624	0.63
CD44	HIF1A	9606.ENSP00000398632	9606.ENSP00000437955	0	0	0	0	0.06	0	0	0.419	0.43

CD44	ESR1	9606.ENSP00003986 32	9606.ENSP000040 5330	0	0	0	0	0	0	0	0.588	0.588
CD44	MYC	9606.ENSP00003986 32	9606.ENSP000047 9618	0	0	0	0	0.07	0	0	0.681	0.69
CD44	VEGFA	9606.ENSP00003986 32	9606.ENSP000047 8570	0	0	0	0	0	0	0	0.804	0.805
CD44	IL10	9606.ENSP00003986 32	9606.ENSP000041 2237	0	0	0	0	0	0	0	0.93	0.931
CDKN1A	MAPK1	9606.ENSP00003848 49	9606.ENSP000021 5832	0	0	0	0	0	0	0	0.538	0.538
CDKN1A	HMOX1	9606.ENSP00003848 49	9606.ENSP000021 6117	0	0	0	0	0.076	0	0	0.418	0.439
CDKN1A	MMP2	9606.ENSP00003848 49	9606.ENSP000021 9070	0	0	0	0	0.061	0	0	0.417	0.429
CDKN1A	SERPINE1	9606.ENSP00003848 49	9606.ENSP000022 3095	0	0	0	0	0.104	0	0	0.424	0.462
CDKN1A	MAPK14	9606.ENSP00003848 49	9606.ENSP000022 9795	0	0	0	0	0	0.379	0	0.454	0.646

CDKN1A	TP63	9606.ENSP0000384849	9606.ENSP00000264731	0	0	0	0	0.06	0.454	0	0.512	0.727
CDKN1A	EGF	9606.ENSP0000384849	9606.ENSP00000265171	0	0	0	0	0	0	0	0.56	0.56
CDKN1A	ERBB2	9606.ENSP0000384849	9606.ENSP00000269571	0	0	0	0	0	0	0	0.531	0.531
CDKN1A	EGFR	9606.ENSP0000384849	9606.ENSP00000275493	0	0	0	0	0.064	0	0	0.57	0.581
CDKN1A	FOS	9606.ENSP0000384849	9606.ENSP00000306245	0	0	0	0	0	0	0	0.518	0.518
CDKN1A	CXCL8	9606.ENSP0000384849	9606.ENSP00000306512	0	0	0	0	0.106	0	0	0.42	0.459
CDKN1A	GSK3B	9606.ENSP0000384849	9606.ENSP00000324806	0	0	0	0	0	0.084	0	0.58	0.599
CDKN1A	PGR	9606.ENSP0000384849	9606.ENSP00000325120	0	0	0	0	0	0.313	0	0.386	0.56
CDKN1A	CTNNB1	9606.ENSP0000384849	9606.ENSP00000344456	0	0	0	0	0	0	0	0.48	0.48

CDKN1A E2F1	9606.ENSP00000384849	9606.ENSP00000345571	0	0	0	0	0.052	0.435	0.9	0.617	0.976
CDKN1A STAT1	9606.ENSP00000384849	9606.ENSP00000354394	0	0	0	0	0.067	0	0	0.419	0.435
CDKN1A E2F2	9606.ENSP00000384849	9606.ENSP00000355249	0	0	0	0	0.052	0.129	0.9	0.608	0.963
CDKN1A PARP1	9606.ENSP00000384849	9606.ENSP00000355759	0	0	0	0	0	0.379	0	0.454	0.646
CDKN1A PTGS2	9606.ENSP00000384849	9606.ENSP00000356438	0	0	0	0	0.095	0	0	0.44	0.471
CDKN1A JUN	9606.ENSP00000384849	9606.ENSP00000360266	0	0	0	0	0.079	0.305	0.9	0.743	0.981
CDKN1A RUNX2	9606.ENSP00000384849	9606.ENSP00000360493	0	0	0	0	0	0	0	0.585	0.585
CDKN1A MMP9	9606.ENSP00000384849	9606.ENSP00000361405	0	0	0	0	0	0	0	0.47	0.47
CDKN1A IGFBP3	9606.ENSP00000384849	9606.ENSP00000370473	0	0	0	0	0.07	0	0	0.446	0.462

CDKN1A	MAPK8	9606.ENSP00000384849	9606.ENSP00000378974	0	0	0	0	0	0.379	0.9	0.593	0.972
CDKN1A	NFE2L2	9606.ENSP00000384849	9606.ENSP00000380252	0	0	0	0	0.06	0.404	0	0.296	0.571
CDKN1A	RELA	9606.ENSP00000384849	9606.ENSP00000384273	0	0	0	0	0.076	0.313	0	0.4	0.585
CDKN1A	IKBKB	9606.ENSP00000384849	9606.ENSP00000430684	0	0	0	0	0	0.084	0	0.52	0.541
CDKN1A	VEGFA	9606.ENSP00000384849	9606.ENSP00000478570	0	0	0	0	0.069	0	0	0.539	0.552
CDKN1A	PRKCA	9606.ENSP00000384849	9606.ENSP00000408695	0	0	0	0	0	0	0	0.576	0.576
CDKN1A	IL6	9606.ENSP00000384849	9606.ENSP00000385675	0	0	0	0	0.076	0	0	0.674	0.686
CDKN1A	ESR1	9606.ENSP00000384849	9606.ENSP00000405330	0	0	0	0	0	0.379	0	0.559	0.714
CDKN1A	HIF1A	9606.ENSP00000384849	9606.ENSP00000437955	0	0	0	0	0	0	0.9	0.399	0.937

CDKN1A	MYC	9606.ENSP00000384849	9606.ENSP000004796 18	0	0	0	0	0	0.378	0.9	0.945	0.996
CHUK	NFKBIA	9606.ENSP00000359424	9606.ENSP000002167 97	0	0	0	0	0.049	0.917	0.9	0.825	0.998
CHUK	HSPB1	9606.ENSP00000359424	9606.ENSP000002485 53	0	0	0	0	0	0.402	0	0.483	0.678
CHUK	IL1A	9606.ENSP00000359424	9606.ENSP000002633 39	0	0	0	0	0.061	0	0.9	0.22	0.92
CHUK	IL1B	9606.ENSP00000359424	9606.ENSP000002633 41	0	0	0	0	0	0.059	0	0.4	0.411
CHUK	TP63	9606.ENSP00000359424	9606.ENSP000002647 31	0	0	0	0	0	0.379	0	0.323	0.561
CHUK	CXCL8	9606.ENSP00000359424	9606.ENSP000003065 12	0	0	0	0	0	0	0	0.409	0.408
CHUK	CTNNB1	9606.ENSP00000359424	9606.ENSP000003444 56	0	0	0	0	0.062	0.444	0	0.517	0.726
CHUK	PTGS2	9606.ENSP00000359424	9606.ENSP000003564 38	0	0	0	0	0.062	0.058	0	0.4	0.423

CHUK	IL6	9606.ENSP00000359424	9606.ENSP00000385675	0	0	0	0	0.048	0	0	0.447	0.451
CHUK	JUN	9606.ENSP00000359424	9606.ENSP00000360266	0	0	0	0	0	0.072	0	0.471	0.488
CHUK	MYC	9606.ENSP00000359424	9606.ENSP00000479618	0	0	0	0	0.05	0.391	0	0.308	0.565
CHUK	ESR1	9606.ENSP00000359424	9606.ENSP00000405330	0	0	0	0	0	0.407	0	0.509	0.696
CHUK	PRKCA	9606.ENSP00000359424	9606.ENSP00000408695	0	0	0	0.568	0	0.358	0.9	0.31	0.941
CHUK	IKBKB	9606.ENSP00000359424	9606.ENSP00000430684	0	0	0	0.942	0	0.91	0.9	0.953	0.991
CHUK	RELA	9606.ENSP00000359424	9606.ENSP00000384273	0	0	0	0	0.067	0.871	0.9	0.705	0.995
COL1A1	MMP2	9606.ENSP00000225964	9606.ENSP00000219070	0	0	0	0	0.89	0.379	0	0.6	0.97
COL1A1	SERPINE1	9606.ENSP00000225964	9606.ENSP00000223095	0	0	0	0	0.214	0.062	0	0.503	0.602

COL1A1	IL1B	9606.<br>ENSP0<br>00002<br>25964	9606.E<br>NSP00<br>00026<br>3341	0	0	0	0	0	0	0	0.42	0.42
COL1A1	ESR1	9606.<br>ENSP0<br>00002<br>25964	9606.E<br>NSP00<br>00040<br>5330	0	0	0	0	0	0.051	0	0.418	0.424
COL1A1	IGF2	9606.<br>ENSP0<br>00002<br>25964	9606.E<br>NSP00<br>00039<br>1826	0	0	0	0	0.182	0	0	0.33	0.428
COL1A1	GJA1	9606.<br>ENSP0<br>00002<br>25964	9606.E<br>NSP00<br>00028<br>2561	0	0	0	0	0.158	0	0	0.353	0.432
COL1A1	CTNNB1	9606.<br>ENSP0<br>00002<br>25964	9606.E<br>NSP00<br>00034<br>4456	0	0	0	0	0	0.05	0	0.43	0.436
COL1A1	PPARG	9606.<br>ENSP0<br>00002<br>25964	9606.E<br>NSP00<br>00028<br>7820	0	0	0	0	0	0.051	0	0.449	0.455
COL1A1	EGFR	9606.<br>ENSP0<br>00002<br>25964	9606.E<br>NSP00<br>00027<br>5493	0	0	0	0	0.172	0.099	0	0.339	0.464
COL1A1	HAS2	9606.<br>ENSP0<br>00002<br>25964	9606.E<br>NSP00<br>00030<br>6991	0	0	0	0	0.152	0	0	0.421	0.488
COL1A1	VEGFA	9606.<br>ENSP0<br>00002<br>25964	9606.E<br>NSP00<br>00047<br>8570	0	0	0	0	0.076	0	0	0.5	0.518

COL1A1	MMP1	9606.ENSP00000225964	9606.ENSP00000322788	0	0	0	0	0.128	0	0	0.569	0.608
COL1A1	MMP3	9606.ENSP00000225964	9606.ENSP00000299855	0	0	0	0	0.159	0	0	0.567	0.621
COL1A1	IGFBP3	9606.ENSP00000225964	9606.ENSP00000370473	0	0	0	0	0.29	0.379	0	0.328	0.677
COL1A1	SPP1	9606.ENSP00000225964	9606.ENSP00000378517	0	0	0	0	0.062	0	0	0.686	0.693
COL1A1	MMP9	9606.ENSP00000225964	9606.ENSP00000361405	0	0	0	0	0.077	0.328	0	0.605	0.733
COL1A1	IL6	9606.ENSP00000225964	9606.ENSP00000385675	0	0	0	0	0.065	0	0	0.735	0.741
COL1A1	RUNX2	9606.ENSP00000225964	9606.ENSP00000360493	0	0	0	0	0	0	0	0.823	0.823
COL1A1	COL3A1	9606.ENSP00000225964	9606.ENSP00000304408	0	0	0.439	0.951	0.944	0	0.9	0.901	0.994
COL3A1	MMP2	9606.ENSP00000304408	9606.ENSP00000219070	0	0	0	0	0.482	0	0	0.552	0.758

COL3A1	SERPINE1	9606.ENSP0000030440 8	9606.ENSP000002230 95	0	0	0	0	0.116	0.062	0	0.417	0.475
COL3A1	VCAM1	9606.ENSP0000030440 8	9606.ENSP000002947 28	0	0	0	0	0.222	0	0	0.292	0.425
COL3A1	MMP3	9606.ENSP0000030440 8	9606.ENSP000002998 55	0	0	0	0	0.134	0	0	0.52	0.566
COL3A1	IGFBP3	9606.ENSP0000030440 8	9606.ENSP000003704 73	0	0	0	0	0.227	0	0	0.266	0.408
COL3A1	SPP1	9606.ENSP0000030440 8	9606.ENSP000003785 17	0	0	0	0	0.061	0	0	0.399	0.411
COL3A1	IL6	9606.ENSP0000030440 8	9606.ENSP000003856 75	0	0	0	0	0.063	0	0	0.398	0.411
COL3A1	HAS2	9606.ENSP0000030440 8	9606.ENSP000003069 91	0	0	0	0	0.098	0	0	0.418	0.453
COL3A1	MMP1	9606.ENSP0000030440 8	9606.ENSP000003227 88	0	0	0	0	0.088	0	0	0.483	0.508
COL3A1	MMP9	9606.ENSP0000030440 8	9606.ENSP000003614 05	0	0	0	0	0.078	0	0	0.541	0.558

CRH	IL2	9606.ENSP00000276571	9606.ENSP00000226730	0	0	0	0	0	0	0	0.626	0.627
CRH	NR3C1	9606.ENSP00000276571	9606.ENSP00000231509	0	0	0	0	0	0	0	0.899	0.9
CRH	OPRD1	9606.ENSP00000276571	9606.ENSP00000234961	0	0	0	0	0	0	0	0.669	0.669
CRH	CRP	9606.ENSP00000276571	9606.ENSP00000255030	0	0	0	0	0	0	0	0.402	0.402
CRH	SLC6A4	9606.ENSP00000276571	9606.ENSP00000261707	0	0	0	0	0.063	0	0	0.668	0.675
CRH	IL1A	9606.ENSP00000276571	9606.ENSP00000263339	0	0	0	0	0	0	0	0.415	0.414
CRH	IL1B	9606.ENSP00000276571	9606.ENSP00000263341	0	0	0	0	0	0	0	0.731	0.731
CRH	GNRH1	9606.ENSP00000276571	9606.ENSP00000276414	0	0	0	0	0	0	0	0.742	0.742
CRH	IL10	9606.ENSP00000276571	9606.ENSP00000412237	0	0	0	0	0	0	0	0.414	0.414

CRH	ESR1	9606.ENSP0000276571	9606.ENSP00000405330	0	0	0	0	0	0	0	0.461	0.461
CRH	HTR3A	9606.ENSP0000276571	9606.ENSP00000347754	0	0	0	0	0.064	0	0	0.553	0.564
CRH	DRD2	9606.ENSP0000276571	9606.ENSP00000354859	0	0	0	0	0.061	0	0	0.556	0.566
CRH	ESR2	9606.ENSP0000276571	9606.ENSP00000343925	0	0	0	0	0	0	0	0.611	0.611
CRH	OPRM1	9606.ENSP0000276571	9606.ENSP00000394624	0	0	0	0	0	0	0	0.677	0.677
CRH	IL6	9606.ENSP0000276571	9606.ENSP00000385675	0	0	0	0	0	0	0	0.743	0.743
CRH	FOS	9606.ENSP0000276571	9606.ENSP00000306245	0	0	0	0	0	0	0	0.915	0.915
CRP	HMOX1	9606.ENSP0000255030	9606.ENSP00000216117	0	0	0	0	0	0	0	0.45	0.45
CRP	MMP2	9606.ENSP0000255030	9606.ENSP00000219070	0	0	0	0	0	0	0	0.707	0.707

CRP	PLAT	9606.ENSP00000255030	9606.ENSP00000220809	0	0	0	0	0	0	0	0.584	0.584
CRP	PON1	9606.ENSP00000255030	9606.ENSP00000222381	0	0	0	0	0.098	0	0	0.649	0.669
CRP	SERPINE1	9606.ENSP00000255030	9606.ENSP00000223095	0	0	0	0	0.049	0	0	0.898	0.899
CRP	MPO	9606.ENSP00000255030	9606.ENSP00000225275	0	0	0	0	0	0	0	0.789	0.789
CRP	IL2	9606.ENSP00000255030	9606.ENSP00000226730	0	0	0	0	0	0	0	0.71	0.71
CRP	IFNG	9606.ENSP00000255030	9606.ENSP00000229135	0	0	0	0	0	0	0	0.676	0.676
CRP	IL4	9606.ENSP00000255030	9606.ENSP00000231449	0	0	0	0	0	0	0	0.669	0.669
CRP	NR3C1	9606.ENSP00000255030	9606.ENSP00000231509	0	0	0	0	0	0	0	0.47	0.47
CRP	JUN	9606.ENSP00000255030	9606.ENSP00000360266	0	0	0	0	0	0	0	0.403	0.403

CRP	CYP3A4	9606.ENSP0000025503 0	9606.ENSP0000033 7915	0	0	0	0	0.085	0	0	0.377	0.405
CRP	ESR1	9606.ENSP0000025503 0	9606.ENSP0000040 5330	0	0	0	0	0.061	0.076	0	0.397	0.431
CRP	KDR	9606.ENSP0000025503 0	9606.ENSP0000026 3923	0	0	0	0	0.061	0	0	0.421	0.433
CRP	MAPK8	9606.ENSP0000025503 0	9606.ENSP0000037 8974	0	0	0	0	0	0	0	0.461	0.461
CRP	IL1A	9606.ENSP0000025503 0	9606.ENSP0000026 3339	0	0	0	0	0.063	0	0	0.45	0.462
CRP	CTNNB1	9606.ENSP0000025503 0	9606.ENSP0000034 4456	0	0	0	0	0	0	0	0.492	0.492
CRP	IGFBP3	9606.ENSP0000025503 0	9606.ENSP0000037 0473	0	0	0	0	0	0	0	0.497	0.497
CRP	SPP1	9606.ENSP0000025503 0	9606.ENSP0000037 8517	0	0	0	0	0	0	0	0.515	0.515
CRP	MMP10	9606.ENSP0000025503 0	9606.ENSP0000027 9441	0	0	0	0	0	0	0	0.525	0.526

CRP	PPARG	9606.ENSP00000255030	9606.ENSP00000287820	0	0	0	0	0	0	0	0.53	0.53
CRP	CXCL10	9606.ENSP00000255030	9606.ENSP00000305651	0	0	0	0	0	0	0	0.559	0.559
CRP	PTGS2	9606.ENSP00000255030	9606.ENSP00000356438	0	0	0	0	0	0	0	0.614	0.614
CRP	F7	9606.ENSP00000255030	9606.ENSP00000364731	0	0	0	0	0.085	0	0	0.631	0.648
CRP	MMP3	9606.ENSP00000255030	9606.ENSP00000299855	0	0	0	0	0	0	0	0.656	0.656
CRP	MMP1	9606.ENSP00000255030	9606.ENSP00000322788	0	0	0	0	0.052	0	0	0.724	0.727
CRP	OLR1	9606.ENSP00000255030	9606.ENSP00000309124	0	0	0	0	0	0	0	0.756	0.756
CRP	THBD	9606.ENSP00000255030	9606.ENSP00000366307	0	0	0	0	0	0	0	0.769	0.769
CRP	IL1B	9606.ENSP00000255030	9606.ENSP00000263341	0	0	0	0	0.062	0	0	0.788	0.792

CRP	SELE	9606.ENSP000002 55030	9606.ENSP0000033 1736	0	0	0	0	0.063	0	0	0.788	0.792
CRP	MMP9	9606.ENSP000002 55030	9606.ENSP0000036 1405	0	0	0	0	0	0	0	0.808	0.808
CRP	VEGFA	9606.ENSP000002 55030	9606.ENSP0000047 8570	0	0	0	0	0	0	0	0.839	0.839
CRP	NOS3	9606.ENSP000002 55030	9606.ENSP0000029 7494	0	0	0	0	0	0	0	0.861	0.861
CRP	IL10	9606.ENSP000002 55030	9606.ENSP0000041 2237	0	0	0	0	0	0	0	0.873	0.873
CRP	VCAM1	9606.ENSP000002 55030	9606.ENSP0000029 4728	0	0	0	0	0	0	0	0.873	0.873
CRP	ICAM1	9606.ENSP000002 55030	9606.ENSP0000026 4832	0	0	0	0	0	0	0	0.875	0.875
CRP	F3	9606.ENSP000002 55030	9606.ENSP0000033 4145	0	0	0	0	0	0	0	0.879	0.879
CRP	CXCL8	9606.ENSP000002 55030	9606.ENSP0000030 6512	0	0	0	0	0	0	0	0.909	0.909

CRP	IL6	9606.ENSP000002 55030	9606.ENSP0000038 5675	0	0	0	0	0	0	0	0.97	0.97
CTNNB1	MAPK1	9606.ENSP000003 44456	9606.ENSP0000021 5832	0	0	0	0	0.065	0.063	0	0.512	0.536
CTNNB1	MMP2	9606.ENSP000003 44456	9606.ENSP0000021 9070	0	0	0	0	0	0.095	0	0.581	0.604
CTNNB1	SERPINE1	9606.ENSP000003 44456	9606.ENSP0000022 3095	0	0	0	0	0	0	0	0.571	0.571
CTNNB1	MAPK14	9606.ENSP000003 44456	9606.ENSP0000022 9795	0	0	0	0	0.064	0.063	0.9	0.335	0.933
CTNNB1	HSPB1	9606.ENSP000003 44456	9606.ENSP0000024 8553	0	0	0	0	0	0	0	0.532	0.532
CTNNB1	KDR	9606.ENSP000003 44456	9606.ENSP0000026 3923	0	0	0	0	0	0.384	0.9	0.519	0.967
CTNNB1	EGF	9606.ENSP000003 44456	9606.ENSP0000026 5171	0	0	0	0	0	0.064	0.9	0.785	0.978
CTNNB1	ERBB2	9606.ENSP000003 44456	9606.ENSP0000026 9571	0	0	0	0	0	0.472	0.9	0.815	0.989

CTNNB1	EGFR	9606.ENSP000003 44456	9606.ENSP0000027 5493	0	0	0	0	0.062	0.472	0.9	0.832	0.99
CTNNB1	GJA1	9606.ENSP000003 44456	9606.ENSP0000028 2561	0	0	0	0	0.073	0.261	0.9	0.546	0.964
CTNNB1	PPARG	9606.ENSP000003 44456	9606.ENSP0000028 7820	0	0	0	0	0	0.385	0	0.754	0.842
CTNNB1	VCAM1	9606.ENSP000003 44456	9606.ENSP0000029 4728	0	0	0	0	0	0	0	0.544	0.544
CTNNB1	NOS3	9606.ENSP000003 44456	9606.ENSP0000029 7494	0	0	0	0	0.064	0.379	0	0.321	0.57
CTNNB1	MMP3	9606.ENSP000003 44456	9606.ENSP0000029 9855	0	0	0	0	0	0.095	0.9	0.417	0.942
CTNNB1	FOS	9606.ENSP000003 44456	9606.ENSP0000030 6245	0	0	0	0	0	0	0.9	0.447	0.942
CTNNB1	CXCL8	9606.ENSP000003 44456	9606.ENSP0000030 6512	0	0	0	0	0	0	0	0.4	0.4
CTNNB1	HAS2	9606.ENSP000003 44456	9606.ENSP0000030 6991	0.1	0	0	0	0.049	0	0	0.516	0.529

CTNNB1	MET	9606. ENSP0 00003 44456	9606.E NSP00 00031 7272	0	0	0	0	0.061	0.444	0.9	0.605	0.976
CTNNB1	GSK3B	9606. ENSP0 00003 44456	9606.E NSP00 00032 4806	0	0	0	0	0.061	0.658	0.9	0.946	0.998
CTNNB1	PGR	9606. ENSP0 00003 44456	9606.E NSP00 00032 5120	0	0	0	0	0	0.051	0	0.494	0.499
CTNNB1	ESR2	9606. ENSP0 00003 44456	9606.E NSP00 00034 3925	0	0	0	0	0	0.07	0	0.398	0.416
CTNNB1	IL6	9606. ENSP0 00003 44456	9606.E NSP00 00038 5675	0	0	0	0	0	0	0	0.484	0.484
CTNNB1	SPP1	9606. ENSP0 00003 44456	9606.E NSP00 00037 8517	0	0	0	0	0	0	0	0.511	0.511
CTNNB1	PARP1	9606. ENSP0 00003 44456	9606.E NSP00 00035 5759	0	0	0	0	0	0.369	0	0.282	0.527
CTNNB1	PRKCA	9606. ENSP0 00003 44456	9606.E NSP00 00040 8695	0	0	0	0	0	0.379	0	0.349	0.578
CTNNB1	RELA	9606. ENSP0 00003 44456	9606.E NSP00 00038 4273	0	0	0	0	0	0.472	0	0.318	0.624

CTNNB1	MMP9	9606.ENSP00000344456	9606.ENSP00000361405	0	0	0	0	0	0.095	0	0.611	0.633
CTNNB1	IKBKB	9606.ENSP00000344456	9606.ENSP00000430684	0	0	0	0	0.058	0.715	0	0.249	0.781
CTNNB1	RUNX2	9606.ENSP00000344456	9606.ENSP00000360493	0	0	0	0	0	0	0	0.793	0.793
CTNNB1	HIF1A	9606.ENSP00000344456	9606.ENSP00000437955	0	0	0	0	0.078	0.379	0	0.67	0.795
CTNNB1	PTGS2	9606.ENSP00000344456	9606.ENSP00000356438	0	0	0	0	0	0.379	0	0.748	0.837
CTNNB1	ESR1	9606.ENSP00000344456	9606.ENSP00000405330	0	0	0	0	0	0.409	0	0.743	0.842
CTNNB1	MAPK8	9606.ENSP00000344456	9606.ENSP00000378974	0	0	0	0	0.101	0.525	0	0.798	0.906
CTNNB1	IGF2	9606.ENSP00000344456	9606.ENSP00000391826	0	0	0	0	0	0	0.9	0.511	0.949
CTNNB1	VEGFA	9606.ENSP00000344456	9606.ENSP00000478570	0	0	0	0	0	0	0.9	0.802	0.979

CTNNB1	JUN	9606.ENSP0000344456	9606.ENSP000360266	0	0	0	0	0	0.265	0.9	0.769	0.981
CTNNB1	MYC	9606.ENSP0000344456	9606.ENSP000479618	0	0	0	0	0	0.328	0.9	0.881	0.991
CTSD	MMP2	9606.ENSP0000236671	9606.ENSP000219070	0	0	0	0	0.051	0	0	0.457	0.462
CTSD	SERPINE1	9606.ENSP0000236671	9606.ENSP000223095	0	0	0	0	0	0	0	0.425	0.425
CTSD	IGF2	9606.ENSP0000236671	9606.ENSP000391826	0	0	0	0	0	0	0	0.4	0.4
CTSD	MYC	9606.ENSP0000236671	9606.ENSP000479618	0	0	0	0	0	0.104	0	0.422	0.459
CTSD	PLAU	9606.ENSP0000236671	9606.ENSP000361850	0	0	0	0	0.065	0	0	0.475	0.488
CTSD	HSPB1	9606.ENSP0000236671	9606.ENSP000248553	0	0	0	0	0.098	0	0	0.472	0.503
CTSD	EGFR	9606.ENSP0000236671	9606.ENSP000275493	0	0	0	0	0	0.099	0	0.495	0.526

CTSD	IGFBP3	9606.ENSP0000236671	9606.ENSP00000370473	0	0	0	0	0.065	0.379	0	0.284	0.547
CTSD	ERBB2	9606.ENSP0000236671	9606.ENSP00000269571	0	0	0	0	0	0	0	0.573	0.573
CTSD	PGR	9606.ENSP0000236671	9606.ENSP00000325120	0	0	0	0	0	0	0	0.63	0.63
CTSD	EGF	9606.ENSP0000236671	9606.ENSP00000265171	0	0	0	0	0	0	0	0.656	0.656
CTSD	HPSE	9606.ENSP0000236671	9606.ENSP00000384262	0	0	0	0	0	0	0.9	0.112	0.907
CTSD	SLPI	9606.ENSP0000236671	9606.ENSP00000342082	0	0	0	0	0.072	0	0.9	0.158	0.915
CTSD	LYZ	9606.ENSP0000236671	9606.ENSP00000261267	0	0	0	0	0.062	0	0.9	0.195	0.917
CTSD	MMP9	9606.ENSP0000236671	9606.ENSP00000361405	0	0	0	0	0.08	0	0.9	0.445	0.944
CTSD	ESR1	9606.ENSP0000236671	9606.ENSP00000405330	0	0	0	0	0.087	0	0.9	0.587	0.959

CXCL10	MAPK1	9606.ENSP00000305651	9606.ENSP00000215832	0	0	0	0	0	0	0	0.45	0.45
CXCL10	HMOX1	9606.ENSP00000305651	9606.ENSP00000216117	0	0	0	0	0.065	0	0	0.641	0.65
CXCL10	NFKBIA	9606.ENSP00000305651	9606.ENSP00000216797	0	0	0	0	0.097	0	0	0.422	0.455
CXCL10	MMP2	9606.ENSP00000305651	9606.ENSP00000219070	0	0	0	0	0	0	0	0.428	0.428
CXCL10	SERPINE1	9606.ENSP00000305651	9606.ENSP00000223095	0	0	0	0	0.063	0	0	0.425	0.438
CXCL10	MPO	9606.ENSP00000305651	9606.ENSP00000225275	0	0	0	0	0	0	0	0.477	0.477
CXCL10	IL2	9606.ENSP00000305651	9606.ENSP00000226730	0	0	0	0	0	0	0	0.788	0.788
CXCL10	IFNG	9606.ENSP00000305651	9606.ENSP00000229135	0	0	0	0	0.118	0	0	0.893	0.902
CXCL10	MAPK14	9606.ENSP00000305651	9606.ENSP00000229795	0	0	0	0	0	0	0	0.561	0.561

CXCL10	IL4	9606.ENSP0000305651	9606.ENSP00000231449	0	0	0	0	0	0	0	0.799	0.799
CXCL10	OPRD1	9606.ENSP0000305651	9606.ENSP00000234961	0	0	0	0	0	0	0.9	0.083	0.904
CXCL10	IRF1	9606.ENSP0000305651	9606.ENSP00000245414	0	0	0	0	0.382	0	0	0.675	0.79
CXCL10	IL1A	9606.ENSP0000305651	9606.ENSP00000263339	0	0	0	0	0.119	0	0	0.641	0.67
CXCL10	IL1B	9606.ENSP0000305651	9606.ENSP00000263341	0	0	0	0	0.244	0	0	0.841	0.876
CXCL10	ICAM1	9606.ENSP0000305651	9606.ENSP00000264832	0	0	0	0	0.145	0	0	0.7	0.732
CXCL10	EGF	9606.ENSP0000305651	9606.ENSP00000265171	0	0	0	0	0	0	0	0.559	0.559
CXCL10	VCAM1	9606.ENSP0000305651	9606.ENSP00000294728	0	0	0	0	0.097	0	0	0.668	0.687
CXCL10	MMP3	9606.ENSP0000305651	9606.ENSP00000299855	0	0	0	0	0.061	0	0	0.482	0.493

CXCL10	SPP1	9606.ENSP000003 05651	9606.ENSP0000037 8517	0	0	0	0	0.061	0	0	0.44	0.451
CXCL10	RELA	9606.ENSP000003 05651	9606.ENSP0000038 4273	0	0	0	0	0.06	0	0	0.441	0.452
CXCL10	MMP1	9606.ENSP000003 05651	9606.ENSP0000032 2788	0	0	0	0	0.065	0	0	0.44	0.454
CXCL10	JUN	9606.ENSP000003 05651	9606.ENSP0000036 0266	0	0	0	0	0.049	0	0	0.457	0.461
CXCL10	NOS2	9606.ENSP000003 05651	9606.ENSP0000032 7251	0	0	0	0	0.063	0	0	0.499	0.511
CXCL10	SELE	9606.ENSP000003 05651	9606.ENSP0000033 1736	0	0	0	0	0.076	0	0	0.549	0.565
CXCL10	PTGS2	9606.ENSP000003 05651	9606.ENSP0000035 6438	0	0	0	0	0.107	0	0	0.54	0.572
CXCL10	MAPK8	9606.ENSP000003 05651	9606.ENSP0000037 8974	0	0	0	0	0	0	0	0.698	0.698
CXCL10	VEGFA	9606.ENSP000003 05651	9606.ENSP0000047 8570	0	0	0	0	0	0	0	0.722	0.722

CXCL10	MMP9	9606.ENSP000003 05651	9606.ENSP0000036 1405	0	0	0	0	0.14	0	0	0.742	0.769
CXCL10	IL6	9606.ENSP000003 05651	9606.ENSP0000038 5675	0	0	0	0	0.18	0	0	0.865	0.884
CXCL10	DRD2	9606.ENSP000003 05651	9606.ENSP0000035 4859	0	0	0	0	0	0	0.9	0.064	0.902
CXCL10	OPRM1	9606.ENSP000003 05651	9606.ENSP0000039 4624	0	0	0	0	0	0	0.9	0.14	0.91
CXCL10	PTGER3	9606.ENSP000003 05651	9606.ENSP0000034 9003	0	0	0	0	0	0	0.9	0.186	0.915
CXCL10	STAT1	9606.ENSP000003 05651	9606.ENSP0000035 4394	0	0	0	0	0.707	0	0	0.84	0.951
CXCL10	CXCL2	9606.ENSP000003 05651	9606.ENSP0000042 7279	0	0	0	0	0.139	0	0.9	0.784	0.979
CXCL10	CXCL8	9606.ENSP000003 05651	9606.ENSP0000030 6512	0	0	0	0	0.144	0	0.9	0.869	0.987
CXCL10	CXCL11	9606.ENSP000003 05651	9606.ENSP0000030 6884	0	0	0	0.818	0.842	0.221	0.9	0.896	0.988

CXCL10	IL10	9606.ENSP0000305651	9606.ENSP0000412237	0	0	0	0	0.083	0	0.9	0.913	0.991
CXCL11	IL2	9606.ENSP0000306884	9606.ENSP0000226730	0	0	0	0	0	0	0	0.557	0.557
CXCL11	IFNG	9606.ENSP0000306884	9606.ENSP0000229135	0	0	0	0	0.116	0	0	0.913	0.92
CXCL11	IL4	9606.ENSP0000306884	9606.ENSP0000231449	0	0	0	0	0	0	0	0.575	0.575
CXCL11	OPRD1	9606.ENSP0000306884	9606.ENSP0000234961	0	0	0	0	0	0	0.9	0.053	0.901
CXCL11	IRF1	9606.ENSP0000306884	9606.ENSP0000245414	0	0	0	0	0.1	0	0	0.475	0.507
CXCL11	IL1A	9606.ENSP0000306884	9606.ENSP0000263339	0	0	0	0	0.09	0	0	0.456	0.483
CXCL11	IL1B	9606.ENSP0000306884	9606.ENSP0000263341	0	0	0	0	0.118	0	0	0.533	0.57
CXCL11	ICAM1	9606.ENSP0000306884	9606.ENSP0000264832	0	0	0	0	0.076	0	0	0.706	0.717

CXCL11	VCAM1	9606.ENSP000003 06884	9606.ENSP0000029 4728	0	0	0	0	0.065	0	0	0.475	0.488
CXCL11	CXCL8	9606.ENSP000003 06884	9606.ENSP0000030 6512	0	0	0	0	0.107	0	0.9	0.678	0.968
CXCL11	MMP1	9606.ENSP000003 06884	9606.ENSP0000032 2788	0	0	0	0	0.076	0	0	0.398	0.419
CXCL11	SELE	9606.ENSP000003 06884	9606.ENSP0000033 1736	0	0	0	0	0.07	0	0	0.443	0.459
CXCL11	MMP9	9606.ENSP000003 06884	9606.ENSP0000036 1405	0	0	0	0	0.088	0	0	0.443	0.47
CXCL11	VEGFA	9606.ENSP000003 06884	9606.ENSP0000047 8570	0	0	0	0	0	0	0	0.511	0.511
CXCL11	IL10	9606.ENSP000003 06884	9606.ENSP0000041 2237	0	0	0	0	0	0	0	0.641	0.641
CXCL11	STAT1	9606.ENSP000003 06884	9606.ENSP0000035 4394	0	0	0	0	0.284	0	0	0.572	0.68
CXCL11	IL6	9606.ENSP000003 06884	9606.ENSP0000038 5675	0	0	0	0	0.098	0	0	0.668	0.687

CXCL11	DRD2	9606.ENSP0000306884	9606.ENSP00000354859	0	0	0	0	0	0	0.9	0	0.9
CXCL11	OPRM1	9606.ENSP0000306884	9606.ENSP00000394624	0	0	0	0	0	0	0.9	0.053	0.901
CXCL11	PTGER3	9606.ENSP0000306884	9606.ENSP00000349003	0	0	0	0	0	0	0.9	0.116	0.907
CXCL11	CXCL2	9606.ENSP0000306884	9606.ENSP00000427279	0	0	0	0	0.107	0.232	0.9	0.717	0.978
CXCL2	NFKBIA	9606.ENSP0000427279	9606.ENSP00000216797	0	0	0	0	0.215	0	0	0.455	0.554
CXCL2	MPO	9606.ENSP0000427279	9606.ENSP00000225275	0	0	0	0	0	0	0	0.652	0.652
CXCL2	IL2	9606.ENSP0000427279	9606.ENSP00000226730	0	0	0	0	0	0	0	0.537	0.537
CXCL2	IFNG	9606.ENSP0000427279	9606.ENSP00000229135	0	0	0	0	0.077	0	0	0.564	0.581
CXCL2	IL4	9606.ENSP0000427279	9606.ENSP00000231449	0	0	0	0	0	0	0	0.607	0.607

CXCL2	OPRD1	9606.ENSP0000427279	9606.ENSP000234961	0	0	0	0	0	0	0.9	0.096	0.905
CXCL2	IRF1	9606.ENSP0000427279	9606.ENSP000245414	0	0	0	0	0.113	0	0	0.399	0.444
CXCL2	IL1A	9606.ENSP0000427279	9606.ENSP000263339	0	0	0	0	0.246	0	0	0.595	0.681
CXCL2	IL1B	9606.ENSP0000427279	9606.ENSP000263341	0	0	0	0	0.592	0	0	0.781	0.906
CXCL2	ICAM1	9606.ENSP0000427279	9606.ENSP000264832	0	0	0	0	0.169	0	0	0.62	0.671
CXCL2	EGF	9606.ENSP0000427279	9606.ENSP000265171	0	0	0	0	0	0	0	0.464	0.464
CXCL2	VCAM1	9606.ENSP0000427279	9606.ENSP000294728	0	0	0	0	0.061	0	0	0.588	0.596
CXCL2	MMP3	9606.ENSP0000427279	9606.ENSP000299855	0	0	0	0	0.063	0	0	0.452	0.464
CXCL2	FOS	9606.ENSP0000427279	9606.ENSP000306245	0	0	0	0	0.18	0	0	0.373	0.464

CXCL2	CXCL8	9606.ENSP0000427279	9606.ENSP00000306512	0	0	0	0.874	0.824	0	0.9	0.767	0.983
CXCL2	MMP1	9606.ENSP0000427279	9606.ENSP00000322788	0	0	0	0	0.097	0	0	0.388	0.424
CXCL2	NOS2	9606.ENSP0000427279	9606.ENSP00000327251	0	0	0	0	0.049	0	0	0.5	0.504
CXCL2	SELE	9606.ENSP0000427279	9606.ENSP00000331736	0	0	0	0	0.089	0	0	0.487	0.513
CXCL2	PTGER3	9606.ENSP0000427279	9606.ENSP00000349003	0	0	0	0	0	0	0.9	0.088	0.904
CXCL2	STAT1	9606.ENSP0000427279	9606.ENSP00000354394	0	0	0	0	0	0	0	0.499	0.499
CXCL2	DRD2	9606.ENSP0000427279	9606.ENSP00000354859	0	0	0	0	0	0	0.9	0	0.9
CXCL2	PTGS2	9606.ENSP0000427279	9606.ENSP00000356438	0	0	0	0	0.538	0	0	0.588	0.802
CXCL2	JUN	9606.ENSP0000427279	9606.ENSP00000360266	0	0	0	0	0.109	0	0.8	0.383	0.88

CXCL2	MMP9	9606.ENSP0000427279	9606.ENSP000361405	0	0	0	0	0.098	0	0	0.643	0.664
CXCL2	MAPK8	9606.ENSP0000427279	9606.ENSP000378974	0	0	0	0	0	0	0	0.419	0.419
CXCL2	RELA	9606.ENSP0000427279	9606.ENSP000384273	0	0	0	0	0.05	0.342	0.8	0.398	0.914
CXCL2	IL6	9606.ENSP0000427279	9606.ENSP000385675	0	0	0	0	0.631	0	0	0.825	0.932
CXCL2	OPRM1	9606.ENSP0000427279	9606.ENSP000394624	0	0	0	0	0	0	0.9	0.088	0.904
CXCL2	IL10	9606.ENSP0000427279	9606.ENSP000412237	0	0	0	0	0.064	0	0.9	0.801	0.979
CXCL2	VEGFA	9606.ENSP0000427279	9606.ENSP000478570	0	0	0	0	0	0	0	0.537	0.537
CXCL8	MAPK1	9606.ENSP0000306512	9606.ENSP000215832	0	0	0	0	0	0	0	0.842	0.842
CXCL8	HMOX1	9606.ENSP0000306512	9606.ENSP000216117	0	0	0	0	0.077	0	0	0.776	0.784

CXCL8	NFKBIA	9606.ENSP0000306512	9606.ENSP00000216797	0	0	0	0	0.272	0	0	0.67	0.749
CXCL8	MMP2	9606.ENSP0000306512	9606.ENSP00000219070	0	0	0	0	0	0	0	0.8	0.8
CXCL8	PLAT	9606.ENSP0000306512	9606.ENSP00000220809	0	0	0	0	0	0	0	0.433	0.433
CXCL8	SERPINE1	9606.ENSP0000306512	9606.ENSP00000223095	0	0	0	0	0.137	0	0	0.678	0.71
CXCL8	MPO	9606.ENSP0000306512	9606.ENSP00000225275	0	0	0	0	0.065	0	0	0.779	0.784
CXCL8	IL2	9606.ENSP0000306512	9606.ENSP00000226730	0	0	0	0	0	0	0	0.863	0.863
CXCL8	IFNG	9606.ENSP0000306512	9606.ENSP00000229135	0	0	0	0	0.062	0	0	0.884	0.887
CXCL8	MAPK14	9606.ENSP0000306512	9606.ENSP00000229795	0	0	0	0	0	0	0.9	0.757	0.974
CXCL8	IL4	9606.ENSP0000306512	9606.ENSP00000231449	0	0	0	0	0	0	0.9	0.927	0.992

CXCL8	NR3C1	9606.ENSP00000306512	9606.ENSP00000231509	0	0	0	0	0.062	0	0	0.531	0.541
CXCL8	OPRD1	9606.ENSP00000306512	9606.ENSP00000234961	0	0	0	0	0	0	0.9	0.25	0.921
CXCL8	IRF1	9606.ENSP00000306512	9606.ENSP00000245414	0	0	0	0	0.119	0	0	0.519	0.558
CXCL8	HSPB1	9606.ENSP00000306512	9606.ENSP00000248553	0	0	0	0	0	0	0	0.427	0.426
CXCL8	IL1A	9606.ENSP00000306512	9606.ENSP00000263339	0	0	0	0	0.232	0	0	0.869	0.895
CXCL8	IL1B	9606.ENSP00000306512	9606.ENSP00000263341	0	0	0	0	0.676	0	0	0.977	0.992
CXCL8	KDR	9606.ENSP00000306512	9606.ENSP00000263923	0	0	0	0	0	0	0	0.772	0.772
CXCL8	ICAM1	9606.ENSP00000306512	9606.ENSP00000264832	0	0	0	0	0.214	0	0	0.912	0.928
CXCL8	EGF	9606.ENSP00000306512	9606.ENSP00000265171	0	0	0	0	0	0	0	0.846	0.847

CXCL8	ERBB2	9606.ENSP00000306512	9606.ENSP000269571	0	0	0	0	0	0	0	0.53	0.53
CXCL8	EGFR	9606.ENSP00000306512	9606.ENSP000275493	0	0	0	0	0	0	0	0.817	0.817
CXCL8	MMP10	9606.ENSP00000306512	9606.ENSP000279441	0	0	0	0	0.13	0	0	0.48	0.529
CXCL8	PPARG	9606.ENSP00000306512	9606.ENSP000287820	0	0	0	0	0.055	0	0	0.537	0.543
CXCL8	VCAM1	9606.ENSP00000306512	9606.ENSP000294728	0	0	0	0	0.061	0	0	0.793	0.798
CXCL8	NOS3	9606.ENSP00000306512	9606.ENSP000297494	0	0	0	0	0.054	0	0	0.583	0.588
CXCL8	MMP3	9606.ENSP00000306512	9606.ENSP000299855	0	0	0	0	0.107	0	0	0.681	0.703
CXCL8	FOS	9606.ENSP00000306512	9606.ENSP000306245	0	0	0	0	0.077	0	0	0.739	0.749
CXCL8	CYP1A1	9606.ENSP00000306512	9606.ENSP000369050	0	0	0	0	0	0	0	0.413	0.412

CXCL8	NFE2L2	9606.ENSP0000306512	9606.ENSP00000380252	0	0	0	0	0	0	0	0.43	0.43
CXCL8	IGFBP3	9606.ENSP0000306512	9606.ENSP00000370473	0	0	0	0	0	0	0	0.43	0.43
CXCL8	MET	9606.ENSP0000306512	9606.ENSP00000317272	0	0	0	0	0	0	0	0.441	0.441
CXCL8	OLR1	9606.ENSP0000306512	9606.ENSP00000309124	0	0	0	0	0.089	0	0	0.418	0.447
CXCL8	PTGS1	9606.ENSP0000306512	9606.ENSP00000354612	0	0	0	0	0.098	0	0	0.43	0.464
CXCL8	ESR1	9606.ENSP0000306512	9606.ENSP00000405330	0	0	0	0	0	0	0	0.492	0.491
CXCL8	G6PD	9606.ENSP0000306512	9606.ENSP00000377192	0	0	0	0	0	0	0	0.509	0.509
CXCL8	HIF1A	9606.ENSP0000306512	9606.ENSP00000437955	0	0	0	0	0.07	0	0	0.508	0.522
CXCL8	NOS2	9606.ENSP0000306512	9606.ENSP00000327251	0	0	0	0	0.054	0	0	0.57	0.576

CXCL8	THBD	9606.<br>ENSP0<br>00003<br>06512	9606.E<br>NSP00<br>00036<br>6307	0	0	0	0	0.139	0	0	0.534	0.581
CXCL8	PLAU	9606.<br>ENSP0<br>00003<br>06512	9606.E<br>NSP00<br>00036<br>1850	0	0	0	0	0.143	0	0	0.54	0.589
CXCL8	SLPI	9606.<br>ENSP0<br>00003<br>06512	9606.E<br>NSP00<br>00034<br>2082	0	0	0	0	0.088	0	0	0.575	0.595
CXCL8	SPP1	9606.<br>ENSP0<br>00003<br>06512	9606.E<br>NSP00<br>00037<br>8517	0	0	0	0	0.082	0	0	0.581	0.599
CXCL8	GRM1	9606.<br>ENSP0<br>00003<br>06512	9606.E<br>NSP00<br>00035<br>4896	0	0	0	0	0	0	0.6	0.083	0.617
CXCL8	STAT1	9606.<br>ENSP0<br>00003<br>06512	9606.E<br>NSP00<br>00035<br>4394	0	0	0	0	0	0	0	0.661	0.661
CXCL8	F3	9606.<br>ENSP0<br>00003<br>06512	9606.E<br>NSP00<br>00033<br>4145	0	0	0	0	0	0	0	0.668	0.668
CXCL8	IKBKB	9606.<br>ENSP0<br>00003<br>06512	9606.E<br>NSP00<br>00043<br>0684	0	0	0	0	0	0	0	0.724	0.724
CXCL8	MAPK8	9606.<br>ENSP0<br>00003<br>06512	9606.E<br>NSP00<br>00037<br>8974	0	0	0	0	0	0	0	0.727	0.727

CXCL8	MYC	9606.ENSP00000306512	9606.ENSP00000479618	0	0	0	0	0.062	0	0	0.721	0.727
CXCL8	MMP1	9606.ENSP00000306512	9606.ENSP00000322788	0	0	0	0	0.19	0	0	0.705	0.751
CXCL8	SELE	9606.ENSP00000306512	9606.ENSP00000331736	0	0	0	0	0.049	0	0	0.788	0.789
CXCL8	DRD2	9606.ENSP00000306512	9606.ENSP00000354859	0	0	0	0	0	0	0.9	0.167	0.913
CXCL8	OPRM1	9606.ENSP00000306512	9606.ENSP00000394624	0	0	0	0	0	0	0.9	0.293	0.926
CXCL8	PTGER3	9606.ENSP00000306512	9606.ENSP00000349003	0	0	0	0	0	0	0.9	0.294	0.926
CXCL8	MMP9	9606.ENSP00000306512	9606.ENSP00000361405	0	0	0	0	0.134	0.379	0	0.885	0.933
CXCL8	VEGFA	9606.ENSP00000306512	9606.ENSP00000478570	0	0	0	0	0.065	0	0	0.953	0.954
CXCL8	PTGS2	9606.ENSP00000306512	9606.ENSP00000356438	0	0	0	0	0.753	0	0	0.839	0.958

CXCL8	JUN	9606.ENSP000003065 12	9606.ENSP000003602 66	0	0	0	0	0.095	0.294	0.8	0.799	0.971
CXCL8	IL6	9606.ENSP000003065 12	9606.ENSP000003856 75	0	0	0	0	0.581	0	0	0.966	0.985
CXCL8	RELA	9606.ENSP000003065 12	9606.ENSP000003842 73	0	0	0	0	0.065	0.342	0.9	0.817	0.987
CXCL8	IL10	9606.ENSP000003065 12	9606.ENSP000004122 37	0	0	0	0	0.061	0	0.9	0.962	0.996
CYP1A1	HMOX1	9606.ENSP000003690 50	9606.ENSP000002161 17	0	0	0	0	0.064	0	0	0.535	0.547
CYP1A1	PON1	9606.ENSP000003690 50	9606.ENSP000002223 81	0	0	0	0	0.055	0	0	0.428	0.436
CYP1A1	NR3C1	9606.ENSP000003690 50	9606.ENSP000002315 09	0	0	0	0	0	0.085	0	0.433	0.459
CYP1A1	SLC6A4	9606.ENSP000003690 50	9606.ENSP000002617 07	0	0	0	0	0	0	0	0.5	0.5
CYP1A1	GSTM1	9606.ENSP000003690 50	9606.ENSP000003114 69	0	0	0	0	0.061	0.077	0.65	0.892	0.962

CYP1A1	PGR	9606.ENSP000003690 50	9606.ENSP000003251 20	0	0	0	0	0.062	0.085	0	0.362	0.404
CYP1A1	CYP3A4	9606.ENSP000003690 50	9606.ENSP000003379 15	0	0	0.443	0.605	0.129	0	0.9	0.895	0.95
CYP1A1	ESR2	9606.ENSP000003690 50	9606.ENSP000003439 25	0	0	0	0	0.064	0.085	0	0.52	0.553
CYP1A1	PTGS2	9606.ENSP000003690 50	9606.ENSP000003564 38	0	0	0	0	0.055	0	0	0.495	0.502
CYP1A1	NR1I3	9606.ENSP000003690 50	9606.ENSP000003569 59	0	0	0	0	0.062	0.085	0	0.656	0.679
CYP1A1	JUN	9606.ENSP000003690 50	9606.ENSP000003602 66	0	0	0	0	0	0.128	0	0.347	0.406
CYP1A1	NFE2L2	9606.ENSP000003690 50	9606.ENSP000003802 52	0	0	0	0	0	0	0	0.466	0.466
CYP1A1	IL6	9606.ENSP000003690 50	9606.ENSP000003856 75	0	0	0	0	0	0	0	0.477	0.477
CYP1A1	ESR1	9606.ENSP000003690 50	9606.ENSP000004053 30	0	0	0	0	0.061	0.085	0	0.675	0.696

CYP1A1	POR	9606.ENSP0000369050	9606.ENSP000000419970	0	0	0	0	0.051	0.053	0	0.72	0.727
CYP1A1	GSTP1	9606.ENSP0000369050	9606.ENSP000000381607	0	0	0	0	0	0.077	0.65	0.738	0.908
CYP1A1	PPARA	9606.ENSP0000369050	9606.ENSP000000385523	0	0	0	0	0.063	0.085	0.9	0.359	0.937
CYP1A1	HSD3B2	9606.ENSP0000369050	9606.ENSP000000445122	0	0	0	0	0.05	0.132	0.9	0.341	0.938
CYP3A4	PON1	9606.ENSP0000337915	9606.ENSP000000222381	0	0	0	0	0.097	0	0	0.421	0.454
CYP3A4	NR3C1	9606.ENSP0000337915	9606.ENSP000000231509	0	0	0	0	0	0.085	0	0.546	0.567
CYP3A4	KDR	9606.ENSP0000337915	9606.ENSP000000263923	0	0	0	0	0	0.075	0	0.402	0.423
CYP3A4	EGFR	9606.ENSP0000337915	9606.ENSP000000275493	0	0	0	0	0	0	0	0.45	0.45
CYP3A4	GSTM1	9606.ENSP0000337915	9606.ENSP000000311469	0	0	0	0	0	0.064	0.65	0.555	0.841

CYP3A4	PGR	9606.ENSP00000337915	9606.ENSP00000325120	0	0	0	0	0	0.085	0	0.391	0.419
CYP3A4	IL6	9606.ENSP00000337915	9606.ENSP00000385675	0	0	0	0	0	0	0	0.421	0.42
CYP3A4	VEGFA	9606.ENSP00000337915	9606.ENSP00000478570	0	0	0	0	0	0	0	0.446	0.446
CYP3A4	HTR3A	9606.ENSP00000337915	9606.ENSP00000347754	0	0	0	0	0	0	0	0.475	0.475
CYP3A4	OPRM1	9606.ENSP00000337915	9606.ENSP00000394624	0	0	0	0	0	0	0	0.476	0.476
CYP3A4	PPARA	9606.ENSP00000337915	9606.ENSP00000385523	0	0	0	0	0.067	0.085	0	0.455	0.494
CYP3A4	G6PD	9606.ENSP00000337915	9606.ENSP00000377192	0	0	0	0	0	0	0	0.513	0.515
CYP3A4	ESR1	9606.ENSP00000337915	9606.ENSP00000405330	0	0	0	0	0.049	0.085	0	0.522	0.548
CYP3A4	NR1I3	9606.ENSP00000337915	9606.ENSP00000356959	0	0	0	0	0.061	0.085	0	0.808	0.821

CYP3A4	GSTP1	9606.ENSP00000337915	9606.ENSP000003816 07	0	0	0	0	0	0.064	0.65	0.551	0.84
CYP3A4	POR	9606.ENSP00000337915	9606.ENSP000004199 70	0	0.38	0	0	0	0.356	0	0.729	0.883
CYP3A4	HSD3B2	9606.ENSP00000337915	9606.ENSP000004451 22	0	0	0	0	0.064	0.132	0.9	0.268	0.932
DRD2	NR3C1	9606.ENSP00000354859	9606.ENSP000002315 09	0	0	0	0	0	0	0	0.448	0.448
DRD2	OPRD1	9606.ENSP00000354859	9606.ENSP000002349 61	0	0	0	0.589	0.062	0	0.9	0.71	0.93
DRD2	SLC6A4	9606.ENSP00000354859	9606.ENSP000002617 07	0	0	0	0	0.061	0.127	0	0.828	0.847
DRD2	GNRH1	9606.ENSP00000354859	9606.ENSP000002764 14	0	0	0	0	0	0	0	0.411	0.41
DRD2	FOS	9606.ENSP00000354859	9606.ENSP000003062 45	0	0	0	0	0	0	0	0.669	0.669
DRD2	HTR3A	9606.ENSP00000354859	9606.ENSP000003477 54	0	0	0	0	0.088	0.047	0	0.669	0.687

DRD2	PTGER3	9606.ENSP0000354859	9606.ENSP000003449003	0	0	0	0	0	0	0.9	0.23	0.919
DRD2	GRM1	9606.ENSP0000354859	9606.ENSP000003544896	0	0	0	0	0.087	0.057	0	0.564	0.592
DRD2	OPRM1	9606.ENSP0000354859	9606.ENSP000003944624	0	0	0	0.583	0	0	0.9	0.789	0.932
E2F1	ERBB2	9606.ENSP0000345571	9606.ENSP000002699571	0	0	0	0	0.061	0	0	0.405	0.417
E2F1	EGFR	9606.ENSP0000345571	9606.ENSP000002755493	0	0	0	0	0.061	0	0	0.501	0.511
E2F1	PPARG	9606.ENSP0000345571	9606.ENSP000002877820	0	0	0	0	0.063	0	0	0.845	0.849
E2F1	GSK3B	9606.ENSP0000345571	9606.ENSP000003244806	0	0	0	0	0	0.521	0	0.22	0.611
E2F1	VEGFA	9606.ENSP0000345571	9606.ENSP000004788570	0	0	0	0	0	0	0	0.599	0.599
E2F1	ESR1	9606.ENSP0000345571	9606.ENSP000004055330	0	0	0	0	0.062	0.379	0	0.413	0.628

E2F1	PARP1	9606.ENSP0000345571	9606.ENSP00000355759	0	0	0	0	0.096	0.472	0	0.525	0.754
E2F1	MYC	9606.ENSP0000345571	9606.ENSP00000479618	0	0	0	0	0.088	0	0	0.763	0.775
E2F1	E2F2	9606.ENSP0000345571	9606.ENSP00000355249	0	0	0	0.855	0.107	0.513	0.9	0.792	0.958
E2F2	IL6	9606.ENSP0000355249	9606.ENSP00000385675	0	0	0	0	0	0	0	0.507	0.507
E2F2	MYC	9606.ENSP0000355249	9606.ENSP00000479618	0	0	0	0	0.06	0	0	0.647	0.654
EGF	MAPK1	9606.ENSP0000265171	9606.ENSP00000215832	0	0	0	0	0	0	0	0.854	0.854
EGF	HMOX1	9606.ENSP0000265171	9606.ENSP00000216117	0	0	0	0	0	0	0	0.678	0.678
EGF	MMP2	9606.ENSP0000265171	9606.ENSP00000219070	0	0	0	0	0	0	0	0.797	0.798
EGF	PLAT	9606.ENSP0000265171	9606.ENSP00000220809	0	0	0	0	0.061	0	0	0.697	0.704

EGF	SERPINE1	9606.ENSP0000265171	9606.ENSP00000223095	0	0	0	0	0	0	0.9	0.798	0.978
EGF	MPO	9606.ENSP0000265171	9606.ENSP00000225275	0	0	0	0	0.061	0.082	0	0.401	0.438
EGF	IL2	9606.ENSP0000265171	9606.ENSP00000226730	0	0	0	0	0	0	0	0.641	0.641
EGF	IFNG	9606.ENSP0000265171	9606.ENSP00000229135	0	0	0	0	0	0	0	0.559	0.559
EGF	MAPK14	9606.ENSP0000265171	9606.ENSP00000229795	0	0	0	0	0	0	0	0.581	0.581
EGF	IL4	9606.ENSP0000265171	9606.ENSP00000231449	0	0	0	0	0	0	0	0.63	0.63
EGF	NR3C1	9606.ENSP0000265171	9606.ENSP00000231509	0	0	0	0	0	0	0	0.415	0.414
EGF	ODC1	9606.ENSP0000265171	9606.ENSP00000234111	0	0	0	0	0	0	0	0.719	0.719
EGF	HSPB1	9606.ENSP0000265171	9606.ENSP00000248553	0	0	0	0	0.064	0	0	0.399	0.413

EGF	SLC6A4	9606.ENSP0000265171	9606.ENSP000261707	0	0	0	0	0	0	0	0.491	0.491
EGF	IL1A	9606.ENSP0000265171	9606.ENSP000263339	0	0	0	0	0	0	0	0.534	0.534
EGF	IL1B	9606.ENSP0000265171	9606.ENSP000263341	0	0	0	0	0	0	0	0.743	0.743
EGF	KDR	9606.ENSP0000265171	9606.ENSP000263923	0	0	0	0	0	0.062	0.6	0.71	0.881
EGF	ICAM1	9606.ENSP0000265171	9606.ENSP000264832	0	0	0	0	0	0	0	0.732	0.732
EGF	RUNX2	9606.ENSP0000265171	9606.ENSP000360493	0	0	0	0	0	0	0	0.427	0.426
EGF	RELA	9606.ENSP0000265171	9606.ENSP000384273	0	0	0	0	0.061	0	0	0.422	0.434
EGF	F7	9606.ENSP0000265171	9606.ENSP000364731	0	0	0	0	0	0	0	0.462	0.461
EGF	ESR2	9606.ENSP0000265171	9606.ENSP000343925	0	0	0	0	0	0	0	0.473	0.473

EGF	HAS2	9606.ENSP0000265171	9606.ENSP00000306991	0	0	0	0	0.064	0	0	0.474	0.487
EGF	F3	9606.ENSP0000265171	9606.ENSP00000334145	0	0	0	0	0	0	0	0.49	0.489
EGF	MMP10	9606.ENSP0000265171	9606.ENSP00000279441	0	0	0	0	0	0	0	0.501	0.501
EGF	GSK3B	9606.ENSP0000265171	9606.ENSP00000324806	0	0	0	0	0	0.052	0	0.499	0.505
EGF	HIF1A	9606.ENSP0000265171	9606.ENSP00000437955	0	0	0	0	0	0	0	0.516	0.516
EGF	IGFBP3	9606.ENSP0000265171	9606.ENSP00000370473	0	0	0	0	0	0	0	0.53	0.53
EGF	G6PD	9606.ENSP0000265171	9606.ENSP00000377192	0	0	0	0	0	0	0	0.532	0.532
EGF	GNRH1	9606.ENSP0000265171	9606.ENSP00000276414	0	0	0	0	0.062	0	0	0.525	0.535
EGF	SELE	9606.ENSP0000265171	9606.ENSP00000331736	0	0	0	0	0	0	0	0.537	0.537

EGF	MMP3	9606.ENSP000002 65171	9606.ENSP0000029 9855	0	0	0	0	0	0	0	0.543	0.543
EGF	VCAM1	9606.ENSP000002 65171	9606.ENSP0000029 4728	0	0	0	0	0	0	0	0.547	0.547
EGF	SPP1	9606.ENSP000002 65171	9606.ENSP0000037 8517	0	0	0	0	0	0	0	0.559	0.559
EGF	NFE2L2	9606.ENSP000002 65171	9606.ENSP0000038 0252	0	0	0	0	0	0	0	0.561	0.561
EGF	SLPI	9606.ENSP000002 65171	9606.ENSP0000034 2082	0	0	0	0	0	0	0	0.609	0.609
EGF	PPARG	9606.ENSP000002 65171	9606.ENSP0000028 7820	0	0	0	0	0	0	0	0.65	0.65
EGF	SREBF1	9606.ENSP000002 65171	9606.ENSP0000034 8069	0	0	0	0	0	0.361	0	0.574	0.716
EGF	NOS3	9606.ENSP000002 65171	9606.ENSP0000029 7494	0	0	0	0	0	0	0	0.719	0.719
EGF	MAPK8	9606.ENSP000002 65171	9606.ENSP0000037 8974	0	0	0	0	0.062	0.099	0	0.702	0.726

EGF	PLAU	9606.ENSP00000265171	9606.ENSP000000361850	0	0	0	0	0	0	0	0.769	0.769
EGF	MMP1	9606.ENSP00000265171	9606.ENSP000000322788	0	0	0	0	0	0	0	0.77	0.771
EGF	IL10	9606.ENSP00000265171	9606.ENSP000000412237	0	0	0	0	0	0	0	0.796	0.796
EGF	GJA1	9606.ENSP00000265171	9606.ENSP000000282561	0	0	0	0	0.061	0	0	0.803	0.807
EGF	PGR	9606.ENSP00000265171	9606.ENSP000000325120	0	0	0	0	0	0	0	0.823	0.823
EGF	ESR1	9606.ENSP00000265171	9606.ENSP000000405330	0	0	0	0	0	0	0	0.836	0.836
EGF	JUN	9606.ENSP00000265171	9606.ENSP000000360266	0	0	0	0	0	0	0	0.837	0.837
EGF	IL6	9606.ENSP00000265171	9606.ENSP000000385675	0	0	0	0	0	0	0	0.844	0.844
EGF	FOS	9606.ENSP00000265171	9606.ENSP000000306245	0	0	0	0	0	0	0	0.862	0.863

EGF	PTGS2	9606.ENSP00000265171	9606.ENSP00000356438	0	0	0	0	0.061	0.082	0	0.856	0.866
EGF	MET	9606.ENSP00000265171	9606.ENSP00000317272	0	0	0	0	0	0.05	0.6	0.689	0.871
EGF	MMP9	9606.ENSP00000265171	9606.ENSP00000361405	0	0	0	0	0	0	0	0.871	0.871
EGF	MYC	9606.ENSP00000265171	9606.ENSP00000479618	0	0	0	0	0	0	0	0.876	0.877
EGF	PRKCA	9606.ENSP00000265171	9606.ENSP00000408695	0	0	0	0	0	0	0.9	0.473	0.945
EGF	STAT1	9606.ENSP00000265171	9606.ENSP00000354394	0	0	0	0	0	0	0.9	0.558	0.953
EGF	IGF2	9606.ENSP00000265171	9606.ENSP00000391826	0	0	0	0	0	0	0.9	0.672	0.965
EGF	VEGFA	9606.ENSP00000265171	9606.ENSP00000478570	0	0	0	0	0	0	0.9	0.937	0.993
EGF	ERBB2	9606.ENSP00000265171	9606.ENSP00000269571	0	0	0	0	0.16	0.406	0.9	0.94	0.996

EGF	EGFR	9606.ENSP0000265171	9606.ENSP00000275493	0	0	0	0	0.16	0.933	0.9	0.972	0.999
EGFR	MAPK1	9606.ENSP0000275493	9606.ENSP00000215832	0	0	0	0.577	0	0.472	0.9	0.887	0.965
EGFR	NFKBIA	9606.ENSP0000275493	9606.ENSP00000216797	0	0	0	0	0	0.176	0	0.389	0.475
EGFR	MMP2	9606.ENSP0000275493	9606.ENSP00000219070	0	0	0	0	0.11	0	0	0.673	0.697
EGFR	SERPINE1	9606.ENSP0000275493	9606.ENSP00000223095	0	0	0	0	0.125	0	0	0.687	0.715
EGFR	IL2	9606.ENSP0000275493	9606.ENSP00000226730	0	0	0	0	0	0	0.6	0.548	0.811
EGFR	IFNG	9606.ENSP0000275493	9606.ENSP00000229135	0	0	0	0	0	0	0.6	0.44	0.766
EGFR	MAPK14	9606.ENSP0000275493	9606.ENSP00000229795	0	0	0	0.577	0	0.723	0	0.751	0.81
EGFR	IL4	9606.ENSP0000275493	9606.ENSP00000231449	0	0	0	0	0.06	0	0.6	0.458	0.778

EGFR	NR3C1	9606.ENSP00000275493	9606.ENSP00000231509	0	0	0	0	0	0.415	0	0.414	0.643
EGFR	IRF1	9606.ENSP00000275493	9606.ENSP00000245414	0	0	0	0	0	0	0	0.593	0.593
EGFR	HSPB1	9606.ENSP00000275493	9606.ENSP00000248553	0	0	0	0	0.089	0.692	0	0.671	0.899
EGFR	IL1B	9606.ENSP00000275493	9606.ENSP00000263341	0	0	0	0	0	0	0	0.415	0.414
EGFR	KDR	9606.ENSP00000275493	9606.ENSP00000263923	0	0	0	0.559	0.074	0.177	0	0.914	0.525
EGFR	TP63	9606.ENSP00000275493	9606.ENSP00000264731	0	0	0	0	0	0	0	0.502	0.502
EGFR	ICAM1	9606.ENSP00000275493	9606.ENSP00000264832	0	0	0	0	0	0.112	0	0.502	0.539
EGFR	ERBB2	9606.ENSP00000275493	9606.ENSP00000269571	0	0	0	0.941	0.077	0.699	0.9	0.967	0.971
EGFR	HAS2	9606.ENSP00000275493	9606.ENSP00000306991	0	0	0	0	0.076	0	0	0.397	0.419

EGFR	SLPI	9606.ENSP0000275493	9606.ENSP00000342082	0	0	0	0	0.076	0	0	0.402	0.423
EGFR	NFE2L2	9606.ENSP0000275493	9606.ENSP00000380252	0	0	0	0	0	0	0	0.44	0.44
EGFR	F3	9606.ENSP0000275493	9606.ENSP00000334145	0	0	0	0	0.11	0	0	0.4	0.443
EGFR	MMP10	9606.ENSP0000275493	9606.ENSP00000279441	0	0	0	0	0	0	0	0.444	0.444
EGFR	NOS3	9606.ENSP0000275493	9606.ENSP00000297494	0	0	0	0	0	0	0	0.45	0.45
EGFR	VCAM1	9606.ENSP0000275493	9606.ENSP00000294728	0	0	0	0	0.076	0	0	0.444	0.464
EGFR	GJA1	9606.ENSP0000275493	9606.ENSP00000282561	0	0	0	0	0.158	0	0	0.389	0.464
EGFR	PPP3CA	9606.ENSP0000275493	9606.ENSP00000378323	0	0	0	0	0.062	0.064	0	0.446	0.471
EGFR	GNRH1	9606.ENSP0000275493	9606.ENSP00000276414	0	0	0	0	0	0	0	0.473	0.473

EGFR	RELA	9606.ENSP00000275493	9606.ENSP00000384273	0	0	0	0	0	0	0	0.473	0.473
EGFR	G6PD	9606.ENSP00000275493	9606.ENSP00000377192	0	0	0	0	0	0	0	0.494	0.494
EGFR	MMP1	9606.ENSP00000275493	9606.ENSP00000322788	0	0	0	0	0.078	0	0	0.522	0.541
EGFR	PARP1	9606.ENSP00000275493	9606.ENSP00000355759	0	0	0	0	0	0.099	0	0.546	0.573
EGFR	RUNX2	9606.ENSP00000275493	9606.ENSP00000360493	0	0	0	0	0	0	0	0.587	0.587
EGFR	MAPK8	9606.ENSP00000275493	9606.ENSP00000378974	0	0	0	0.563	0	0.393	0	0.764	0.593
EGFR	NOX1	9606.ENSP00000275493	9606.ENSP00000362057	0	0	0	0	0	0	0	0.596	0.597
EGFR	GSTP1	9606.ENSP00000275493	9606.ENSP00000381607	0	0	0	0	0.068	0.379	0	0.419	0.634
EGFR	SPP1	9606.ENSP00000275493	9606.ENSP00000378517	0	0	0	0	0	0	0	0.694	0.694

EGFR	MET	9606.ENSP00000275493	9606.ENSP00000317272	0	0	0	0.58	0.229	0.418	0	0.933	0.715
EGFR	ESR2	9606.ENSP00000275493	9606.ENSP00000343925	0	0	0	0	0	0.077	0	0.739	0.749
EGFR	PPARG	9606.ENSP00000275493	9606.ENSP00000287820	0	0	0	0	0.062	0.402	0	0.65	0.787
EGFR	PGR	9606.ENSP00000275493	9606.ENSP00000325120	0	0	0	0	0	0.077	0	0.804	0.811
EGFR	MMP9	9606.ENSP00000275493	9606.ENSP00000361405	0	0	0	0	0	0	0	0.828	0.828
EGFR	IGF2	9606.ENSP00000275493	9606.ENSP00000391826	0	0	0	0	0.088	0	0.6	0.579	0.833
EGFR	IGFBP3	9606.ENSP00000275493	9606.ENSP00000370473	0	0	0	0	0.278	0.379	0	0.72	0.863
EGFR	PTGS2	9606.ENSP00000275493	9606.ENSP00000356438	0	0	0	0	0.061	0	0	0.869	0.872
EGFR	MYC	9606.ENSP00000275493	9606.ENSP00000479618	0	0	0	0	0	0	0	0.871	0.872

EGFR	IL10	9606.ENSP00000275493	9606.ENSP00000412237	0	0	0	0	0	0	0.6	0.713	0.88
EGFR	JUN	9606.ENSP00000275493	9606.ENSP00000360266	0	0	0	0	0.06	0.185	0	0.856	0.88
EGFR	FOS	9606.ENSP00000275493	9606.ENSP00000306245	0	0	0	0	0	0.533	0	0.794	0.9
EGFR	IL6	9606.ENSP00000275493	9606.ENSP00000385675	0	0	0	0	0	0	0.6	0.811	0.921
EGFR	ESR1	9606.ENSP00000275493	9606.ENSP00000405330	0	0	0	0	0	0.402	0	0.908	0.943
EGFR	PLAU	9606.ENSP00000275493	9606.ENSP00000361850	0	0	0	0	0.088	0	0.9	0.513	0.951
EGFR	MMP3	9606.ENSP00000275493	9606.ENSP00000299855	0	0	0	0	0.076	0	0.9	0.543	0.954
EGFR	PRKCA	9606.ENSP00000275493	9606.ENSP00000408695	0	0	0	0.557	0.049	0.44	0.9	0.676	0.959
EGFR	HIF1A	9606.ENSP00000275493	9606.ENSP00000437955	0	0	0	0	0	0	0.9	0.743	0.973

EGFR	VEGFA	9606.ENSP00000275493	9606.ENSP00000478570	0	0	0	0	0.089	0	0.6	0.947	0.979
EGFR	STAT1	9606.ENSP00000275493	9606.ENSP00000354394	0	0	0	0	0.061	0.42	0.9	0.764	0.985
ELK1	MAPK1	9606.ENSP00000483056	9606.ENSP00000215832	0	0	0	0	0	0.463	0.9	0.158	0.95
ELK1	MAPK14	9606.ENSP00000483056	9606.ENSP00000229795	0	0	0	0	0	0.456	0.9	0.043	0.943
ELK1	FOS	9606.ENSP00000483056	9606.ENSP00000306245	0	0	0	0	0	0.379	0.9	0.532	0.968
ELK1	JUN	9606.ENSP00000483056	9606.ENSP00000360266	0	0	0	0	0	0.193	0.9	0.126	0.923
ELK1	MAPK8	9606.ENSP00000483056	9606.ENSP00000378974	0	0	0	0	0	0.379	0.8	0.135	0.883
ELK1	PRKCA	9606.ENSP00000483056	9606.ENSP00000408695	0	0	0	0	0	0	0.9	0.187	0.915
ERBB2	MAPK1	9606.ENSP00000269571	9606.ENSP00000215832	0	0	0	0.575	0	0.185	0	0.738	0.436

ERBB2	MMP2	9606.ENSP00000269571	9606.ENSP00000219070	0	0	0	0	0.062	0	0	0.752	0.758
ERBB2	SERPINE1	9606.ENSP00000269571	9606.ENSP00000223095	0	0	0	0	0.061	0	0	0.475	0.486
ERBB2	IL2	9606.ENSP00000269571	9606.ENSP00000226730	0	0	0	0	0	0	0	0.584	0.584
ERBB2	IFNG	9606.ENSP00000269571	9606.ENSP00000229135	0	0	0	0	0	0	0	0.427	0.426
ERBB2	IL4	9606.ENSP00000269571	9606.ENSP00000231449	0	0	0	0	0.061	0	0	0.43	0.441
ERBB2	HSPB1	9606.ENSP00000269571	9606.ENSP00000248553	0	0	0	0	0.062	0	0	0.451	0.463
ERBB2	KDR	9606.ENSP00000269571	9606.ENSP00000263923	0	0	0	0.556	0.051	0.177	0	0.772	0.461
ERBB2	TP63	9606.ENSP00000269571	9606.ENSP00000264731	0	0	0	0	0	0	0	0.418	0.418
ERBB2	ICAM1	9606.ENSP00000269571	9606.ENSP00000264832	0	0	0	0	0	0	0	0.419	0.418

ERBB2	GSTP1	9606.ENSP00000269571	9606.ENSP00000381607	0	0	0	0	0.062	0	0	0.4	0.413
ERBB2	SPP1	9606.ENSP00000269571	9606.ENSP00000378517	0	0	0	0	0	0	0	0.428	0.428
ERBB2	GNRH1	9606.ENSP00000269571	9606.ENSP00000276414	0	0	0	0	0	0	0	0.429	0.429
ERBB2	IL10	9606.ENSP00000269571	9606.ENSP00000412237	0	0	0	0	0	0	0	0.447	0.447
ERBB2	MET	9606.ENSP00000269571	9606.ENSP00000317272	0	0	0	0.579	0.091	0.102	0	0.837	0.447
ERBB2	PPP3CA	9606.ENSP00000269571	9606.ENSP00000378323	0	0	0	0	0.062	0.064	0	0.433	0.458
ERBB2	IGFBP3	9606.ENSP00000269571	9606.ENSP00000370473	0	0	0	0	0.084	0	0	0.45	0.474
ERBB2	PLAU	9606.ENSP00000269571	9606.ENSP00000361850	0	0	0	0	0.055	0	0	0.491	0.498
ERBB2	PARP1	9606.ENSP00000269571	9606.ENSP00000355759	0	0	0	0	0	0	0	0.519	0.519

ERBB2	HIF1A	9606.ENSP000002 69571	9606.ENSP0000043 7955	0	0	0	0	0	0	0	0.539	0.539
ERBB2	FOS	9606.ENSP000002 69571	9606.ENSP0000030 6245	0	0	0	0	0	0.082	0	0.523	0.543
ERBB2	PPARG	9606.ENSP000002 69571	9606.ENSP0000028 7820	0	0	0	0	0	0.077	0	0.58	0.596
ERBB2	NFE2L2	9606.ENSP000002 69571	9606.ENSP0000038 0252	0	0	0	0	0	0	0	0.61	0.61
ERBB2	ESR2	9606.ENSP000002 69571	9606.ENSP0000034 3925	0	0	0	0	0	0.077	0	0.659	0.671
ERBB2	JUN	9606.ENSP000002 69571	9606.ENSP0000036 0266	0	0	0	0	0.059	0.185	0	0.65	0.708
ERBB2	MMP1	9606.ENSP000002 69571	9606.ENSP0000032 2788	0	0	0	0	0	0	0	0.721	0.721
ERBB2	MMP9	9606.ENSP000002 69571	9606.ENSP0000036 1405	0	0	0	0	0	0	0	0.78	0.781
ERBB2	IGF2	9606.ENSP000002 69571	9606.ENSP0000039 1826	0	0	0	0	0.061	0	0.6	0.511	0.8

ERBB2	PTGS2	9606.ENSP000002 69571	9606.ENSP000003 56438	0	0	0	0	0.061	0.305	0	0.75	0.822
ERBB2	MYC	9606.ENSP000002 69571	9606.ENSP000004 79618	0	0	0	0	0.06	0.139	0	0.88	0.895
ERBB2	STAT1	9606.ENSP000002 69571	9606.ENSP000003 54394	0	0	0	0	0.061	0.696	0	0.711	0.91
ERBB2	ESR1	9606.ENSP000002 69571	9606.ENSP000004 05330	0	0	0	0	0	0.32	0	0.926	0.947
ERBB2	PGR	9606.ENSP000002 69571	9606.ENSP000003 25120	0	0	0	0	0	0.36	0	0.931	0.954
ERBB2	VEGFA	9606.ENSP000002 69571	9606.ENSP000004 78570	0	0	0	0	0	0	0.6	0.895	0.956
ERBB2	IL6	9606.ENSP000002 69571	9606.ENSP000003 85675	0	0	0	0	0	0	0.9	0.739	0.972
ERBB2	MAPK8	9606.ENSP000002 69571	9606.ENSP000003 78974	0	0	0	0	0	0	0.9	0.788	0.978
ESR1	MAPK1	9606.ENSP000004 05330	9606.ENSP000002 15832	0	0	0	0	0	0.399	0.9	0.57	0.971

ESR1	MMP2	9606.ENSP00000405330	9606.ENSP00000219070	0	0	0	0	0	0	0	0.532	0.532
ESR1	SERPINE1	9606.ENSP00000405330	9606.ENSP00000223095	0	0	0	0	0	0.051	0	0.45	0.455
ESR1	MAPK14	9606.ENSP00000405330	9606.ENSP00000229795	0	0	0	0	0	0.399	0.9	0.421	0.962
ESR1	NR3C1	9606.ENSP00000405330	9606.ENSP00000231509	0	0	0	0.66	0	0	0.9	0.751	0.925
ESR1	ODC1	9606.ENSP00000405330	9606.ENSP00000234111	0	0	0	0	0	0	0	0.432	0.432
ESR1	HSPB1	9606.ENSP00000405330	9606.ENSP00000248553	0	0	0	0	0	0.336	0.9	0.414	0.957
ESR1	IL1B	9606.ENSP00000405330	9606.ENSP00000263341	0	0	0	0	0	0	0	0.44	0.44
ESR1	KDR	9606.ENSP00000405330	9606.ENSP00000263923	0	0	0	0	0	0.051	0	0.511	0.516
ESR1	SOD1	9606.ENSP00000405330	9606.ENSP00000270142	0	0	0	0	0.061	0	0	0.586	0.595

ESR1	GNRH1	9606.ENSP000004 05330	9606.ENSP0000027 6414	0	0	0	0	0	0	0	0.757	0.757
ESR1	GJA1	9606.ENSP000004 05330	9606.ENSP0000028 2561	0	0	0	0	0	0	0	0.614	0.614
ESR1	NOS3	9606.ENSP000004 05330	9606.ENSP0000029 7494	0	0	0	0	0	0.472	0.9	0.749	0.985
ESR1	MMP3	9606.ENSP000004 05330	9606.ENSP0000029 9855	0	0	0	0	0	0	0	0.426	0.426
ESR1	FOS	9606.ENSP000004 05330	9606.ENSP0000030 6245	0	0	0	0	0	0.475	0.9	0.688	0.982
ESR1	MET	9606.ENSP000004 05330	9606.ENSP0000031 7272	0	0	0	0	0	0.085	0	0.448	0.473
ESR1	MMP1	9606.ENSP000004 05330	9606.ENSP0000032 2788	0	0	0	0	0	0	0	0.633	0.633
ESR1	GSK3B	9606.ENSP000004 05330	9606.ENSP0000032 4806	0	0	0	0	0	0.085	0	0.38	0.409
ESR1	PGR	9606.ENSP000004 05330	9606.ENSP0000032 5120	0	0	0	0.679	0.102	0.472	0.9	0.964	0.964

ESR1	ESR2	9606.ENSP00000405330	9606.ENSP00000343925	0	0	0	0.925	0.061	0.379	0.9	0.967	0.941
ESR1	SREBF1	9606.ENSP00000405330	9606.ENSP00000348069	0	0	0	0	0	0.379	0	0.389	0.604
ESR1	STAT1	9606.ENSP00000405330	9606.ENSP00000354394	0	0	0	0	0	0.448	0.9	0.458	0.967
ESR1	PARP1	9606.ENSP00000405330	9606.ENSP00000355759	0	0	0	0	0	0.342	0	0.652	0.761
ESR1	PTGS2	9606.ENSP00000405330	9606.ENSP00000356438	0	0	0	0	0	0.05	0	0.771	0.773
ESR1	JUN	9606.ENSP00000405330	9606.ENSP00000360266	0	0	0	0	0	0.472	0.9	0.646	0.979
ESR1	RUNX2	9606.ENSP00000405330	9606.ENSP00000360493	0	0	0	0	0	0.185	0	0.707	0.751
ESR1	MMP9	9606.ENSP00000405330	9606.ENSP00000361405	0	0	0	0	0	0	0	0.581	0.581
ESR1	PLAU	9606.ENSP00000405330	9606.ENSP00000361850	0	0	0	0	0.061	0	0	0.44	0.451

ESR1	IGFBP3	9606.<br>ENSP0<br>00004<br>05330	9606.E<br>NSP00<br>00037<br>0473	0	0	0	0	0	0	0	0.53	0.53
ESR1	SPP1	9606.<br>ENSP0<br>00004<br>05330	9606.E<br>NSP00<br>00037<br>8517	0	0	0	0	0	0	0	0.477	0.477
ESR1	MAPK8	9606.<br>ENSP0<br>00004<br>05330	9606.E<br>NSP00<br>00037<br>8974	0	0	0	0	0	0	0.8	0.559	0.908
ESR1	GSTP1	9606.<br>ENSP0<br>00004<br>05330	9606.E<br>NSP00<br>00038<br>1607	0	0	0	0	0	0.064	0	0.552	0.562
ESR1	RELA	9606.<br>ENSP0<br>00004<br>05330	9606.E<br>NSP00<br>00038<br>4273	0	0	0	0	0	0.393	0	0.473	0.666
ESR1	IL6	9606.<br>ENSP0<br>00004<br>05330	9606.E<br>NSP00<br>00038<br>5675	0	0	0	0	0	0	0	0.769	0.769
ESR1	IGF2	9606.<br>ENSP0<br>00004<br>05330	9606.E<br>NSP00<br>00039<br>1826	0	0	0	0	0	0	0	0.556	0.556
ESR1	IL10	9606.<br>ENSP0<br>00004<br>05330	9606.E<br>NSP00<br>00041<br>2237	0	0	0	0	0	0	0	0.422	0.422
ESR1	HIF1A	9606.<br>ENSP0<br>00004<br>05330	9606.E<br>NSP00<br>00043<br>7955	0	0	0	0	0	0.413	0	0.695	0.813

ESR1	VEGFA	9606.ENSP000004 05330	9606.ENSP0000047 8570	0	0	0	0	0.061	0	0	0.813	0.816
ESR1	MYC	9606.ENSP000004 05330	9606.ENSP0000047 9618	0	0	0	0	0	0.442	0.9	0.721	0.983
ESR2	MAPK1	9606.ENSP000003 43925	9606.ENSP0000021 5832	0	0	0	0	0	0.399	0.8	0.351	0.915
ESR2	MAPK14	9606.ENSP000003 43925	9606.ENSP0000022 9795	0	0	0	0	0	0.073	0.8	0.167	0.832
ESR2	GNRH1	9606.ENSP000003 43925	9606.ENSP0000027 6414	0	0	0	0	0	0	0	0.657	0.657
ESR2	NOS3	9606.ENSP000003 43925	9606.ENSP0000029 7494	0	0	0	0	0	0.091	0.9	0.671	0.967
ESR2	FOS	9606.ENSP000003 43925	9606.ENSP0000030 6245	0	0	0	0	0	0.299	0.8	0.695	0.953
ESR2	HSD3B2	9606.ENSP000003 43925	9606.ENSP0000044 5122	0	0	0	0	0	0	0	0.411	0.411
ESR2	IL6	9606.ENSP000003 43925	9606.ENSP0000038 5675	0	0	0	0	0	0	0	0.421	0.42

ESR2	MYC	9606.ENSP00000343925	9606.ENSP00000479618	0	0	0	0	0	0	0	0.429	0.429
ESR2	SREBF1	9606.ENSP00000343925	9606.ENSP00000348069	0	0	0	0	0	0.379	0	0.123	0.432
ESR2	PTGS2	9606.ENSP00000343925	9606.ENSP00000356438	0	0	0	0	0	0.05	0	0.439	0.444
ESR2	HIF1A	9606.ENSP00000343925	9606.ENSP00000437955	0	0	0	0	0	0.095	0	0.532	0.558
ESR2	VEGFA	9606.ENSP00000343925	9606.ENSP00000478570	0	0	0	0	0	0	0	0.671	0.671
ESR2	MAPK8	9606.ENSP00000343925	9606.ENSP00000378974	0	0	0	0	0.061	0	0.8	0.268	0.85
ESR2	JUN	9606.ENSP00000343925	9606.ENSP00000360266	0	0	0	0	0	0.082	0.8	0.412	0.882
F3	MMP2	9606.ENSP00000334145	9606.ENSP00000219070	0	0	0	0	0.061	0	0	0.408	0.42
F3	PLAT	9606.ENSP00000334145	9606.ENSP00000220809	0	0	0	0	0.076	0	0	0.788	0.795

F3	SERPINE1	9606.ENSP0000334145	9606.ENSP00000223095	0	0	0	0	0.091	0	0	0.821	0.83
F3	MPO	9606.ENSP0000334145	9606.ENSP00000225275	0	0	0	0	0	0	0	0.556	0.556
F3	IL2	9606.ENSP0000334145	9606.ENSP00000226730	0	0	0	0	0	0	0	0.426	0.426
F3	IFNG	9606.ENSP0000334145	9606.ENSP00000229135	0	0	0	0	0	0	0	0.417	0.416
F3	IL4	9606.ENSP0000334145	9606.ENSP00000231449	0	0	0	0	0	0	0	0.633	0.633
F3	IL1B	9606.ENSP0000334145	9606.ENSP00000263341	0	0	0	0	0.061	0	0	0.542	0.551
F3	KDR	9606.ENSP0000334145	9606.ENSP00000263923	0	0	0	0	0	0	0	0.435	0.435
F3	ICAM1	9606.ENSP0000334145	9606.ENSP00000264832	0	0	0	0	0	0	0	0.676	0.676
F3	VCAM1	9606.ENSP0000334145	9606.ENSP00000294728	0	0	0	0	0.061	0	0	0.681	0.688

F3	NOS3	9606.ENSP00000334145	9606.ENSP00000297494	0	0	0	0	0	0	0	0.557	0.557
F3	OLR1	9606.ENSP00000334145	9606.ENSP00000309124	0	0	0	0	0	0	0	0.41	0.41
F3	SELE	9606.ENSP00000334145	9606.ENSP00000331736	0	0	0	0	0	0	0	0.703	0.703
F3	NOX1	9606.ENSP00000334145	9606.ENSP00000362057	0	0	0	0	0	0	0	0.462	0.462
F3	PTGS2	9606.ENSP00000334145	9606.ENSP00000356438	0	0	0	0	0.085	0	0	0.441	0.466
F3	PPARA	9606.ENSP00000334145	9606.ENSP00000385523	0	0	0	0	0	0	0	0.515	0.515
F3	MMP9	9606.ENSP00000334145	9606.ENSP00000361405	0	0	0	0	0	0	0	0.532	0.532
F3	PLAU	9606.ENSP00000334145	9606.ENSP00000361850	0	0	0	0	0.076	0	0	0.535	0.551
F3	IL10	9606.ENSP00000334145	9606.ENSP00000412237	0	0	0	0	0	0	0	0.723	0.723

F3	IL6	9606.ENSP00000334145	9606.ENSP00000385675	0	0	0	0	0.088	0	0	0.797	0.807
F3	VEGFA	9606.ENSP00000334145	9606.ENSP00000478570	0	0	0	0	0.061	0	0	0.81	0.814
F3	THBD	9606.ENSP00000334145	9606.ENSP00000366307	0	0	0	0	0.061	0	0	0.86	0.862
F3	F7	9606.ENSP00000334145	9606.ENSP00000364731	0	0	0	0	0	0.901	0.9	0.967	0.999
F7	SERPINE1	9606.ENSP00000364731	9606.ENSP00000223095	0	0	0	0	0	0.129	0	0.788	0.807
F7	IL6	9606.ENSP00000364731	9606.ENSP00000385675	0	0	0	0	0	0	0	0.473	0.473
F7	THBD	9606.ENSP00000364731	9606.ENSP00000366307	0	0	0	0	0	0.157	0	0.668	0.708
FOS	MAPK1	9606.ENSP00000306245	9606.ENSP00000215832	0	0	0	0	0	0.523	0.9	0.852	0.992
FOS	HMOX1	9606.ENSP00000306245	9606.ENSP00000216117	0	0	0	0	0	0	0.9	0.482	0.946

FOS	NFKBIA	9606.ENSP00000306245	9606.ENSP00000216797	0	0	0	0	0.097	0.077	0	0.518	0.563
FOS	MMP2	9606.ENSP00000306245	9606.ENSP00000219070	0	0	0	0	0	0	0	0.521	0.521
FOS	SERPINE1	9606.ENSP00000306245	9606.ENSP00000223095	0	0	0	0	0.062	0	0	0.441	0.453
FOS	MPO	9606.ENSP00000306245	9606.ENSP00000225275	0	0	0	0	0	0	0	0.45	0.45
FOS	IL2	9606.ENSP00000306245	9606.ENSP00000226730	0	0	0	0	0	0.313	0	0.751	0.822
FOS	IFNG	9606.ENSP00000306245	9606.ENSP00000229135	0	0	0	0	0.049	0	0	0.441	0.445
FOS	MAPK14	9606.ENSP00000306245	9606.ENSP00000229795	0	0	0	0	0	0.264	0.9	0.733	0.978
FOS	IL4	9606.ENSP00000306245	9606.ENSP00000231449	0	0	0	0	0	0	0	0.726	0.726
FOS	NR3C1	9606.ENSP00000306245	9606.ENSP00000231509	0	0	0	0	0.055	0.299	0.9	0.634	0.972

FOS	ODC1	9606.ENSP0000306245	9606.ENSP00000234111	0	0	0	0	0	0	0	0.582	0.582
FOS	OPRD1	9606.ENSP0000306245	9606.ENSP00000234961	0	0	0	0	0	0	0	0.582	0.582
FOS	IRF1	9606.ENSP0000306245	9606.ENSP00000245414	0	0	0	0	0.063	0	0	0.473	0.485
FOS	HSPB1	9606.ENSP0000306245	9606.ENSP00000248553	0	0	0	0	0.061	0	0	0.406	0.419
FOS	SLC6A4	9606.ENSP0000306245	9606.ENSP00000261707	0	0	0	0	0	0	0	0.485	0.485
FOS	IL1A	9606.ENSP0000306245	9606.ENSP00000263339	0	0	0	0	0.062	0	0.9	0.425	0.941
FOS	IL1B	9606.ENSP0000306245	9606.ENSP00000263341	0	0	0	0	0.063	0	0	0.802	0.806
FOS	ICAM1	9606.ENSP0000306245	9606.ENSP00000264832	0	0	0	0	0	0	0	0.671	0.671
FOS	GNRH1	9606.ENSP0000306245	9606.ENSP00000276414	0	0	0	0	0	0	0	0.811	0.811

FOS	MMP10	9606.<br>ENSP0<br>00003<br>06245	9606.E<br>NSP00<br>00027<br>9441	0	0	0	0	0	0	0	0.564	0.564
FOS	GJA1	9606.<br>ENSP0<br>00003<br>06245	9606.E<br>NSP00<br>00028<br>2561	0	0	0	0	0	0	0	0.619	0.619
FOS	PPARG	9606.<br>ENSP0<br>00003<br>06245	9606.E<br>NSP00<br>00028<br>7820	0	0	0	0	0	0.098	0.9	0.683	0.968
FOS	NOS3	9606.<br>ENSP0<br>00003<br>06245	9606.E<br>NSP00<br>00029<br>7494	0	0	0	0	0	0	0	0.429	0.429
FOS	MMP3	9606.<br>ENSP0<br>00003<br>06245	9606.E<br>NSP00<br>00029<br>9855	0	0	0	0	0	0	0	0.476	0.476
FOS	PLAU	9606.<br>ENSP0<br>00003<br>06245	9606.E<br>NSP00<br>00036<br>1850	0	0	0	0	0	0	0	0.42	0.42
FOS	HIF1A	9606.<br>ENSP0<br>00003<br>06245	9606.E<br>NSP00<br>00043<br>7955	0	0	0	0	0	0	0	0.443	0.443
FOS	IGF2	9606.<br>ENSP0<br>00003<br>06245	9606.E<br>NSP00<br>00039<br>1826	0	0	0	0	0	0	0	0.451	0.451
FOS	SPP1	9606.<br>ENSP0<br>00003<br>06245	9606.E<br>NSP00<br>00037<br>8517	0	0	0	0	0	0	0	0.473	0.473

FOS	PGR	9606.ENSP0000306245	9606.ENSP00000325120	0	0	0	0	0	0.098	0	0.483	0.514
FOS	VEGFA	9606.ENSP0000306245	9606.ENSP00000478570	0	0	0	0	0	0	0	0.586	0.586
FOS	MMP9	9606.ENSP0000306245	9606.ENSP00000361405	0	0	0	0	0.062	0	0	0.644	0.652
FOS	RUNX2	9606.ENSP0000306245	9606.ENSP00000360493	0	0	0	0	0	0.294	0	0.53	0.653
FOS	HTR3A	9606.ENSP0000306245	9606.ENSP00000347754	0	0	0	0	0	0	0	0.711	0.712
FOS	IL10	9606.ENSP0000306245	9606.ENSP00000412237	0	0	0	0	0	0.063	0	0.743	0.748
FOS	OPRM1	9606.ENSP0000306245	9606.ENSP00000394624	0	0	0	0	0	0	0	0.792	0.792
FOS	PTGS2	9606.ENSP0000306245	9606.ENSP00000356438	0	0	0	0	0.109	0	0	0.802	0.817
FOS	MMP1	9606.ENSP0000306245	9606.ENSP00000322788	0	0	0	0	0	0.379	0	0.723	0.821

FOS	STAT1	9606.ENSP0000306245	9606.ENSP00000354394	0	0	0	0	0	0.52	0	0.762	0.881
FOS	PRKCA	9606.ENSP0000306245	9606.ENSP00000408695	0	0	0	0	0	0	0.8	0.473	0.89
FOS	POR	9606.ENSP0000306245	9606.ENSP00000419970	0	0	0	0	0	0	0.9	0.047	0.9
FOS	MYC	9606.ENSP0000306245	9606.ENSP00000479618	0	0	0	0	0.064	0.442	0	0.851	0.915
FOS	NFE2L2	9606.ENSP0000306245	9606.ENSP00000380252	0	0	0	0	0	0.051	0.9	0.426	0.94
FOS	RELA	9606.ENSP0000306245	9606.ENSP00000384273	0	0	0	0	0	0.379	0.9	0.539	0.968
FOS	IL6	9606.ENSP0000306245	9606.ENSP00000385675	0	0	0	0	0.088	0	0.9	0.819	0.982
FOS	MAPK8	9606.ENSP0000306245	9606.ENSP00000378974	0	0	0	0	0	0.26	0.9	0.885	0.99
FOS	JUN	9606.ENSP0000306245	9606.ENSP00000360266	0	0	0	0	0.656	0.879	0.9	0.975	0.999

G6PD	HMOX1	9606.ENSP0000377192	9606.ENSP00000216117	0	0	0	0	0.089	0	0	0.482	0.508
G6PD	ODC1	9606.ENSP0000377192	9606.ENSP00000234111	0	0	0	0	0.063	0.05	0	0.447	0.465
G6PD	HSPB1	9606.ENSP0000377192	9606.ENSP00000248553	0	0	0	0	0.076	0.379	0	0.58	0.738
G6PD	TP63	9606.ENSP0000377192	9606.ENSP00000264731	0	0	0	0	0	0	0	0.412	0.412
G6PD	SOD1	9606.ENSP0000377192	9606.ENSP00000270142	0	0	0	0	0.062	0	0	0.553	0.563
G6PD	NOS3	9606.ENSP0000377192	9606.ENSP00000297494	0.1	0	0	0	0.062	0	0	0.414	0.448
G6PD	SREBF1	9606.ENSP0000377192	9606.ENSP00000348069	0	0	0	0	0.076	0.09	0	0.724	0.748
G6PD	NFE2L2	9606.ENSP0000377192	9606.ENSP00000380252	0	0	0	0	0	0	0	0.418	0.418
G6PD	VEGFA	9606.ENSP0000377192	9606.ENSP00000478570	0	0	0	0	0	0	0	0.537	0.537

G6PD	POR	9606.ENSP0000377192	9606.ENSP00000419970	0.1	0	0	0	0.09	0	0	0.607	0.641
GJA1	MAPK1	9606.ENSP0000282561	9606.ENSP00000215832	0	0	0	0	0	0.379	0.8	0.45	0.925
GJA1	MMP2	9606.ENSP0000282561	9606.ENSP00000219070	0	0	0	0	0.228	0	0	0.418	0.531
GJA1	MAPK14	9606.ENSP0000282561	9606.ENSP00000229795	0	0	0	0	0	0	0	0.404	0.404
GJA1	IL1B	9606.ENSP0000282561	9606.ENSP00000263341	0	0	0	0	0	0	0	0.644	0.644
GJA1	KDR	9606.ENSP0000282561	9606.ENSP00000263923	0	0	0	0	0.188	0	0	0.42	0.509
GJA1	MYC	9606.ENSP0000282561	9606.ENSP00000479618	0	0	0	0	0.049	0	0	0.399	0.404
GJA1	SPP1	9606.ENSP0000282561	9606.ENSP00000378517	0	0	0	0	0.088	0	0	0.4	0.429
GJA1	RUNX2	9606.ENSP0000282561	9606.ENSP00000360493	0	0	0	0	0	0	0	0.443	0.443

GJA1	IL6	9606.ENSP00000282561	9606.ENSP000003856 75	0	0	0	0	0	0	0	0.452	0.452
GJA1	MMP9	9606.ENSP00000282561	9606.ENSP000003614 05	0	0	0	0	0	0	0	0.457	0.457
GJA1	NOS3	9606.ENSP00000282561	9606.ENSP000002974 94	0	0	0	0	0	0	0	0.492	0.491
GJA1	JUN	9606.ENSP00000282561	9606.ENSP000003602 66	0	0	0	0	0	0	0	0.625	0.625
GJA1	PTGS2	9606.ENSP00000282561	9606.ENSP000003564 38	0	0	0	0	0	0	0	0.65	0.65
GJA1	MAPK8	9606.ENSP00000282561	9606.ENSP000003789 74	0	0	0	0	0	0	0	0.656	0.656
GJA1	VEGFA	9606.ENSP00000282561	9606.ENSP000004785 70	0	0	0	0	0	0	0	0.755	0.755
GJA1	PRKCA	9606.ENSP00000282561	9606.ENSP000004086 95	0	0	0	0	0	0.551	0	0.59	0.808
GNRH1	MAPK1	9606.ENSP00000276414	9606.ENSP000002158 32	0	0	0	0	0	0	0	0.416	0.416

GNRH1	IL2	9606.ENSP0000276414	9606.ENSP00000226730	0	0	0	0	0	0	0	0.53	0.53
GNRH1	NR3C1	9606.ENSP0000276414	9606.ENSP00000231509	0	0	0	0	0	0	0	0.46	0.46
GNRH1	OPRD1	9606.ENSP0000276414	9606.ENSP00000234961	0	0	0	0	0	0	0	0.447	0.447
GNRH1	IL1B	9606.ENSP0000276414	9606.ENSP00000263341	0	0	0	0	0	0	0	0.558	0.558
GNRH1	JUN	9606.ENSP0000276414	9606.ENSP00000360266	0	0	0	0	0	0	0	0.421	0.42
GNRH1	HSD3B2	9606.ENSP0000276414	9606.ENSP00000445122	0	0	0	0	0	0	0	0.421	0.42
GNRH1	IGFBP3	9606.ENSP0000276414	9606.ENSP00000370473	0	0	0	0	0	0	0	0.422	0.422
GNRH1	OPRM1	9606.ENSP0000276414	9606.ENSP00000394624	0	0	0	0	0	0	0	0.475	0.475
GNRH1	VEGFA	9606.ENSP0000276414	9606.ENSP00000478570	0	0	0	0	0	0	0	0.515	0.515

GNRH1	NOS3	9606.ENSP00000276414	9606.ENSP00000297494	0	0	0	0	0	0	0	0.558	0.559
GNRH1	PGR	9606.ENSP00000276414	9606.ENSP00000325120	0	0	0	0	0	0	0	0.707	0.707
GNRH1	GRM1	9606.ENSP00000276414	9606.ENSP00000354896	0	0	0	0	0.063	0	0.9	0.231	0.921
GRM1	OPRD1	9606.ENSP00000354896	9606.ENSP00000234961	0	0	0	0	0.063	0.057	0	0.426	0.448
GRM1	OPRM1	9606.ENSP00000354896	9606.ENSP00000394624	0	0	0	0	0.062	0.057	0	0.444	0.465
GRM1	PRKCA	9606.ENSP00000354896	9606.ENSP00000408695	0	0	0	0	0.062	0.413	0	0.218	0.532
GSK3B	MAPK1	9606.ENSP00000324806	9606.ENSP00000215832	0	0	0.265	0.773	0.288	0.446	0	0.543	0.657
GSK3B	NR3C1	9606.ENSP00000324806	9606.ENSP00000231509	0	0	0	0	0	0.085	0.9	0.432	0.943
GSK3B	TP63	9606.ENSP00000324806	9606.ENSP00000264731	0	0	0	0	0	0	0.9	0.101	0.906

GSK3B	SOD1	9606.ENSP0000324806	9606.ENSP0000270142	0	0	0	0	0	0.085	0	0.508	0.53
GSK3B	PPARG	9606.ENSP0000324806	9606.ENSP0000287820	0	0	0	0	0	0.085	0	0.558	0.578
GSK3B	VEGFA	9606.ENSP0000324806	9606.ENSP0000478570	0	0	0	0	0	0	0	0.411	0.411
GSK3B	IGF2	9606.ENSP0000324806	9606.ENSP0000391826	0	0	0	0	0	0.262	0	0.27	0.438
GSK3B	RELA	9606.ENSP0000324806	9606.ENSP0000384273	0	0	0	0	0	0.294	0	0.244	0.443
GSK3B	PRKCA	9606.ENSP0000324806	9606.ENSP0000408695	0	0	0	0.589	0	0.399	0	0.297	0.465
GSK3B	PPP3CA	9606.ENSP0000324806	9606.ENSP0000378323	0	0	0	0	0.081	0.28	0	0.291	0.491
GSK3B	IL6	9606.ENSP0000324806	9606.ENSP0000385675	0	0	0	0	0	0	0	0.573	0.573
GSK3B	NFE2L2	9606.ENSP0000324806	9606.ENSP0000380252	0	0	0	0	0	0.401	0	0.377	0.611

GSK3B	PTGS2	9606.ENSP00000324806	9606.ENSP00000356438	0	0	0	0	0	0.058	0	0.63	0.637
GSK3B	SREBF1	9606.ENSP00000324806	9606.ENSP00000348069	0	0	0	0	0	0.391	0	0.574	0.73
GSK3B	STAT1	9606.ENSP00000324806	9606.ENSP00000354394	0	0	0	0	0.062	0.124	0.9	0.209	0.926
GSK3B	HSF1	9606.ENSP00000324806	9606.ENSP00000431512	0	0	0	0	0.061	0.098	0.9	0.469	0.949
GSK3B	JUN	9606.ENSP00000324806	9606.ENSP00000360266	0	0	0	0	0	0.541	0.9	0.574	0.978
GSK3B	MYC	9606.ENSP00000324806	9606.ENSP00000479618	0	0	0	0	0.063	0.391	0.9	0.732	0.982
GSTM1	HMOX1	9606.ENSP00000311469	9606.ENSP00000216117	0	0	0	0	0	0	0	0.554	0.554
GSTM1	PON1	9606.ENSP00000311469	9606.ENSP00000222381	0	0	0	0	0.061	0	0	0.479	0.489
GSTM1	MPO	9606.ENSP00000311469	9606.ENSP00000225275	0	0	0	0	0	0	0	0.479	0.479

GSTM1	OPRD1	9606.ENSP00000311469	9606.ENSP00000234961	0	0	0	0	0	0.083	0	0.514	0.535
GSTM1	SOD1	9606.ENSP00000311469	9606.ENSP00000270142	0	0	0	0	0.064	0	0	0.415	0.429
GSTM1	SPP1	9606.ENSP00000311469	9606.ENSP00000378517	0	0	0	0	0	0	0	0.402	0.402
GSTM1	NFE2L2	9606.ENSP00000311469	9606.ENSP00000380252	0	0	0	0	0	0	0	0.509	0.509
GSTM1	OPRM1	9606.ENSP00000311469	9606.ENSP00000394624	0	0	0	0	0	0.083	0	0.63	0.646
GSTM1	GSTP1	9606.ENSP00000311469	9606.ENSP00000381607	0	0	0	0.782	0	0	0.65	0.927	0.72
GSTP1	MAPK1	9606.ENSP00000381607	9606.ENSP00000215832	0	0	0	0	0	0.336	0	0.257	0.485
GSTP1	HMOX1	9606.ENSP00000381607	9606.ENSP00000216117	0	0	0	0	0	0	0	0.53	0.53
GSTP1	PON1	9606.ENSP00000381607	9606.ENSP00000222381	0	0	0	0	0.061	0	0	0.389	0.401

GSTP1	MPO	9606.ENSP000003816 07	9606.ENSP000002252 75	0	0	0	0	0	0	0	0.441	0.441
GSTP1	SOD1	9606.ENSP000003816 07	9606.ENSP000002701 42	0	0	0	0	0.063	0.043	0	0.486	0.499
GSTP1	PTGS2	9606.ENSP000003816 07	9606.ENSP000003564 38	0	0	0	0	0	0	0	0.491	0.491
GSTP1	JUN	9606.ENSP000003816 07	9606.ENSP000003602 66	0	0	0	0	0	0.098	0	0.709	0.726
GSTP1	MAPK8	9606.ENSP000003816 07	9606.ENSP000003789 74	0	0	0	0	0	0.566	0	0.686	0.858
GSTP1	NFE2L2	9606.ENSP000003816 07	9606.ENSP000003802 52	0	0	0	0	0	0	0	0.539	0.539
GSTP1	MYC	9606.ENSP000003816 07	9606.ENSP000004796 18	0	0	0	0	0	0	0	0.4	0.4
HAS2	MMP2	9606.ENSP000003069 91	9606.ENSP000002190 70	0	0	0	0	0.152	0	0	0.36	0.434
HAS2	MMP3	9606.ENSP000003069 91	9606.ENSP000002998 55	0	0	0	0	0.098	0	0	0.366	0.404

HAS2	RUNX2	9606.ENSP00000306991	9606.ENSP00000360493	0	0	0	0	0	0	0	0.469	0.469
HAS2	PLAU	9606.ENSP00000306991	9606.ENSP00000361850	0	0	0	0	0.091	0	0	0.528	0.553
HAS2	PTGS2	9606.ENSP00000306991	9606.ENSP00000356438	0	0	0	0	0.062	0	0	0.591	0.6
HIF1A	MAPK1	9606.ENSP00000437955	9606.ENSP00000215832	0	0	0	0	0	0.294	0.8	0.455	0.916
HIF1A	HMOX1	9606.ENSP00000437955	9606.ENSP00000216117	0	0	0	0	0	0	0	0.729	0.73
HIF1A	MMP2	9606.ENSP00000437955	9606.ENSP00000219070	0	0	0	0	0	0	0	0.72	0.721
HIF1A	SERPINE1	9606.ENSP00000437955	9606.ENSP00000223095	0	0	0	0	0.076	0	0	0.672	0.684
HIF1A	NR3C1	9606.ENSP00000437955	9606.ENSP00000231509	0	0	0	0	0	0.095	0.9	0.326	0.933
HIF1A	IL1B	9606.ENSP00000437955	9606.ENSP00000263341	0	0	0	0	0	0	0	0.447	0.447

HIF1A	KDR	9606.ENSP0000437955	9606.ENSP000263923	0	0	0	0	0.061	0	0	0.682	0.688
HIF1A	TP63	9606.ENSP0000437955	9606.ENSP000264731	0	0	0	0	0	0.379	0	0.145	0.446
HIF1A	SOD1	9606.ENSP0000437955	9606.ENSP000270142	0	0	0	0	0	0	0	0.427	0.427
HIF1A	PPARG	9606.ENSP0000437955	9606.ENSP000287820	0	0	0	0	0.062	0.095	0	0.473	0.513
HIF1A	NOS3	9606.ENSP0000437955	9606.ENSP000297494	0	0	0	0	0	0	0.9	0.706	0.969
HIF1A	MMP3	9606.ENSP0000437955	9606.ENSP000299855	0	0	0	0	0	0	0	0.488	0.488
HIF1A	MET	9606.ENSP0000437955	9606.ENSP000317272	0	0	0	0	0	0.086	0	0.394	0.422
HIF1A	MMP1	9606.ENSP0000437955	9606.ENSP000322788	0	0	0	0	0.065	0	0	0.463	0.477
HIF1A	NOS2	9606.ENSP0000437955	9606.ENSP000327251	0	0	0	0	0	0	0	0.404	0.404

HIF1A	SREBF1	9606.ENSP00000437955	9606.ENSP00000348069	0	0	0	0	0.061	0	0	0.5	0.51
HIF1A	VCP	9606.ENSP00000437955	9606.ENSP00000351777	0	0	0	0	0	0.515	0	0.216	0.603
HIF1A	STAT1	9606.ENSP00000437955	9606.ENSP00000354394	0	0	0	0	0.061	0.057	0	0.608	0.623
HIF1A	PARP1	9606.ENSP00000437955	9606.ENSP00000355759	0	0	0	0	0	0.379	0	0.519	0.689
HIF1A	PTGS2	9606.ENSP00000437955	9606.ENSP00000356438	0	0	0	0	0.077	0	0	0.533	0.55
HIF1A	JUN	9606.ENSP00000437955	9606.ENSP00000360266	0	0	0	0	0	0.379	0.9	0.561	0.97
HIF1A	RUNX2	9606.ENSP00000437955	9606.ENSP00000360493	0	0	0	0	0	0.379	0	0.322	0.56
HIF1A	MMP9	9606.ENSP00000437955	9606.ENSP00000361405	0	0	0	0	0	0	0	0.55	0.55
HIF1A	MAPK8	9606.ENSP00000437955	9606.ENSP00000378974	0	0	0	0	0	0	0	0.491	0.491

HIF1A	NFE2L2	9606.ENSP0000437955	9606.ENSP000380252	0	0	0	0	0.152	0	0	0.401	0.47
HIF1A	RELA	9606.ENSP0000437955	9606.ENSP000384273	0	0	0	0	0	0.379	0	0.378	0.597
HIF1A	PPARA	9606.ENSP0000437955	9606.ENSP000385523	0	0	0	0	0	0.095	0	0.651	0.671
HIF1A	IL6	9606.ENSP0000437955	9606.ENSP000385675	0	0	0	0	0.065	0	0	0.539	0.55
HIF1A	IGF2	9606.ENSP0000437955	9606.ENSP000391826	0	0	0	0	0	0	0	0.648	0.648
HIF1A	IL10	9606.ENSP0000437955	9606.ENSP000412237	0	0	0	0	0	0	0	0.624	0.624
HIF1A	IKBKB	9606.ENSP0000437955	9606.ENSP000430684	0	0	0	0	0.062	0.345	0	0.231	0.486
HIF1A	HSF1	9606.ENSP0000437955	9606.ENSP000431512	0	0	0	0	0	0.063	0	0.439	0.452
HIF1A	MYC	9606.ENSP0000437955	9606.ENSP000479618	0	0	0	0	0	0.433	0	0.783	0.872

HIF1A	VEGFA	9606.ENSP00000437955	9606.ENSP00000478570	0	0	0	0	0.062	0.348	0.9	0.947	0.996
HMOX1	MAPK1	9606.ENSP00000216117	9606.ENSP00000215832	0	0	0	0	0	0	0	0.778	0.779
HMOX1	IL2	9606.ENSP00000216117	9606.ENSP00000226730	0	0	0	0	0	0	0	0.421	0.42
HMOX1	MYC	9606.ENSP00000216117	9606.ENSP00000479618	0	0	0	0	0	0	0	0.421	0.42
HMOX1	STAT1	9606.ENSP00000216117	9606.ENSP00000354394	0	0	0	0	0	0	0	0.425	0.424
HMOX1	MMP2	9606.ENSP00000216117	9606.ENSP00000219070	0	0	0	0	0	0	0	0.449	0.449
HMOX1	SERPINE1	9606.ENSP00000216117	9606.ENSP00000223095	0	0	0	0	0.085	0	0	0.473	0.497
HMOX1	HSPB1	9606.ENSP00000216117	9606.ENSP00000248553	0	0	0	0	0.062	0	0	0.498	0.509
HMOX1	SELE	9606.ENSP00000216117	9606.ENSP00000331736	0	0	0	0	0	0	0	0.511	0.511

HMOX1	NOX1	9606.ENSP00000216117	9606.ENSP00000362057	0	0	0	0	0	0	0	0.57	0.57
HMOX1	MMP1	9606.ENSP00000216117	9606.ENSP00000322788	0	0	0	0	0.068	0	0	0.583	0.595
HMOX1	VCAM1	9606.ENSP00000216117	9606.ENSP00000294728	0	0	0	0	0.061	0	0	0.589	0.598
HMOX1	SPP1	9606.ENSP00000216117	9606.ENSP00000378517	0	0	0	0	0.061	0	0	0.6	0.608
HMOX1	ICAM1	9606.ENSP00000216117	9606.ENSP00000264832	0	0	0	0	0.077	0	0	0.64	0.653
HMOX1	IFNG	9606.ENSP00000216117	9606.ENSP00000229135	0	0	0	0	0	0	0	0.67	0.67
HMOX1	MPO	9606.ENSP00000216117	9606.ENSP00000225275	0	0	0	0	0	0	0	0.683	0.683
HMOX1	SOD1	9606.ENSP00000216117	9606.ENSP00000270142	0	0	0	0	0	0	0	0.728	0.728
HMOX1	PPARG	9606.ENSP00000216117	9606.ENSP00000287820	0	0	0	0	0	0	0	0.735	0.735

HMOX1	MMP9	9606.ENSP0000216117	9606.ENSP00000361405	0	0	0	0	0.088	0	0	0.745	0.759
HMOX1	MAPK8	9606.ENSP0000216117	9606.ENSP00000378974	0	0	0	0	0	0	0	0.8	0.8
HMOX1	IL1B	9606.ENSP0000216117	9606.ENSP00000263341	0	0	0	0	0.07	0	0	0.814	0.819
HMOX1	NOS2	9606.ENSP0000216117	9606.ENSP00000327251	0	0	0	0	0	0.185	0	0.789	0.821
HMOX1	PTGS2	9606.ENSP0000216117	9606.ENSP00000356438	0	0	0	0	0.076	0	0	0.827	0.834
HMOX1	IL6	9606.ENSP0000216117	9606.ENSP00000385675	0	0	0	0	0.073	0	0	0.841	0.847
HMOX1	POR	9606.ENSP0000216117	9606.ENSP00000419970	0	0	0	0	0.071	0.472	0	0.726	0.854
HMOX1	NOS3	9606.ENSP0000216117	9606.ENSP00000297494	0	0	0	0	0	0.185	0	0.829	0.855
HMOX1	VEGFA	9606.ENSP0000216117	9606.ENSP00000478570	0	0	0	0	0	0	0	0.867	0.867

HMOX1	IL10	9606.ENSP0000216117	9606.ENSP000412237	0	0	0	0	0	0	0	0.867	0.868
HMOX1	IL4	9606.ENSP0000216117	9606.ENSP000231449	0	0	0	0	0	0	0.9	0.475	0.945
HMOX1	MAPK14	9606.ENSP0000216117	9606.ENSP000229795	0	0	0	0	0	0	0.9	0.735	0.972
HMOX1	JUN	9606.ENSP0000216117	9606.ENSP000360266	0	0	0	0	0	0	0.9	0.784	0.977
HMOX1	NFE2L2	9606.ENSP0000216117	9606.ENSP000380252	0	0	0	0	0	0	0.9	0.968	0.996
HPSE	LYZ	9606.ENSP0000384262	9606.ENSP000261267	0	0	0	0	0.061	0	0.9	0.121	0.91
HPSE	SLPI	9606.ENSP0000384262	9606.ENSP000342082	0	0	0	0	0.061	0	0.9	0	0.902
HPSE	VEGFA	9606.ENSP0000384262	9606.ENSP000478570	0	0	0	0	0	0	0	0.599	0.599
HSD3B2	POR	9606.ENSP0000445122	9606.ENSP000419970	0	0	0	0	0.063	0	0	0.555	0.565

HSF1	MAPK1	9606.ENSP000004 31512	9606.ENSP0000021 5832	0	0	0	0	0.064	0.465	0.9	0.32	0.961
HSF1	MAPK14	9606.ENSP000004 31512	9606.ENSP0000022 9795	0	0	0	0	0.063	0.174	0	0.383	0.48
HSF1	HSPB1	9606.ENSP000004 31512	9606.ENSP0000024 8553	0	0	0	0	0.09	0.294	0	0.755	0.828
HSF1	SOD1	9606.ENSP000004 31512	9606.ENSP0000027 0142	0	0	0	0	0	0	0	0.497	0.497
HSF1	VCP	9606.ENSP000004 31512	9606.ENSP0000035 1777	0	0	0	0	0	0.05	0.9	0.163	0.913
HSF1	STAT1	9606.ENSP000004 31512	9606.ENSP0000035 4394	0	0	0	0	0.063	0.379	0	0.103	0.432
HSF1	PARP1	9606.ENSP000004 31512	9606.ENSP0000035 5759	0	0	0	0	0	0	0	0.579	0.579
HSF1	MAPK8	9606.ENSP000004 31512	9606.ENSP0000037 8974	0	0	0	0	0	0.379	0	0.626	0.758
HSF1	IL6	9606.ENSP000004 31512	9606.ENSP0000038 5675	0	0	0	0	0	0	0	0.508	0.508

HSPB1	MAPK1	9606.ENSP00000248553	9606.ENSP00000215832	0	0	0	0	0	0.185	0	0.788	0.82
HSPB1	NFKBIA	9606.ENSP00000248553	9606.ENSP00000216797	0	0	0	0	0	0.379	0	0.266	0.524
HSPB1	MMP2	9606.ENSP00000248553	9606.ENSP00000219070	0	0	0	0	0.086	0	0	0.615	0.633
HSPB1	IFNG	9606.ENSP00000248553	9606.ENSP00000229135	0	0	0	0	0	0	0	0.524	0.524
HSPB1	MAPK14	9606.ENSP00000248553	9606.ENSP00000229795	0	0	0	0	0	0.432	0.9	0.846	0.99
HSPB1	NOS3	9606.ENSP00000248553	9606.ENSP00000297494	0	0	0	0	0.061	0.112	0	0.348	0.408
HSPB1	MYC	9606.ENSP00000248553	9606.ENSP00000479618	0	0	0	0	0	0.099	0	0.445	0.478
HSPB1	SOD1	9606.ENSP00000248553	9606.ENSP00000270142	0	0	0	0	0.076	0	0	0.476	0.495
HSPB1	IL6	9606.ENSP00000248553	9606.ENSP00000385675	0	0	0	0	0	0	0	0.511	0.511

HSPB1	VEGFA	9606.ENSP0000248553	9606.ENSP0000478570	0	0	0	0	0.049	0.132	0	0.476	0.529
HSPB1	IKBKB	9606.ENSP0000248553	9606.ENSP0000430684	0	0	0	0	0	0.402	0	0.288	0.556
HSPB1	JUN	9606.ENSP0000248553	9606.ENSP0000360266	0	0	0	0	0.062	0	0	0.571	0.58
HSPB1	MMP9	9606.ENSP0000248553	9606.ENSP0000361405	0	0	0	0	0	0	0	0.6	0.6
HSPB1	MAPK8	9606.ENSP0000248553	9606.ENSP0000378974	0	0	0	0	0	0.159	0	0.648	0.691
HTR3A	OPRD1	9606.ENSP0000347754	9606.ENSP0000234961	0	0	0	0	0.062	0.047	0	0.669	0.678
HTR3A	SLC6A4	9606.ENSP0000347754	9606.ENSP0000261707	0	0	0	0	0	0	0	0.816	0.816
HTR3A	OPRM1	9606.ENSP0000347754	9606.ENSP0000394624	0	0	0	0	0	0.047	0	0.672	0.674
ICAM1	MAPK1	9606.ENSP0000264832	9606.ENSP0000215832	0	0	0	0	0	0	0	0.723	0.723

ICAM1	NFKBIA	9606.ENSP0000264832	9606.ENSP00000216797	0	0	0	0	0.126	0	0	0.769	0.789
ICAM1	MMP2	9606.ENSP0000264832	9606.ENSP00000219070	0	0	0	0	0	0	0	0.743	0.743
ICAM1	PLAT	9606.ENSP0000264832	9606.ENSP00000220809	0	0	0	0	0	0	0	0.481	0.481
ICAM1	SERPINE1	9606.ENSP0000264832	9606.ENSP00000223095	0	0	0	0	0.088	0	0	0.683	0.698
ICAM1	MPO	9606.ENSP0000264832	9606.ENSP00000225275	0	0	0	0	0	0	0	0.797	0.797
ICAM1	IL2	9606.ENSP0000264832	9606.ENSP00000226730	0	0	0	0	0	0	0	0.834	0.834
ICAM1	IFNG	9606.ENSP0000264832	9606.ENSP00000229135	0	0	0	0	0	0	0	0.87	0.87
ICAM1	MAPK14	9606.ENSP0000264832	9606.ENSP00000229795	0	0	0	0	0	0	0	0.655	0.655
ICAM1	IL4	9606.ENSP0000264832	9606.ENSP00000231449	0	0	0	0	0	0	0	0.862	0.862

ICAM1	IRF1	9606.ENSP00000264832	9606.ENSP00000245414	0	0	0	0	0.156	0	0.9	0.477	0.952
ICAM1	IL1A	9606.ENSP00000264832	9606.ENSP00000263339	0	0	0	0	0.113	0	0	0.801	0.816
ICAM1	IL1B	9606.ENSP00000264832	9606.ENSP00000263341	0	0	0	0	0.154	0	0	0.916	0.926
ICAM1	KDR	9606.ENSP00000264832	9606.ENSP00000263923	0	0	0	0	0.055	0	0	0.591	0.597
ICAM1	PLAU	9606.ENSP00000264832	9606.ENSP00000361850	0	0	0	0	0.098	0	0	0.398	0.433
ICAM1	IKBKB	9606.ENSP00000264832	9606.ENSP00000430684	0	0	0	0	0.061	0	0	0.45	0.461
ICAM1	MYC	9606.ENSP00000264832	9606.ENSP00000479618	0	0	0	0	0.052	0	0	0.457	0.463
ICAM1	NOS2	9606.ENSP00000264832	9606.ENSP00000327251	0	0	0	0	0	0.061	0	0.544	0.554
ICAM1	STAT1	9606.ENSP00000264832	9606.ENSP00000354394	0	0	0	0	0.061	0	0	0.575	0.584

ICAM1	OLR1	9606.ENSP0000264832	9606.ENSP000309124	0	0	0	0	0.065	0	0	0.577	0.588
ICAM1	MMP1	9606.ENSP0000264832	9606.ENSP000322788	0	0	0	0	0.088	0	0	0.575	0.596
ICAM1	THBD	9606.ENSP0000264832	9606.ENSP000366307	0	0	0	0	0.106	0	0	0.658	0.681
ICAM1	NOS3	9606.ENSP0000264832	9606.ENSP000297494	0	0	0	0	0	0	0	0.705	0.705
ICAM1	PTGS2	9606.ENSP0000264832	9606.ENSP000356438	0	0	0	0	0.123	0	0	0.677	0.705
ICAM1	PPARG	9606.ENSP0000264832	9606.ENSP000287820	0	0	0	0	0.061	0	0	0.698	0.705
ICAM1	MMP3	9606.ENSP0000264832	9606.ENSP000299855	0	0	0	0	0.063	0	0	0.709	0.716
ICAM1	SPP1	9606.ENSP0000264832	9606.ENSP000378517	0	0	0	0	0	0	0	0.719	0.719
ICAM1	RELA	9606.ENSP0000264832	9606.ENSP000384273	0	0	0	0	0.065	0.417	0	0.554	0.735

ICAM1	MMP9	9606.ENSP0000264832	9606.ENSP000003614O5	0	0	0	0	0.093	0	0	0.722	0.737
ICAM1	JUN	9606.ENSP0000264832	9606.ENSP00000360266	0	0	0	0	0	0	0	0.755	0.755
ICAM1	MAPK8	9606.ENSP0000264832	9606.ENSP00000378974	0	0	0	0	0	0	0	0.777	0.777
ICAM1	VEGFA	9606.ENSP0000264832	9606.ENSP00000478570	0	0	0	0	0	0	0	0.89	0.89
ICAM1	VCAM1	9606.ENSP0000264832	9606.ENSP00000294728	0	0	0	0	0.069	0	0	0.938	0.94
ICAM1	IL6	9606.ENSP0000264832	9606.ENSP00000385675	0	0	0	0	0.152	0	0	0.947	0.953
ICAM1	SELE	9606.ENSP0000264832	9606.ENSP00000331736	0	0	0	0	0.058	0	0	0.953	0.954
ICAM1	IL10	9606.ENSP0000264832	9606.ENSP00000412237	0	0	0	0	0	0	0.9	0.858	0.985
IFNG	MAPK1	9606.ENSP0000229135	9606.ENSP00000215832	0	0	0	0	0	0	0	0.462	0.462

IFNG	NFKBIA	9606.ENSP0000229135	9606.ENSP00000216797	0	0	0	0	0	0	0	0.434	0.434
IFNG	MMP2	9606.ENSP0000229135	9606.ENSP00000219070	0	0	0	0	0	0	0	0.638	0.638
IFNG	SERPINE1	9606.ENSP0000229135	9606.ENSP00000223095	0	0	0	0	0	0	0	0.471	0.471
IFNG	MPO	9606.ENSP0000229135	9606.ENSP00000225275	0	0	0	0	0	0	0	0.581	0.581
IFNG	IL2	9606.ENSP0000229135	9606.ENSP00000226730	0	0	0	0	0.087	0	0	0.935	0.938
IFNG	MMP1	9606.ENSP0000229135	9606.ENSP00000322788	0	0	0	0	0.054	0	0	0.4	0.408
IFNG	MMP3	9606.ENSP0000229135	9606.ENSP00000299855	0	0	0	0	0.054	0	0	0.417	0.424
IFNG	MYC	9606.ENSP0000229135	9606.ENSP00000479618	0	0	0	0	0	0	0	0.448	0.448
IFNG	PPARG	9606.ENSP0000229135	9606.ENSP00000287820	0	0	0	0	0	0	0	0.449	0.449

IFNG	NOS3	9606.ENSP0000229135	9606.ENSP00000297494	0	0	0	0	0	0	0	0.451	0.451
IFNG	NOX1	9606.ENSP0000229135	9606.ENSP00000362057	0	0	0	0	0.061	0	0	0.48	0.491
IFNG	SPP1	9606.ENSP0000229135	9606.ENSP00000378517	0	0	0	0	0	0	0	0.51	0.51
IFNG	MAPK8	9606.ENSP0000229135	9606.ENSP00000378974	0	0	0	0	0	0	0	0.517	0.517
IFNG	IGF2	9606.ENSP0000229135	9606.ENSP00000391826	0	0	0	0	0.055	0	0	0.536	0.543
IFNG	PTGS2	9606.ENSP0000229135	9606.ENSP00000356438	0	0	0	0	0	0	0	0.594	0.594
IFNG	MAPK14	9606.ENSP0000229135	9606.ENSP00000229795	0	0	0	0	0	0	0	0.603	0.603
IFNG	VEGFA	9606.ENSP0000229135	9606.ENSP00000478570	0	0	0	0	0	0	0	0.669	0.669
IFNG	VCAM1	9606.ENSP0000229135	9606.ENSP00000294728	0	0	0	0	0	0	0	0.684	0.684

IFNG	SELE	9606.<br>ENSP0<br>00002<br>29135	9606.E<br>NSP00<br>00033<br>1736	0	0	0	0	0	0	0	0.713	0.713
IFNG	IL1A	9606.<br>ENSP0<br>00002<br>29135	9606.E<br>NSP00<br>00026<br>3339	0	0	0	0	0	0	0	0.731	0.731
IFNG	MMP9	9606.<br>ENSP0<br>00002<br>29135	9606.E<br>NSP00<br>00036<br>1405	0	0	0	0	0	0	0	0.735	0.735
IFNG	NOS2	9606.<br>ENSP0<br>00002<br>29135	9606.E<br>NSP00<br>00032<br>7251	0	0	0	0	0	0	0	0.836	0.836
IFNG	IL1B	9606.<br>ENSP0<br>00002<br>29135	9606.E<br>NSP00<br>00026<br>3341	0	0	0	0	0.082	0	0	0.898	0.902
IFNG	IL4	9606.<br>ENSP0<br>00002<br>29135	9606.E<br>NSP00<br>00023<br>1449	0	0	0	0	0	0	0	0.921	0.921
IFNG	IL6	9606.<br>ENSP0<br>00002<br>29135	9606.E<br>NSP00<br>00038<br>5675	0	0	0	0	0.064	0	0	0.937	0.939
IFNG	IL10	9606.<br>ENSP0<br>00002<br>29135	9606.E<br>NSP00<br>00041<br>2237	0	0	0	0	0.064	0	0	0.944	0.946
IFNG	JUN	9606.<br>ENSP0<br>00002<br>29135	9606.E<br>NSP00<br>00036<br>0266	0	0	0	0	0	0	0.9	0.518	0.949

IFNG	RELA	9606.ENSP000002 29135	9606.ENSP0000038 4273	0	0	0	0	0	0	0.9	0.614	0.959
IFNG	IRF1	9606.ENSP000002 29135	9606.ENSP0000024 5414	0	0	0	0	0.089	0	0.9	0.805	0.98
IFNG	STAT1	9606.ENSP000002 29135	9606.ENSP0000035 4394	0	0	0	0	0.063	0	0.9	0.898	0.989
IGF2	MMP2	9606.ENSP000003 91826	9606.ENSP0000021 9070	0	0	0	0	0.165	0	0.9	0.399	0.945
IGF2	SERPINE1	9606.ENSP000003 91826	9606.ENSP0000022 3095	0	0	0	0	0.088	0	0.9	0.311	0.931
IGF2	MAPK14	9606.ENSP000003 91826	9606.ENSP0000022 9795	0	0	0	0	0	0	0	0.411	0.411
IGF2	NR3C1	9606.ENSP000003 91826	9606.ENSP0000023 1509	0	0	0	0	0	0	0	0.426	0.426
IGF2	KDR	9606.ENSP000003 91826	9606.ENSP0000026 3923	0	0	0	0	0.075	0	0.6	0.44	0.774
IGF2	MET	9606.ENSP000003 91826	9606.ENSP0000031 7272	0	0	0	0	0.061	0.262	0.6	0.419	0.817

IGF2	MMP1	9606.ENSP0000391826	9606.ENSP00000322788	0	0	0	0	0.076	0	0.9	0.257	0.925
IGF2	PGR	9606.ENSP0000391826	9606.ENSP00000325120	0	0	0	0	0.066	0	0	0.399	0.414
IGF2	JUN	9606.ENSP0000391826	9606.ENSP00000360266	0	0	0	0	0.044	0	0	0.405	0.407
IGF2	MMP9	9606.ENSP0000391826	9606.ENSP00000361405	0	0	0	0	0	0	0	0.413	0.413
IGF2	IGFBP3	9606.ENSP0000391826	9606.ENSP00000370473	0	0	0	0	0.155	0.379	0.9	0.938	0.996
IGF2	SPP1	9606.ENSP0000391826	9606.ENSP00000378517	0	0	0	0	0.076	0	0	0.448	0.468
IGF2	IL6	9606.ENSP0000391826	9606.ENSP00000385675	0	0	0	0	0	0	0	0.697	0.698
IGF2	MYC	9606.ENSP0000391826	9606.ENSP00000479618	0	0	0	0	0	0	0	0.578	0.578
IGF2	VEGFA	9606.ENSP0000391826	9606.ENSP00000478570	0	0	0	0	0.069	0	0.9	0.809	0.98

IGFBP3	MMP2	9606.ENSP0000370473	9606.ENSP00000219070	0	0	0	0	0.196	0.379	0.9	0.413	0.966
IGFBP3	SERPINE1	9606.ENSP0000370473	9606.ENSP00000223095	0	0	0	0	0.192	0	0	0.484	0.565
IGFBP3	PPARG	9606.ENSP0000370473	9606.ENSP00000287820	0	0	0	0	0	0.379	0	0.588	0.733
IGFBP3	NOS3	9606.ENSP0000370473	9606.ENSP00000297494	0	0	0	0	0.061	0	0	0.527	0.537
IGFBP3	MMP3	9606.ENSP0000370473	9606.ENSP00000299855	0	0	0	0	0.125	0.379	0	0.354	0.618
IGFBP3	MMP1	9606.ENSP0000370473	9606.ENSP00000322788	0	0	0	0	0.142	0.379	0.9	0.327	0.959
IGFBP3	STAT1	9606.ENSP0000370473	9606.ENSP00000354394	0	0	0	0	0	0	0	0.54	0.54
IGFBP3	PTGS2	9606.ENSP0000370473	9606.ENSP00000356438	0	0	0	0	0.086	0	0	0.382	0.411
IGFBP3	MMP9	9606.ENSP0000370473	9606.ENSP00000361405	0	0	0	0	0	0	0	0.655	0.656

IGFBP3	PLAU	9606.ENSP00000370473	9606.ENSP00000361850	0	0	0	0	0.098	0	0	0.378	0.415
IGFBP3	VEGFA	9606.ENSP00000370473	9606.ENSP00000478570	0	0	0	0	0.091	0	0	0.754	0.767
IGFBP3	MAPK8	9606.ENSP00000370473	9606.ENSP00000378974	0	0	0	0	0	0	0.9	0.292	0.926
IGFBP3	SPP1	9606.ENSP00000370473	9606.ENSP00000378517	0	0	0	0	0.07	0	0.9	0.359	0.935
IGFBP3	IL6	9606.ENSP00000370473	9606.ENSP00000385675	0	0	0	0	0.076	0	0.9	0.536	0.953
IKBKB	NFKBIA	9606.ENSP00000430684	9606.ENSP00000216797	0	0	0	0	0.061	0.919	0.9	0.906	0.999
IKBKB	MAPK14	9606.ENSP00000430684	9606.ENSP00000229795	0	0	0	0.648	0	0.574	0	0.447	0.637
IKBKB	IL1B	9606.ENSP00000430684	9606.ENSP00000263341	0	0	0	0	0	0.059	0	0.628	0.634
IKBKB	TP63	9606.ENSP00000430684	9606.ENSP00000264731	0	0	0	0	0	0.379	0.9	0.139	0.941

IKBKB	VCAM1	9606.ENSP00000430684	9606.ENSP00000294728	0	0	0	0	0.062	0	0	0.4	0.413
IKBKB	STAT1	9606.ENSP00000430684	9606.ENSP00000354394	0	0	0	0	0.061	0.124	0	0.361	0.429
IKBKB	PTGS2	9606.ENSP00000430684	9606.ENSP00000356438	0	0	0	0	0.064	0.058	0	0.477	0.498
IKBKB	JUN	9606.ENSP00000430684	9606.ENSP00000360266	0	0	0	0	0	0.374	0	0.578	0.725
IKBKB	RELA	9606.ENSP00000430684	9606.ENSP00000384273	0	0	0	0	0.063	0.871	0.9	0.831	0.997
IKBKB	IL6	9606.ENSP00000430684	9606.ENSP00000385675	0	0	0	0	0.048	0	0	0.794	0.796
IKBKB	PRKCA	9606.ENSP00000430684	9606.ENSP00000408695	0	0	0	0.567	0	0.358	0.9	0.268	0.939
IKBKB	IL10	9606.ENSP00000430684	9606.ENSP00000412237	0	0	0	0	0	0.079	0	0.441	0.463
IKBKB	VEGFA	9606.ENSP00000430684	9606.ENSP00000478570	0	0	0	0	0.064	0	0	0.593	0.603

IL10	MAPK1	9606.ENSP000004 12237	9606.ENSP00000021 5832	0	0	0	0	0	0	0	0.777	0.777
IL10	NFKBIA	9606.ENSP000004 12237	9606.ENSP00000021 6797	0	0	0	0	0.061	0	0	0.511	0.521
IL10	MMP2	9606.ENSP000004 12237	9606.ENSP00000021 9070	0	0	0	0	0	0	0	0.584	0.584
IL10	SERPINE1	9606.ENSP000004 12237	9606.ENSP00000022 3095	0	0	0	0	0	0	0	0.578	0.578
IL10	MPO	9606.ENSP000004 12237	9606.ENSP00000022 5275	0	0	0	0	0	0	0	0.772	0.772
IL10	IL2	9606.ENSP000004 12237	9606.ENSP00000022 6730	0	0	0	0	0	0	0	0.956	0.957
IL10	MAPK14	9606.ENSP000004 12237	9606.ENSP00000022 9795	0	0	0	0	0	0	0	0.754	0.754
IL10	IL4	9606.ENSP000004 12237	9606.ENSP00000023 1449	0	0	0	0	0	0	0	0.972	0.972
IL10	NR3C1	9606.ENSP000004 12237	9606.ENSP00000023 1509	0	0	0	0	0	0.328	0	0.671	0.77

IL10	IRF1	9606.ENSP00000412237	9606.ENSP00000245414	0	0	0	0	0.07	0	0	0.58	0.593
IL10	IL1A	9606.ENSP00000412237	9606.ENSP00000263339	0	0	0	0	0.063	0	0.9	0.738	0.973
IL10	IL1B	9606.ENSP00000412237	9606.ENSP00000263341	0	0	0	0	0.085	0	0.9	0.96	0.996
IL10	KDR	9606.ENSP00000412237	9606.ENSP00000263923	0	0	0	0	0	0	0	0.454	0.454
IL10	SOD1	9606.ENSP00000412237	9606.ENSP00000270142	0	0	0	0	0	0	0	0.433	0.433
IL10	MMP10	9606.ENSP00000412237	9606.ENSP00000279441	0	0	0	0	0.052	0	0	0.442	0.448
IL10	PPARG	9606.ENSP00000412237	9606.ENSP00000287820	0	0	0	0	0	0	0	0.556	0.556
IL10	VCAM1	9606.ENSP00000412237	9606.ENSP00000294728	0	0	0	0	0.055	0	0	0.696	0.7
IL10	NOS3	9606.ENSP00000412237	9606.ENSP00000297494	0	0	0	0	0	0	0	0.708	0.709

IL10	MMP3	9606.ENSP0000412237	9606.ENSP00000299855	0	0	0	0	0.052	0	0	0.598	0.603
IL10	MMP1	9606.ENSP0000412237	9606.ENSP00000322788	0	0	0	0	0.052	0	0	0.55	0.555
IL10	NOS2	9606.ENSP0000412237	9606.ENSP00000327251	0	0	0	0	0	0	0	0.696	0.696
IL10	SELE	9606.ENSP0000412237	9606.ENSP00000331736	0	0	0	0	0.049	0	0	0.802	0.803
IL10	SLPI	9606.ENSP0000412237	9606.ENSP00000342082	0	0	0	0	0	0	0	0.4	0.4
IL10	STAT1	9606.ENSP0000412237	9606.ENSP00000354394	0	0	0	0	0	0.096	0	0.858	0.866
IL10	PTGS1	9606.ENSP0000412237	9606.ENSP00000354612	0	0	0	0	0.05	0	0	0.419	0.425
IL10	PARP1	9606.ENSP0000412237	9606.ENSP00000355759	0	0	0	0	0	0.379	0	0.249	0.513
IL10	PTGS2	9606.ENSP0000412237	9606.ENSP00000356438	0	0	0	0	0.061	0	0	0.831	0.836

IL10	JUN	9606.ENSP000004 12237	9606.ENSP0000036 0266	0	0	0	0	0	0	0	0.759	0.76
IL10	MMP9	9606.ENSP000004 12237	9606.ENSP0000036 1405	0	0	0	0	0	0	0	0.831	0.831
IL10	TNFSF15	9606.ENSP000004 12237	9606.ENSP0000036 3157	0	0	0	0	0.077	0	0	0.438	0.459
IL10	THBD	9606.ENSP000004 12237	9606.ENSP0000036 6307	0	0	0	0	0	0	0	0.526	0.526
IL10	SPP1	9606.ENSP000004 12237	9606.ENSP0000037 8517	0	0	0	0	0	0	0	0.556	0.556
IL10	MAPK8	9606.ENSP000004 12237	9606.ENSP0000037 8974	0	0	0	0	0	0	0	0.674	0.674
IL10	NFE2L2	9606.ENSP000004 12237	9606.ENSP0000038 0252	0	0	0	0	0	0	0	0.429	0.429
IL10	RELA	9606.ENSP000004 12237	9606.ENSP0000038 4273	0	0	0	0	0	0	0	0.547	0.547
IL10	IL6	9606.ENSP000004 12237	9606.ENSP0000038 5675	0	0	0	0	0.077	0	0.9	0.974	0.997

IL10	MYC	9606.ENSP0000412237	9606.ENSP00000479618	0	0	0	0	0	0	0	0.492	0.491
IL10	VEGFA	9606.ENSP0000412237	9606.ENSP00000478570	0	0	0	0	0	0	0	0.884	0.885
IL1A	NFKBIA	9606.ENSP0000263339	9606.ENSP00000216797	0	0	0	0	0.085	0	0	0.53	0.551
IL1A	MMP2	9606.ENSP0000263339	9606.ENSP00000219070	0	0	0	0	0	0	0	0.44	0.44
IL1A	PLAT	9606.ENSP0000263339	9606.ENSP00000220809	0	0	0	0	0	0	0	0.554	0.554
IL1A	SERPINE1	9606.ENSP0000263339	9606.ENSP00000223095	0	0	0	0	0.091	0	0	0.423	0.453
IL1A	MPO	9606.ENSP0000263339	9606.ENSP00000225275	0	0	0	0	0.063	0	0	0.426	0.439
IL1A	IL2	9606.ENSP0000263339	9606.ENSP00000226730	0	0	0	0	0	0	0	0.763	0.763
IL1A	IL4	9606.ENSP0000263339	9606.ENSP00000231449	0	0	0	0	0	0	0.9	0.732	0.972

IL1A	IRF1	9606.ENSP00000263339	9606.ENSP00000245414	0	0	0	0	0.108	0	0	0.425	0.465
IL1A	NOS2	9606.ENSP00000263339	9606.ENSP00000327251	0	0	0	0	0.063	0	0	0.399	0.412
IL1A	MMP9	9606.ENSP00000263339	9606.ENSP00000361405	0	0	0	0	0.069	0	0	0.53	0.543
IL1A	MMP3	9606.ENSP00000263339	9606.ENSP00000299855	0	0	0	0	0.069	0	0	0.536	0.549
IL1A	VEGFA	9606.ENSP00000263339	9606.ENSP00000478570	0	0	0	0	0	0	0	0.55	0.55
IL1A	PLAU	9606.ENSP00000263339	9606.ENSP00000361850	0	0	0	0	0.098	0	0	0.592	0.616
IL1A	SELE	9606.ENSP00000263339	9606.ENSP00000331736	0	0	0	0	0.062	0	0	0.721	0.727
IL1A	VCAM1	9606.ENSP00000263339	9606.ENSP00000294728	0	0	0	0	0.061	0	0	0.735	0.741
IL1A	MMP1	9606.ENSP00000263339	9606.ENSP00000322788	0	0	0	0	0.111	0	0	0.725	0.745

IL1A	PTGS2	9606.ENSP00000263339	9606.ENSP00000356438	0	0	0	0	0.213	0	0	0.772	0.813
IL1A	RELA	9606.ENSP00000263339	9606.ENSP00000384273	0	0	0	0	0	0	0.9	0.355	0.932
IL1A	JUN	9606.ENSP00000263339	9606.ENSP00000360266	0	0	0	0	0	0	0.9	0.442	0.941
IL1A	IL6	9606.ENSP00000263339	9606.ENSP00000385675	0	0	0	0	0.375	0	0	0.918	0.946
IL1A	IL1B	9606.ENSP00000263339	9606.ENSP00000263341	0	0	0	0	0.753	0	0.9	0.921	0.997
IL1B	MAPK1	9606.ENSP00000263341	9606.ENSP00000215832	0	0	0	0	0	0	0	0.794	0.794
IL1B	NFKBIA	9606.ENSP00000263341	9606.ENSP00000216797	0	0	0	0	0.155	0	0	0.833	0.853
IL1B	MMP2	9606.ENSP00000263341	9606.ENSP00000219070	0	0	0	0	0	0.379	0	0.802	0.872
IL1B	PLAT	9606.ENSP00000263341	9606.ENSP00000220809	0	0	0	0	0	0	0	0.411	0.41

IL1B	SERPINE1	9606.ENSP00000263341	9606.ENSP00000223095	0	0	0	0	0.063	0	0	0.58	0.589
IL1B	MPO	9606.ENSP00000263341	9606.ENSP00000225275	0	0	0	0	0.098	0	0	0.807	0.819
IL1B	IL2	9606.ENSP00000263341	9606.ENSP00000226730	0	0	0	0	0	0	0	0.9	0.9
IL1B	MAPK14	9606.ENSP00000263341	9606.ENSP00000229795	0	0	0	0	0	0	0.9	0.79	0.978
IL1B	IL4	9606.ENSP00000263341	9606.ENSP00000231449	0	0	0	0	0	0	0.9	0.916	0.991
IL1B	NR3C1	9606.ENSP00000263341	9606.ENSP00000231509	0	0	0	0	0	0	0	0.529	0.529
IL1B	IRF1	9606.ENSP00000263341	9606.ENSP00000245414	0	0	0	0	0.205	0	0	0.716	0.765
IL1B	LYZ	9606.ENSP00000263341	9606.ENSP00000261267	0	0	0	0	0.13	0	0	0.354	0.414
IL1B	SLC6A4	9606.ENSP00000263341	9606.ENSP00000261707	0	0	0	0	0	0	0	0.61	0.61

IL1B	KDR	9606.<br>ENSP0<br>00002<br>63341	9606.E<br>NSP00<br>00026<br>3923	0	0	0	0	0.061	0	0	0.39	0.402
IL1B	MYC	9606.<br>ENSP0<br>00002<br>63341	9606.E<br>NSP00<br>00047<br>9618	0	0	0	0	0.044	0.099	0	0.399	0.437
IL1B	SLPI	9606.<br>ENSP0<br>00002<br>63341	9606.E<br>NSP00<br>00034<br>2082	0	0	0	0	0.085	0	0	0.413	0.44
IL1B	THBD	9606.<br>ENSP0<br>00002<br>63341	9606.E<br>NSP00<br>00036<br>6307	0	0	0	0	0.153	0	0	0.413	0.481
IL1B	SOD1	9606.<br>ENSP0<br>00002<br>63341	9606.E<br>NSP00<br>00027<br>0142	0	0	0	0	0	0	0	0.518	0.518
IL1B	MMP10	9606.<br>ENSP0<br>00002<br>63341	9606.E<br>NSP00<br>00027<br>9441	0	0	0	0	0.128	0	0	0.48	0.528
IL1B	PGR	9606.<br>ENSP0<br>00002<br>63341	9606.E<br>NSP00<br>00032<br>5120	0	0	0	0	0	0	0	0.543	0.543
IL1B	NFE2L2	9606.<br>ENSP0<br>00002<br>63341	9606.E<br>NSP00<br>00038<br>0252	0	0	0	0	0	0	0	0.547	0.547
IL1B	RUNX2	9606.<br>ENSP0<br>00002<br>63341	9606.E<br>NSP00<br>00036<br>0493	0	0	0	0	0.076	0	0	0.597	0.612

IL1B	NOX1	9606.<br>ENSP0<br>00002<br>63341	9606.E<br>NSP00<br>00036<br>2057	0	0	0	0	0.064	0	0	0.61	0.62
IL1B	OPRM1	9606.<br>ENSP0<br>00002<br>63341	9606.E<br>NSP00<br>00039<br>4624	0	0	0	0	0	0	0	0.624	0.624
IL1B	PLAU	9606.<br>ENSP0<br>00002<br>63341	9606.E<br>NSP00<br>00036<br>1850	0	0	0	0	0.076	0	0	0.61	0.625
IL1B	PPARA	9606.<br>ENSP0<br>00002<br>63341	9606.E<br>NSP00<br>00038<br>5523	0	0	0	0	0	0	0	0.65	0.65
IL1B	SPP1	9606.<br>ENSP0<br>00002<br>63341	9606.E<br>NSP00<br>00037<br>8517	0	0	0	0	0.061	0	0	0.708	0.714
IL1B	NOS3	9606.<br>ENSP0<br>00002<br>63341	9606.E<br>NSP00<br>00029<br>7494	0	0	0	0	0.062	0	0	0.723	0.729
IL1B	MAPK8	9606.<br>ENSP0<br>00002<br>63341	9606.E<br>NSP00<br>00037<br>8974	0	0	0	0	0	0	0	0.733	0.733
IL1B	STAT1	9606.<br>ENSP0<br>00002<br>63341	9606.E<br>NSP00<br>00035<br>4394	0	0	0	0	0.062	0	0	0.742	0.748
IL1B	PTGS1	9606.<br>ENSP0<br>00002<br>63341	9606.E<br>NSP00<br>00035<br>4612	0	0	0	0	0.156	0	0	0.723	0.756

IL1B	PPARG	9606.ENSP00000263341	9606.ENSP00000287820	0	0	0	0	0.061	0	0	0.789	0.794
IL1B	SELE	9606.ENSP00000263341	9606.ENSP00000331736	0	0	0	0	0.061	0	0	0.794	0.799
IL1B	NOS2	9606.ENSP00000263341	9606.ENSP00000327251	0	0	0	0	0.066	0	0	0.845	0.85
IL1B	MMP1	9606.ENSP00000263341	9606.ENSP00000322788	0	0	0	0	0.128	0	0	0.845	0.859
IL1B	VCAM1	9606.ENSP00000263341	9606.ENSP00000294728	0	0	0	0	0.062	0	0	0.865	0.868
IL1B	MMP3	9606.ENSP00000263341	9606.ENSP00000299855	0	0	0	0	0.107	0	0	0.862	0.872
IL1B	VEGFA	9606.ENSP00000263341	9606.ENSP00000478570	0	0	0	0	0	0	0	0.877	0.877
IL1B	MMP9	9606.ENSP00000263341	9606.ENSP00000361405	0	0	0	0	0.2	0	0	0.895	0.913
IL1B	RELA	9606.ENSP00000263341	9606.ENSP00000384273	0	0	0	0	0	0	0.9	0.559	0.954

IL1B	JUN	9606.ENSP000002 63341	9606.ENSP0000036 0266	0	0	0	0	0.052	0	0.8	0.82	0.963
IL1B	PTGS2	9606.ENSP000002 63341	9606.ENSP0000035 6438	0	0	0	0	0.561	0	0	0.943	0.974
IL1B	IL6	9606.ENSP000002 63341	9606.ENSP0000038 5675	0	0	0	0	0.43	0	0	0.973	0.984
IL2	MAPK1	9606.ENSP000002 26730	9606.ENSP0000021 5832	0	0	0	0	0	0	0.9	0.559	0.954
IL2	NFKBIA	9606.ENSP000002 26730	9606.ENSP0000021 6797	0	0	0	0	0	0	0	0.429	0.429
IL2	MMP2	9606.ENSP000002 26730	9606.ENSP0000021 9070	0	0	0	0	0	0	0	0.44	0.44
IL2	MPO	9606.ENSP000002 26730	9606.ENSP0000022 5275	0	0	0	0	0	0	0	0.548	0.548
IL2	SPP1	9606.ENSP000002 26730	9606.ENSP0000037 8517	0	0	0	0	0	0	0	0.4	0.4
IL2	TNFSF15	9606.ENSP000002 26730	9606.ENSP0000036 3157	0	0	0	0	0	0	0	0.413	0.412

IL2	MMP3	9606.ENSP0000226730	9606.ENSP000299855	0	0	0	0	0	0	0	0.43	0.43
IL2	NR3C1	9606.ENSP0000226730	9606.ENSP000231509	0	0	0	0	0	0	0	0.473	0.473
IL2	KDR	9606.ENSP0000226730	9606.ENSP000263923	0	0	0	0	0	0	0	0.473	0.473
IL2	NOS2	9606.ENSP0000226730	9606.ENSP000327251	0	0	0	0	0	0	0	0.486	0.486
IL2	IRF1	9606.ENSP0000226730	9606.ENSP000245414	0	0	0	0	0	0	0	0.496	0.495
IL2	PTGER3	9606.ENSP0000226730	9606.ENSP000349003	0	0	0	0	0	0	0	0.527	0.527
IL2	ODC1	9606.ENSP0000226730	9606.ENSP000234111	0	0	0	0	0	0	0	0.555	0.555
IL2	MMP9	9606.ENSP0000226730	9606.ENSP000361405	0	0	0	0	0	0	0	0.556	0.556
IL2	SELE	9606.ENSP0000226730	9606.ENSP000331736	0	0	0	0	0	0	0	0.575	0.575

IL2	PTGS2	9606.ENSP00000226730	9606.ENSP00000356438	0	0	0	0	0	0	0	0.577	0.577
IL2	VCAM1	9606.ENSP00000226730	9606.ENSP00000294728	0	0	0	0	0	0	0	0.627	0.627
IL2	VEGFA	9606.ENSP00000226730	9606.ENSP00000478570	0	0	0	0	0	0	0	0.733	0.733
IL2	MYC	9606.ENSP00000226730	9606.ENSP00000479618	0	0	0	0	0	0	0	0.792	0.792
IL2	MAPK14	9606.ENSP00000226730	9606.ENSP00000229795	0	0	0	0	0	0	0.9	0.44	0.941
IL2	IL6	9606.ENSP00000226730	9606.ENSP00000385675	0	0	0	0	0	0	0	0.958	0.959
IL2	IL4	9606.ENSP00000226730	9606.ENSP00000231449	0	0	0	0	0.062	0	0	0.963	0.964
IL2	STAT1	9606.ENSP00000226730	9606.ENSP00000354394	0	0	0	0	0	0	0.9	0.672	0.965
IL2	JUN	9606.ENSP00000226730	9606.ENSP00000360266	0	0	0	0	0	0.313	0.9	0.621	0.971

IL2	MAPK8	9606.ENSP000002 26730	9606.ENSP0000037 8974	0	0	0	0	0	0	0.9	0.769	0.975
IL2	RELA	9606.ENSP000002 26730	9606.ENSP0000038 4273	0	0	0	0	0	0.342	0.9	0.694	0.978
IL4	MAPK1	9606.ENSP000002 31449	9606.ENSP0000021 5832	0	0	0	0	0	0	0	0.55	0.55
IL4	MMP2	9606.ENSP000002 31449	9606.ENSP0000021 9070	0	0	0	0	0	0	0	0.473	0.473
IL4	SERPINE1	9606.ENSP000002 31449	9606.ENSP0000022 3095	0	0	0	0	0	0	0	0.42	0.42
IL4	MPO	9606.ENSP000002 31449	9606.ENSP0000022 5275	0	0	0	0	0	0	0	0.609	0.609
IL4	MAPK14	9606.ENSP000002 31449	9606.ENSP0000022 9795	0	0	0	0	0	0	0.9	0.641	0.962
IL4	NR3C1	9606.ENSP000002 31449	9606.ENSP0000023 1509	0	0	0	0	0	0	0	0.473	0.473
IL4	SPP1	9606.ENSP000002 31449	9606.ENSP0000037 8517	0	0	0	0	0	0	0	0.473	0.473

IL4	MMP3	9606.ENSP0000231449	9606.ENSP00000299855	0	0	0	0	0	0	0	0.5	0.5
IL4	PARP1	9606.ENSP0000231449	9606.ENSP00000355759	0	0	0	0	0	0	0	0.52	0.52
IL4	PPARG	9606.ENSP0000231449	9606.ENSP00000287820	0	0	0	0	0.062	0	0	0.51	0.52
IL4	ODC1	9606.ENSP0000231449	9606.ENSP00000234111	0	0	0	0	0	0	0	0.527	0.527
IL4	PLAU	9606.ENSP0000231449	9606.ENSP00000361850	0	0	0	0	0	0	0	0.541	0.541
IL4	IRF1	9606.ENSP0000231449	9606.ENSP00000245414	0	0	0	0	0	0	0	0.553	0.553
IL4	OPRM1	9606.ENSP0000231449	9606.ENSP00000394624	0	0	0	0	0	0	0	0.586	0.586
IL4	MAPK8	9606.ENSP0000231449	9606.ENSP00000378974	0	0	0	0	0	0	0	0.591	0.591
IL4	KDR	9606.ENSP0000231449	9606.ENSP00000263923	0	0	0	0	0	0	0	0.593	0.593

IL4	PTGS1	9606.ENSP0000231449	9606.ENSP00000354612	0	0	0	0	0.058	0	0	0.588	0.596
IL4	THBD	9606.ENSP0000231449	9606.ENSP00000366307	0	0	0	0	0	0	0	0.642	0.642
IL4	MMP9	9606.ENSP0000231449	9606.ENSP00000361405	0	0	0	0	0.062	0	0	0.641	0.648
IL4	NOS2	9606.ENSP0000231449	9606.ENSP00000327251	0	0	0	0	0.064	0	0	0.651	0.66
IL4	MMP1	9606.ENSP0000231449	9606.ENSP00000322788	0	0	0	0	0	0	0	0.685	0.685
IL4	MYC	9606.ENSP0000231449	9606.ENSP00000479618	0	0	0	0	0	0	0	0.71	0.71
IL4	VEGFA	9606.ENSP0000231449	9606.ENSP00000478570	0	0	0	0	0	0	0	0.726	0.726
IL4	SELE	9606.ENSP0000231449	9606.ENSP00000331736	0	0	0	0	0.055	0	0	0.739	0.743
IL4	PTGS2	9606.ENSP0000231449	9606.ENSP00000356438	0	0	0	0	0.062	0	0	0.795	0.8

IL4	STAT1	9606.ENSP00000231449	9606.ENSP00000354394	0	0	0	0	0	0	0	0.821	0.821
IL4	VCAM1	9606.ENSP00000231449	9606.ENSP00000294728	0	0	0	0	0	0	0	0.861	0.861
IL4	RELA	9606.ENSP00000231449	9606.ENSP00000384273	0	0	0	0	0	0	0.9	0.451	0.942
IL4	JUN	9606.ENSP00000231449	9606.ENSP00000360266	0	0	0	0	0	0	0.9	0.559	0.954
IL4	IL6	9606.ENSP00000231449	9606.ENSP00000385675	0	0	0	0	0	0	0.9	0.968	0.996
IL6	MAPK1	9606.ENSP00000385675	9606.ENSP00000215832	0	0	0	0	0	0	0.9	0.834	0.982
IL6	NFKBIA	9606.ENSP00000385675	9606.ENSP00000216797	0	0	0	0	0.109	0	0	0.67	0.693
IL6	MMP2	9606.ENSP00000385675	9606.ENSP00000219070	0	0	0	0	0.076	0	0	0.816	0.823
IL6	PLAT	9606.ENSP00000385675	9606.ENSP00000220809	0	0	0	0	0.076	0	0	0.529	0.546

IL6	PON1	9606.ENSP000003 85675	9606.ENSP0000022 2381	0	0	0	0	0	0	0	0.708	0.708
IL6	SERPINE1	9606.ENSP000003 85675	9606.ENSP0000022 3095	0	0	0	0	0.159	0	0	0.789	0.815
IL6	MPO	9606.ENSP000003 85675	9606.ENSP0000022 5275	0	0	0	0	0.061	0	0	0.848	0.852
IL6	MAPK14	9606.ENSP000003 85675	9606.ENSP0000022 9795	0	0	0	0	0	0	0	0.835	0.835
IL6	NR3C1	9606.ENSP000003 85675	9606.ENSP0000023 1509	0	0	0	0	0	0	0	0.646	0.646
IL6	OPRD1	9606.ENSP000003 85675	9606.ENSP0000023 4961	0	0	0	0	0	0	0	0.417	0.416
IL6	IRF1	9606.ENSP000003 85675	9606.ENSP0000024 5414	0	0	0	0	0.082	0	0	0.777	0.787
IL6	SLC6A4	9606.ENSP000003 85675	9606.ENSP0000026 1707	0	0	0	0	0	0	0	0.419	0.418
IL6	KDR	9606.ENSP000003 85675	9606.ENSP0000026 3923	0	0	0	0	0	0	0	0.59	0.59

IL6	SOD1	9606.ENSP000003 85675	9606.ENSP0000027 0142	0	0	0	0	0	0	0	0.541	0.541
IL6	MMP10	9606.ENSP000003 85675	9606.ENSP0000027 9441	0	0	0	0	0.117	0	0	0.731	0.753
IL6	PPARG	9606.ENSP000003 85675	9606.ENSP0000028 7820	0	0	0	0	0	0	0	0.812	0.812
IL6	VCAM1	9606.ENSP000003 85675	9606.ENSP0000029 4728	0	0	0	0	0.083	0	0	0.891	0.896
IL6	NOS3	9606.ENSP000003 85675	9606.ENSP0000029 7494	0	0	0	0	0.058	0	0	0.796	0.8
IL6	MMP3	9606.ENSP000003 85675	9606.ENSP0000029 9855	0	0	0	0	0.112	0	0	0.747	0.766
IL6	OLR1	9606.ENSP000003 85675	9606.ENSP0000030 9124	0	0	0	0	0.089	0	0	0.48	0.507
IL6	MET	9606.ENSP000003 85675	9606.ENSP0000031 7272	0	0	0	0	0.07	0	0	0.5	0.515
IL6	MMP1	9606.ENSP000003 85675	9606.ENSP0000032 2788	0	0	0	0	0.154	0	0	0.814	0.836

IL6	PGR	9606.<br>ENSP0<br>00003<br>85675	9606.E<br>NSP00<br>00032<br>5120	0	0	0	0	0.062	0	0	0.43	0.442
IL6	NOS2	9606.<br>ENSP0<br>00003<br>85675	9606.E<br>NSP00<br>00032<br>7251	0	0	0	0	0.063	0	0	0.735	0.741
IL6	SELE	9606.<br>ENSP0<br>00003<br>85675	9606.E<br>NSP00<br>00033<br>1736	0	0	0	0	0.12	0	0	0.79	0.807
IL6	SLPI	9606.<br>ENSP0<br>00003<br>85675	9606.E<br>NSP00<br>00034<br>2082	0	0	0	0	0.061	0	0	0.495	0.505
IL6	SREBF1	9606.<br>ENSP0<br>00003<br>85675	9606.E<br>NSP00<br>00034<br>8069	0	0	0	0	0	0	0	0.576	0.576
IL6	PTGER3	9606.<br>ENSP0<br>00003<br>85675	9606.E<br>NSP00<br>00034<br>9003	0	0	0	0	0	0	0	0.46	0.46
IL6	VCP	9606.<br>ENSP0<br>00003<br>85675	9606.E<br>NSP00<br>00035<br>1777	0	0	0	0	0	0	0	0.491	0.491
IL6	STAT1	9606.<br>ENSP0<br>00003<br>85675	9606.E<br>NSP00<br>00035<br>4394	0	0	0	0	0.058	0.163	0.9	0.818	0.983
IL6	PTGS1	9606.<br>ENSP0<br>00003<br>85675	9606.E<br>NSP00<br>00035<br>4612	0	0	0	0	0.094	0	0	0.53	0.556

IL6	PARP1	9606.ENSP00000385675	9606.ENSP00000355759	0	0	0	0	0	0	0	0.628	0.628
IL6	PTGS2	9606.ENSP00000385675	9606.ENSP00000356438	0	0	0	0	0.314	0	0	0.922	0.945
IL6	JUN	9606.ENSP00000385675	9606.ENSP00000360266	0	0	0	0	0.053	0	0.9	0.85	0.984
IL6	RUNX2	9606.ENSP00000385675	9606.ENSP00000360493	0	0	0	0	0	0	0	0.529	0.529
IL6	MMP9	9606.ENSP00000385675	9606.ENSP00000361405	0	0	0	0	0.074	0	0	0.92	0.923
IL6	PLAU	9606.ENSP00000385675	9606.ENSP00000361850	0	0	0	0	0.134	0	0	0.496	0.544
IL6	NOX1	9606.ENSP00000385675	9606.ENSP00000362057	0	0	0	0	0.061	0	0	0.448	0.46
IL6	TNFSF15	9606.ENSP00000385675	9606.ENSP00000363157	0	0	0	0	0.063	0	0	0.505	0.517
IL6	THBD	9606.ENSP00000385675	9606.ENSP00000366307	0	0	0	0	0.098	0	0	0.63	0.651

IL6	SPP1	9606. ENSP0 00003 85675	9606.E NSP00 00037 8517	0	0	0	0	0.06	0	0.9	0.793	0.978
IL6	MAPK8	9606. ENSP0 00003 85675	9606.E NSP00 00037 8974	0	0	0	0	0	0	0	0.885	0.885
IL6	NFE2L2	9606. ENSP0 00003 85675	9606.E NSP00 00038 0252	0	0	0	0	0	0	0	0.56	0.56
IL6	RELA	9606. ENSP0 00003 85675	9606.E NSP00 00038 4273	0	0	0	0	0	0.223	0.9	0.804	0.983
IL6	PPARA	9606. ENSP0 00003 85675	9606.E NSP00 00038 5523	0	0	0	0	0	0	0	0.517	0.517
IL6	PRKCA	9606. ENSP0 00003 85675	9606.E NSP00 00040 8695	0	0	0	0	0	0	0	0.558	0.558
IL6	OPRM1	9606. ENSP0 00003 85675	9606.E NSP00 00039 4624	0	0	0	0	0	0	0	0.655	0.655
IL6	MYC	9606. ENSP0 00003 85675	9606.E NSP00 00047 9618	0	0	0	0	0	0	0	0.85	0.85
IL6	VEGFA	9606. ENSP0 00003 85675	9606.E NSP00 00047 8570	0	0	0	0	0.063	0	0	0.957	0.959

IRF1	NFKBIA	9606.ENSP0000245414	9606.ENSP00000216797	0	0	0	0	0.274	0	0	0.473	0.601
IRF1	NR3C1	9606.ENSP0000245414	9606.ENSP00000231509	0	0	0	0	0	0	0.9	0.326	0.929
IRF1	SLPI	9606.ENSP0000245414	9606.ENSP00000342082	0	0	0	0	0	0	0	0.448	0.448
IRF1	PTGS2	9606.ENSP0000245414	9606.ENSP00000356438	0	0	0	0	0.089	0	0	0.424	0.453
IRF1	MYC	9606.ENSP0000245414	9606.ENSP00000479618	0	0	0	0	0	0	0	0.472	0.472
IRF1	SPP1	9606.ENSP0000245414	9606.ENSP00000378517	0	0	0	0	0	0	0	0.509	0.509
IRF1	VEGFA	9606.ENSP0000245414	9606.ENSP00000478570	0	0	0	0	0	0	0	0.553	0.553
IRF1	MAPK8	9606.ENSP0000245414	9606.ENSP00000378974	0	0	0	0	0	0	0	0.65	0.65
IRF1	NOS2	9606.ENSP0000245414	9606.ENSP00000327251	0	0	0	0	0	0	0	0.707	0.707

IRF1	JUN	9606.ENSP0000245414	9606.ENSP000360266	0	0	0	0	0.062	0	0.9	0.554	0.954
IRF1	VCAM1	9606.ENSP0000245414	9606.ENSP000294728	0	0	0	0	0.066	0	0.9	0.663	0.965
IRF1	STAT1	9606.ENSP0000245414	9606.ENSP000354394	0	0	0	0	0.387	0.379	0	0.932	0.972
IRF1	RELA	9606.ENSP0000245414	9606.ENSP000384273	0	0	0	0	0.062	0.379	0.9	0.709	0.98
JUN	MAPK1	9606.ENSP0000360266	9606.ENSP000215832	0	0	0	0	0	0.411	0.9	0.952	0.996
JUN	NFKBIA	9606.ENSP0000360266	9606.ENSP000216797	0	0	0	0	0.125	0	0.9	0.671	0.968
JUN	MMP2	9606.ENSP0000360266	9606.ENSP000219070	0	0	0	0	0	0	0	0.64	0.64
JUN	SERPINE1	9606.ENSP0000360266	9606.ENSP000223095	0	0	0	0	0.073	0	0	0.533	0.548
JUN	MPO	9606.ENSP0000360266	9606.ENSP000225275	0	0	0	0	0	0	0	0.457	0.457

JUN	MAPK14	9606.ENSP0000360266	9606.ENSP000229795	0	0	0	0	0	0.471	0.9	0.918	0.995
JUN	NR3C1	9606.ENSP0000360266	9606.ENSP000231509	0	0	0	0	0	0.472	0.9	0.739	0.985
JUN	ODC1	9606.ENSP0000360266	9606.ENSP000234111	0	0	0	0	0	0	0	0.506	0.506
JUN	KDR	9606.ENSP0000360266	9606.ENSP000263923	0	0	0	0	0	0.081	0	0.498	0.519
JUN	SOD1	9606.ENSP0000360266	9606.ENSP000270142	0	0	0	0	0	0	0	0.461	0.461
JUN	MMP10	9606.ENSP0000360266	9606.ENSP000279441	0	0	0	0	0	0	0	0.421	0.421
JUN	PPARG	9606.ENSP0000360266	9606.ENSP000287820	0	0	0	0	0	0.144	0.9	0.533	0.956
JUN	VCAM1	9606.ENSP0000360266	9606.ENSP000294728	0	0	0	0	0	0	0	0.499	0.499
JUN	NOS3	9606.ENSP0000360266	9606.ENSP000297494	0	0	0	0	0	0	0.9	0.542	0.952

JUN	MMP3	9606.ENSP000003 60266	9606.ENSP00000299855	0	0	0	0	0	0	0	0.754	0.754
JUN	MET	9606.ENSP000003 60266	9606.ENSP00000317272	0	0	0	0	0	0.157	0	0.454	0.52
JUN	MMP1	9606.ENSP000003 60266	9606.ENSP00000322788	0	0	0	0	0	0.379	0	0.641	0.767
JUN	PGR	9606.ENSP000003 60266	9606.ENSP00000325120	0	0	0	0	0	0.082	0	0.445	0.469
JUN	NOS2	9606.ENSP000003 60266	9606.ENSP00000327251	0	0	0	0	0	0	0.9	0.584	0.956
JUN	SELE	9606.ENSP000003 60266	9606.ENSP00000331736	0	0	0	0	0	0	0	0.44	0.44
JUN	SREBF1	9606.ENSP000003 60266	9606.ENSP00000348069	0	0	0	0	0.061	0	0	0.669	0.677
JUN	STAT1	9606.ENSP000003 60266	9606.ENSP00000354394	0	0	0	0	0	0.465	0	0.798	0.888
JUN	PARP1	9606.ENSP000003 60266	9606.ENSP00000355759	0	0	0	0	0.063	0.379	0	0.455	0.655

JUN	PTGS2	9606. ENSP0 00003 60266	9606.E NSP00 00035 6438	0	0	0	0	0.066	0	0	0.704	0.711
JUN	NOX1	9606. ENSP0 00003 60266	9606.E NSP00 00036 2057	0	0	0	0	0.061	0	0	0.39	0.403
JUN	PLAU	9606. ENSP0 00003 60266	9606.E NSP00 00036 1850	0	0	0	0	0	0	0	0.473	0.473
JUN	TNFSF15	9606. ENSP0 00003 60266	9606.E NSP00 00036 3157	0	0	0	0	0	0.379	0	0.217	0.492
JUN	PPP3CA	9606. ENSP0 00003 60266	9606.E NSP00 00037 8323	0	0	0	0	0	0.379	0	0.247	0.512
JUN	PRKCA	9606. ENSP0 00003 60266	9606.E NSP00 00040 8695	0	0	0	0	0	0	0	0.513	0.513
JUN	RUNX2	9606. ENSP0 00003 60266	9606.E NSP00 00036 0493	0	0	0	0	0	0.294	0	0.525	0.65
JUN	SPP1	9606. ENSP0 00003 60266	9606.E NSP00 00037 8517	0	0	0	0	0	0	0	0.681	0.681
JUN	MMP9	9606. ENSP0 00003 60266	9606.E NSP00 00036 1405	0	0	0	0	0	0.313	0	0.85	0.893

JUN	POR	9606.ENSP000003 60266	9606.ENSP0000041 9970	0	0	0	0	0	0	0.9	0.079	0.903
JUN	PPARA	9606.ENSP000003 60266	9606.ENSP0000038 5523	0	0	0	0	0	0.082	0.9	0.385	0.938
JUN	NFE2L2	9606.ENSP000003 60266	9606.ENSP0000038 0252	0	0	0	0	0	0.4	0.9	0.578	0.972
JUN	RELA	9606.ENSP000003 60266	9606.ENSP0000038 4273	0	0	0	0	0.069	0.457	0.9	0.667	0.98
JUN	VEGFA	9606.ENSP000003 60266	9606.ENSP0000047 8570	0	0	0	0	0.066	0.261	0.9	0.794	0.983
JUN	MYC	9606.ENSP000003 60266	9606.ENSP0000047 9618	0	0	0	0	0.063	0.379	0.9	0.878	0.992
JUN	MAPK8	9606.ENSP000003 60266	9606.ENSP0000037 8974	0	0	0	0	0	0.788	0.9	0.985	0.999
KDR	MAPK1	9606.ENSP000002 63923	9606.ENSP0000021 5832	0	0	0	0.601	0	0.408	0	0.628	0.553
KDR	MMP2	9606.ENSP000002 63923	9606.ENSP0000021 9070	0	0	0	0	0.098	0	0	0.648	0.668

KDR	SERPINE1	9606.ENSP0000263923	9606.ENSP00000223095	0	0	0	0	0.05	0	0	0.448	0.453
KDR	MAPK14	9606.ENSP0000263923	9606.ENSP00000229795	0	0	0	0.579	0	0.084	0.9	0.442	0.921
KDR	MMP3	9606.ENSP0000263923	9606.ENSP00000299855	0	0	0	0	0	0	0	0.426	0.426
KDR	PLAU	9606.ENSP0000263923	9606.ENSP00000361850	0	0	0	0	0.061	0	0	0.415	0.427
KDR	PGR	9606.ENSP0000263923	9606.ENSP00000325120	0	0	0	0	0	0.051	0	0.421	0.427
KDR	PPARA	9606.ENSP0000263923	9606.ENSP00000385523	0	0	0	0	0	0.051	0	0.454	0.459
KDR	SPP1	9606.ENSP0000263923	9606.ENSP00000378517	0	0	0	0	0.076	0	0	0.457	0.476
KDR	PTGS2	9606.ENSP0000263923	9606.ENSP00000356438	0	0	0	0	0	0	0	0.511	0.511
KDR	MYC	9606.ENSP0000263923	9606.ENSP00000479618	0	0	0	0	0	0	0	0.567	0.567

KDR	STAT1	9606.ENSP0000263923	9606.ENSP000354394	0	0	0	0	0	0.414	0	0.311	0.579
KDR	MET	9606.ENSP0000263923	9606.ENSP000317272	0	0	0	0.564	0	0.472	0	0.727	0.636
KDR	SELE	9606.ENSP0000263923	9606.ENSP000331736	0	0	0	0	0.128	0.124	0	0.571	0.643
KDR	MMP1	9606.ENSP0000263923	9606.ENSP000322788	0	0	0	0	0	0	0	0.655	0.655
KDR	MMP9	9606.ENSP0000263923	9606.ENSP000361405	0	0	0	0	0	0	0	0.677	0.677
KDR	NOS3	9606.ENSP0000263923	9606.ENSP000297494	0	0	0	0	0.062	0	0.9	0.832	0.982
KDR	VEGFA	9606.ENSP0000263923	9606.ENSP000478570	0	0	0	0	0.061	0.472	0.9	0.987	0.999
LYZ	MAPK1	9606.ENSP0000261267	9606.ENSP000215832	0	0	0	0	0	0	0.9	0.186	0.915
LYZ	MPO	9606.ENSP0000261267	9606.ENSP000225275	0	0	0	0	0.138	0	0.9	0.472	0.95

LYZ	NOS2	9606.ENSP0000261267	9606.ENSP000003327251	0	0	0	0	0.049	0.336	0	0.164	0.426
LYZ	VCP	9606.ENSP0000261267	9606.ENSP000003351777	0	0	0	0	0	0	0.9	0.082	0.904
LYZ	MMP9	9606.ENSP0000261267	9606.ENSP000003361405	0	0	0	0	0.134	0	0.9	0.251	0.929
LYZ	SLPI	9606.ENSP0000261267	9606.ENSP000003422082	0	0	0	0	0.096	0	0.9	0.387	0.939
MAPK1	PLAU	9606.ENSP0000215832	9606.ENSP000003361850	0	0	0	0	0	0.053	0	0.42	0.427
MAPK1	SPP1	9606.ENSP0000215832	9606.ENSP000003378517	0	0	0	0	0	0	0	0.455	0.455
MAPK1	SERPINE1	9606.ENSP0000215832	9606.ENSP000002223095	0	0	0	0	0	0	0	0.458	0.458
MAPK1	NOX1	9606.ENSP0000215832	9606.ENSP000003362057	0	0	0	0	0	0.124	0	0.437	0.486
MAPK1	VCAM1	9606.ENSP0000215832	9606.ENSP000002944728	0	0	0	0	0	0.044	0	0.499	0.501

MAPK1	NOS2	9606.ENSP0000215832	9606.ENSP00000327251	0	0	0	0	0.05	0	0	0.517	0.521
MAPK1	PPP3CA	9606.ENSP0000215832	9606.ENSP00000378323	0	0	0	0	0.063	0.302	0	0.333	0.526
MAPK1	SOD1	9606.ENSP0000215832	9606.ENSP00000270142	0	0	0	0	0	0.185	0	0.445	0.528
MAPK1	MMP3	9606.ENSP0000215832	9606.ENSP00000299855	0	0	0	0	0.049	0	0	0.524	0.528
MAPK1	NFE2L2	9606.ENSP0000215832	9606.ENSP00000380252	0	0	0	0	0	0.068	0	0.518	0.532
MAPK1	PLAT	9606.ENSP0000215832	9606.ENSP00000220809	0	0	0	0	0.05	0.408	0	0.262	0.549
MAPK1	SREBF1	9606.ENSP0000215832	9606.ENSP00000348069	0	0	0	0	0.055	0.393	0	0.306	0.567
MAPK1	PPARG	9606.ENSP0000215832	9606.ENSP00000287820	0	0	0	0	0	0.399	0	0.476	0.672
MAPK1	VEGFA	9606.ENSP0000215832	9606.ENSP00000478570	0	0	0	0	0	0	0	0.672	0.672

MAPK1	MMP1	9606.ENSP00000215832	9606.ENSP00000322788	0	0	0	0	0.049	0	0	0.714	0.717
MAPK1	NOS3	9606.ENSP00000215832	9606.ENSP00000297494	0	0	0	0	0.05	0	0	0.723	0.726
MAPK1	MMP2	9606.ENSP00000215832	9606.ENSP00000219070	0	0	0	0	0.049	0	0	0.743	0.746
MAPK1	PTGS2	9606.ENSP00000215832	9606.ENSP00000356438	0	0	0	0	0	0.266	0	0.678	0.753
MAPK1	MMP9	9606.ENSP00000215832	9606.ENSP00000361405	0	0	0	0	0.049	0	0	0.797	0.799
MAPK1	PARP1	9606.ENSP00000215832	9606.ENSP00000355759	0	0	0	0	0.063	0.379	0	0.688	0.802
MAPK1	MAPK14	9606.ENSP00000215832	9606.ENSP00000229795	0	0	0.412	0.926	0.1	0.379	0.8	0.92	0.889
MAPK1	MET	9606.ENSP00000215832	9606.ENSP00000317272	0	0	0	0.589	0.059	0.146	0.9	0.481	0.929
MAPK1	PRKCA	9606.ENSP00000215832	9606.ENSP00000408695	0	0	0	0.594	0.056	0.261	0.9	0.575	0.941

MAPK1	MPO	9606.ENSP0000215832	9606.ENSP00000225275	0	0	0	0	0	0.077	0.9	0.445	0.944
MAPK1	VCP	9606.ENSP0000215832	9606.ENSP00000351777	0	0	0	0	0.145	0.309	0.9	0.204	0.946
MAPK1	MAPK8	9606.ENSP0000215832	9606.ENSP00000378974	0	0	0.386	0.886	0.081	0.384	0.9	0.98	0.947
MAPK1	PPARA	9606.ENSP0000215832	9606.ENSP00000385523	0	0	0	0	0	0.399	0.9	0.264	0.951
MAPK1	RELA	9606.ENSP0000215832	9606.ENSP00000384273	0	0	0	0	0	0	0.9	0.541	0.952
MAPK1	PGR	9606.ENSP0000215832	9606.ENSP00000325120	0	0	0	0	0	0.399	0.9	0.354	0.957
MAPK1	NR3C1	9606.ENSP0000215832	9606.ENSP00000231509	0	0	0	0	0	0.399	0.9	0.413	0.961
MAPK1	NFKBIA	9606.ENSP0000215832	9606.ENSP00000216797	0	0	0	0	0	0.185	0.9	0.561	0.961
MAPK1	RUNX2	9606.ENSP0000215832	9606.ENSP00000360493	0	0	0	0	0	0	0.9	0.74	0.972

MAPK1	STAT1	9606.<br>ENSP0<br>00002<br>15832	9606.E<br>NSP00<br>00035<br>4394	0	0	0	0	0.06	0.263	0.9	0.649	0.972
MAPK1	MYC	9606.<br>ENSP0<br>00002<br>15832	9606.E<br>NSP00<br>00047<br>9618	0	0	0	0	0	0.379	0.9	0.798	0.986
MAPK14	NFKBIA	9606.<br>ENSP0<br>00002<br>29795	9606.E<br>NSP00<br>00021<br>6797	0	0	0	0	0	0.593	0.9	0.485	0.977
MAPK14	MMP2	9606.<br>ENSP0<br>00002<br>29795	9606.E<br>NSP00<br>00021<br>9070	0	0	0	0	0.049	0	0	0.656	0.659
MAPK14	MPO	9606.<br>ENSP0<br>00002<br>29795	9606.E<br>NSP00<br>00022<br>5275	0	0	0	0	0	0.077	0	0.446	0.467
MAPK14	NOX1	9606.<br>ENSP0<br>00002<br>29795	9606.E<br>NSP00<br>00036<br>2057	0	0	0	0	0	0.124	0	0.351	0.407
MAPK14	SREBF1	9606.<br>ENSP0<br>00002<br>29795	9606.E<br>NSP00<br>00034<br>8069	0	0	0	0	0	0.064	0	0.398	0.413
MAPK14	PARP1	9606.<br>ENSP0<br>00002<br>29795	9606.E<br>NSP00<br>00035<br>5759	0	0	0	0	0.058	0	0	0.404	0.415
MAPK14	SOD1	9606.<br>ENSP0<br>00002<br>29795	9606.E<br>NSP00<br>00027<br>0142	0	0	0	0	0	0.177	0	0.413	0.496

MAPK14	NOS2	9606.ENSP0000229795	9606.ENSP00000327251	0	0	0	0	0.05	0	0	0.5	0.505
MAPK14	PPP3CA	9606.ENSP0000229795	9606.ENSP00000378323	0	0	0	0	0.102	0.302	0	0.314	0.532
MAPK14	RUNX2	9606.ENSP0000229795	9606.ENSP00000360493	0	0	0	0	0	0	0	0.549	0.549
MAPK14	PPARG	9606.ENSP0000229795	9606.ENSP00000287820	0	0	0	0	0	0.073	0	0.573	0.588
MAPK14	MMP3	9606.ENSP0000229795	9606.ENSP00000299855	0	0	0	0	0.049	0	0	0.594	0.598
MAPK14	NOS3	9606.ENSP0000229795	9606.ENSP00000297494	0	0	0	0	0.05	0	0	0.608	0.611
MAPK14	VCAM1	9606.ENSP0000229795	9606.ENSP00000294728	0	0	0	0	0	0	0	0.619	0.619
MAPK14	MMP1	9606.ENSP0000229795	9606.ENSP00000322788	0	0	0	0	0.049	0	0	0.619	0.622
MAPK14	VEGFA	9606.ENSP0000229795	9606.ENSP00000478570	0	0	0	0	0	0	0	0.661	0.661

MAPK14	MMP9	9606.ENSP0000229795	9606.ENSP000000361405	0	0	0	0	0.049	0	0	0.695	0.698
MAPK14	PTGS2	9606.ENSP0000229795	9606.ENSP000000356438	0	0	0	0	0	0.086	0	0.774	0.785
MAPK14	PGR	9606.ENSP0000229795	9606.ENSP000000325120	0	0	0	0	0	0.073	0.8	0.228	0.844
MAPK14	NR3C1	9606.ENSP0000229795	9606.ENSP000000231509	0	0	0	0	0	0.073	0.9	0.347	0.934
MAPK14	MAPK8	9606.ENSP0000229795	9606.ENSP000000378974	0	0	0.396	0.924	0.064	0.386	0.9	0.937	0.943
MAPK14	RELA	9606.ENSP0000229795	9606.ENSP000000384273	0	0	0	0	0	0.106	0.9	0.465	0.948
MAPK14	MYC	9606.ENSP0000229795	9606.ENSP000000479618	0	0	0	0	0	0.139	0.9	0.46	0.949
MAPK14	STAT1	9606.ENSP0000229795	9606.ENSP000000354394	0	0	0	0	0.06	0.406	0.9	0.718	0.982
MAPK8	NFKBIA	9606.ENSP0000378974	9606.ENSP000000216797	0	0	0	0	0	0.261	0	0.714	0.78

MAPK8	MMP2	9606.ENSP000003 78974	9606.ENSP00000219070	0	0	0	0	0	0	0	0.812	0.813
MAPK8	SERPINE1	9606.ENSP000003 78974	9606.ENSP00000223095	0	0	0	0	0	0	0	0.72	0.72
MAPK8	MPO	9606.ENSP000003 78974	9606.ENSP00000225275	0	0	0	0	0	0	0	0.528	0.528
MAPK8	NR3C1	9606.ENSP000003 78974	9606.ENSP00000231509	0	0	0	0	0.054	0.379	0.9	0.476	0.965
MAPK8	SOD1	9606.ENSP000003 78974	9606.ENSP00000270142	0	0	0	0	0.042	0	0	0.734	0.734
MAPK8	PPARG	9606.ENSP000003 78974	9606.ENSP00000287820	0	0	0	0	0	0.379	0	0.722	0.82
MAPK8	VCAM1	9606.ENSP000003 78974	9606.ENSP00000294728	0	0	0	0	0	0	0	0.748	0.748
MAPK8	NOS3	9606.ENSP000003 78974	9606.ENSP00000297494	0	0	0	0	0.055	0	0	0.595	0.601
MAPK8	MMP3	9606.ENSP000003 78974	9606.ENSP00000299855	0	0	0	0	0	0	0	0.564	0.564

MAPK8	MET	9606.ENSP000003 78974	9606.ENSP00000317272	0	0	0	0.56	0.062	0.073	0.9	0.447	0.923
MAPK8	MMP1	9606.ENSP000003 78974	9606.ENSP00000322788	0	0	0	0	0	0	0	0.755	0.756
MAPK8	PGR	9606.ENSP000003 78974	9606.ENSP00000325120	0	0	0	0	0.061	0	0.8	0.34	0.865
MAPK8	NOS2	9606.ENSP000003 78974	9606.ENSP00000327251	0	0	0	0	0.055	0	0	0.574	0.58
MAPK8	SELE	9606.ENSP000003 78974	9606.ENSP00000331736	0	0	0	0	0	0	0	0.48	0.48
MAPK8	SREBF1	9606.ENSP000003 78974	9606.ENSP00000348069	0	0	0	0	0	0	0	0.692	0.693
MAPK8	STAT1	9606.ENSP000003 78974	9606.ENSP00000354394	0	0	0	0	0	0	0	0.804	0.805
MAPK8	PARP1	9606.ENSP000003 78974	9606.ENSP00000355759	0	0	0	0	0	0	0	0.735	0.735
MAPK8	PTGS2	9606.ENSP000003 78974	9606.ENSP00000356438	0	0	0	0	0	0	0	0.84	0.84

MAPK8	RUNX2	9606.ENSP0000378974	9606.ENSP000360493	0	0	0	0	0	0	0	0.706	0.706
MAPK8	MMP9	9606.ENSP0000378974	9606.ENSP000361405	0	0	0	0	0	0	0	0.833	0.834
MAPK8	PLAU	9606.ENSP0000378974	9606.ENSP000361850	0	0	0	0	0	0	0	0.632	0.632
MAPK8	NOX1	9606.ENSP0000378974	9606.ENSP000362057	0	0	0	0	0	0	0	0.539	0.539
MAPK8	SPP1	9606.ENSP0000378974	9606.ENSP000378517	0	0	0	0	0	0	0	0.511	0.511
MAPK8	OPRM1	9606.ENSP0000378974	9606.ENSP000394624	0	0	0	0	0.055	0.076	0	0.591	0.612
MAPK8	VEGFA	9606.ENSP0000378974	9606.ENSP000478570	0	0	0	0	0	0	0	0.817	0.817
MAPK8	NFE2L2	9606.ENSP0000378974	9606.ENSP000380252	0	0	0	0	0	0.34	0	0.74	0.821
MAPK8	MYC	9606.ENSP0000378974	9606.ENSP000479618	0	0	0	0	0	0.379	0	0.818	0.882

MAPK8	RELA	9606.ENSP00000378974	9606.ENSP00000384273	0	0	0	0	0	0	0.8	0.769	0.952
MET	MMP2	9606.ENSP00000317272	9606.ENSP00000219070	0	0	0	0	0.065	0	0	0.511	0.523
MET	PTGS2	9606.ENSP00000317272	9606.ENSP00000356438	0	0	0	0	0.061	0.058	0	0.4	0.422
MET	PLAU	9606.ENSP00000317272	9606.ENSP00000361850	0	0	0	0	0.088	0.05	0	0.462	0.493
MET	MMP9	9606.ENSP00000317272	9606.ENSP00000361405	0	0	0	0	0	0	0	0.546	0.546
MET	PARP1	9606.ENSP00000317272	9606.ENSP00000355759	0	0	0	0	0.086	0.057	0	0.555	0.583
MET	MYC	9606.ENSP00000317272	9606.ENSP00000479618	0	0	0	0	0.05	0.157	0	0.582	0.636
MET	SPP1	9606.ENSP00000317272	9606.ENSP00000378517	0	0	0	0	0.061	0.079	0	0.633	0.655
MET	VEGFA	9606.ENSP00000317272	9606.ENSP00000478570	0	0	0	0	0.067	0	0.6	0.826	0.929

MMP1	MMP2	9606.ENSP0000322788	9606.ENSP00000219070	0	0	0	0.869	0.154	0	0.9	0.921	0.922
MMP1	PLAT	9606.ENSP0000322788	9606.ENSP00000220809	0	0	0	0	0.137	0	0	0.445	0.501
MMP1	SERPINE1	9606.ENSP0000322788	9606.ENSP00000223095	0	0	0	0	0.303	0	0	0.576	0.692
MMP1	MPO	9606.ENSP0000322788	9606.ENSP00000225275	0	0	0	0	0	0	0	0.424	0.424
MMP1	MMP10	9606.ENSP0000322788	9606.ENSP00000279441	0	0	0.28	0.948	0.361	0	0.9	0.76	0.936
MMP1	PPARG	9606.ENSP0000322788	9606.ENSP00000287820	0	0	0	0	0	0	0	0.567	0.567
MMP1	VCAM1	9606.ENSP0000322788	9606.ENSP00000294728	0	0	0	0	0.065	0	0	0.537	0.548
MMP1	MMP3	9606.ENSP0000322788	9606.ENSP00000299855	0	0	0.298	0.953	0.529	0	0.9	0.921	0.953
MMP1	MYC	9606.ENSP0000322788	9606.ENSP00000479618	0	0	0	0	0	0	0	0.408	0.408

MMP1	SELE	9606.ENSP000003 22788	9606.ENSP0000033 1736	0	0	0	0	0.051	0	0	0.473	0.478
MMP1	SPP1	9606.ENSP000003 22788	9606.ENSP0000037 8517	0	0	0	0	0.049	0	0	0.516	0.52
MMP1	PLAU	9606.ENSP000003 22788	9606.ENSP0000036 1850	0	0	0	0	0.198	0	0	0.572	0.642
MMP1	PTGS2	9606.ENSP000003 22788	9606.ENSP0000035 6438	0	0	0	0	0.138	0	0	0.661	0.696
MMP1	VEGFA	9606.ENSP000003 22788	9606.ENSP0000047 8570	0	0	0	0	0.069	0	0	0.827	0.832
MMP1	MMP9	9606.ENSP000003 22788	9606.ENSP0000036 1405	0	0	0	0.75	0.518	0	0.9	0.91	0.961
MMP10	MMP2	9606.ENSP000002 79441	9606.ENSP0000021 9070	0	0	0	0.874	0.071	0	0.9	0.703	0.911
MMP10	SERPINE1	9606.ENSP000002 79441	9606.ENSP0000022 3095	0	0	0	0	0.098	0	0	0.45	0.483
MMP10	VCAM1	9606.ENSP000002 79441	9606.ENSP0000029 4728	0	0	0	0	0	0	0	0.409	0.409

MMP10	PLAU	9606.ENSP00000279441	9606.ENSP00000361850	0	0	0	0	0.097	0	0	0.403	0.438
MMP10	PTGS2	9606.ENSP00000279441	9606.ENSP00000356438	0	0	0	0	0.107	0	0	0.455	0.492
MMP10	VEGFA	9606.ENSP00000279441	9606.ENSP00000478570	0	0	0	0	0	0	0	0.685	0.685
MMP10	MMP9	9606.ENSP00000279441	9606.ENSP00000361405	0	0	0	0.786	0.53	0.379	0.9	0.83	0.973
MMP10	MMP3	9606.ENSP00000279441	9606.ENSP00000299855	0	0	0.448	0.98	0.311	0.825	0.9	0.805	0.987
MMP2	RUNX2	9606.ENSP00000219070	9606.ENSP00000360493	0	0	0	0	0	0	0	0.421	0.42
MMP2	PGR	9606.ENSP00000219070	9606.ENSP00000325120	0	0	0	0	0	0	0	0.42	0.42
MMP2	MPO	9606.ENSP00000219070	9606.ENSP00000225275	0	0	0	0	0	0	0	0.511	0.511
MMP2	SELE	9606.ENSP00000219070	9606.ENSP00000331736	0	0	0	0	0	0	0	0.516	0.516

MMP2	NOS3	9606.ENSP00000219070	9606.ENSP00000297494	0	0	0	0	0	0	0	0.572	0.572
MMP2	RELA	9606.ENSP00000219070	9606.ENSP00000384273	0	0	0	0	0	0.085	0	0.57	0.59
MMP2	VCAM1	9606.ENSP00000219070	9606.ENSP00000294728	0	0	0	0	0.111	0	0	0.583	0.613
MMP2	PLAT	9606.ENSP00000219070	9606.ENSP00000220809	0	0	0	0	0.158	0	0	0.581	0.632
MMP2	PPARG	9606.ENSP00000219070	9606.ENSP00000287820	0	0	0	0	0.061	0	0	0.65	0.658
MMP2	PTGS2	9606.ENSP00000219070	9606.ENSP00000356438	0	0	0	0	0.066	0	0	0.696	0.704
MMP2	SERPINE1	9606.ENSP00000219070	9606.ENSP00000223095	0	0	0	0	0.213	0	0	0.669	0.728
MMP2	PLAU	9606.ENSP00000219070	9606.ENSP00000361850	0	0	0	0	0.152	0	0	0.7	0.734
MMP2	MYC	9606.ENSP00000219070	9606.ENSP00000479618	0	0	0	0	0	0	0	0.734	0.734

MMP2	SPP1	9606.ENSP00000219070	9606.ENSP00000378517	0	0	0	0	0.076	0	0	0.793	0.801
MMP2	MMP9	9606.ENSP00000219070	9606.ENSP00000361405	0	0	0	0.929	0.061	0	0.9	0.958	0.908
MMP2	MMP3	9606.ENSP00000219070	9606.ENSP00000299855	0	0	0	0.88	0.181	0	0.9	0.892	0.923
MMP2	VEGFA	9606.ENSP00000219070	9606.ENSP00000478570	0	0	0	0	0.065	0	0.8	0.899	0.979
MMP3	PLAT	9606.ENSP00000299855	9606.ENSP00000220809	0	0	0	0	0.092	0	0	0.522	0.548
MMP3	SERPINE1	9606.ENSP00000299855	9606.ENSP00000223095	0	0	0	0	0.159	0.379	0	0.594	0.769
MMP3	MPO	9606.ENSP00000299855	9606.ENSP00000225275	0	0	0	0	0	0	0	0.456	0.456
MMP3	VCAM1	9606.ENSP00000299855	9606.ENSP00000294728	0	0	0	0	0.061	0	0	0.523	0.532
MMP3	MYC	9606.ENSP00000299855	9606.ENSP00000479618	0	0	0	0	0	0	0	0.409	0.409

MMP3	RUNX2	9606.ENSP0000299855	9606.ENSP00000360493	0	0	0	0	0	0	0	0.413	0.413
MMP3	SELE	9606.ENSP0000299855	9606.ENSP00000331736	0	0	0	0	0	0	0	0.464	0.464
MMP3	OLR1	9606.ENSP0000299855	9606.ENSP00000309124	0	0	0	0	0	0	0	0.61	0.61
MMP3	PLAU	9606.ENSP0000299855	9606.ENSP00000361850	0	0	0	0	0.098	0	0	0.593	0.618
MMP3	VEGFA	9606.ENSP0000299855	9606.ENSP00000478570	0	0	0	0	0	0	0	0.669	0.669
MMP3	PTGS2	9606.ENSP0000299855	9606.ENSP00000356438	0	0	0	0	0.095	0	0	0.774	0.787
MMP3	PRKCA	9606.ENSP0000299855	9606.ENSP00000408695	0	0	0	0	0	0	0.9	0.152	0.911
MMP3	MMP9	9606.ENSP0000299855	9606.ENSP00000361405	0	0	0	0.791	0.518	0	0.9	0.911	0.959
MMP3	SPP1	9606.ENSP0000299855	9606.ENSP00000378517	0	0	0	0	0.062	0.379	0.9	0.578	0.972

MMP9	NFKBIA	9606.ENSP0000361405	9606.ENSP00000216797	0	0	0	0	0.092	0	0	0.525	0.55
MMP9	PLAT	9606.ENSP0000361405	9606.ENSP00000220809	0	0	0	0	0	0	0	0.817	0.817
MMP9	SERPINE1	9606.ENSP0000361405	9606.ENSP00000223095	0	0	0	0	0.061	0	0	0.707	0.714
MMP9	MPO	9606.ENSP0000361405	9606.ENSP00000225275	0	0	0	0	0.076	0	0	0.704	0.714
MMP9	SOD1	9606.ENSP0000361405	9606.ENSP00000270142	0	0	0	0	0	0	0	0.42	0.42
MMP9	PPARG	9606.ENSP0000361405	9606.ENSP00000287820	0	0	0	0	0	0	0	0.709	0.709
MMP9	VCAM1	9606.ENSP0000361405	9606.ENSP00000294728	0	0	0	0	0.061	0	0	0.69	0.696
MMP9	NOS3	9606.ENSP0000361405	9606.ENSP00000297494	0	0	0	0	0.062	0	0	0.778	0.783
MMP9	OLR1	9606.ENSP0000361405	9606.ENSP00000309124	0	0	0	0	0	0	0	0.424	0.424

MMP9	PGR	9606.ENSP0000361405	9606.ENSP00000325120	0	0	0	0	0	0	0	0.473	0.473
MMP9	NOS2	9606.ENSP0000361405	9606.ENSP00000327251	0	0	0	0	0.064	0	0	0.519	0.53
MMP9	SELE	9606.ENSP0000361405	9606.ENSP00000331736	0	0	0	0	0	0	0	0.644	0.644
MMP9	SLPI	9606.ENSP0000361405	9606.ENSP00000342082	0	0	0	0	0.062	0	0	0.637	0.645
MMP9	PTGER3	9606.ENSP0000361405	9606.ENSP00000349003	0	0	0	0	0	0.064	0	0.569	0.579
MMP9	STAT1	9606.ENSP0000361405	9606.ENSP00000354394	0	0	0	0	0	0	0	0.572	0.572
MMP9	PTGS2	9606.ENSP0000361405	9606.ENSP00000356438	0	0	0	0	0.097	0	0	0.725	0.741
MMP9	RUNX2	9606.ENSP0000361405	9606.ENSP00000360493	0	0	0	0	0.061	0	0	0.723	0.729
MMP9	THBD	9606.ENSP0000361405	9606.ENSP00000366307	0	0	0	0	0.133	0	0	0.355	0.416

MMP9	NOX1	9606.ENSP00000361405	9606.ENSP00000362057	0	0	0	0	0.062	0	0	0.498	0.509
MMP9	MYC	9606.ENSP00000361405	9606.ENSP00000479618	0	0	0	0	0	0	0	0.664	0.664
MMP9	PLAU	9606.ENSP00000361405	9606.ENSP00000361850	0	0	0	0	0.076	0	0	0.713	0.724
MMP9	RELA	9606.ENSP00000361405	9606.ENSP00000384273	0	0	0	0	0	0.344	0	0.683	0.783
MMP9	SPP1	9606.ENSP00000361405	9606.ENSP00000378517	0	0	0	0	0.109	0	0	0.804	0.818
MMP9	VEGFA	9606.ENSP00000361405	9606.ENSP00000478570	0	0	0	0	0	0	0.8	0.946	0.989
MPO	PON1	9606.ENSP00000225275	9606.ENSP00000222381	0	0	0	0	0	0	0	0.575	0.575
MPO	SERPINE1	9606.ENSP00000225275	9606.ENSP00000223095	0	0	0	0	0	0	0	0.51	0.51
MPO	THBD	9606.ENSP00000225275	9606.ENSP00000366307	0	0	0	0	0	0	0	0.4	0.4

MPO	SLPI	9606.ENSP00000225275	9606.ENSP00000342082	0	0	0	0	0	0	0	0.415	0.414
MPO	SOD1	9606.ENSP00000225275	9606.ENSP00000270142	0	0	0	0	0.061	0	0	0.417	0.429
MPO	NFE2L2	9606.ENSP00000225275	9606.ENSP00000380252	0	0	0	0	0	0	0	0.448	0.448
MPO	PPARG	9606.ENSP00000225275	9606.ENSP00000287820	0	0	0	0	0.061	0.05	0	0.452	0.468
MPO	NOX1	9606.ENSP00000225275	9606.ENSP00000362057	0	0	0	0	0.049	0.085	0	0.519	0.545
MPO	VEGFA	9606.ENSP00000225275	9606.ENSP00000478570	0	0	0	0	0.058	0	0	0.58	0.587
MPO	NOS2	9606.ENSP00000225275	9606.ENSP00000327251	0	0	0	0	0	0.157	0	0.595	0.644
MPO	VCAM1	9606.ENSP00000225275	9606.ENSP00000294728	0	0	0	0	0	0	0	0.659	0.659
MPO	SELE	9606.ENSP00000225275	9606.ENSP00000331736	0	0	0	0	0	0.051	0	0.671	0.675

MPO	NOS3	9606.ENSP0000225275	9606.ENSP00000297494	0	0	0	0	0	0.157	0	0.633	0.677
MPO	PTGS2	9606.ENSP0000225275	9606.ENSP00000356438	0	0	0	0	0.065	0	0	0.691	0.699
MPO	VCP	9606.ENSP0000225275	9606.ENSP00000351777	0	0	0	0	0	0	0.9	0.065	0.902
MPO	PTGS1	9606.ENSP0000225275	9606.ENSP00000354612	0	0	0	0	0.076	0	0.9	0.408	0.94
MYC	NFKBIA	9606.ENSP0000479618	9606.ENSP00000216797	0	0	0	0	0.057	0	0.9	0.445	0.943
MYC	SERPINE1	9606.ENSP0000479618	9606.ENSP00000223095	0	0	0	0	0.096	0	0	0.44	0.472
MYC	NR3C1	9606.ENSP0000479618	9606.ENSP00000231509	0	0	0	0	0	0	0	0.514	0.514
MYC	ODC1	9606.ENSP0000479618	9606.ENSP00000234111	0	0	0	0	0.094	0.192	0	0.81	0.849
MYC	TP63	9606.ENSP0000479618	9606.ENSP00000264731	0	0	0	0	0	0.129	0	0.445	0.496

MYC	SOD1	9606.ENSP00000479618	9606.ENSP00000270142	0	0	0	0	0	0	0	0.402	0.402
MYC	PPARG	9606.ENSP00000479618	9606.ENSP00000287820	0	0	0	0	0	0	0	0.496	0.496
MYC	PGR	9606.ENSP00000479618	9606.ENSP00000325120	0	0	0	0	0	0.305	0	0.587	0.701
MYC	NOS2	9606.ENSP00000479618	9606.ENSP00000327251	0	0	0	0	0	0	0.9	0.222	0.918
MYC	SREBF1	9606.ENSP00000479618	9606.ENSP00000348069	0	0	0	0	0	0.05	0	0.715	0.718
MYC	STAT1	9606.ENSP00000479618	9606.ENSP00000354394	0	0	0	0	0	0	0	0.667	0.667
MYC	PARP1	9606.ENSP00000479618	9606.ENSP00000355759	0	0	0	0	0	0	0	0.754	0.755
MYC	PTGS2	9606.ENSP00000479618	9606.ENSP00000356438	0	0	0	0	0	0	0	0.57	0.57
MYC	RUNX2	9606.ENSP00000479618	9606.ENSP00000360493	0	0	0	0	0	0.176	0	0.531	0.597

MYC	PLAU	9606.ENSP000004796 18	9606.ENSP000003618 50	0	0	0	0	0.076	0.098	0	0.4	0.456
MYC	PPP3CA	9606.ENSP000004796 18	9606.ENSP000003783 23	0	0	0	0	0	0.05	0	0.453	0.458
MYC	SPP1	9606.ENSP000004796 18	9606.ENSP000003785 17	0	0	0	0	0	0	0	0.446	0.446
MYC	NFE2L2	9606.ENSP000004796 18	9606.ENSP000003802 52	0	0	0	0	0	0.405	0	0.673	0.797
MYC	RELA	9606.ENSP000004796 18	9606.ENSP000003842 73	0	0	0	0	0	0.379	0.9	0.587	0.972
MYC	PPARA	9606.ENSP000004796 18	9606.ENSP000003855 23	0	0	0	0	0	0	0.9	0.355	0.932
MYC	VEGFA	9606.ENSP000004796 18	9606.ENSP000004785 70	0	0	0	0	0	0	0	0.854	0.854
NFE2L2	SOD1	9606.ENSP000003802 52	9606.ENSP000002701 42	0	0	0	0	0	0	0	0.683	0.683
NFE2L2	PPARG	9606.ENSP000003802 52	9606.ENSP000002878 20	0	0	0	0	0	0.379	0	0.685	0.796

NFE2L2	NOS3	9606.ENSP000003 80252	9606.ENSP0000029 7494	0	0	0	0	0	0	0	0.472	0.472
NFE2L2	PTGS2	9606.ENSP000003 80252	9606.ENSP0000035 6438	0	0	0	0	0	0	0	0.57	0.57
NFE2L2	NR1I3	9606.ENSP000003 80252	9606.ENSP0000035 6959	0	0	0	0	0	0	0	0.445	0.445
NFE2L2	NOX1	9606.ENSP000003 80252	9606.ENSP0000036 2057	0	0	0	0	0	0	0	0.445	0.445
NFE2L2	VEGFA	9606.ENSP000003 80252	9606.ENSP0000047 8570	0	0	0	0	0	0	0	0.44	0.44
NFE2L2	POR	9606.ENSP000003 80252	9606.ENSP0000041 9970	0	0	0	0	0	0	0.9	0.097	0.905
NFE2L2	PRKCA	9606.ENSP000003 80252	9606.ENSP0000040 8695	0	0	0	0	0	0.379	0.9	0.145	0.942
NFKBIA	NR3C1	9606.ENSP000002 16797	9606.ENSP0000023 1509	0	0	0	0	0	0	0	0.417	0.416
NFKBIA	VEGFA	9606.ENSP000002 16797	9606.ENSP0000047 8570	0	0	0	0	0	0	0	0.423	0.422

NFKBIA	SELE	9606.ENSP000002167 97	9606.ENSP000003317 36	0	0	0	0	0	0	0	0.43	0.43
NFKBIA	PPARG	9606.ENSP000002167 97	9606.ENSP000002878 20	0	0	0	0	0.061	0	0	0.428	0.439
NFKBIA	VCP	9606.ENSP000002167 97	9606.ENSP000003517 77	0	0	0	0	0.055	0.402	0	0.095	0.444
NFKBIA	VCAM1	9606.ENSP000002167 97	9606.ENSP000002947 28	0	0	0	0	0	0	0	0.531	0.531
NFKBIA	STAT1	9606.ENSP000002167 97	9606.ENSP000003543 94	0	0	0	0	0.061	0.321	0	0.456	0.623
NFKBIA	PTGS2	9606.ENSP000002167 97	9606.ENSP000003564 38	0	0	0	0	0.076	0	0	0.794	0.801
NFKBIA	NOS2	9606.ENSP000002167 97	9606.ENSP000003272 51	0	0	0	0	0.055	0.064	0.9	0.54	0.953
NFKBIA	PRKCA	9606.ENSP000002167 97	9606.ENSP000004086 95	0	0	0	0	0	0.39	0.9	0.356	0.957
NFKBIA	PPARA	9606.ENSP000002167 97	9606.ENSP000003855 23	0	0	0	0	0	0	0.9	0.614	0.959

NFKBIA	RELA	9606.ENSP0000216797	9606.ENSP000003842 73	0	0	0	0	0.084	0.953	0.9	0.77	0.998
NOS2	ODC1	9606.ENSP0000327251	9606.ENSP000002341 11	0	0	0	0	0.061	0	0	0.653	0.662
NOS2	SOD1	9606.ENSP0000327251	9606.ENSP000002701 42	0	0	0	0	0	0	0	0.425	0.425
NOS2	PPARG	9606.ENSP0000327251	9606.ENSP000002878 20	0	0	0	0	0.062	0.091	0	0.417	0.459
NOS2	VCAM1	9606.ENSP0000327251	9606.ENSP000002947 28	0	0	0	0	0	0	0	0.668	0.668
NOS2	NOS3	9606.ENSP0000327251	9606.ENSP000002974 94	0	0	0.447	0.944	0	0	0.8	0.814	0.813
NOS2	SELE	9606.ENSP0000327251	9606.ENSP000003317 36	0	0	0	0	0.062	0	0	0.423	0.435
NOS2	OPRM1	9606.ENSP0000327251	9606.ENSP000003946 24	0	0	0	0	0	0	0	0.448	0.448
NOS2	PTGS1	9606.ENSP0000327251	9606.ENSP000003546 12	0	0	0	0	0	0.187	0	0.356	0.454

NOS2	PARP1	9606.ENSP00000327251	9606.ENSP00000355759	0	0	0	0	0.061	0	0	0.512	0.523
NOS2	VEGFA	9606.ENSP00000327251	9606.ENSP00000478570	0	0	0	0	0.064	0	0	0.527	0.538
NOS2	STAT1	9606.ENSP00000327251	9606.ENSP00000354394	0	0	0	0	0.061	0	0	0.741	0.747
NOS2	PTGS2	9606.ENSP00000327251	9606.ENSP00000356438	0	0	0	0	0.063	0.299	0	0.868	0.906
NOS2	PPARA	9606.ENSP00000327251	9606.ENSP00000385523	0	0	0	0	0	0.091	0.9	0.211	0.922
NOS2	RELA	9606.ENSP00000327251	9606.ENSP00000384273	0	0	0	0	0	0.343	0.9	0.327	0.951
NOS3	PLAT	9606.ENSP00000297494	9606.ENSP00000220809	0	0	0	0	0	0	0	0.445	0.445
NOS3	PON1	9606.ENSP00000297494	9606.ENSP00000222381	0	0	0	0	0	0	0	0.516	0.516
NOS3	SERPINE1	9606.ENSP00000297494	9606.ENSP00000223095	0	0	0	0	0.053	0	0	0.668	0.672

NOS3	NR3C1	9606.ENSP0000297494	9606.ENSP000231509	0	0	0	0	0	0.091	0	0.608	0.629
NOS3	SLC6A4	9606.ENSP0000297494	9606.ENSP000261707	0	0	0	0	0	0.261	0	0.237	0.412
NOS3	SOD1	9606.ENSP0000297494	9606.ENSP000270142	0	0	0	0	0	0	0	0.584	0.584
NOS3	PPARG	9606.ENSP0000297494	9606.ENSP000287820	0	0	0	0	0.053	0.091	0	0.741	0.757
NOS3	VCAM1	9606.ENSP0000297494	9606.ENSP000294728	0	0	0	0	0	0	0	0.83	0.83
NOS3	SPP1	9606.ENSP0000297494	9606.ENSP000378517	0	0	0	0	0	0	0	0.411	0.41
NOS3	RELA	9606.ENSP0000297494	9606.ENSP000384273	0	0	0	0	0	0.261	0	0.267	0.435
NOS3	PTGS1	9606.ENSP0000297494	9606.ENSP000354612	0	0	0	0	0	0.193	0	0.473	0.556
NOS3	THBD	9606.ENSP0000297494	9606.ENSP000366307	0	0	0	0	0.061	0	0	0.584	0.592

NOS3	PPARA	9606.ENSP00000297494	9606.ENSP00000385523	0	0	0	0	0.061	0.091	0	0.624	0.651
NOS3	NOX1	9606.ENSP00000297494	9606.ENSP00000362057	0	0	0	0	0.061	0	0	0.675	0.681
NOS3	SELE	9606.ENSP00000297494	9606.ENSP00000331736	0	0	0	0	0.078	0	0	0.669	0.681
NOS3	OLR1	9606.ENSP00000297494	9606.ENSP00000309124	0	0	0	0	0.055	0	0	0.772	0.775
NOS3	PTGS2	9606.ENSP00000297494	9606.ENSP00000356438	0	0	0	0	0	0.261	0	0.786	0.835
NOS3	VEGFA	9606.ENSP00000297494	9606.ENSP00000478570	0	0	0	0	0.062	0	0.9	0.934	0.993
NOX1	SOD1	9606.ENSP00000362057	9606.ENSP00000270142	0	0	0	0	0	0.19	0	0.585	0.65
NOX1	PPARG	9606.ENSP00000362057	9606.ENSP00000287820	0	0	0	0	0.055	0	0	0.555	0.562
NOX1	VCAM1	9606.ENSP00000362057	9606.ENSP00000294728	0	0	0	0	0.064	0	0	0.408	0.422

NOX1	PTGS2	9606.ENSP000003 62057	9606.ENSP0000035 6438	0	0	0	0	0.049	0.085	0	0.657	0.676
NOX1	VEGFA	9606.ENSP000003 62057	9606.ENSP0000047 8570	0	0	0	0	0	0	0	0.444	0.444
NR1I3	SREBF1	9606.ENSP000003 56959	9606.ENSP0000034 8069	0	0	0	0	0.057	0	0	0.39	0.4
NR1I3	POR	9606.ENSP000003 56959	9606.ENSP0000041 9970	0	0	0	0	0	0.091	0	0.417	0.447
NR3C1	SREBF1	9606.ENSP000002 31509	9606.ENSP0000034 8069	0	0	0	0	0	0	0	0.422	0.422
NR3C1	VEGFA	9606.ENSP000002 31509	9606.ENSP0000047 8570	0	0	0	0	0	0	0	0.47	0.47
NR3C1	PTGS2	9606.ENSP000002 31509	9606.ENSP0000035 6438	0	0	0	0	0.069	0.05	0	0.475	0.495
NR3C1	PPARG	9606.ENSP000002 31509	9606.ENSP0000028 7820	0	0	0	0.578	0.06	0.379	0	0.595	0.539
NR3C1	SLC6A4	9606.ENSP000002 31509	9606.ENSP0000026 1707	0	0	0	0	0	0.05	0	0.669	0.672

NR3C1	PGR	9606.ENSP00000231509	9606.ENSP00000325120	0	0	0	0.839	0	0	0.9	0.78	0.912
NR3C1	STAT1	9606.ENSP00000231509	9606.ENSP00000354394	0	0	0	0	0.065	0.053	0.9	0.4	0.939
NR3C1	RELA	9606.ENSP00000231509	9606.ENSP00000384273	0	0	0	0	0	0.393	0.9	0.443	0.963
ODC1	PTGS2	9606.ENSP00000234111	9606.ENSP00000356438	0	0	0	0	0	0	0	0.515	0.515
OLR1	PON1	9606.ENSP00000309124	9606.ENSP00000222381	0	0	0	0	0	0	0	0.433	0.433
OLR1	VCAM1	9606.ENSP00000309124	9606.ENSP00000294728	0	0	0	0	0.061	0	0	0.579	0.588
OLR1	SELE	9606.ENSP00000309124	9606.ENSP00000331736	0	0	0	0	0	0	0	0.481	0.481
OLR1	SPP1	9606.ENSP00000309124	9606.ENSP00000378517	0	0	0	0	0.061	0	0	0.534	0.544
OLR1	VEGFA	9606.ENSP00000309124	9606.ENSP00000478570	0	0	0	0	0	0	0	0.597	0.597

OLR1	PLAU	9606.ENSP0000309124	9606.ENSP00000361850	0	0	0	0	0	0	0.9	0.111	0.907
OPRD1	PRKCA	9606.ENSP0000234961	9606.ENSP00000408695	0	0	0	0	0.063	0.388	0	0.121	0.452
OPRD1	SLC6A4	9606.ENSP0000234961	9606.ENSP00000261707	0	0	0	0	0	0.073	0	0.538	0.553
OPRD1	PTGER3	9606.ENSP0000234961	9606.ENSP00000349003	0	0	0	0	0	0	0.9	0.316	0.928
OPRD1	OPRM1	9606.ENSP0000234961	9606.ENSP00000394624	0	0	0	0.961	0.069	0.447	0.9	0.832	0.945
OPRM1	SLC6A4	9606.ENSP0000394624	9606.ENSP00000261707	0	0	0	0	0	0.073	0	0.668	0.679
OPRM1	PTGER3	9606.ENSP0000394624	9606.ENSP00000349003	0	0	0	0	0	0	0.9	0.335	0.93
OPRM1	PARP1	9606.ENSP0000394624	9606.ENSP00000355759	0	0	0	0	0	0	0	0.416	0.416
OPRM1	PPP3CA	9606.ENSP0000394624	9606.ENSP00000378323	0	0	0	0	0	0.056	0	0.397	0.406

OPRM1	VEGFA	9606.ENSP00000394624	9606.ENSP00000478570	0	0	0	0	0	0	0	0.529	0.529
PARP1	PGR	9606.ENSP00000355759	9606.ENSP00000325120	0	0	0	0	0	0.32	0	0.361	0.547
PARP1	VEGFA	9606.ENSP00000355759	9606.ENSP00000478570	0	0	0	0	0	0	0	0.4	0.4
PARP1	RELA	9606.ENSP00000355759	9606.ENSP00000384273	0	0	0	0	0	0.379	0	0.616	0.751
PGR	SERPINE1	9606.ENSP00000325120	9606.ENSP00000223095	0	0	0	0	0	0.051	0	0.408	0.414
PGR	PLAU	9606.ENSP00000325120	9606.ENSP00000361850	0	0	0	0	0	0	0	0.416	0.416
PGR	RELA	9606.ENSP00000325120	9606.ENSP00000384273	0	0	0	0	0	0.393	0	0.217	0.505
PGR	PTGS2	9606.ENSP00000325120	9606.ENSP00000356438	0	0	0	0	0.061	0.05	0	0.573	0.585
PGR	VEGFA	9606.ENSP00000325120	9606.ENSP00000478570	0	0	0	0	0	0	0	0.63	0.63

PLAT	VCAM1	9606.ENSP000002 20809	9606.ENSP0000029 4728	0	0	0	0	0.087	0	0	0.477	0.503
PLAT	SELE	9606.ENSP000002 20809	9606.ENSP0000033 1736	0	0	0	0	0	0	0	0.511	0.511
PLAT	PLAU	9606.ENSP000002 20809	9606.ENSP0000036 1850	0	0	0.442	0.883	0.112	0.379	0	0.94	0.513
PLAT	VEGFA	9606.ENSP000002 20809	9606.ENSP0000047 8570	0	0	0	0	0.084	0	0	0.707	0.721
PLAT	THBD	9606.ENSP000002 20809	9606.ENSP0000036 6307	0	0	0	0	0.061	0.157	0	0.707	0.747
PLAT	SERPINE1	9606.ENSP000002 20809	9606.ENSP0000022 3095	0	0	0	0	0.148	0.881	0.9	0.956	0.999
PLAU	PON1	9606.ENSP000003 61850	9606.ENSP0000022 2381	0	0	0	0	0	0	0	0.501	0.501
PLAU	SERPINE1	9606.ENSP000003 61850	9606.ENSP0000022 3095	0	0	0	0	0.214	0.881	0.9	0.926	0.999
PLAU	VCAM1	9606.ENSP000003 61850	9606.ENSP0000029 4728	0	0	0	0	0.062	0	0	0.443	0.455

PLAU	PTGS2	9606.ENSP00000361850	9606.ENSP00000356438	0	0	0	0	0.102	0.057	0	0.481	0.522
PLAU	SPP1	9606.ENSP00000361850	9606.ENSP00000378517	0	0	0	0	0.075	0	0	0.442	0.462
PLAU	THBD	9606.ENSP00000361850	9606.ENSP00000366307	0	0	0	0	0.098	0.157	0	0.447	0.542
PLAU	VEGFA	9606.ENSP00000361850	9606.ENSP00000478570	0	0	0	0	0.069	0	0	0.666	0.675
PON1	SERPINE1	9606.ENSP00000222381	9606.ENSP00000223095	0	0	0	0	0	0	0	0.4	0.4
PON1	SOD1	9606.ENSP00000222381	9606.ENSP00000270142	0	0	0	0	0.057	0	0	0.39	0.4
PON1	PPARG	9606.ENSP00000222381	9606.ENSP00000287820	0	0	0	0	0.061	0	0	0.655	0.662
PPARA	SREBF1	9606.ENSP00000385523	9606.ENSP00000348069	0	0	0	0	0	0	0	0.751	0.751
PPARA	STAT1	9606.ENSP00000385523	9606.ENSP00000354394	0	0	0	0	0	0.321	0	0.265	0.48

PPARA	PTGS2	9606.ENSP0000385523	9606.ENSP00000356438	0	0	0	0	0	0.05	0	0.686	0.689
PPARA	SPP1	9606.ENSP0000385523	9606.ENSP00000378517	0	0	0	0	0	0	0	0.493	0.493
PPARA	RELA	9606.ENSP0000385523	9606.ENSP00000384273	0	0	0	0	0	0.393	0.9	0.21	0.947
PPARA	PRKCA	9606.ENSP0000385523	9606.ENSP00000408695	0	0	0	0	0	0.379	0	0.214	0.491
PPARG	SERPINE1	9606.ENSP0000287820	9606.ENSP00000223095	0	0	0	0	0.057	0.051	0	0.711	0.719
PPARG	SOD1	9606.ENSP0000287820	9606.ENSP00000270142	0	0	0	0	0	0	0	0.443	0.443
PPARG	PTGS1	9606.ENSP0000287820	9606.ENSP00000354612	0	0	0	0	0.059	0.05	0	0.407	0.423
PPARG	SPP1	9606.ENSP0000287820	9606.ENSP00000378517	0	0	0	0	0	0	0	0.518	0.518
PPARG	PRKCA	9606.ENSP0000287820	9606.ENSP00000408695	0	0	0	0	0	0.379	0	0.286	0.537

PPARG	VCAM1	9606.ENSP0000287820	9606.ENSP000294728	0	0	0	0	0	0	0	0.673	0.673
PPARG	RUNX2	9606.ENSP0000287820	9606.ENSP000360493	0	0	0	0	0	0.185	0	0.692	0.738
PPARG	STAT1	9606.ENSP0000287820	9606.ENSP000354394	0	0	0	0	0.061	0.321	0	0.638	0.749
PPARG	VEGFA	9606.ENSP0000287820	9606.ENSP000478570	0	0	0	0	0	0	0	0.769	0.769
PPARG	SREBF1	9606.ENSP0000287820	9606.ENSP000348069	0	0	0	0	0	0	0	0.813	0.813
PPARG	PTGS2	9606.ENSP0000287820	9606.ENSP000356438	0	0	0	0	0	0.05	0	0.817	0.819
PPARG	RELA	9606.ENSP0000287820	9606.ENSP000384273	0	0	0	0	0	0.472	0.9	0.348	0.962
PPP3CA	SOD1	9606.ENSP0000378323	9606.ENSP000270142	0	0	0	0	0	0.379	0.8	0.319	0.908
PPP3CA	PRKCA	9606.ENSP0000378323	9606.ENSP000408695	0	0	0	0	0.063	0.084	0.9	0.408	0.942

PRKCA	RELA	9606.ENSP00000408695	9606.ENSP000003824273	0	0	0	0	0	0	0.9	0.214	0.918
PRKCA	VEGFA	9606.ENSP00000408695	9606.ENSP000004788570	0	0	0	0	0	0	0	0.627	0.627
PTGER3	PTGS1	9606.ENSP00000349003	9606.ENSP000003544612	0	0	0	0	0	0.056	0	0.577	0.584
PTGER3	VEGFA	9606.ENSP00000349003	9606.ENSP000004788570	0	0	0	0	0	0	0	0.642	0.642
PTGER3	PTGS2	9606.ENSP00000349003	9606.ENSP000003566438	0	0	0	0	0	0.056	0	0.821	0.824
PTGS1	VEGFA	9606.ENSP00000354612	9606.ENSP000004788570	0	0	0	0	0.063	0	0	0.429	0.442
PTGS1	PTGS2	9606.ENSP00000354612	9606.ENSP000003566438	0	0	0.432	0.967	0.14	0.379	0.8	0.956	0.889
PTGS2	SERPINE1	9606.ENSP00000356438	9606.ENSP000002223095	0	0	0	0	0.118	0	0	0.517	0.556
PTGS2	SOD1	9606.ENSP00000356438	9606.ENSP000002770142	0	0	0	0	0.061	0	0	0.467	0.478

PTGS2	VCAM1	9606.ENSP00000356438	9606.ENSP00000294728	0	0	0	0	0.061	0	0	0.778	0.783
PTGS2	SELE	9606.ENSP00000356438	9606.ENSP00000331736	0	0	0	0	0.065	0.051	0	0.527	0.544
PTGS2	STAT1	9606.ENSP00000356438	9606.ENSP00000354394	0	0	0	0	0	0	0	0.704	0.704
PTGS2	RUNX2	9606.ENSP00000356438	9606.ENSP00000360493	0	0	0	0	0.076	0	0	0.406	0.428
PTGS2	SPP1	9606.ENSP00000356438	9606.ENSP00000378517	0	0	0	0	0.062	0	0	0.511	0.521
PTGS2	RELA	9606.ENSP00000356438	9606.ENSP00000384273	0	0	0	0	0	0	0	0.558	0.558
PTGS2	VEGFA	9606.ENSP00000356438	9606.ENSP00000478570	0	0	0	0	0.082	0	0	0.914	0.918
RELA	TP63	9606.ENSP00000384273	9606.ENSP00000264731	0	0	0	0	0	0.379	0	0.213	0.49
RELA	VCAM1	9606.ENSP00000384273	9606.ENSP00000294728	0	0	0	0	0	0.281	0	0.481	0.61

RELA	SELE	9606.ENSP0000384273	9606.ENSP00000331736	0	0	0	0	0	0.328	0	0.416	0.59
RELA	STAT1	9606.ENSP0000384273	9606.ENSP00000354394	0	0	0	0	0.065	0.513	0	0.563	0.783
RELA	VEGFA	9606.ENSP0000384273	9606.ENSP00000478570	0	0	0	0	0	0.313	0	0.477	0.625
RUNX2	VCAM1	9606.ENSP0000360493	9606.ENSP00000294728	0	0	0	0	0	0	0	0.42	0.42
RUNX2	STAT1	9606.ENSP0000360493	9606.ENSP00000354394	0	0	0	0	0	0	0	0.852	0.852
RUNX2	VEGFA	9606.ENSP0000360493	9606.ENSP00000478570	0	0	0	0	0	0	0	0.806	0.806
RUNX2	SPP1	9606.ENSP0000360493	9606.ENSP00000378517	0	0	0	0	0.062	0	0	0.94	0.942
SELE	SERPINE1	9606.ENSP0000331736	9606.ENSP00000223095	0	0	0	0	0.061	0	0	0.67	0.676
SELE	VCAM1	9606.ENSP0000331736	9606.ENSP00000294728	0	0	0	0	0.12	0	0	0.926	0.932

SELE	SPP1	9606.ENSP000003 31736	9606.ENSP0000037 8517	0	0	0	0	0	0	0	0.441	0.441
SELE	TNFSF15	9606.ENSP000003 31736	9606.ENSP0000036 3157	0	0	0	0	0	0.185	0	0.497	0.573
SELE	THBD	9606.ENSP000003 31736	9606.ENSP0000036 6307	0	0	0	0	0.076	0	0	0.7	0.71
SELE	VEGFA	9606.ENSP000003 31736	9606.ENSP0000047 8570	0	0	0	0	0	0	0	0.82	0.82
SERPINE	SREBF1	9606.ENSP000002 23095	9606.ENSP0000034 8069	0	0	0	0	0	0	0	0.422	0.422
SERPINE	SPP1	9606.ENSP000002 23095	9606.ENSP0000037 8517	0	0	0	0	0.084	0	0	0.518	0.539
SERPINE	VCAM1	9606.ENSP000002 23095	9606.ENSP0000029 4728	0	0	0	0	0.062	0	0	0.679	0.686
SERPINE	THBD	9606.ENSP000002 23095	9606.ENSP0000036 6307	0	0	0	0	0.095	0	0	0.738	0.752
SERPINE	VEGFA	9606.ENSP000002 23095	9606.ENSP0000047 8570	0	0	0	0	0.076	0	0.9	0.821	0.982

SLPI	STAT1	9606.ENSP0000342082	9606.ENSP00000354394	0	0	0	0	0	0	0	0.476	0.476
SOD1	VEGFA	9606.ENSP0000270142	9606.ENSP00000478570	0	0	0	0	0.063	0	0	0.709	0.716
SOD1	VCP	9606.ENSP0000270142	9606.ENSP00000351777	0	0	0	0	0.065	0.044	0	0.717	0.725
SPP1	VCAM1	9606.ENSP0000378517	9606.ENSP00000294728	0	0	0	0	0.07	0	0	0.586	0.598
SPP1	VEGFA	9606.ENSP0000378517	9606.ENSP00000478570	0	0	0	0	0	0	0	0.882	0.882
STAT1	VCAM1	9606.ENSP0000354394	9606.ENSP00000294728	0	0	0	0	0.062	0	0.9	0.472	0.946
STAT1	VEGFA	9606.ENSP0000354394	9606.ENSP00000478570	0	0	0	0	0	0.062	0	0.743	0.749
THBD	VCAM1	9606.ENSP0000366307	9606.ENSP00000294728	0	0	0	0	0.061	0	0	0.671	0.677
THBD	VEGFA	9606.ENSP0000366307	9606.ENSP00000478570	0	0	0	0	0.065	0	0	0.53	0.541

TNFSF15	VEGFA	9606.ENSP0000363157	9606.ENSP00000478570	0	0	0	0	0	0	0	0.577	0.577
VCAM1	VEGFA	9606.ENSP0000294728	9606.ENSP00000478570	0	0	0	0	0.061	0	0	0.848	0.852

Table S9 | Key targets in the network.

Average	**Betweennes**	**ClosenessCe**	ClusteringCo	**Degr**	Eccer	EdgeC	Indeg	IsSingleNod	**name**	Neighborhc	Num	Numbe	Outde	Partr	Radia	selected
1.2214	0.05980276	0.81875	0.39025319	103	3	103	54	FALSE	IL6	44	0	103	49	0	1	FALSE
1.2366	0.05076883	0.80864198	0.40666667	100	2	100	3	FALSE	AKT1	44.92	0	100	97	0	1	FALSE
1.2748	0.04263488	0.78443114	0.42956327	95	2	95	3	FALSE	ALB	46.2526316	0	95	92	0	1	FALSE
1.2901	0.0420672	0.77514793	0.44322508	95	3	95	95	FALSE	VEGFA	46.7578947	0	95	0	0	1	FALSE
1.3282	0.02791816	0.75287356	0.4762278	88	2	88	49	FALSE	JUN	48.4090909	0	88	39	0	1	FALSE
1.374	0.01650736	0.72777778	0.52544414	82	2	82	73	FALSE	PTGS2	50.7682927	0	82	9	0	1	FALSE
1.3893	0.01472515	0.71978022	0.5203252	82	3	82	8	FALSE	CASP3	50.3048781	0	82	74	0	1	FALSE
1.3817	0.02163418	0.72375691	0.50092593	81	2	81	47	FALSE	MAPK1	49.654321	0	81	34	0	1	FALSE
1.3969	0.01568627	0.715847	0.52839506	81	3	81	57	FALSE	MMP9	50.5925926	0	81	24	0	1	FALSE
1.3893	0.0227678	0.71978022	0.50679012	81	3	81	21	FALSE	CXCL8	50.0123457	0	81	60	0	1	FALSE
1.3969	0.0185645	0.715847	0.51075949	80	3	80	21	FALSE	EGF	50.1875	0	80	59	0	1	FALSE
1.3893	0.02164188	0.71978022	0.49303798	80	2	80	25	FALSE	EGFR	49.1	0	80	55	0	1	FALSE
1.3969	0.01476487	0.715847	0.53943525	79	2	79	50	FALSE	MAPK8	51.5696203	0	79	29	0	1	FALSE
1.4046	0.01847901	0.71195652	0.50682651	78	2	78	57	FALSE	MYC	49.4615385	0	78	21	0	1	FALSE
1.4122	0.01685133	0.70810811	0.50582751	78	3	78	35	FALSE	IL1B	49.4358974	0	78	43	0	1	FALSE
1.458	0.02612128	0.68586387	0.52034429	72	3	72	25	FALSE	FOS	51.1527778	0	72	47	0	1	FALSE
1.4733	0.01027369	0.67875648	0.56729265	72	3	72	8	FALSE	CCL2	51.9583333	0	72	64	0	1	FALSE
1.4733	0.01141698	0.67875648	0.54929578	71	3	71	16	FALSE	CCND1	50.8732394	0	71	55	0	1	FALSE
1.4656	0.01516574	0.68229167	0.52298137	70	2	70	31	FALSE	ESR1	50.2571429	0	70	39	0	1	FALSE
1.5115	0.00812232	0.66161616	0.60696517	67	3	67	29	FALSE	IL10	54.2835821	0	67	38	0	1	FALSE
1.5115	0.00677569	0.66161616	0.62460425	67	3	67	50	FALSE	MMP2	54.9104478	0	67	17	0	1	FALSE
1.5191	0.00824885	0.65829146	0.59230769	65	3	65	41	FALSE	MAPK14	54.0461539	0	65	24	0	0.99	FALSE
1.5649	0.00414362	0.63902439	0.67344633	60	3	60	24	FALSE	ICAM1	56.6	0	60	36	0	0.99	FALSE
1.5573	0.00767975	0.64215686	0.59588627	58	2	58	22	FALSE	ERBB2	53.362069	0	58	36	0	0.99	FALSE
1.5802	0.00422741	0.63285024	0.67453116	58	3	58	28	FALSE	IL4	56.9655172	0	58	30	0	0.99	FALSE
1.5802	0.00620819	0.63285024	0.63581367	58	3	58	53	FALSE	SERPINE	55.4137931	0	58	5	0	0.99	FALSE
1.5878	0.00692462	0.62980769	0.60776942	57	3	57	45	FALSE	PPARG	55.3859649	0	57	12	0	0.99	FALSE
1.5725	0.00883245	0.63592233	0.55639098	57	3	57	39	FALSE	NOS3	52.2807018	0	57	18	0	0.99	FALSE
1.5802	0.00503678	0.63285024	0.65476191	57	3	57	52	FALSE	RELA	57.0526316	0	57	5	0	0.99	FALSE
1.5649	0.01500098	0.63902439	0.53634085	57	2	57	3	FALSE	AR	50.4912281	0	57	54	0	0.99	FALSE

1.5878	0.00543363	0.62980769	0.6538961	56	3	56	30	FALSE	IL2	56.3928571	0	56	26	0	0.99	FALSE
1.6031	0.00415484	0.62380952	0.65519481	56	3	56	55	FALSE	VCAM1	55.1428571	0	56	1	0	0.99	FALSE
1.5954	0.00484628	0.62679426	0.63434343	55	3	55	18	FALSE	CTNNB1	54.8	0	55	37	0	0.99	FALSE
1.6031	0.00355623	0.62380952	0.67946128	55	3	55	5	FALSE	BCL2L1	57.2	0	55	50	0	0.99	FALSE
1.5878	0.00799554	0.62980769	0.6338225	54	2	54	23	FALSE	HMOX1	56.4444444	0	54	31	0	0.99	FALSE
1.6107	0.00413813	0.62085308	0.66666667	54	3	54	52	FALSE	STAT1	57.5185185	0	54	2	0	0.99	FALSE
1.6107	0.00520697	0.62085308	0.6512928	54	3	54	9	FALSE	CASP8	56.4259259	0	54	45	0	0.99	FALSE
1.6107	0.00265037	0.62085308	0.73165618	54	3	54	10	FALSE	CD44	59.5740741	0	54	44	0	0.99	FALSE
1.6031	0.00515229	0.62380952	0.6850508	53	3	53	51	FALSE	SPP1	58.0377359	0	53	2	0	0.99	FALSE
1.6336	0.00182646	0.61214953	0.75339367	52	3	52	21	FALSE	IFNG	60.0384615	0	52	31	0	0.99	FALSE
1.6183	0.00511892	0.61792453	0.6372549	52	3	52	18	FALSE	HIF1A	56.9423077	0	52	34	0	0.99	FALSE
1.626	0.00270434	0.61502347	0.71764706	51	3	51	36	FALSE	MMP1	59.5686275	0	51	15	0	0.99	FALSE
1.6336	0.00930528	0.61214953	0.60979592	50	3	50	34	FALSE	MPO	52.94	0	50	16	0	0.99	FALSE
1.6489	0.00357295	0.60648148	0.69469388	50	3	50	36	FALSE	MMP3	58.28	0	50	14	0	0.99	FALSE
1.6489	0.00436314	0.60648148	0.65691489	48	3	48	15	FALSE	CDKN1A	55.375	0	48	33	0	0.99	FALSE
1.6565	0.00465589	0.60368664	0.62608696	46	3	46	38	FALSE	NR3C1	57.2173913	0	46	8	0	0.99	FALSE
1.6641	0.00167063	0.60091743	0.75072464	46	3	46	28	FALSE	KDR	61.4565217	0	46	18	0	0.99	FALSE
1.6794	0.00393041	0.59545455	0.64141414	45	3	45	6	FALSE	CAV1	56.9111111	0	45	39	0	0.99	FALSE
1.6794	0.00400109	0.59545455	0.65353535	45	3	45	6	FALSE	CRP	54.8	0	45	39	0	0.99	FALSE
1.687	0.00285782	0.59276018	0.74312896	44	3	44	7	FALSE	CXCL10	58.4772727	0	44	37	0	0.99	FALSE
1.6947	0.00305041	0.59009009	0.68770764	43	3	43	29	FALSE	NOS2	57.4186047	0	43	14	0	0.99	FALSE
1.7099	0.00249767	0.58482143	0.69435216	43	3	43	31	FALSE	NFKBIA	57.6976744	0	43	12	0	0.99	FALSE
1.7099	0.00205882	0.58482143	0.7530454	43	3	43	37	FALSE	SELE	57.3488372	0	43	6	0	0.99	FALSE
1.7099	0.00161334	0.58482143	0.75493612	42	3	42	9	FALSE	CASP9	61.0714286	0	42	33	0	0.99	FALSE
1.7099	0.00312966	0.58482143	0.68873403	42	3	42	35	FALSE	PLAU	58.0714286	0	42	7	0	0.99	FALSE
1.687	0.00772346	0.59276018	0.63414634	41	2	41	0	FALSE	AHR	57.195122	0	41	41	0	0.99	FALSE
1.7099	0.00176791	0.58482143	0.73717949	40	3	40	35	FALSE	PGR	60.65	0	40	5	0	0.99	FALSE
1.7176	0.00496616	0.58222222	0.61024182	38	3	38	36	FALSE	SOD1	55	0	38	2	0	0.99	FALSE
1.7557	7.10E-04	0.56956522	0.82882883	37	3	37	6	FALSE	CD40LG	63.2432432	0	37	31	0	0.99	FALSE
1.7328	0.00722372	0.57709251	0.63529412	35	2	35	26	FALSE	NFE2L2	58.3428571	0	35	9	0	0.99	FALSE
1.7557	0.0018905	0.56956522	0.74957983	35	3	35	5	FALSE	CXCL2	56.7428571	0	35	30	0	0.99	FALSE
1.771	0.00120215	0.56465517	0.77142857	35	3	35	14	FALSE	IL1A	57.7428571	0	35	21	0	0.99	FALSE

1.7634	0.00141606	0.56709957	0.75044563	34	3	34	20	FALSE	HSPB1	62.7058824	0	34	14	0	0.99	FALSE
1.7634	0.00125361	0.56709957	0.80035651	34	3	34	11	FALSE	CCNB1	59.2058824	0	34	23	0	0.99	FALSE
1.7634	0.00163304	0.56709957	0.79857398	34	3	34	30	FALSE	RUNX2	64.4411765	0	34	4	0	0.99	FALSE
1.771	0.00297261	0.56465517	0.60795455	33	3	33	15	FALSE	GSK3B	54	0	33	18	0	0.99	FALSE
1.7939	0.00144113	0.55744681	0.7405303	33	3	33	9	FALSE	F3	57.4242424	0	33	24	0	0.99	FALSE
1.771	7.34E-04	0.56465517	0.8125	33	3	33	20	FALSE	IRF1	61.3636364	0	33	13	0	0.99	FALSE
1.771	0.0015005	0.56465517	0.72916667	33	3	33	30	FALSE	PARP1	58.6060606	0	33	3	0	0.99	FALSE
1.7863	0.00121651	0.55982906	0.74621212	33	3	33	19	FALSE	IGFBP3	60.969697	0	33	14	0	0.99	FALSE
1.7786	5.97E-04	0.56223176	0.80443548	32	3	32	17	FALSE	IGF2	62.21875	0	32	15	0	0.99	FALSE
1.8092	2.67E-04	0.55274262	0.88669951	29	3	29	13	FALSE	GJA1	69.4827586	0	29	16	0	0.99	FALSE
1.8092	7.73E-04	0.55274262	0.83862434	28	3	28	9	FALSE	CCNA2	61.3928571	0	28	19	0	0.99	FALSE
1.8168	1.42E-04	0.55042017	0.91798942	28	3	28	20	FALSE	MET	67.6785714	0	28	8	0	0.99	FALSE
1.8092	0.0032025	0.55274262	0.71794872	27	3	27	13	FALSE	ESR2	63.8518519	0	27	14	0	0.99	FALSE
1.8397	9.87E-04	0.54356847	0.73219373	27	3	27	14	FALSE	IKBKB	60.3703704	0	27	13	0	0.99	FALSE
1.855	6.93E-04	0.53909465	0.78153846	26	3	26	7	FALSE	COL1A1	59.2692308	0	26	19	0	0.99	FALSE
1.8168	0.00379939	0.55042017	0.48615385	26	3	26	24	FALSE	PRKCA	48.8076923	0	26	2	0	0.99	FALSE
1.8397	0.00341992	0.54356847	0.69333333	25	3	25	25	FALSE	SREBF1	59.92	0	25	0	0	0.99	FALSE
1.8244	0.00516571	0.54811716	0.46333333	25	3	25	20	FALSE	OPRM1	44.6	0	25	5	0	0.99	FALSE
1.8397	0.00144626	0.54356847	0.61956522	24	3	24	18	FALSE	PPARA	56.75	0	24	6	0	0.99	FALSE
1.8321	0.00415157	0.54583333	0.57608696	24	3	24	11	FALSE	GNRH1	54.3333333	0	24	13	0	0.99	FALSE
1.8779	3.92E-04	0.53252033	0.83695652	24	3	24	7	FALSE	CASP7	57.4166667	0	24	17	0	0.99	FALSE
1.9008	0.00102301	0.52610442	0.74637681	24	3	24	3	FALSE	CXCL11	52.7916667	0	24	21	0	0.99	FALSE
1.855	5.88E-04	0.53909465	0.85375494	23	3	23	18	FALSE	NOX1	66.3478261	0	23	5	0	0.99	FALSE
1.8626	3.17E-04	0.53688525	0.86956522	23	3	23	1	FALSE	ALOX5	66.7391304	0	23	22	0	0.99	FALSE
1.855	0.01677915	0.53909465	0.58008658	22	3	22	12	FALSE	GSTP1	53.1363636	0	22	10	0	0.99	FALSE
1.8397	0.00698905	0.54356847	0.40692641	22	3	22	5	FALSE	CYP3A4	40.3636364	0	22	17	0	0.99	FALSE
1.8473	0.0046441	0.54132231	0.48051948	22	3	22	4	FALSE	CYP1A1	43.3636364	0	22	18	0	0.99	FALSE
1.9084	0.00261496	0.524	0.64935065	22	3	22	6	FALSE	CTSD	54	0	22	16	0	0.99	FALSE
1.8931	1.01E-04	0.52822581	0.91341991	22	3	22	16	FALSE	PLAT	62.9090909	0	22	6	0	0.99	FALSE
1.8779	2.57E-05	0.53252033	0.96666667	21	3	21	13	FALSE	MMP10	72.6190476	0	21	8	0	0.99	FALSE
1.9237	3.32E-04	0.51984127	0.87142857	21	3	21	19	FALSE	THBD	59.0476191	0	21	2	0	0.99	FALSE
1.8931	7.98E-04	0.52822581	0.69473684	20	3	20	5	FALSE	CHUK	58.55	0	20	15	0	0.99	FALSE

1.9466	6.67E-04	0.51372549	0.65263158	20	3	20	2	FALSE	BCL2	51.35	0	20	18	0	0.99	FALSE
1.8931	0.00201169	0.52822581	0.49019608	18	3	18	8	FALSE	G6PD	49.9444444	0	18	10	0	0.99	FALSE
1.9466	0.0045632	0.51372549	0.43137255	18	3	18	14	FALSE	OPRD1	30.7777778	0	18	4	0	0.99	FALSE
1.9084	0.00151294	0.524	0.5751634	18	3	18	2	FALSE	CRH	45.5	0	18	16	0	0.99	FALSE
1.916	3.93E-04	0.52191235	0.81045752	18	3	18	2	FALSE	BAX	59.1111111	0	18	16	0	0.99	FALSE
1.9542	3.42E-04	0.51171875	0.79738562	18	3	18	9	FALSE	E2F1	54.1111111	0	18	9	0	0.99	FALSE
1.916	0.00365729	0.52191235	0.51470588	17	3	17	8	FALSE	DRD2	37.3529412	0	17	9	0	0.99	FALSE
1.9008	0.00168441	0.52610442	0.63235294	17	3	17	0	FALSE	ADRB2	56.4705882	0	17	17	0	0.99	FALSE
2.0229	1.73E-04	0.49433962	0.88970588	17	3	17	2	FALSE	BIRC5	53.2941177	0	17	15	0	0.99	FALSE
1.9237	0.00435242	0.51984127	0.525	16	3	16	16	FALSE	SLC6A4	41.875	0	16	0	0	0.99	FALSE
1.9389	1.58E-04	0.51574803	0.86666667	16	3	16	10	FALSE	OLR1	61.625	0	16	6	0	0.99	FALSE
1.916	7.06E-04	0.52191235	0.675	16	3	16	13	FALSE	PTGER3	53.5	0	16	3	0	0.99	FALSE
1.9847	0.00650613	0.50384615	0.41904762	15	3	15	6	FALSE	GSTM1	33.2666667	0	15	9	0	0.99	FALSE
1.9313	9.32E-04	0.51778656	0.57142857	15	3	15	12	FALSE	PON1	47.6	0	15	3	0	0.99	FALSE
1.9847	1.40E-04	0.50384615	0.84761905	15	3	15	13	FALSE	PTGS1	61.8	0	15	2	0	0.99	FALSE
1.9695	4.42E-04	0.50775194	0.6043956	14	3	14	12	FALSE	PPP3CA	56.5	0	14	2	0	0.99	FALSE
1.9466	1.48E-04	0.51372549	0.79120879	14	3	14	13	FALSE	ODC1	68.2142857	0	14	1	0	0.99	FALSE
1.9466	0.00115961	0.51372549	0.65934066	14	3	14	13	FALSE	SLPI	59.9285714	0	14	1	0	0.99	FALSE
2.0305	1.69E-04	0.4924812	0.78205128	13	3	13	3	FALSE	COL3A1	55	0	13	10	0	0.99	FALSE
1.9542	5.22E-04	0.51171875	0.56060606	12	3	12	12	FALSE	VCP	50.25	0	12	0	0	0.99	FALSE
1.9695	1.57E-04	0.50775194	0.72727273	12	3	12	3	FALSE	HSF1	57	0	12	9	0	0.99	FALSE
2.1298	2.90E-04	0.46953405	0.56060606	12	3	12	12	FALSE	TP63	46.6666667	0	12	0	0	0.99	FALSE
2.0229	4.92E-04	0.49433962	0.54545455	11	3	11	0	FALSE	ACLY	44.4545455	0	11	11	0	0.99	FALSE
2.0305	0.00110061	0.4924812	0.52727273	11	3	11	5	FALSE	LYZ	44.6363636	0	11	6	0	0.99	FALSE
2.0916	1.10E-04	0.47810219	0.78181818	11	3	11	6	FALSE	HAS2	53	0	11	5	0	0.99	FALSE
2.0153	4.44E-05	0.49621212	0.88888889	9	3	9	7	FALSE	E2F2	55.2222222	0	9	2	0	0.99	FALSE
2.1298	8.44E-04	0.46953405	0.5	9	3	9	9	FALSE	POR	35.8888889	0	9	0	0	0.99	FALSE
2.229	1.80E-04	0.44863014	0.71428571	8	3	8	5	FALSE	HTR3A	25.625	0	8	3	0	0.99	FALSE
2.0305	1.57E-04	0.4924812	0.64285714	8	3	8	0	FALSE	BMPR2	55.375	0	8	8	0	0.99	FALSE
2.0382	1.14E-04	0.4906367	0.66666667	7	3	7	4	FALSE	GRM1	41.5714286	0	7	3	0	0.99	FALSE
2.0611	8.81E-06	0.48518519	0.90476191	7	3	7	4	FALSE	F7	62.1428571	0	7	3	0	0.99	FALSE
2.6183	9.41E-04	0.3819242	0.46666667	6	4	6	1	FALSE	ADH1B	15.1666667	0	6	5	0	0.98	FALSE

2.4427	1.16E-04	0.409375	0.53333333	6	3	6	4	FALSE	NR1I3	25.6666667	0	6	2	0	0.99	FALSE
2.374	1.57E-04	0.42122187	0.53333333	6	3	6	5	FALSE	HSD3B2	26.8333333	0	6	1	0	0.99	FALSE
2.1527	1.24E-05	0.46453901	0.86666667	6	3	6	0	FALSE	ELK1	68.5	0	6	6	0	0.99	FALSE
2.0687	0	0.48339483	1	6	3	6	5	FALSE	TNFSF15	75.3333333	0	6	1	0	0.99	FALSE
2.2137	4.21E-05	0.45172414	0.5	4	4	4	1	FALSE	HPSE	35.5	0	4	3	0	0.99	FALSE
2.7786	0	0.35989011	1	3	4	3	0	FALSE	ADH1A	14.3333333	0	3	3	0	0.98	FALSE
				33												

Self	shared r	Stress	Topological(
0	IL6	10356	0.33846154
0	AKT1	9102	0.34290076
0	ALB	8730	0.35307352
0	VEGFA	7948	0.36521382
0	JUN	6834	0.36953505
0	PTGS2	4624	0.38754422
0	CASP3	4432	0.3899603
0	MAPK1	5204	0.37904062
0	MMP9	4144	0.39219064
0	CXCL8	5138	0.38471035
0	EGF	4550	0.38605769
0	EGFR	5612	0.37480916
0	MAPK8	4400	0.39366122
0	MYC	5030	0.377569
0	IL1B	4320	0.38027613
0	FOS	5826	0.39348291
0	CCL2	3192	0.40592448
0	CCND1	3492	0.3943662
0	ESR1	3860	0.38364231
0	IL10	2600	0.42409049
0	MMP2	2358	0.42898787
0	MAPK14	2636	0.41896243
0	ICAM1	1572	0.4421875
0	ERBB2	2556	0.40734404
0	IL4	1594	0.4450431
0	SERPINI	1864	0.43292026
0	PPARG	1932	0.43270285
0	NOS3	2208	0.40215924
0	RELA	1704	0.44226846
0	AR	3862	0.38542922

0	IL2	1584	0.43715393
0	VCAM1	1472	0.43419573
0	CTNNB:	1626	0.4248062
0	BCL2L1	1422	0.446875
0	HMOX1	2368	0.43087362
0	STAT1	1236	0.44936343
0	CASP8	1700	0.44082755
0	CD44	1054	0.46542245
0	SPP1	1496	0.44644412
0	IFNG	816	0.47274379
0	HIF1A	1644	0.44141324
0	MMP1	1174	0.46177231
0	MPO	2000	0.4103876
0	MMP3	1232	0.45889764
0	CDKN1/	1462	0.42926357
0	NR3C1	1244	0.44013378
0	KDR	618	0.47640715
0	CAV1	1034	0.44461806
0	CRP	984	0.428125
0	CXCL10	846	0.45685369
0	NOS2	836	0.44858285
0	NFKBIA	632	0.45791805
0	SELE	638	0.4551495
0	CASP9	746	0.48087739
0	PLAU	952	0.45725534
0	AHR	2674	0.43660398
0	PGR	568	0.47015504
0	SOD1	1212	0.42307692
0	CD40LG	362	0.5019305
0	NFE2L2	2506	0.44536532
0	CXCL2	556	0.44330357
0	IL1A	366	0.45827664

0	HSPB1	400	0.48988971
0	CCNB1	432	0.46254596
0	RUNX2	514	0.50344669
0	GSK3B	834	0.421875
0	F3	350	0.45939394
0	IRF1	308	0.47940341
0	PARP1	448	0.45785985
0	IGFBP3	446	0.48388648
0	IGF2	252	0.48608398
0	GJA1	112	0.54710834
0	CCNA2	290	0.4796317
0	MET	66	0.53290214
0	ESR2	1322	0.4949756
0	IKBKB	476	0.48296296
0	COL1A1	288	0.47797767
0	PRKCA	796	0.3783542
0	SREBF1	1288	0.4752381
0	OPRM1	1054	0.34573643
0	PPARA	352	0.44335938
0	GNRH1	1206	0.42118863
0	CASP7	164	0.46680217
0	CXCL11	258	0.43993056
0	NOX1	166	0.52242383
0	ALOX5	128	0.52967564
0	GSTP1	3594	0.41512784
0	CYP3A4	1870	0.31048951
0	CYP1A1	1518	0.33615222
0	CTSD	450	0.44628099
0	PLAT	48	0.51145602
0	MMP10	18	0.57634165
0	THBD	62	0.49206349
0	CHUK	326	0.4684

0	BCL2	156	0.43516949
0	G6PD	570	0.39326334
0	OPRD1	970	0.25648148
0	CRH	422	0.364
0	BAX	180	0.47670251
0	E2F1	100	0.45471522
0	DRD2	1044	0.29882353
0	ADRB2	558	0.4446503
0	BIRC5	38	0.48012719
0	SLC6A4	1116	0.335
0	OLR1	42	0.50101626
0	PTGER3	198	0.42460318
0	GSTM1	1320	0.2819209
0	PON1	220	0.3808
0	PTGS1	32	0.52372881
0	PPP3CA	106	0.46694215
0	ODC1	46	0.55011521
0	SLPI	254	0.48329493
0	COL3A1	66	0.48245614
0	VCP	118	0.402
0	HSF1	36	0.46341463
0	TP63	60	0.45751634
0	ACLY	94	0.37995338
0	LYZ	176	0.38479624
0	HAS2	42	0.49074074
0	E2F2	36	0.46018519
0	POR	346	0.34179894
0	HTR3A	28	0.27553763
0	BMPR2	44	0.46533613
0	GRM1	28	0.34933974
0	F7	4	0.53571429
0	ADH1B	90	0.33703704

0	NR1I3	18	0.38636364
0	HSD3B2	32	0.35307018
0	ELK1	4	0.65238095
0	TNFSF1[illegible]	0	0.64942529
0	HPSE	16	0.3525
0	ADH1A	0	0.5308642

Table S10 | Details of GO enrichment analyses.

ID	Description	GeneRatio	BgRatio	pvalue	p.adjust	qvalue	geneID	Count
GO:0005126	cytokine rec	18/134	271/18352	1.26E-12	5.43E-10	3.63E-10	CASP3/CASI	18
GO:0004879	nuclear rece	10/134	52/18352	3.74E-12	5.43E-10	3.63E-10	PGR/AR/PP/	10
GO:0098531	ligand-activ	10/134	52/18352	3.74E-12	5.43E-10	3.63E-10	PGR/AR/PP/	10
GO:0005125	cytokine act	16/134	235/18352	1.67E-11	1.82E-09	1.22E-09	TNFSF15/VE	16
GO:0140297	DNA-bindir	18/134	347/18352	7.71E-11	6.73E-09	4.50E-09	BCL2/JUN/F	18
GO:0048018	receptor liga	20/134	487/18352	4.00E-10	2.91E-08	1.94E-08	TNFSF15/VE	20
GO:0030546	signaling re	20/134	492/18352	4.78E-10	2.98E-08	1.99E-08	TNFSF15/VE	20
GO:0061629	RNA polyme	15/134	267/18352	1.09E-09	5.94E-08	3.97E-08	JUN/PPARG	15
GO:0019902	phosphatas	13/134	194/18352	1.78E-09	8.62E-08	5.76E-08	BCL2/PPAR(	13
GO:0019903	protein pho	11/134	149/18352	1.22E-08	5.31E-07	3.55E-07	BCL2/PPAR(	11
GO:0001223	transcriptior	6/134	29/18352	5.60E-08	2.22E-06	1.49E-06	PGR/AR/REI	6
GO:0070491	repressing ti	8/134	74/18352	6.58E-08	2.39E-06	1.60E-06	BCL2/PPAR(	8
GO:0001221	transcriptior	7/134	51/18352	8.37E-08	2.81E-06	1.88E-06	PGR/AR/REI	7
GO:0005178	integrin bin	10/134	144/18352	1.03E-07	3.20E-06	2.14E-06	PRKCA/ICAI	10
GO:0019207	kinase regul	11/134	216/18352	5.40E-07	1.56E-05	1.04E-05	CASP3/GSTI	11
GO:0051879	Hsp90 prote	6/134	42/18352	5.72E-07	1.56E-05	1.04E-05	CYP1A1/AH	6
GO:0003707	steroid horn	5/134	26/18352	1.12E-06	2.87E-05	1.92E-05	PGR/ESR1/I	5
GO:0033613	activating tr	7/134	80/18352	1.93E-06	4.67E-05	3.12E-05	JUN/PPARG	7
GO:0097153	cysteine-typ	4/134	15/18352	3.49E-06	7.77E-05	5.20E-05	CASP9/CASI	4
GO:0044389	ubiquitin-lil	12/134	316/18352	3.56E-06	7.77E-05	5.20E-05	BCL2/JUN/C	12
GO:0004175	endopeptid	14/134	440/18352	4.20E-06	8.73E-05	5.84E-05	CASP9/CASI	14
GO:0002020	protease bir	8/134	137/18352	7.43E-06	0.0001424	9.52E-05	BCL2/CASP3	8
GO:0045236	CXCR chem	4/134	18/18352	7.68E-06	0.0001424	9.52E-05	CXCL8/CXCL	4
GO:0020037	heme bindir	8/134	138/18352	7.84E-06	0.0001424	9.52E-05	PTGS1/PTGS	8
GO:0019887	protein kina	9/134	185/18352	8.75E-06	0.00015254	0.00010201	CASP3/CCN	9
GO:0070851	growth fact	8/134	141/18352	9.18E-06	0.00015399	0.00010298	VEGFA/IL10/	8
GO:0008144	drug bindin	7/134	104/18352	1.11E-05	0.00018001	0.00012038	SLC6A4/PPA	7
GO:0042379	chemokine r	6/134	70/18352	1.21E-05	0.00018849	0.00012605	STAT1/CCL2	6
GO:0005496	steroid bind	7/134	106/18352	1.26E-05	0.00018995	0.00012703	PGR/AR/CY	7
GO:0046906	tetrapyrrole	8/134	148/18352	1.31E-05	0.00019027	0.00012724	PTGS1/PTGS	8

GO:0016709 oxidoreduct	5/134	45/18352	1.86E-05	0.00026172	0.00017503	NOS2/CYP3	5
GO:0001046 core promot	5/134	46/18352	2.08E-05	0.0002828	0.00018913	RELA/STAT1	5
GO:0016705 oxidoreduct	8/134	162/18352	2.52E-05	0.00032863	0.00021977	PTGS1/PTGS	8
GO:0032813 tumor necro	5/134	48/18352	2.56E-05	0.00032863	0.00021977	CASP3/CASI	5
GO:0008009 chemokine a	5/134	49/18352	2.84E-05	0.00035344	0.00023637	CCL2/CXCL8	5
GO:0016538 cyclin-deper	5/134	50/18352	3.13E-05	0.00037958	0.00025385	CASP3/CCN	5
GO:0004252 serine-type	8/134	169/18352	3.41E-05	0.00040204	0.00026887	PLAU/F7/MI	8
GO:0016209 antioxidant	6/134	86/18352	3.95E-05	0.0004527	0.00030275	PTGS1/PTGS	6
GO:0031072 heat shock p	7/134	127/18352	4.08E-05	0.00045621	0.00030509	BAX/CYP1A	7
GO:0097199 cysteine-typ	3/134	10/18352	4.40E-05	0.00047441	0.00031727	CASP9/CASI	3
GO:0051117 ATPase binc	6/134	88/18352	4.49E-05	0.00047441	0.00031727	PGR/AR/PPI	6
GO:0050661 NADP bindi	5/134	54/18352	4.57E-05	0.00047441	0.00031727	NOS2/POR/	5
GO:0051427 hormone re	8/134	177/18352	4.74E-05	0.00048072	0.00032149	PPARG/STA	8
GO:0051400 BH domain l	3/134	11/18352	6.02E-05	0.00058301	0.00038989	BCL2/BAX/E	3
GO:0070513 death doma	3/134	11/18352	6.02E-05	0.00058301	0.00038989	BCL2/BAX/E	3
GO:0019838 growth facto	7/134	136/18352	6.31E-05	0.00059834	0.00040015	EGFR/KDR/E	7
GO:0031625 ubiquitin pr	10/134	297/18352	6.60E-05	0.00061225	0.00040945	BCL2/JUN/C	10
GO:0008236 serine-type	8/134	187/18352	6.99E-05	0.00063448	0.00042431	PLAU/F7/MI	8
GO:0005164 tumor necro	4/134	31/18352	7.34E-05	0.0006529	0.00043663	CASP8/TNFS	4
GO:0017171 serine hydro	8/134	191/18352	8.10E-05	0.0007064	0.00047241	PLAU/F7/MI	8
GO:0016653 oxidoreduct	3/134	13/18352	0.00010319	0.00088217	0.00058996	NOS2/POR/	3
GO:0046982 protein hete	10/134	321/18352	0.00012531	0.0010507	0.00070267	BCL2/BAX/I	10
GO:0008083 growth facto	7/134	162/18352	0.00018841	0.00154991	0.00103651	VEGFA/IL10/	7
GO:0010181 FMN bindin	3/134	16/18352	0.00019883	0.00160538	0.00107361	NOS2/POR/	3
GO:0030331 estrogen rec	4/134	42/18352	0.0002453	0.00194452	0.00130042	PPARG/ESR	4
GO:0046332 SMAD bindi	5/134	79/18352	0.00028151	0.00219176	0.00146576	JUN/FOS/PA	5
GO:0019199 transmembr	5/134	80/18352	0.00029854	0.00228353	0.00152713	EGFR/KDR/E	5
GO:0001091 RNA polyme	3/134	19/18352	0.00033858	0.00254516	0.0017021	AR/AHR/ESI	3
GO:0001102 RNA polyme	4/134	47/18352	0.00038002	0.00280829	0.00187807	JUN/FOS/NI	4
GO:0008013 beta-cateni	5/134	85/18352	0.00039566	0.00283188	0.00189385	AR/ESR1/GS	5
GO:0004707 MAP kinase	3/134	20/18352	0.0003962	0.00283188	0.00189385	MAPK8/MAI	3
GO:0042277 peptide binc	9/134	308/18352	0.00043848	0.00308349	0.00206211	ADRB2/OPR	9

GO:0070888 E-box bindi	4/134	50/18352	0.00048245	0.00333246	0.00222861	PPARG/AHF	4
GO:0019209 kinase activa	5/134	89/18352	0.00048917	0.00333246	0.00222861	CDKN1A/EG	5
GO:0004601 peroxidase a	4/134	52/18352	0.00056078	0.00376155	0.00251557	PTGS1/PTGS	4
GO:0070412 R-SMAD bir	3/134	23/18352	0.00060573	0.00400147	0.00267602	JUN/FOS/PA	3
GO:0035257 nuclear horr	6/134	144/18352	0.00066257	0.00431163	0.00288344	PPARG/STA	6
GO:0000979 RNA polyme	3/134	24/18352	0.00068858	0.004415	0.00295257	RELA/STAT1	3
GO:0001085 RNA polyme	4/134	56/18352	0.00074387	0.00463326	0.00309853	AR/GSK3B/E	4
GO:0016684 oxidoreduct	4/134	56/18352	0.00074387	0.00463326	0.00309853	PTGS1/PTGS	4
GO:0004497 monooxyge	5/134	101/18352	0.00087065	0.00534651	0.00357553	NOS2/CYP3	5
GO:0031406 carboxylic ac	7/134	212/18352	0.00094609	0.0057291	0.00383139	NOS2/PPAR	7
GO:0097110 scaffold pro	4/134	60/18352	0.00096564	0.00576739	0.003857	CASP8/IKBK	4
GO:0004714 transmembr	4/134	61/18352	0.00102761	0.00605459	0.00404906	EGFR/KDR/E	4
GO:0004222 metalloendc	5/134	108/18352	0.00117651	0.00683946	0.00457395	MMP1/MMF	5
GO:0043177 organic acid	7/134	224/18352	0.00130143	0.0074661	0.00499302	NOS2/PPAR	7
GO:0004674 protein serir	10/134	435/18352	0.00135005	0.00764444	0.00511229	PRKCA/IKBK	10
GO:0001664 G protein-cc	8/134	293/18352	0.0014096	0.00787931	0.00526936	STAT1/CCL2	8
GO:0030291 protein serir	3/134	31/18352	0.00147325	0.00802921	0.00536961	CASP3/CDKI	3
GO:0051059 NF-kappaB	3/134	31/18352	0.00147325	0.00802921	0.00536961	RELA/GSK3E	3
GO:0042826 histone dea	5/134	115/18352	0.00155551	0.00837288	0.00559944	RELA/MAPK	5
GO:0051721 protein pho	3/134	32/18352	0.00161703	0.00859786	0.0057499	BCL2/AKT1/	3
GO:0033218 amide bindi	9/134	381/18352	0.00194936	0.01023999	0.00684808	ADRB2/OPR	9
GO:0019825 oxygen binc	3/134	36/18352	0.00227877	0.01161837	0.00776989	CYP3A4/CYI	3
GO:0004955 prostagland	2/134	10/18352	0.0022917	0.01161837	0.00776989	PPARG/PTG	2
GO:0018455 alcohol deh	2/134	10/18352	0.0022917	0.01161837	0.00776989	ADH1B/ADH	2
GO:0004954 prostanoid r	2/134	11/18352	0.00278759	0.01365606	0.00913261	PPARG/PTG	2
GO:0048407 platelet-der	2/134	11/18352	0.00278759	0.01365606	0.00913261	COL1A1/CO	2
GO:1990459 transferrin r	2/134	11/18352	0.00278759	0.01365606	0.00913261	IKBKB/CHUK	2
GO:0005504 fatty acid bi	3/134	39/18352	0.00287066	0.01390676	0.00930028	PPARG/GST	3
GO:0035258 steroid horn	4/134	81/18352	0.00292862	0.01403163	0.00938378	PPARG/ESR	4
GO:0030295 protein kina	4/134	82/18352	0.00306163	0.01450946	0.00970334	CDKN1A/EG	4
GO:0004861 cyclin-deper	2/134	12/18352	0.00332915	0.01511988	0.01011155	CASP3/CDKI	2
GO:0043176 amine bindi	2/134	12/18352	0.00332915	0.01511988	0.01011155	SLC6A4/HTF	2

GO:0043295 glutathione	2/134	12/18352	0.00332915	0.01511988	0.01011155	GSTP1/GSTM	2
GO:0051378 serotonin bi	2/134	12/18352	0.00332915	0.01511988	0.01011155	SLC6A4/HTR	2
GO:1900750 oligopeptid	2/134	13/18352	0.00391569	0.0176004	0.01177043	GSTP1/GSTM	2
GO:0140296 general tran	3/134	44/18352	0.00405124	0.0180239	0.01205365	AR/AHR/ESI	3
GO:0004712 protein serir	3/134	45/18352	0.00431769	0.01901527	0.01271663	AKT1/MAPK	3
GO:0051213 dioxygenase	4/134	91/18352	0.0044489	0.01939719	0.01297205	PTGS1/PTGS	4
GO:0050998 nitric-oxide	2/134	14/18352	0.00454653	0.01943418	0.01299678	SLC6A4/CAV	2
GO:1901338 catecholami	2/134	14/18352	0.00454653	0.01943418	0.01299678	ADRB2/DRD	2
GO:0004953 icosanoid re	2/134	15/18352	0.00522101	0.02210057	0.01477996	PPARG/PTG	2
GO:0005149 interleukin-	2/134	16/18352	0.00593846	0.02489585	0.01664933	IL1B/IL1A	2
GO:0016922 nuclear rece	4/134	101/18352	0.00642781	0.02669071	0.01784966	PPARG/ESR	4
GO:0035173 histone kina	2/134	17/18352	0.00669823	0.02755123	0.01842513	PRKCA/CCN	2
GO:0051087 chaperone b	4/134	104/18352	0.007119	0.02900824	0.01939952	BAX/SOD1/	4
GO:0004708 MAP kinase	2/134	18/18352	0.00749968	0.0302765	0.02024768	MAPK14/MA	2
GO:0016651 oxidoreduct	4/134	109/18352	0.00837699	0.03350798	0.02240876	NOS2/POR/	4
GO:0004745 retinol dehy	2/134	20/18352	0.00922505	0.03623533	0.02423271	ADH1B/ADH	2
GO:0005123 death recep	2/134	20/18352	0.00922505	0.03623533	0.02423271	CASP3/CASI	2
GO:0005507 copper ion b	3/134	60/18352	0.00961914	0.03744595	0.02504232	SOD1/IL1A/	3
GO:0004197 cysteine-typ	4/134	114/18352	0.00977256	0.03770653	0.02521658	CASP9/CASI	4
GO:0051428 peptide hor	2/134	21/18352	0.01014771	0.03881054	0.0259549	GNRH1/CRH	2
GO:0001227 DNA-bindir	7/134	335/18352	0.01159274	0.04373718	0.02924963	JUN/PPARG	7
GO:0016616 oxidoreduct	4/134	120/18352	0.0116365	0.04373718	0.02924963	ADH1B/HSD	4
GO:0001217 DNA-bindir	7/134	336/18352	0.01176935	0.04385844	0.02933073	JUN/PPARG	7
GO:0004860 protein kina	3/134	65/18352	0.01195937	0.04418885	0.0295517	CASP3/CDKI	3
GO:0002039 p53 binding	3/134	66/18352	0.01246315	0.0454499	0.03039503	GSK3B/TP63	3
GO:1901681 sulfur comp	6/134	262/18352	0.01259154	0.0454499	0.03039503	GSTP1/GSTM	6
GO:0008237 metallopept	5/134	189/18352	0.01261339	0.0454499	0.03039503	MMP1/MMF	5
GO:0019210 kinase inhib	3/134	69/18352	0.01404679	0.04921898	0.03291564	CASP3/CDKI	3
GO:0004190 aspartic-typ	2/134	25/18352	0.01422383	0.04921898	0.03291564	CASP3/CTSD	2
GO:0004364 glutathione	2/134	25/18352	0.01422383	0.04921898	0.03291564	GSTP1/GSTM	2
GO:0016702 oxidoreduct	2/134	25/18352	0.01422383	0.04921898	0.03291564	PTGS2/ALO	2
GO:0070330 aromatase a	2/134	25/18352	0.01422383	0.04921898	0.03291564	CYP3A4/CYI	2

Table S11 | Results of Molecular Docking

Core Compo	Target	PDB ID	Binding Energy (kcal/mol)
Quercetin	IL-6	1ALU	-5.38
	AKT1	4GV1	-6.51
Kaempferol	IL-6	1ALU	-5.95
	AKT1	4GV1	-8.44
Beta-sitoster	IL-6	1ALU	-7.97
	AKT1	4GV1	-9.23
Stigmasterol	AKT1	4GV1	-8.77
7-O-methylis	AKT1	4GV1	-6.17
Epiquinidine	AKT1	4GV1	-8.23
(S)-Scoulerin	AKT1	4GV1	-7.44
sesamin	AKT1	4GV1	-7.85
3-Hydroxym	AKT1	4GV1	-7.15

Table S12 | Details of KEGG pathway enrichment analyses.

ID	Description	GeneRatio	BgRatio	pvalue	p.adjust	qvalue	geneID	Count
hsa04933	AGE-RAGE s	29/127	100/8087	8.48E-30	1.96E-27	6.34E-28	BCL2/BAX/JU	29
hsa05418	Fluid shear st	31/127	139/8087	5.81E-28	6.71E-26	2.17E-26	BCL2/JUN/RE	31
hsa04657	IL-17 signalin	24/127	94/8087	3.16E-23	2.43E-21	7.88E-22	PTGS2/JUN/C	24
hsa04668	TNF signaling	25/127	112/8087	1.36E-22	7.86E-21	2.54E-21	PTGS2/JUN/C	25
hsa05167	Kaposi sarcom	30/127	193/8087	3.62E-22	1.67E-20	5.41E-21	PTGS2/BAX/	30
hsa05215	Prostate cance	23/127	97/8087	1.78E-21	6.86E-20	2.22E-20	BCL2/CASP9	23
hsa05161	Hepatitis B	27/127	162/8087	8.51E-21	2.81E-19	9.09E-20	BCL2/BAX/C	27
hsa05163	Human cytom	29/127	225/8087	4.28E-19	1.23E-17	3.99E-18	PTGS2/BAX/	29
hsa05142	Chagas diseas	20/127	102/8087	5.13E-17	1.32E-15	4.26E-16	JUN/CASP8/M	20
hsa05212	Pancreatic ca	18/127	76/8087	5.76E-17	1.33E-15	4.30E-16	BAX/CASP9/	18
hsa05160	Hepatitis C	23/127	157/8087	1.70E-16	3.56E-15	1.15E-15	BAX/CASP9/	23
hsa05205	Proteoglycans	25/127	205/8087	6.20E-16	1.17E-14	3.79E-15	CASP3/PRKC	25
hsa05219	Bladder cance	14/127	41/8087	6.59E-16	1.17E-14	3.79E-15	MMP1/EGFR	14
hsa05162	Measles	21/127	139/8087	2.09E-15	3.46E-14	1.12E-14	BCL2/BAX/C	21
hsa05169	Epstein-Barr	24/127	202/8087	4.64E-15	7.15E-14	2.31E-14	BCL2/BAX/C	24
hsa05145	Toxoplasmos	19/127	112/8087	5.58E-15	8.05E-14	2.60E-14	BCL2/CASP9	19
hsa01522	Endocrine res	18/127	98/8087	7.11E-15	9.66E-14	3.13E-14	BCL2/BAX/JU	18
hsa05210	Colorectal ca	17/127	86/8087	1.16E-14	1.49E-13	4.81E-14	BCL2/BAX/C	17
hsa04620	Toll-like rece	18/127	104/8087	2.12E-14	2.45E-13	7.93E-14	JUN/CASP8/I	18
hsa04625	C-type lectin	18/127	104/8087	2.12E-14	2.45E-13	7.93E-14	PTGS2/JUN/C	18
hsa04659	Th17 cell diff	18/127	107/8087	3.57E-14	3.93E-13	1.27E-13	JUN/RELA/IK	18
hsa05222	Small cell lun	17/127	92/8087	3.79E-14	3.98E-13	1.29E-13	PTGS2/BCL2	17
hsa04210	Apoptosis	19/127	136/8087	2.21E-13	2.22E-12	7.18E-13	BCL2/BAX/C	19
hsa04660	T cell recepto	17/127	104/8087	3.16E-13	3.04E-12	9.84E-13	JUN/RELA/IK	17
hsa05133	Pertussis	15/127	76/8087	4.77E-13	4.40E-12	1.42E-12	JUN/CASP3/M	15
hsa05140	Leishmaniasis	15/127	77/8087	5.84E-13	5.19E-12	1.68E-12	PTGS2/JUN/M	15
hsa04066	HIF-1 signali	17/127	109/8087	7.03E-13	6.02E-12	1.95E-12	BCL2/PRKCA	17
hsa04926	Relaxin signa	18/127	129/8087	1.02E-12	8.40E-12	2.72E-12	JUN/PRKCA/	18
hsa05225	Hepatocellula	20/127	168/8087	1.07E-12	8.56E-12	2.77E-12	BAX/PRKCA	20
hsa05164	Influenza A	20/127	171/8087	1.50E-12	1.16E-11	3.75E-12	BAX/CASP9/	20

hsa05166	Human T-cel	22/127	219/8087	2.34E-12	1.74E-11	5.64E-12	BAX/JUN/RE	22
hsa05213	Endometrial	13/127	58/8087	3.29E-12	2.38E-11	7.69E-12	BAX/CASP9/	13
hsa05235	PD-L1 expres	15/127	89/8087	5.40E-12	3.78E-11	1.22E-11	JUN/RELA/II	15
hsa05224	Breast cancer	18/127	147/8087	9.92E-12	6.74E-11	2.18E-11	PGR/BAX/JU	18
hsa04380	Osteoclast dif	17/127	128/8087	1.03E-11	6.83E-11	2.21E-11	JUN/PPARG/	17
hsa01521	EGFR tyrosin	14/127	79/8087	1.41E-11	9.05E-11	2.93E-11	BCL2/BAX/P	14
hsa04010	MAPK signal	24/127	294/8087	1.88E-11	1.17E-10	3.79E-11	JUN/CASP3/I	24
hsa04151	PI3K-Akt sig	26/127	354/8087	2.64E-11	1.60E-10	5.19E-11	BCL2/CASP9	26
hsa05135	Yersinia infec	17/127	137/8087	3.15E-11	1.87E-10	6.04E-11	JUN/RELA/II	17
hsa05152	Tuberculosis	19/127	180/8087	3.59E-11	2.07E-10	6.70E-11	BCL2/BAX/C	19
hsa05134	Legionellosis	12/127	57/8087	5.17E-11	2.91E-10	9.43E-11	CASP9/CASP	12
hsa04064	NF-kappa B s	15/127	104/8087	5.54E-11	3.05E-10	9.86E-11	PTGS2/BCL2	15
hsa05223	Non-small ce	13/127	72/8087	6.15E-11	3.30E-10	1.07E-10	BAX/CASP9/	13
hsa01524	Platinum dru	13/127	73/8087	7.38E-11	3.87E-10	1.25E-10	BCL2/BAX/C	13
hsa04658	Th1 and Th2	14/127	92/8087	1.20E-10	6.17E-10	2.00E-10	JUN/RELA/II	14
hsa05220	Chronic myel	13/127	76/8087	1.26E-10	6.30E-10	2.04E-10	BAX/RELA/I	13
hsa04932	Non-alcoholi	17/127	150/8087	1.36E-10	6.70E-10	2.17E-10	BAX/JUN/CA	17
hsa05144	Malaria	11/127	50/8087	2.06E-10	9.90E-10	3.20E-10	ICAM1/SELE	11
hsa04510	Focal adhesio	19/127	201/8087	2.47E-10	1.17E-09	3.77E-10	BCL2/JUN/PI	19
hsa04218	Cellular senes	17/127	156/8087	2.55E-10	1.18E-09	3.81E-10	RELA/AKT1/	17
hsa05171	Coronavirus	20/127	232/8087	4.18E-10	1.89E-09	6.12E-10	JUN/PRKCA/	20
hsa05170	Human immu	19/127	212/8087	6.18E-10	2.75E-09	8.88E-10	BCL2/BAX/C	19
hsa04917	Prolactin sign	12/127	70/8087	6.55E-10	2.80E-09	9.06E-10	RELA/AKT1/	12
hsa05120	Epithelial cell	12/127	70/8087	6.55E-10	2.80E-09	9.06E-10	JUN/CASP3/I	12
hsa04215	Apoptosis - m	9/127	32/8087	9.03E-10	3.79E-09	1.23E-09	BCL2/BAX/C	9
hsa04370	VEGF signali	11/127	59/8087	1.37E-09	5.64E-09	1.83E-09	PTGS2/CASP	11
hsa05132	Salmonella in	20/127	249/8087	1.46E-09	5.93E-09	1.92E-09	BCL2/BAX/JU	20
hsa05323	Rheumatoid a	13/127	93/8087	1.70E-09	6.79E-09	2.20E-09	JUN/MMP1/I	13
hsa04621	NOD-like rec	17/127	181/8087	2.61E-09	1.02E-08	3.31E-09	BCL2/JUN/C	17
hsa04915	Estrogen sign	15/127	138/8087	3.23E-09	1.24E-08	4.02E-09	PGR/OPRM1/	15
hsa05146	Amoebiasis	13/127	102/8087	5.45E-09	2.06E-08	6.68E-09	CASP3/PRKC	13
hsa05202	Transcription	17/127	192/8087	6.46E-09	2.41E-08	7.78E-09	BAX/PLAU/F	17

hsa04012	ErbB signalin	12/127	85/8087	6.64E-09	2.43E-08	7.87E-09	JUN/PRKCA/	12
hsa05165	Human papill	22/127	331/8087	7.17E-09	2.59E-08	8.38E-09	PTGS2/BAX/	22
hsa05226	Gastric cance	15/127	149/8087	9.38E-09	3.33E-08	1.08E-08	BCL2/BAX/A	15
hsa05130	Pathogenic E	17/127	197/8087	9.54E-09	3.34E-08	1.08E-08	BAX/CASP9/	17
hsa05206	MicroRNAs i	21/127	310/8087	1.18E-08	4.07E-08	1.32E-08	PTGS2/BCL2	21
hsa04068	FoxO signalir	14/127	131/8087	1.40E-08	4.74E-08	1.53E-08	IKBKB/AKT	14
hsa04115	p53 signaling	11/127	73/8087	1.44E-08	4.84E-08	1.57E-08	BCL2/BAX/C	11
hsa05321	Inflammatory	10/127	65/8087	5.51E-08	1.82E-07	5.88E-08	JUN/RELA/S	10
hsa05143	African trypa	8/127	37/8087	7.81E-08	2.54E-07	8.22E-08	PRKCA/ICAN	8
hsa05218	Melanoma	10/127	72/8087	1.50E-07	4.82E-07	1.56E-07	BAX/AKT1/E	10
hsa04630	JAK-STAT si	14/127	162/8087	2.09E-07	6.60E-07	2.14E-07	BCL2/AKT1/	14
hsa05214	Glioma	10/127	75/8087	2.23E-07	6.97E-07	2.26E-07	BAX/PRKCA	10
hsa05131	Shigellosis	17/127	246/8087	2.53E-07	7.79E-07	2.52E-07	BCL2/BAX/JU	17
hsa05022	Pathways of r	24/127	475/8087	2.70E-07	8.22E-07	2.66E-07	PTGS2/BCL2	24
hsa04919	Thyroid horn	12/127	121/8087	3.66E-07	1.10E-06	3.55E-07	CASP9/PRKC	12
hsa04662	B cell recepto	10/127	82/8087	5.25E-07	1.56E-06	5.03E-07	JUN/RELA/II	10
hsa05221	Acute myeloi	9/127	67/8087	8.67E-07	2.54E-06	8.20E-07	RELA/IKBKI	9
hsa04622	RIG-I-like rec	9/127	70/8087	1.27E-06	3.66E-06	1.18E-06	CASP8/RELA	9
hsa05216	Thyroid canc	7/127	37/8087	1.39E-06	3.96E-06	1.28E-06	BAX/PPARG	7
hsa04071	Sphingolipid	11/127	119/8087	2.33E-06	6.47E-06	2.09E-06	BCL2/BAX/P	11
hsa04722	Neurotrophin	11/127	119/8087	2.33E-06	6.47E-06	2.09E-06	BCL2/BAX/JU	11
hsa04014	Ras signaling	15/127	232/8087	3.13E-06	8.61E-06	2.79E-06	PRKCA/REL	15
hsa05010	Alzheimer dis	19/127	369/8087	4.25E-06	1.16E-05	3.74E-06	PTGS2/CASP	19
hsa04931	Insulin resista	10/127	108/8087	6.74E-06	1.81E-05	5.86E-06	RELA/IKBKI	10
hsa04062	Chemokine si	13/127	192/8087	9.02E-06	2.39E-05	7.75E-06	RELA/IKBKI	13
hsa05230	Central carbo	8/127	70/8087	1.24E-05	3.25E-05	1.05E-05	AKT1/EGFR/	8
hsa04912	GnRH signali	9/127	93/8087	1.38E-05	3.59E-05	1.16E-05	JUN/PRKCA/	9
hsa05231	Choline metal	9/127	98/8087	2.12E-05	5.44E-05	1.76E-05	JUN/PRKCA/	9
hsa04921	Oxytocin sign	11/127	154/8087	2.78E-05	7.07E-05	2.29E-05	PTGS2/JUN/I	11
hsa04934	Cushing synd	11/127	155/8087	2.96E-05	7.43E-05	2.40E-05	AHR/EGFR/C	11
hsa05416	Viral myocar	7/127	60/8087	3.86E-05	9.59E-05	3.10E-05	CASP9/CASP	7
hsa04623	Cytosolic DN	7/127	63/8087	5.32E-05	0.00013075	4.23E-05	RELA/IKBKI	7

hsa04060	Cytokine-cyto	15/127	295/8087	5.51E-05	0.00013403	4.34E-05	TNFSF15/IL1	15
hsa04664	Fc epsilon RI	7/127	68/8087	8.74E-05	0.00021022	6.80E-05	PRKCA/AKT	7
hsa04020	Calcium signa	13/127	240/8087	9.44E-05	0.00022394	7.25E-05	ADRB2/PRKC	13
hsa04920	Adipocytokin	7/127	69/8087	9.60E-05	0.00022394	7.25E-05	RELA/IKBKI	7
hsa05211	Renal cell car	7/127	69/8087	9.60E-05	0.00022394	7.25E-05	JUN/AKT1/V	7
hsa04935	Growth horm	9/127	119/8087	9.88E-05	0.00022817	7.38E-05	PRKCA/AKT	9
hsa01523	Antifolate res	5/127	31/8087	0.00010809	0.00024721	8.00E-05	RELA/IKBKI	5
hsa04061	Viral protein	8/127	100/8087	0.00016471	0.00037302	0.00012069	IL10/IL6/CCI	8
hsa05330	Allograft reje	5/127	38/8087	0.00029243	0.00065584	0.00021219	IL10/IL2/IFN	5
hsa05204	Chemical carc	7/127	83/8087	0.000308	0.00068411	0.00022134	PTGS2/CYP3	7
hsa05020	Prion disease	13/127	273/8087	0.00033832	0.0007443	0.00024081	BAX/CASP9/	13
hsa04726	Serotonergic	8/127	115/8087	0.00043029	0.0009377	0.00030338	PTGS1/PTGS	8
hsa04540	Gap junction	7/127	88/8087	0.0004414	0.00094894	0.00030702	PRKCA/DRD	7
hsa04015	Rap1 signalin	11/127	210/8087	0.00044366	0.00094894	0.00030702	PRKCA/AKT	11
hsa05332	Graft-versus-l	5/127	42/8087	0.00047136	0.00099602	0.00032225	IL6/IL1B/IL2	5
hsa04929	GnRH secreti	6/127	64/8087	0.0004743	0.00099602	0.00032225	PRKCA/AKT	6
hsa04024	cAMP signali	11/127	216/8087	0.00056284	0.00117131	0.00037896	ADRB2/JUN/	11
hsa04137	Mitophagy - a	6/127	68/8087	0.00065793	0.00135697	0.00043903	JUN/RELA/M	6
hsa04110	Cell cycle	8/127	124/8087	0.00071205	0.00145561	0.00047094	CCNA2/GSK3	8
hsa04217	Necroptosis	9/127	159/8087	0.00085759	0.00173775	0.00056223	BCL2/BAX/C	9
hsa04914	Progesterone-	7/127	100/8087	0.00095296	0.0019142	0.00061931	PGR/AKT1/M	7
hsa04672	Intestinal imn	5/127	49/8087	0.00096798	0.00192761	0.00062365	IL10/IL6/IL2	5
hsa04728	Dopaminergi	8/127	132/8087	0.00107296	0.00211841	0.00068538	PRKCA/AKT	8
hsa04928	Parathyroid h	7/127	106/8087	0.00134259	0.00260474	0.00084273	BCL2/PRKCA	7
hsa05203	Viral carcino	10/127	204/8087	0.00134531	0.00260474	0.00084273	BAX/JUN/CA	10
hsa00980	Metabolism o	6/127	78/8087	0.00135908	0.00260474	0.00084273	CYP3A4/CYI	6
hsa04371	Apelin signal	8/127	137/8087	0.00136439	0.00260474	0.00084273	NOS2/AKT1/	8
hsa04923	Regulation of	5/127	57/8087	0.00192341	0.00364187	0.00117828	PTGS1/PTGS	5
hsa04670	Leukocyte tra	7/127	114/8087	0.00204453	0.00383973	0.00124229	PRKCA/ICAM	7
hsa04610	Complement	6/127	85/8087	0.0021169	0.00394357	0.00127589	PLAU/F7/F3/	6
hsa04611	Platelet activa	7/127	124/8087	0.0032882	0.0060766	0.001966	PTGS1/AKT1	7
hsa04310	Wnt signaling	8/127	160/8087	0.00360862	0.00661581	0.00214045	JUN/PRKCA/	8

hsa04940	Type I diabet	4/127	43/8087	0.00445944	0.00811126	0.00262429	IL1B/IL2/IFN	4
hsa05168	Herpes simple	16/127	498/8087	0.0047764	0.00861991	0.00278885	BCL2/BAX/C	16
hsa04520	Adherens jun	5/127	71/8087	0.0050248	0.0089979	0.00291115	EGFR/MAPK	5
hsa00982	Drug metabol	5/127	72/8087	0.00533319	0.00947666	0.00306604	CYP3A4/GST	5
hsa04140	Autophagy -	7/127	137/8087	0.00568445	0.00994779	0.00321847	BCL2/AKT1/	7
hsa04910	Insulin signal	7/127	137/8087	0.00568445	0.00994779	0.00321847	IKBKB/AKT1	7
hsa04550	Signaling path	7/127	143/8087	0.00715222	0.01242227	0.00401905	AKT1/MAPK	7
hsa04913	Ovarian stero	4/127	51/8087	0.00820258	0.01414027	0.00457489	PTGS2/CYP1	4
hsa04072	Phospholipase	7/127	148/8087	0.00857642	0.01467521	0.00474796	PRKCA/AKT	7
hsa05320	Autoimmune	4/127	53/8087	0.00938387	0.01593877	0.00515677	IL10/IL2/CD4	4
hsa04390	Hippo signali	7/127	157/8087	0.01165133	0.01964567	0.00635608	GSK3B/CCNI	7
hsa00480	Glutathione n	4/127	57/8087	0.01206681	0.02019879	0.00653504	GSTP1/GSTM	4
hsa05310	Asthma	3/127	31/8087	0.01233789	0.02050398	0.00663378	IL10/CD40L(	3
hsa04730	Long-term de	4/127	60/8087	0.01437193	0.02371369	0.00767223	PRKCA/MAP	4
hsa04114	Oocyte meios	6/127	129/8087	0.01578179	0.02585527	0.00836511	PGR/AR/PPP3	6
hsa04650	Natural killer	6/127	131/8087	0.01690913	0.02736267	0.00885281	CASP3/PRKC	6
hsa05217	Basal cell carc	4/127	63/8087	0.01693879	0.02736267	0.00885281	BAX/GSK3B/	4
hsa04640	Hematopoieti	5/127	99/8087	0.01953332	0.0313347	0.01013791	IL6/IL1B/IL1	5
hsa04720	Long-term pc	4/127	67/8087	0.02078259	0.03310881	0.0107119	PRKCA/PPP3	4
hsa00830	Retinol metab	4/127	69/8087	0.0228897	0.03596953	0.01163744	CYP3A4/CYI	4
hsa05031	Amphetamine	4/127	69/8087	0.0228897	0.03596953	0.01163744	JUN/PRKCA/	4
hsa04723	Retrograde er	6/127	148/8087	0.02879974	0.04495095	0.01454325	PTGS2/PRKC	6
hsa04261	Adrenergic si	6/127	150/8087	0.03048677	0.04726473	0.01529185	ADRB2/BCL2	6
hsa03320	PPAR signalii	4/127	76/8087	0.0312641	0.04814671	0.0155772	PPARG/MMI	4
hsa04725	Cholinergic sy	5/127	113/8087	0.03229188	0.04940016	0.01598274	BCL2/PRKCA	5
