# Supplementary figures and images for: Elucidation of the Underlying Mechanism of Gujian Oral Liquid Acting on Osteoarthritis through Network Pharmacology, Molecular Docking, and Experiment
Source: Biomed Res Int. 2022 Jul 28;2022:9230784. doi: 10.1155/2022/9230784 (PMC9352474; doi:10.1155/2022/9230784)

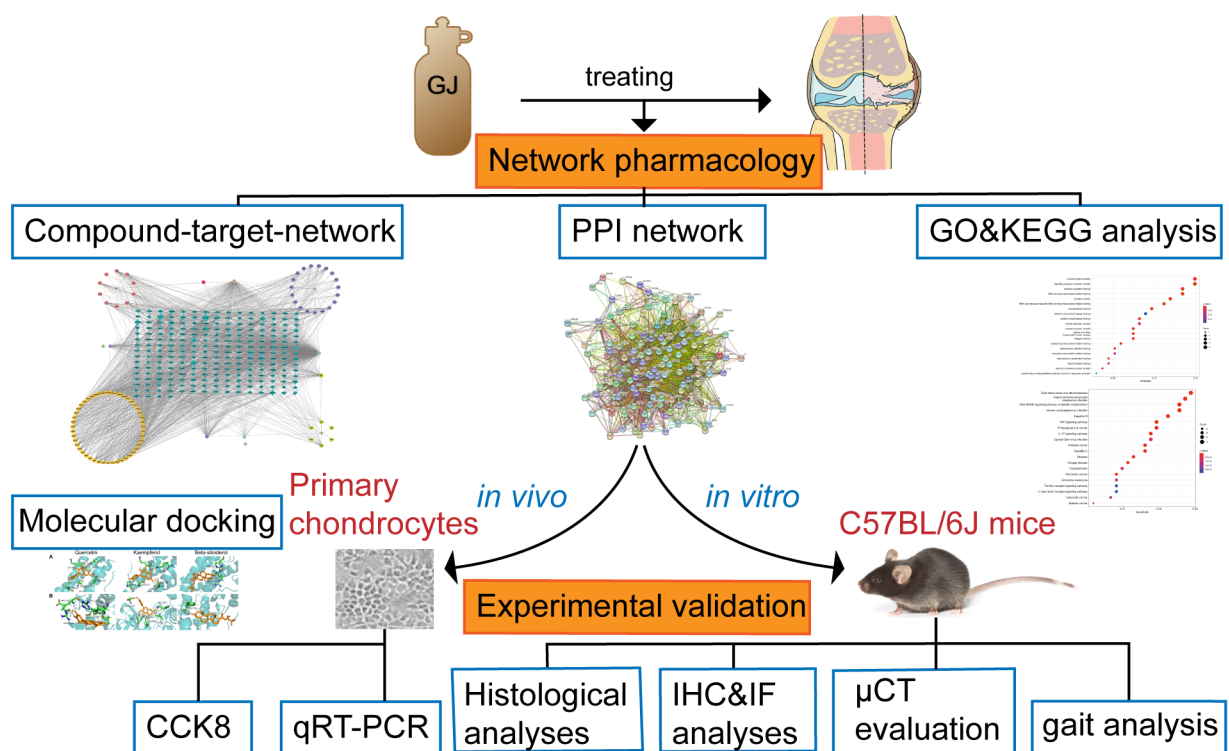

Supplement: Supplementary Materials — Table S1: ingredients of each herb contained in GJ oral liquid (OB ≥ 30%, DL ≥ 0.18). Table S2: known therapeutic targets correspond to the active ingredients. Table S3: the target protein corresponds to the gene name from UniProt. Table S4: known therapeutic targets for KOA. Table S5: the overlapping gene symbols between disease (osteoarthritis) and drug (GJ oral liquid). Supplementary Table S6: details of the active ingredients and the gene symbols. Supplementary Table S7: the top ten potentially effective compounds and the docking compounds in the prescription. Table S8: details of the PPI network. Table S9: key targets in the network. Table S10: details of GO enrichment analyses. Table S11: results of molecular docking. Table S12: details of KEGG pathway enrichment analyses. [file 9230784.f1.zip › Graphical abstract.pdf]
